# Photoenolization of *α,β*-Unsaturated Esters Enables Enantioselective Contra-Thermodynamic Positional Isomerization to α-Tertiary *β,γ*-Alkenyl Esters

**DOI:** 10.1021/jacs.4c15732

**Published:** 2025-02-24

**Authors:** Kuei-Chen Chang, Hung-Hsuan Chiu, Pin-Gong Huang, Shinje Miñoza, Wen-Hsuan Lee, Prem Kumar Keerthipati, Sasirome Racochote, Yi-Hua Lee, Chih-Ju Chou, Che-Ming Hsu, Che-Wei Chang, Darunee Soorukram, Cheng-chau Chiu, Hsuan-Hung Liao

**Affiliations:** † Department of Chemistry, National Sun Yat-sen University, Kaohsiung 80424, Taiwan (R.O.C.); ‡ Department of Chemistry and Center of Excellence for Innovation in Chemistry (PERCH−CIC), Faculty of Science, Mahidol University, Bangkok 10400, Thailand; § Department of Applied and Medicinal Chemistry, Kaohsiung Medical University, Kaohsiung 80708, Taiwan (R.O.C.)

## Abstract

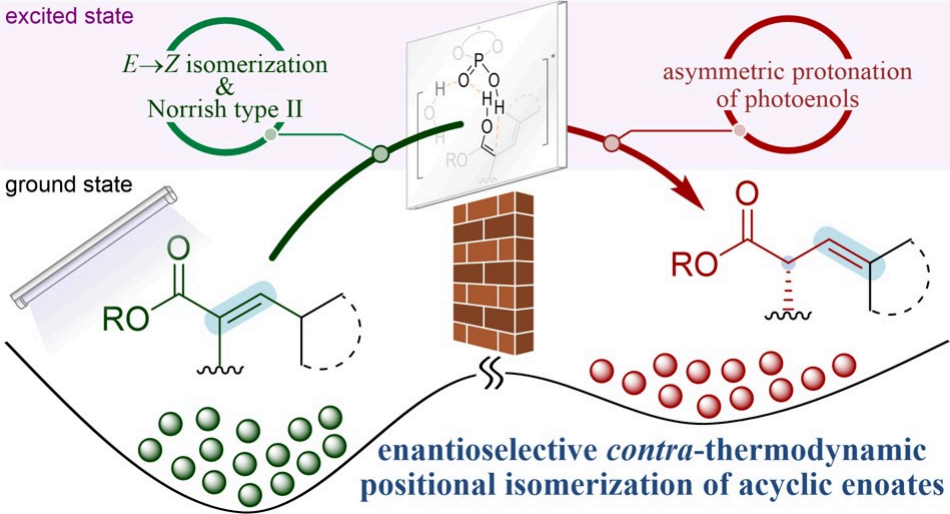

The enantioselective protonation of prochiral enolates is an ideal and straightforward platform to synthesize stereodefined α-tertiary esters, which are recurring motifs in a myriad of biorelevant molecules and important intermediates thereof. However, this approach remains onerous, particularly when dealing with α-unactivated esters and related acids, as enantioinduction on the nascent nucleophile necessitates peremptory reaction conditions, thus far only achieved via preformed enolates. A complementary and contra-thermodynamic catalytic strategy is herein described, where a transient photoenol, in the form of a ketene hemiacetal, is enantioselectively protonated with a chiral phosphoric acid (CPA). The prochiral photoketene hemiacetals are procured from excited *α,β*-unsaturated esters, specifically from the *Z*-geometric isomer through [1,5]-hydride shift as a chemically productive nonradiative relaxation pathway. Tautomerization via formal 1,3-proton transfer in the photoketene hemiacetal with CPA as a proton shuttle delivers α-branched *β,γ*-alkenyl esters in good to excellent yields and enantioselectivity under mild conditions. Furthermore, the current protocol was coupled to functional group interconversion experiments, as well as in a formal total synthesis of a known marine γ-butyrolactone-type metabolite. Performing the reaction in a continuous photoflow setup also enabled a gram-scale synthesis of a *β,γ*-alkenyl ester with up to 92% *ee*.

## 1 Introduction

**Scheme 1:**
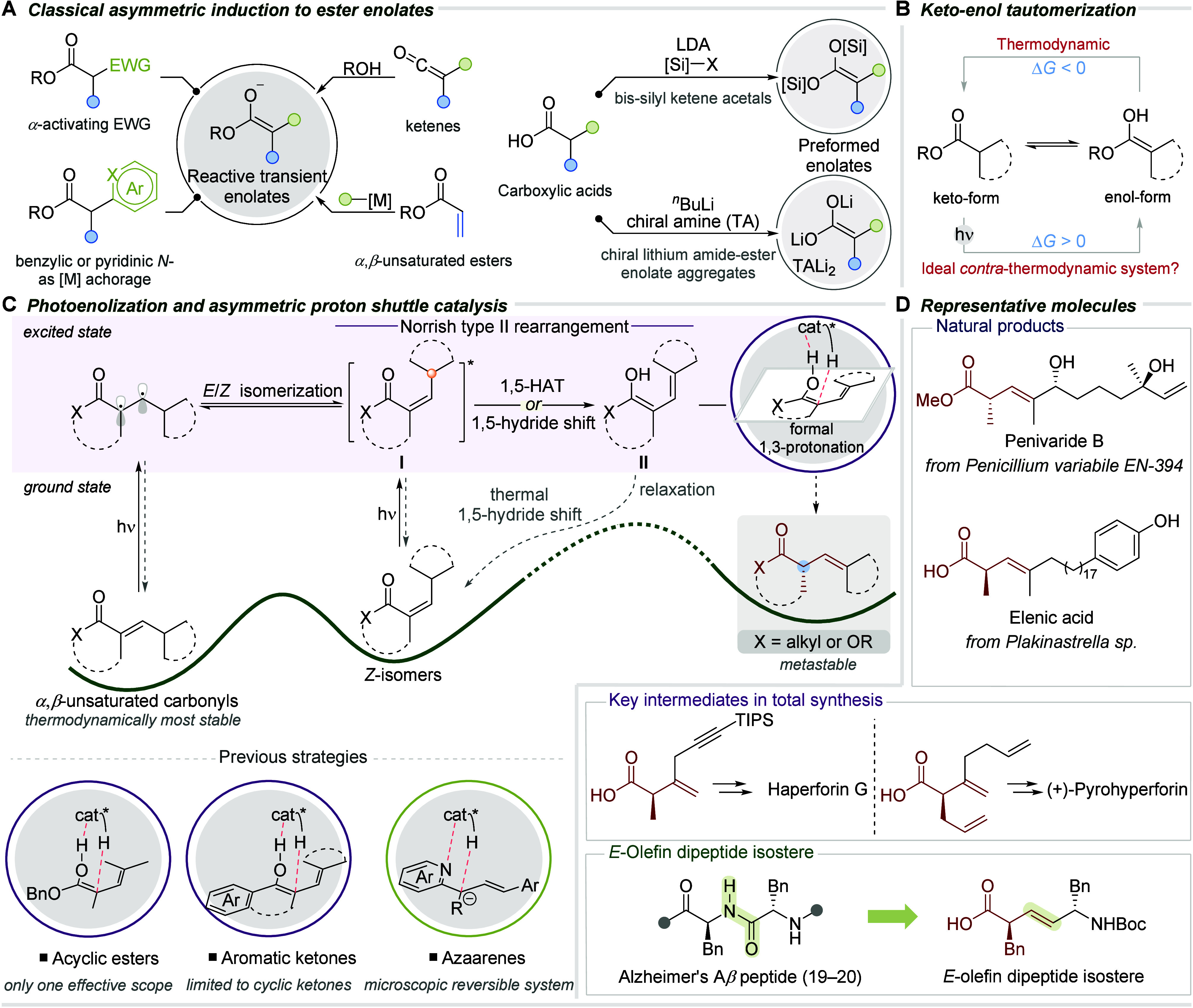
Asymmetric Synthesis of α-Branched Esters: (A) Classical Enolate Chemistry in Asymmetric Synthesis; (B) Thermodynamic versus Light-Driven Contra-Thermodynamic Keto-Enol Tautomerization of Unactivated Acyclic Esters; (C) Photoenolization of *α,β*-Unsaturated Esters and Subsequent Asymmetric Proton Shuttle Catalysis for Positional Isomerization to α-Tertiary *β,γ*-Alkenyl Esters; (D) Representative Molecules Bearing the Stereodefined α-Branched Alkenyl Esters

The chemistry of carbonyls is a classical synthetic paradigm extensively implicated in asymmetric synthesis. Stereodefined α-tertiary branched carbonyls are recurring motifs in a myriad of biorelevant molecules in addition to being important key intermediates for their preparation.^1,2^ α-Tertiary stereocenters in carbonyls are introduced through textbook enolate chemistry, and catalytic symmetry breaking often depends on π-facial selective C–C or C–H bond formation in the nascent prochiral enolate.^3−5^ This orthodox approach caters to carbonyl substrates of varying oxidation states, but unactivated acyclic esters as pronucleophiles remain underused in asymmetric synthesis, arguably due to the intrinsic high p*K*_a_ value of esters relative to other carbonyl derivatives, as well as the instability and short lifetimes of the thermodynamically unfavorable enol tautomer, enediol, or ketene hemiacetal. These hindrances were eluded by prior arts with the use of α-activating (electron-withdrawing groups) and anchoring groups in esters (Scheme 1A),^6−9^ especially α-amino and α-imino esters that are well-explored, to promote formation of enolates often by direct deprotonation or decarboxylation. Reactive transient enolates such as those from conjugate additions with organometallic reagents^10,11^ and 1,2-additions of ketenes,^12,13^ or preformed examples such as bis-silyl ketene acetals^14−18^ and the widely used chiral lithium amide-ester enolate aggregates^19^ have also been invoked as complementary routes to α-chiral esters while avoiding directly dealing with unstable naked enol intermediates, aside from cross-coupling^20,21^ and chiral auxiliary strategies.^22−24^

The use of photons has recently emancipated endergonic and stereoediting reactions from fundamental kinetic and thermochemical barriers, namely the principle of detailed balance and negative entropy change, by decoupling the forward and reverse steps in the thermodynamic equilibrium into distinct potential energy surfaces.^25−27^ With this logic, defying the canonical ground-state keto–enol equilibrium through a photostationary state enriched with the prochiral ester-enol tautomer could prove to be a mild platform for unlocking unactivated acyclic esters as pronucleophiles (Scheme 1B). We identified the photodeconjugation of *α,β*-unsaturated esters into *β,γ*-ones, as pioneered by Pete and co-workers, as a viable system (Scheme1C).^28−30^ Through *E/Z* photoisomerization of the *α,β*-unsaturated ester, re-excitation of the *Z*-isomer marshals a 1,5-hydrogen atom transfer (HAT) or a 1,5-sigmatropic hydride shift, which produces a transient photoketene hemiacetal. Nonradiative relaxation of the obtained reactive species is predicated by two orbital-symmetry allowed sigmatropic rearrangements: through (*i*) thermal [1,5]-hydride shift, reverting to the *α,β*-unsaturated ester, or (*ii*) photochemical [1,3]-hydride shift leading toward a photodeconjugated product, an α-tertiary *β,γ*-alkenyl ester, manifesting a net endergonic positional olefin isomerization. Of note, α-tertiary alkenyl esters are motifs found in natural products such as penivaride B and elenic acid and are used as key precursors for more complex architectures such as in haperforin G and pyrohyperforin.^31−34^ This motif is also a known isostere to phenylalanine dipeptide (Phe19–Phe20) in Alzheimer’s disease-related amyloid β peptide (Scheme 1D).^35^

Excited-state enantioselective protonation is well-explored in the literature, with most approaches hinging on the use of excited proton sources, called photoacids, while protonation of excited-state prochiral species is scarce in organic synthesis.^36^ In this context, previous efforts to procure enantiopure α-branched alkenyl esters via protonation of ester-derived photoketene hemiacetals were intensively examined by Pete’s group. The group managed to identify a bridged amino alcohol as a chiral proton shuttle to deliver high enantioselectivity for one example (91% *ee*), proceeding via a formal 1,3-proton transfer (tautomerization), a mechanistically distinct scenario from the [1,3]-hydride shift proposed for its uncatalyzed counterpart. Nevertheless, a comprehensive substrate scope and the promised synthetic utility of the transformation was not realized presumably due to the lack of generality and consistency in the enantiocontrol, being reliant on the interplay of catalyst and substrate control.^28,37^ Furthermore, the installation of a chiral auxiliary was necessary to ensure the practicality of the enantioselectivity.^38^ Protonation of photoenols from chromophoric aryl ketones was also reported with up to 90% *ee*; however, enantioinduction remains impaired for acyclic substrates.^39^ Tandem HAT/protonation as a distinct route to achieving positional isomerization in allylic azaarenes was shown to be plausible, with optical purities reaching up to 94%. The catalytic system is nonetheless subject to microscopic reversibility, so incomplete conversion must be accepted to limit the undesired loss of optical purity.^40^ At large, the photoactivation of esters and related acids,^41^ relative to ketones and aldehydes, and the asymmetric protonation of their corresponding ground-state enolates (transient or preformed) remains challenging. Specifically, previous protonation protocols required strict conditions such as cryogenic temperatures, slow reagent addition rates, and being limited to only electronically biased substrates.^4,5^ The List group, to this end, recently disclosed a highly enantioselective catalytic protonation/hydrolysis of bis-silyl ketene acetals en route to α-branched carboxylic acids, employing chiral disulfonimides at room temperature under protic conditions.^16^

## 2 Results and Discussion

**Figure 1:**
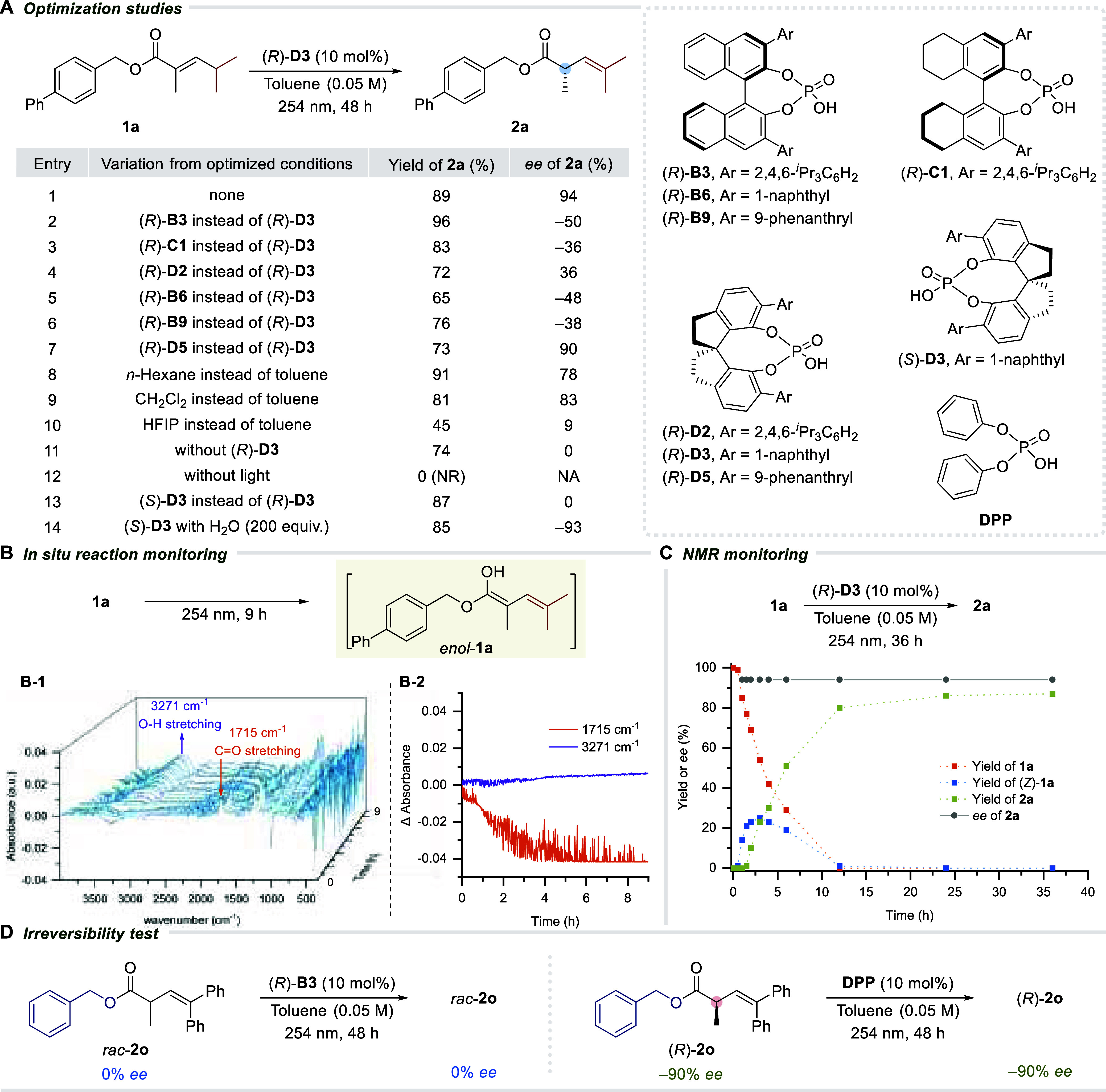
(A) Optimization studies. (B) Orthogonal in situ reaction monitoring with infrared spectroscopy. (C) NMR monitoring studies. (D) Probing microscopic reversibility.

In this work, we report a general enantioselective protonation of photoketene hemiacetals, as ester enolate analogs, as a viable complementary approach to synthesizing α-branched alkenyl esters. After a series of optimization runs using [1,1′-biphenyl]-4-ylmethyl (*E*)-2,4-dimethylpent-2-enoate (**1a**) as a model substrate, (*R*)-CPA **D3** was identified as the best catalyst for the enantioselective protonation of photoketene hemiacetal under UV-light irradiation in toluene (0.05 M) for 48 h (Figure 1A). This delivered an 89% yield of the photodeconjugated product **2a** in 94% *ee*. Among three CPA backbones initially tested, namely, (*R*)-BINOL (entry 2, **B3**), (*R*)-H_8_–BINOL (entry 3, **C1**), and (*R*)-SPINOL (entry 4, **D2**) containing 2,4,6-triisopropylphenyl pendant groups, (*R*)-BINOL CPA **B3** gave the highest *ee* (−50%), leading us to explore other BINOL-based CPAs. Disappointingly, neither 1-naphthyl (entry 5, (*R*)-**B6**) and 9-phenanthryl (entry 6, (*R*)-**B9**) increased the *ee* to desirable values. However, changing to SPINOL-CPA such as 9-phenanthryl containing (*R*)-**D5** afforded the target product in 90% *ee* (entry 7). Other nonpolar solvents such as *n*-hexane and CH_2_Cl_2_ can also provide amenable optical purities (78–83%, entries 8, 9), while a polar solvent such as HFIP only managed to produce 45% yield and 9% *ee*.

On the other hand, no enantioselectivity was observed in the absence of CPA as anticipated (entry 11), while product formation was inhibited in the absence of light (entry 12). To validate enantiodivergence, we changed (*R*)-**D3** to its (*S*)-CPA counterpart, and to our bewilderment, no optical activity was detected in the product despite the high yield (entry 13). We suspected that the (*R*)-CPA employed contained substantial moisture while a freshly opened bottle of (*S*)-CPA did not. Upon introduction of water (200 equiv) to the reaction, a –94% *ee* materialized (entry 14); a stark contrast to the previously reported sensitivity to protic solvents and impurities.^28−30^ Several reports accounted that water could perform varied roles in CPA-catalyzed asymmetric organic reactions, some of which include as an external proton source^42,43^ or substrate,^44^ as an ancillary species during enantioinduction as water bridges,^45^ or via the hydrophobic effect.^46,47^ Further mechanistic investigations should be conducted to ascertain the crucial role of water in the transformation under study and to uncover the origins of the enantioselectivity. Nevertheless, preliminary control experiments corroborate that water is essential for the enantioselectivity of the reaction (see Supporting Information, Section 6.8).

We then monitored the photoketene hemiacetal using in situ infrared spectroscopy monitoring (Figure 1B-1). The nascent ketene hemiacetal can be differentiated from the ester with the appearance of a distinctive broad O–H stretch peak at 3271 cm^–1^. The delay in the appearance of the O–H band, called the induction period, is attributed to the requisite *E/Z* isomerization of the olefin, preorganizing the γ-proton to the carbonyl moiety for the ensuing spontaneous [1,5]-hydride shift in a singlet state process.^48^ Steady-state formation of the excited intermediate is evident with continuous illumination of the system, albeit at minimal concentration (Figure 1B-2). A diminishing amount of excited ester over the first few hours is observed, corroborating the trend for ketene hemiacetal formation. The initial decreasing trend in IR absorbance for the C=O stretch at 1715 cm^–1^ may imply the conversion of the ester keto-form to its enol tautomer, shifting the equilibrium toward the enol, indicating a light-driven contra-thermodynamic keto–enol system.

The low steady-state concentration of the nascent prochiral photoketene hemiacetal is conducive to achieving high levels of enantioselectivity by minimizing unwanted background reaction during the π-facial selective proton transfer, which is also the turnover limiting step of the overall transformation (see KIE experiments, Supporting Information, Section 6.8).^39^ Nevertheless, inefficient enantioinduction was previously observed in the works of Pete as the chiral amino alcohol-substrate interaction is sensitive to solvent effects (protic and basic counterproductive interactions), aside from the limited solubility of the chiral catalyst in nonpolar solvents that is further amplified by the required lower temperatures.^28−30^ We opted to use chiral phosphoric acids (CPA) as proton shuttles in the hope of harnessing their robust dual activation mode capabilities. Using the identified *R*-CPA (**D3**) (see optimization studies, Supporting Information, Section 3.1), complete control of the π-facial proton transfer throughout the course of the reaction was achieved (Figure 1C). ^1^H NMR monitoring also showed *E/Z* geometric isomerization of the *α,β*-unsaturated ester, with the *Z*-isomer yield peaking at 23% at the 3 h mark, in accordance with the starting point of the photostationary state. The consistent *ee* exhibited suggests that the product formed is not influenced by microscopic reversibility. Control experiments using product **2o** with (*R*)-**B3** or **DPP** showed no back reaction or decrease in optical purity under standard conditions (Figure 1D).

**Scheme 2:**
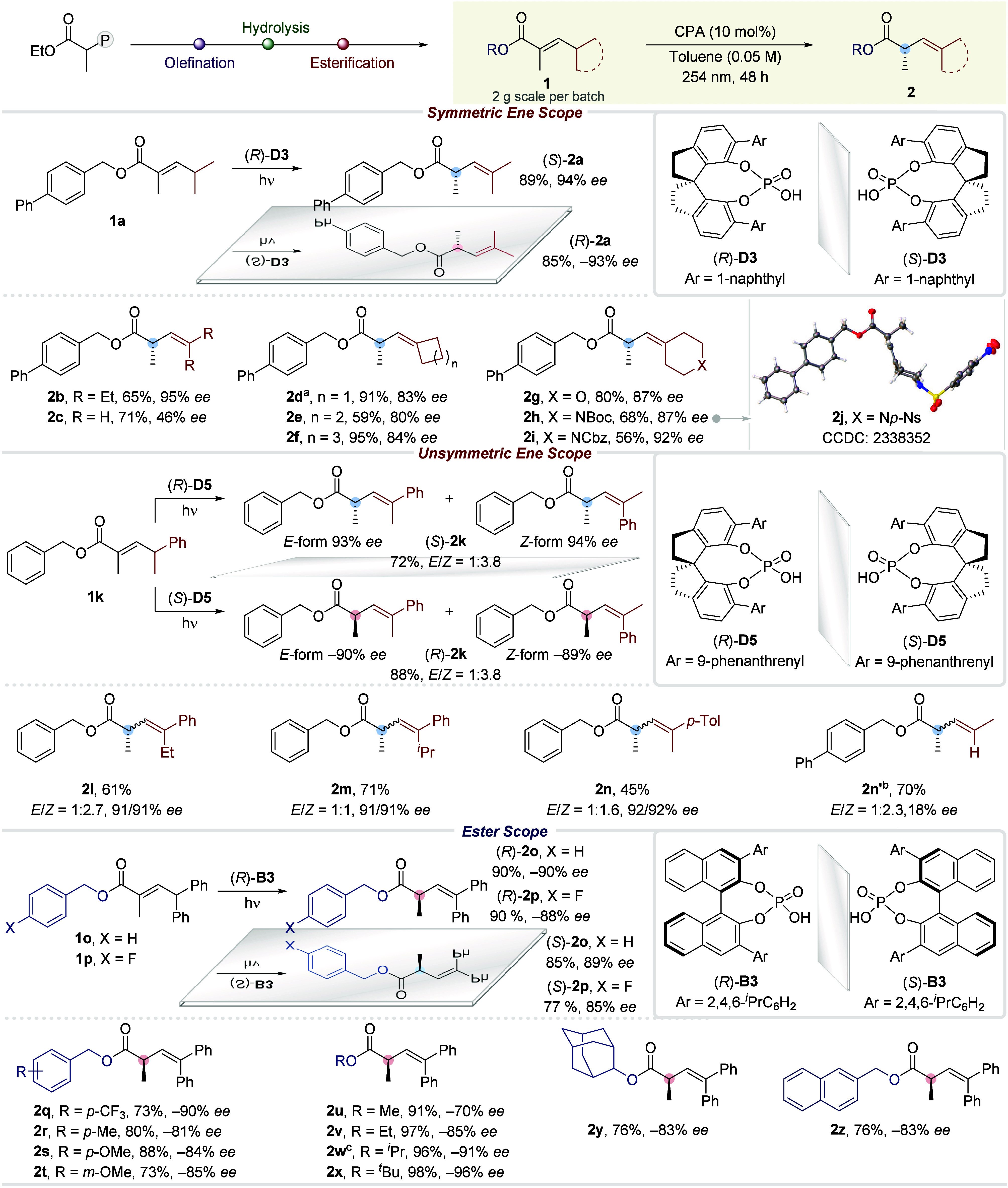
Substrate Scope (*R*)-**D5**. (*R*)-**D3**. (*R*)-**C1** as the catalyst. The reactions were performed on a 0.05–0.10 mmol scale, and all reported yields were of isolated material after purification.

After a series of optimization experiments, gram-scale preparations of the starting materials were carried out via standard Wittig or Horner-Wadsworth-Emmons (HWE) olefination, followed by hydrolysis/esterification to diversify the alkoxy substituent. The scope for the asymmetric protonation of photoketene hemiacetals obtained from the contra-thermodynamic positional olefin isomerization in *α,β*-unsaturated esters was evaluated (Scheme 2), starting with variations in the β-substituents across the double bond. Alkyl groups such as isopropyl (**1a**), 3-pentyl (**1b**), and cycloalkyls (**1d** to **1f**) afforded excellent enantioselectivities while methyl (**1c**) gave a diminished *ee* (46%). The corresponding enantiomer of **1a** can also be prepared with the use of (*S*)-**D3** in a similar yield and −93% *ee*. Heteroalicyclic rings presented a similar performance; among those tested were 4-pyranyl (**1g**) and *N*-protected 4-piperidinyls (**1h**, **1i**). The absolute configuration of all products obtained under standard conditions, utilizing (*R*)-**D3**, was conjectured from the crystal structure of sulfone **2j**, derived from **2h**, established to possess the (*S*)-configuration.

Enantioenrichment of γ-racemic *α,β*-unsaturated esters was also demonstrated using (*R*)-CPA **D5** as the identified optimal catalyst (see Supporting Information, Section 3.2). This provided optically enriched α-tertiary esters (**2k** to **2n**) with *E/Z* ratios and *ee* values of up to 1:3.8 and 94%, respectively. γ-Methyl alkenyl ester (**1n′**) was also tolerated under our conditions, providing 70% yield of **2n′** in a 1:2.3 *E/Z* ratio and a meager 18% *ee*.

Similar *E/Z* distribution was also observed upon switching the catalyst to (*S*)-**D5**, offering around −90% *ee* for the photodeconjugated product (*R*)-**2k**. The *E/Z* ratios can be further manipulated to favor the *Z*-isomer through extended light exposure of the product mixture (see Supporting Information, Section 6.7).

Next, variation in the ester group was evaluated using (*R*)-**B3**, which came out as the most optimal catalyst for this set of substrates (see Supporting Information, Section 3.3). Changing the ester group to other *p*-substituted benzyloxy derivatives (**1o** to **1s**) was well tolerated under our conditions regardless of the electronic nature of the *p*-substituent (-H, -F, −CF_3_, -Me, and -OMe). Moving the methoxy group from *p*- to *m*-position (**1t**) was permissible, but *o*-OMe disrupted the reaction (see Supporting Information, Table S4.4, for other unsuccessful examples). Alkoxy groups (**1u** to **1x**) were investigated as well, and the enantioselectivity increased when moving from less hindered appendages, such as methyl (**1u**) and ethyl (**1v**), to more bulky appendages, namely isopropyl (**1w**) and *t*-butyl (**1x**). Adamantyl (**1y**) and naphthalen-2-ylmethoxy (**1z**) also participated in the reaction with an efficiency analogous to that of previous examples. In general, π–π stacking interactions between the ester and the CPA catalyst was not a prerequisite for efficient enantioinduction since nonaryl containing substrates exhibited similar enantioenrichment to aryl-containing ones. Using (*S*)-**B3** for the transformation of **2o** and **2p** gave their corresponding opposite enantiomers in analogous optical purities, as expected.

**Scheme 3:**
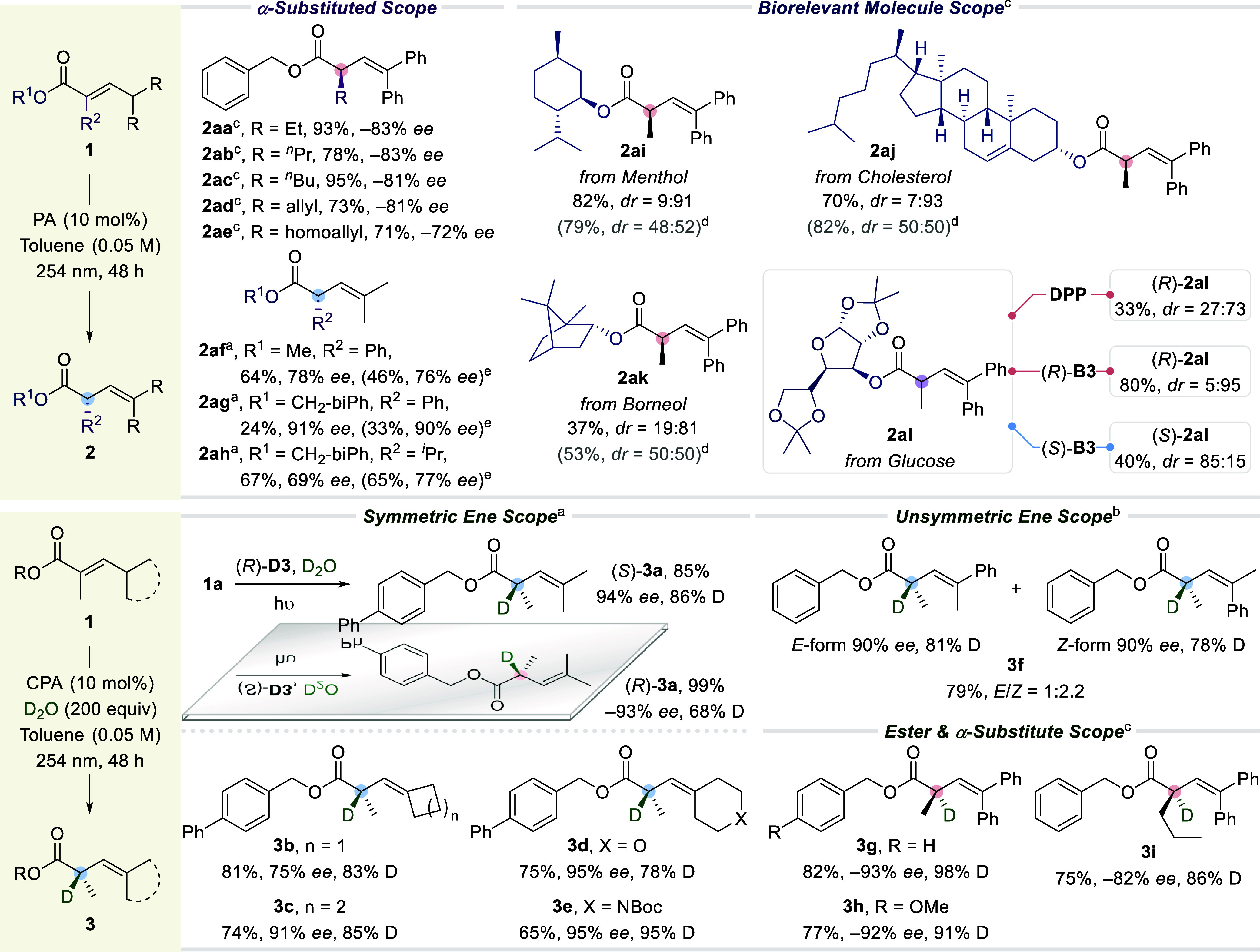
Substrate Scope—Continuation (*R*)-**D3**. (*R*)-**D5**. (*R*)-**B3**. DPP as the catalyst. Z-isomer as starting material. PA = phosphoric acid. The reactions were performed on a 0.05–0.10 mmol scale, and all reported yields were of isolated material after purification.

The α-branch in the substrates was also varied. Good product optical purities were exhibited for several linear alkyl chains (**1aa** to **1ad**), while a modest *ee* was obtained for homoallyl (**1ae**) (Scheme 3). Employing α-phenyl methyl ester (**1af**) gave 78% *ee* and its *Z*-isomer resulted in 76% *ee* albeit in a lower yield. On the other hand, α-phenyl (**2ag**) and α-isopropyl (**2ah**) methylnaphthalene esters were delivered in 90% and 77% *ee*, respectively, from *Z*-starting materials **1ag** and **1ah**.

Employing ester derivatives of biorelevant chiral alcohols such as menthol (**1ai**), cholesterol (**1aj**), and borneol (**1ak**) resulted in a highly diastereoselective positional isomerization compared to the achiral control reaction, wherein no selectivity was detected. In addition, a glucose derivative (**1al**) gave rise to substantial substrate control toward the diastereoselective protonation of the transient photoketene hemiacetal (33% yield, *dr* = 27:73). This result can be further elevated to a *dr* of 5:95 with 80% yield using the *R*-enantiomer of the catalyst, while employing (*S*)-**B3** yielded 40% with 85:15 *dr*, overriding the latent chiral substrate control of the installed glucose. Overall, these examples showcase the potential of asymmetric photodeconjugation in late-stage modifications of secondary metabolites.

Inspired by our deuteration experiments (see Supporting Information, Section 6.7), we performed α-deuteration on various substrates using D_2_O. Isopropyl (**3a**), cycloalkyls (**3b**, **3c**), tetrahydropyranyl (**3d**), and piperidinyl (**3e**) α-deuterated alkenyl esters were delivered in good to excellent yields with high D incorporations and *ee* of up to 95%. Enantioenrichment of γ-racemic substrates was realized as well, providing γ-methyl γ-phenyl methylidene (**3f**) in 90% *ee* (*E/Z* = 1:2.2) with 78–81% D content. Besides, benzyloxy (**3g**) and 4-methoxybenzyloxy (**3h**) afforded 98% and 91% D incorporation, while an α-propylated alkenyl ester (**3i**) furnished 86% D.

**Scheme 4:**
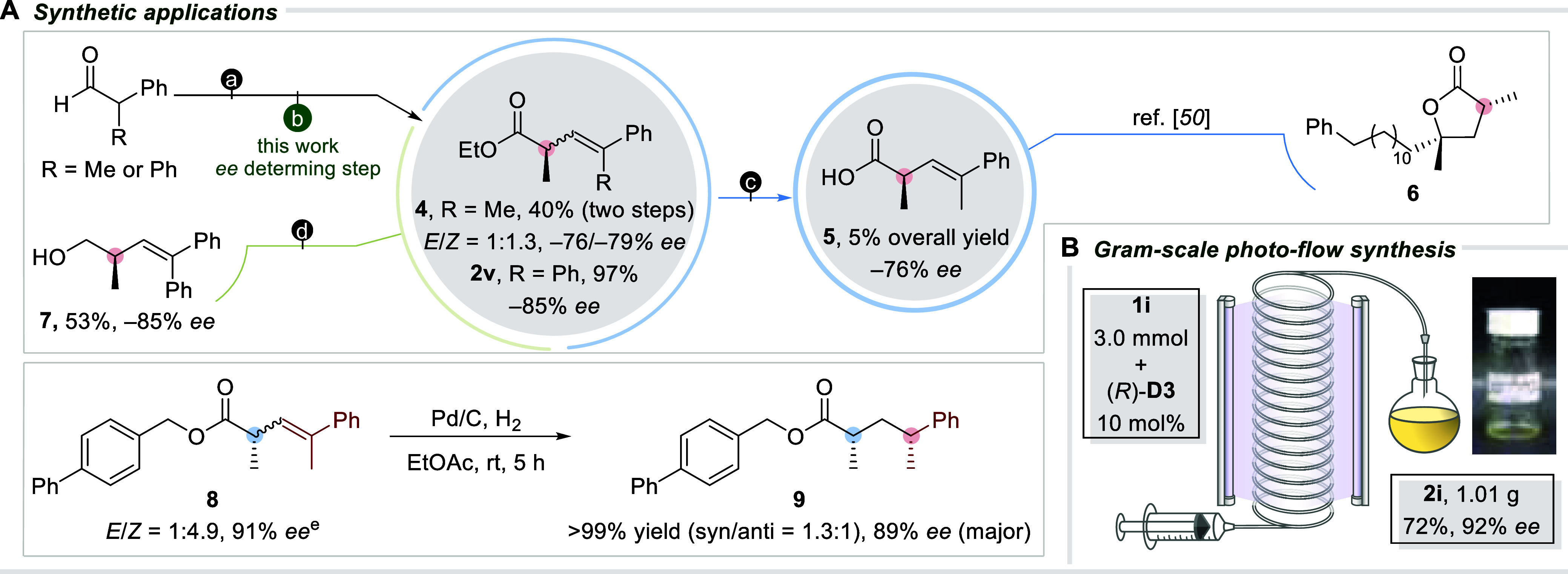
Synthetic Applications: (A) Functional Group Interconversion (FGI) and formal Synthesis of a Marine Butyrolactone-Type Natural Product. (B) Gram-Scale Photoflow Chemistry R = Me: ethyl 2-(triphenylphosphoranylidene) propanoate, DCM, rt; R = Ph: ethyl 2-(diethoxyphosphoryl) propanoate, DIPEA, LiCl, MeCN, rt. R = Me: (*S*)-**D5**, toluene, 254 nm; R = Ph: (*R*)-**B3**, toluene, 254 nm. LiOH, H_2_O_2_, THF/H_2_O, 0 °C to rt. DIBAL-H, DCM, −78 °C. *E*-isomer.

A formal synthesis of a marine butyrolactone-type metabolite **6** from the dark brown deep-water sponge *Plakortis nigra* was then executed (Scheme 4A).^49^ We sought to prepare Rousseau’s (*R*)-α-methyl alkenyl acid **5** as the key intermediate to procure the target metabolite.^50^ Starting from α-methyl aldehyde, photodeconjugated ethyl ester **4** was obtained in −76% *ee* and 1:1.3 *E/Z* ratio under standard conditions after securing the *α,β*-unsaturated ester via Wittig olefination. Hydrolysis of the ethoxy group and subsequent separation of the geometric isomers led to the isolation of Rousseau’s (*R*)-α-methyl alkenyl acid **5** in a 5% overall yield and −76% *ee* over three linear steps. In contrast to the previous report, Rousseau’s α-chiral acid intermediate **5** was procured via methylation of a *β,γ*-unsaturated acid with Evan’s oxazolidinone auxiliary preinstalled. Still, separation of the diastereomers was required via normal-phase liquid chromatography, as only a mild diastereoselectivity (60:40 *dr*) was manifested in their methylation step. Separation of the major diastereomer and subsequent hydrolysis affords the identified key intermediate, translating to 28% yield over 3 steps. Our current methodology, on the other hand, offers a more straightforward platform to obtain the desired chiral intermediate, albeit in a much lower yield, which could be further improved by optimization to efficiently shorten the total synthesis of the natural product of interest.

We also embarked on functional group interconversion (FGI) reactions. Reduction of enantiopure α-tertiary alkenyl ester **2v**, obtained from the HWE olefination of phenyl aldehyde and subsequent photodeconjugation under standard conditions, using DIBAL-H afforded primary chiral alcohol **7** in 53% yield with a −85% optical purity. Hydrogenation of alkenyl ester **8** (1:4.9 *E/Z* mixture) over Pd/C delivered saturated butanoate derivative **9** in 89% *ee* with a 1.3:1 *syn/anti* distribution. Interestingly, the hydrogenation product **9** is reminiscent of the 1,3-dimethyl deoxypropionate substructure that is often encountered in polyketide natural products.^51,52^ Aside from postfunctionalization, the current photodeconjugation method was also performed in photoflow conditions (Scheme 4B). This enabled the gram-scale synthesis of alkenyl ester **2i** in 92% *ee*.

## 3 Conclusion

This work presents a viable approach to preparing enantiopure α-branched *β,γ*-alkenyl esters via CPA-catalyzed enantioselective protonation of prochiral photoketene hemiacetals. The photoketene hemiacetal intermediates were derived from *α,β*-unsaturated esters through tandem *E/Z* geometric isomerization and [1,5]-hydride shift of *α,β*-unsaturated esters and underwent enantioselective formal 1,3-proton transfer (tautomerization) through the aid of a bifunctional CPA, acting as a proton shuttle, and water to permit efficient enantiocontrol in mild conditions to a diverse set of substrates. Moreover, it is anticipated that the developed protocol can be applied to the preparation of biorelevant molecules containing the α-branched *β,γ*-alkenyl ester motif, as demonstrated in the formal synthesis of a marine butyrolactone-type natural product.

This current approach leveraged two contra-thermodynamic processes: first, the ability to tilt the keto–enol equilibrium of esters in favor of the enol-tautomer, when employing *α,β*-unsaturated esters, using only light, and second, the possibility to procure a higher energy product, from a conjugated to deconjugated ester, resulting in a net endergonic transformation. Additionally, we herein showed that ultraviolet (UV) light remains an indispensable tool in the development of efficient photoreactions despite its current less fashionable status in the synthetic community.^53^

## Supporting Information Available

Detailed optimization data, experimental procedures, characterization data for all compounds, and DFT calculations (PDF)
Cartesian coordinates (ZIP)
NMR raw data (ZIP)
Crystallographic data (ZIP)
