# Supplementary material for: Photoenolization of α,β-Unsaturated Esters Enables Enantioselective Contra-Thermodynamic Positional Isomerization to α-Tertiary β,γ-Alkenyl Esters
Source: J Am Chem Soc. 2025 Feb 24;147(9):7452–60. doi: 10.1021/jacs.4c15732 (PMC11887454; doi:10.1021/jacs.4c15732)
Supplement: Supplementary file 1 — ja4c15732_si_001.pdf [file ja4c15732_si_001.pdf]

Supplementary Information

Photoenolization of *α,β*-Unsaturated Esters Enables Enantioselective Contra-Thermodynamic Positional Isomerization to *α*-Tertiary *β,γ*-Alkenyl Esters

Kuei-Chen Chang[1]†, Hung-Hsuan Chiu[1]†, Pin-Gong Huang[1], Shinje Miñoza[1], Wen-Hsuan Lee[1], Prem Kumar Keerthipati[1], Sasirome Racochote[2], Yi-Hua Lee[1], Chih-Ju Chou[1], Che-Ming Hsu[1], Che-Wei Chang[1], Darunee Soorukram[2], Cheng-chau Chiu[1], Hsuan-Hung Liao[1,3]*

[1]Department of Chemistry, National Sun Yat-sen University; Kaohsiung 80424, Taiwan (R.O.C.)

[2]Department of Chemistry and Center of Excellence for Innovation in Chemistry (PERCH-CIC), Faculty of Science, Mahidol University; Bangkok 10400, Thailand.

[3]Department of Applied and Medicinal Chemistry, Kaohsiung Medical University; Kaohsiung 80708, Taiwan (R.O.C.)

*Corresponding author. Email: hsuan-hung.liao@mail.nsysu.edu.tw

†These authors contributed equally to this work.

Back to contents

Contents

1. General Information 1
1.1 Pre-treatment of Experiments, Solvents and Reagents 1
1.2 Chromatography, Data Analysis, and Collection 1
1.3 Instrument Catalog 2
1.4 Purchasing Reagents 3
1.5 Summary Table of Synthesized Compounds 11
2. General Procedures 14
2.1 Synthesis of ethyl *α*,*β*-unsaturated esters 14
General Procedure A: Ethyl *α*,*β*-unsaturated esters from Wittig reaction 14
General Procedure B: Ethyl *α*,*β*-unsaturated esters from Horner-Wadsworth-Emmons reaction 21
2.2 General Procedure C: Synthesis of *α*,*β*-unsaturated carboxylic acids 28
2.3 General Procedure D: Synthesis of photodeconjugation precursors 43
2.4 General Procedure E: Photodeconjugation of *α*,*β*-unsaturated esters 43
2.5 General Procedure F: Synthesis of *α*-deuterated *α*,*β*-unsaturated esters 43
3. Optimization Studies 44
3.1 Photodeconjugation of symmetric *ene* scopes 44
3.2 Photodeconjugation of unsymmetric *ene* scopes 46
3.3 Photodeconjugation of ester scopes 47
4. Overview of Substrate Scope 49
4.1 Characterization data of compound **1** 54
4.2 Characterization data of compound **2** 98
4.3 Characterization data of compound **3** 138
4.4 Characterization data of compound **S5** 148
5. Synthetic Application 150
5.1 Functionalization group interconversion and formal synthesis 150
5.2 Gram-scale photoflow chemistry 157
6. Mechanism Studies 158
6.1 Orthogonal *in situ* reaction monitoring with infrared spectroscopy 158
6.2 NMR monitoring studies 158
6.3 Light on-off experiments 160
6.4 Reversible test of the *E*/*Z* isomerization 161
6.5 Irreversible test of the keto-enol tautomerization 162
6.6 Control experiments 162

Back to contents

Background reaction test ... 162
Effect of oxygen ... 163
6.7 *E*/*Z* isomerization of unsymmetric product ... 163
6.8 Effect of water ... 164
Effect of water for (*S*)-**D3** ... 164
Correlation of water loading to *ee* ratio ... 166
Isotope labelling experiment ... 166
Alcohol as external proton source ... 167
KIE experiment of 1,5-HAT step ... 168
KIE experiment of formal 1,3-protonation step ... 169
DFT calculation ... 169
7. X-ray ... 201
8. References ... 220
9. NMR Spectra ... 222
10. HPLC Spectra ... 351

1. General Information

1.1 Pre-treatment of Experiments, Solvents and Reagents

Glassware All glassware has been oven-dried after cleaning with the washing machine Steelco LAB 500CL.

Solvents All solvents were purchased from suppliers (Table S1.2 to S1.4). Unless otherwise stated, the following ACS-grade solvents (acetonitrile, dichloromethane, diethyl ether, 1,4-dioxane, *N*,*N*-dimethylformamide, tetrahydrofuran, toluene) were stored over microwave-activated 3Å molecular sieves for at least one night and transferred into dry engineering alumina column drying system (Vigor Gas Purification Technologies Co., Ltf, VSPS-7) prior to use.

Chemicals The Purification of Laboratory Chemicals (ISBN: 978-1-85617-567-8) prior to use. To maintain the activity, highly sensitive compounds such as metal catalysts and ligands were opened and stored in the glovebox. Other chemicals were directly used as received and were always filled with nitrogen before storage and twined with parafilm carefully. See section S1.4 for the complete supplier list of each chemical. All the names of the chemicals listed herein were generated from ChemBioDraw Ultra 14.0.

Experiment Except as specified, air- and water-sensitive reactions were carried out in nitrogen-filled glovebox (Vigor SG1200/750TS-F) or standard Schlenk techniques.

1.2 Chromatography, Data Analysis and Collection

Thin-layer chromatography (TLC) Merck aluminium-backed sheets coated with $60F_{254}$ silica gel. Visualization of the silica plates was achieved using a UV lamp (λ_{max} = 254 nm), and/or PMA stain (10% PMA in 95% EtOH), CAM stain (2.5% ammonium molybdate tetrahydrate, 1% cerium ammonium sulfate dihydrate in water with sulfuric acid), aqueous solution of $KMnO_4$, ninhydrin solution, aqueous solution of DNP, or *p*-anisaldehyde solution followed by heating.

Silica gel column chromatography Silica gel column chromatography was carried out using KM3 scientific silica gel (45 – 75 μm) purchased from KM3 Scientific.

Flash chromatography instrument Flash chromatography was carried out using Biotage® Selekt. Before loading the crude product, thin-layer chromatography (TLC) was used to confirm the polarity of the predicted product. Visualization was achieved by UV light or staining with an aqueous solution of $KMnO_4$, PMA solution, ninhydrin solution, aqueous solution of DNP, *p*-anisaldehyde solution, or CAM solution followed by heating.

1.3 Instrument Catalog

Nuclear magnetic resonance spectroscopy (NMR) ^{1}H-, ^{13}C- and ^{19}F-Nuclear Magnetic Resonance (NMR) spectra were used to identify the structure of starting materials and products. The spectra were recorded on Bruker Avance 300 MHz, Jeol ECZS 400 MHz, Bruker Avance 500 MHz, or Jeol ECZR 600 MHz. Coupling constants are abridged as follows: s = singlet, br.s = broad singlet, d = doublet, t = triplet, q = quartet, quin = quintet, sext = sextet, sept = septet, m = multiplet, dd = doublet of doublets.

Gas chromatography–Mass spectrometry (GC-MS) The operation method was set as follows: 1.0 mL sample was injected by auto-sampler in a split mode (100:1) with 0.5 mL air gap into the GC-MS system consisting of an Agilent 8860 gas chromatograph, an Agilent 5977B mass selective detector, and Agilent 7693A autoinjector. Gas chromatography was performed on a 30 m HP-5MS with 0.25 mm inner diameter (I.D.) and 0.25 mm film thickness with an initial injection temperature of 50 ºC to 300 ºC, MSD transfer line of 280 ºC, and the ion source adjusted to 230 ºC. The helium carrier gas was set at a constant flow rate of 1.197 ml min^{-1}. The mass spectrometer was operated in positive electron impact mode (EI), with ionization energy in the *m/z* 50 – 550 scan range. The spectra of all chromatogram peaks were evaluated using the MSD Chemstation.

High-performance liquid chromatography (HPLC) The enantiomeric ratio was determined by Agilent 1260 Infinity series using Daicel Chiralpak analytical column (4.6 mm × 250 mm) IA-3, IC-3, OD-3, or OJ-3. All runs were performed in isocratic condition at 20 ºC. Sample solutions for semipreparative separation (approximately 1.0 mg/mL) were prepared by dissolving the analytes in the eluent and filtering through 0.45 µm Acrodisc syringe filters. The injection volume was 5 – 10 µL, and the eluent used was a mixture of HPLC *n*-hexane/isopropanol. UV absorption was monitored at 254 nm.

High resolution mass spectrometry (HRMS) High resolution mass measurement was performed on a Jeol AccuTOF GCx-plus / Shimadzu QP2020.

Optical rotation ($[\alpha]_D^T$) Optical rotation was measured on a JASCO P-2000 Digital Polarimeter.

Photoreactor The photoreaction was carried out using a Panchum Photochemical Reactor PR-2000 with a 254 nm Hg lamp.

Back to contents

Table S1.1 Supplier of equipment and instruments.

Equipment	Supplier and Product model
Electronic balance	Shimadzu, UW2200H/ATX224
Hot plate stirrer	Corning, PC-420D
Immersion cooler	Panchum, IC-9090
Pump of the rotary evaporator	KNF Laboport, N820.3FT.18
Refrigerated circulator bath	Panchum, CC-300
Rotary evaporator	Heidolph, Hei-Vap Core HL G3
Ultra-low temp. reaction bath	Panchum, UR-8500
Vacuum pump	Edwards, RV5
Instruments	Supplier and Product model
FT-IR spectrophotometer (Fourier Transform-Infrared spectrophotometer)	PerkinElmer, Spectrum Two
High-Performance Liquid Chromatography (HPLC)	Agilent, 1260 Infinity II
Gas Chromatograph-Mass Spectrometer (GC-MS)	Agilent, 5977B
Glovebox	Vigor, SG1200/750TS-F
Melting point apparatus	RY-1G
Polarimeter	JASCO, P-2000
Photoreactor	Panchum, PR-2000

1.4 Purchasing Reagents

Table S1.2 Supplier of solvents.

Solvent	Supplier	Solvent	Supplier
ACS Acetone	Duksan	ACS Hexane	Duksan
ACS Acetonitrile	J.T. Baker	ACS Methanol	Macron
ACS Benzene	Echo	ACS Tetrahydrofuran	Macron
ACS Chloroform	Acros	ACS Toluene	Echo
ACS Dichloromethane	Macron	HPLC Acetonitrile	J.T. Baker
ACS Diethyl ether	Duksan	HPLC *N*,*N*-Dimethylformamide	Macron
ACS Dimethyl sulfoxide	UR	HPLC Ethyl acetate	Merck
ACS Ethanol	J.T. Baker	HPLC Hexane	Echo

Back to contents

ACS Ethyl acetate	Macron	HPLC Isopropanol	Echo

Table S1.3 Supplier of solvents in solvent purification system.

Solvent	Supplier	Solvent	Supplier
Acetonitrile	J.T. Baker	1,4-Dioxane	J.T. Baker
Dichloromethane	Macron	Tetrahydrofuran	Macron
N,*N*-Dimethylformamide	Macron	Toluene	J.T. Baker
Diethyl ether	Echo		

Table S1.4 Supplier of deuterated solvents.

Solvent	Supplier	Solvent	Supplier
Acetonitrile-d_3	Sigma-Aldrich	Dimethyl sulfoxide-d_6	Sigma-Aldrich
Benzene-d_6	Sigma-Aldrich	Dichloromethane-d_2	Sigma-Aldrich
Chloroform-d_1	Merck	Methanol-d_4	Sigma-Aldrich
Water-d_2	Sigma-Aldrich	Toluene-d_8	Sigma-Aldrich

Table S1.5 Supplier of chiral reagents.

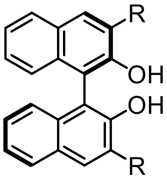

No.	R group	Name	Supplier / CAS#
(*R*)-**A1**	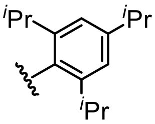	(*R*)-3,3'-Bis(2,4,6-triisopropylphenyl)-[1,1'-binaphthalene]-2,2'-diol	BLD<br>247123-09-7

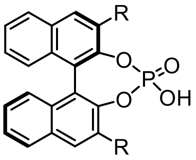

No.	R group	Name	Supplier / CAS#
(*R*)-**B1**	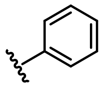	(11b*R*)-4-Hydroxy-2,6-diphenyldinaphtho[2,1-d:1',2'-f][1,3,2]dioxaphosphepine 4-oxide	Suifu<br>695162-86-8
(*R*)-**B2**	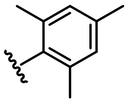	(11b*R*)-4-Hydroxy-2,6-dimesityldinaphtho[2,1-d:1',2'-f][1,3,2]dioxaphosphepine 4-oxide	Suifu<br>695162-87-9

(*R*)-**B3**	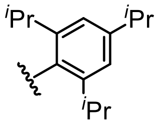	(11b*R*)-4-Hydroxy-2,6-bis(2,4,6-triisopropylphenyl)dinaphtho[2,1-d:1',2'-f][1,3,2]dioxaphosphepine 4-oxide	Suifu<br>791616-63-2
(*R*)-**B4**	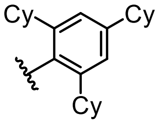	(*R*)-4-Hydroxy-2,6-bis(2,4,6-tricyclohexylphenyl)dinaphtho[2,1-d:1',2'-f][1,3,2]dioxaphosphepine 4-oxide	Suifu<br>1359764-39-8
(*R*)-**B5**	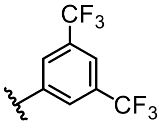	(*R*)-3,3'-Bis[3,5-bis(trifluoromethyl)phenyl]-1,1'-binaphthyl-2,2'-diyl hydrogen phosphate	Suifu<br>791616-62-1
(*R*)-**B6**	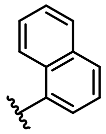	(*R*)-3,3'-Bis(1-naphthalenyl)-1,1'-binaphthyl-2,2'-diyl hydrogen phosphate	Suifu<br>864943-23-7
(*R*)-**B7**	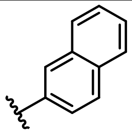	(*R*)-3,3'-Bis(2-naphthalenyl)-1,1'-binaphthyl-2,2'-diyl hydrogen phosphate	Suifu<br>791616-56-3
(*R*)-**B8**	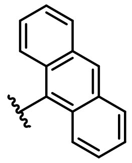	(11b*R*)-2,6-Di(anthracen-9-yl)-4-hydroxydinaphtho[2,1-d:1',2'-f][1,3,2]dioxaphosphepine 4-oxide	Suifu<br>361342-51-0
(*R*)-**B9**	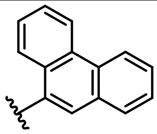	(*R*)-3,3'-Bis(9-phenanthrenyl)-1,1'-binaphthyl-2,2'-diyl hydrogen phosphate	Suifu<br>864943-22-6
(*R*)-**B10**	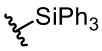	(11b*R*)-4-Hydroxy-2,6-bis(triphenylsilyl)dinaphtho[2,1-d:1',2'-f][1,3,2]dioxaphosphepine 4-oxide	Suifu<br>791616-55-2

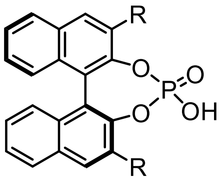

No.	R group	Name	Supplier<br>CAS#
(*S*)-**B3**	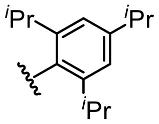	(11b*S*)-4-Hydroxy-2,6-bis(2,4,6-triisopropylphenyl)dinaphtho[2,1-d:1',2'-f][1,3,2]dioxaphosphepine 4-oxide	Suifu<br>874948-63-7

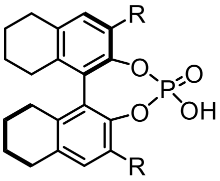

No.	R group	Name	Supplier<br>CAS#
(*R*)-**C1**	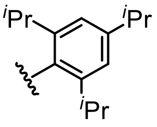	(11b*R*)-4-Hydroxy-2,6-bis(2,4,6-triisopropylphenyl)-8,9,10,11,12,13,14,15-octahydrodinaphtho[2,1-d:1',2'-f][1,3,2]dioxaphosphepine 4-oxide	Suifu<br>929294-27-9

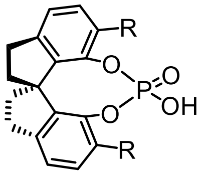

No.	R group	Name	Supplier<br>CAS#
(*R*)-**D1**	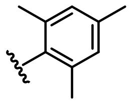	(11a*R*)-12-Hydroxy-1,10-dimesityl-4,5,6,7-tetrahydrodiindeno[7,1-de:1',7'-fg][1,3,2]dioxaphosphocine 12-oxide	Suifu<br>1801196-27-9
(*R*)-**D2**	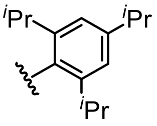	(*R*)-12-Hydroxy-1,10-bis(2,4,6-triisopropylphenyl)-4,5,6,7-tetrahydrodiindeno[7,1-de:1',7'-fg][1,3,2]dioxaphosphocine 12-oxide	Suifu<br>1372719-95-3
(*R*)-**D3**	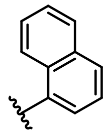	(11a*R*)-12-Hydroxy-1,10-di(naphthalen-1-yl)-4,5,6,7-tetrahydrodiindeno[7,1-de:1',7'-fg][1,3,2]dioxaphosphocine 12-oxide	Suifu<br>1297613-73-0
(*R*)-**D4**	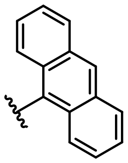	(11a*R*)-3,7-Di-9-anthracenyl-10,11,12,13-tetrahydro-5-hydroxy-5-oxide-diindeno[7,1-de:1',7'-fg][1,3,2]dioxaphosphocin	Suifu<br>1345628-08-1
(*R*)-**D5**	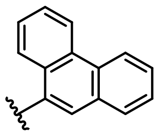	(11a*R*)-10,11,12,13-Tetrahydro-5-hydroxy-3,7-di-9-phenanthrenyl-diindeno[7,1-de:1',7'-fg][1,3,2]dioxaphosphocin-5-oxide	BLD<br>1372719-93-1

Back to contents

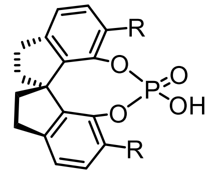

No.	R group	Name	Supplier	CAS#
(*S*)-**D3**	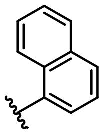	(11a*S*)-12-Hydroxy-1,10-di(naphthalen-1-yl)-4,5,6,7-tetrahydrodiindeno[7,1-de:1',7'-fg][1,3,2]dioxaphosphocine 12-oxide	BLD	1258327-08-0
(*S*)-**D5**	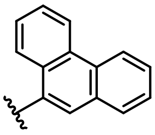	(11a*S*)-10,11,12,13-Tetrahydro-5-hydroxy-3,7-di-9-phenanthrenyl-5-oxide-diindeno[7,1-de:1',7'-fg][1,3,2]dioxaphosphocin	BLD	1585988-92-6

Table S1.6 Catalog of commercial reagents.

Alcohols			
Structure	Name	CAS#	Supplier
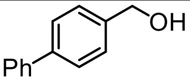	[1,1'-Biphenyl]-4-ylmethanol	3597-91-9	BLD
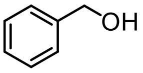	Benzyl alcohol	100-51-6	TCI
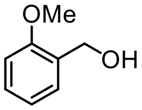	2-Methoxybenzyl alcohol	612-16-8	TCI
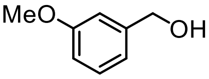	3-Methoxybenzyl alcohol	6971-51-3	TCI
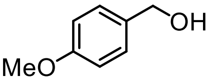	4-Methoxybenzyl alcohol	105-13-5	Acros
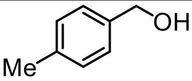	4-Methylbenzyl alcohol	589-18-4	BLD
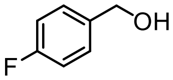	4-Fluorobenzyl alcohol	459-56-3	Nova-Malts
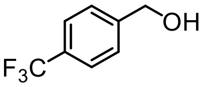	4-(Trifluoromethyl)benzyl alcohol	349-95-1	Nova-Malts
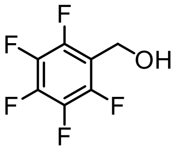	2,3,4,5,6-Pentafluorobenzyl alcohol	440-60-8	BLD
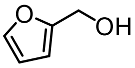	(2-Furyl)methyl alcohol	98-00-0	Acros
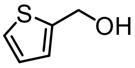	2-Thiophenemethanol	636-72-6	Sigma-Aldrich

Back to contents

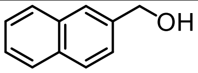	2-Naphthalenemethanol	1592-38-7	BLD
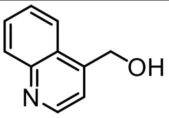	Quinolin-4-ylmethanol	6281-32-9	BLD
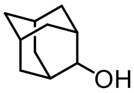	2-Adamantanol	700-57-2	Nova-Malts
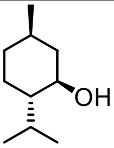	(-)-Menthol	2216-51-5	Acros
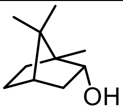	(-)-Borneol	464-45-9	Nova-Malts
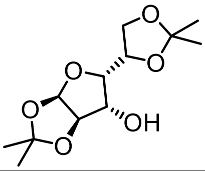	Diacetone-D-glucose	582-52-5	Nova-Malts
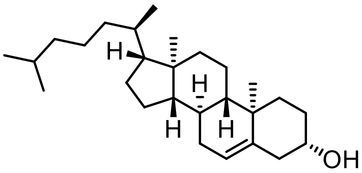	Cholesterol	57-88-5	Sigma-Aldrich
Aldehydes			
Structure	Name	CAS#	Supplier
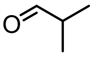	2-Methylpropanal	78-84-2	Alfa Aesar
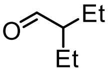	2-Ethylbutyraldehyde	97-96-1	TCI
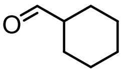	Cyclohexanecarbaldehyde	2043-61-0	Nova-Malts
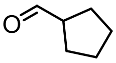	Cyclopentanecarbaldehyde	872-53-7	BLD
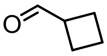	Cyclobutanecarbaldehyde	2987-17-9	BLD
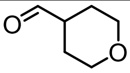	Tetrahydropyran-4-carbaldehyde	50675-18-8	BLD
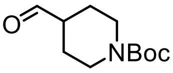	*tert*-Butyl 4-formylpiperidine-1-carboxylate	137076-22-3	BLD
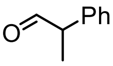	2-Phenylpropanal	93-53-8	Nova-Malts
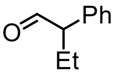	2-Phenylbutanal	2439-43-2	BLD
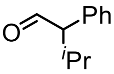	3-Methyl-2-phenylbutanal	2439-44-3	BLD

Back to contents

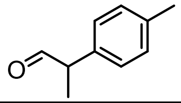	2-(4-Methylphenyl)propanal	99-72-9	BLD
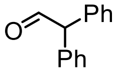	Diphenylacetaldehyde	947-91-1	BLD
Amines			
Structure	Name	CAS#	Supplier
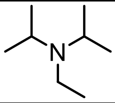	*N*,*N*-Diisopropylethylamine (DIPEA)	7087-68-5	Sigma-Aldrich
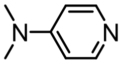	*N*,*N*-Dimethylpyridin-4-amine (DMAP)	1122-58-3	Merck
Hydride reagents			
Structure	Name	CAS#	Supplier
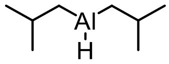	Diisobutylaluminum hydride (DIBAL-H)	1191-15-7	Alfa Aesar
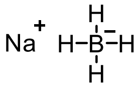	Sodium borohydride	16940-66-2	Sigma-Aldrich
Inorganic acids			
Chemical formula	Name	CAS#	Supplier
HCl	Hydrochloric acid	7647-01-0	Merck
H_2SO_4	Sulfuric acid	7664-93-9	Fluka
Inorganic salts			
Chemical formula	Name	CAS#	Supplier
$CuBr_2$	Cupric bromide	7789-45-9	BLD
LiCl	Lithium chloride	7447-41-8	Merck
LiOH	Lithium hydroxide	1310-65-2	Acros
K_2CO_3	Potassium carbonate	584-08-7	DUKSAN
KOH	Potassium hydroxide	1310-58-3	Acros
Na_2CO_3	Sodium carbonate	497-19-8	Merck
NaOH	Sodium hydroxide	1310-73-2	UR
Iodine			
Chemical formula	Name	CAS#	Supplier
I_2	Iodine	7553-56-2	TCI
Palladium			
Chemical formula	Name	CAS#	Supplier
Pd/C	Palladium on carbon	7440-05-3	Merck
Peroxides			
Structure	Name	CAS#	Supplier
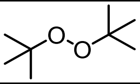	Di-*tert*-butyl peroxide (DTBP)	110-05-4	Acros
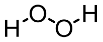	Hydrogen peroxide	7722-84-1	Shimakyu

Phosphonates			
Structure	Name	CAS#	Supplier
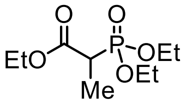	Ethyl 2-(diethoxyphosphoryl)propanoate	3699-66-9	Alfa Aesar
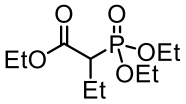	Ethyl 2-(diethoxyphosphoryl)butanoate	17145-91-4	BLD
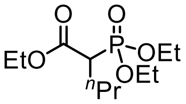	Ethyl 2-(diethoxyphosphoryl)pentanoate	35051-49-1	Alfa Aesar
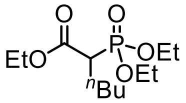	Ethyl 2-(diethoxyphosphoryl)hexanoate	4134-14-9	BLD
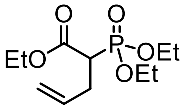	Ethyl 2-(diethoxyphosphoryl)pent-4-enoate	108298-18-6	Chemhere
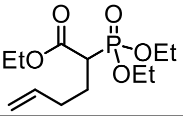	Ethyl 2-(diethoxyphosphoryl)hex-5-enoate	124032-26-4	Chemhere
Phosphonium ylide			
Structure	Name	CAS#	Supplier
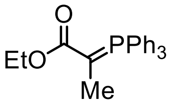	Ethyl 2-(triphenylphosphoranylidene) propanoate	5717-37-3	BLD
Radical trapping agent			
Structure	Name	CAS#	Supplier
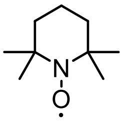	(2,2,6,6-Tetramethylpiperidin-1-yl)oxyl (TEMPO)	2564-83-2	Alfa Aesar
Other reagents			
Structure	Name	CAS#	Supplier
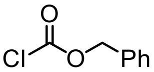	Benzyl chloroformate	501-53-1	Acros
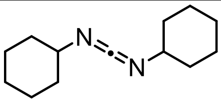	*N,N'*-Dicyclohexylcarbodiimide (DCC)	538-75-0	Merck
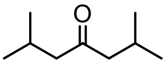	2,6-Dimethyl-4-heptanone	108-83-8	Merck
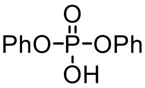	Diphenyl phosphate (DPP)	838-85-7	BLD
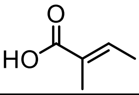	*trans*-2-methyl-2-butenoic acid	80-59-1	Nova-Malts
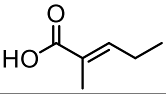	*trans*-2-Methyl-2-pentenoic acid	16957-70-3	Nova-Malts

Back to contents

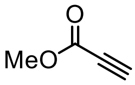	Methyl propiolate	922-67-8	Sigma-Aldrich
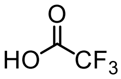	Trifluoroacetic acid (TFA)	76-05-1	Merck
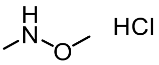	*N*,*O*-Dimethylhydroxylamine hydrochloride	6638-79-5	Sigma-Aldrich
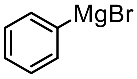	Phenylmagnesium bromide (PhMgBr)	100-58-3	Sigma-Aldrich

1.5 Summary Table of Synthesized Compounds

Table S1.7 Catalog of ethyl α,β-unsaturated esters

No.	Structure	Name	Reference
**S1a**	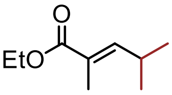	Ethyl (*E*)-2,4-dimethylpent-2-enoate	ref. 1 link
**S1b**	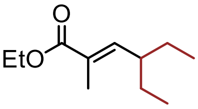	Ethyl (*E*)-4-ethyl-2-methylhex-2-enoate	ref. 2 link
**S1d**	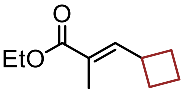	Ethyl (*E*)-3-cyclobutyl-2-methylacrylate	unpublished compound
**S1e**	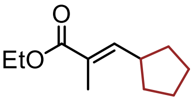	Ethyl (*E*)-3-cyclopentyl-2-methylacrylate	ref. 2 link
**S1f**	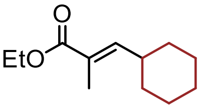	Ethyl (*E*)-3-cyclohexyl-2-methylacrylate	ref. 3 link
**S1g**	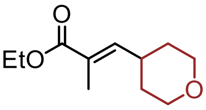	Ethyl (*E*)-2-methyl-3-(tetrahydro-2*H*-pyran-4-yl)acrylate	unpublished compound
**S1h**	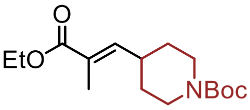	*tert*-Butyl (*E*)-4-(3-ethoxy-2-methyl-3-oxoprop-1-en-1-yl)piperidine-1-carboxylate	unpublished compound
**S1k**	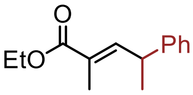	Ethyl (*E*)-2-methyl-4-phenylpent-2-enoate	ref. 4 link
**S1l**	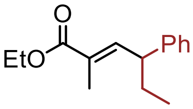	Ethyl (*E*)-2-methyl-4-phenylhex-2-enoate	unpublished compound
**S1m**	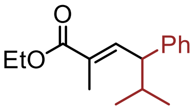	Ethyl (*E*)-2,5-dimethyl-4-phenylhex-2-enoate	unpublished compound

Back to contents

**S1n**	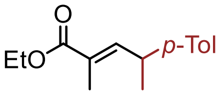	Ethyl (*E*)-2-methyl-4-(*p*-tolyl)pent-2-enoate	unpublished compound
See procedure			
**S2a**	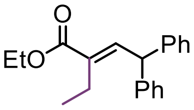	Ethyl (*E*)-2-ethyl-4,4-diphenylbut-2-enoate	unpublished compound
**S2b**	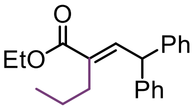	Ethyl (*E*)-2-(2,2-diphenylethylidene)pentanoate	unpublished compound
**S2c**	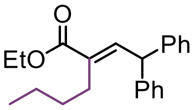	Ethyl (*E*)-2-(2,2-diphenylethylidene)hexanoate	unpublished compound
**S2d**	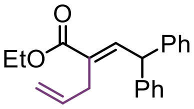	Ethyl (*E*)-2-(2,2-diphenylethylidene)pent-4-enoate	unpublished compound
**S2e**	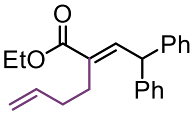	Ethyl (*E*)-2-(2,2-diphenylethylidene)hex-5-enoate	unpublished compound
See procedure			

Table S1.8 Catalog of *α*,*β*-unsaturated carboxylic acids

No.	Structure	Name	Reference
**S3a**	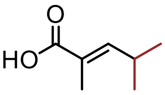	(*E*)-2,4-Dimethylpent-2-enoic acid	ref. 5 link
**S3b**	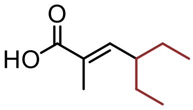	(*E*)-4-Ethyl-2-methylhex-2-enoic acid	unpublished compound
**S3d**	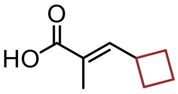	(*E*)-3-Cyclobutyl-2-methylacrylic acid	unpublished compound
**S3e**	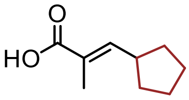	(*E*)-3-Cyclopentyl-2-methylacrylic acid	unpublished compound
**S3f**	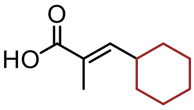	(*E*)-3-Cyclohexyl-2-methylacrylic acid	ref. 6 link
**S3g**	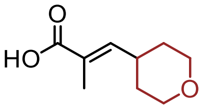	(*E*)-2-Methyl-3-(tetrahydro-2*H*-pyran-4-yl)acrylic acid	unpublished compound
**S3h**	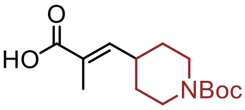	(*E*)-3-(1-(*tert*-Butoxycarbonyl)piperidin-4-yl)-2-methylacrylic acid	unpublished compound

Back to contents

**S3k**	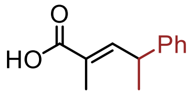	(*E*)-2-Methyl-4-phenylpent-2-enoic acid	ref. 7<br>link
**S3l**	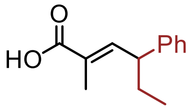	(*E*)-2-Methyl-4-phenylhex-2-enoic acid	unpublished compound
**S3m**	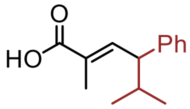	(*E*)-2,5-Dimethyl-4-phenylhex-2-enoic acid	unpublished compound
**S3n**	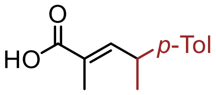	(*E*)-2-Methyl-4-(*p*-tolyl)pent-2-enoic acid	unpublished compound
**S3o**	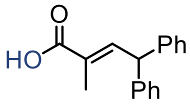	(*E*)-2-Methyl-4,4-diphenylbut-2-enoic acid	unpublished compound
**S3p**	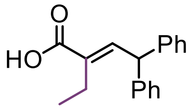	(*E*)-2-Ethyl-4,4-diphenylbut-2-enoic acid	unpublished compound
**S3q**	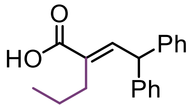	(*E*)-2-(2,2-Diphenylethylidene)pentanoic acid	unpublished compound
**S3r**	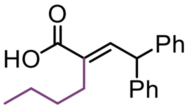	(*E*)-2-(2,2-Diphenylethylidene)hexanoic acid	unpublished compound
**S3s**	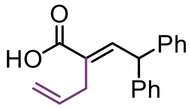	(*E*)-2-(2,2-Diphenylethylidene)pent-4-enoic acid	unpublished compound
**S3t**	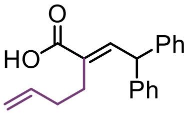	(*E*)-2-(2,2-diphenylethylidene)hex-5-enoic acid	unpublished compound
See procedure			

Back to contents

2. General Procedures

2.1 Synthesis of ethyl *α,β*-unsaturated esters

General Procedure A: Ethyl *α,β*-unsaturated esters from Wittig reaction

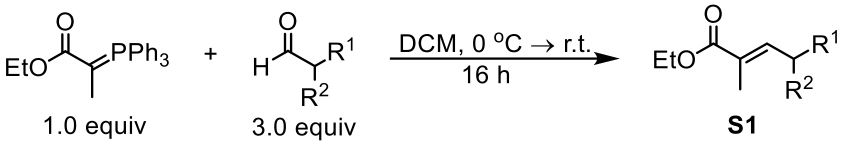

An oven-dried flask containing a stirring bar was cooled to room temperature under a high vacuum and refilled with nitrogen, where ethyl 2-(diethoxyphosphoryl) propanoate (1.0 equiv) was added. Dry DCM (0.1 M) was added into the flask, which was then placed in a 0 °C bath (ice/water) for 15 minutes. Aldehyde (3.0 equiv) was then added dropwise to a vigorously stirring flask. The mixture was taken out of the ice bath and stirred at room temperature for 16 h. Then, the solvent was removed under reduced pressure. The crude product was purified by column chromatography (height of packing silica gel: 6.0 – 8.0 cm; dry loading, gradient elution) to afford the pure ester. **S1a** (380 mg, 2.8 mmol scale, 91%, known compound), **S1b** (440 mg, 2.8 mmol scale, 87%, known compound), **S1d** (390 mg, 2.8 mmol scale, 82%, unpublished compound), **S1e** (420 mg, 2.8 mmol scale, 84%, known compound), **S1f** (450 mg, 2.8 mmol scale, 83%, known compound), **S1g** (440 mg, 2.8 mmol scale, 80%, unpublished compound), **S1h** (660 mg, 2.8 mmol scale, 80%, unpublished compound), **S1k** (470 mg, 2.8 mmol scale, 75%, known compound), **S1l** (523 mg, 2.8 mmol scale, 82%, unpublished compound), **S1m** (531 mg, 2.8 mmol scale, 78%, unpublished compound) and **S1n** (527 mg, 2.8 mmol scale, 82%, unpublished compound).

*All characterization data for **S1a**[1], **S1b**[2], **S1e**[2], **S1f**[3], and **S1k**[4] are consistent with their respective reported literature.*

See the summary table of ethyl α,β-unsaturated esters

Back to contents

Ethyl (*E*)-3-cyclobutyl-2-methylacrylate (S1d)

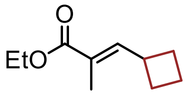

S1d

According to General Procedure A, ethyl 2-(diethoxyphosphoryl) propanoate (1.0 g, 2.8 mmol, 1.0 equiv) and cyclobutanecarbaldehyde (696 mg, 8.3 mmol, 3.0 equiv) were used to obtain the crude residue, which was purified by silica gel column chromatography (*n*-hexane/ethyl acetate 100:1, v/v), to afford **S1d** as a colorless oil (380 mg, 2.3 mmol, 82%).

^{1}H NMR (400 MHz, $CDCl_3$) δ 6.82 (d, J = 8.6 Hz, 1H), 4.16 (q, J = 7.1 Hz, 2H), 3.26 – 3.16 (m, 1H), 2.20 – 2.16 (m, 2H), 1.93 – 1.85 (m, 4H), 1.75 (s, 3H), 1.27 (t, J = 7.1 Hz, 3H) ppm.

^{13}C{^{1}H} NMR (101 MHz, $CDCl_3$) δ 168.4, 146.6, 126.0, 60.3, 34.6, 28.8, 19.0, 14.2, 12.4 ppm.

See NMR Spectra

HRMS (*m/z*): (ESI) calc'd for $C_{10}H_{16}O_2{}^{23}Na$ $[M+Na]^+$: 191.1043, found: 191.1042.

IR (ATR) ν_{max}: 2976, 2936, 2869, 1707, 1645, 1445, 1366, 1255, 1105, 1032 and 744 cm^{-1}.

TLC: R_f = 0.56 (*n*-hexane/ethyl acetate 10:1, v/v)

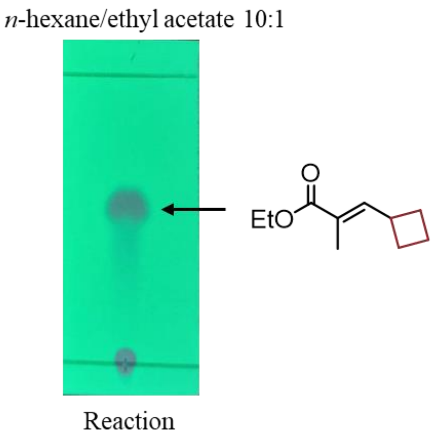

Back to contents

Ethyl (*E*)-2-methyl-3-(tetrahydro-2*H*-pyran-4-yl)acrylate (S1g)

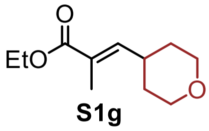

S1g

According to General Procedure A, ethyl 2-(diethoxyphosphoryl) propanoate (1.0 g, 2.8 mmol, 1.0 equiv) and tetrahydropyran-4-carbaldehyde (945 mg, 8.3 mmol, 3.0 equiv) were used to obtain the crude residue, which was purified by silica gel column chromatography (*n*-hexane/ethyl acetate 100:1, v/v), to afford **S1g** as a colorless oil (440 mg, 2.2 mmol, 80%).

^{1}H NMR (400 MHz, $CDCl_3$) δ 6.45 (d, J = 9.5 Hz, 1H), 4.13 – 4.05 (m, 2H), 3.89 – 3.84 (m, 2H), 3.39 – 3.33 (m, 2H), 2.53 – 2.43 (m, 1H), 1.75 (s, 3H), 1.72 – 1.68 (m, 2H), 1.48 – 1.43 (m, 2H), 1.18 (t, J = 7.1 Hz, 3H) ppm.

^{13}C{^{1}H} NMR (101 MHz, $CDCl_3$) δ 167.9, 144.5, 126.9, 67.0, 60.3, 34.7, 31.3, 14.0, 12.2 ppm.

Note: ethyl tetrahydro-2*H*-pyran-4-carboxylate is present as an impurity in the collected NMR spectra.

See NMR Spectra

HRMS (*m/z*): (ESI) calc'd for $C_{11}H_{18}O_3{}^{23}Na$ [M+Na]$^+$: 221.1148, found: 221.1148.

IR (ATR) ν_{max}: 2933, 1844, 1706, 1650, 1229, 1099, 982, 869 and 749 cm^{-1}.

TLC: R_f = 0.43 (*n*-hexane/ethyl acetate 10:1, v/v)

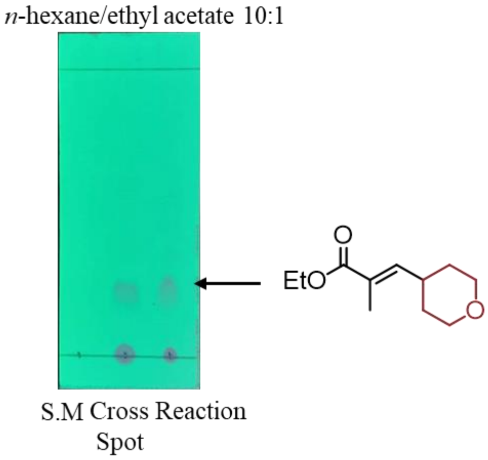

Back to contents

tert-Butyl (_E_)-4-(3-ethoxy-2-methyl-3-oxoprop-1-en-1-yl)piperidine-1-carboxylate (S1h)

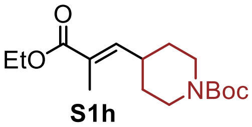

S1h

According to General Procedure A, ethyl 2-(diethoxyphosphoryl) propanoate (1.0 g, 2.8 mmol, 1.0 equiv) and *tert*-butyl 4-formylpiperidine-1-carboxylate (1765 mg, 8.3 mmol, 3.0 equiv) were used to obtain the crude residue, which was purified by silica gel column chromatography (*n*-hexane/ethyl acetate 100:1, v/v), to afford **S1h** as a colorless oil (660 mg, 2.2 mmol, 80%).

^{1}H NMR (300 MHz, $CDCl_3$) δ 6.44 – 6.41 (m, 1H), 4.07 – 3.97 (m, 4H), 2.66 – 2.63 (m, 2H), 2.38 – 2.35 (m, 1H), 1.75 (s, 3H), 1.49 (d, J = 11.2 Hz, 4H), 1.34 – 1.20 (m, 9H), 1.17 – 1.14 (m, 3H) ppm.

^{13}C{^{1}H} NMR (101 MHz, $CDCl_3$) δ 168.1, 154.7, 144.5, 127.2, 79.3, 60.4, 43.3, 35.8, 30.7, 28.3, 14.7, 12.4 ppm.

See NMR Spectra

HRMS (*m/z*): (ESI) calc'd for $C_{16}H_{27}O_4N^{23}Na$ $[M+Na]^+$: 320.1832, found: 320.1832.

IR (ATR) ν_{max}: 2976, 2931, 2851, 1692, 1365, 1146, 1099, 868 and 749 cm^{-1}.

TLC: R_f = 0.33 (*n*-hexane/ethyl acetate 4:1, v/v)

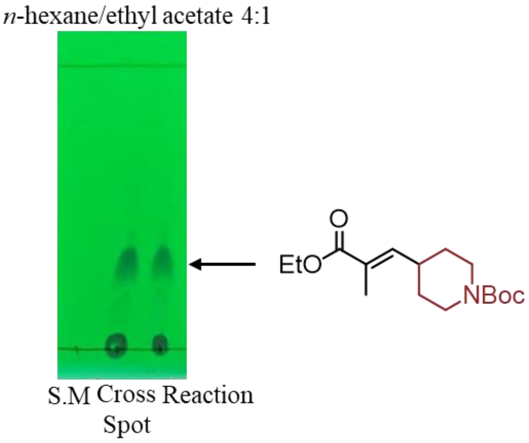

Back to contents

Ethyl (*E*)-2-methyl-4-phenylhex-2-enoate (S1l)

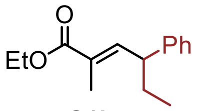

S1l

According to General Procedure A, ethyl 2-(diethoxyphosphoryl) propanoate (1.0 g, 2.8 mmol, 1.0 equiv) and 2-phenylbutanal (1227 mg, 8.3 mmol, 3.0 equiv) were used to obtain the crude residue, which was purified by silica gel column chromatography (*n*-hexane/ethyl acetate 100:1, v/v), to afford **S1l** as a colorless oil (523 mg, 2.3 mmol, 82%).

^{1}H NMR (400 MHz, $CDCl_3$) δ 7.32 – 7.29 (m, 2H), 7.22 – 7.19 (m, 3H), 6.89 – 6.86 (m, 1H), 4.18 (q, J = 7.1 Hz, 2H), 3.51 – 3.47 (m, 1H), 1.89 (d, J = 1.2 Hz, 3H), 1.84 – 1.73 (m, 2H), 1.28 (t, J = 7.1 Hz, 3H), 0.88 (t, J = 7.4 Hz, 3H) ppm.

^{13}C{^{1}H} NMR (101 MHz, $CDCl_3$) δ 168.3, 144.9, 143.4, 128.6, 127.9, 127.5, 126.4, 60.5, 46.7, 29.3, 14.3, 12.7, 12.0 ppm.

See NMR Spectra

HRMS (*m/z*): (ESI) calc'd for $C_{15}H_{20}O_2{}^{23}Na$ [M+Na]$^+$: 255.1356, found: 255.1356.

IR (ATR) ν_{max}: 2918, 2849, 1712, 1463, 1377, 1261, 1021 and 801 cm^{-1}.

TLC: R_f = 0.44 (*n*-hexane/ethyl acetate 10:1, v/v)

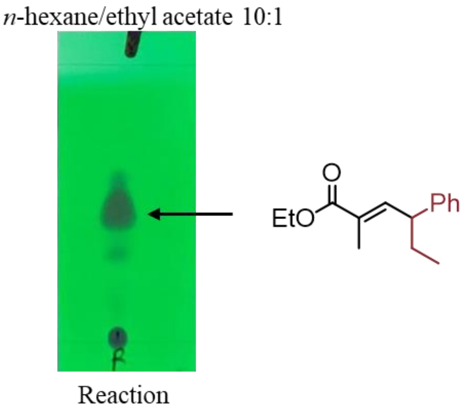

Ethyl (*E*)-2,5-dimethyl-4-phenylhex-2-enoate (S1m)

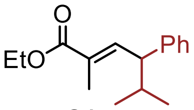

S1m

According to General Procedure A, ethyl 2-(diethoxyphosphoryl) propanoate (1.0 g, 2.8 mmol, 1.0 equiv) and 3-methyl-2-phenylbutanal (1343 mg, 8.3 mmol, 3.0 equiv) were used to obtain the crude residue, which was purified by silica gel column chromatography (*n*-hexane/ethyl acetate 100:1, v/v), to afford **S1m** as a colorless oil (531 mg, 2.2 mmol, 78%).

^{1}H NMR (300 MHz, $CDCl_3$) δ 7.36 – 7.24 (m, 5H), 7.04 (d, J = 10.3 Hz, 1H), 4.24 (dd, J = 18.1, 8.8 Hz, 2H), 3.29 (t, J = 9.7 Hz, 1H), 2.12 – 2.03 (m, 1H), 1.93 (s, 3H), 1.35 (t, J = 6.7 Hz, 3H), 1.02 (d, J = 6.3 Hz, 3H), 0.85 (d, J = 6.3 Hz, 3H) ppm.

^{13}C{^{1}H} NMR (101 MHz, $CDCl_3$) δ 168.2, 144.3, 142.8, 128.5, 128.0, 127.6, 126.3, 60.5, 52.8, 33.7, 20.8, 20.6, 14.3, 12.7 ppm.

See NMR Spectra

HRMS (*m/z*): (ESI) calc'd for $C_{16}H_{22}O_2{}^{23}Na$ $[M+Na]^+$: 269.1512, found: 269.1510.

IR (ATR) ν_{max}: 2957, 2929, 2870, 1708, 1464, 1453, 1231, 745 and 700 cm^{-1}.

TLC: R_f = 0.31 (*n*-hexane/ethyl acetate 10:1, v/v)

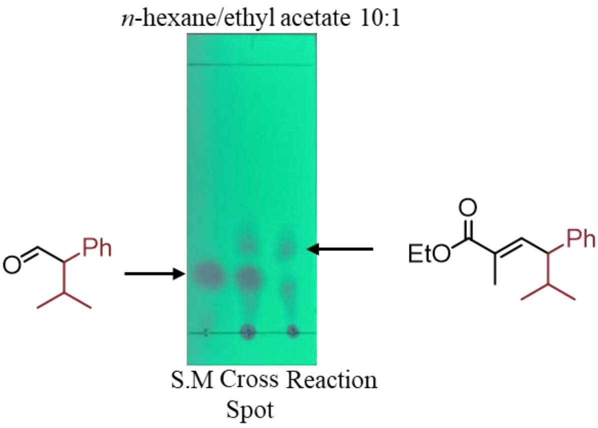

Back to contents

Ethyl (*E*)-2-methyl-4-(*p*-tolyl)pent-2-enoate (S1n)

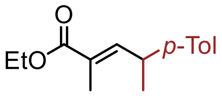

S1n

According to General Procedure A, ethyl 2-(diethoxyphosphoryl) propanoate (1.0 g, 2.8 mmol, 1.0 equiv) and 2-(4-methylphenyl)propanal (1227 mg, 8.3 mmol, 3.0 equiv) were used to obtain the crude residue, which was purified by silica gel column chromatography (*n*-hexane/ethyl acetate 100:1, v/v), to afford **S1n** as a colorless oil (527 mg, 2.3 mmol, 82%).

^{1}H NMR (400 MHz, $CDCl_3$) δ 7.14 (s, 4H), 6.85 (dd, *J* = 9.9, 1.1 Hz, 1H), 4.18 (q, *J* = 7.1 Hz, 2H), 3.81 – 3.73 (m, 1H), 2.33 (s, 3H), 1.91 (s, 3H), 1.39 (d, *J* = 7.0 Hz, 3H), 1.29 (t, *J* = 7.1 Hz, 3H) ppm.

^{13}C{^{1}H} NMR (101 MHz, $CDCl_3$) δ 168.3, 146.1, 141.5, 136.0, 129.3, 127.3, 126.8, 126.4, 60.5, 53.4, 38.2, 21.3, 20.9, 14.2, 12.5 ppm.

See NMR Spectra

HRMS (*m/z*): (ESI) calc'd for $C_{15}H_{20}O_2{}^{23}Na$ $[M+Na]^+$: 255.1356, found: 255.1354.

IR (ATR) ν_{max}: 2979, 2934, 1732, 1451, 1161, 1024, 802 and 700 cm^{-1}.

TLC: R_f = 0.43 (*n*-hexane/ethyl acetate 10:1, v/v)

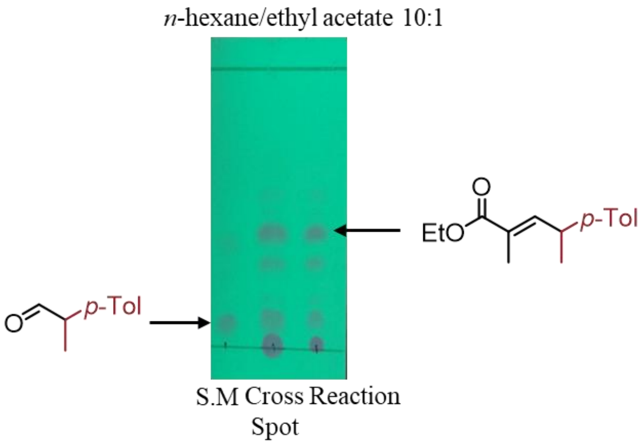

General Procedure B: Ethyl *α,β*-unsaturated esters from Horner-Wadsworth-Emmons reaction

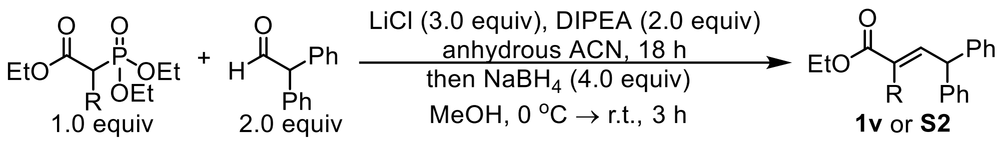

An oven-dried flask containing a stirring bar was introduced into the nitrogen-filled glovebox, where LiCl (3.0 equiv), phosphate (1.0 equiv) and dry ACN (0.2 M) were added. The flask was taken out from the glovebox and DIPEA (2.0 equiv) was then added to the solution. After being stirred for 30 minutes, diphenylacetaldehyde (2.0 equiv) was added dropwise into the mixture. After the addition was complete, the resulting mixture was stirred at room temperature for 18 h. The reaction was filtered, concentrated and extracted with DCM (30 mL x 3). The combined organic phase was washed with brine, dried over $MgSO_4$, filtered and concentrated under reduced pressure. $NaBH_4$ was then added to this residue in a 0 °C bath (ice/water) under nitrogen atmosphere, stirring for 3 h. After the benzophenone totally transferred to diphenylmethanol, the mixture was concentrated and extracted with DCM (30 mL x 3). The combined organic phase was washed with brine, dried over $MgSO_4$, filtered and concentrated under reduced pressure. The crude residue was purified by silica gel column chromatography (height of packing silica gel: 6.0 – 8.0 cm; dry loading, gradient elution) to obtain desired product.

See the summary table of ethyl α,β-unsaturated ester

Ethyl (*E*)-2-methyl-4,4-diphenylbut-2-enoate (1v)

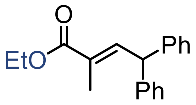

1v

According to General Procedure B, ethyl 2-(diethoxyphosphoryl)propanoate (2.0 g, 10.2 mmol, 1.0 equiv) was used to obtain crude residue, which was purified by silica gel column chromatography (*n*-hexane/ethyl acetate 100:1, v/v), to afford **1v** as a colorless oil (1.0 g, 3.8 mmol, 37%).

^{1}H NMR (300 MHz, $CDCl_3$) δ 7.32 – 7.27 (m, 4H), 7.22 – 7.19 (m, 6H), 6.36 (dd, J = 10.4, 0.9 Hz, 1H), 5.84 (d, J = 10.4 Hz, 1H), 4.21 (q, J = 7.1 Hz, 2H), 2.00 (s, 3H), 1.28 (t, J = 7.1 Hz, 3H) ppm.

^{13}C{^{1}H} NMR (101 MHz, $CDCl_3$) δ 167.7, 143.7, 142.9, 128.5, 128.3, 127.4, 126.4, 60.4, 49.2, 21.0, 14.2 ppm.

See NMR Spectra

HRMS (*m/z*): (EI) calc'd for $C_{19}H_{20}O_2$ $[M]^+$: 280.1458, found 280.1455.

IR (ATR) ν_{max}: 3025, 1713, 1492, 1450, 1211, 738 and 698 cm^{-1}.

TLC: R_f = 0.69 (*n*-hexane/ethyl acetate 4:1, v/v)

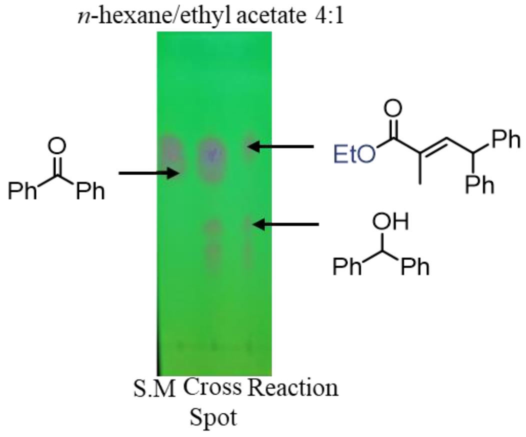

Back to contents

Ethyl (*E*)-2-ethyl-4,4-diphenylbut-2-enoate (S2a)

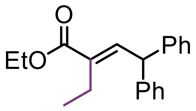

S2a

According to General Procedure B, ethyl 2-(diethoxyphosphoryl)butanoate (1.4 g, 7.1 mmol, 1.0 equiv) was used to obtain crude residue, which was purified by silica gel column chromatography (*n*-hexane/ethyl acetate 100:1, v/v), to afford **S2a** as a yellow oil (130 mg, 0.4 mmol, 6%).

^{1}H NMR (400 MHz, $CDCl_3$) δ 7.28 – 7.20 (m, 10H), 6.26 (d, J = 9.7 Hz, 1H), 5.69 (d, J = 9.8 Hz, 1H), 4.21 – 4.19 (m, 2H), 2.37 – 2.35 (m, 2H), 1.28 – 1.25 (m, 3H), 1.07 (t, J = 6.4 Hz, 3H) ppm.

^{13}C{^{1}H} NMR (101 MHz, $CDCl_3$) δ 167.9, 143.8, 140.3, 133.9, 128.5, 128.3, 126.3, 60.3, 49.2, 27.7, 14.2, 13.5 ppm.

See NMR Spectra

HRMS (*m/z*): (EI) calc'd for $C_{20}H_{22}O_2$ $[M]^+$: 294.1614, found: 294.1614.

IR (ATR) ν_{max}: 3061, 1708, 1492, 1213, 1029, 698 and 511 cm^{-1}.

TLC: R_f = 0.62 (*n*-hexane/ethyl acetate 4:1, v/v)

Hexane/ethyl acetate 4/1

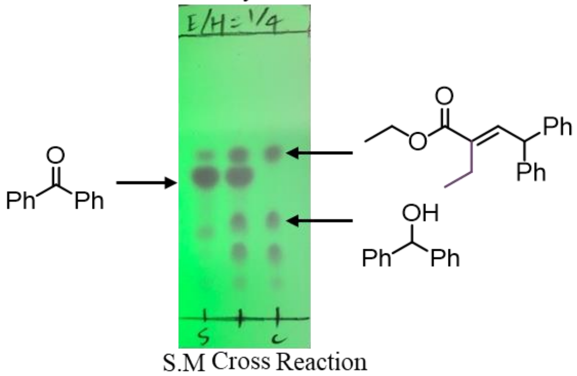

S.M Cross Reaction
Spot

Ethyl (*E*)-2-(2,2-diphenylethylidene)pentanoate (S2b)

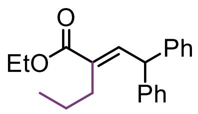

S2b

According to General Procedure B, ethyl 2-(diethoxyphosphoryl)hexanoate (2.7 g, 10.2 mmol,1.0 equiv) was used to obtain crude residue, which was purified by silica gel column chromatography (*n*-hexane/ethyl acetate 100:1, v/v), to afford **S2b** as a yellow oil (503 mg, 1.6 mmol, 16%).

^{1}H NMR (400 MHz, $CDCl_3$) δ 7.31 – 7.29 (m, 4H), 7.26 – 7.23 (m, 6H), 6.29 (d, J = 10.3 Hz, 1H), 5.72 (d, J = 10.3 Hz, 1H), 4.23 (q, J = 7.0 Hz, 2H), 2.34 (t, J = 7.2 Hz, 2H), 1.55 – 1.49 (m, 2H), 1.30 (t, J = 6.9 Hz, 3H), 0.93 (t, J = 7.3 Hz, 3H) ppm.

^{13}C{^{1}H} NMR (101 MHz, $CDCl_3$) δ 167.9, 143.8, 141.3, 132.4, 128.4, 128.3, 126.3, 60.3, 49.4, 36.8, 22.1, 14.2, 13.6 ppm.

See NMR Spectra

HRMS (*m/z*): (ESI) calc’d for $C_{21}H_{24}O_2{}^{23}Na$ [M+Na]$^+$: 331.1668, found :331.1669.

IR (ATR) ν_{max}: 3027, 1708, 1377, 1276, 1030, 697 and 598 cm^{-1}.

TLC: R_f = 0.80 (*n*-hexane/ethyl acetate 4:1, v/v)

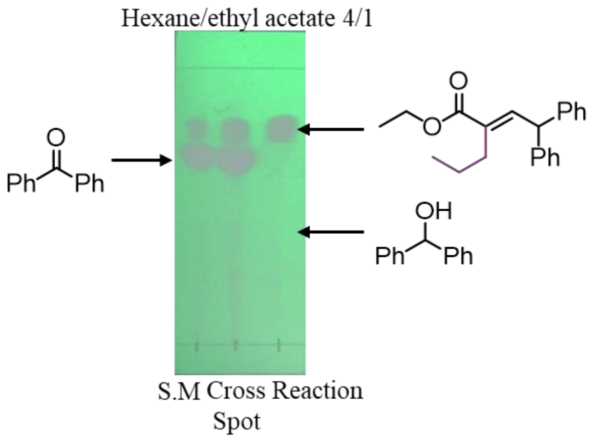

Ethyl (*E*)-2-(2,2-diphenylethylidene)hexanoate (S2c)

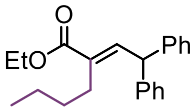

S2c

According to General Procedure B, ethyl 2-(diethoxyphosphoryl)hexanoate (2.0 g, 10.2 mmol,1.0 equiv) was used to obtain crude residue, which was purified by silica gel column chromatography (*n*-hexane/ethyl acetate 100:1, v/v), to afford **S2c** as a yellow oil (364 mg, 1.1 mmol, 11%).

^{1}H NMR (400 MHz, $CDCl_3$) δ 7.35 – 7.32 (m, 4H), 7.26 – 7.24 (m, 6H), 6.32 (d, *J* = 10.2 Hz, 1H), 5.75 (d, *J* = 10.2 Hz, 1H), 4.26 (q, *J* = 6.9 Hz, 2H), 2.41 – 2.38 (m, 2H), 1.52 – 1.51 (m, 2H), 1.39 – 1.30 (m, 5H), 0.95 (t, *J* = 7.1 Hz, 3H) ppm.

^{13}C{^{1}H} NMR (101 MHz, $CDCl_3$) δ 168.0, 143.8, 141.1, 132.7, 128.5, 128.3, 126.3, 60.3, 49.3, 34.5, 31.2, 22.3, 14.2, 13.9 ppm.

See NMR Spectra

HRMS (*m/z*): (EI) calc'd for $C_{22}H_{26}O_2$ $[M]^+$: 322.1927, found: 322.1925.

IR (ATR) ν_{max}: 2958, 2853, 1720, 1465, 1377, 722 and 502 cm^{-1}.

TLC: R_f = 0.75 (*n*-hexane/ethyl acetate 3:1, v/v)

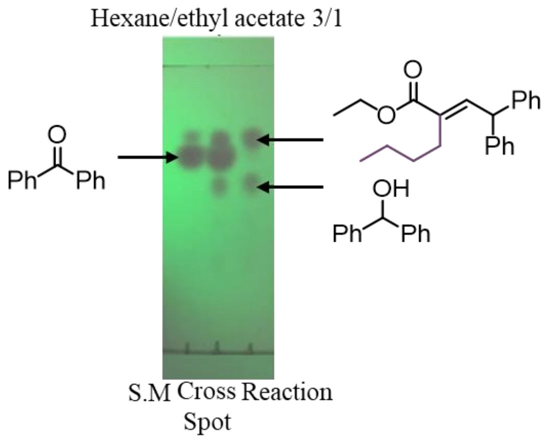

Ethyl (*E*)-2-(2,2-diphenylethylidene)pent-4-enoate (S2d)

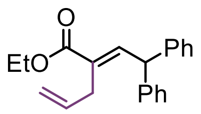

S2d

According to General Procedure B, ethyl 2-(diethoxy phosphoryl)pent-4-enoate (1.5 g, 7.5 mmol, 1.0 equiv) was used to obtain crude residue, which was purified by silica gel column chromatography (*n*-hexane/ethyl acetate 100:1, v/v), to afford **S2d** as a colorless oil (257 mg, 0.8 mmol, 11%).

^{1}H NMR (400 MHz, $CDCl_3$) δ 7.36 – 7.32 (m, 6H), 7.25 – 7.24 (m, 4H), 6.39 (d, J = 10.4 Hz, 1H), 5.95 – 5.84 (m, 2H), 5.15 – 5.09 (m, 2H), 4.25 (q, J = 7.1 Hz, 2H), 3.15 (d, J = 6.3 Hz, 2H), 1.32 (t, J = 7.1 Hz, 3H) ppm.

^{13}C{^{1}H} NMR (101 MHz, $CDCl_3$) δ 167.3, 143.6, 142.8, 135.5, 130.4, 128.5, 128.3, 126.4, 116.6, 60.4, 49.2, 38.4, 14.2 ppm.

See NMR Spectra

HRMS (*m/z*): (EI) calc'd for $C_{21}H_{22}O_2$ $[M]^+$: 306.1643, found: 306.1641.

IR (ATR) ν_{max}: 2960, 2924, 2855, 1756, 1465, 1376 and 722 cm^{-1}.

TLC: R_f = 0.70 (*n*-hexane/ethyl acetate 6:1, v/v)

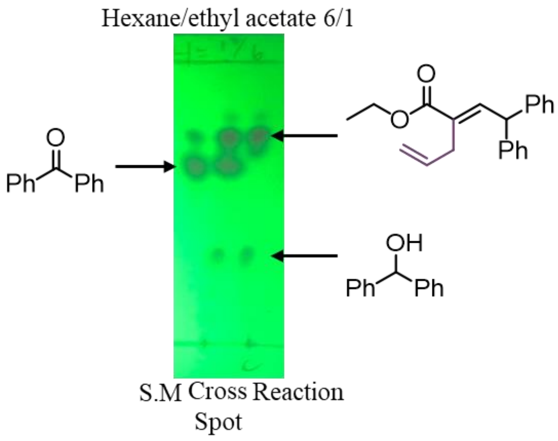

Back to contents

Ethyl (*E*)-2-(2,2-diphenylethylidene)hex-5-enoate (S2e)

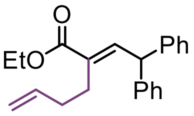

S2e

According to General Procedure B, ethyl 2-(diethoxy phosphoryl)hex-5-enoate (500 mg, 1.8 mmol, 1.0 equiv) was used to obtain crude residue, which was purified by silica gel column chromatography (*n*-hexane/ethyl acetate 20:1, v/v), to afford **S2e** as a colorless oil (150 mg, 0.8 mmol, 47%).

^{1}H NMR (300 MHz, $CDCl_3$) δ 7.31 – 7.29 (m, 4H), 7.23 – 7.18 (m, 6H), 6.29 (d, J = 10.3 Hz, 1H), 5.86 – 5.71 (m, 2H), 5.04 – 4.95 (m, 2H), 4.21 (q, J = 7.1 Hz, 2H), 2.44 (t, J = 7.4 Hz, 2H), 2.24 (q, J = 7.0 Hz, 2H), 1.28 (t, J = 6.9 Hz, 3H) ppm.

^{13}C{^{1}H} NMR (101 MHz, $CDCl_3$) δ 167.6, 143.7, 142.2, 137.7, 131.6, 130.1, 128.4, 126.4, 115.3, 60.4, 49.3, 34.2, 33.2, 14.2 ppm.

See NMR Spectra

HRMS (*m/z*): (EI) calc'd for $C_{22}H_{24}O_2$ $[M]^+$: 320.1771, found: 320.1770.

IR (ATR) ν_{max}: 3034, 2922, 1710, 1490,1212, 1089 and 698 cm^{-1}.

TLC: R_f = 0.70 (*n*-hexane/ethyl acetate 10:1, v/v)

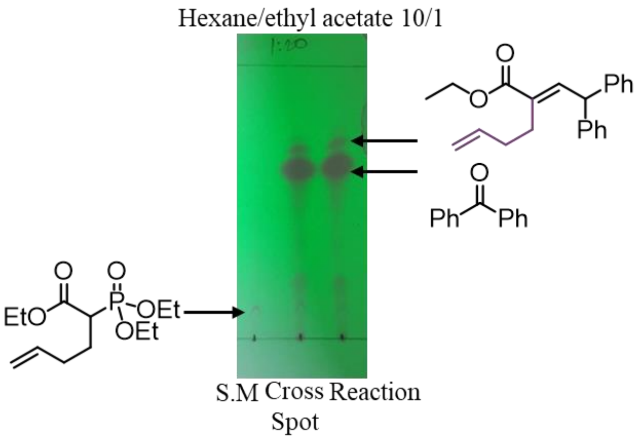

2.2 General Procedure C: Synthesis of *α,β*-unsaturated carboxylic acids

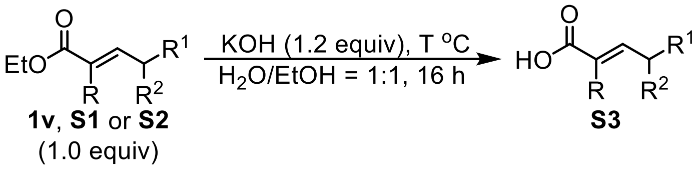

A solution of compound **1v**, **S1** or **S2** (1.0 equiv) and KOH (1.2 equiv) in H_2O/EtOH (1:1, 0.1 M) was stirred at T °C for 16 h. The ethanol was stripped off under reduced pressure, and the remaining mixture was washed with diethyl ether. Under cooling with an ice/water bath, 2.0 M $HCl_{(aq.)}$ was added to adjust the solutions to pH 3, and the resulting mixture was extracted with diethyl ether (30 mL x 3). The combined organic phase was washed with brine, dried over $MgSO_4$, filtered and concentrated under reduced pressure. The residue was purified by column chromatography (height of packing silica gel: 6.0 – 8.0 cm; wet loading, gradient elution) affording desired product. **S3a** (450 mg, 6.4 mmol scale, 55%, known compound), **S3b** (430 mg, 5.4 mmol scale, 51%, unpublished compound), **S3d** (450 mg, 5.9 mmol scale, 54%, unpublished compound), **S3e** (450 mg, 5.5 mmol scale, 53%, unpublished compound), **S3f** (380 mg, 5.1 mmol scale, 44%, known compound), **S3g** (430 mg, 5.0 mmol scale, 50%, unpublished compound), **S3h** (473 mg, 4.3 mmol scale, 52%, unpublished compound), **S3k** (600 mg, 4.6 mmol scale, 69%, known compound), **S3l** (470 mg, 4.3 mmol scale, 54%, unpublished compound), **S3m** (477 mg, 4.1 mmol scale, 54%, unpublished compound), **S3n** (467 mg, 4.3 mmol scale, 53%, unpublished compound), **S3o** (468 mg, 3.5 mmol scale, 53%, unpublished compound), **S3p** (196 mg, 1.5 mmol scale, 49%, unpublished compound), **S3q** (254 mg, 1.6 mmol scale, 56%, unpublished compound), **S3r** (33 mg, 0.3 mmol scale, 36%, unpublished compound), **S3s** (65 mg, 0.7 mmol scale, 35%, unpublished compound), **S3t** (123 mg, 0.5 mmol scale, 90%, unpublished compound).

*All characterization data for **S3a**[5], **S3f**[6] and **S3k**[7] are consistent with their respective reported literature.*

See the summary table of α,β-unsaturated carboxylic acids

(*E*)-4-Ethyl-2-methylhex-2-enoic acid (S3b)

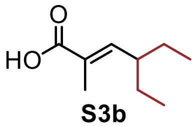

S3b

According to General Procedure C, **S1b** (1.00 g, 5.4 mmol, 1.0 equiv) and KOH (365 mg, 6.5 mmol, 1.2 equiv) were used to obtain the crude residue, which was purified by silica gel column chromatography (*n*-hexane/ethyl acetate 10:1, v/v), to afford **S3b** as a white solid (430 mg, 2.8 mmol, 51%).

^{1}H NMR (400 MHz, $CDCl_3$) δ 6.66 – 6.64 (m, 1H), 2.29 – 2.21 (m, 1H), 1.85 – 1.85 (m, 3H), 1.57 – 1.47 (m, 2H), 1.36 – 1.25 (m, 2H), 0.84 (t, J = 7.4 Hz, 6H) ppm.

^{13}C{^{1}H} NMR (101 MHz, $CDCl_3$) δ 173.6, 149.9, 127.1, 42.5, 27.6, 12.5, 11.8 ppm.

See NMR Spectra

HRMS (*m/z*): (ESI) calc'd for $C_9H_{16}O_2{}^{23}Na$ $[M+Na]^+$: 179.1043, found:179.1042.

IR (ATR) ν_{max}: 2960, 2926, 2875, 1683, 1643, 1419, 1287, 1246, 936 and 666 cm^{-1}.

Melting point: 53 – 56 °C.

TLC: R_f = 0.53 (*n*-hexane/ethyl acetate 5:1, v/v)

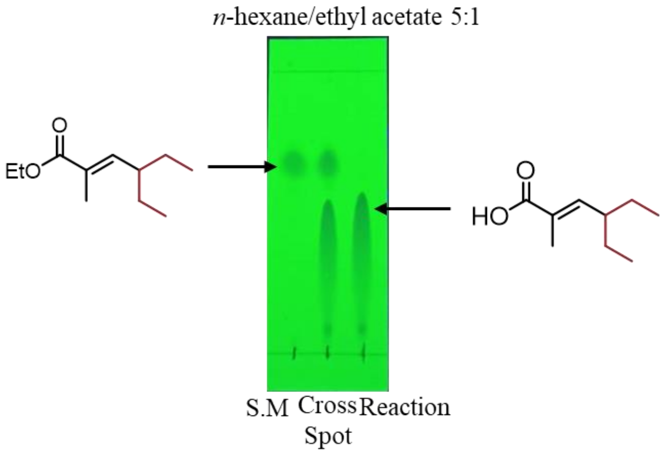

Back to contents

(*E*)-3-Cyclobutyl-2-methylacrylic acid (S3d)

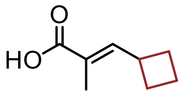

S3d

According to General Procedure C, **S1d** (1.00 g, 5.9 mmol, 1.0 equiv) and KOH (400 mg, 7.1 mmol, 1.2 equiv) were used to obtain the crude residue, which was purified by silica gel column chromatography (*n*-hexane/ethyl acetate 10:1, v/v), to afford **S3d** as white solid (450 mg, 3.2 mmol, 54%).

^{1}H NMR (400 MHz, $CDCl_3$) δ 7.00 (d, J = 9.6 Hz, 1H), 3.31 – 3.21 (m, 1H), 2.25 – 2.20 (m, 2H), 2.01 – 1.89 (m, 4H), 1.79 (s, 3H) ppm.

^{13}C{^{1}H} NMR (101 MHz, $CDCl_3$) δ 174.1, 149.5, 125.3, 34.7, 28.7, 19.0, 12.0 ppm.

See NMR Spectra

HRMS (*m/z*): (EI) calc'd for $C_8H_{13}O_2$ $[M]^+$: 141.0910, found: 141.0911.

IR (ATR) ν_{max}: 2963, 2937, 2865, 1680, 1638, 1417, 1278, 938 and 746 cm^{-1}.

Melting point: 51 – 53 °C.

TLC: R_f = 0.13 (*n*-hexane/ethyl acetate 10:1, v/v)

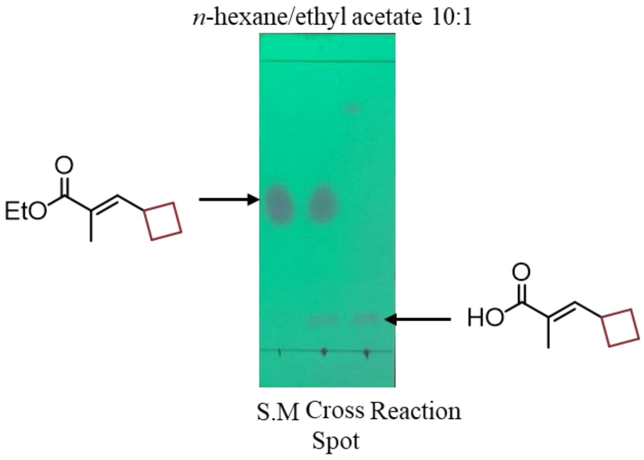

Back to contents

(*E*)-3-Cyclopentyl-2-methylacrylic acid (S3e)

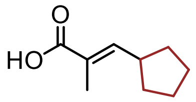

S3e

According to General Procedure C, **S1e** (1.00 g, 5.5 mmol, 1.0 equiv) and KOH (369 mg, 6.6 mmol, 1.2 equiv) were used to obtain the crude residue, which was purified by silica gel column chromatography (*n*-hexane/ethyl acetate 10:1, v/v), to afford **S3e** as a white solid (450 mg, 2.9 mmol, 53%).

^{1}H NMR (300 MHz, $CDCl_3$) δ 6.84 (d, J = 9.6 Hz, 1H), 2.83 – 2.69 (m, 1H), 1.85 (s, 3H), 1.72 – 1.59 (m, 5H), 1.38 – 1.31 (m, 3H) ppm.

^{13}C{^{1}H} NMR (101 MHz, $CDCl_3$) δ 173.7, 150.3, 125.4, 39.5, 32.9, 25.5, 12.1 ppm.

See NMR Spectra

HRMS (*m/z*): (ESI) calc'd for $C_9H_{14}O_2{}^{23}Na$ $[M+Na]^+$: 177.0886, found: 177.0886.

IR (ATR) ν_{max}: 2951, 2862, 1671, 1427, 1295, 1266, 1190, 937 and 754 cm^{-1}.

Melting point: 55 – 58 °C.

TLC: R_f = 0.06 (*n*-hexane/ethyl acetate 10:1, v/v)

n-hexane/ethyl acetate 10:1

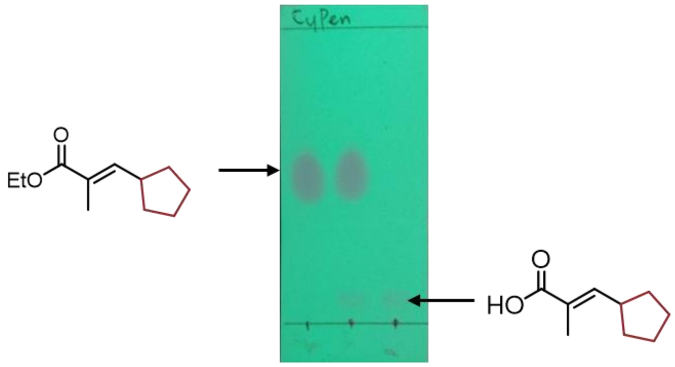

S.M Cross Reaction
Spot

Back to contents

(*E*)-2-Methyl-3-(tetrahydro-2*H*-pyran-4-yl)acrylic acid (S3g)

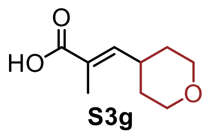

S3g

According to General Procedure C, **S1g** (1.00 g, 5.0 mmol, 1.0 equiv) and KOH (340 mg, 6.1 mmol, 1.2 equiv) were used to obtain the crude residue, which was purified by silica gel column chromatography (*n*-hexane/ethyl acetate 10:1, v/v), to afford **S3g** as a white solid (430 mg, 2.5 mmol, 50%).

^{1}H NMR (300 MHz, $CDCl_3$) δ 6.71 (d, J = 9.5 Hz, 1H), 4.01 – 3.96 (m, 2H), 3.51 – 3.41 (m, 2H), 2.64 – 2.54 (m, 1H), 1.88 (s, 3H), 1.59 – 1.52 (m, 4H) ppm.

^{13}C{^{1}H} NMR (101 MHz, $CDCl_3$) δ 172.4, 147.5, 126.2, 67.2, 67.0, 35.1, 31.3, 28.4, 12.1 ppm.

See NMR Spectra

HRMS (*m/z*): (ESI) calc'd for $C_9H_{14}O_3{}^{23}Na$ $[M+Na]^+$: 193.0835, found: 193.0835.

IR (ATR) ν_{max}: 2943, 2870, 1698, 1650, 1386, 1268, 1225, 1075, 979 and 750 cm^{-1}.

Melting point: 70 – 75 °C.

TLC: R_f = 0.07 (*n*-hexane/ethyl acetate 10:1, v/v)

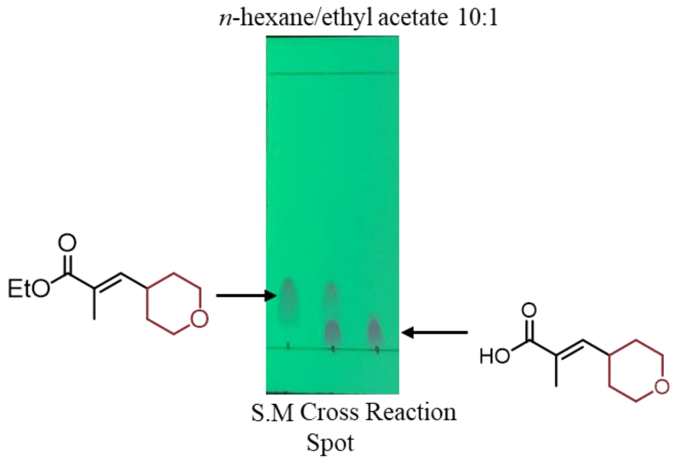

(*E*)-3-(1-(*tert*-Butoxycarbonyl)piperidin-4-yl)-2-methylacrylic acid (S3h)

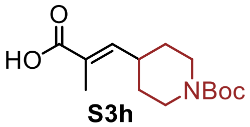

S3h

According to General Procedure C, **S1h** (1.00 g, 3.4 mmol, 1.0 equiv) and KOH (226 mg, 4.0 mmol, 1.2 equiv) were used to obtain the crude residue, which was purified by silica gel column chromatography (*n*-hexane/ethyl acetate 10:1, v/v), to afford **S3h** as a white solid (473 mg, 1.8 mmol, 52%).

^{1}H NMR (300 MHz, $CDCl_3$) δ 9.86 (br.s, 1H), 6.58 (d, J = 6.4 Hz, 1H), 4.01 (s, 2H), 2.70 (s, 2H), 2.40 – 2.39 (m, 1H), 1.79 – 1.76 (m, 3H), 1.38 – 1.35 (m, 9H) ppm.

^{13}C{^{1}H} NMR (101 MHz, $CDCl_3$) δ 172.6, 154.8, 147.3, 126.5, 79.6, 67.6, 36.1, 30.6, 28.4, 12.2 ppm.

See NMR Spectra

HRMS (*m/z*): (ESI) calc'd for $C_{14}H_{23}O_4N^{23}Na$ $[M+Na]^+$: 292.1519, found: 292.1519.

IR (ATR) ν_{max}: 2975, 2929, 2853, 1690, 1425, 1366, 1158, 1006 and 866 cm^{-1}.

Melting point: 85 – 88 °C.

TLC: R_f = 0.23 (*n*-hexane/ethyl acetate 5:1, v/v)

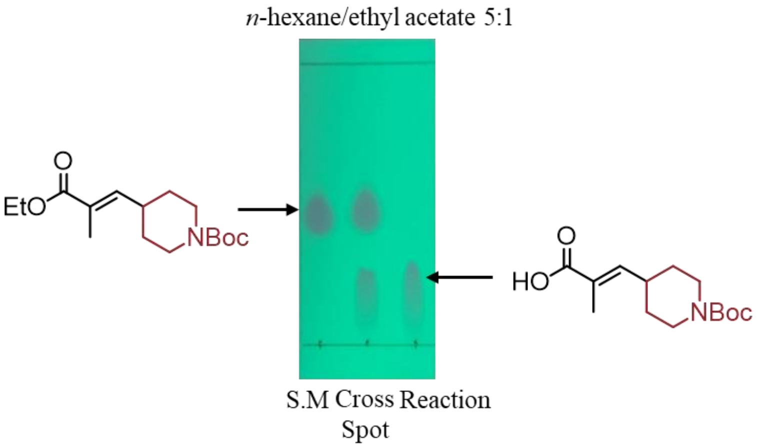

Back to contents

(*E*)-2-Methyl-4-phenylhex-2-enoic acid (S3l)

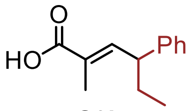

S3l

According to General Procedure C, **S3b** (1.00 g, 4.3 mmol, 1.0 equiv) and KOH (290 mg, 5.2 mmol, 1.2 equiv) were used to obtain the crude residue, which was purified by silica gel column chromatography (*n*-hexane/ethyl acetate 10:1, v/v), to afford **S3l** as a white solid (470 mg, 2.3 mmol, 54%).

^{1}H NMR (400 MHz, $CDCl_3$) δ 7.34 – 7.30 (m, 2H), 7.24 – 7.21 (m, 3H), 7.07 – 7.04 (m, 1H), 3.56 – 3.50 (m, 1H), 1.91 (s, 3H), 1.87 – 1.73 (m, 2H), 0.90 (t, J = 7.4 Hz, 3H) ppm.

^{13}C{^{1}H} NMR (101 MHz, $CDCl_3$) δ 173.8, 147.8, 143.0, 128.7, 127.5, 126.8, 46.8, 29.3, 12.3, 12.0 ppm.

See NMR Spectra

HRMS (*m/z*): (ESI) calc'd for $C_{13}H_{16}O_2{}^{23}Na$ $[M+Na]^+$: 227.1043, found: 227.1041.

IR (ATR) ν_{max}: 2062, 2925, 1682, 1642, 1417, 1287, 1247, 1045 and 935 cm^{-1}.

Melting point: 78 – 80 °C.

TLC: R_f = 0.62 (*n*-hexane/ethyl acetate 1:1, v/v)

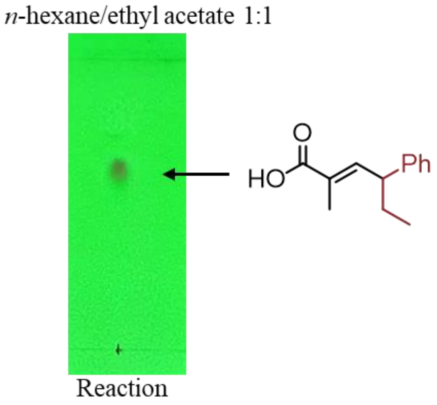

(*E*)-2,5-Dimethyl-4-phenylhex-2-enoic acid (S3m)

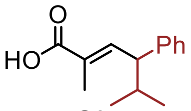

S3m

According to General Procedure C, **S3c** (1.00 g, 4.1 mmol, 1.0 equiv) and KOH (273 mg, 4.9 mmol, 1.2 equiv) were used to obtain the crude residue, which was purified by silica gel column chromatography (*n*-hexane/ethyl acetate 10:1, v/v), to afford **S3m** as a white solid (477 mg, 2.2 mmol, 54%).

^{1}H NMR (300 MHz, $CDCl_3$) δ 7.34 – 7.15 (m, 6H), 3.26 (t, J = 9.8 Hz, 1H), 2.11 – 1.99 (m, 1H), 1.88 (s, 3H), 0.97 (d, J = 6.6 Hz, 3H), 0.81 (d, J = 6.6 Hz, 3H) ppm.

^{13}C{^{1}H} NMR (101 MHz, $CDCl_3$) δ 173.9, 147.2, 142.4, 128.6, 128.0, 126.9, 126.5, 53.0, 33.8, 20.8, 20.6, 12.3 ppm.

See NMR Spectra

HRMS (*m/z*): (ESI) calc'd for $C_{14}H_{18}O_2{}^{23}Na$ [M+Na]$^+$: 241.1199, found: 241.1198.

IR (ATR) ν_{max}: 2957, 2928, 2870, 1682, 1638, 1418, 1288, 1246, 700 and 584 cm^{-1}.

Melting point: 61 – 65 °C.

TLC: R$_f$ = 0.26 (*n*-hexane/ethyl acetate 10:1, v/v)

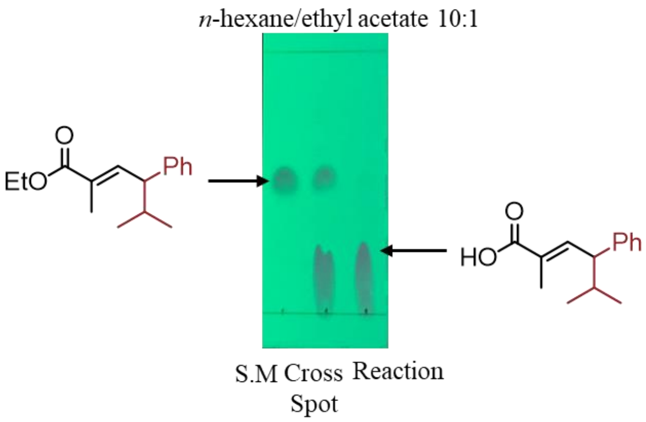

Back to contents

(*E*)-2-Methyl-4-(*p*-tolyl)pent-2-enoic acid (S3n)

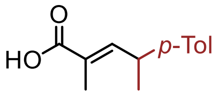

S3n

According to General Procedure C, **S3d** (1.00 g, 4.3 mmol, 1.0 equiv) and KOH (290 mg, 5.2 mmol, 1.2 equiv) were used to obtain the crude residue, which was purified by silica gel column chromatography (*n*-hexane/ethyl acetate 10:1, v/v), to afford **S3n** as a white solid (467 mg, 2.8 mmol, 53%).

^{1}H NMR (600 MHz, $CDCl_3$) δ 7.13 (s, 4H), 7.00 – 6.98 (m, 1H), 3.80 – 3.74 (m, 1H), 2.32 (s, 3H), 1.90 (d, J = 1.0 Hz, 3H), 1.39 (d, J = 6.9 Hz, 3H) ppm.

^{13}C{^{1}H} NMR (151 MHz, $CDCl_3$) δ 173.4, 148.7, 141.2, 136.1, 129.4, 126.8, 125.7, 38.5, 21.3, 21.0, 12.2 ppm.

See NMR Spectra

HRMS (*m/z*): (ESI) calc'd for $C_{13}H_{16}O_2{}^{23}Na$ $[M+Na]^+$: 227.1150, found: 227.1151.

IR (ATR) ν_{max}: 2977, 2922, 1732, 1707, 1514, 1375, 1202, 1022 and 818 cm^{-1}.

Melting point: 64 – 66 °C.

TLC: R_f = 0.23 (*n*-hexane/ethyl acetate 10:1, v/v)

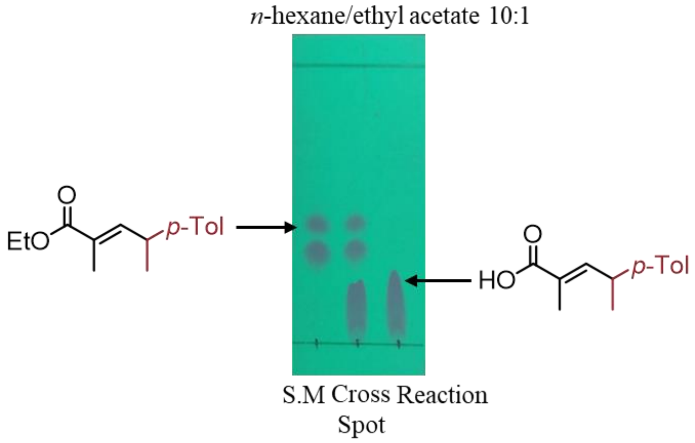

Back to contents

(*E*)-2-Methyl-4,4-diphenylbut-2-enoic acid (S3o)

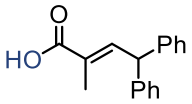

S3o

According to General Procedure C, **1v** (1.10 g, 3.5 mmol, 1.0 equiv) and KOH (236 mg, 4.2 mmol, 1.2 equiv) were used to obtain the crude residue, which was purified by silica gel column chromatography (*n*-hexane/ethyl acetate 10:1, v/v), to afford **S3o** as a white solid (468 mg, 1.9 mmol, 53%).

^{1}H NMR (600 MHz, $CDCl_3$) δ 7.32 – 7.29 (m, 4H), 7.24 – 7.21 (m, 6H), 6.52 – 6.51 (m, 1H), 5.97 (d, J = 10.7 Hz, 1H), 2.03 (s, 3H) ppm.

^{13}C{^{1}H} NMR (151 MHz, $CDCl_3$) δ 172.5, 146.1, 143.4, 128.5, 128.4, 126.5, 126.1, 49.2, 20.9 ppm.

See NMR Spectra

HRMS (*m/z*): (EI) calc'd for $C_{17}H_{16}O_2$ $[M]^+$: 252.1145, found: 252.1146.

IR (ATR) ν_{max}: 3024, 1683, 1493, 1454, 1251, 914, and 696 cm^{-1}.

Melting point: 86 – 88 °C.

TLC: R_f = 0.09 (*n*-hexane/ethyl acetate 5:1, v/v)

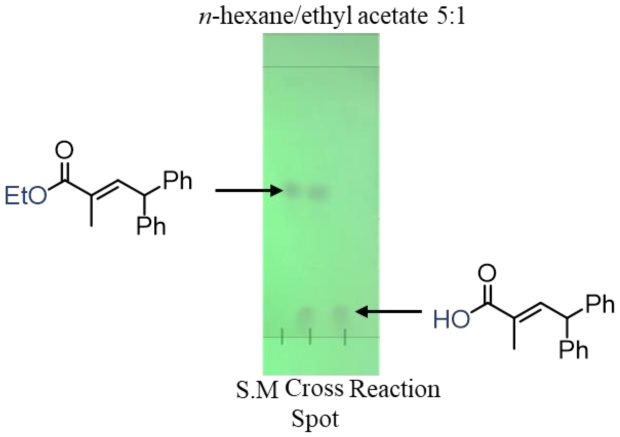

[Back to contents](#)

(*E*)-2-Ethyl-4,4-diphenylbut-2-enoic acid (S3p)

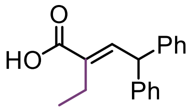

S3p

According to General Procedure C, **S2a** (440 mg, 1.5 mmol, 1.0 equiv) and KOH (101 mg, 1.8 mmol, 1.2 equiv) were used to obtain the crude residue, which was purified by silica gel column chromatography (*n*-hexane/ethyl acetate 6:1, v/v), to afford **S3p** as a white solid (196 mg, 0.7 mmol, 49%).

^{1}H NMR (300 MHz, $CDCl_3$) δ 7.33 – 7.28 (m, 4H), 7.24 – 7.19 (m, 6H), 6.44 (d, J = 10.6 Hz, 1H), 5.88 (d, J = 10.6 Hz, 1H), 2.40 (q, J = 7.2 Hz, 2H), 1.12 (t, J = 7.4 Hz, 3H) ppm.

^{13}C{^{1}H} NMR (101 MHz, $CDCl_3$) δ 172.4, 143.9, 143.5, 132.5, 128.5, 128.4, 126.5, 49.1, 27.6, 13.6 ppm.

See NMR Spectra

HRMS (*m/z*): (ESI) calc'd for $C_{18}H_{18}O_2{}^{23}Na$ $[M+Na]^+$: 289.1199, found: 289.1199.

IR (ATR) ν_{max}: 3024, 1683, 1493, 1454, 1251, 1147, 914, 741 and 696 cm^{-1}.

Melting point: 91 – 94 °C.

TLC: R_f = 0.05 (*n*-hexane/ethyl acetate 6:1, v/v)

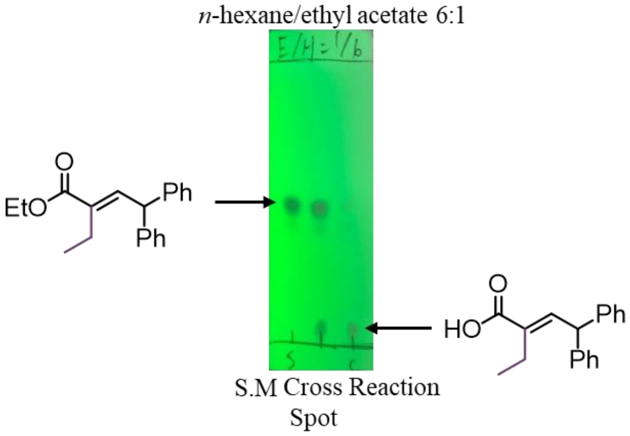

Back to contents

(*E*)-2-(2,2-Diphenylethylidene)pentanoic acid (S3q)

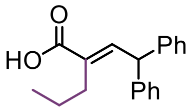

S3q

According to General Procedure C, **S2b** (500 mg, 1.6 mmol, 1.0 equiv) and KOH (107 mg, 1.9 mmol, 1.2 equiv) were used to obtain the crude residue, which was purified by silica gel column chromatography (*n*-hexane/ethyl acetate 6:1, v/v), to afford **S3q** as a white solid (254 mg, 0.9 mmol, 56%).

^{1}H NMR (400 MHz, $CDCl_3$) δ 7.33 – 7.29 (m, 4H), 7.24 – 7.20 (m, 6H), 6.45 (d, J = 10.7 Hz, 1H), 5.90 (d, J = 10.7 Hz, 1H), 2.34 (t, J = 7.5 Hz, 2H), 1.60 – 1.50 (m, 2H), 0.93 (t, J = 7.3 Hz, 3H) ppm.

^{13}C{^{1}H} NMR (101 MHz, $CDCl_3$) δ 173.2, 145.1, 143.4, 130.9, 128.5, 128.4, 126.5, 49.2, 36.7, 22.3, 13.7 ppm.

See NMR Spectra

HRMS (*m/z*): (EI) calc'd for $C_{19}H_{20}O_2$ $[M]^+$: 280.1458, found 279.1456.

IR (ATR) ν_{max}: 2928, 1730, 1444, 1165, 759, 702 and 473 cm^{-1}.

Melting point: 98 – 101 °C.

TLC: R_f = 0.30 (*n*-hexane/ethyl acetate 4:1, v/v)

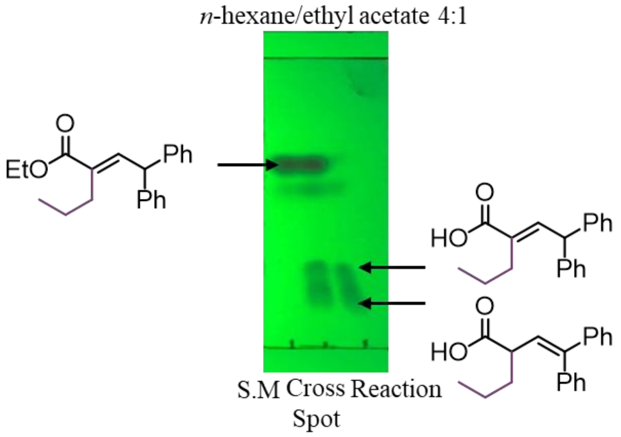

Back to contents

(*E*)-2-(2,2-Diphenylethylidene)hexanoic acid (S3r)

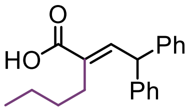

S3r

According to General Procedure C, **S2c** (100 mg, 0.3 mmol, 1.0 equiv) and KOH (20 mg, 0.4 mmol, 1.2 equiv) were used to obtain the crude residue, which was purified by silica gel column chromatography (*n*-hexane/ethyl acetate 6:1, v/v), to afford **S3r** as a white solid (33 mg, 0.1 mmol, 36%).

^{1}H NMR (400 MHz, $CDCl_3$) δ 7.29 – 7.25 (m, 4H), 7.22 – 7.19 (m, 6H), 6.42 (d, J = 10.1 Hz, 1H), 5.86 (d, J = 10.1 Hz, 1H), 2.35 (t, J = 6.8 Hz, 2H), 1.48 (t, J = 6.1 Hz, 2H), 1.32 (dt, J = 14.2, 7.0 Hz, 2H), 0.90 (t, J = 6.8 Hz, 3H) ppm.

^{13}C{^{1}H} NMR (101 MHz, $CDCl_3$) δ 172.8, 144.6, 143.5, 128.5, 128.4, 128.2, 126.5, 49.2, 34.4, 31.3, 22.3, 13.9 ppm.

See NMR Spectra

HRMS (*m/z*): (EI) calc'd for $C_{20}H_{22}O_2$ $[M]^+$: 294.1620, found 294.1621.

IR (ATR) ν_{max}: 2975, 1726, 1492, 1221, 1161, 765 and 698 cm^{-1}.

Melting point: 105 – 108 °C.

TLC: R_f = 0.35 (*n*-hexane/ethyl acetate 6:1, v/v)

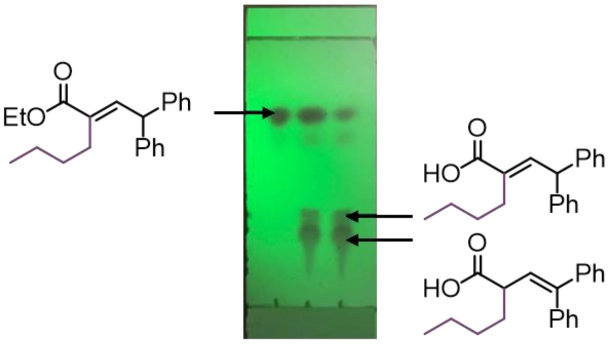

[Back to contents](#)

(*E*)-2-(2,2-Diphenylethylidene)pent-4-enoic acid (S3s)

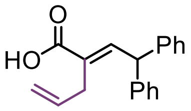

S3s

According to General Procedure C, **S2d** (200 mg, 0.7 mmol, 1.0 equiv) and KOH (47 mg, 0.8 mmol, 1.2 equiv) were used to obtain the crude residue, which was purified by silica gel column chromatography (*n*-hexane/ethyl acetate 6:1, v/v), to afford **S3s** as a white solid (65 mg, 0.2 mmol, 35%).

^{1}H NMR (400 MHz, $CDCl_3$) δ 7.34 – 7.30 (m, 4H), 7.25 – 7.20 (m, 6H), 6.52 (d, J = 10.7 Hz, 1H), 5.96 (d, J = 10.9 Hz, 1H), 5.93 – 5.85 (m, 1H), 5.14 – 5.09 (m, 2H), 3.13 (d, J = 6.3 Hz, 2H) ppm.

^{13}C{^{1}H} NMR (101 MHz, $CDCl_3$) δ 172.7, 146.4, 143.3, 135.4, 129.0, 128.6, 128.4, 126.5, 116.9, 49.1, 38.1 ppm.

See NMR Spectra

HRMS (*m/z*): (ESI) calc'd for $C_{19}H_{18}O_2{}^{23}Na$ [M+Na]$^{+}$: 301.1199, found 301.1200.

IR (ATR) ν_{max}: 3061, 2910, 1704, 1492, 1198, 698 and 501 cm^{-1}.

Melting point: 101 – 103 °C.

TLC: R$_f$ = 0.12 (*n*-hexane/ethyl acetate 4:1, v/v)

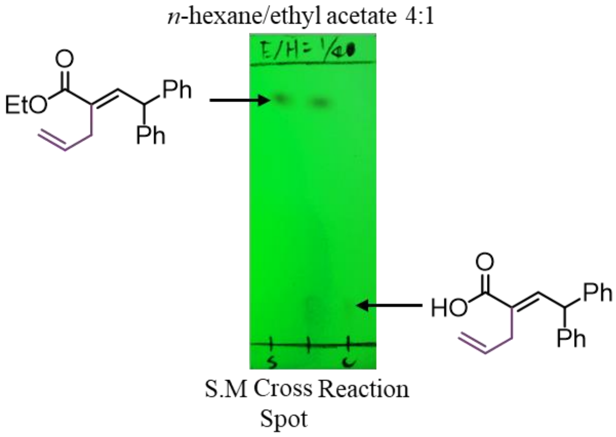

Back to contents

(*E*)-2-(2,2-Diphenylethylidene)hex-5-enoic acid (S3t)

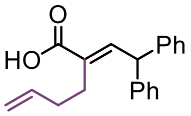

S3t

According to General Procedure C, **S2e** (150 mg, 0.5 mmol, 1.0 equiv) and KOH (34 mg, 0.6 mmol, 1.2 equiv) were used to obtain the crude residue, which was purified by silica gel column chromatography (*n*-hexane/ethyl acetate 100:1, v/v), to afford **S3t** as colorless oil (123 mg, 0.5 mmol, 90%).

^{1}H NMR (300 MHz, $CDCl_3$) δ 7.30 – 7.27 (m, 5H), 7.24 – 7.21 (m, 5H), 6.46 (d, J = 10.5 Hz, 1H), 5.89 (d, J = 10.6 Hz, 1H), 5.84 – 5.73 (m, 1H), 5.00 (t, J = 14.2 Hz, 2H), 2.45 (d, J = 7.6 Hz, 2H), 2.28 (d, J = 6.9 Hz, 2H) ppm.

^{13}C{^{1}H} NMR (101 MHz, $CDCl_3$) δ 172.7, 146.0, 143.3, 137.5, 130.1, 128.5, 128.4, 126.5, 115.5, 49.2, 34.0, 33.3 ppm.

See NMR Spectra

HRMS (*m/z*): (EI) calc'd for $C_{20}H_{20}O_2$ $[M]^+$: 292.1458, found: 292.1460.

IR (ATR) ν_{max}: 3072, 2934, 1716, 1502, 1198, 1124 and 698 cm^{-1}.

TLC: R_f = 0.06 (*n*-hexane/ethyl acetate 20:1, v/v)

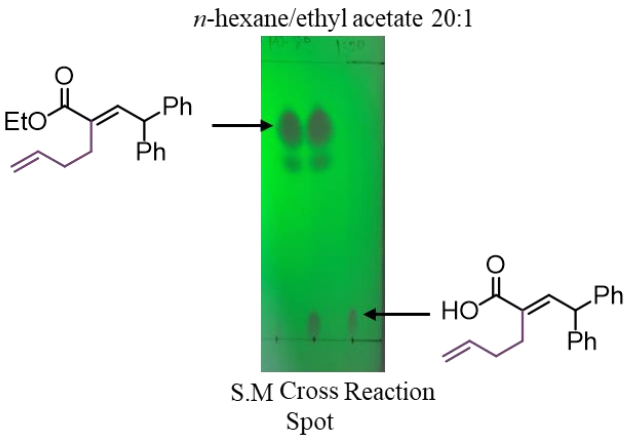

2.3 General Procedure D: Synthesis of photodeconjugation precursors

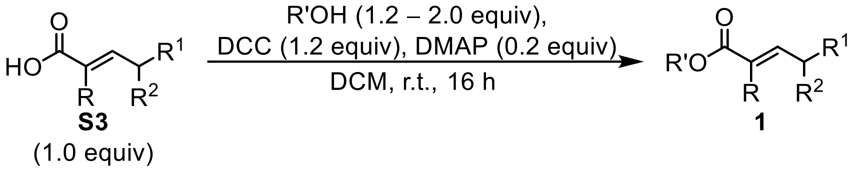

An oven-dried flask containing a solution of compound **S3** (1.0 equiv) in dry DCM (0.1 M) was added DCC (1.2 equiv) and DMAP (0.2 equiv) and stirred at r.t. for 15 minutes. The alcohol (1.2 – 2.0 equiv) was then added to the solution and stirred for 16 h. The resulting mixture was filtered through a celite pad, washed with diethyl ether, and the filtrate was concentrated under reduced pressure. The crude residue was purified by column chromatography (height of packing silica gel: 6.0 – 8.0 cm; dry loading, gradient elution) to afford desired product **1**.

2.4 General Procedure E: Photodeconjugation of *α,β*-unsaturated esters

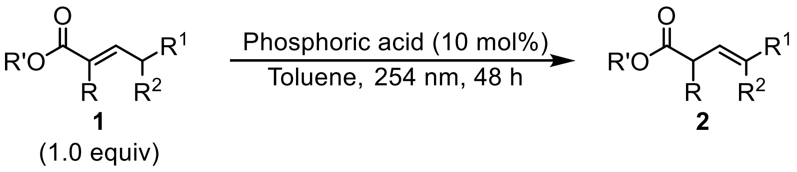

A quartz tube containing a stirring bar was charged with compound **1** (1.0 equiv) and phosphoric acid (10 mol%) in degassed toluene (0.05 M) under nitrogen condition. The resulting mixture was irradiated at 254 nm for 48 h. The reaction was then concentrated under reduced pressure. The crude residue was purified by column chromatography (height of packing silica gel: 6.0 – 8.0 cm; wet loading, gradient elution) to afford compound **2**.

2.5 General Procedure F: Synthesis of *α*-deuterated *α,β*-unsaturated esters

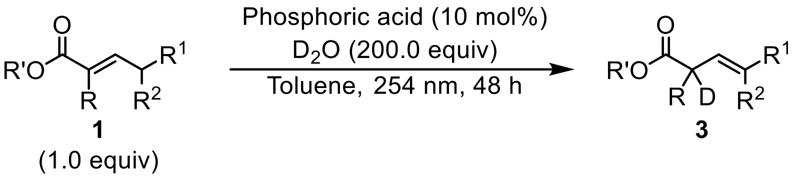

A quartz tube containing a stirring bar was charged with compound **1** (1.0 equiv) and phosphoric acid (10 mol%) in degassed toluene (0.05 M) under nitrogen condition. The resulting mixture was added D_2O (200.0 equiv) and was irradiated at 254 nm for 48 h. The reaction was then concentrated under reduced pressure. The crude residue was purified by column chromatography (height of packing silica gel: 6.0 – 8.0 cm; wet loading, gradient elution) to afford compound **3**.

3. Optimization Studies

3.1 Photodeconjugation of symmetric *ene* scopes

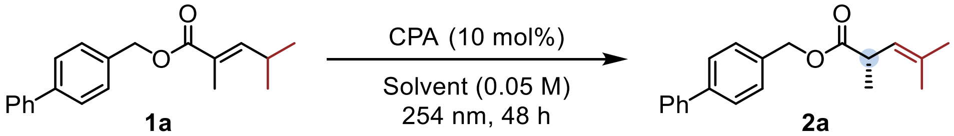

General procedure of reaction optimization: A quartz tube with septum under nitrogen was charged with **1a** (0.05 mmol, 1.0 equiv) and chiral phosphoric acid (10 mol%). Subsequently, degassed solvent (1.0 mL, 0.05 M) was added, and the mixture was irradiated at 254 nm for 48 h. The reaction mixture was concentrated under reduced pressure and high vacuum. The crude residue was diluted with chloroform-d_1, and tetrachloroethane (CAS# 79-34-5, 17 – 20 mg) was added as NMR internal standard. The yield of **2a** was determined by ^{1}H NMR analysis (**Figure S3.1**).

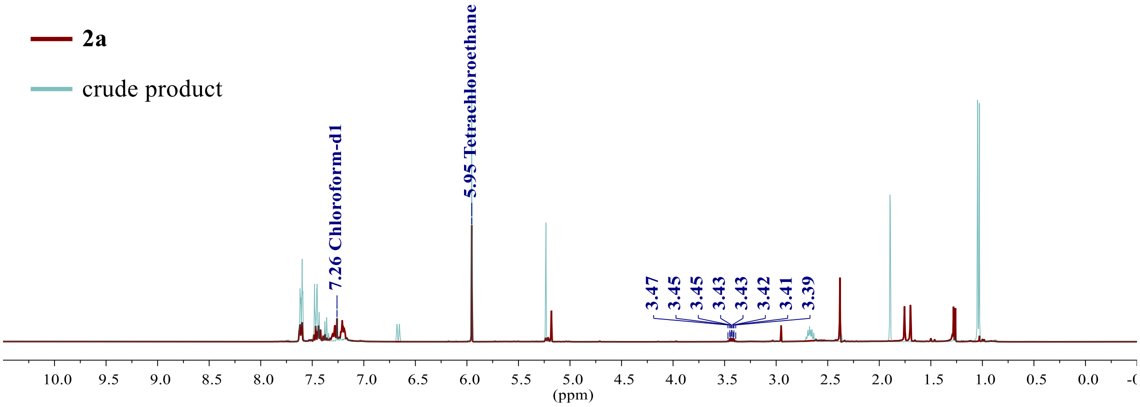

Figure S3.1 The chemical shift (δ) of tetrachloroethane is 5.96 ppm and the characterization peak of **2a** was 3.47 – 3.39 (m, 1H) ppm.

Back to contents

Table S3.1 CPA screening.

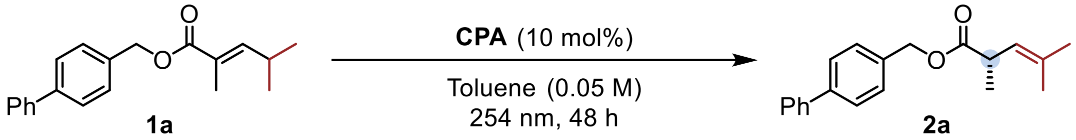

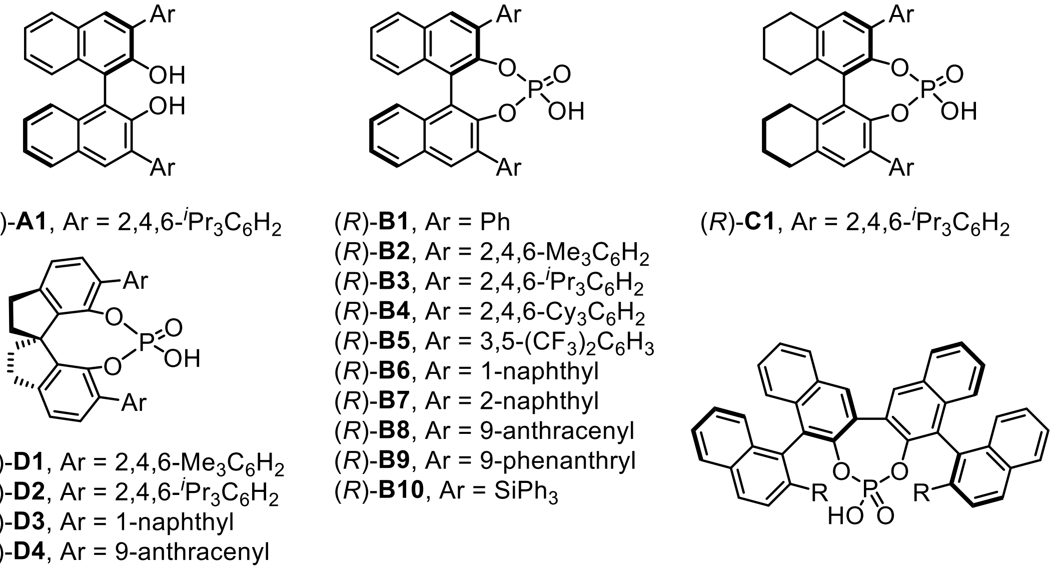

(*R*)-**A1**, Ar = 2,4,6-^{i}Pr$_3$C$_6$H$_2$

(*R*)-**B1**, Ar = Ph
(*R*)-**B2**, Ar = 2,4,6-Me$_3$C$_6$H$_2$
(*R*)-**B3**, Ar = 2,4,6-^{i}Pr$_3$C$_6$H$_2$
(*R*)-**B4**, Ar = 2,4,6-Cy$_3$C$_6$H$_2$
(*R*)-**B5**, Ar = 3,5-(CF$_3$)$_2$C$_6$H$_3$
(*R*)-**B6**, Ar = 1-naphthyl
(*R*)-**B7**, Ar = 2-naphthyl
(*R*)-**B8**, Ar = 9-anthracenyl
(*R*)-**B9**, Ar = 9-phenanthryl
(*R*)-**B10**, Ar = SiPh$_3$

(*R*)-**C1**, Ar = 2,4,6-^{i}Pr$_3$C$_6$H$_2$

(*R*)-**D1**, Ar = 2,4,6-Me$_3$C$_6$H$_2$
(*R*)-**D2**, Ar = 2,4,6-^{i}Pr$_3$C$_6$H$_2$
(*R*)-**D3**, Ar = 1-naphthyl
(*R*)-**D4**, Ar = 9-anthracenyl
(*R*)-**D5**, Ar = 9-phenanthryl

(*R*)-**E1**, R = Cy

Entry	CPA	Yield of **2a** (%)	*ee* of **2a** (%)	Entry	CPA	Yield of **2a** (%)	*ee* of **2a** (%)
1	(*R*)-**A1**	77	0	10	(*R*)-**B9**	76	−38
2	(*R*)-**B1**	70	−67	11	(*R*)-**B10**	75	−38
3	(*R*)-**B2**	90	−74	12	(*R*)-**C1**	83	−36
4	(*R*)-**B3**	96	−50	13	(*R*)-**D1**	77	87
5	(*R*)-**B4**	64	−65	14	(*R*)-**D2**	72	36
6	(*R*)-**B5**	50	−60	15	(*R*)-**D3**	89	94
7	(*R*)-**B6**	65	−48	16	(*R*)-**D4**	50	83
8	(*R*)-**B7**	91	−60	17	(*R*)-**D5**	73	90
9	(*R*)-**B8**	85	−71	18	(*R*)-**E1**	52	46

Table S3.2 Solvent screening.

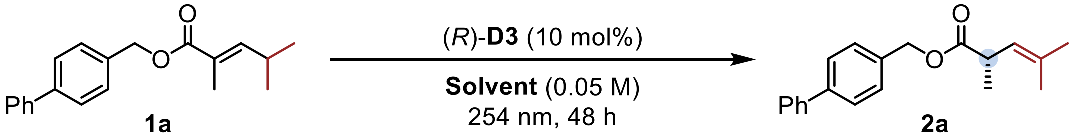

Entry	Solvent	Yield of **2a** (%)	*ee* of **2a** (%)	Entry	Solvent	Yield of **2a** (%)	*ee* of **2a** (%)
1	*n*-Hexane	91	78	4	HFIP	45	9
2	CH$_2$Cl$_2$	81	83	5	Toluene	89	94
3	THF	95	77	6	Toluene (0.025M)	88	94

Table S3.3 Additive screening.

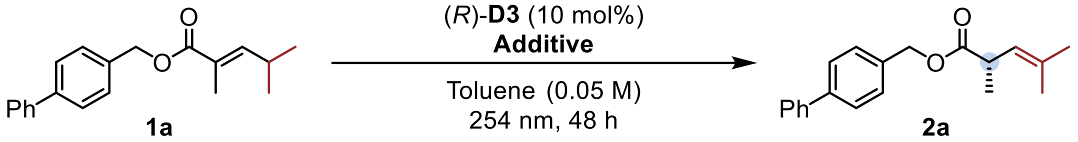

Entry	Additive[a]	Yield of **2a** (%)	*ee* of **2a** (%)	Entry	Additive[a]	Yield of **2a** (%)	*ee* of **2a** (%)
1	4Å MS	75	94	3	$MgSO_4$	67	41
2	3Å MS	51	94	4	Na_2SO_4	75	42

[a]*The reaction was pre-stirred for 30 min before irradiation*

To confirm the effect of water on the reaction, 4Å MS and 3Å MS were added but observed no change in the ee value. Furthermore, the addition of $MgSO_4$ and Na_2SO_4 resulted in a decrease in the ee value of the reaction.

3.2 Photodeconjugation of unsymmetric *ene* scopes

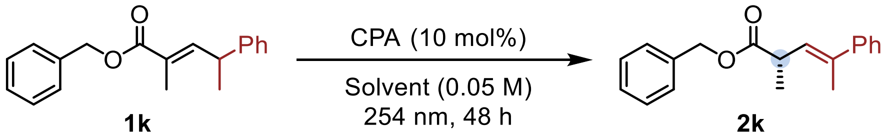

General procedure of reaction optimization: A quartz tube with septum under nitrogen was charged with **3a** (0.05 mmol, 1.0 equiv) and chiral phosphoric acid (10 mol%). Subsequently, degassed solvent (1.0 mL, 0.05 M) was added, and the mixture was irradiated at 254 nm for 48 h. The reaction mixture was concentrated under reduced pressure and high vacuum. The crude residue was purified by silica gel column chromatography (Hexane/ethyl acetate 100:1, v/v) to afford **2k**.

Table S3.4 CPA screening.

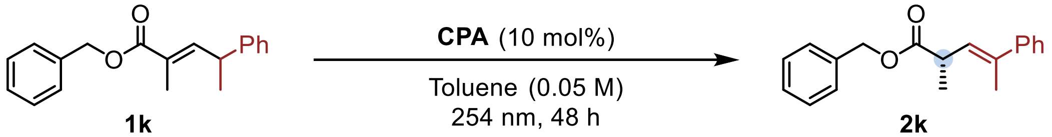

1k **2k**

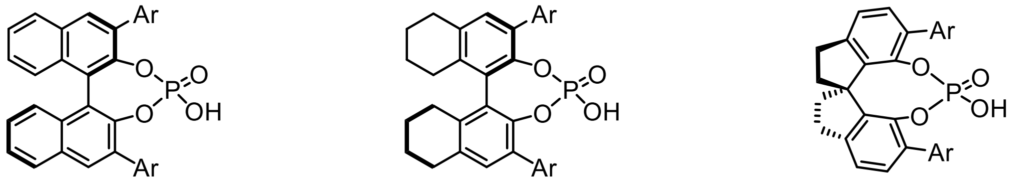

(*R*)-**B1**, Ar = Ph
(*R*)-**B3**, Ar = 2,4,6-$^{i}Pr_3C_6H_2$
(*R*)-**B6**, Ar = 1-naphthyl
(*R*)-**B8**, Ar = 9-anthracenyl
(*R*)-**B9**, Ar = 9-phenanthryl

(*R*)-**C1**, Ar = 2,4,6-$^{i}Pr_3C_6H_2$

(*R*)-**D1**, Ar = 2,4,6-$Me_3C_6H_2$
(*R*)-**D2**, Ar = 2,4,6-$^{i}Pr_3C_6H_2$
(*R*)-**D3**, Ar = 1-naphthyl
(*R*)-**D4**, Ar = 9-anthracenyl
(*R*)-**D5**, Ar = 9-phenanthryl

Entry	CPA	Yield of **2k** (%)	*ee* of **2k** (%)	Entry	CPA	Yield of **2k** (%)	*ee* of **2k** (%)
1	(*R*)-**B1**	59 (1.8:1)	− 11/15	7	(*R*)-**D1**	82 (1.5:1)	78/71
2	(*R*)-**B3**	82 (1.5:1)	− 58/46	8	(*R*)-**D2**	75 (1.7:1)	18/21
3	(*R*)-**B6**	52 (1.6:1)	− 67/66	9	(*R*)-**D3**	52 (1.7:1)	83/80
4	(*R*)-**B8**	79 (1.4:1)	− 63/68	10	(*R*)-**D4**	68 (1.4:1)	86/78
5	(*R*)-**B9**	60 (1.6:1)	− 40/39	11	(*R*)-**D5**	72 (2.0:1)	93/94
6	(*R*)-**C1**	95 (1.4:1)	− 56/44				

3.3 Photodeconjugation of ester scopes

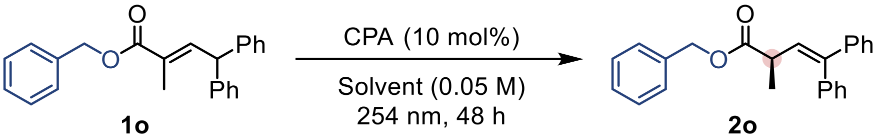

1o **2o**

General procedure of reaction optimization: A quartz tube with septum under nitrogen was charged with **5a** (0.05 mmol, 1.0 equiv) and chiral phosphoric acid (10 mol%). Subsequently, degassed solvent (1.0 mL, 0.05 M) was added, and the mixture was irradiated at 254 nm for 48 h. The reaction mixture was concentrated under reduced pressure and high vacuum. The crude residue was purified by silica gel column chromatography (Hexane/ethyl acetate 100:1, v/v) to afford **2o**.

Table S3.5 CPA screening.

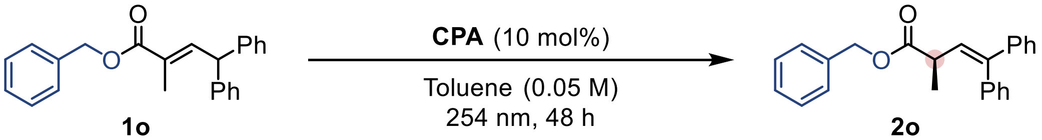

1o 2o

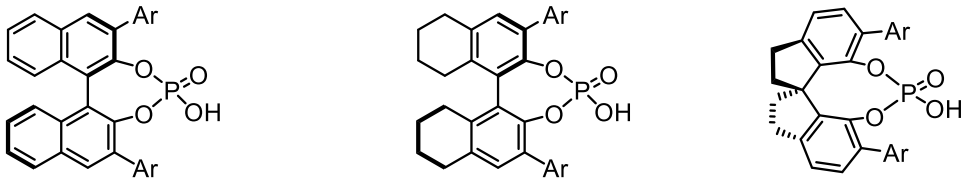

(*R*)-**B1**, Ar = Ph
(*R*)-**B2**, Ar = 2,4,6-$Me_3C_6H_2$
(*R*)-**B3**, Ar = 2,4,6-$^{i}Pr_3C_6H_2$
(*R*)-**B4**, Ar = 2,4,6-$Cy_3C_6H_2$
(*R*)-**B6**, Ar = 1-naphthyl
(*R*)-**B7**, Ar = 2-naphthyl
(*R*)-**B8**, Ar = 9-anthracenyl
(*R*)-**B9**, Ar = 9-phenanthryl

(*R*)-**C1**, Ar = 2,4,6-$^{i}Pr_3C_6H_2$

(*R*)-**D2**, Ar = 2,4,6-$^{i}Pr_3C_6H_2$

Entry	CPA	Yield of **2o** (%)	*ee* of **2o** (%)	Entry	CPA	Yield of **2o** (%)	*ee* of **2o** (%)
1	(*R*)-**B1**	91	−60	6	(*R*)-**B7**	95	−51
2	(*R*)-**B2**	98	−87	7	(*R*)-**B8**	90	−78
3	(*R*)-**B3**	90	−90	8	(*R*)-**B9**	70	−65
4	(*R*)-**B4**	84	−70	9	(*R*)-**C1**	62	−85
5	(*R*)-**B6**	81	−70	10	(*R*)-**D2**	59	74

Table S3.6 Solvent screening.

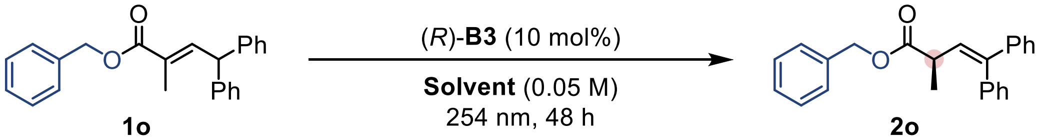

1o 2o

Entry	Solvent	Yield of **2o** (%)	*ee* of **2o** (%)	Entry	Solvent	Yield of **2o** (%)	*ee* of **2o** (%)
1	*n*-Hexane	66	−87	9	Chlorobenzene	17	−58
2	Hexane with 4A MS	68	−89	10	CH_2Cl_2	57	−82
3	Toluene	90	−90	11	$CHCl_3$	35	−6
4	Toluene with 4A MS	85	−90	12	*p*-Xylene	77	−69
5	*n*-Hexane/Toluene (10:1)	76	−89	13	1,4-Dioxane	22	−50
6	*n*-Hexane/Toluene (1:1)	70	−88	14	THF	60	−27
7	*n*-Hexane/Toluene (1:10)	47	−84	15	HFIP	19	−9
8	Cyclohexane	66	−78				

Back to contents / Back to substrate scope table

4. Overview of Substrate Scope

Table S4.1 Substrate scope of compound **1**.

Precursors of symmetric and unsymmetric *ene* scopes		
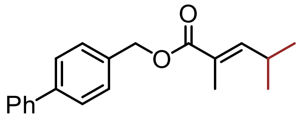 Compound **1a**	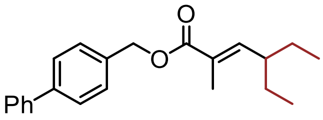 Compound **1b**	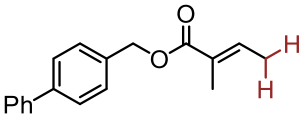 Compound **1c**
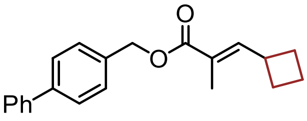 Compound **1d**	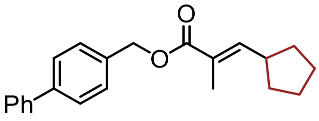 Compound **1e**	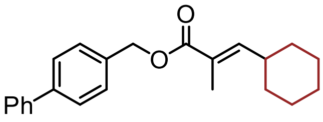 Compound **1f**
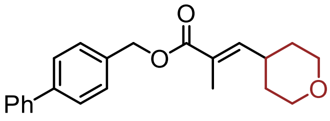 Compound **1g**	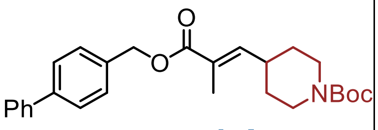 Compound **1h**	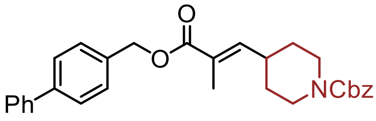 Compound **1i**
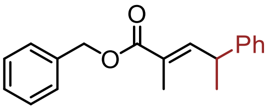 Compound **1k**	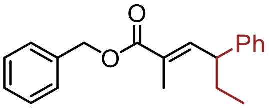 Compound **1l**	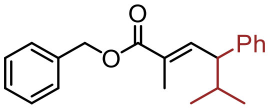 Compound **1m**
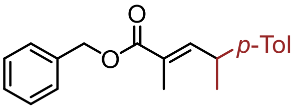 Compound **1n**	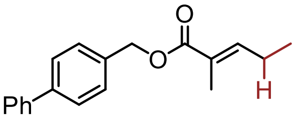 Compound **1n'**	
**Precursors of ester scopes**		
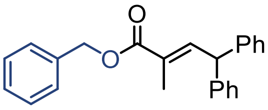 Compound **1o**	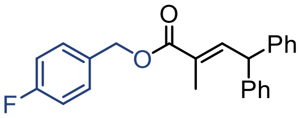 Compound **1p**	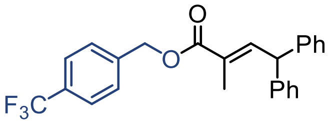 Compound **1q**
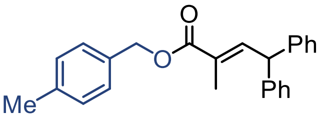 Compound **1r**	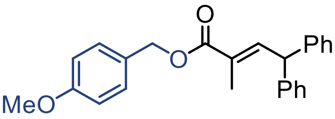 Compound **1s**	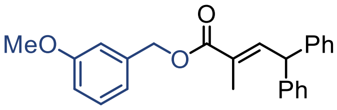 Compound **1t**
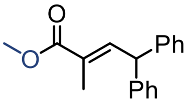 Compound **1u**	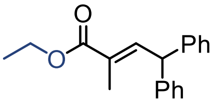 Compound **1v**	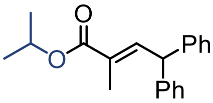 Compound **1w**
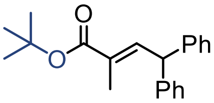 Compound **1x**	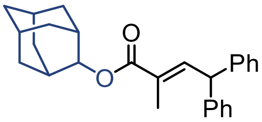 Compound **1y**	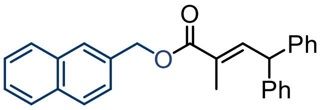 Compound **1z**

**Precursors of *α*-substituted scopes**		
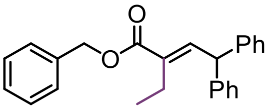 Compound **1aa**	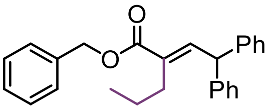 Compound **1ab**	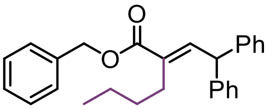 Compound **1ac**
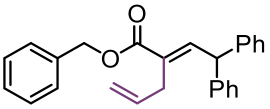 Compound **1ad**	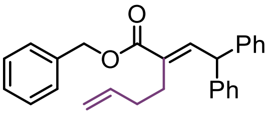 Compound **1ae**	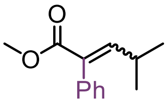 Compound **1af + (*Z*)-1af**
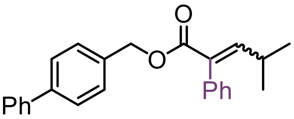 Compound **1ag + (*Z*)-1ag**	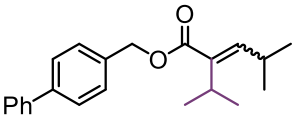 Compound **1ah + (*Z*)-1ah**	

**Precursors of biorelevant scopes**	
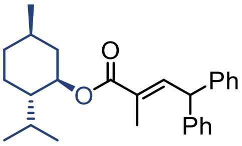 Compound **1ai**	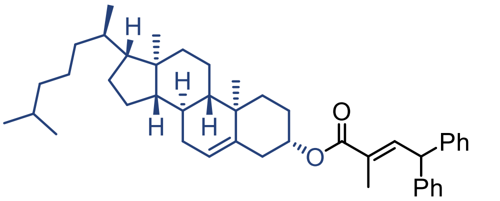 Compound **1aj**
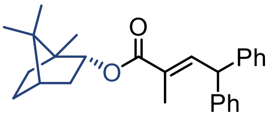 Compound **1ak**	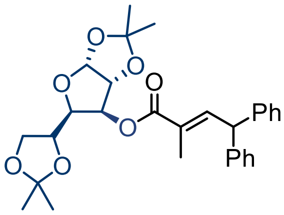 Compound **1al**

Table S4.2 Substrate scope of compound **2**.

**Symmetric and unsymmetric *ene* scopes**		
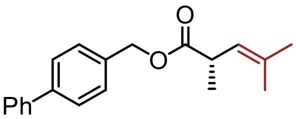 Compound **2a**	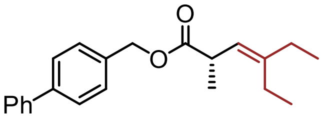 Compound **2b**	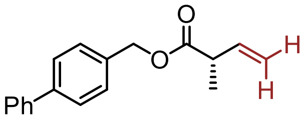 Compound **2c**
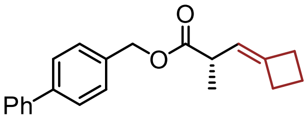 Compound **2d**	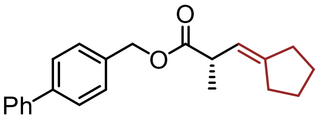 Compound **2e**	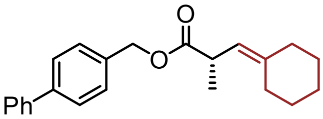 Compound **2f**
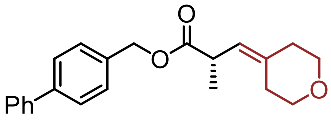 Compound **2g**	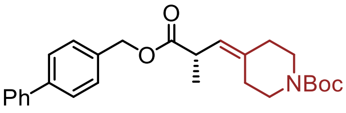 Compound **2h**	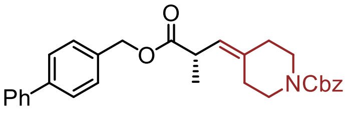 Compound **2i**

Back to contents / Back to substrate scope table

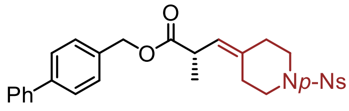 Compound **2j**	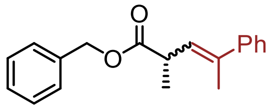 Compound **2k**	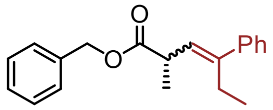 Compound **2l**
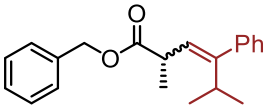 Compound **2m**	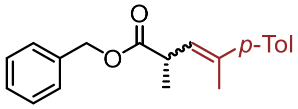 Compound **2n**	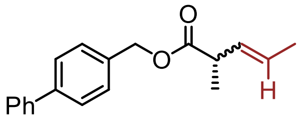 Compound **2n'**
**Ester scopes**		
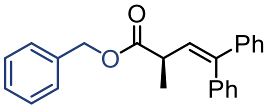 Compound **2o**	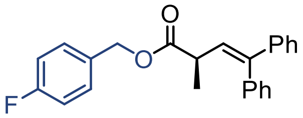 Compound **2p**	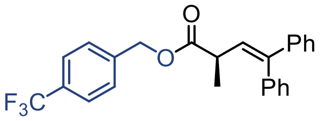 Compound **2q**
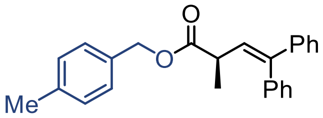 Compound **2r**	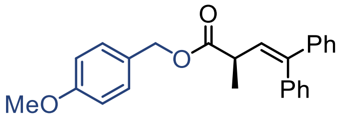 Compound **2s**	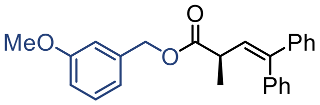 Compound **2t**
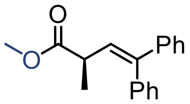 Compound **2u**	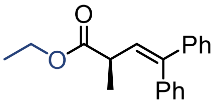 Compound **2v**	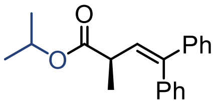 Compound **2w**
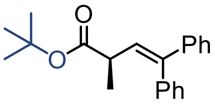 Compound **2x**	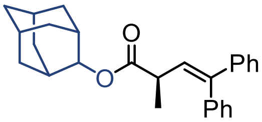 Compound **2y**	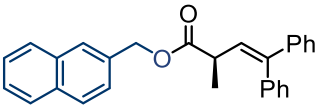 Compound **2z**
***α*-Substituted scopes**		
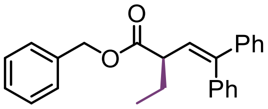 Compound **2aa**	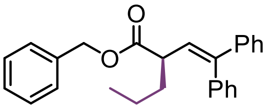 Compound **2ab**	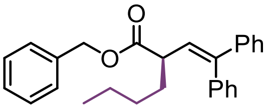 Compound **2ac**
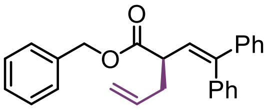 Compound **2ad**	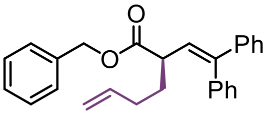 Compound **2ae**	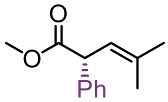 Compound **2af**
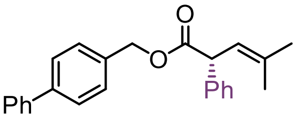 Compound **2ag**	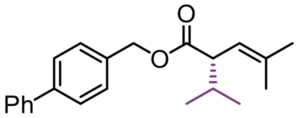 Compound **2ah**	

Back to contents / Back to substrate scope table

Biorelevant scopes	
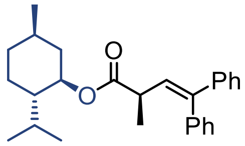 Compound **2ai**	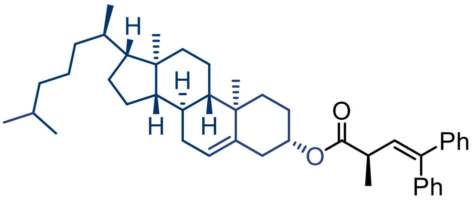 Compound **2aj**
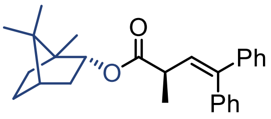 Compound **2ak**	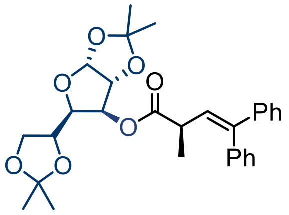 Compound **2al**

Table S4.3 Substrate scope of compound **3**.

Deteurium scopes		
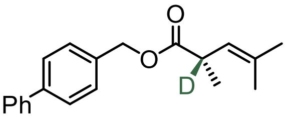 Compound **3a**	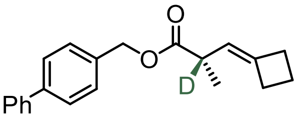 Compound **3b**	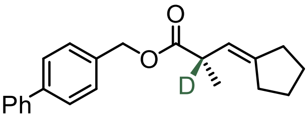 Compound **3c**
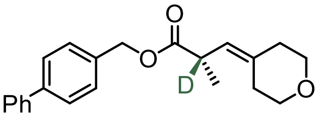 Compound **3d**	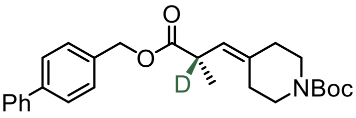 Compound **3e**	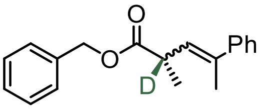 Compound **3f**
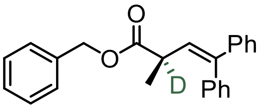 Compound **3g**	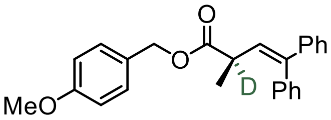 Compound **3h**	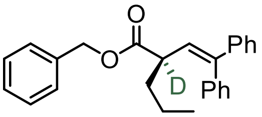 Compound **3i**

Table S4.4 Substrate scope of compound **S5**.

Ketone scopes	
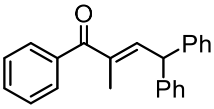 Compound **S5a**	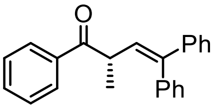 Compound **S5b**

Back to contents / Back to substrate scope table

Table S4.5 Unsuccessful examples.

**Unsuccessful photodeconjugation products**			
Structure	Result	Structure	Result
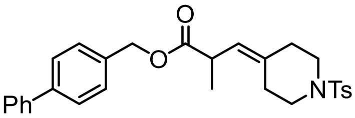	No desired product was observed.	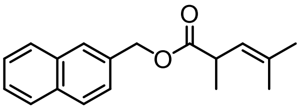	No desired product was observed.
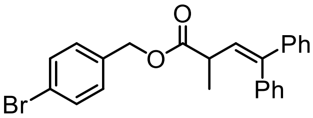	No desired product was observed.	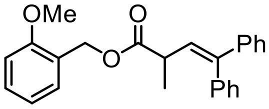	Trace product was observed from ^{1}H NMR.
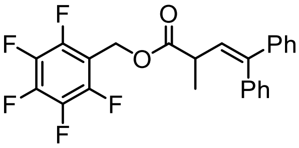	No desired product was observed.	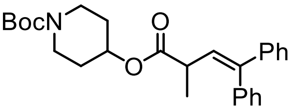	No desired product was observed.
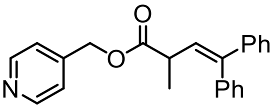	No desired product was observed.	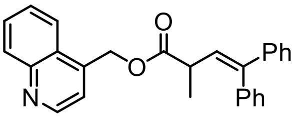	No desired product was observed.

Back to contents / Back to substrate scope table

4.1 Characterization data of compound 1

[1,1'-Biphenyl]-4-ylmethyl (*E*)-2,4-dimethylpent-2-enoate (1a)

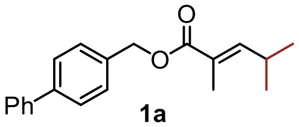

1a

According to General Procedure D, **S3a** (0.10 g, 0.6 mmol, 1.0 equiv), [1,1'-biphenyl]-4-ylmethanol (177 mg, 1.0 mmol, 1.5 equiv), DCC (158 mg, 0.8 mmol, 1.2 equiv), and DMAP (16 mg, 0.1 mmol, 0.2 equiv) were used to obtain the crude residue, which was purified by silica gel column chromatography (*n*-hexane/ethyl acetate 100:1, v/v), to afford **1a** as a white solid (105 mg, 0.3 mmol, 56%).

^{1}H NMR (400 MHz, $CDCl_3$) δ 7.61 – 7.59 (m, 4H), 7.47 – 7.43 (m, 4H), 7.37 – 7.33 (m, 1H), 6.65 (dd, J = 9.7, 0.9 Hz, 1H), 5.23 (s, 2H), 2.70 – 2.61 (m, 1H), 1.88 (d, J = 0.8 Hz, 3H), 1.03 (d, J = 6.7 Hz, 6H) ppm.

^{13}C{^{1}H} NMR (101 MHz, $CDCl_3$) δ 168.4, 149.6, 141.0, 140.7, 135.5, 128.8, 128.5, 127.4, 127.3, 127.1, 125.4, 66.0, 28.0, 21.9, 12.3 ppm.

See NMR Spectra

HRMS (*m/z*): (ESI) calc'd for $C_{20}H_{22}O_2{}^{23}Na$ [M+Na]$^{+}$: 317.1512, found: 317.1511.

IR (ATR) ν_{max}: 2927, 2853, 2114, 1711, 1448, 1300, 1240, 1045, 891 and 761 cm^{-1}.

Melting point: 49 – 53 °C.

TLC: R_f = 0.83 (*n*-hexane/ethyl acetate 3:1, v/v)

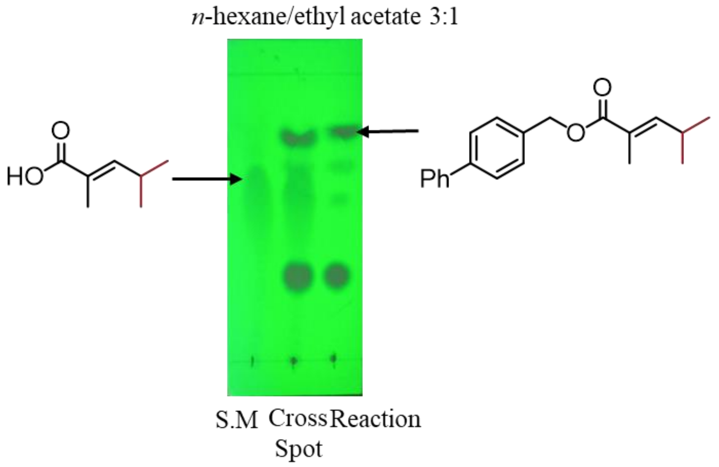

[1,1'-Biphenyl]-4-ylmethyl (*E*)-4-ethyl-2-methylhex-2-enoate (1b)

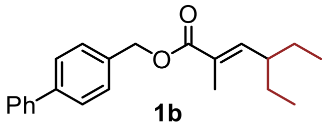

1b

According to General Procedure D, **S3b** (0.10 g, 0.6 mmol, 1.0 equiv), [1,1'-biphenyl]-4-ylmethanol (177 mg, 1.0 mmol, 1.5 equiv), DCC (158 mg, 0.8 mmol, 1.2 equiv), and DMAP (16 mg, 0.1 mmol, 0.2 equiv) were used to obtain the crude residue, which was purified by silica gel column chromatography (*n*-hexane/ethyl acetate 100:1, v/v), to afford **1b** as a white solid (120 mg, 0.3 mmol, 58%).

^{1}H NMR (400 MHz, $CDCl_3$) δ 7.62 – 7.59 (m, 4H), 7.48 – 7.43 (m, 4H), 7.38 – 7.33 (m, 1H), 6.59 (d, J = 13.9 Hz, 1H), 5.25 (s, 2H), 2.32 – 2.19 (m, 1H), 1.90 (s, 3H), 1.59 – 1.45 (m, 2H), 1.37 – 1.23 (m, 2H), 0.88 – 0.83 (m, 6H) ppm.

^{13}C{^{1}H} NMR (101 MHz, $CDCl_3$) δ 168.2, 147.8, 141.0, 140.7, 135.5, 128.8, 128.4, 127.6, 127.4, 127.2, 127.1, 65.9, 42.3, 27.6, 13.0, 11.9 ppm.

See NMR Spectra

HRMS (*m/z*): (ESI) calc'd for $C_{22}H_{26}O_2{}^{23}Na$ [M+Na]$^+$: 345.1825, found: 345.1825.

IR (ATR) ν_{max}: 2959, 2926, 2114, 1710, 1279, 1230, 1144, 1092, 760 and 697 cm^{-1}.

Melting point: 52 – 54 °C.

TLC: R_f = 0.20 (*n*-hexane/ethyl acetate 10:1, v/v)

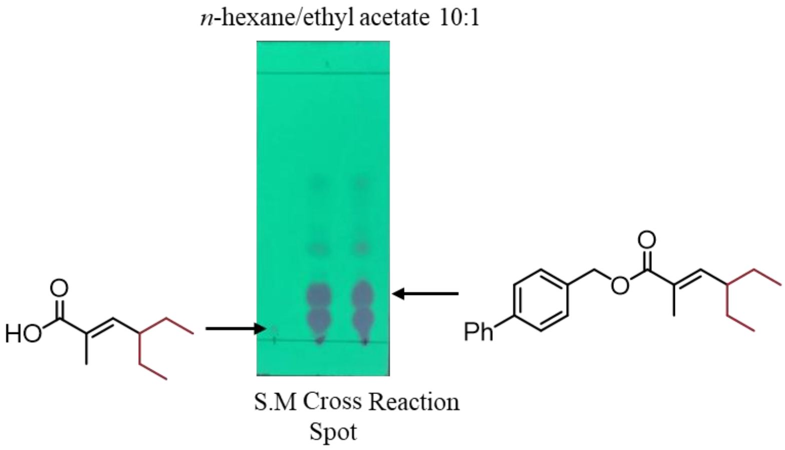

[1,1'-Biphenyl]-4-ylmethyl (*E*)-2-methylbut-2-enoate (1c)

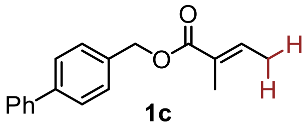

1c

According to General Procedure D, *trans*-2-methyl-2-butenoic acid (0.10 g, 1.0 mmol, 1.0 equiv), [1,1'-biphenyl]-4-ylmethanol (276 mg, 1.5 mmol, 1.5 equiv), DCC (247 mg, 1.2 mmol, 1.2 equiv), and DMAP (24 mg, 0.2 mmol, 0.2 equiv) were used to obtain the crude residue, which was purified by silica gel column chromatography (*n*-hexane/ethyl acetate 100:1, v/v), to afford **1c** as a colorless oil (141 mg, 0.5 mmol, 48%).

^{1}H NMR (400 MHz, $CDCl_3$) δ 7.64 – 7.62 (m, 4H), 7.50 – 7.45 (m, 4H), 7.40 – 7.36 (m, 1H), 6.99 (q, J = 7.0 Hz, 1H), 5.27 (s, 2H), 1.93 (s, 3H), 1.83 (d, J = 7.0 Hz, 3H) ppm.

^{13}C{^{1}H} NMR (101 MHz, $CDCl_3$) δ 167.8, 140.9, 140.6, 137.6, 135.4, 128.7, 128.5, 128.4, 127.3, 127.2, 127.0, 65.8, 14.3, 12.0 ppm.

See NMR Spectra

HRMS (*m/z*): (ESI) calc'd for $C_{18}H_{18}O_2{}^{23}Na$ $[M+Na]^+$: 289.1199, found: 289.1198.

IR (ATR) ν_{max}: 3030, 2924, 1706, 1651, 1488, 1254, 1130, 761 and 679 cm^{-1}.

TLC: R_f = 0.36 (*n*-hexane/ethyl acetate 10:1, v/v)

n-hexane/ethyl acetate 10:1

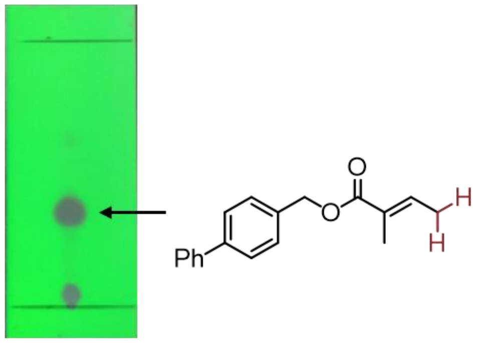

Reaction

[1,1'-Biphenyl]-4-ylmethyl (*E*)-3-cyclobutyl-2-methylacrylate (1d)

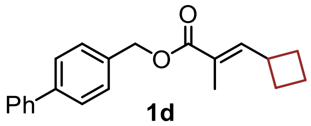

1d

According to General Procedure D, **S3d** (0.10 g, 0.7 mmol, 1.0 equiv), [1,1'-biphenyl]-4-ylmethanol (197 mg, 1.1 mmol, 1.5 equiv), DCC (177 mg, 0.9 mmol, 1.2 equiv), and DMAP (17 mg, 0.1 mmol, 0.2 equiv) were used to obtain the crude residue, which was purified by silica gel column chromatography (*n*-hexane/ethyl acetate 100:1, v/v), to afford **1d** as a white solid (140 mg, 0.4 mmol, 64%).

^{1}H NMR (300 MHz, $CDCl_3$) δ 7.62 – 7.59 (m, 4H), 7.48 – 7.33 (m, 5H), 6.95 (d, J = 8.5 Hz, 1H), 5.24 (s, 2H), 3.33 – 3.20 (m, 1H), 2.27 – 2.19 (m, 2H), 1.97 – 1.89 (m, 4H), 1.84 (s, 3H) ppm.

^{13}C{^{1}H} NMR (101 MHz, $CDCl_3$) δ 168.2, 147.5, 141.0, 140.7, 135.4, 128.8, 128.5, 127.4, 127.2, 127.1, 125.8, 65.9, 34.7, 28.8, 19.1, 12.5 ppm.

See NMR Spectra

HRMS (*m/z*): (ESI) calc'd for $C_{21}H_{22}O_2{}^{23}Na$ [M+Na]$^+$: 329.1512, found: 329.1512.

IR (ATR) ν_{max}: 2601, 2935, 2866, 1708, 1643, 1489, 1255, 1107, 1008 and 697 cm^{-1}.

Melting point: 68 – 75 °C.

TLC: R_f = 0.38 (*n*-hexane/ethyl acetate 10:1, v/v)

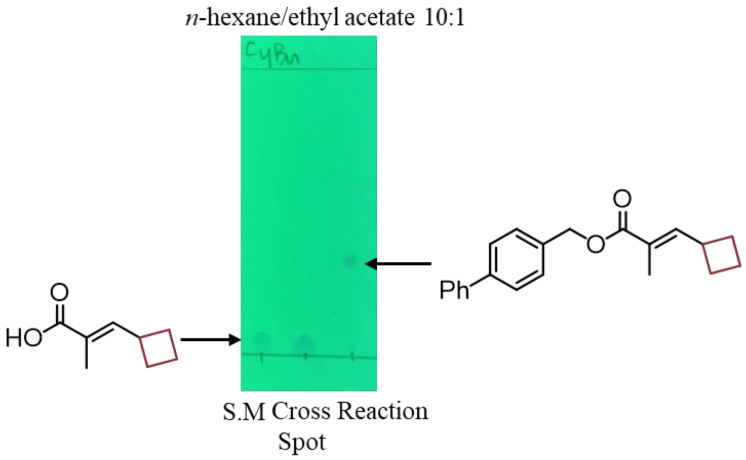

[1,1'-Biphenyl]-4-ylmethyl (*E*)-3-cyclopentyl-2-methylacrylate (1e)

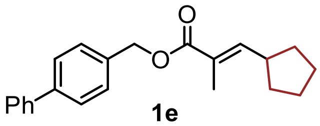

1e

According to General Procedure D, **S3e** (0.10 g, 0.6 mmol, 1.0 equiv), [1,1'-biphenyl]-4-ylmethanol (179 mg, 1.0 mmol, 1.5 equiv), DCC (161 mg, 0.8 mmol, 1.2 equiv), and DMAP (16 mg, 0.1 mmol, 0.2 equiv) were used to obtain the crude residue, which was purified by silica gel column chromatography (*n*-hexane/ethyl acetate 100:1, v/v), to afford **1e** as a white solid (110 mg, 0.3 mmol, 53%).

^{1}H NMR (400 MHz, $CDCl_3$) δ 7.62 – 7.60 (m, 4H), 7.48 – 7.43 (m, 4H), 7.38 – 7.34 (m, 1H), 6.79 (dd, J = 9.6, 0.8 Hz, 1H), 5.24 (s, 2H), 2.80 – 2.74 (m, 1H), 1.91 (d, J = 1.0 Hz, 3H), 1.88 – 1.83 (m, 2H), 1.75 – 1.69 (m, 2H), 1.67 – 1.60 (m, 2H), 1.39 – 1.32 (m, 2H) ppm.

^{13}C{^{1}H} NMR (101 MHz, $CDCl_3$) δ 168.3, 148.2, 141.0, 140.7, 135.5, 128.8, 128.5, 127.4, 127.2, 127.1, 126.0, 65.9, 39.4, 32.9, 25.5, 12.6 ppm.

See NMR Spectra

HRMS (*m/z*): (ESI) calc'd for $C_{22}H_{24}O_2{}^{23}Na$ [M+Na]$^+$: 343.1669, found: 343.1668.

IR (ATR) ν_{max}: 2950, 1866, 1706, 1645, 1488, 1239, 1101, 1008 and 760 cm^{-1}.

Melting point: 41 – 43 °C.

TLC: R_f = 0.31 (*n*-hexane/ethyl acetate 10:1, v/v)

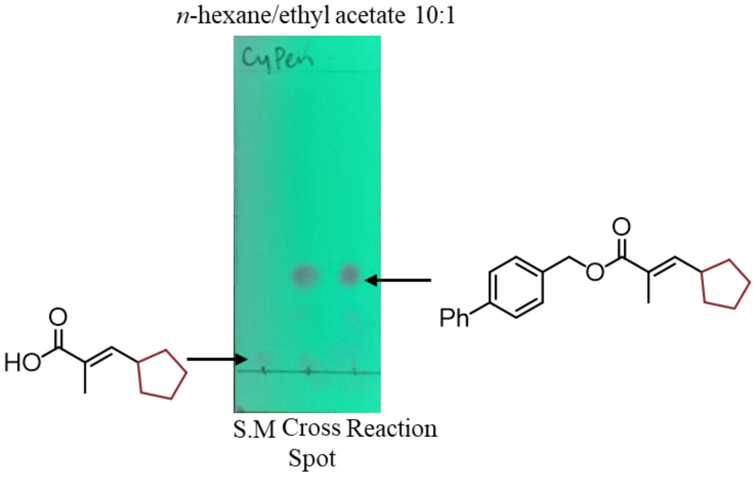

[1,1'-Biphenyl]-4-ylmethyl (*E*)-3-cyclohexyl-2-methylacrylate (1f)

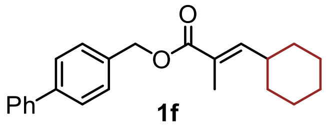

1f

According to General Procedure D, **S3f** (0.10 g, 0.6 mmol, 1.0 equiv), [1,1'-biphenyl]-4-ylmethanol (164 mg, 0.9 mmol, 1.5 equiv), DCC (147 mg, 0.7 mmol, 1.2 equiv), and DMAP (15 mg, 0.1 mmol, 0.2 equiv) were used to obtain the crude residue, which was purified by silica gel column chromatography (*n*-hexane/ethyl acetate 100:1, v/v), to afford **1f** as a white solid (105 mg, 0.3 mmol, 53%).

^{1}H NMR (400 MHz, $CDCl_3$) δ 7.61 – 7.59 (m, 4H), 7.47 – 7.42 (m, 4H), 7.38 – 7.33 (m, 1H), 6.67 (d, J = 9.6 Hz, 1H), 5.23 (s, 2H), 2.39 – 2.28 (m, 1H), 1.89 (s, 3H), 1.77 – 1.57 (m, 5H), 1.37 – 1.09 (m, 5H) ppm.

^{13}C{^{1}H} NMR (101 MHz, $CDCl_3$) δ 168.4, 148.1, 141.0, 140.7, 135.5, 128.8, 128.5, 127.4, 127.3, 127.1, 125.7, 65.9, 37.8, 31.9, 25.8, 25.6, 12.5 ppm.

See NMR Spectra

HRMS (*m/z*): (ESI) calc'd for $C_{23}H_{26}O_2{}^{23}Na$ [M+Na]$^+$: 357.1825, found: 357.1824.

IR (ATR) ν_{max}: 2957, 2923, 1710, 1648, 1304, 1243, 1150, 1086 and 760 cm^{-1}.

Melting point: 61 – 64 °C.

TLC: R_f = 0.43 (*n*-hexane/ethyl acetate 10:1, v/v)

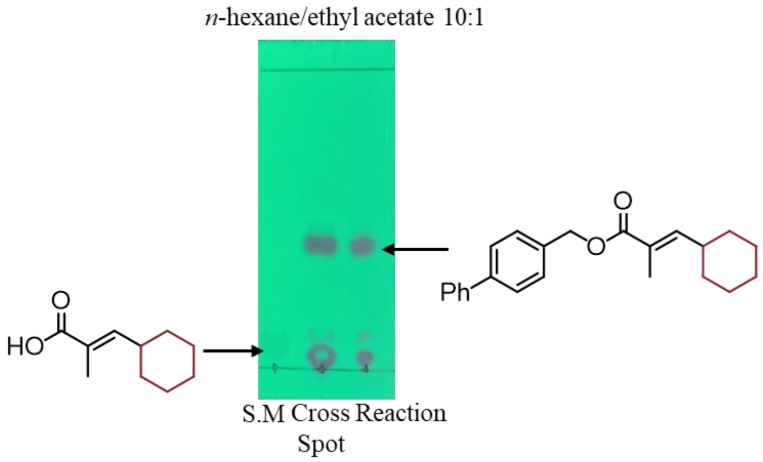

[1,1'-Biphenyl]-4-ylmethyl (*E*)-2-methyl-3-(tetrahydro-2H-pyran-4-yl)acrylate (1g)

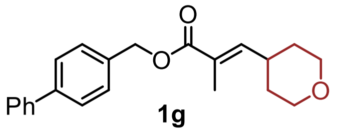

1g

According to General Procedure D, **S3g** (0.10 g, 0.6 mmol, 1.0 equiv), [1,1'-biphenyl]-4-ylmethanol (162 mg, 0.9 mmol, 1.5 equiv), DCC (145 mg, 0.7 mmol, 1.2 equiv), and DMAP (14 mg, 0.1 mmol, 0.2 equiv) were used to obtain the crude residue, which was purified by silica gel column chromatography (*n*-hexane/ethyl acetate 100:1, v/v), to afford **1g** as a white solid (110 mg, 0.3 mmol, 56%).

^{1}H NMR (600 MHz, $CDCl_3$) δ 7.61 – 7.59 (m, 4H), 7.47 – 7.44 (m, 4H), 7.37 – 7.35 (m, 1H), 6.65 (d, J = 9.4 Hz, 1H), 5.24 (s, 2H), 3.98 (dt, J = 11.5, 2.9 Hz, 2H), 3.46 (td, J = 11.5, 5.6 Hz, 2H), 2.63 – 2.57 (m, 1H), 1.92 (s, 3H), 1.57 – 1.54 (m, 4H) ppm.

^{13}C{^{1}H} NMR (151 MHz, $CDCl_3$) δ 168.1, 145.6, 141.1, 140.7, 135.3, 128.8, 128.6, 127.4, 127.3, 127.1, 126.9, 67.2, 66.1, 35.0, 31.4, 12.5 ppm.

See NMR Spectra

HRMS (*m/z*): (ESI) calc'd for $C_{22}H_{24}O_3{}^{23}Na$ [M+Na]$^+$: 359.1618, found: 359.1616.

IR (ATR) ν_{max}: 2952, 2841, 1731, 1708, 1488, 1276, 1226, 1125, 1093, 1008, 838, 762 and 698 cm^{-1}.

Melting point: 67 – 70 °C.

TLC: R_f = 0.17 (*n*-hexane/ethyl acetate 10:1, v/v)

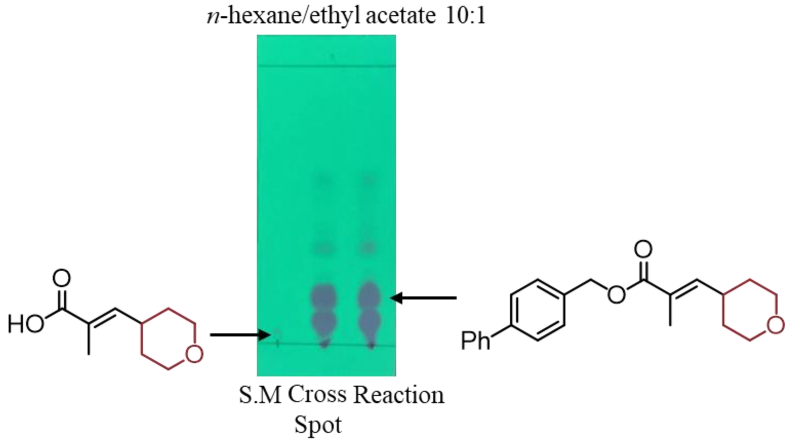

tert-Butyl (_E_)-4-(3-([1,1'-biphenyl]-4-ylmethoxy)-2-methyl-3-oxoprop-1-en-1-yl)piperidine-1-carboxylate (1h)

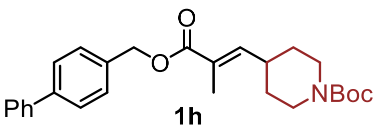

1h

According to General Procedure D, **S3h** (0.1 g, 0.4 mmol, 1.0 equiv), [1,1'-biphenyl]-4-ylmethanol (103 mg, 0.6 mmol, 1.5 equiv), DCC (92 mg, 0.4 mmol, 1.2 equiv), and DMAP (9 mg, 0.1 mmol, 0.2 equiv) were used to obtain the crude residue, which was purified by silica gel column chromatography (*n*-hexane/ethyl acetate 100:1, v/v), to afford **1h** as a white solid (110 mg, 0.3 mmol, 68%).

^{1}H NMR (600 MHz, $CDCl_3$) δ 7.60 – 7.58 (m, 4H), 7.46 – 7.43 (m, 4H), 7.37 – 7.34 (m, 1H), 6.64 – 6.62 (m, 1H), 5.23 (s, 2H), 4.10 (brs, 2H), 2.78 (t, J = 11.6 Hz, 2H), 2.52 – 2.45 (m, 1H), 1.92 – 1.91 (m, 3H), 1.62 – 1.60 (m, 2H), 1.46 (s, 9H), 1.39 – 1.33 (m, 2H) ppm.

^{13}C{^{1}H} NMR (151 MHz, $CDCl_3$) δ 168.0, 154.8, 145.4, 141.1, 140.7, 135.2, 128.8, 128.6, 127.4, 127.3, 127.1, 127.0, 79.5, 66.1, 43.3, 36.0, 30.7, 28.4, 12.6 ppm.

See NMR Spectra

HRMS (*m/z*): (ESI) calc'd for $C_{27}H_{33}O_4N^{23}Na$ $[M+Na]^+$: 458.2302, found: 458.2299.

IR (ATR) ν_{max}: 2930, 2851, 1691, 1650, 1422, 1365, 1246, 1148 and 762 cm^{-1}.

Melting point: 87 – 90 °C.

TLC: R_f = 0.44 (*n*-hexane/ethyl acetate 10:1, v/v)

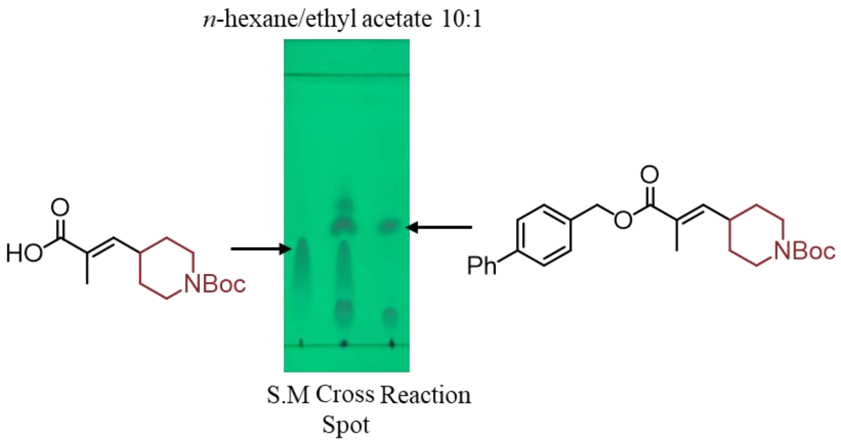

Benzyl (*E*)-4-(3-([1,1'-biphenyl]-4-ylmethoxy)-2-methyl-3-oxoprop-1-en-1-yl)piperidine-1-carboxylate (1i)

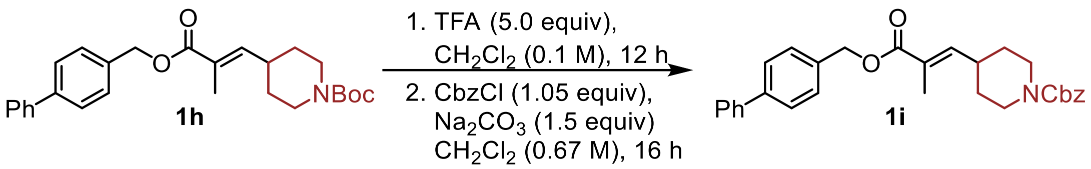

1h (0.10 g, 2.3 mmol, 1.0 equiv) was dissolved in CH_2Cl_2 (0.1 M) at room temperature. Then, the mixture solution was cooled to 0 ºC with an ice/water bath, and trifluoroacetic acid (1.30 g, 11.5 mmol, 5.0 equiv) was added continuously. After the reaction was cooled to 0 ºC for 10 min, it was allowed to stir at room temperature for 16 h. The reaction was cooled to 0 ºC and neutralization by 1N NaOH solution. Next, the solution was extracted by CH_2Cl_2 (30 mL x 3) and the organic layers were combined, dried over anhydrous Na_2SO_4, filtered and concentrated to give a crude reaction. Afterward, the filtrated solution was to afford the crude amine, which were used without purification.

The solution of crude amine (0.10 g, 3.0 mmol, 1.0 equiv) in CH_2Cl_2 (0.67 M), cooled to 0 °C with an ice/water bath, was added benzyl chloroformate (534 mg, 3.1 mmol, 1.05 equiv) and Na_2CO_3 (1.0 M in H_2O, 4.5 mmol, 1.5 equiv). The mixture was allowed to warm up to room temperature and stirred for 16 h. The organic layer was separated and the aqueous layer was extracted with CH_2Cl_2 (50 mL x 3). The combined organic layers were washed with sat. $NaHCO_3$ (100 mL) and brine (100 mL) and dried over anhydrous Na_2SO_4. After concentration under reduced pressure, the crude product was purified by silica gel column chromatography (*n*-hexane/ethyl acetate 100:1, v/v), to afford **1i** as a colorless oil (900 mg, 1.9 mmol, 64% for two steps).

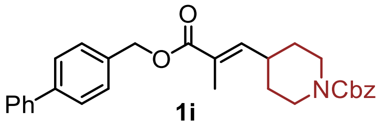

1i

<u>^{1}H NMR</u> (400 MHz, $CDCl_3$) δ 7.61 – 7.59 (m, 4H), 7.46 – 7.30 (m, 10H), 6.63 (d, J = 9.4 Hz, 1H), 5.23 (s, 2H), 5.14 (s, 2H), 4.20 (d, J = 10.3 Hz, 2H), 2.87 (t, J = 12.2 Hz, 2H), 2.57 – 2.47 (m, 1H), 1.92 (s, 3H), 1.64 (d, J = 9.0 Hz, 2H), 1.44 – 1.34 (m, 2H) ppm.

<u>$^{13}C\{^1H\}$ NMR</u> (101 MHz, $CDCl_3$) δ 167.9, 155.2, 145.1, 141.1, 140.7, 136.8, 135.2, 128.8, 128.6, 128.5, 128.0, 127.9, 127.4, 127.3, 127.2, 127.1, 67.1, 66.1, 43.5, 35.8, 30.7, 12.6 ppm.

See NMR Spectra

<u>HRMS (*m/z*)</u>: (ESI) calc'd for $C_{30}H_{31}O_4{}^{23}Na$ [M+Na]$^+$: 492.2145, found: 492.2142.

<u>IR (ATR) ν_{max}</u>: 2936, 2845, 1697, 1649, 1431, 1272, 1243, 1217 and 736 cm^{-1}.

<u>TLC</u>: R_f = 0.75 (*n*-hexane/ethyl acetate 3:1, v/v)

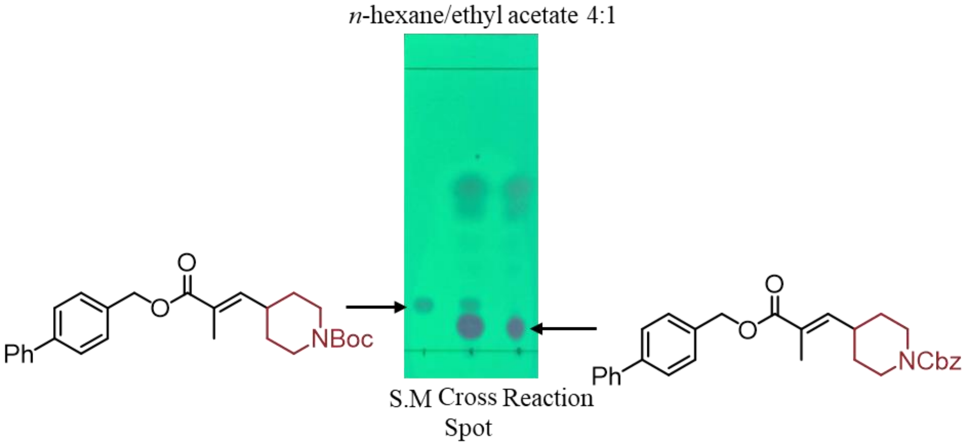

Benzyl (*E*)-2-methyl-4-phenylpent-2-enoate (1k)

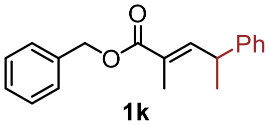

1k

According to General Procedure D, **S3k** (0.10 g, 0.5 mmol, 1.0 equiv), benzyl alcohol (85 mg, 0.8 mmol, 1.5 equiv), DCC (130 mg, 0.6 mmol, 1.2 equiv), and DMAP (13 mg, 0.1 mmol, 0.2 equiv) were used to obtain the crude residue, which was purified by silica gel column chromatography (*n*-hexane/ethyl acetate 100:1, v/v), to afford **1k** as a yellow oil (105 mg, 0.4 mmol, 71%).

^{1}H NMR (600 MHz, $CDCl_3$) δ 7.39 – 7.32 (m, 7H), 7.25 – 7.22 (m, 3H), 6.96 (d, J = 9.8 Hz, 1H), 5.20 (ABq, J = 12.6 Hz, 2H), 3.85 – 3.80 (m, 1H), 1.97 (s, 3H), 1.42 (d, J = 7.0 Hz, 3H) ppm.

^{13}C{^{1}H} NMR (151 MHz, $CDCl_3$) δ 168.0, 146.5, 144.4, 136.3, 128.6, 128.5, 128.0, 128.0, 127.0, 126.4, 126.4, 66.3, 38.7, 21.2, 12.6 ppm.

See NMR Spectra

HRMS (*m/z*): (ESI) calc'd for $C_{19}H_{20}O_2{}^{23}Na$ [M+Na]$^+$: 303.1356, found: 303.1355.

IR (ATR) ν_{max}: 3029, 2964, 2927, 1708, 1453, 1235, 1020, 744 and 698 cm^{-1}.

TLC: R_f = 0.52 (*n*-hexane/ethyl acetate 10:1, v/v)

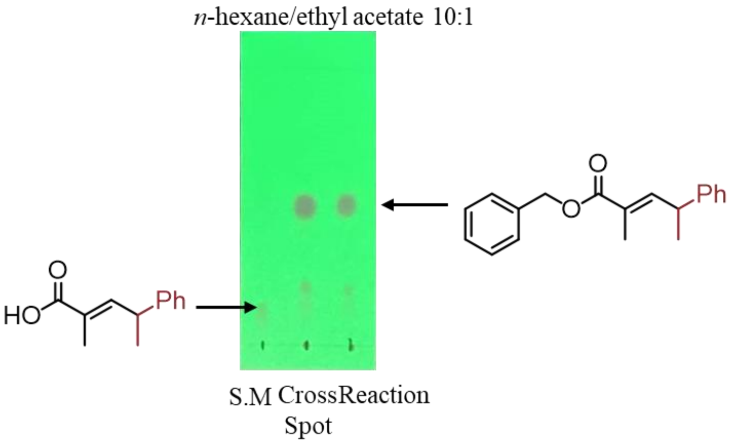

Back to contents / Back to substrate scope table

Benzyl (*E*)-2-methyl-4-phenylhex-2-enoate (1l)

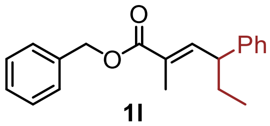

1l

According to General Procedure D, **S3l** (0.10 g, 0.5 mmol, 1.0 equiv), benzyl alcohol (79 mg, 0.8 mmol, 1.5 equiv), DCC (121 mg, 0.6 mmol, 1.2 equiv), and DMAP (12 mg, 0.1 mmol, 0.2 equiv) were used to obtain the crude residue, which was purified by silica gel column chromatography (*n*-hexane/ethyl acetate 100:1, v/v), to afford **1l** as a yellow oil (102 mg, 0.4 mmol, 71%).

^{1}H NMR (600 MHz, $CDCl_3$) δ 7.38 – 7.29 (m, 7H), 7.22 – 7.20 (m, 3H), 6.95 (dd, J = 10.1, 1.3 Hz, 1H), 5.18 (ABq, J = 9.4 Hz, 2H), 3.53 – 3.49 (m, 1H), 1.93 (d, J = 1.2 Hz, 3H), 1.84 – 1.73 (m, 2H), 0.88 (t, J = 7.4 Hz, 3H) ppm.

^{13}C{^{1}H} NMR (151 MHz, $CDCl_3$) δ 168.0, 145.7, 143.3, 136.3, 128.6, 128.5, 128.0, 127.5, 127.2, 126.4, 66.3 46.7, 29.3, 12.8, 12.0 ppm.

See NMR Spectra

HRMS (*m/z*): (ESI) calc'd for $C_{20}H_{22}O_2{}^{23}Na$ $[M+Na]^+$: 317.1512, found: 317.1512.

IR (ATR) ν_{max}: 2965, 2930, 1732, 1455, 1161, 752 and 699 cm^{-1}.

TLC: R_f = 0.60 (*n*-hexane/ethyl acetate 10:1, v/v)

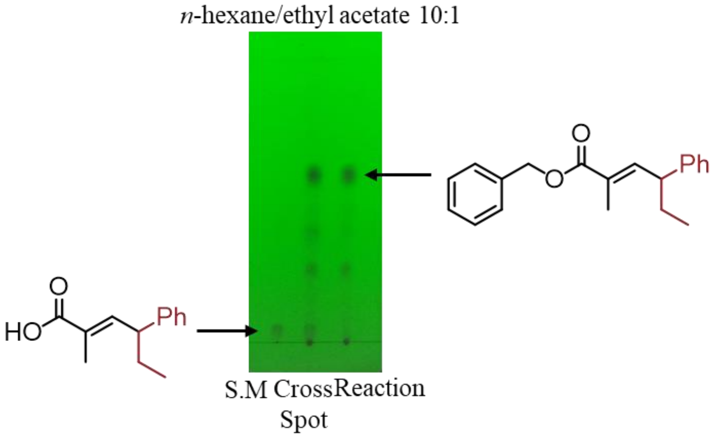

Benzyl (*E*)-2,5-dimethyl-4-phenylhex-2-enoate (1m)

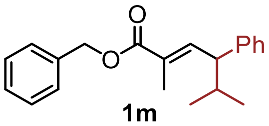

1m

According to General Procedure D, **S3m** (0.10 g, 0.5 mmol, 1.0 equiv), benzyl alcohol (74 mg, 0.7 mmol, 1.5 equiv), DCC (113 mg, 0.6 mmol, 1.2 equiv), and DMAP (11 mg, 0.1 mmol, 0.2 equiv) were used to obtain the crude residue, which was purified by silica gel column chromatography (*n*-hexane/ethyl acetate 100:1, v/v), to afford **1m** as a yellow oil (107 mg, 0.4 mmol, 76%).

^{1}H NMR (600 MHz, $CDCl_3$) δ 7.38 – 7.28 (m, 7H), 7.21 – 7.17 (m, 3H), 7.05 (dd, J = 10.4, 0.8 Hz, 1H), 5.18 (ABq, J = 11.0 Hz, 2H), 3.23 (t, J = 4.9 Hz, 1H), 2.05 – 1.99 (m, 1H), 1.90 (d, J = 0.8 Hz, 3H), 0.95 (d, J = 6.6 Hz, 3H), 0.78 (t, J = 6.6 Hz, 3H) ppm.

^{13}C{^{1}H} NMR (151 MHz, $CDCl_3$) δ 168.0, 145.1, 142.7, 136.4, 128.5, 128.0, 127.9, 127.3, 126.4, 66.3, 52.8, 33.7, 20.8, 12.8 ppm.

See NMR Spectra

HRMS (*m/z*): (ESI) calc'd for $C_{21}H_{24}O_2{}^{23}Na$ $[M+Na]^+$: 331.1669, found: 331.1668.

IR (ATR) ν_{max}: 2955, 2921, 2850, 1711, 1454, 1275, 1288, 1094 and 744 cm^{-1}.

TLC: R_f = 0.56 (*n*-hexane/ethyl acetate 10:1, v/v)

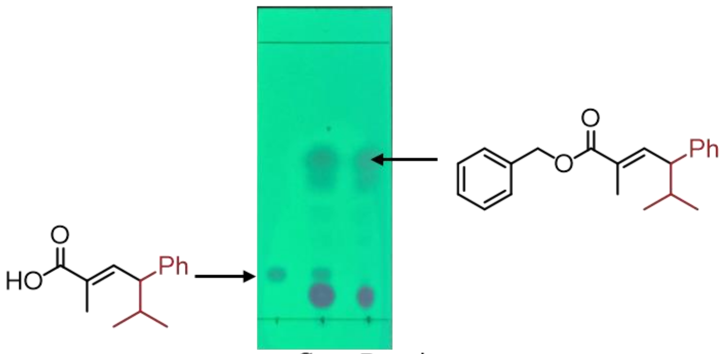

Benzyl (*E*)-2-methyl-4-(*p*-tolyl)pent-2-enoate (1n)

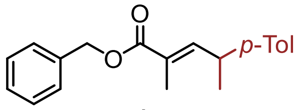

1n

According to General Procedure D, **S3n** (0.10 g, 0.5 mmol, 1.0 equiv), benzyl alcohol (79 mg, 0.7 mmol, 1.5 equiv), DCC (121 mg, 0.6 mmol, 1.2 equiv), and DMAP (12 mg, 0.1 mmol, 0.2 equiv) were used to obtain the crude residue, which was purified by silica gel column chromatography (*n*-hexane/ethyl acetate 100:1, v/v), to afford **1n** as a yellow oil (108 mg, 0.4 mmol, 75%).

^{1}H NMR (600 MHz, $CDCl_3$) δ 7.37 – 7.31 (m, 5H), 7.13 (s, 4H), 6.91 (dd, J = 9.9, 1.3 Hz, 1H), 5.18 (ABq, J = 10.8 Hz, 2H), 3.80 – 3.75 (m, 1H), 2.32 (s, 3H), 1.94 (d, J = 1.2 Hz, 3H), 1.38 (d, J = 6.7 Hz, 3H) ppm.

^{13}C{^{1}H} NMR (151 MHz, $CDCl_3$) δ 168.1, 146.8, 141.4, 136.3, 136.0, 129.3, 128.5, 128.1, 128.0, 126.8, 126.2, 66.3, 38.3, 21.3, 21.0, 12.6 ppm.

See NMR Spectra

HRMS (*m/z*): (ESI) calc'd for $C_{20}H_{22}O_2{}^{23}Na$ [M+Na]$^+$: 317.1512, found: 317.1513.

IR (ATR) ν_{max}: 2962, 2924, 1709, 1454, 1234, 1017, 755 and 697 cm^{-1}.

TLC: R_f = 0.38 (*n*-hexane/ethyl acetate 10:1, v/v)

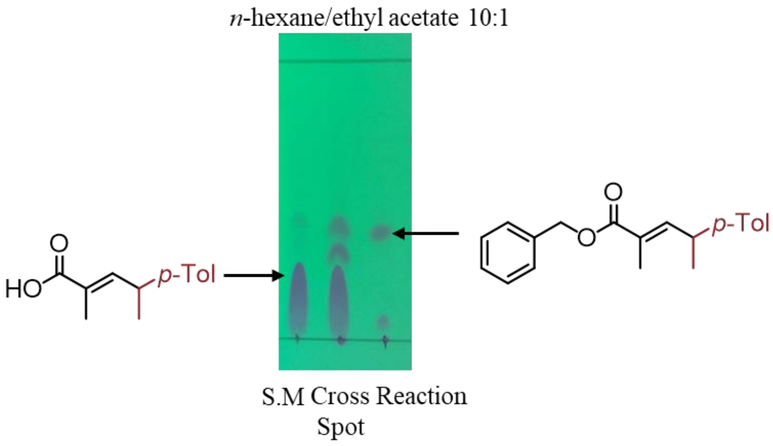

[1,1'-Biphenyl]-4-ylmethyl (*E*)-2-methylpent-2-enoate (1n')

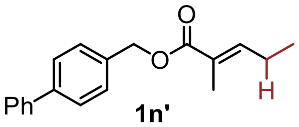

1n'

According to General Procedure D, *trans*-2-methyl-2-pentenoic acid (0.30 g, 2.6 mmol, 1.0 equiv), [1,1'-biphenyl]-4-ylmethanol (726 mg, 3.9 mmol, 1.5 equiv), DCC (651 mg, 3.2 mmol, 1.2 equiv), and DMAP (64 mg, 0.5 mmol, 0.2 equiv) were used to obtain the crude residue, which was purified by silica gel column chromatography (*n*-hexane/ethyl acetate 100:1, v/v), to afford **1n'** as a colorless oil (449 mg, 1.6 mmol, 62%).

^{1}H NMR (600 MHz, $CDCl_3$) δ 7.63 – 7.61 (m, 4H), 7.49 – 7.45 (m, 4H), 7.39 – 7.36 (m, 1H), 6.89 – 6.86 (m, 1H), 5.26 (s, 2H), 2.23 (p, J = 7.5 Hz, 2H), 1.91 (s, 3H), 1.08 (t, J = 7.6 Hz, 3H) ppm.

^{13}C{^{1}H} NMR (151 MHz, $CDCl_3$) δ 168.0, 144.4, 140.9, 140.7, 135.4, 128.7, 128.5, 127.3, 127.2, 127.04, 126.95, 65.9, 22.0, 12.9, 12.2 ppm.

See NMR Spectra

HRMS (*m/z*): (ESI) calc'd for $C_{19}H_{20}O_2{}^{23}Na$ [M+Na]$^+$: 303.1356, found: 303.1354.

IR (ATR) ν_{max}: 3322, 2929, 2850, 1706, 1488, 1241, 1138, 1087, 760 and 697 cm^{-1}.

TLC: R_f = 0.82 (*n*-hexane/ethyl acetate 6:1, v/v)

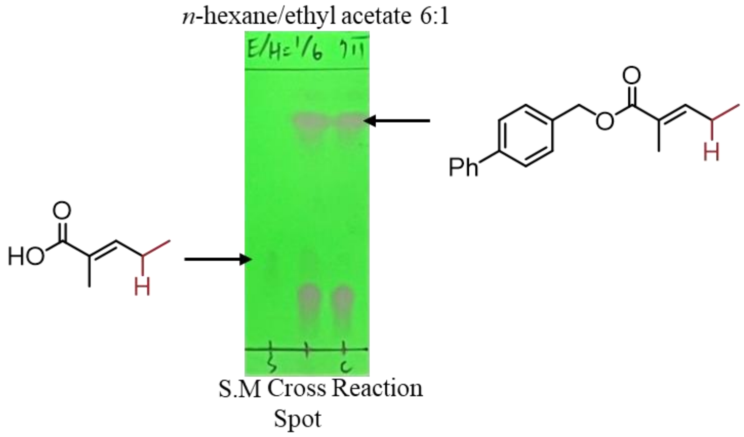

Benzyl (*E*)-2-methyl-4,4-diphenylbut-2-enoate (1o)

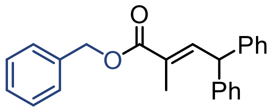

1o

According to General Procedure D, **S3o** (284 mg, 1.1 mmol, 1.0 equiv) and benzyl alcohol (243 mg, 2.3 mmol, 2.0 equiv) were used to obtain the residue, which was purified by silica gel column chromatography (*n*-hexane/ethyl acetate 100:1, v/v), to afford **1o** as a colorless oil (201 mg, 0.6 mmol, 53%).

^{1}H NMR (400 MHz, $CDCl_3$) δ 7.38 – 7.34 (m, 5H), 7.29 – 7.26 (m, 4H), 7.22 – 7.18 (m, 2H), 7.15 – 7.14 (m, 4H), 6.40 (dd, *J* = 10.5, 1.2 Hz, 1H), 5.81 (d, *J* = 10.5 Hz, 1H), 5.20 (s, 2H), 2.02 (d, *J* = 0.8 Hz, 3H) ppm.

^{13}C{^{1}H} NMR (101 MHz, $CDCl_3$) δ 167.7, 143.7, 142.9, 128.5, 128.3, 127.4, 126.4, 60.4, 49.2, 21.0, 14.2 ppm.

See NMR Spectra

HRMS (*m/z*): (EI) calc'd for $C_{24}H_{22}O_2$ $[M]^+$: 342.1614, found :342.1617.

IR (ATR) ν_{max}: 3031, 1713, 1492, 1215, 911, 742 and 697 cm^{-1}.

TLC: R_f = 0.74 (*n*-hexane/ethyl acetate 4:1, v/v)

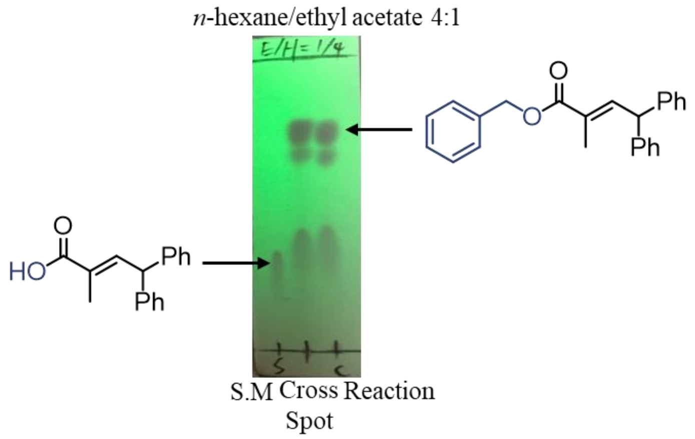

4-Fluorobenzyl (*E*)-2-methyl-4,4-diphenylbut-2-enoate (1p)

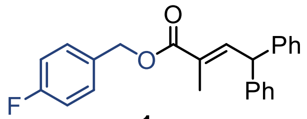

1p

According to General Procedure D, **S3o** (200 mg, 0.8 mmol, 1.0 equiv) and 4-fluoro-benzyl alcohol (201 mg, 1.6 mmol, 2.0 equiv) were used to obtain the residue, which was purified by silica gel column chromatography (*n*-hexane/ethyl acetate 100:1, v/v), to afford **1p** as a colorless oil (91 mg, 0.3 mmol, 32%).

^{1}H NMR (300 MHz, $CDCl_3$) δ 7.28 – 7.14 (m, 12H), 7.05 – 7.00 (m, 2H), 6.40 (d, J = 10.4 Hz, 1H), 5.80 (d, J = 10.5 Hz, 1H), 5.16 (s, 2H), 2.02 (s, 3H) ppm.

^{13}C{^{1}H} NMR (101 MHz, $CDCl_3$) δ 167.4, 162.6 (d, J = 246.7 Hz), 143.64, 143.59, 131.8 (d, J = 3.2 Hz), 130.1 (d, J = 8.3 Hz), 128.5, 128.3, 127.0, 126.4, 115.4 (d, J = 21.6 Hz), 65.5, 49.3, 21.0 ppm.

^{19}F NMR (282 MHz, $CDCl_3$) δ -113.71 ppm.

See NMR Spectra

HRMS (*m/z*): (EI) calc'd for $C_{24}H_{21}O_2F$ $[M]^+$: 360.1520, found 360.1519.

IR (ATR) ν_{max}: 3029, 1711, 1512, 1208, 1128, 825, 741 and 698 cm^{-1}.

TLC: R_f = 0.51 (*n*-hexane/ethyl acetate 10:1, v/v)

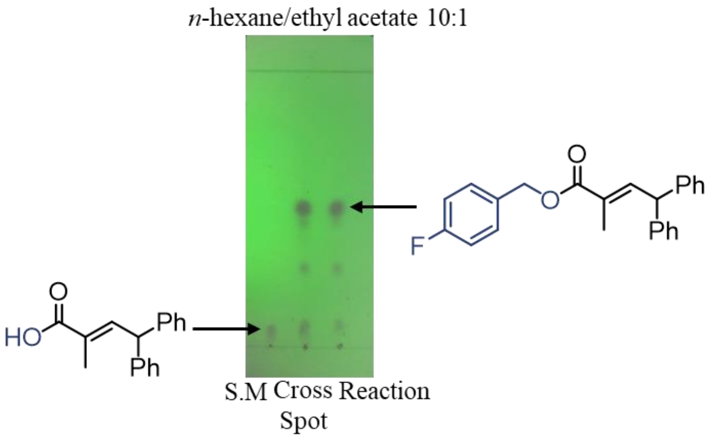

4-(Trifluoromethyl)benzyl (*E*)-2-methyl-4,4-diphenylbut-2-enoate (1q)

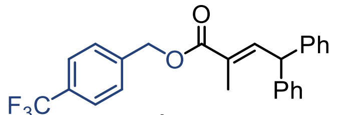

1q

According to General Procedure D, **S3o** (200 mg, 0.8 mmol, 1.0 equiv) and 4-(trifluoromethyl)-benzyl alcohol (1.2 mL, 1.6 mmol, 2.0 equiv) were used to obtain the residue, which was purified by silica gel column chromatography (*n*-hexane/ethyl acetate 100:1, v/v), to afford **1q** as a colorless oil (229 mg, 0.6 mmol, 70%).

^{1}H NMR (300 MHz, $CDCl_3$) δ 7.66 (d, J = 8.0 Hz, 2H), 7.47 (d, J = 7.9 Hz, 2H), 7.38 – 7.24 (m, 10H), 6.53 (d, J = 10.3 Hz, 1H), 5.91 (d, J = 10.4 Hz, 1H), 5.31 (s, 2H), 2.13 (s, 3H) ppm.

^{13}C{^{1}H} NMR (101 MHz, $CDCl_3$) δ 167.2, 144.2, 143.5, 139.9, 128.5, 128.3, 128.0, 126.8, 126.5, 125.53, 125.50, 125.46, 65.2, 49.3, 20.9 ppm.

Note: *The coupling of C and F was overlapped with aromatic carbons and could be interpreted.*

^{19}F NMR (282 MHz, $CDCl_3$) δ -62.57 ppm.

See NMR Spectra

HRMS (*m/z*): (EI) calc'd for $C_{25}H_{21}O_2F_3$ $[M]^+$: 410.1488, found 410.1485.

IR (ATR) ν_{max}: 3027, 1711, 1323, 1211, 1125, 828, 740 and 520 cm^{-1}.

TLC: R_f = 0.59 (*n*-hexane/ethyl acetate 10:1, v/v)

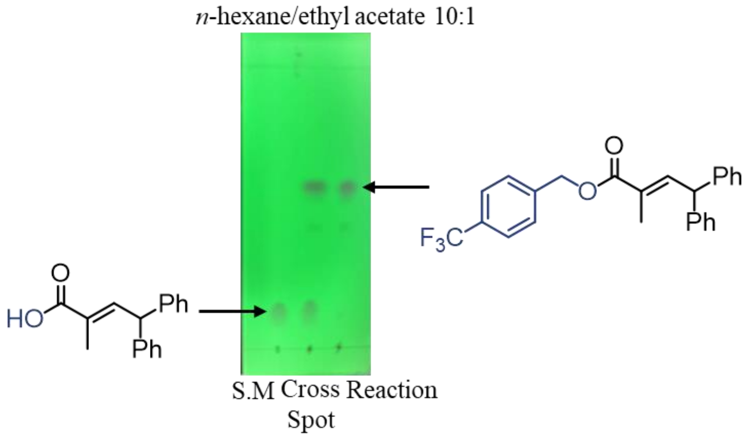

4-Methylbenzyl (*E*)-2-methyl-4,4-diphenylbut-2-enoate (1r)

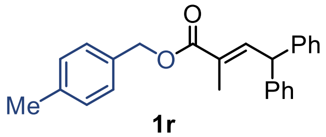

1r

According to General Procedure D, **S3o** (200 mg, 0.8 mmol, 1.0 equiv) and 4-methylbenzyl alcohol (147 mg, 1.2 mmol, 1.5 equiv) were used to obtain the residue, which was purified by silica gel column chromatography (*n*-hexane/ethyl acetate 100:1, v/v), to afford **1r** as a colorless oil (153 mg, 0.4 mmol, 54%).

^{1}H NMR (400 MHz, $CDCl_3$) δ 7.36 – 7.21 (m, 14H), 6.45 (d, J = 10.5 Hz, 1H), 5.90 (d, J = 10.5 Hz, 1H), 5.23 (s, 2H), 2.42 (s, 3H), 2.08 (s, 3H) ppm.

^{13}C{^{1}H} NMR (101 MHz, $CDCl_3$) δ 167.5, 143.6, 143.3, 137.9, 132.9, 129.2, 128.4, 128.3, 128.2, 127.1, 126.3, 66.1, 49.1, 21.1, 21.0 ppm.

See NMR Spectra

HRMS (*m/z*): (ESI) calc'd for $C_{25}H_{24}O_2{}^{23}Na$ [M+Na]$^+$: 379.1667, found: 379.1667.

IR (ATR) ν_{max}: 3027, 1712, 1453, 1210, 1125, 740 and 698 cm^{-1}.

TLC: R_f = 0.61 (*n*-hexane/ethyl acetate 10:1, v/v)

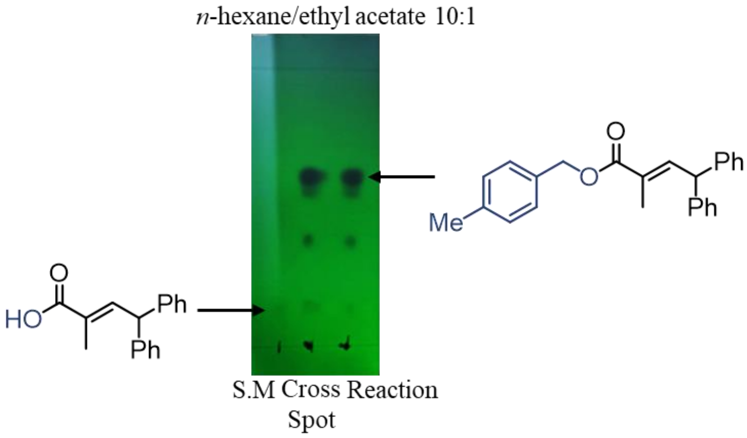

4-Methoxybenzyl (*E*)-2-methyl-4,4-diphenylbut-2-enoate (1s)

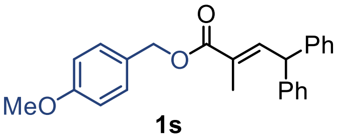

1s

According to General Procedure D, **S3o** (100 mg, 0.4 mmol, 1.0 equiv) and 4-methoxy-benzyl alcohol (110 mg, 0.8 mmol, 2.0 equiv) were used to obtain the residue, which was purified by silica gel column chromatography (*n*-hexane/ethyl acetate 100:1, v/v), to afford **1s** as a colorless oil (55 mg, 0.1 mmol, 37%).

^{1}H NMR (300 MHz, $CDCl_3$) δ 7.29 – 7.13 (m, 12H), 6.88 (d, J = 8.4 Hz, 2H), 6.38 (d, J = 10.5 Hz, 1H), 5.80 (d, J = 10.5 Hz, 1H), 5.14 (s, 2H), 3.82 (s, 3H), 2.01 (s, 3H) ppm.

$^{13}C\{^1H\}$ NMR (101 MHz, $CDCl_3$) δ 167.6, 159.6, 143.7, 143.2, 130.0, 128.5, 128.3, 128.1, 127.2, 126.4, 113.9, 66.0, 55.3, 49.2, 21.0 ppm.

See NMR Spectra

HRMS (*m/z*): (ESI) calc'd for $C_{25}H_{24}O_3{}^{23}Na$ $[M+Na]^+$: 395.1618, found 395.1617.

IR (ATR) ν_{max}: 2970, 1733, 1510, 1226, 830, 762, 702 and 490 cm^{-1}.

TLC: R_f = 0.50 (*n*-hexane/ethyl acetate 10:1, v/v)

n-hexane/ethyl acetate 10:1

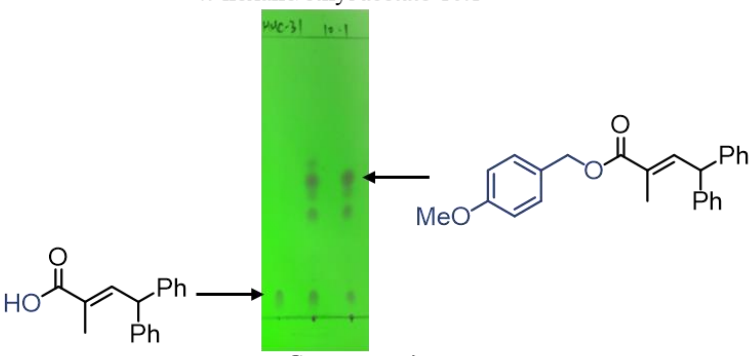

S.M Cross Reaction
Spot

3-Methoxybenzyl (*E*)-2-methyl-4,4-diphenylbut-2-enoate (1t)

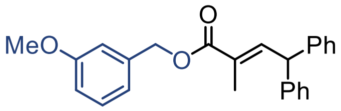

1t

According to General Procedure D, **S3o** (100 mg, 0.4 mmol, 1.0 equiv) and 3-methoxy-benzyl alcohol (110 mg, 0.8 mmol, 2.0 equiv) were used to obtain the residue, which was purified by silica gel column chromatography (*n*-hexane/ethyl acetate 100:1, v/v), to afford **1t** as a colorless oil (49 mg, 0.1 mmol, 33%).

^{1}H NMR (300 MHz, $CDCl_3$) δ 7.31 – 7.15 (m, 11H), 6.94 – 6.87 (m, 3H), 6.41 (d, J = 10.5 Hz, 1H), 5.84 (d, J = 10.5 Hz, 1H), 5.19 (s, 2H), 3.78 (s, 3H), 2.04 (s, 3H) ppm.

^{13}C{^{1}H} NMR (101 MHz, $CDCl_3$) δ 167.7, 159.7, 143.60, 143.58, 137.5, 129.6, 128.5, 128.3, 127.0, 126.4, 120.3, 113.8, 113.4, 66.1, 55.2, 49.2, 21.0 ppm.

See NMR Spectra

HRMS (*m/z*): (ESI) calc'd for $C_{25}H_{24}O_3{}^{23}Na$ [M+Na]$^+$: 395.1618, found 395.1616.

IR (ATR) ν_{max}: 2931, 1732, 1491, 1265, 1159, 763 and 697 cm^{-1}.

TLC: R_f = 0.48 (*n*-hexane/ethyl acetate 10:1, v/v)

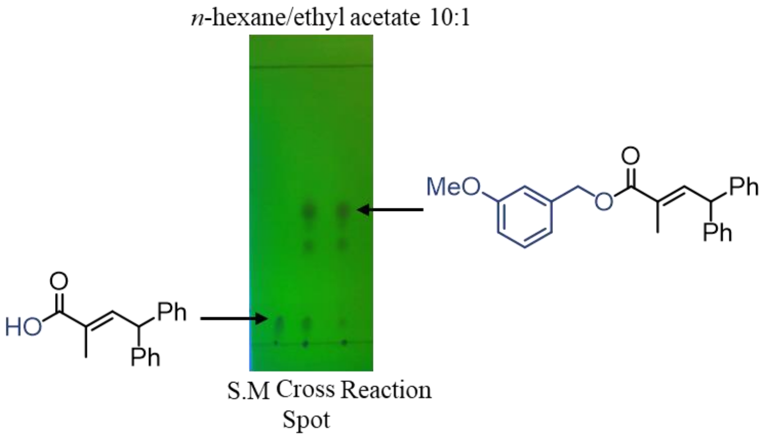

Methyl (*E*)-2-methyl-4,4-diphenylbut-2-enoate (1u)

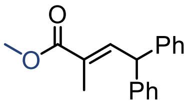

1u

S3o (1.10 g, 4.1 mmol, 1.0 equiv) was added sulfuric acid (0.1 mL) in MeOH (0.1 M) and reflux for 22 h. The reaction was extracted with ethyl acetate (10 mL x 3). The combined organic layers was dried over anhydrous sodium sulphate, filtered and concentrated under reduced pressure to afford **1u** as a yellow oil (931 mg, 3.5 mmol, 85%).

^{1}H NMR (400 MHz, $CDCl_3$) δ 7.36 – 7.32 (m, 4H), 7.27 – 7.25 (m, 6H), 6.44 (d, *J* = 9.4 Hz, 1H), 5.91 (d, *J* = 10.2 Hz, 1H), 3.79 (s, 3H), 2.05 (s, 3H) ppm.

^{13}C{^{1}H} NMR (101 MHz, $CDCl_3$) δ 168.0, 143.7, 143.5, 128.5, 128.3, 127.0, 126.4, 51.4, 49.2, 20.9 ppm.

See NMR Spectra

HRMS (*m/z*): (ESI) calc'd for $C_{18}H_{18}O_2{}^{23}Na$ $[M+Na]^+$: 289.1199 , found 289.1199.

IR (ATR) ν_{max}: 3060, 1714, 1598, 1449, 1218, 1129 and 699 cm^{-1}.

TLC: R_f = 0.57 (*n*-hexane/ethyl acetate 10:1, v/v)

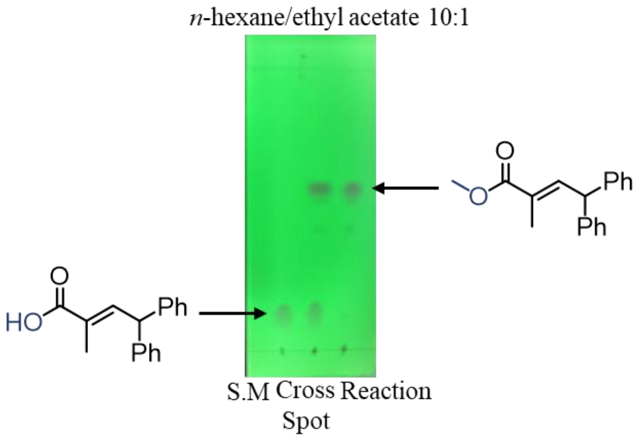

Back to contents / Back to substrate scope table

Isopropyl (*E*)-2-methyl-4,4-diphenylbut-2-enoate (1w)

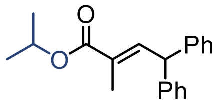

1w

S3o (200 mg, 0.8 mmol, 1.0 equiv) was added sulfuric acid (0.1 mL) in ^{i}PrOH (0.1 M) and reflux for 22 h. The crude reaction was purified by silica gel column chromatography (*n*-hexane/ethyl acetate 100:1, v/v), to afford **1w** as a pale-yellow oil (68 mg, 0.2 mmol, 29%).

^{1}H NMR (300 MHz, $CDCl_3$) δ 7.32 – 7.27 (m, 4H), 7.22 – 7.19 (m, 6H), 6.32 (d, J = 10.4 Hz, 1H), 5.82 (d, J = 10.4 Hz, 1H), 5.15 – 5.03 (m, 1H), 1.98 (s, 3H), 1.26 (d, J = 6.2 Hz, 6H) ppm.

^{13}C{^{1}H} NMR (101 MHz, $CDCl_3$) δ 167.4, 143.8, 142.1, 128.5, 128.3, 127.9, 126.3, 67.9, 49.2, 21.9, 21.0 ppm.

See NMR Spectra

HRMS (*m/z*): (ESI) calc'd for $C_{20}H_{22}O_2{}^{23}Na$ $[M+Na]^+$: 317.1512, found 317.1512.

IR (ATR) ν_{max}: 2990, 2922, 1716, 1616, 1296, 703 and 498 cm^{-1}.

TLC: R_f = 0.83 (*n*-hexane/ethyl acetate 6:1, v/v)

n-hexane/ethyl acetate 6:1

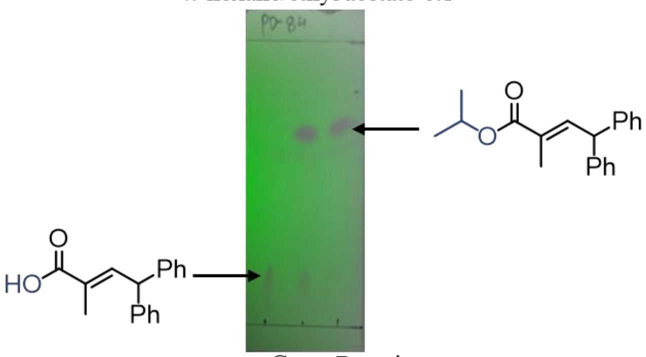

S.M Cross Reaction
Spot

tert-Butyl (_E_)-2-methyl-4,4-diphenylbut-2-enoate (1x)

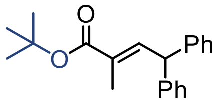

1x

S3o (1.0 g, 3.9 mmol, 1.0 equiv) was added sulfuric acid (0.1 mL) in ^{t}BuOH (0.1 M) and reflux for 22 h. The crude reaction was purified by silica gel column chromatography (*n*-hexane/ethyl acetate 100:1, v/v), to afford **1x** as a pale-yellow oil (28 mg, 0.1 mmol, 2%).

^{1}H NMR (300 MHz, $CDCl_3$) δ 7.32 – 7.27 (m, 4H), 7.21 – 7.18 (m, 6H), 6.21 (d, J = 10.4 Hz, 1H), 5.75 (d, J = 10.4 Hz, 1H), 1.95 (s, 3H), 1.47 (s, 9H) ppm.

^{13}C{^{1}H} NMR (101 MHz, $CDCl_3$) δ 167.4, 143.9, 140.4, 129.2, 128.5, 128.3, 126.3, 80.9, 49.2, 28.2, 21.2 ppm.

See NMR Spectra

HRMS (*m/z*): (EI) calc'd for $C_{21}H_{24}O_2$ $[M]^+$: 308.1771, found 308.1770.

IR (ATR) ν_{max}: 2981, 2930, 1714, 1378, 1121, 699 and 501 cm^{-1}.

TLC: R_f = 0.85 (*n*-hexane/ethyl acetate 10:1, v/v)

n-hexane/ethyl acetate 10:1

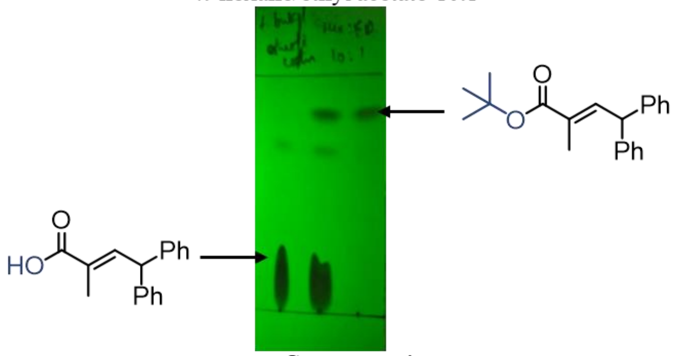

S.M Cross Reaction
Spot

(1*R*,3*R*,5*R*,7*R*)-Adamantan-2-yl (*E*)-2-methyl-4,4-diphenylbut-2-enoate (1y)

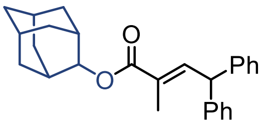

1y

According to General Procedure D, **S3o** (300 mg, 1.2 mmol, 1.0 equiv) and (1*r*,3*r*,5*r*,7*r*)-adamantan-2-ol (190 mg, 1.2 mmol, 1.5 equiv) were used to obtain the residue, which was purified by silica gel column chromatography (*n*-hexane/ethyl acetate 100:1, v/v), to afford **1y** (114 mg, 0.3 mmol, 25%) as a colorless oil.

^{1}H NMR (400 MHz, $CDCl_3$) δ 7.31 – 7.27 (m, 4H), 7.22 – 7.18 (m, 6H), 6.35 (d, J = 10.5 Hz, 1H), 5.95 (d, J = 10.5 Hz, 1H), 5.03 (s, 1H), 2.04 – 1.94 (m, 8H), 1.87 – 1.73 (m, 9H) ppm.

^{13}C{^{1}H} NMR (101 MHz, $CDCl_3$) δ 167.1, 143.8, 142.7, 128.5, 128.4, 127.9, 126.3, 49.1, 37.4, 36.3, 31.9, 27.2, 27.0, 21.2 ppm.

See NMR Spectra

HRMS (*m/z*): (ESI) calc'd for $C_{27}H_{30}O_2{}^{23}Na$ [M+Na]$^+$: 409.2138, found :409.2137.

IR (ATR) ν_{max}: 2922, 2846, 1704, 1452, 1362, 1236 and 695 cm^{-1}.

TLC: R_f = 0.87 (*n*-hexane/ethyl acetate 6:1, v/v)

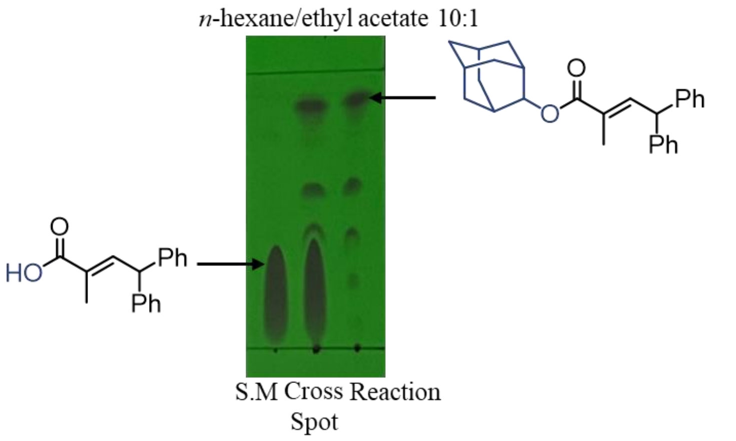

Naphthalen-2-ylmethyl (*E*)-2-methyl-4,4-diphenylbut-2-enoate (1z)

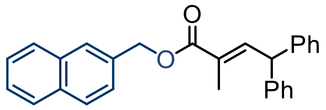

1z

According to General Procedure D, **S3o** (200 mg, 0.8 mmol, 1.0 equiv) and naphthalen-2-ylmethanol (190 mg, 1.2 mmol, 1.5 equiv) were used to obtain the residue, which was purified by silica gel column chromatography (*n*-hexane/ethyl acetate 100:1, v/v), to afford **1z** as a colorless oil (189 mg, 0.5 mmol, 61%).

^{1}H NMR (400 MHz, $CDCl_3$) δ 7.87 – 7.83 (m, 4H), 7.54 – 7.48 (m, 3H), 7.31 – 7.18 (m, 10H), 6.46 (d, J = 10.5 Hz, 1H), 5.90 (d, J = 10.5 Hz, 1H), 5.41 (s, 2H), 2.09 (s, 3H) ppm.

^{13}C{^{1}H} NMR (101 MHz, $CDCl_3$) δ 167.5, 143.6, 143.5, 133.3, 133.1, 133.0, 128.5, 128.32, 128.28, 128.0, 127.7, 127.2, 127.1, 126.4, 126.23, 126.20, 125.8, 66.3, 49.2, 21.0 ppm.

See NMR Spectra

HRMS (*m/z*): (ESI) calc'd for $C_{28}H_{24}O_2{}^{23}Na$ $[M+Na]^+$: 415.1669, found: 415.1669.

IR (ATR) ν_{max}: 3027, 2922, 1710, 1210, 1124, 701 and 474 cm^{-1}.

TLC: R_f = 0.62 (*n*-hexane/ethyl acetate 5:1, v/v)

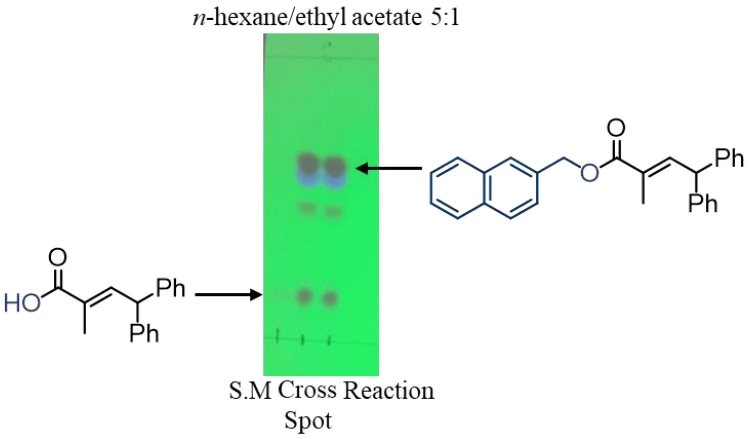

Back to contents / Back to substrate scope table

Benzyl (*E*)-2-ethyl-4,4-diphenylbut-2-enoate (1aa)

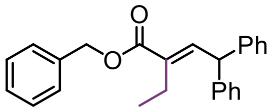

1aa

According to General Procedure D, **S3p** (196 mg, 0.7 mmol, 1.0 equiv) and benzyl alcohol (159 mg, 1.5 mmol, 2.0 equiv) were used to obtain the residue, which was purified by silica gel column chromatography (*n*-hexane/ethyl acetate 100:1, v/v), to afford **1aa** as a colorless oil (260 mg, 0.7 mmol, 99%).

^{1}H NMR (500 MHz, $CDCl_3$) δ 7.35 – 7.34 (m, 5H), 7.29 – 7.27 (m, 4H), 7.22 – 7.19 (m, 2H), 7.14 – 7.13 (m, 4H), 6.30 (d, J = 10.5 Hz, 1H), 5.67 (d, J = 10.5 Hz, 1H), 5.21 (s, 2H), 2.40 (q, J = 7.2 Hz, 2H), 1.09 (t, J = 7.4 Hz, 3H) ppm.

^{13}C{^{1}H} NMR (101 MHz, $CDCl_3$) δ 167.7, 143.7, 140.9, 135.9, 133.6, 128.52, 128.46, 128.3, 128.22, 128.15, 126.3, 66.2, 49.2, 27.7, 13.5 ppm.

See NMR Spectra

HRMS (*m/z*): (EI) calc'd for $C_{25}H_{24}O_2$ $[M]^+$: 356,1771, found: 356.1770.

IR (ATR) ν_{max}: 2961, 1712, 1211, 1127, 739, 696 and 497 cm^{-1}.

TLC: R_f = 0.65 (*n*-hexane/ethyl acetate 2:1, v/v)

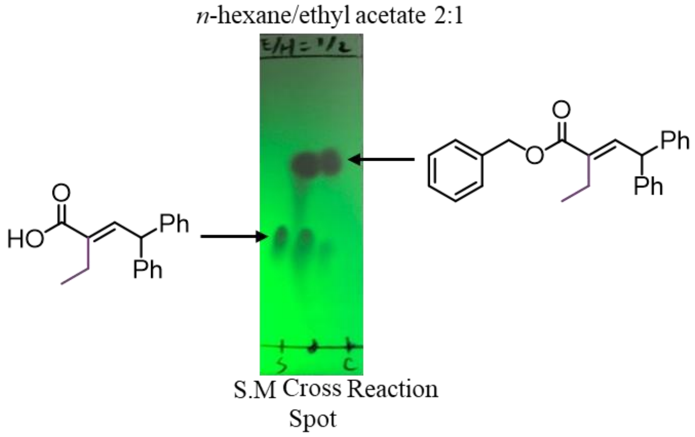

Benzyl (*E*)-2-*n*-propyl-4,4-diphenylbut-2-enoate (1ab)

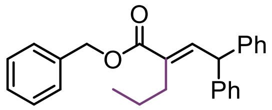

1ab

According to General Procedure D, **S3q** (240 mg, 0.9 mmol, 1.0 equiv) and benzyl alcohol (191 mg, 1.8 mmol, 2.0 equiv) were used to obtain the residue, which was purified by silica gel column chromatography (*n*-hexane/ethyl acetate 100:1, v/v), to afford **1ab** as a colorless oil (215 mg, 0.6 mmol, 68%).

^{1}H NMR (400 MHz, $CDCl_3$) δ 7.34 – 7.34 (m, 5H), 7.30 – 7.28 (m, 4H), 7.22 – 7.19 (m, 2H), 7.15 – 7.13 (m, 4H), 6.31 (d, J = 10.5 Hz, 1H), 5.66 (d, J = 10.5 Hz, 1H), 5.21 (s, 2H), 2.35 (t, J = 7.4 Hz, 2H), 1.55 – 1.46 (m, 2H), 0.91 (t, J = 7.3 Hz, 3H) ppm.

^{13}C{^{1}H} NMR (101 MHz, $CDCl_3$) δ 167.7, 143.7, 141.8, 135.9, 132.1, 128.5, 128.4, 128.3, 128.2, 128.1, 126.3, 66.2, 49.3, 36.8, 22.2, 13.6 ppm.

See NMR Spectra

HRMS (*m/z*): (ESI) calc'd for $C_{26}H_{26}O_2{}^{23}Na$ [M+Na]$^+$: 393.1825, found 393.1824.

IR (ATR) ν_{max}: 2961, 1714, 1492, 1451, 1205, 1133, 739 and 699 cm^{-1}.

TLC: R_f = 0.65 (*n*-hexane/ethyl acetate 4:1, v/v)

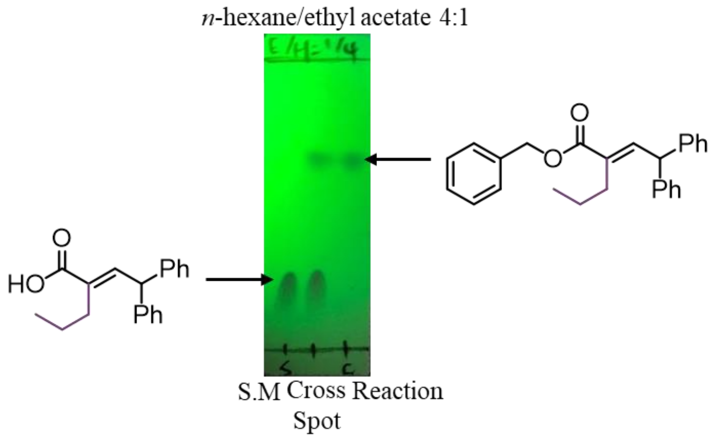

Benzyl (*E*)-2-(2,2-diphenylethylidene)hexanoate (1ac)

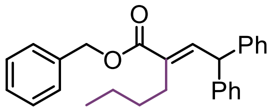

1ac

According to General Procedure D, **S3r** (200 mg, 0.7 mmol, 1.0 equiv) and benzyl alcohol (151 mg, 1.4 mmol, 2.0 equiv) were used to obtain the residue, which was purified by silica gel column chromatography (*n*-hexane/ethyl acetate 100:1, v/v), to afford **1ac** as a colorless oil (205 mg, 0.5 mmol, 79%).

^{1}H NMR (400 MHz, $CDCl_3$) δ 7.36 – 7.34 (m, 5H), 7.30 – 7.28 (m, 4H), 7.22 – 7.19 (m, 2H), 7.15 – 7.13 (m, 4H), 6.30 (d, J = 5.2 Hz, 1H), 5.65 (d, J = 5.2 Hz, 1H), 5.21 (s, 2H), 2.37 (t, J = 7.4 Hz, 2H), 1.47 – 1.42 (m, 2H), 1.35 – 1.30 (m, 2H), 0.89 (t, J = 7.0 Hz, 3H) ppm.

^{13}C{^{1}H} NMR (101 MHz, $CDCl_3$) δ 167.7, 143.7, 141.5, 135.9, 132.3, 128.5, 128.4, 128.3, 128.2, 128.1, 126.3, 66.2, 49.3, 34.5, 31.1, 22.3, 13.8 ppm.

See NMR Spectra

HRMS (*m/z*): (EI) calc'd for $C_{27}H_{28}O_2$ $[M]^+$: 384.2084, found: 384.2083.

IR (ATR) ν_{max}: 2942, 1671, 1443, 1248, 1179, 699 and 474 cm^{-1}.

TLC: R_f = 0.65 (*n*-hexane/ethyl acetate 4:1, v/v)

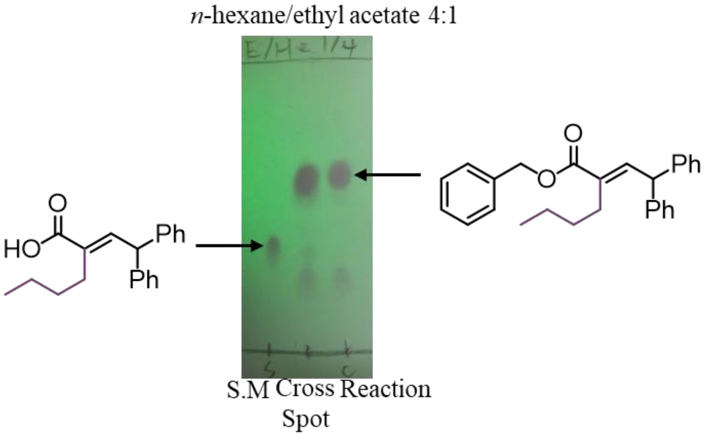

Benzyl (*E*)-2-(2,2-diphenylethylidene)pent-4-enoate (1ad)

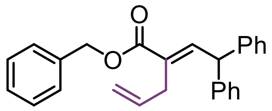

1ad

According to General Procedure D, **S3s** (433 mg, 1.6 mmol, 1.0 equiv) and benzyl alcohol (332 mg, 3.1 mmol, 2.0 equiv) were used to obtain the residue, which was purified by silica gel column chromatography (*n*-hexane/ethyl acetate 100:1, v/v), to afford **1ad** as a colorless oil (349 mg, 1.0 mmol, 61%).

^{1}H NMR (300 MHz, $CDCl_3$) δ 7.33 – 7.30 (m, 7H), 7.25 – 7.11 (m, 8H), 6.37 (d, J = 10.5 Hz, 1H), 5.91 – 5.80 (m, 1H), 5.75 (d, J = 10.5 Hz, 1H), 5.19 (s, 2H), 5.08 (d, J = 7.0 Hz, 1H), 5.03 (s, 1H), 3.12 (d, J = 6.4 Hz, 2H) ppm.

^{13}C{^{1}H} NMR (101 MHz, $CDCl_3$) δ 168.1, 143.7, 143.5, 143.2, 142.8, 128.7, 128.5, 128.3, 128.2, 127.0, 126.7, 126.4, 51.4, 49.2, 21.0 ppm.

See NMR Spectra

HRMS (*m/z*): (ESI) calc'd for $C_{26}H_{24}O_2{}^{23}Na$ $[M+Na]^+$: 391.1669, found : 391.1669.

IR (ATR) ν_{max}: 2925,1733, 1443, 1153, 919, 755 and 696 cm^{-1}.

TLC: R_f = 0.65 (*n*-hexane/ethyl acetate 6:1, v/v)

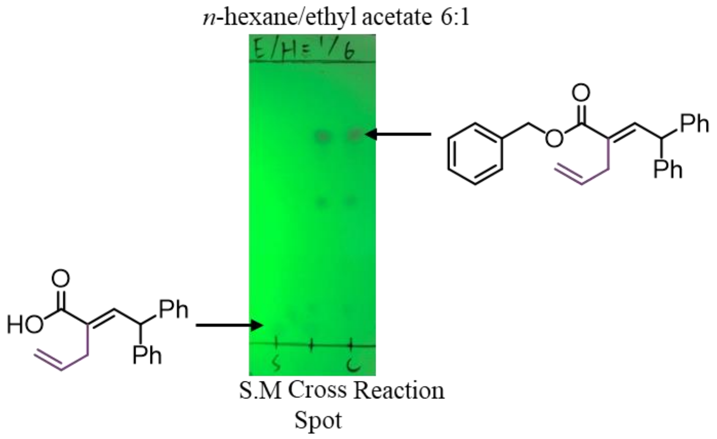

Benzyl (*E*)-2-(2,2-diphenylethylidene)hex-5-enoate (1ae)

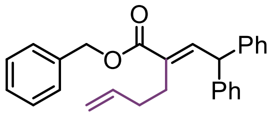

1ae

According to General Procedure D, **S3t** (270 mg, 0.9 mmol, 1.0 equiv) and benzyl alcohol (150 mg, 1.4 mmol, 1.5 equiv) were used to obtain the residue, which was purified by silica gel column chromatography (*n*-hexane/ethyl acetate 100:1, v/v), to afford **1ae** (114 mg, 0.3 mmol, 32%) as a colorless oil.

^{1}H NMR (400 MHz, $CDCl_3$) δ 7.33 – 7.28 (m, 6H), 7.25 – 7.20 (m, 5H), 7.13 – 7.12 (m, 4H), 6.32 (d, J = 10.3 Hz, 1H), 5.82 – 5.72 (m, 1H), 5.68 (d, J = 10.2 Hz, 1H), 5.20 (s, 2H), 4.96 (t, J = 12.7 Hz, 2H), 2.45 (t, J = 7.1 Hz, 2H), 2.23 (q, J = 7.5 Hz, 2H) ppm.

^{13}C{^{1}H} NMR (101 MHz, $CDCl_3$) δ 167.4, 143.6, 142.8, 137.6, 135.9, 131.3, 128.54, 128.46, 128.3, 128.23, 128.19, 126.4, 115.4, 66.3, 49.3, 34.2, 33.2 ppm.

See NMR Spectra

HRMS (*m/z*): (ESI) calc'd for $C_{27}H_{26}O_2{}^{23}Na$ $[M+Na]^+$: 405.1825, found 405.1823.

IR (ATR) ν_{max}: 3034, 2928, 1716, 1444, 1198, 1142 and 701 cm^{-1}.

TLC: R_f = 0.71 (*n*-hexane/ethyl acetate 10:1, v/v)

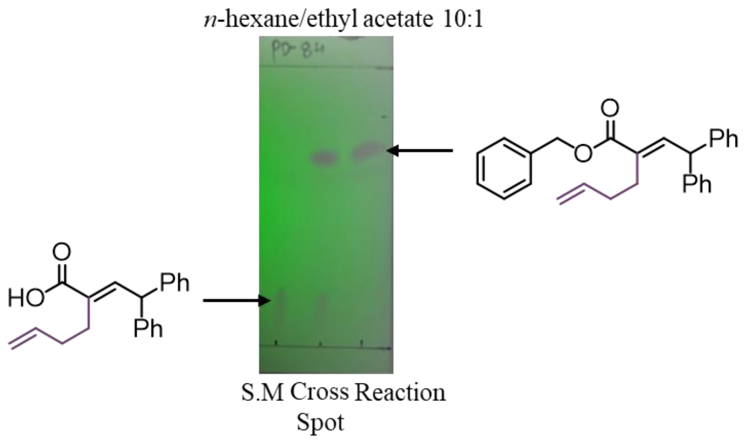

Methyl 4-methyl-2-phenylpent-2-enoate [1af + (*Z*)-1af]

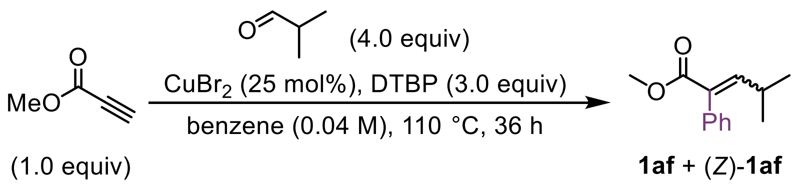

An oven-dried reaction seal tube was charged with $CuBr_2$ (266 mg, 1.2 mmol, 25 mol%), benzene (0.04 M), methyl propiolate (400 mg, 4.8 mmol, 1.0 equiv), 2-methylpropanal (1.37 g, 19.0 mmol, 4.0 equiv), and di-*tert*-butyl peroxide (DTBP, 2.09 g, 14.0 mmol, 3.0 equiv). Then, the reaction tube was sealed and stirred at 110 ºC for 26 h. After the reaction was finished, the resulting mixture was cooled to room temperature and concentrated to remove the solvent *in vacuo*. The residue was purified by chromatography and preparative TLC with a mixture of *n*-hexane/CH_2Cl_2 = 3:1 as an eluting system to provide the desired **1af** (146 mg, 0.7 mmol, 15%) and (*Z*)-**1af** (152 mg, 0.8 mmol, 16%).

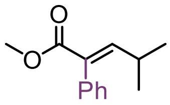

1af

^{1}H NMR (600 MHz, $CDCl_3$) δ 7.36 (t, *J* = 7.3 Hz, 2H), 7.32 (t, *J* = 7.3 Hz, 1H), 7.16 (d, *J* = 7.1 Hz, 2H), 6.86 (d, *J* = 10.6 Hz, 1H), 3.72 (s, 3H), 2.43 – 2.35 (m, 1H), 0.99 (d, *J* = 6.6 Hz, 6H) ppm.

^{13}C{^{1}H} NMR (151 MHz, $CDCl_3$) δ 168.0, 151.7, 135.6, 131.5, 129.5, 128.0, 127.4, 52.0, 28.5, 22.2 ppm.

See NMR Spectra

HRMS (*m/z*): (ESI) calc'd for $C_{13}H_{16}O_2{}^{23}Na$ $[M+Na]^+$: 227.1043, found 227.1041.

IR (ATR) ν_{max}: 2961, 2901, 1716, 1434, 1242, 1038, 770 and 705 cm^{-1}.

TLC: R_f = 0.25 (*n*-hexane/CH_2Cl_2 3:1, v/v)

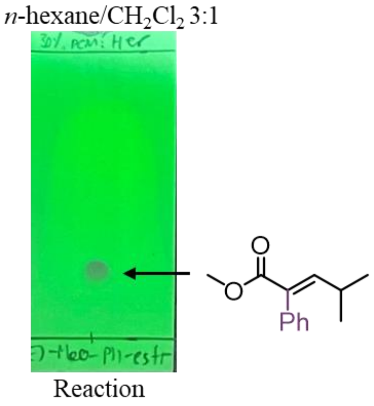

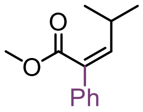

(*Z*)-**1af**

^{1}H NMR (600 MHz, $CDCl_3$) δ 7.34 – 7.27 (m, 5H), 5.97 (d, *J* = 10.0 Hz, 1H), 3.80 (s, 3H), 3.00 – 2.92 (m, 1H), 1.10 (d, *J* = 6.6 Hz, 6H) ppm.

^{13}C{^{1}H} NMR (151 MHz, $CDCl_3$) δ 168.8, 146.6, 137.8, 132.2, 128.3, 127.5, 127.1, 51.7, 29.4, 22.7 ppm.

See NMR Spectra

HRMS (*m/z*): (ESI) calc'd for $C_{13}H_{16}O_2{}^{23}Na$ $[M+Na]^+$: 227.1043, found 227.1041.

IR (ATR) ν_{max}: 2959, 2869, 1721, 1359, 1201, 1009, 760 and 697 cm^{-1}.

TLC: R_f = 0.28 (*n*-hexane/CH_2Cl_2 3:1, v/v)

n-hexane/CH_2Cl_2 3:1

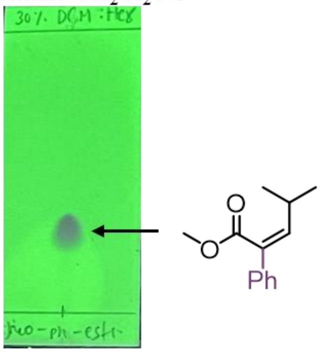

Reaction

Back to contents / Back to substrate scope table

[1,1'-Biphenyl]-4-ylmethyl 4-methyl-2-phenylpent-2-enoate [1ag + (*Z*)-1ag]

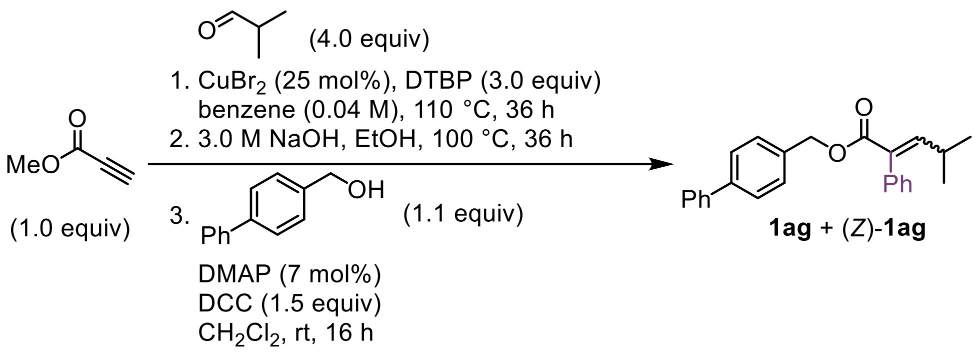

An oven-dried reaction seal tube was charged with $CuBr_2$ (332 mg, 1.5 mmol, 25 mol%), benzene (0.04 M), methyl propiolate (500 mg, 6.0 mmol, 1.0 equiv), 2-methylpropanal (1.72 g, 24.0 mmol, 4.0 equiv), and di-*tert*-butyl peroxide (DTBP, 2.61 g, 18.0 mmol, 3.0 equiv). Then, the reaction tube was sealed and stirred at 110 ºC for 36 h. After the reaction was finished, the resulting mixture was cooled to room temperature and concentrated to remove the solvent *in vacuo*. The obtained ester residue was further hydrolysed without purification.

A solution containing ester residue in EtOH (20 mL) was treated with 3.0 M NaOH (20 mL). Then, reaction flask was equipped with condenser, and the mixture solution was stirred at 100 ºC for 36 h. After the mixture esters were hydrolysed completely, the reaction flask was cooled at room temperature, and then Et_2O (30 mL) was added into the reaction solution to remove the impurity by extraction. The aqueous phase was next acidified by 10% HCl to pH 1. To obtain the acid product, the acidified aqueous layer was extracted with CH_2Cl_2 (20 mL x 3). The combined CH_2Cl_2 phase was dried over anhydrous Na_2SO_4 and concentrated *in vacuo* to provide the crude carboxylic acid, which was used in the next step without further purification.

A two-necked round bottom flask was charged with a crude acid, [1,1'-biphenyl]-4-ylmethanol (1.15 g, 6.2 mmol, 1.1 equiv), DMAP (51 mg, 0.4 mmol, 7 mol%), and dry DCM (0.3 M). Then, the mixture solution was cooled at 0 ºC, and a solution of DCC (1.84 g, 8.9 mmol, 1.5 equiv) in dry CH_2Cl_2 (10.0 mL) was added continuously. After the reaction was stirred at 0 ºC for 10 min, it was allowed to stir at room temperature for 16 h. The suspension was removed by filtration *via* celite, and the filtrated solution was concentrated *in vacuo*. Next, the residue was purified by column chromatography and preparative TLC with a mixture of *n*-hexane/CH_2Cl_2 = 3:1 as an eluting system to provide the desired **1ag** (318 mg, 0.9 mmol, 15%) and (*Z*)-**1ag** (446 mg, 1.26 mmol, 21%).

Back to contents / Back to substrate scope table

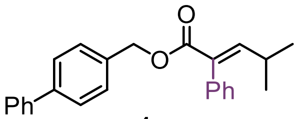

1ag

^{1}H NMR (600 MHz, $CDCl_3$) δ 7.59 – 7.56 (m, 4H), 7.44 (t, *J* = 7.7 Hz, 2H), 7.39 – 7.32 (m, 6H), 7.20 (d, *J* = 7.0 Hz, 2H), 6.91 (d, *J* = 10.6 Hz, 1H), 5.24 (s, 2H), 2.47 – 2.39 (m, 1H), 1.00 (d, *J* = 6.6 Hz, 6H) ppm.

^{13}C{^{1}H} NMR (151 MHz, $CDCl_3$) δ 167.3, 151.9, 140.9, 140.7, 135.6, 135.3, 131.6, 129.6, 128.8, 128.3, 128.0, 127.4, 127.2, 127.1, 66.1, 29.7, 28.6, 22.2 ppm.

See NMR Spectra

HRMS (*m/z*): (ESI) calc'd for $C_{25}H_{24}O_2{}^{23}Na$ $[M+Na]^+$: 379.1669, found 379.1666.

IR (ATR) ν_{max}: 2960, 2922, 1713, 1235, 1177, 1074, 761 and 702 cm^{-1}.

TLC: R_f = 0.13 (*n*-hexane/CH_2Cl_2 3:1, v/v)

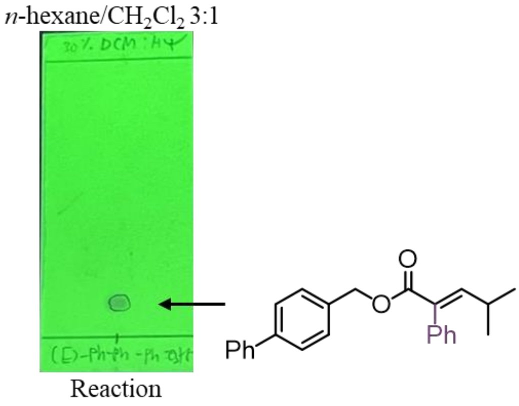

Back to contents / Back to substrate scope table

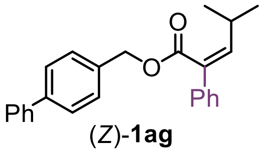

(*Z*)-**1ag**

^{1}H NMR (600 MHz, $CDCl_3$) δ 7.60 – 7.58 (m, 4H), 7.46 – 7.43 (m, 4H), 7.36 (t, *J* = 7.4 Hz, 1H), 7.32 – 7.27 (m, 5H), 5.99 (d, *J* = 10.1 Hz, 1H), 5.32 (s, 2H), 3.00 – 2.92 (m, 1H), 1.08 (d, *J* = 6.6 Hz, 6H) ppm.

^{13}C{^{1}H} NMR (151 MHz, $CDCl_3$) δ 168.2, 146.3, 141.1, 140.7, 137.6, 134.8, 132.2, 128.8, 128.7, 128.3, 127.5, 127.4, 127.2, 127.12, 127.10, 66.2, 29.4, 22.7 ppm.

See NMR Spectra

HRMS (*m/z*): (ESI) calc'd for $C_{25}H_{24}O_2{}^{23}Na$ $[M+Na]^+$: 379.1669, found 379.1666.

IR (ATR) ν_{max}: 2967, 2920, 1720, 1376, 1169, 1075, 759 and 696 cm^{-1}.

TLC: R_f = 0.23 (*n*-hexane/CH_2Cl_2, v/v)

n-hexane/CH_2Cl_2 3:1

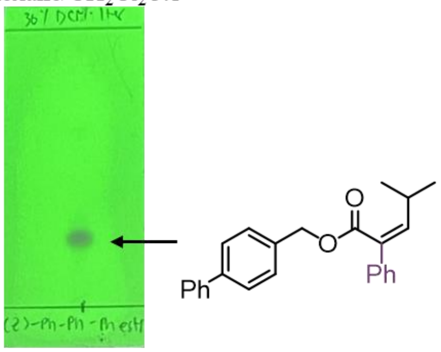

Reaction

[1,1'-Biphenyl]-4-ylmethyl 2-isopropyl-4-methylpent-2-enoate [1ah + (*Z*)-1ah]

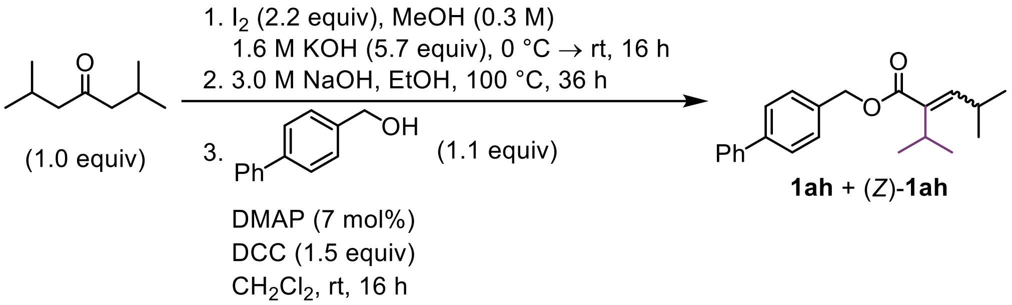

To a solution of I_2 (3.93 g, 16.0 mmol, 2.2 equiv) in MeOH (0.3 M), the starting ketone (1.00 g, 7.0 mmol, 1.0 equiv) was added slowly, and then the mixture solution was cooled at 5 ºC. Then, 1.6 M KOH (25 mL, 29.0 mmol, 5.7 equiv) in MeOH was then added into the reaction flask at 5 ºC over 25 min. Next, the solution was allowed to warm to room temperature for 16 h. After the reaction was finished, the solution was concentrated *in vacuo*; subsequently, the resulting suspension was diluted with DCM and filtrated. Afterward, the filtrated solution was concentration to afford the crude α,β-unsaturated esters, which were further submitted to hydrolysis step without purification.

A solution containing ester residue in EtOH (20 mL) was treated with 3.0 M NaOH (20 mL). Then, reaction flask was equipped with condenser, and the mixture solution was stirred at 100 ºC (heating metal temperature) for 36 h. After the mixture esters were hydrolyzed completely, the reaction flask was cooled at room temperature, and then Et_2O (30 mL) was added into the reaction solution to remove the impurity by extraction. The aqueous phase was next acidified by 10% HCl to pH 1. To obtain the acid product, the acidified aqueous layer was extracted with CH_2Cl_2 (20 mL x 3). The combined DCM phase was dried over anhydrous Na_2SO_4 and concentrated *in vacuo* to provide acid (95 mg, 0.6 mmol, 9% for two steps).

A two-necked round bottom flask was charged with acid (95 mg, 0.6 mmol, 1.0 equiv), [1,1'-biphenyl]-4-ylmethanol (118 mg, 0.6 mmol, 1.1 equiv), DMAP (5 mg, 0.04 mmol, 7 mol%), and dry CH_2Cl_2 (0.30 M). Then, the mixture solution was cooled at 0 ºC, and a solution of DCC (189 mg, 0.9 mmol, 1.5 equiv) in dry CH_2Cl_2 (3.0 mL) was added continuously. After the reaction was stirred at 0 ºC for 10 min, it was allowed to stir at room temperature for 16 h. The suspension was removed by filtration *via* celite, and the filtrated solution was concentrated *in vacuo*. Next, the residue was purified by preparative TLC with a mixture of *n*-hexane/CH_2Cl_2 = 3:1 as an eluting system to provide the product **1ah** (12 mg, 0.04 mmol, 6%) and (*Z*)-**1ah** (31 mg, 0.10 mmol, 16%).

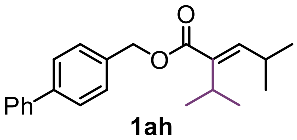

1ah

^{1}H NMR (600 MHz, $CDCl_3$) δ 7.60 (d, *J* = 7.5 Hz, 4H), 7.46 – 7.43 (m, 4H), 7.35 (t, *J* = 7.3 Hz, 1H), 6.46 (d, *J* = 9.9 Hz, 1H), 5.21 (s, 2H), 2.98 – 2.91 (m, 1H), 2.77 – 2.71 (m, 1H), 1.20 (d, *J* = 6.9 Hz, 6H), 1.02 (d, *J* = 6.5 Hz, 6H) ppm.

^{13}C{^{1}H} NMR (151 MHz, $CDCl_3$) δ 167.9, 148.4, 140.9, 140.7, 135.5, 128.8, 128.6, 127.4, 127.2, 127.1, 65.7, 30.9, 27.6, 27.4, 22.5, 21.3 ppm.

See NMR Spectra

HRMS (*m/z*): (ESI) calc'd for $C_{22}H_{26}O_2{}^{23}Na$ $[M+Na]^+$: 345.1825, found 345.1823.

IR (ATR) ν_{max}: 2986, 2902, 1737, 1373, 1236, 1045 and 847 cm^{-1}.

TLC: R_f = 0.30 (*n*-hexane/CH_2Cl_2 3:1, v/v)

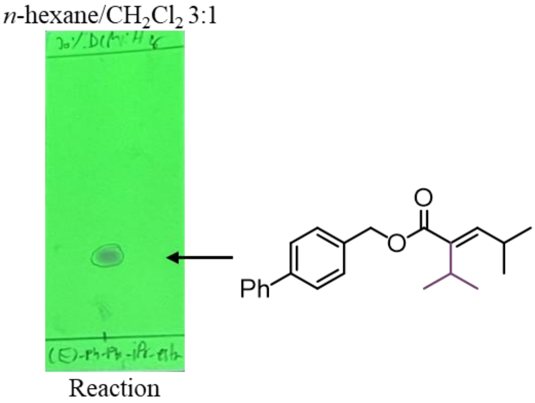

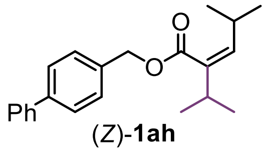

(*Z*)-**1ah**

^{1}H NMR (600 MHz, $CDCl_3$) δ 7.59 (d, *J* = 8.0 Hz, 4H), 7.46 – 7.43 (m, 4H), 7.35 (t, *J* = 7.4 Hz, 1H), 5.50 – 5.48 (m, 1H), 5.24 (s, 2H), 2.92 – 2.84 (m, 1H), 2.71 – 2.65 (m, 1H), 1.05 (d, *J* = 6.9 Hz, 6H), 0.97 (d, *J* = 6.6 Hz, 6H) ppm.

^{13}C{^{1}H} NMR (151 MHz, $CDCl_3$) δ 169.1, 142.3, 141.0, 140.7, 136.5, 135.2, 128.8, 128.6, 127.4, 127.2, 127.1, 65.8, 31.3, 28.5, 22.9, 21.8 ppm.

See NMR Spectra

HRMS (*m/z*): (ESI) calc'd for $C_{22}H_{26}O_2{}^{23}Na$ $[M+Na]^+$: 345.1825, found 345.1822.

IR (ATR) ν_{max}: 2967, 1716, 1394, 1222, 1066, 761 and 697 cm^{-1}.

TLC: R_f = 0.38 (*n*-hexane/CH_2Cl_2 3:1, v/v)

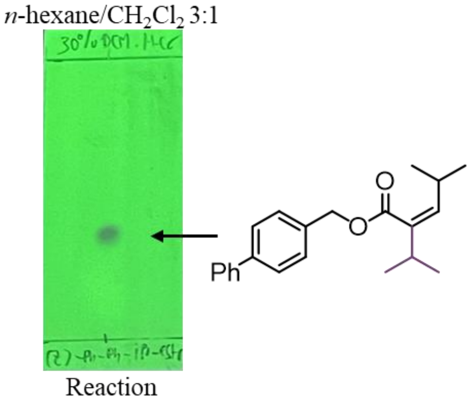

(1*R*,2*S*,5*R*)-2-Isopropyl-5-methylcyclohexyl (*E*)-2-methyl-4,4-diphenylbut-2-enoate (1ai)

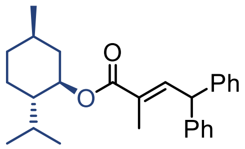

1ai

According to General Procedure D, **S3o** (0.10 g, 0.5 mmol, 1.0 equiv), (-)-menthol (110 mg, 0.7 mmol, 1.5 equiv), DCC (121 mg, 0.6 mmol, 1.2 equiv), and DMAP (12 mg, 0.1 mmol, 0.2 equiv) were used to obtain the crude residue, which was purified by silica gel column chromatography (*n*-hexane/ethyl acetate 100:1, v/v), to afford **1ai** as a white solid (117 mg, 0.3 mmol, 60%).

^{1}H NMR (600 MHz, $CDCl_3$) δ 7.33 – 7.29 (m, 4H), 7.23 – 7.20 (m, 6H), 6.32 (d, J = 10.4, Hz, 1H), 5.81 (d, J = 10.4 Hz, 1H), 4.83 – 4.76 (m, 1H), 2.00 (s, 3H), 1.85 – 1.81 (m, 1H), 1.71 – 1.67 (m, 2H), 1.51 (s, 2H), 1.40 (t, J =11.9 Hz, 1H), 1.13 – 0.96 (m, 3H), 0.92 (d, J = 6.5 Hz, 3H), 0.85 (d, J = 7.0 Hz, 3H), 0.75 (d, J = 6.9 Hz, 3H) ppm.

^{13}C{^{1}H} NMR (151 MHz, $CDCl_3$) δ 167.5, 143.84, 143.78, 141.9, 128.5, 128.3, 128.0, 126.3, 74.4, 49.3, 47.1, 40.9, 34.2, 31.4, 29.7, 26.2, 23.3, 22.0, 21.0, 20.8, 16.2 ppm.

See NMR Spectra

HRMS (*m/z*): (ESI) calc'd for $C_{27}H_{34}O_2{}^{23}Na$ $[M+Na]^+$: 413.2451, found: 413.2450.

IR (ATR) ν_{max}: 2953, 2926, 2868, 1727, 1707, 1217, 1132 and 699 cm^{-1}.

Melting point: 89 – 91 °C.

$[\alpha]_D^{24}$ = 30.22 (c = 0.01, CH_2Cl_2).

TLC: R_f = 0.49 (*n*-hexane/ethyl acetate 10:1, v/v)

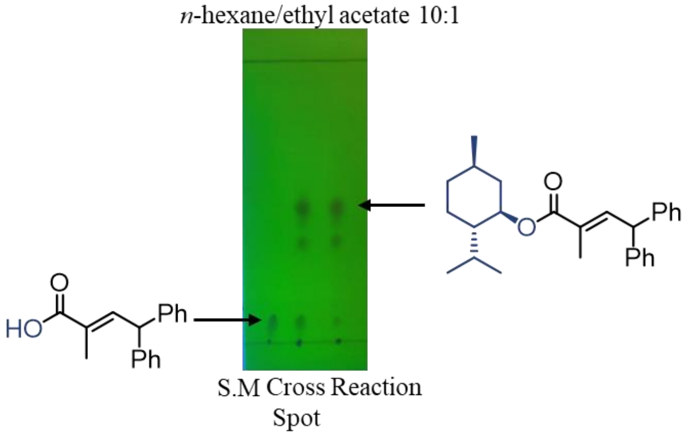

(3*S*,8*S*,9*S*,10*R*,13*R*,14*S*,17*R*)-10,13-Dimethyl-17-((*R*)-6-methylheptan-2-yl)-2,3,4,7,8,9,10,11,12,13,14,15,16,17-tetradecahydro-1*H*-cyclopenta[a]phenanthren-3-yl (*E*)-2-methyl-4,4-diphenylbut-2-enoate (1aj)

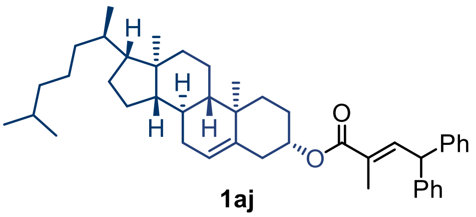

1aj

According to General Procedure D, **S3o** (0.10 g, 0.5 mmol, 1.0 equiv), cholesterol (270 mg, 0.7 mmol, 1.5 equiv), DCC (121 mg, 0.6 mmol, 1.2 equiv), and DMAP (12 mg, 0.1 mmol, 0.2 equiv) were used to obtain the crude residue, which was purified by silica gel column chromatography (*n*-hexane/ethyl acetate 100:1, v/v), to afford **1aj** as a white solid (176 mg, 0.3 mmol, 57%).

^{1}H NMR (400 MHz, $CDCl_3$) δ 7.32 – 7.29 (m, 5H), 7.22 (d, J = 6.5 Hz, 5H), 6.36 – 6.33 (m, 1H), 5.82 (d, J = 10.4 Hz, 1H), 5.40 (s, 1H), 4.74 – 4.67 (m, 1H), 2.35 – 2.31 (m, 2H), 2.04 – 1.97 (m, 5H), 1.89 – 1.86 (m, 3H), 1.59 – 1.55 (m, 4H), 1.53 – 1.49 (m, 2H), 1.45 (s, 1H), 1.37 – 1.35 (m, 2H), 1.27 (s, 1H), 1.18 – 1.07 (m, 5H), 1.03 (d, J = 2.3 Hz, 4H), 0.93 (d, J = 6.3 Hz, 3H), 0.88 (d, J = 6.5 Hz, 6H), 0.69 (s, 3H) ppm.

^{13}C{^{1}H} NMR (101 MHz, $CDCl_3$) δ 167.2, 143.8, 142.3, 139.6, 128.5, 128.3, 127.9, 126.3, 122.7, 74.2, 56.7, 56.1, 50.0, 49.3, 42.3, 39.7, 39.5, 38.1, 37.0, 36.6, 36.2, 35.8, 31.9, 31.8, 28.2, 28.0, 27.8, 24.3, 23.8, 22.8, 22.5, 21.0, 19.3, 18.7, 11.8 ppm.

See NMR Spectra

HRMS (*m/z*): (ESI) calc'd for $C_{44}H_{60}O_2{}^{23}Na$ [M+Na]$^+$: 643.4486, found: 643.4484.

IR (ATR) ν_{max}: 2933, 2867, 1710, 1216, 1133 and 698 cm^{-1}.

Melting point: 95 – 98 °C.

$[\alpha]_D^{24}$ = 25.41 (c = 0.01, CH_2Cl_2).

TLC: R_f = 0.55 (*n*-hexane/ethyl acetate 10:1, v/v)

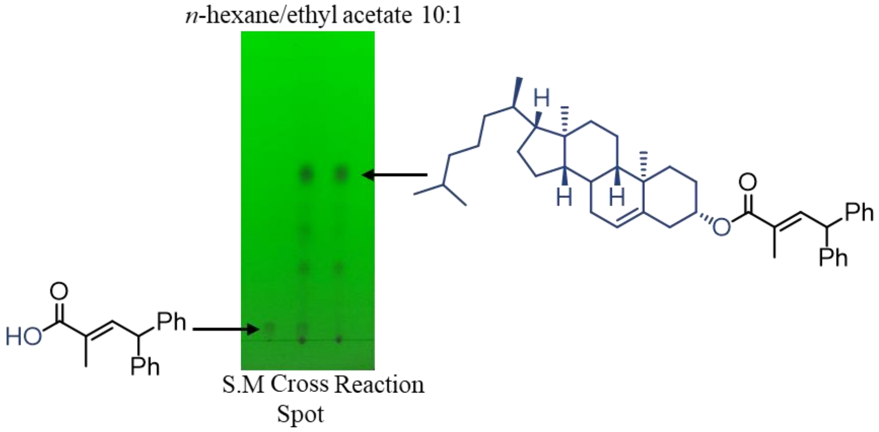

(1*S*,2*R*,4*S*)-1,7,7-Trimethylbicyclo[2.2.1]heptan-2-yl (*E*)-2-methyl-4,4-diphenylbut-2-enoate (1ak)

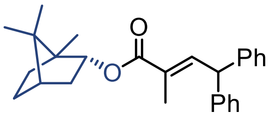

1ak

According to General Procedure D, **S3o** (0.10 g, 0.5 mmol, 1.0 equiv), (-)-borneol (106 mg, 0.7 mmol, 1.5 equiv), DCC (121 mg, 0.6 mmol, 1.2 equiv), and DMAP (12 mg, 0.1 mmol, 0.2 equiv) were used to obtain the crude residue, which was purified by silica gel column chromatography (*n*-hexane/ethyl acetate 100:1, v/v), to afford **1ak** as a white solid (144 mg, 0.4 mmol, 75%).

^{1}H NMR (400 MHz, $CDCl_3$) δ 7.33 – 7.29 (m, 4H), 7.24 – 7.20 (m, 6H), 6.34 (dd, J = 10.6, 1.2 Hz, 1H), 5.92 (d, J = 10.6 Hz, 1H), 5.00 – 4.97 (m, 1H), 2.42 – 2.41 (m, 1H), 2.04 (d, J = 0.96 Hz, 3H), 1.92 – 1.87 (m, 1H), 1.75 – 1.68 (m, 2H), 1.32 – 1.28 (m, 1H), 1.23 – 1.17 (m, 1H), 1.00 (dd, J = 13.8, 3.4 Hz, 1H), 0.93 (s, 3H), 0.88 (s, 3H), 0.85 (s, 3H) ppm.

^{13}C{^{1}H} NMR (101 MHz, $CDCl_3$) δ 168.1, 143.74, 143.70, 142.5, 128.5, 128.37, 128.35, 127.7, 126.4, 80.3, 49.1, 48.8, 47.8, 44.9, 37.0, 29.7, 28.0, 27.3, 21.1, 19.7, 18.8, 13.6 ppm.

See NMR Spectra

HRMS (*m/z*): (ESI) calc'd for $C_{27}H_{32}O_2{}^{23}Na$ $[M+Na]^+$: 411.2295, found: 411.2292.

IR (ATR) ν_{max}: 2951, 2877, 1709, 1452, 1217, 1131, 1018, 740 and 698 cm^{-1}.

Melting point: 86 – 88 °C.

$[\alpha]_D^{24}$ = 12.87 (c = 0.01, CH_2Cl_2).

TLC: R_f = 0.56 (*n*-hexane/ethyl acetate 8:1, v/v)

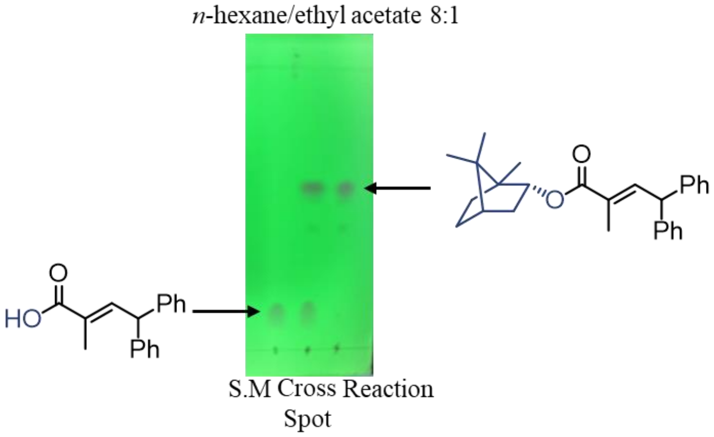

(3a*R*,5*R*,6*S*,6a*R*)-5-((*R*)-2,2-Dimethyl-1,3-dioxolan-4-yl)-2,2-dimethyltetrahydrofuro[2,3-d][1,3]dioxol-6-yl (*E*)-2-methyl-4,4-diphenylbut-2-enoate (1al)

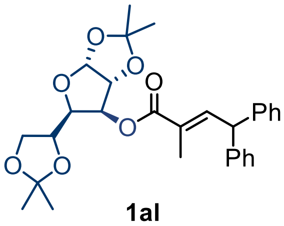

1al

According to General Procedure D, **S3o** (0.10 g, 0.5 mmol, 1.0 equiv), diacetone-D-glucose (183 mg, 0.7 mmol, 1.5 equiv), DCC (121 mg, 0.6 mmol, 1.2 equiv), and DMAP (12 mg, 0.1 mmol, 0.2 equiv) were used to obtain the crude residue, which was purified by silica gel column chromatography (*n*-hexane/ethyl acetate 100:1, v/v), to afford **1al** as a white solid (154 mg, 0.3 mmol, 63%).

^{1}H NMR (400 MHz, $CDCl_3$) δ 7.33 – 7.29 (m, 4H), 7.24 – 7.20 (m, 6H), 6.44 (dd, J = 3.6, 1.2 Hz, 1H), 5.85 (d, J = 10.4 Hz, 1H), 5.79 (d, J = 3.6 Hz, 1H), 5.31 (d, J = 2.9 Hz, 1H), 4.42 (d, J = 3.7 Hz, 1H), 4.21 (dd, J = 8.3, 3.0 Hz, 1H), 4.15 – 4.10 (m, 1H), 4.04 – 3.96 (m, 2H), 2.02 (d, J = 0.84, 3H), 1.53 (s, 3H), 1.41 (s, 3H), 1.30 (s, 3H), 1.26 (s, 3H) ppm.

^{13}C{^{1}H} NMR (101 MHz, $CDCl_3$) δ 166.2, 144.6, 143.5, 143.4, 128.5, 128.4, 128.3, 126.7, 126.53, 126.50, 112.2, 109.4, 105.0, 83.3, 79.9, 76.4, 72.4, 67.4, 49.2, 26.8, 26.7, 26.2, 25.1, 20.8 ppm.

See NMR Spectra

HRMS (*m/z*): (ESI) calc'd for $C_{29}H_{34}O_7{}^{23}Na$ $[M+Na]^+$: 517.2197, found: 517.2195.

IR (ATR) ν_{max}: 2986, 2988, 1717, 1494, 1208, 1074, 1021, 740 and 699 cm^{-1}.

Melting point: 87 – 89 °C.

$[\alpha]_D^{24}$ = 51.94 (c = 0.01, CH_2Cl_2).

TLC: R_f = 0.08 (*n*-hexane/ethyl acetate 10:1, v/v)

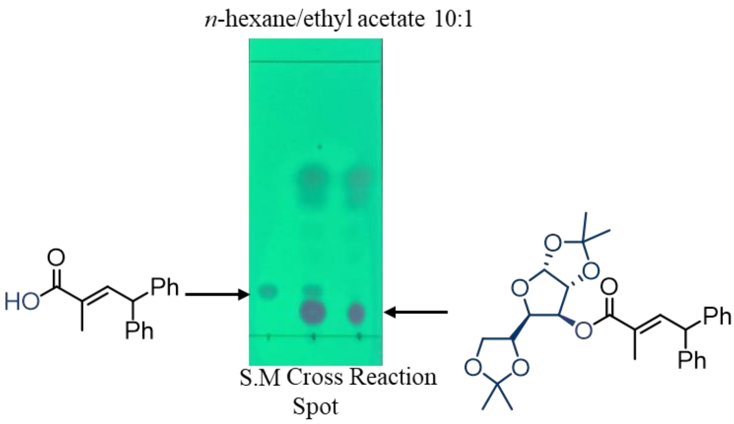

4.2 Characterization data of compound 2

[1,1'-Biphenyl]-4-ylmethyl (*S*)-2,4-dimethylpent-3-enoate (2a)

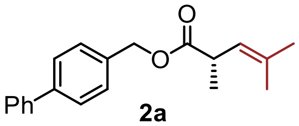

2a

According to General Procedure E, **1a** (20 mg, 0.06 mmol, 1.0 equiv) and (*R*)-**D3** or (*S*)-**D3** (4 mg, 10 mol%) were used to obtain the crude residue, which was purified by silica gel column chromatography (*n*-hexane/ethyl acetate 100:1, v/v), to afford (*S*)-**2a** as colorless oil (18 mg, 0.05 mmol, 89% yield, 94% *ee*) or (*R*)-**2a** as a colorless oil (17 mg, 0.05 mmol, 85% yield, -93% *ee*).

^{1}H NMR (400 MHz, $CDCl_3$) δ 7.59 (d, J = 7.1 Hz, 4H), 7.46 – 7.10 (m, 4H), 7.35 (t, J = 7.2 Hz, 1H), 5.20 – 5.16 (m, 3H), 3.44 – 3.36 (m, 1H), 1.73 (s, 3H), 1.67 (s, 3H), 1.24 (d, J = 7.0 Hz, 3H) ppm.

^{13}C{^{1}H} NMR (101 MHz, $CDCl_3$) δ 175.3, 141.0, 140.7, 135.3, 134.2, 128.8, 128.3, 127.4, 127.2, 127.1, 123.8, 65.8, 39.0, 25.7, 18.1, 18.0 ppm.

See NMR Spectra

HRMS (*m/z*): (ESI) calc'd for $C_{20}H_{22}O_2{}^{23}Na$ [M+Na]$^+$: 317.1512, found: 317.1512.

IR (ATR) ν_{max}: 3005, 2253, 1711, 1360, 1221, 912, 731, 648 and 530 cm^{-1}.

Chiral HPLC (Chiralpak® OJ-3, Hexane/^{i}PrOH = 90:10, 0.9 mL/min) *er* = 96.9:3.1, t_R: 15.6 min (major), t_R: 18.6 min (minor) for (*S*)-**2a**; *er* = 3.6:96.4, t_R: 15.1 min (minor), t_R: 17.7 min (major) for (*R*)-**2a**.

See HPLC Spectra

$[\alpha]_D^{24}$ = 3.83 (c = 0.01, CH_2Cl_2) for (*S*)-**2a**; -3.80 (c = 0.01, CH_2Cl_2) for (*R*)-**2a**.

TLC: R_f = 0.83 (*n*-hexane/ethyl acetate 5:1, v/v)

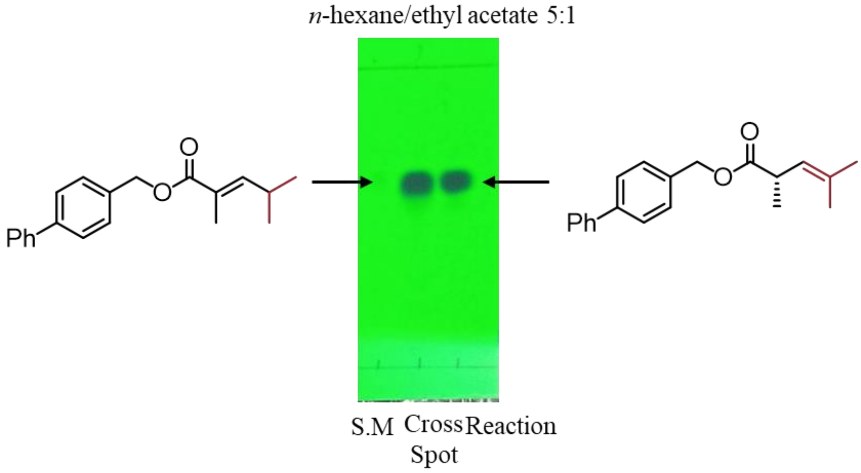

[1,1'-Biphenyl]-4-ylmethyl (*S*)-4-ethyl-2-methylhex-3-enoate (2b)

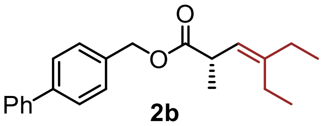

2b

According to General Procedure E, **1b** (20 mg, 0.06 mmol, 1.0 equiv) and (*R*)-**D3** (4 mg, 10 mol%) were used to obtain the crude residue, which was purified by silica gel column chromatography (*n*-hexane/ethyl acetate 100:1, v/v), to afford **2b** as a colorless oil (13 mg, 0.04 mmol, 65% yield, 95% *ee*).

^{1}H NMR (600 MHz, $CDCl_3$) δ 7.61 – 7.58 (m, 4H), 7.47 – 7.41 (m, 4H), 7.38 – 7.35 (m, 1H), 5.17 – 5.15 (m, 3H), 3.48 – 3.45 (m, 1H), 2.15 – 2.04 (m, 4H), 1.27 (d, J = 4.6 Hz, 3H), 1.03 – 0.97 (m, 6H) ppm.

^{13}C{^{1}H} NMR (151 MHz, $CDCl_3$) δ 175.4, 145.3, 141.0, 140.7, 135.3, 128.8, 128.3, 127.4, 127.2, 127.1, 121.8, 65.8, 38.6, 29.0, 23.6, 18.4, 13.2, 12.6 ppm.

See NMR Spectra

HRMS (*m/z*): (ESI) calc'd for $C_{22}H_{26}O_2{}^{23}Na$ $[M+Na]^+$: 345.1825, found: 345.1827.

IR (ATR) ν_{max}: 2955, 2916, 1709, 1376, 1260, 1100, 1025 and 804 cm^{-1}.

Chiral HPLC (Chiralpak® OJ-3, Hexane/^{i}PrOH = 90:10, 0.8 mL/min) *er* = 97.3:2.7, t_R: 10.0 min (major), t_R: 10.6 min (minor).

See HPLC Spectra

$[\alpha]_D^{24}$ = 5.52 (c = 0.01, CH_2Cl_2).

TLC: R_f = 0.51 (*n*-hexane/ethyl acetate 10:1, v/v)

n-hexane/ethyl acetate 10:1

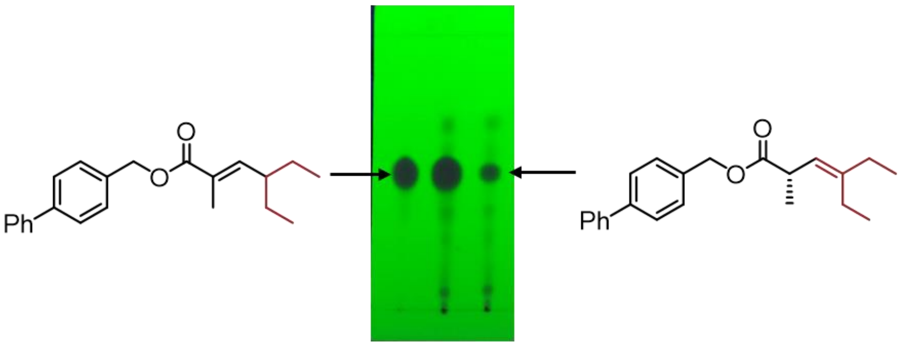

S.M Cross Reaction
Spot

[1,1'-Biphenyl]-4-ylmethyl (*S*)-2-methylbut-3-enoate (2c)

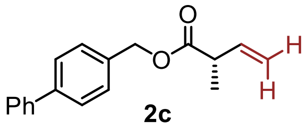

2c

According to General Procedure E, **1c** (20 mg, 0.06 mmol, 1.0 equiv) and (*R*)-**D3** (4 mg, 10 mol%) were used to obtain the crude residue, which was purified by silica gel column chromatography (*n*-hexane/ethyl acetate 100:1, v/v), to afford **2c** as a colorless oil (14 mg, 0.04 mmol, 71% yield, 46% *ee*).

^{1}H NMR (400 MHz, $CDCl_3$) δ 7.59 (d, J = 8.2 Hz, 4H), 7.46 – 7.41 (m, 4H), 7.36 (t, J = 7.3 Hz, 1H), 6.01 – 5.92 (m, 1H), 5.19 – 5.12 (m, 4H), 3.27 – 3.20 (m, 1H), 1.32 (d, J = 7.0 Hz, 3H) ppm.

^{13}C{^{1}H} NMR (101 MHz, $CDCl_3$) δ 174.3, 141.1, 140.7, 137.0, 135.0, 128.8, 128.5, 127.4, 127.3, 127.1, 116.1, 66.1, 43.7, 16.7 ppm.

See NMR Spectra

HRMS (*m/z*): (ESI) calc'd for $C_{18}H_{18}O_2{}^{23}Na$ $[M+Na]^+$: 289.1199, found: 289.1198.

IR (ATR) ν_{max}: 2961, 2917, 2849, 1735, 1259, 1014, 762 and 697 cm^{-1}.

Chiral HPLC (Chiralpak® OJ-3, Hexane/^{i}PrOH = 99:01, 0.9 mL/min) *er* = 73.1:26.9, t_R: 41.7 min (major), t_R: 43.5 min (minor).

See HPLC Spectra

$[\alpha]_D^{24}$ = 3.86 (c = 0.01, CH_2Cl_2).

TLC: R_f = 0.43 (*n*-hexane/ethyl acetate 10:1, v/v)

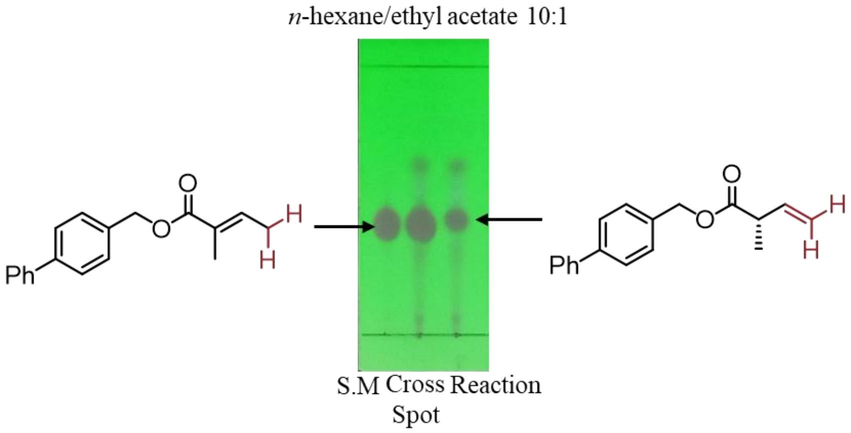

Back to contents / Back to substrate scope table

[1,1'-Biphenyl]-4-ylmethyl (*S*)-3-cyclobutylidene-2-methylpropanoate (2d)

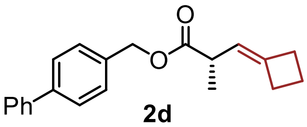

2d

According to General Procedure E, **1d** (20 mg, 0.06 mmol, 1.0 equiv) and (*R*)-**D5** (4 mg, 10 mol%) were used to obtain the crude residue, which was purified by silica gel column chromatography (*n*-hexane/ethyl acetate 100:1, v/v), to afford **2d** as a colorless oil (18 mg, 0.06 mmol, 91% yield, 83% *ee*).

^{1}H NMR (600 MHz, $CDCl_3$) δ 7.59 (d, J = 5.6 Hz, 4H), 7.46 – 7.41 (m, 4H), 7.37 – 7.35 (m, 1H), 5.16 – 5.13 (m, 3H), 3.17 – 3.15 (m, 1H), 2.73 – 2.63 (m, 4H), 1.97 – 1.92 (m, 2H), 1.24 (d, J = 7.1 Hz, 3H) ppm.

^{13}C{^{1}H} NMR (151 MHz, $CDCl_3$) δ 175.2, 143.1, 141.0, 140.7, 135.3, 128.8, 128.3, 127.4, 127.2, 127.1, 119.3, 65.8, 39.2, 30.9, 29.3, 17.8, 16.9 ppm.

See NMR Spectra

HRMS (*m/z*): (ESI) calc'd for $C_{21}H_{22}O_2{}^{23}Na$ [M+Na]$^+$: 329.1512, found: 329.1513.

IR (ATR) ν_{max}: 2930, 2856, 1707, 1643, 1254, 1103, 761 and 697 cm^{-1}.

Chiral HPLC (Chiralpak® IA-3, Hexane/^{i}PrOH = 99:01, 0.5 mL/min) *er* = 91.6:8.4, t_R: 20.0 min (major), t_R: 22.5 min (minor).

See HPLC Spectra

$[\alpha]_D^{24}$ = 22.23 (c = 0.01, CH_2Cl_2).

TLC: R_f = 0.34 (*n*-hexane/ethyl acetate 10:1, v/v)

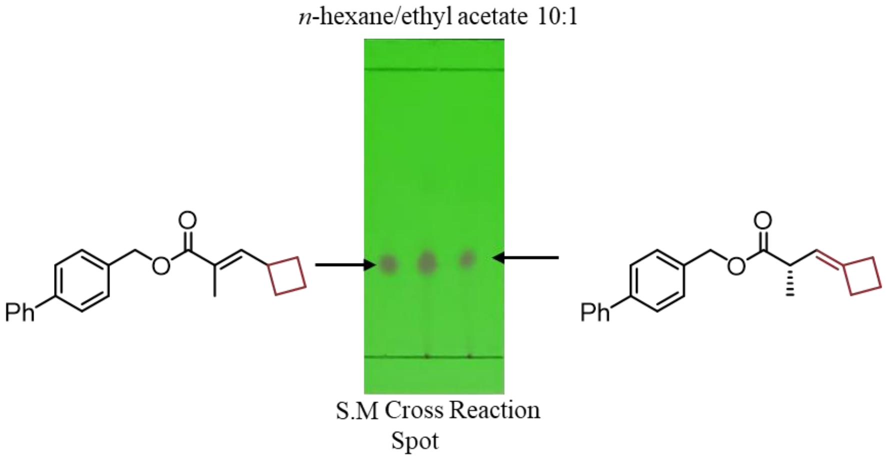

[1,1'-Biphenyl]-4-ylmethyl (*S*)-3-cyclopentylidene-2-methylpropanoate (2e)

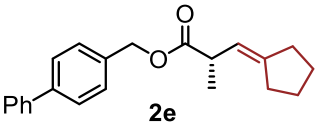

2e

According to General Procedure E, **1e** (20 mg, 0.06 mmol, 1.0 equiv) and (*R*)-**D3** (4 mg, 10 mol%) were used to obtain the crude residue, which was purified by silica gel column chromatography (*n*-hexane/ethyl acetate 100:1, v/v), to afford **2e** as a colorless oil (12 mg, 0.03 mmol, 59% yield, 80% *ee*).

^{1}H NMR (600 MHz, $CDCl_3$) δ 7.60 – 7.58 (m, 4H), 7.46 – 7.40 (m, 4H), 7.37 – 7.34 (m, 1H), 5.32 – 5.29 (m, 1H), 5.18 – 5.14 (m, 2H), 3.32 – 3.27 (m, 1H), 2.30 – 2.17 (m, 4H), 1.69 – 1.59 (m, 4H), 1.25 (d, *J* = 7.0 Hz, 3H) ppm.

^{13}C{^{1}H} NMR (151 MHz, $CDCl_3$) δ 175.3, 146.1, 141.0, 140.7, 135.3, 128.8, 128.3, 127.4, 127.2, 127.1, 119.1, 65.8, 40.7, 33.7, 30.9, 28.8, 26.3, 17.8 ppm.

See NMR Spectra

HRMS (*m/z*): (ESI) calc'd for $C_{22}H_{24}O_2{}^{23}Na$ [M+Na]$^+$: 343.1669, found: 343.1667.

IR (ATR) ν_{max}: 2932, 2856, 1706, 1643, 1254, 1104, 761 and 697 cm^{-1}.

Chiral HPLC (Chiralpak® IA-3, Hexane/^{i}PrOH = 99:01, 0.5 mL/min) *er* = 89.8:10.2, t_R: 23.8 min (major), t_R: 24.4 min (minor).

See HPLC Spectra

$[\alpha]_D^{24}$ = 34.86 (c = 0.01, CH_2Cl_2).

TLC: R_f = 0.51 (*n*-hexane/ethyl acetate 8:1, v/v)

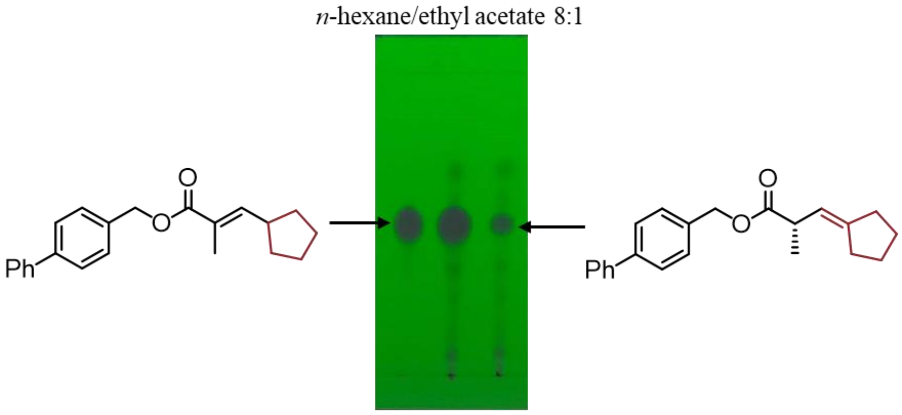

[1,1'-Biphenyl]-4-ylmethyl (*S*)-3-cyclohexylidene-2-methylpropanoate (2f)

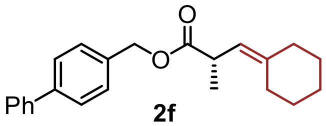

2f

According to General Procedure E, **1f** (20 mg, 0.06 mmol, 1.0 equiv) and (*R*)-**D3** (4 mg, 10 mol%) were used to obtain the crude residue, which was purified by silica gel column chromatography (*n*-hexane/ethyl acetate 100:1, v/v), to afford **2f** as a colorless oil (19 mg, 0.06 mmol, 95% yield, 84% *ee*).

^{1}H NMR (600 MHz, $CDCl_3$) δ 7.60 – 7.58 (m, 4H), 7.46 – 7.40 (m, 4H), 7.37 – 7.35 (m, 1H), 5.20 (d, J = 9.2 Hz, 1H), 5.16 (s, 2H), 3.43 – 3.38 (m, 1H), 2.17 (s, 1H), 1.70 (d, J = 33.7 Hz, 6H), 1.60 (s, 1H), 1.26 – 1.24 (m, 5H) ppm.

^{13}C{^{1}H} NMR (151 MHz, $CDCl_3$) δ 175.3, 141.0, 140.7, 135.3, 134.2, 128.8, 128.3, 127.4, 127.2, 127.1, 123.7, 65.8, 39.0, 25.7, 18.1, 18.0 ppm.

See NMR Spectra

HRMS (*m/z*): (ESI) calc'd for $C_{23}H_{26}O_2{}^{23}Na$ [M+Na]$^+$: 357.1825, found: 357.1827.

IR (ATR) ν_{max}: 2918, 2849, 1734, 1377, 1156, 1064, 821, 760 and 697 cm^{-1}.

Chiral HPLC (Chiralpak® IA-3, Hexane/^{i}PrOH = 99:01, 0.8 mL/min) *er* = 92.2:7.8, t_R: 13.7 min (major), t_R: 13.1 min (minor).

See HPLC Spectra

$[\alpha]_D^{24}$ = 12.55 (c = 0.01, CH_2Cl_2).

TLC: R_f = 0.49 (*n*-hexane/ethyl acetate 10:1, v/v)

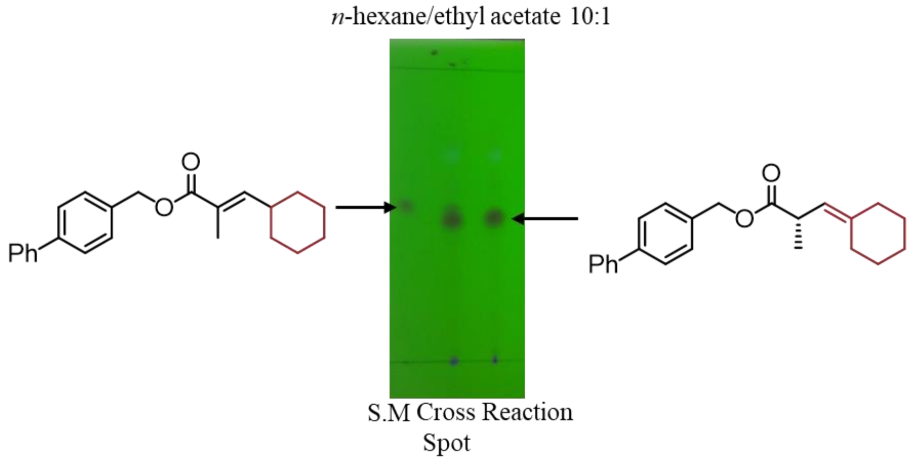

[1,1'-Biphenyl]-4-ylmethyl (*S*)-2-methyl-3-(tetrahydro-4*H*-pyran-4-ylidene)propanoate (2g)

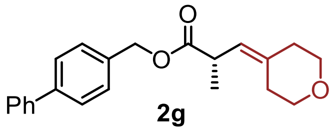

2g

According to General Procedure E, **1g** (20 mg, 0.06 mmol, 1.0 equiv) and (*R*)-**D3** (3 mg, 10 mol%) were used to obtain the crude residue, which was purified by silica gel column chromatography (*n*-hexane/ethyl acetate 10:1, v/v), to afford **2g** as a colorless oil (16 mg, 0.05 mmol, 80% yield, 87% *ee*).

^{1}H NMR (600 MHz, $CDCl_3$) δ 7.59 (d, J = 8.1 Hz, 4H), 7.46 – 7.40 (m, 4H), 7.37 – 7.34 (m, 1H), 5.25 (d, J = 9.2 Hz, 1H), 5.19 – 5.13 (m, 2H), 3.70 – 3.57 (m, 4H), 3.45 – 3.42 (m, 1H), 2.36 – 2.32 (m, 1H), 2.29 – 2.21 (m, 3H), 1.27 (d, J = 7.0 Hz, 3H) ppm.

^{13}C{^{1}H} NMR (151 MHz, $CDCl_3$) δ 174.9, 141.2, 140.6, 136.9, 135.1, 128.8, 128.5, 127.5, 127.3, 127.1, 122.3, 69.4, 68.5, 66.0, 37.9, 36.7, 30.1, 18.2 ppm.

See NMR Spectra

HRMS (*m/z*): (ESI) calc'd for $C_{22}H_{24}O_3{}^{23}Na$ $[M+Na]^+$: 359.1618, found: 359.1618.

IR (ATR) ν_{max}: 2914, 2849, 1710, 1256, 1016, 762 and 697 cm^{-1}.

Chiral HPLC (Chiralpak® IA-3, Hexane/^{i}PrOH = 99:01, 0.8 mL/min) *er* = 6.3:93.7, t_R: 15.6 min (minor), t_R: 16.3 min (major).

See HPLC Spectra

$[\alpha]_D^{24}$ = 4.28 (c = 0.01, CH_2Cl_2).

TLC: R_f = 0.24 (*n*-hexane/ethyl acetate 10:1, v/v)

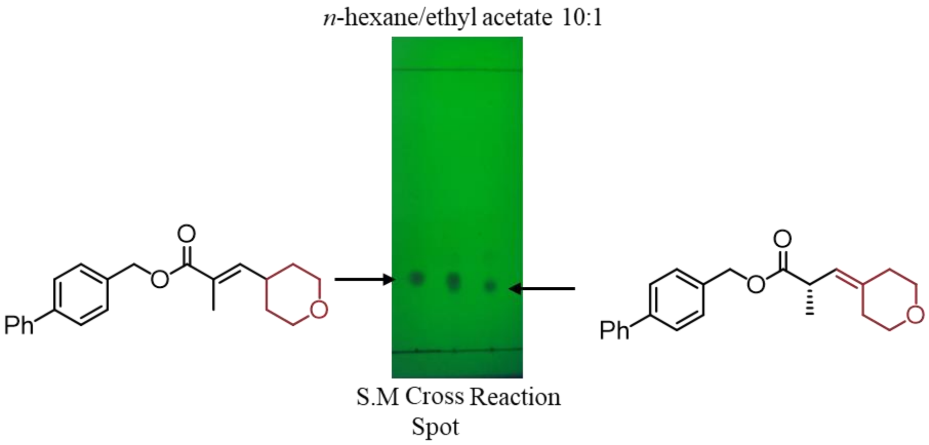

Back to contents / Back to substrate scope table

tert-Butyl (S)-4-(3-([1,1'-biphenyl]-4-ylmethoxy)-2-methyl-3-oxopropylidene)piperidine-1-carboxylate (2h)

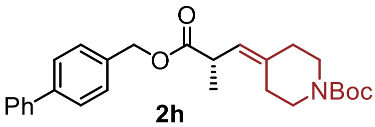

2h

According to General Procedure E, **1h** (20 mg, 0.06 mmol, 1.0 equiv) and (*R*)-**D3** (3 mg, 10 mol%) were used to obtain the crude residue, which was purified by silica gel column chromatography (*n*-hexane/ethyl acetate 10:1, v/v), to afford **2h** as a colorless oil (14 mg, 0.04 mmol, 68% yield, 87% *ee*).

^{1}H NMR (400 MHz, $CDCl_3$) δ 7.58 (d, J = 7.9 Hz, 4H), 7.46 – 7.33 (m, 5H), 5.28 (d, J = 9.2 Hz, 1H), 5.19 – 5.12 (m, 2H), 3.46 – 3.40 (m, 3H), 3.35 – 3.26 (m, 2H), 2.28 – 2.13 (m, 4H), 1.45 (s, 9H), 1.27 (d, J = 6.9 Hz, 3H) ppm.

^{13}C{^{1}H} NMR (101 MHz, $CDCl_3$) δ 174.8, 154.7, 141.2, 140.6, 137.7, 135.1, 128.8, 128.5, 127.4, 127.3, 127.1, 123.0, 79.5, 66.0, 38.1, 35.7, 29.7, 28.8, 28.4, 18.2 ppm.

See NMR Spectra

HRMS (*m/z*): (ESI) calc'd for $C_{27}H_{33}O_4N^{23}Na$ $[M+Na]^+$: 458.2302, found: 458.2301.

IR (ATR) ν_{max}: 2917, 2849, 1733, 1689, 1422, 1240, 1164, 761 and 733 cm^{-1}.

Chiral HPLC (Chiralpak® IC-3, Hexane/^{i}PrOH = 93:07, 0.9 mL/min) *er* = 93.5:6.5, t_R: 22.8 min (major), t_R: 23.9 min (minor).

See HPLC Spectra

$[\alpha]_D^{24}$ = 55.80 (c = 0.01, CH_2Cl_2).

TLC: R_f = 0.56 (*n*-hexane/ethyl acetate 4:1, v/v)

n-hexane/ethyl acetate 4:1

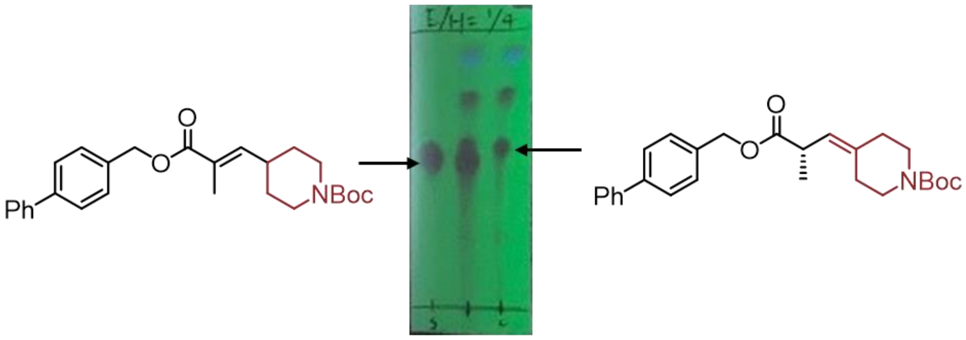

S.M Cross Reaction Spot

Benzyl (*S*)-4-(3-([1,1'-biphenyl]-4-ylmethoxy)-2-methyl-3-oxopropylidene)piperidine-1-carboxylate (2i)

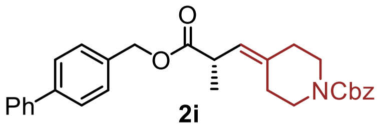

2i

According to General Procedure E, **1i** (20 mg, 0.06 mmol, 1.0 equiv) and (*R*)-**D3** (2 mg, 10 mol%) were used to obtain the crude residue, which was purified by silica gel column chromatography (*n*-hexane/ethyl acetate 10:1, v/v), to afford **2i** as a colorless oil (11 mg, 0.03 mmol, 56% yield, 92% *ee*).

^{1}H NMR (400 MHz, $CDCl_3$) δ 7.59 – 7.57 (m, 4H), 7.46 – 7.30 (m, 10H), 5.29 (d, J = 9.3 Hz, 1H), 5.16 – 5.12 (m, 4H), 3.56 – 3.34 (m, 5H), 2.29 – 2.17 (m, 4H), 1.27 (d, J = 6.5 Hz, 3H) ppm.

^{13}C{^{1}H} NMR (101 MHz, $CDCl_3$) δ 174.7, 155.2, 141.2, 140.6, 137.2, 136.8, 135.0, 128.8, 128.5, 128.0, 127.8, 127.5, 127.3, 127.1, 123.4, 67.1, 66.1, 45.6, 44.7, 38.1, 29.7, 18.1 ppm.

See NMR Spectra

HRMS (*m/z*): (ESI) calc'd for $C_{30}H_{31}O_4N^{23}Na$ $[M+Na]^+$: 492.2145, found: 492.2144.

IR (ATR) ν_{max}: 3030, 2917, 2850, 1731, 1697, 1428, 1222, 762 and 697 cm^{-1}.

Chiral HPLC (Chiralpak® IA-3, Hexane/^{i}PrOH = 90:10, 0.9 mL/min) *er* = 4.1:95.9, t_R: 16.0 min (minor), t_R: 16.6 min (major).

See HPLC Spectra

$[\alpha]_D^{24}$ = 44.17 (c = 0.01, CH_2Cl_2).

TLC: R_f = 0.47 (*n*-hexane/ethyl acetate 3:1, v/v)

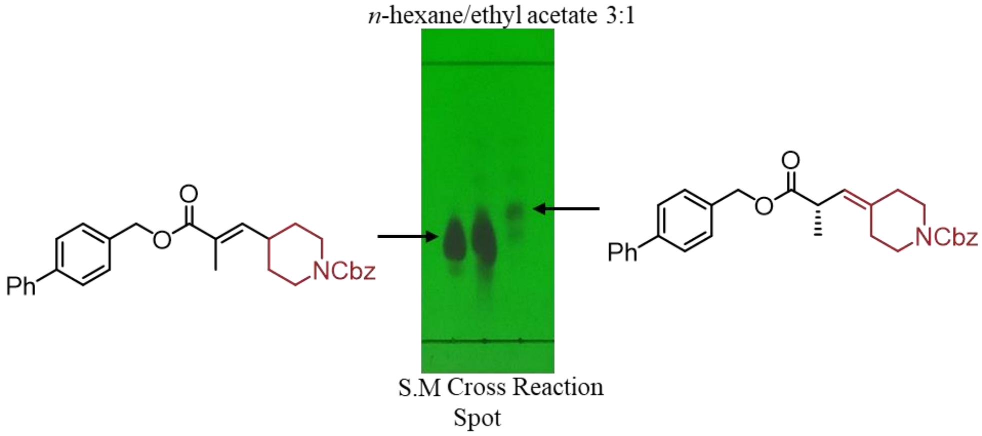

[1,1'-Biphenyl]-4-ylmethyl (*S*)-2-methyl-3-(1-((4-nitrophenyl)sulfonyl)piperidin-4-ylidene)propanoate (2j)

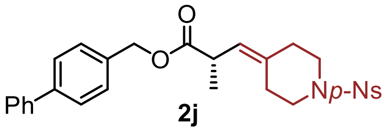

2j

To a solution of **2h** (70 mg, 0.2 mmol, 1.0 equiv) in DCM (3.0 mL) was added TFA (0.1 mL, 1.6 mmol, 10.0 equiv) in an ice bath and the mixture was stirred at rt for 18 h. Then the mixture was added saturated $NaHCO_3$ in an ice bath and extracted with diethyl ether. The organic layer was washed with brine and water, dried over Na_2SO_4 and concentrated under reduced pressure to give a yellow solid. The yellow solid was dissolved in CH_2Cl_2 (3 mL) and the solution was added triethyl amine (0.1 mL). Then 4-nitrobenzenesulfonyl chloride was added to the mixture in an ice bath and the reaction was stirred at rt for 18 h. Then the mixture was added water (1 mL) in an ice bath and extracted with CH_2Cl_2. The organic layer was washed with water, dried over Na_2SO_4 and concentrated under reduced pressure to give the crude product. The crude product was purified by column chromatography (*n*-hexane/ethyl acetate 10:1, v/v) to afford **2j** as a white solid (33 mg, 0.1 mmol, 38%).

^{1}H NMR (400 MHz, $CDCl_3$) δ 8.30 (d, J = 8.8 Hz, 2H), 7.89 (d, J = 8.8 Hz, 2H), 7.59 – 7.54 (m, 4H), 7.48 – 7.44 (m, 2H), 7.39 – 7.33 (m, 3H), 5.27 (d, J = 9.2 Hz, 1H), 5.11 – 5.10 (m, 2H), 3.37 – 3.33 (m, 1H), 3.20 – 3.12 (m, 2H), 3.03 – 2.96 (m, 2H), 2.42 – 2.33 (m, 2H), 2.29 (t, J = 5.6 Hz, 2H), 1.22 (d, J = 7.0 Hz, 3H) ppm.

^{13}C{^{1}H} NMR (101 MHz, $CDCl_3$) δ 174.3, 150.1, 142.8, 141.3, 140.5, 135.0, 134.8, 128.9, 128.6, 128.4, 127.6, 127.2, 127.1, 124.6, 124.3, 66.1, 47.6, 46.9, 38.1, 35.1, 29.9, 28.2, 18.1 ppm.

See NMR Spectra

HRMS (*m/z*): (ESI) calc'd for $C_{28}H_{28}N_2O_6{}^{23}Na^{32}S$ [M+Na]$^+$: 543.1560, found 543.1558.

IR (ATR) ν_{max}: 2972, 2901, 1732, 1530, 1350, 1167, 1050, 742, 699 cm^{-1}.

TLC: R_f = 0.96 (*n*-hexane/ethyl acetate 1:1 + 1% Et_3N, v/v)

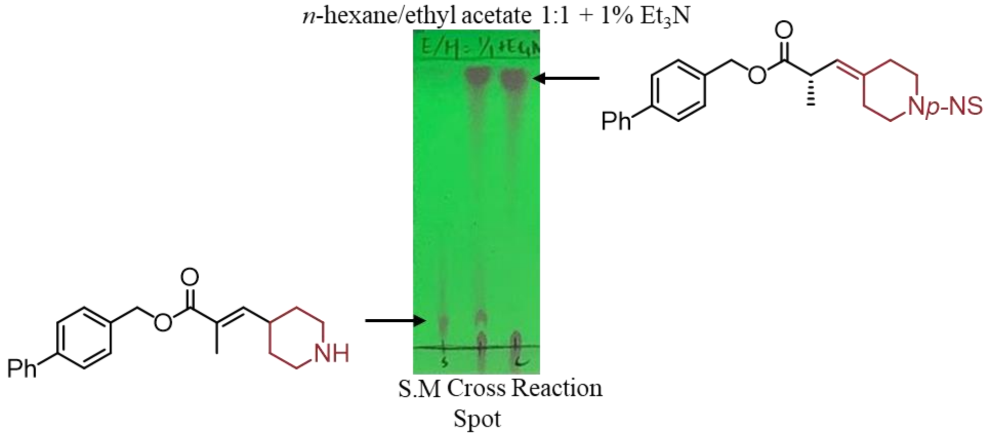

Benzyl (*S*)-2-methyl-4-phenylpent-3-enoate (2k)

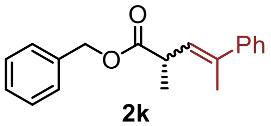

2k

According to General Procedure E, **1k** (20 mg, 0.06 mmol, 1.0 equiv) and (*R*)-**D5** or (*S*)-**D5** (5 mg, 10 mol%) were used to obtain the crude residue, which was purified by silica gel column chromatography (*n*-hexane/ethyl acetate 100:1, v/v), to afford (*S*)-**2k** as colorless oil (14 mg, 0.04 mmol, 72% yield, *E*/*Z* = 1:3.8, *E-isomer* 93% *ee*, *Z-isomer* 94% *ee*) or (*R*)-**2k** as a colorless oil (17 mg, 0.05 mmol, 88% yield, *E*/*Z* = 1:3.8, *E-isomer* -90% *ee*, *Z-isomer* -89% *ee*).

^{1}H NMR (600 MHz, $CDCl_3$) *E-isomer*: δ 7.38 – 7.16 (m, 10H), 5.80 – 5.78 (m, 1H), 5.16 (ABq, J = 6.9 Hz, 2H), 3.59 – 3.57 (m, 1H), 2.09 (s, 3H), 1.35 (d, J = 7.0 Hz, 3H) ppm; *Z-isomer*: δ 7.38 – 7.16 (m, 10H), 5.53 – 5.51 (m, 1H), 5.14 (ABq, J = 6.9 Hz, 2H), 3.26 – 3.21 (m, 1H), 2.05 (s, 3H), 1.20 (d, J = 7.0 Hz, 3H) ppm.

^{13}C{^{1}H} NMR (151 MHz, $CDCl_3$) δ 175.1, 174.7, 143.1, 141.3, 138.9, 137.0, 136.2, 128.51, 128.47, 128.22, 128.19, 128.1, 128.0, 127.93, 127.86, 127.7, 127.1, 126.88, 126.85, 126.0, 125.8, 66.3, 66.1, 39.8, 39.7, 25.8, 18.4, 17.9 ppm.

See NMR Spectra

HRMS (*m/z*): (ESI) calc'd for $C_{19}H_{20}O_2{}^{23}Na$ [M+Na]$^+$: 303.1356, found: 303.1354.

IR (ATR) ν_{max}: 2969, 2932, 1732, 1454, 1162, 752 and 699 cm^{-1}.

Chiral HPLC (Chiralpak® IA-3, Hexane/^{i}PrOH = 99:01, 0.7 mL/min) *E-isomer er* = 96.4:3.6, t_R: 10.2 min (major), t_R: 10.8 min (minor), and *Z-isomer er* = 96.9:3.1, t_R: 12.2 min (major), t_R = 12.5 min (minor) for (*S*)-**2k**; *E-isomer er* = 4.8:95.2, t_R: 10.6 min (minor), t_R: 11.0 min (major), and *Z-isomer er* = 5.5:94.5, t_R: 12.0 min (minor), t_R = 12.5 min (major) for (*R*)-**2k**.

See HPLC Spectra

$[\alpha]_D^{24}$ = 59.25 (c = 0.01, CH_2Cl_2) for (*S*)-**2k**; -58.99 (c = 0.01, CH_2Cl_2) for (*R*)-**2k**.

TLC: R_f = 0.67 (*n*-hexane/ethyl acetate 6:1, v/v)

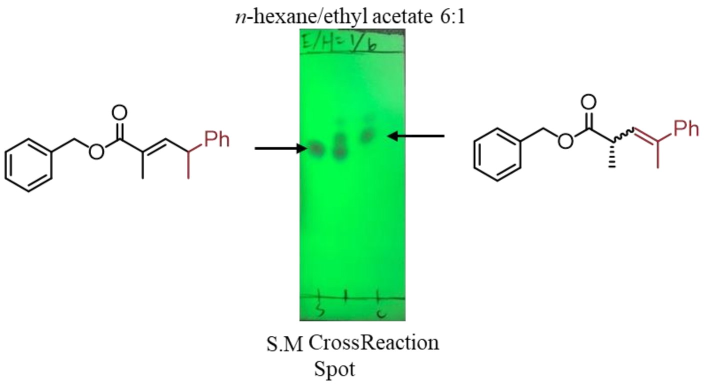

Benzyl (*S*)-2-methyl-4-phenylhex-3-enoate (2l)

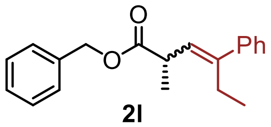

2l

According to General Procedure E, **1l** (20 mg, 0.06 mmol, 1.0 equiv) and (*R*)-**D5** (5 mg, 10 mol%) were used to obtain the crude residue, which was purified by silica gel column chromatography (*n*-hexane/ethyl acetate 100:1, v/v), to afford **2l** as a colorless oil (12 mg, 0.04 mmol, 61% yield, *E*/*Z* = 1:2.7, *E-isomer* 91% *ee*, *Z-isomer* 91% *ee*).

^{1}H NMR (600 MHz, $CDCl_3$) *E-isomer*: δ 7.38 – 7.11 (m, 10H), 5.63 (d, *J* = 10.0 Hz, 1H), 5.14 – 5.11 (m, 2H), 3.59 – 3.58 (m, 1H), 2.56 – 2.53 (m, 2H), 1.35 (s, 3H), 0.98 – 0.95 (m, 3H) ppm; *Z-isomer*: δ 7.38 – 7.11 (m, 10H), 5.48 (d, *J* = 10.1 Hz, 1H), 5.11 – 5.08 (m, 2H), 3.18 – 3.15 (m, 1H), 2.36 – 2.32 (m, 2H), 1.18 (s, 3H), 0.98 – 0.95 (m, 3H) ppm.

^{13}C{^{1}H} NMR (151 MHz, $CDCl_3$) δ 175.2, 174.7, 145.1, 143.7, 142.2, 140.7, 136.2, 136.1, 128.51, 128.46, 128.2, 128.12, 128.10, 128.0, 127.9, 127.8, 127.03, 126.99, 126.8, 126.5, 124.4, 66.3, 66.0, 39.6, 39.4, 32.2, 29.7, 23.3, 18.4, 18.3, 13.6, 12.7 ppm.

See NMR Spectra

HRMS (*m/z*): (ESI) calc'd for $C_{20}H_{22}O_2{}^{23}Na$ [M+Na]$^+$: 317.1512, found: 317.1511.

IR (ATR) ν_{max}: 2967, 2932, 1732, 1455, 1161, 753 and 699 cm^{-1}.

Chiral HPLC (Chiralpak® IA-3, Hexane/^{i}PrOH = 99:01, 0.7 mL/min) *E-isomer er* = 95.5:4.5, t_R: 10.5 min (major), t_R: 11.3 min (minor); *Z-isomer er* = 95.4:4.6, t_R: 12.6 min (major), t_R = 12.8 min (minor).

See HPLC Spectra

$[\alpha]_D^{24}$ = 22.41 (c = 0.01, CH_2Cl_2).

TLC: R_f = 0.28 (*n*-hexane/ethyl acetate 10:1, v/v)

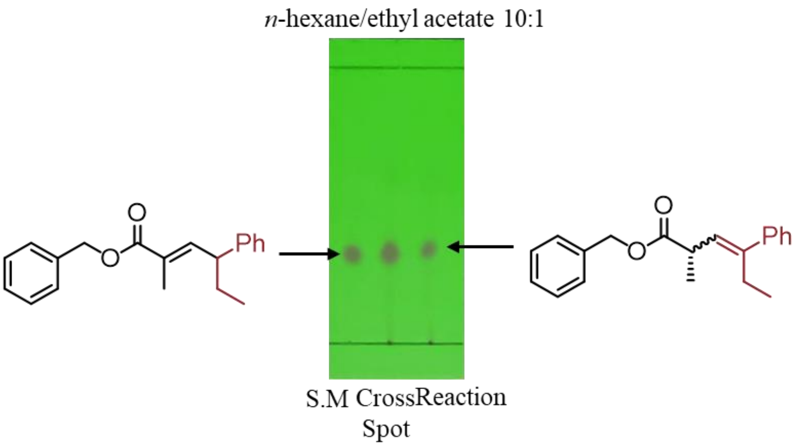

Benzyl (*S*)-2,5-dimethyl-4-phenylhex-3-enoate (2m)

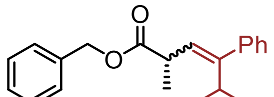

2m

According to General Procedure E, **1m** (20 mg, 0.06 mmol, 1.0 equiv) and (*R*)-**D5** (5 mg, 10 mol%) were used to obtain the crude residue, which was purified by silica gel column chromatography (*n*-hexane/ethyl acetate 100:1, v/v), to afford **2m** as a colorless oil (14 mg, 0.04 mmol, 71% yield, *E*/*Z* = 1:1, *E-isomer* 91% *ee*, *Z-isomer* 91% *ee*).

^{1}H NMR (600 MHz, $CDCl_3$) *E-isomer*: δ 7.37 – 7.04 (m, 10H), 5.47 (d, *J* = 9.5 Hz, 1H), 5.48 – 5.28 (m, 2H), 3.67 – 3.65 (m, 1H), 3.10 – 3.02 (m, 1H), 1.33 (d, *J* = 6.9 Hz, 3H), 1.05 – 0.99 (m, 6H) ppm. *Z-isomer*: δ 7.37 – 7.04 (m, 10H), 5.28 (d, *J* = 9.7 Hz, 1H), 5.19 – 5.11 (m, 2H), 3.10 – 3.02 (m, 1H), 2.57 – 2.52 (m, 1H), 1.16 (d, *J* = 7.0 Hz, 3H), 1.05 – 0.99 (m, 6H) ppm.

^{13}C{^{1}H} NMR (151 MHz, $CDCl_3$) δ 175.2, 174.7, 149.6, 148.8, 142.2, 140.2, 136.3, 136.2, 128.7, 128.6, 128.5, 128.4, 128.1, 128.0, 127.93, 127.89, 127.7, 127.5, 127.4, 126.6, 126.5, 123.5, 66.2, 65.9, 39.6, 38.6, 36.0, 29.6, 21.9, 21.6, 21.5, 18.34, 18.28 ppm.

See NMR Spectra

HRMS (*m/z*): (ESI) calc'd for $C_{21}H_{24}O_2{}^{23}Na$ $[M+Na]^+$: 331.1669, found: 331.1668.

IR (ATR) ν_{max}: 2961, 2925, 1733, 1455, 1159, 1029, 752 and 701 cm^{-1}.

Chiral HPLC (Chiralpak® IA-3, Hexane/^{i}PrOH = 97:03, 0.5 mL/min) *E-isomer er* = 95.5:4.5, t_R: 10.7 min (major), t_R: 11.3 min (minor); *Z-isomer er* = 95.4:4.6, t_R: 12.9 min (major), t_R = 13.1 min (minor).

See HPLC Spectra

$[\alpha]_D^{24}$ = 32.12 (c = 0.01, CH_2Cl_2).

TLC: R_f = 0.62 (*n*-hexane/ethyl acetate 6:1, v/v)

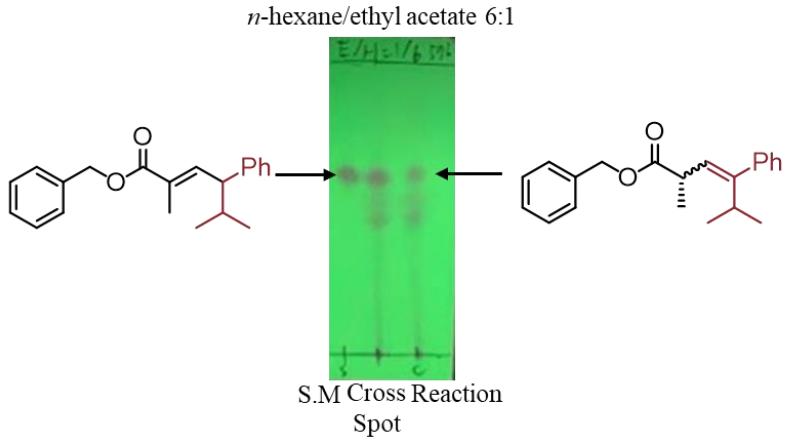

Benzyl (*S*)-2,5-dimethyl-4-(*p*-tolyl)hex-3-enoate (2n)

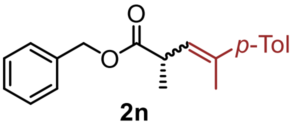

2n

According to General Procedure E, **1n** (20 mg, 0.06 mmol, 1.0 equiv) and (*R*)-**D5** (5 mg, 0.006 mmol, 0.1 equiv) were used to obtain the crude residue, which was purified by silica gel column chromatography (*n*-hexane/ethyl acetate 100:1, v/v), to afford **2n** as a colorless oil (9.0 mg, 0.03 mmol, 45% yield, *E*/*Z* = 1:1.6, *E-isomer* 92% *ee*, *Z-isomer* 92% *ee*).

^{1}H NMR (600 MHz, $CDCl_3$) *E-isomer*: δ 7.38 – 7.06 (m, 9H), 5.76 – 5.74 (m, 1H), 5.17 – 5.11 (m, 2H), 3.58 – 3.56 (m, 1H), 2.34 (s, 3H), 2.06 (s, 3H), 1.33 (d, *J* = 7.0 Hz, 3H) ppm. *Z-isomer*: δ 7.38 – 7.06 (m, 9H), 5.49 – 5.47 (m, 1H), 5.17 – 5.11 (m, 2H), 3.26 – 3.22 (m, 1H), 2.34 (s, 3H), 2.03 (s, 3H), 1.18 (d, *J* = 7.0 Hz, 3H) ppm.

^{13}C{^{1}H} NMR (151 MHz, $CDCl_3$) δ 175.2, 174.8, 140.2, 138.8, 138.4, 136.80, 136.79, 136.5, 136.21, 136.19, 128.91, 128.88, 128.51, 128.47, 128.1, 128.0, 127.9, 127.7, 126.1, 125.8, 125.7, 66.2, 66.1, 39.8, 39.6, 29.7, 25.8, 21.1, 21.0, 18.5, 17.9, 16.2 ppm.

See NMR Spectra

HRMS (*m/z*): (ESI) calc'd for $C_{20}H_{22}O_2{}^{23}Na$ $[M+Na]^+$: 317.1512, found: 317.1511.

IR (ATR) ν_{max}: 2922, 2853, 1733, 1454, 1161, 1039, 815, 733 and 697 cm^{-1}.

Chiral HPLC (Chiralpak® IA-3, Hexane/^{i}PrOH = 97:03, 0.5 mL/min) *E-isomer er* = 96.1:3.9, t_R: 10.7 min (major), t_R: 11.3 min (minor); *Z-isomer er* = 96.1:3.9, t_R: 12.9 min (major), t_R = 13.1 min (minor).

See HPLC Spectra

$[\alpha]_D^{24}$ = 61.31 (c = 0.01, CH_2Cl_2).

TLC: R_f = 0.62 (*n*-hexane/ethyl acetate 6:1, v/v)

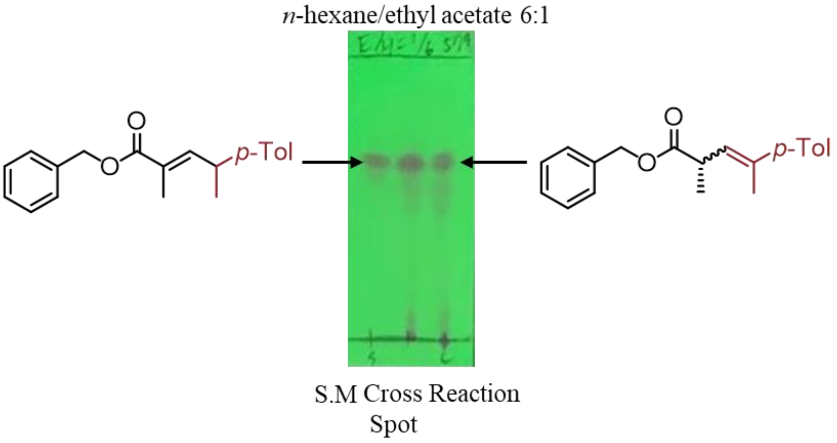

[1,1'-Biphenyl]-4-ylmethyl (*S*)-2-methylpent-3-enoate (2n')

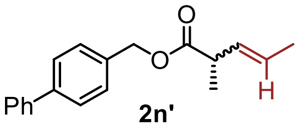

2n'

According to General Procedure E, **1n'** (20 mg, 0.07 mmol, 1.0 equiv) and (*R*)-**D3** (4 mg, 0.007 mmol, 0.1 equiv) were used to obtain the crude residue, which was purified by silica gel column chromatography (*n*-hexane/ethyl acetate 100:1, v/v), to afford **2n'** as a colorless oil (14.0 mg, 0.05 mmol, 70% yield, *E*/*Z* = 1:2.3, *E-isomer* 18% *ee*).

^{1}H NMR (600 MHz, $CDCl_3$) *E-isomer*: δ 7.60 – 7.58 (m, 4H), 7.46 – 7.41 (m, 4H), 7.37 – 7.34 (m, 1H), 5.61 – 5.44 (m, 2H), 5.16 (s, 2H), 3.55 – 3.50 (m, 1H), 1.69 – 1.67 (m, 3H), 1.28 – 1.26 (m, 3H) ppm. *Z-isomer*: δ 7.60 – 7.58 (m, 4H), 7.46 – 7.41 (m, 4H), 7.37 – 7.34 (m, 1H), 5.61 – 5.44 (m, 2H), 5.16 (s, 2H), 3.19 – 3.15 (m, 1H), 1.69 – 1.67 (m, 3H), 1.28 – 1.26 (m, 3H) ppm.

^{13}C{^{1}H} NMR (151 MHz, $CDCl_3$) δ 174.9, 141.09, 141.06, 140.7, 135.2, 135.1, 129.8, 129.4, 128.8, 128.5, 128.4, 127.4, 127.3, 127.1, 127.0, 126.1, 66.0, 42.9, 37.9, 29.7, 17.9, 17.8, 17.4, 13.0 ppm.

See NMR Spectra

HRMS (*m/z*): (ESI) calc'd for $C_{19}H_{20}O_2{}^{23}Na$ $[M+Na]^+$: 303.1356, found: 303.1354.

IR (ATR) ν_{max}: 2973, 2934, 1732, 1488, 1234, 1159, 965, 760 and 697 cm^{-1}.

Chiral HPLC (Chiralpak® OJ-3, Hexane/^{i}PrOH = 95:05, 0.5 mL/min) *E-isomer er* = 58.8:41.2, t_R: 41.2 min (major), t_R: 43.2 min (minor).

Note: *The peaks for Z-isomer were not separatable.*

See HPLC Spectra

$[\alpha]_D^{24}$ = 9.63 (c = 0.01, CH_2Cl_2).

TLC: R_f = 0.41 (*n*-hexane/ethyl acetate 10:1, v/v)

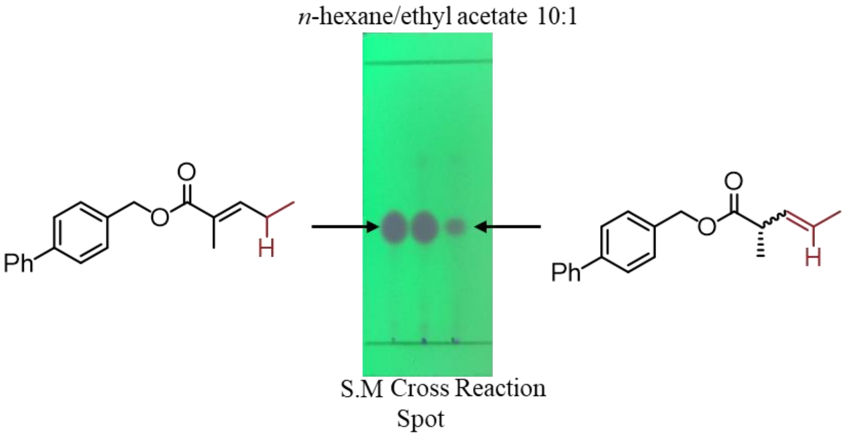

Benzyl (*R*)-2-methyl-4,4-diphenylbut-3-enoate (2o)

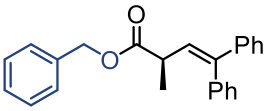

2o

According to General Procedure E, **1o** (21 mg, 0.06 mmol, 1.0 equiv) and (*R*)-**B3** or (*S*)-**B3** (5 mg, 10 mol%) were used to obtain the residue, which was purified by silica gel column chromatography (*n*-hexane/ethyl acetate 100:1, v/v), to afford (*R*)-**2o** as a colorless oil (19 mg, 0.05 mmol, 90% yield, -90% *ee*) or (*S*)-**2o** as a colorless oil (18 mg, 0.05 mmol, 85% yield, 89% *ee*).

^{1}H NMR (400 MHz, $CDCl_3$) δ 7.36 – 7.35 (m, 9H), 7.26 – 7.18(m, 6H), 6.14 (d, J = 10.4 Hz, 1H), 5.14 (m, 2H), 3.38 – 3.33 (m, 1H), 1.14 – 1.10 (m, 3H) ppm.

^{13}C{^{1}H} NMR (101 MHz, $CDCl_3$) δ 168.2, 149.4, 136.4, 128.4, 127.98, 127.96, 125.3, 66.1, 27.9, 21.9, 12.3 ppm.

See NMR Spectra

HRMS (*m/z*): (EI) calc'd for $C_{24}H_{22}O_2$ $[M]^+$: 342.1614, found :342.1617.

IR (ATR) ν_{max}: 3026, 1712, 1494, 1212, 1127, 699 and 513 cm^{-1}.

Chiral HPLC (Chiralpak® IC-3, Hexane/^{i}PrOH = 93:07, 0.7 mL/min) *er* = 5.2:94.8, t_R: 9.6 min (minor), t_R: 10.0 min (major) for (*R*)-**2o**; *er* = 94.3:5.7, t_R: 8.8 min (major), t_R: 9.2 min (minor) for (*S*)-**2o**.

See HPLC Spectra

$[\alpha]_D^{24}$ = -12.92 (c = 0.01, CH_2Cl_2) for (*R*)-**2o**; 12.87 (c = 0.01, CH_2Cl_2) for (*S*)-**2o**.

TLC: R_f = 0.56 (*n*-hexane/ethyl acetate 6:1, v/v)

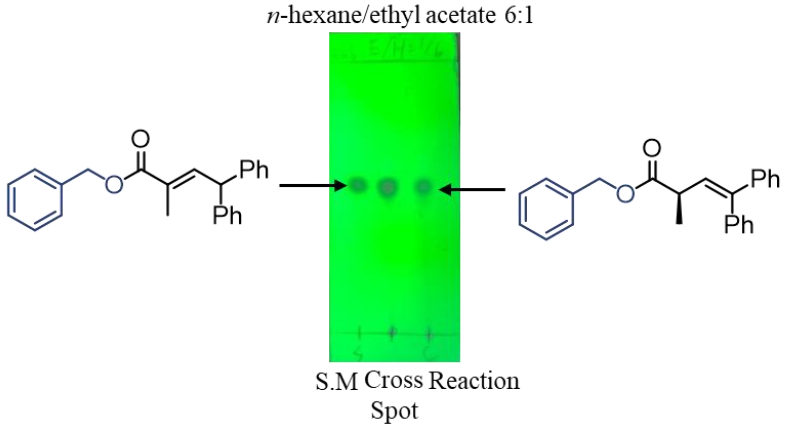

4-Fluorobenzyl (*R*)-2-methyl-4,4-diphenylbut-3-enoate (2p)

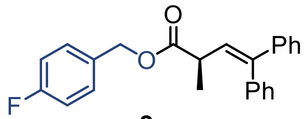

2p

According to General Procedure E, **1p** (22 mg, 0.06 mmol, 1.0 equiv) and (*R*)-**B3** or (*S*)-**B3** (5 mg, 10 mol%) were used to obtain the residue, which was purified by silica gel column chromatography (*n*-hexane/ethyl acetate 100:1, v/v), to afford (*R*)-**2p** as a colorless oil (20 mg, 0.05 mmol, 90% yield, -88% *ee*) or (*S*)-**2p** as a colorless oil (17 mg, 0.05 mmol, 77% yield, 85% *ee*).

^{1}H NMR (400 MHz, $CDCl_3$) δ 7.42 – 7.28 (m, 6H), 7.22 – 7.17 (m, 6H), 7.09 – 6.99 (m, 2H), 6.10 (d, J = 10.1 Hz, 1H), 5.08 (s, 2H), 3.36 – 3.29 (m, 1H), 1.29 (d, J = 6.2 Hz, 3H) ppm.

^{13}C{^{1}H} NMR (101 MHz, $CDCl_3$) δ 174.6, 143.4, 141.8, 139.3, 129.98, 129.90, 129.7, 128.3, 128.1, 127.5, 127.3, 115.5, 115.3, 65.6, 40.4, 30.9, 18.3 ppm.

Note: *The coupling of C and F was not observed.*

^{19}F NMR (282 MHz, $CDCl_3$) δ -113.82 ppm.

See NMR Spectra

HRMS (*m/z*): (EI) calc'd $C_{24}H_{21}O_2F$ for $[M]^+$: 360.1520, found: 360.1521.

IR (ATR) ν_{max}: 3025, 1711, 1511, 1213, 1125, 826, 698 and 488 cm^{-1}.

Chiral HPLC (Chiralpak® IC-3, Hexane/^{i}PrOH = 99:01, 0.7 mL/min) *er* = 5.9:94.1, t_R: 10.0 min (minor), t_R: 10.3 min (major) for (*R*)-**2p**; *er* = 7.4:92.6, t_R: 10.8 min (major), t_R: 12.5 min (minor) for (*S*)-**2p**.

See HPLC Spectra

$[\alpha]_D^{24}$ = -31.48 (c = 0.01, CH_2Cl_2) for (*R*)-**2p**; 31.35 (c = 0.01, CH_2Cl_2) for (*S*)-**2p**.

TLC: R_f = 0.43 (*n*-hexane/ethyl acetate 6:1, v/v)

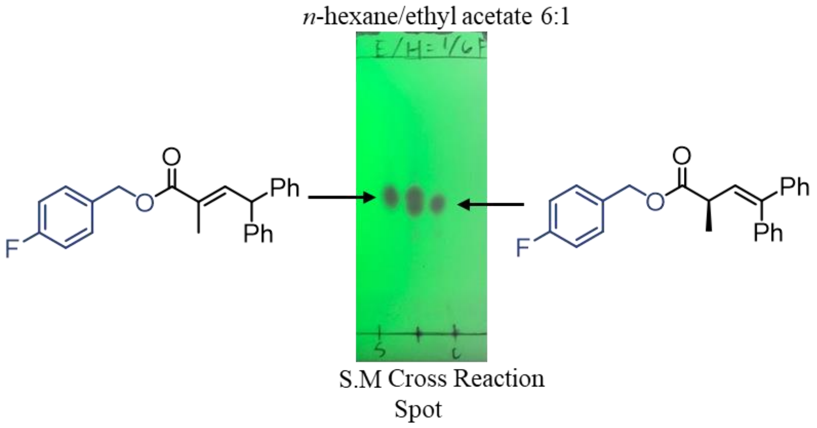

Back to contents / Back to substrate scope table

4-(Trifluoromethyl)benzyl (*R*)-2-methyl-4,4-diphenylbut-3-enoate (2q)

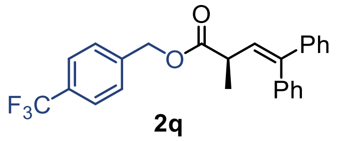

2q

According to General Procedure E, **1q** (22 mg, 0.05 mmol, 1.0 equiv) and (*R*)-**B3** (5 mg, 10 mol%) were used to obtain the residue, which was purified by silica gel column chromatography (*n*-hexane/ethyl acetate 100:1, v/v), to afford **2q** as a colorless oil (16 mg, 0.04 mmol, 73% yield, -90% *ee*).

^{1}H NMR (400 MHz, $CDCl_3$) δ 7.63 – 7.61 (m, 2H), 7.45 – 7.44 (m, 2H), 7.39 – 7.28 (m, 5H), 7.25 – 7.18 (m, 5H), 6.11 (d, *J* = 10.2 Hz, 1H), 5.22 – 5.14 (m, 2H), 3.42 – 3.34 (m, 1H), 1.32 (d, *J* = 7.0 Hz, 3H) ppm.

^{13}C{^{1}H} NMR (101 MHz, $CDCl_3$) δ 167.6, 144.7, 143.2, 129.5, 128.3, 128.1, 126.8, 126.5, 125.5, 87.0, 61.7, 52.4, 25.2 ppm.

Note: *The coupling of C and F was not observed.*

^{19}F NMR (282 MHz, $CDCl_3$) δ -62.67 ppm.

See NMR Spectra

HRMS (*m/z*): (EI) calc'd $C_{25}H_{21}O_2F_3$ for $[M]^+$: 410.1488, found: 410.1485.

IR (ATR) ν_{max}: 2931, 1716, 1324, 1124, 1066, 743 and 699 cm^{-1}.

Chiral HPLC (Chiralpak® IC-3, Hexane/^{i}PrOH = 99:01, 0.7 mL/min) *er* = 4.8:95.2, t_R: 9.6 min (minor), t_R: 9.8 min (major).

See HPLC Spectra

$[\alpha]_D^{24}$ = -20.99 (c = 0.01, CH_2Cl_2).

TLC: R_f = 0.41 (*n*-hexane/ethyl acetate 6:1, v/v)

n-hexane/ethyl acetate 6:1

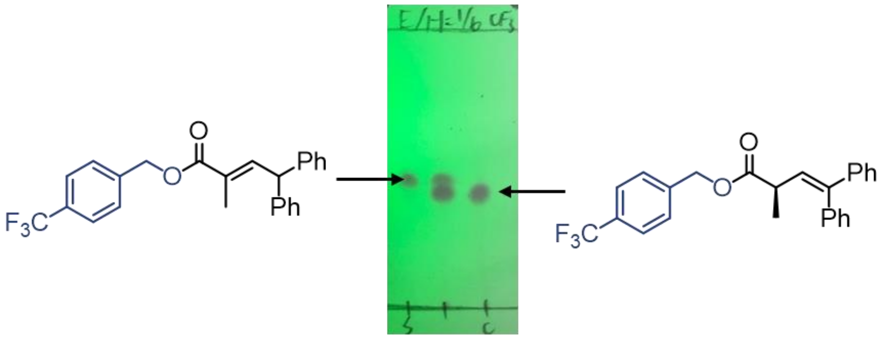

S.M Cross Reaction Spot

4-Methylbenzyl (*R*)-2-methyl-4,4-diphenylbut-3-enoate (2r)

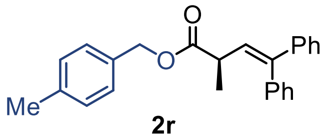

2r

According to General Procedure E, **1r** (20 mg, 0.06 mmol, 1.0 equiv) and (*R*)-**B3** (4.6 mg, 10 mol%) were used to obtain the residue, which was purified by silica gel column chromatography (*n*-hexane/ethyl acetate 100:1, v/v), to afford **2r** as a colorless oil (16 mg, 0.05 mmol, 80% yield, -81% *ee*).

^{1}H NMR (400 MHz, $CDCl_3$) δ 7.37 – 7.17 (m, 14H), 6.13 (d, J = 10.2 Hz, 1H), 5.10 (s, 2H), 3.34 (dq, J = 10.2, 7.0 Hz), 2.37 (s, 3H), 1.30 (d, J = 7.0 Hz, 3H) ppm.

^{13}C{^{1}H} NMR (101 MHz, $CDCl_3$) δ 174.7, 143.2, 141.8, 139.3, 137.9, 129.7, 129.2, 128.3, 128.10, 128.06, 127.7, 127.4, 127.3, 66.3, 40.4, 21.2, 18.4 ppm.

See NMR Spectra

HRMS (*m/z*): (ESI) calc'd $C_{25}H_{24}O_2{}^{23}Na$ for $[M+Na]^+$: 379.1669, found: 379.1667.

IR (ATR) ν_{max}: 3026, 1710, 1448, 1211, 1127, 699 and 478 cm^{-1}.

Chiral HPLC (Chiralpak® IC-3, Hexane/^{i}PrOH = 93:07, 0.7 mL/min) *er* = 9.5:90.5, t_R: 8.9 min (minor), t_R: 9.4 min (major).

See HPLC Spectra

$[\alpha]_D^{24}$ = -11.99 (c = 0.01, CH_2Cl_2).

TLC: R_f = 0.54 (*n*-hexane/ethyl acetate 5:1, v/v)

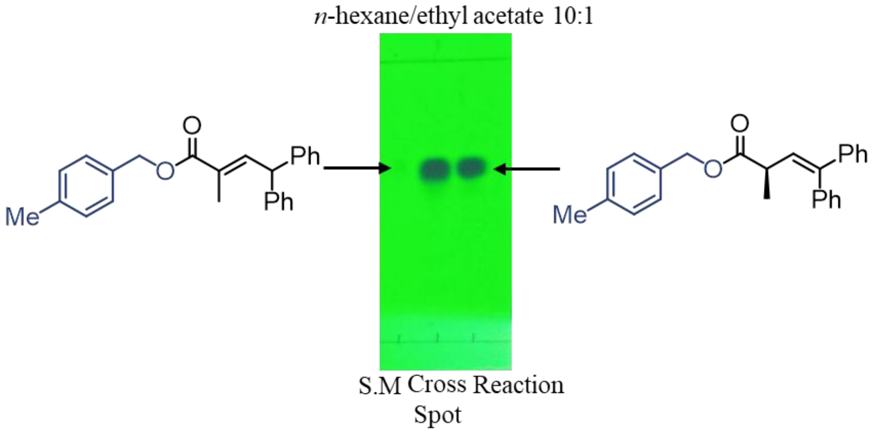

Back to contents / Back to substrate scope table

4-Methoxybenzyl (*R*)-2-methyl-4,4-diphenylbut-3-enoate (2s)

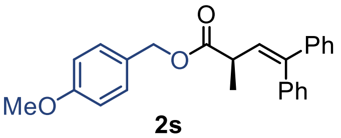

2s

According to General Procedure E, **1s** (16 mg, 0.04 mmol, 1.0 equiv) and (*R*)-**B3** (3 mg, 10 mol%) were used to obtain the residue, which was purified by silica gel column chromatography (*n*-hexane/ethyl acetate 100:1, v/v), to afford **2s** as a colorless oil (14 mg, 0.04 mmol, 88% yield, -84% *ee*).

^{1}H NMR (400 MHz, $CDCl_3$) δ 7.37 – 7.27 (m, 5H), 7.25 – 7.21 (m, 5H), 7.18 – 7.16 (m, 2H), 6.89 (d, J = 8.6 Hz, 2H), 6.11 (d, J = 10.2 Hz, 1H), 5.07 (d, J = 1.8 Hz, 2H), 3.81 (s, 3H), 3.35 – 3.27 (m, 1H), 1.28 (d, J = 7.0 Hz, 3H) ppm.

^{13}C{^{1}H} NMR (101 MHz, $CDCl_3$) δ 174.7, 159.5, 143.2, 141.8, 139.3, 129.8, 129.7, 128.3, 128.2, 128.1, 127.7, 127.4, 127.3, 113.9, 66.1, 55.3, 40.4, 18.4 ppm.

See NMR Spectra

HRMS (*m/z*): (ESI) calc'd $C_{25}H_{24}O_3{}^{23}Na$ for $[M+Na]^+$: 395.1618, found: 391.1617.

IR (ATR) ν_{max}: 2934, 1734, 1269, 1154, 867, 765 and 696 cm^{-1}.

Chiral HPLC (Chiralpak® IC-3, Hexane/^{i}PrOH = 93:07, 0.7 mL/min) *er* = 8.2:91.8, t_R: 10.4 min (minor), t_R: 10.8 min (major).

See HPLC Spectra

$[\alpha]_D^{24}$ = -34.82 (c = 0.01, CH_2Cl_2).

TLC: R_f = 0.47 (*n*-hexane/ethyl acetate 10:1, v/v)

n-hexane/ethyl acetate 10:1

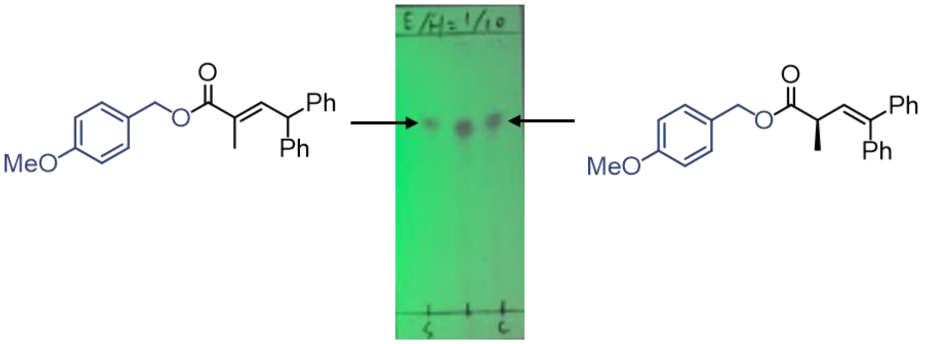

S.M Cross Reaction
Spot

3-Methoxybenzyl (*R*)-2-methyl-4,4-diphenylbut-3-enoate (2t)

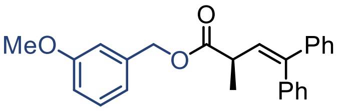

2t

According to General Procedure E, **1t** (20 mg, 0.05 mmol, 1.0 equiv) and (*R*)-**B3** (4 mg, 10 mol%) were used to obtain the residue, which was purified by silica gel column chromatography (*n*-hexane/ethyl acetate 100:1, v/v), to afford **2t** as a colorless oil (15 mg, 0.04 mmol, 73% yield, -85% *ee*).

^{1}H NMR (400 MHz, $CDCl_3$) δ 7.38 – 7.19 (m, 11H), 6.94 – 6.86 (m, 3H), 6.14 (d, J = 10.0 Hz, 1H), 5.12 (s, 2H), 3.78 (s, 3H), 3.78 – 3.33 (m, 1H), 1.32 (d, J = 6.1 Hz, 3H) ppm.

^{13}C{^{1}H} NMR (101 MHz, $CDCl_3$) δ 174.6, 159.7, 143.3, 141.8, 139.3, 137.6, 129.7, 129.6, 128.3, 128.1, 127.6, 127.4, 127.4, 120.0, 113.7, 113.1, 66.1, 55.2, 40.4, 18.4 ppm.

See NMR Spectra

HRMS (*m/z*): (ESI) calc'd $C_{25}H_{24}O_3{}^{23}Na$ for $[M+Na]^+$: 395.1618, found: 391.1616.

IR (ATR) ν_{max}: 2933, 1733, 1491, 1269, 1157, 765 and 700 cm^{-1}.

Chiral HPLC (Chiralpak® IC-3, Hexane/^{i}PrOH = 93:07, 0.7 mL/min) *er* = 7.6:92.4, t_R: 10.3 min (minor), t_R: 11.0 min (major).

See HPLC Spectra

$[\alpha]_D^{24}$ = -34.80 (c = 0.01, CH_2Cl_2).

TLC: R_f = 0.22 (*n*-hexane/ethyl acetate 10:1, v/v)

n-hexane/ethyl acetate 20:1

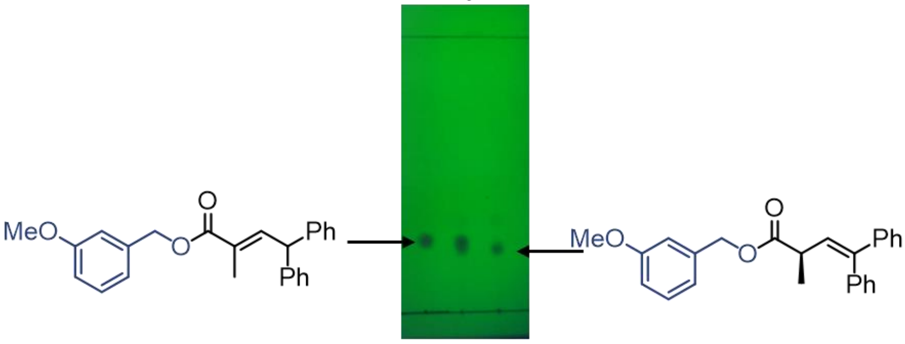

S.M Cross Reaction Spot

Methyl (*R*)-2-methyl-4,4-diphenylbut-3-enoate (2u)

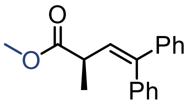

2u

According to General Procedure E, **1u** (22 mg, 0.08 mmol, 1.0 equiv) and (*R*)-**B3** (6 mg, 10 mol%) were used to obtain the residue, which was purified by silica gel column chromatography (*n*-hexane/ethyl acetate, v/v), to afford **2u** as a colorless oil (20 mg, 0.07 mmol, 91% yield, -70% *ee*).

^{1}H NMR (300 MHz, $CDCl_3$) δ 7.45 – 7.28 (m, 4H), 7.26 – 7.14 (m, 6H), 6.12 (d, J = 10.2 Hz, 1H), 3.71 (s, 3H), 3.38 – 3.22 (m, 1H), 1.27 (d, J = 7.0 Hz, 3H) ppm.

^{13}C{^{1}H} NMR (101 MHz, $CDCl_3$) δ 175.4, 143.0, 141.8, 139.3, 129.7, 128.3, 128.1, 127.7, 127.3, 51.9, 40.3, 18.5 ppm.

See NMR Spectra

HRMS (*m/z*): (ESI) calc'd $C_{18}H_{18}O_2{}^{23}Na$ for $[M+Na]^+$: 289.1199, found: 289.1199.

IR (ATR) ν_{max}: 2959, 1731, 1444, 1194, 1091, 765 and 699 cm^{-1}.

Chiral HPLC (Chiralpak® IC-3, Hexane/^{i}PrOH = 93:07, 0.7 mL/min) *er* = 15.0:85.0, t_R: 9.4 min (minor), t_R: 9.6 min (major).

See HPLC Spectra

$[\alpha]_D^{24}$ = -6.50 (c = 0.01, CH_2Cl_2).

TLC: R_f = 0.56 (*n*-hexane/ethyl acetate 10:1, v/v)

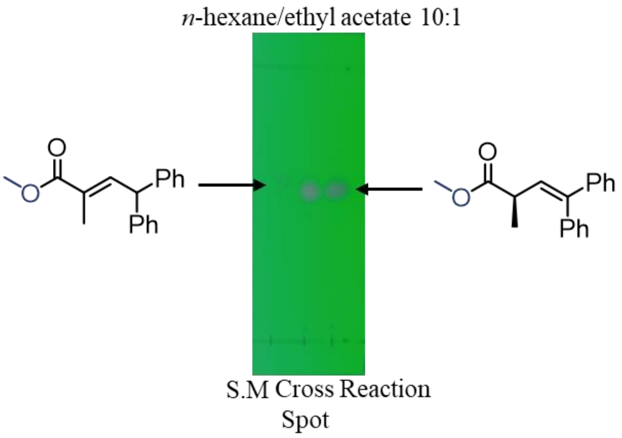

Back to contents / Back to substrate scope table

Ethyl (*R*)-2-methyl-4,4-diphenylbut-3-enoate (2v)

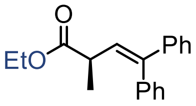

2v

According to General Procedure E, **1v** (14 mg, 0.05 mmol, 1.0 equiv) and (*R*)-**B3** (4 mg, 10 mol%) were used to obtain the residue, which was purified by silica gel column chromatography (*n*-hexane/ethyl acetate 100:1, v/v), to afford **2v** as a yellow oil (13 mg, 0.05 mmol, 97% yield, -85% *ee*).

^{1}H NMR (400 MHz, $CDCl_3$) δ 7.42 – 7.31 (m, 10H), 6.10 (d, J = 10.3 Hz, 1H) 5.21 – 5.07 (m, 2H), 3.29 – 3.07 (m, 1H), 1.27 (s, 3H), 0.90 – 0.83 (m, 3H) ppm.

^{13}C{^{1}H} NMR (101 MHz, $CDCl_3$) δ 174.1, 144.8, 141.9, 140.2, 136.5, 130.7, 128.5, 128.3, 128.1, 128.0, 127.4, 127.34, 127.27, 126.5, 67.0, 47.6, 25.0, 10.0 ppm.

See NMR Spectra

HRMS (*m/z*): (EI) calc'd $C_{19}H_{20}O_2$ for $[M]^+$: 280.1458, found: 280.1455.

IR (ATR) ν_{max}: 2928, 1711, 1214, 1129, 743, 698 and 504 cm^{-1}.

Chiral HPLC (Chiralpak® IC-3, Hexane/^{i}PrOH = 93:07, 0.7 mL/min) *er* = 7.6:92.4, t_R: 9.3 min (minor), t_R: 9.6 min (major).

See HPLC Spectra

$[\alpha]_D^{24}$ = -8.90 (c = 0.01, CH_2Cl_2).

TLC: R_f = 0.59 (*n*-hexane/ethyl acetate 5:1, v/v)

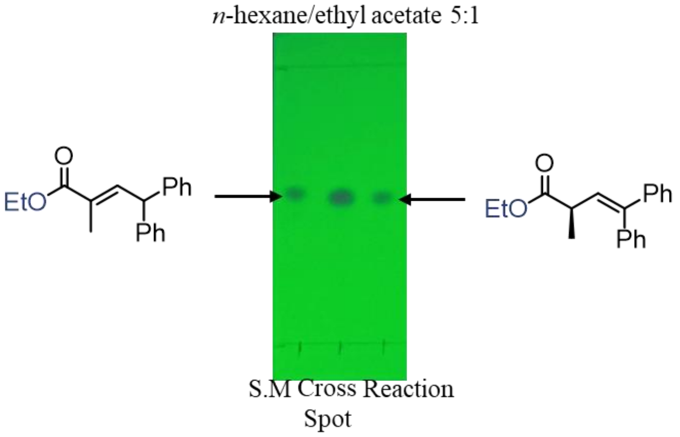

Back to contents / Back to substrate scope table

Isopropyl (*R*)-2-methyl-4,4-diphenylbut-3-enoate (2w)

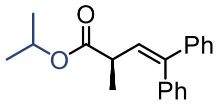

2w

According to General Procedure E, **1w** (19 mg, 0.06 mmol, 1.0 equiv) and (*R*)-**C1** (5 mg, 10 mol%) were used to obtain the residue, which was purified by silica gel column chromatography (*n*-hexane/ethyl acetate 100:1, v/v), to afford **2w** as a colorless oil (18 mg, 0.06 mmol, 96% yield, -91% *ee*).

^{1}H NMR (300 MHz, $CDCl_3$) δ 7.43 – 7.26 (m, 5H), 7.24 – 7.16 (m, 5H), 6.11 (d, J = 10.5 Hz, 1H), 5.06 – 4.93 (m, 1H), 3.29 – 3.15 (m, 1H), 1.27 – 1.19 (m, 9H) ppm.

^{13}C{^{1}H} NMR (101 MHz, $CDCl_3$) δ 174.4, 142.9, 142.0, 139.5, 129.8, 128.3, 128.1, 127.4, 67.8, 40.6, 21.7, 18.4 ppm.

See NMR Spectra

HRMS (*m/z*): (ESI) calc'd $C_{20}H_{22}O_2{}^{23}Na$ for $[M+Na]^+$: 317.1512, found 317.1512.

IR (ATR) ν_{max}: 2984, 2934, 1704, 1452, 1382, 699 and 498 cm^{-1}.

Chiral HPLC (Chiralpak® IC-3, Hexane/^{i}PrOH = 93:07, 0.7 mL/min) *er* = 4.3:95.7, t_R: 9.2 min (minor), t_R: 9.4 min (major).

See HPLC Spectra

$[\alpha]_D^{24}$ = -11.60 (c = 0.01, CH_2Cl_2).

TLC: R_f = 0.41 (*n*-hexane/ethyl acetate 10:1, v/v)

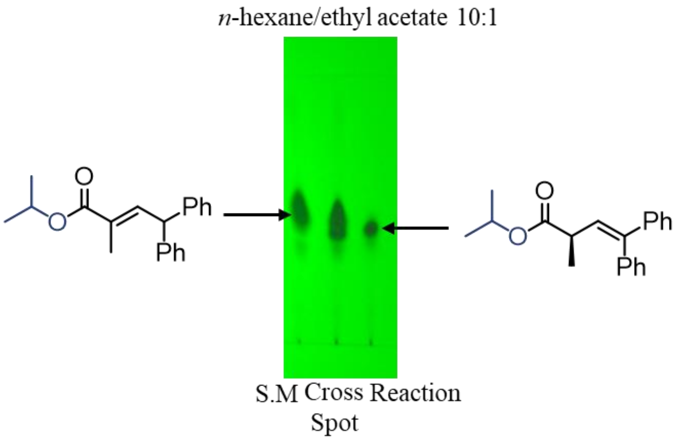

tert-Butyl (R)-2-methyl-4,4-diphenylbut-3-enoate (2x)

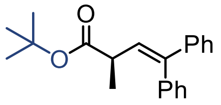

2x

According to General Procedure E, **1x** (20 mg, 0.06 mmol, 1.0 equiv) and (*R*)-**B3** (5 mg, 10 mol%) were used to obtain the residue, which was purified by silica gel column chromatography (*n*-hexane/ethyl acetate 100:1, v/v), to afford **2x** as a colorless oil (20 mg, 0.06 mmol, 98% yield, -96% *ee*).

^{1}H NMR (300 MHz, $CDCl_3$) δ 7.40 – 7.35 (m, 4H), 7.25 (s, 6H), 6.10 (d, J = 10.1 Hz, 1H), 3.21 – 3.15 (m, 1H), 1.46 (s, 9H), 1.24 (d, J = 6.6 Hz, 3H) ppm.

^{13}C{^{1}H} NMR (101 MHz, $CDCl_3$) δ 167.4, 143.9, 140.4, 129.2, 128.5, 128.4, 126.3, 49.3, 28.2, 21.3 ppm.

See NMR Spectra

HRMS (*m/z*): (EI) calc'd $C_{21}H_{24}O_2$ for $[M]^+$: 308.1771, found 308.1770.

IR (ATR) ν_{max}: 2984, 2934, 1704, 1452, 1382, 699 and 498 cm^{-1}.

Chiral HPLC (Chiralpak® IC-3, Hexane/^{i}PrOH = 99:01, 0.8 mL/min) *er* = 1.8:98.2, t_R: 7.5 min (minor), t_R: 7.7 min (major).

See HPLC Spectra

$[\alpha]_D^{24}$ = -16.49 (c = 0.01, CH_2Cl_2).

TLC: R_f = 0.83 (*n*-hexane/ethyl acetate 10:1, v/v)

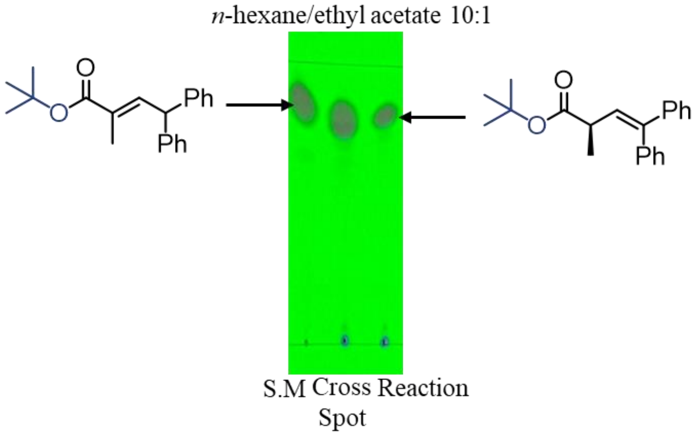

Back to contents / Back to substrate scope table

(1*R*,3*R*,5*R*,7*R*)-Adamantan-2-yl (*R*)-2-methyl-4,4-diphenylbut-3-enoate (2y)

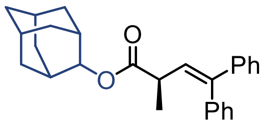

2y

According to General Procedure E, **1y** (21 mg, 0.05 mmol, 1.0 equiv) and (*R*)-**B3** (5 mg, 10 mol%) were used to obtain the residue, which was purified by silica gel column chromatography (*n*-hexane/ethyl acetate 100:1, v/v), to afford **2y** as a colorless oil (16 mg, 0.04 mmol, 76% yield, -83% *ee*).

^{1}H NMR (400 MHz, $CDCl_3$) δ 7.41 – 7.31 (m, 4H), 7.26 – 7.21 (m, 6H), 6.12 (d, J = 8.0 Hz, 1H), 4.94 – 4.92 (m, 1H), 3.36 – 3.28 (m, 1H), 2.02 – 1.98 (m, 4H),1.88 – 1.74 (m, 10 H), 1.30 (d, J = 8.0 Hz, 3H) ppm.

$^{13}C\{^1H\}$ NMR (101 MHz, $CDCl_3$) δ 174.3, 143.1, 142.2, 139.7, 129.9, 128.5, 128.4, 128.3, 127.6, 127.5, 40.4, 37.5, 36.4, 32.0, 31.9, 27.4, 27.1, 18.5 ppm.

See NMR Spectra

HRMS (*m/z*): (ESI) calc'd $C_{27}H_{30}O_2{}^{23}Na$ for $[M+Na]^+$: 409.2138, found 409.2137.

IR (ATR) ν_{max}: 3922, 2852, 1722, 1458, 1198, 701 and 498 cm^{-1}.

Chiral HPLC (Chiralpak® IC-3, Hexane, 1.8 mL/min) *er* = 8.6:91.4, t_R: 9.3 min (minor), t_R: 9.7 min (major).

See HPLC Spectra

$[\alpha]_D^{24}$ = -17.99 (c = 0.01, CH_2Cl_2).

TLC: R_f = 0.95 (*n*-hexane/ethyl acetate 10:1, v/v)

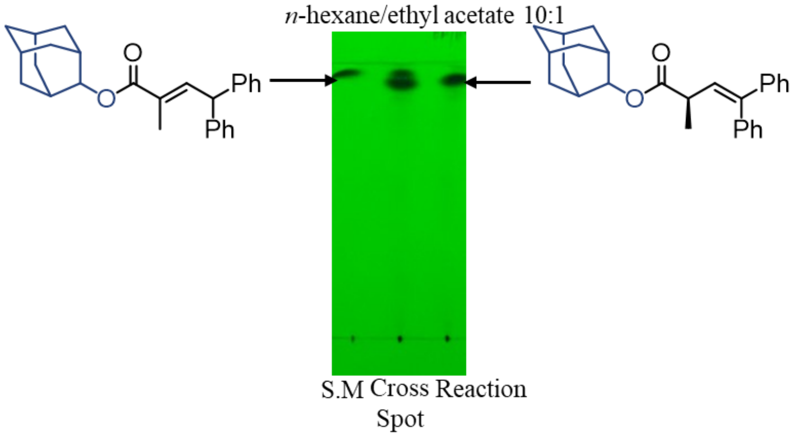

Naphthalen-2-ylmethyl (*R*)-2-methyl-4,4-diphenylbut-3-enoate (2z)

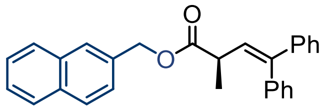

2z

According to General Procedure E, **1z** (21 mg, 0.05 mmol, 1.0 equiv) and (*R*)-**B3** (5 mg, 10 mol%) were used to obtain the residue, which was purified by silica gel column chromatography (*n*-hexane/ethyl acetate 100:1, v/v), to afford **2z** as a colorless oil (16 mg, 0.04 mmol, 76% yield, -83% *ee*).

^{1}H NMR (400 MHz, $CDCl_3$) δ 7.82 – 7.77 (m, 5H), 7.47 – 7.40 (m, 3H), 7.31 – 7.15 (m, 9H), 6.11 (d, *J* = 10.3 Hz, 1H), 5.27 (m, 2H), 3.37 – 3.31 (m, 1H), 1.29 (d, *J* = 7.0 Hz, 3H) ppm.

$^{13}C\{^1H\}$ NMR (101 MHz, $CDCl_3$) δ 174.6, 143.3, 141.8, 139.3, 133.5, 133.2, 133.1, 129.7, 128.3, 128.1, 128.0, 127.4, 126.9, 126.3, 126.2, 125.6, 66.4, 40.5, 18.4 ppm.

See NMR Spectra

HRMS (*m/z*): (ESI) calc'd $C_{28}H_{24}O_2{}^{23}Na$ for $[M+Na]^+$: 415.1669, found: 415.1670.

IR (ATR) ν_{max}: 2931, 1731, 1161, 816, 762, 700 and 473 cm^{-1}.

Chiral HPLC (Chiralpak® IC-3, Hexane/^{i}PrOH = 93:07, 0.7 mL/min) *er* = 8.6:91.4, t_R: 10.0 min (minor), t_R: 10.4 min (major).

See HPLC Spectra

$[\alpha]_D^{24}$ = -17.99 (c = 0.01, CH_2Cl_2).

TLC: R_f = 0.72 (*n*-hexane/ethyl acetate 10:1, v/v)

n-hexane/ethyl acetate 6:1

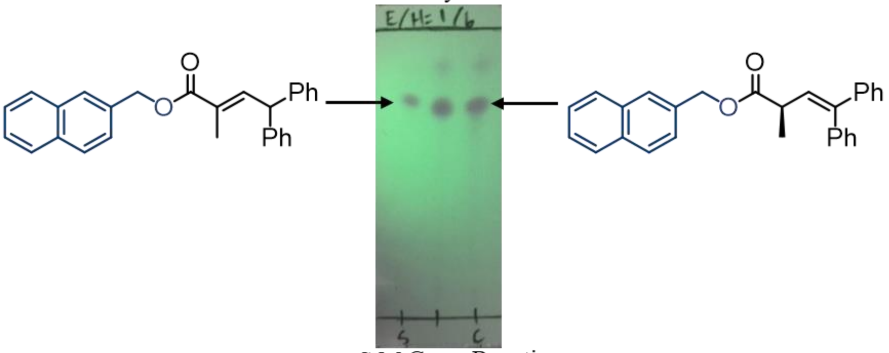

S.M Cross Reaction
Spot

Benzyl (*R*)-2-ethyl-4,4-diphenylbut-3-enoate (2aa)

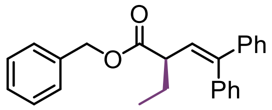

2aa

According to General Procedure E, **1aa** (25 mg, 0.07 mmol, 1.0 equiv) and (*R*)-**B3** (5 mg, 10 mol%) were used to obtain the residue, which was purified by silica gel column chromatography (*n*-hexane/ethyl acetate 100:1, v/v), to afford **2aa** as a yellow oil (19 mg, 0.07 mmol, 93% yield, -83% *ee*).

^{1}H NMR (400 MHz, $CDCl_3$) δ 7.35 – 7.32 (m, 8H), 7.25 – 7.23 (m, 5H), 7.17 – 7.14 (m, 2H), 6.10 (d, J = 10.4 Hz, 1H), 5.18 – 5.09 (m, 2H), 3.20 – 3.14 (m, 1H), 1.86 – 1.79 (m, 1H), 1.69 – 1.62 (m, 1H), 0.87 (t, J = 7.4 Hz, 3H) ppm.

^{13}C{^{1}H} NMR (101 MHz, $CDCl_3$) δ 174.0, 144.3, 141.9, 139.4, 136.1, 129.8, 128.5, 128.3, 128.1, 128.0, 127.4, 127.33, 127.26, 126.5, 66.2, 47.6, 26.4, 11.5 ppm.

See NMR Spectra

HRMS (*m/z*): (EI) calc'd $C_{25}H_{24}O_2$ for $[M]^+$:356.1771, found: 356.1774.

IR (ATR) ν_{max}: 2963, 1709, 1210, 1129, 740, 698 and 518 cm^{-1}.

Chiral HPLC (Chiralpak® IC-3, Hexane/^{i}PrOH = 90:10, 0.4 mL/min) *er* = 8.7:91.3, t_R: 11.6 min (minor), t_R: 12.0 min (major).

See HPLC Spectra

$[\alpha]_D^{24}$ = -59.39 (c = 0.01, CH_2Cl_2).

TLC: R_f = 0.46 (*n*-hexane/ethyl acetate 10:1, v/v)

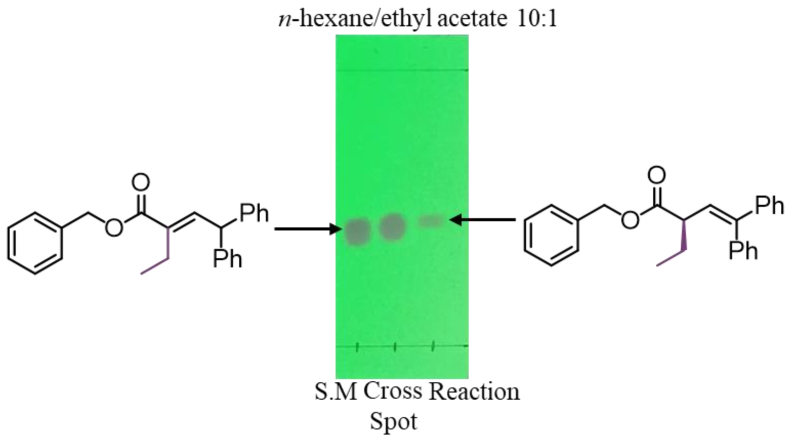

Benzyl (*R*)-2-(2,2-diphenylvinyl)pentanoate (2ab)

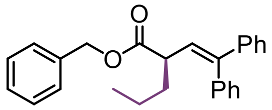

2ab

According to General Procedure E, **1ab** (27 mg, 0.07 mmol, 1.0 equiv) and (*R*)-**B3** (6 mg, 10 mol%) were used to obtain the residue, which was purified by silica gel column chromatography (*n*-hexane/ethyl acetate 100:1, v/v), to afford **2ab** as a yellow oil (21 mg, 0.05 mmol, 78% yield, -83% *ee*).

^{1}H NMR (400 MHz, $CDCl_3$) δ 7.33 (d, J = 9.3 Hz, 8H), 7.24 – 7.23 (m, 5H), 7.15 (d, J = 6.5 Hz, 2H), 6.08 (d, J = 10.4 Hz, 1H), 5.13 (ABq, J = 12.5 Hz, 2H), 3.26 – 3.24 (m, 1H), 1.78 – 1.74 (m, 1H), 1.63 – 1.60 (m, 1H), 1.33 – 1.19 (m, 2H), 0.80 (t, J = 7.3 Hz, 3H) ppm.

^{13}C{^{1}H} NMR (101 MHz, $CDCl_3$) δ 174.2, 144.1, 141.9, 139.4, 136.1, 129.8, 128.5, 128.3, 128.1, 128.0, 127.34, 127.27, 126.7, 66.2, 45.9, 35.2, 20.2, 13.8 ppm.

See NMR Spectra

HRMS (*m/z*): (EI) calc'd $C_{26}H_{26}O_2$ for $[M]^+$: 370.1927, found: 370.1929.

IR (ATR) ν_{max}: 2965, 1730, 1495, 1217, 1155, 698 and 522 cm^{-1}.

Chiral HPLC (Chiralpak® IC-3, Hexane/^{i}PrOH = 93:07, 0.7 mL/min) *er* = 8.7:91.3, t_R: 9.1 min (minor), t_R: 9.3 min (major).

See HPLC Spectra

$[\alpha]_D^{24}$ = -83.91 (c = 0.01, CH_2Cl_2).

TLC: R_f = 0.60 (*n*-hexane/ethyl acetate 10:1, v/v)

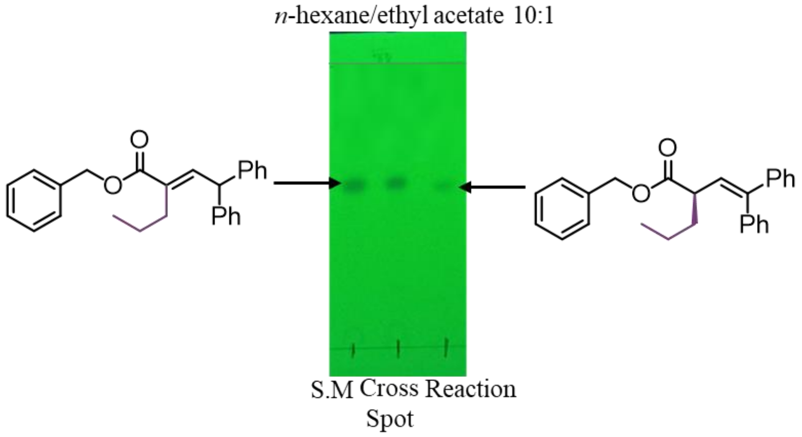

Benzyl (*R*)-2-(2,2-diphenylvinyl)hexanoate (2ac)

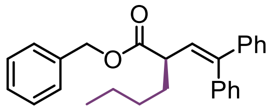

2ac

According to General Procedure E, **1ac** (20 mg, 0.05 mmol, 1.0 equiv) and (*R*)-**B3** (4 mg, 10 mol%) were used to obtain the residue, which was purified by silica gel column chromatography (*n*-hexane/ethyl acetate 100:1, v/v), to afford **2ac** as a yellow oil (19 mg, 0.05 mmol, 95% yield, -81% *ee*).

^{1}H NMR (400 MHz, $CDCl_3$) δ 7.33 (d, J = 8.3 Hz, 8H), 7.24 – 7.23 (m, 5H), 7.15 (d, J = 7.5 Hz, 2H), 6.09 (d, J = 10.4 Hz, 1H), 5.18 – 5.09 (m, 2H), 3.26 – 3.20 (m, 1H), 1.81 – 1.77 (m, 1H), 1.64 – 1.59 (m, 1H), 1.26 – 1.17 (m, 4H), 0.81 (t, J = 6.9 Hz, 3H) ppm.

^{13}C{^{1}H} NMR (101 MHz, $CDCl_3$) δ 174.2, 144.1, 141.9, 139.4, 136.1, 129.8, 128.5, 128.2, 128.1, 128.0, 127.4, 127.33, 127.26, 126.7, 66.2, 46.1, 32.8, 29.1, 22.4, 13.8 ppm.

See NMR Spectra

HRMS (*m/z*): (EI) calc'd $C_{27}H_{28}O_2$ for $[M]^+$: 384.2084, found: 384.2085.

IR (ATR) ν_{max}: 2938, 2870, 1670, 1605, 1180, 705 and 548 cm^{-1}.

Chiral HPLC (Chiralpak® IC-3, Hexane/^{i}PrOH = 93:07, 0.7 mL/min) *er* = 9.7:90.3, t_R: 8.9 min (minor), t_R: 9.1 min (major).

See HPLC Spectra

$[\alpha]_D^{24}$ = -85.25 (c = 0.01, CH_2Cl_2).

TLC: R_f = 0.57 (*n*-hexane/ethyl acetate 10:1, v/v)

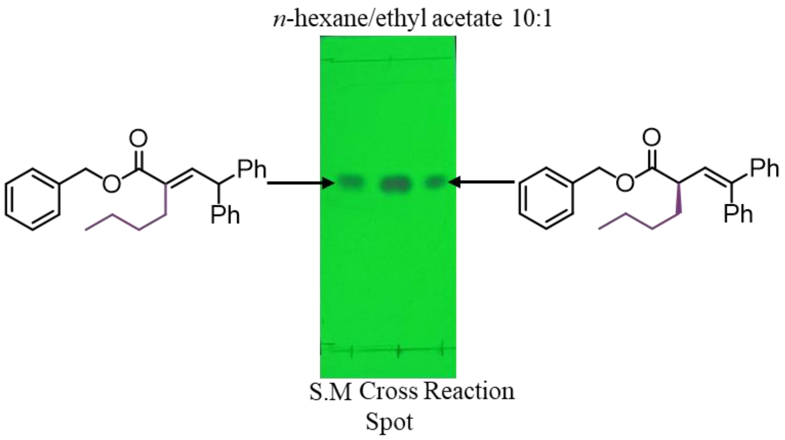

Benzyl (*R*)-2-(2,2-diphenylvinyl)pent-4-enoate (2ad)

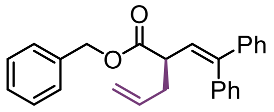

2ad

According to General Procedure E, **1ad** (24 mg, 0.07 mmol, 1.0 equiv) and (*R*)-**B3** (5 mg, 10 mol%) were used to obtain the residue, which was purified by silica gel column chromatography (*n*-hexane/ethyl acetate 100:1, v/v), to afford **2ad** as a yellow oil (17 mg, 0.05 mmol, 73% yield, -81% *ee*).

^{1}H NMR (400 MHz, $CDCl_3$) δ 7.34 – 7.32 (m, 10H), 7.25 – 7.14 (m, 5H), 6.09 (d, J = 10.3 Hz, 1H), 5.70 – 5.60 (m, 1H), 5.17 – 5.12 (m, 2H), 5.09 – 4.97 (m, 2H), 3.37 – 3.31 (m, 1H), 2.56 – 2.36 (m, 2H) ppm.

^{13}C{^{1}H} NMR (101 MHz, $CDCl_3$) δ 173.4, 144.5, 141.8, 139.3, 136.0, 134.6, 129.8, 128.5, 128.3, 128.14, 128.12, 128.07, 127.5, 127.40, 127.36, 125.8, 117.2, 66.3, 45.9, 37.4 ppm.

See NMR Spectra

HRMS (*m/z*): (ESI) calc'd $C_{26}H_{24}O_2{}^{23}Na$ for $[M]^+$: 391.1669, found: 391.1668.

IR (ATR) ν_{max}: 2926, 1733, 1325, 1154, 918, 697 and 513 cm^{-1}.

Chiral HPLC (Chiralpak® IC-3, Hexane/^{i}PrOH = 95:05, 0.5 mL/min) *er* = 9.4:90.6, t_R: 9.1 min (minor), t_R: 9.3 min (major).

See HPLC Spectra

$[\alpha]_D^{24}$ = -79.83 (c = 0.01, CH_2Cl_2).

TLC: R_f = 0.48 (*n*-hexane/ethyl acetate 10:1, v/v)

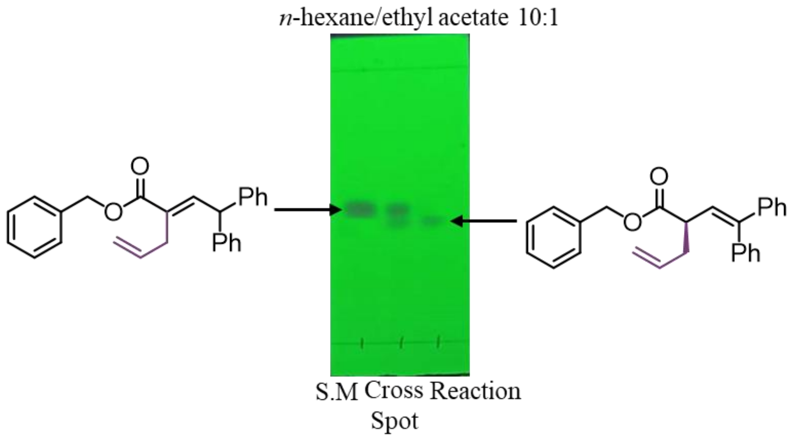

Benzyl (*R*)-2-(2,2-diphenylvinyl)hex-5-enoate (2ae)

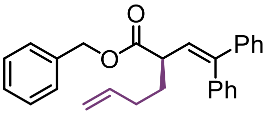

2ae

According to General Procedure E, **1ae** (15 mg, 0.04 mmol, 1.0 equiv) and (*R*)-**B3** (3 mg, 10 mol%) were used to obtain the residue, which was purified by silica gel column chromatography (*n*-hexane/ethyl acetate 100:1, v/v), to afford **2ae** as a yellow oil (11 mg, 0.03 mmol, 71% yield, -72% *ee*).

^{1}H NMR (400 MHz, $CDCl_3$) δ 7.35 – 7.28 (m, 10H), 7.24 – 7.13 (m, 5H), 6.08 (d, J = 10.4 Hz, 1H), 5.71 – 5.64 (m, 1H), 5.15 (q, J = 12.4 Hz, 2H), 4.93 – 4.87 (m, 2H), 3.29 – 3.27 (m, 1H), 1.96 – 1.73 (m, 4H) ppm.

^{13}C{^{1}H} NMR (151 MHz, $CDCl_3$) δ 173.9, 144.5, 141.8, 139.3, 137.5, 136.0, 129.8, 128.51, 128.45, 128.3, 128.1, 128.0, 127.5, 127.4, 127.3, 126.3, 115.1, 66.3, 45.6, 32.3, 31.1 ppm.

See NMR Spectra

HRMS (*m/z*): (ESI) calc'd $C_{27}H_{26}O_2{}^{23}Na$ for $[M+Na]^+$: 405.1825, found: 405.1823.

IR (ATR) ν_{max}: 3072, 2922, 1716, 1496, 1288, 1218 and 513 cm^{-1}.

Chiral HPLC (Chiralpak® IC-3, Hexane/^{i}PrOH = 99:01, 0.6 mL/min) *er* = 86.0:14.0, t_R: 13.1 min (minor), t_R: 13.4 min (major).

See HPLC Spectra

$[\alpha]_D^{24}$ = -62.40 (c = 0.01, CH_2Cl_2).

TLC: R_f = 0.77 (*n*-hexane/ethyl acetate 10:1, v/v)

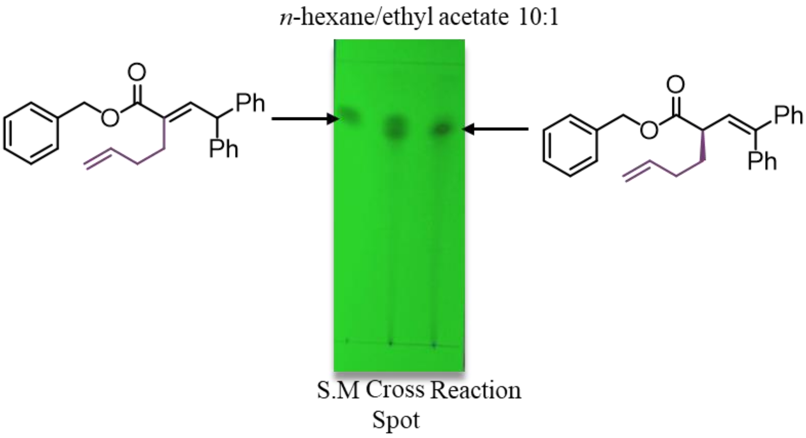

Methyl (*R*)-4-methyl-2-phenylpent-3-enoate (2af)

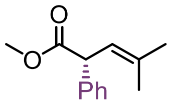

2af

According to General Procedure E, **1af** or (*Z*)-**1af** (10 mg, 0.05 mmol, 1.0 equiv) and (*R*)-**D3** (3 mg, 10 mol%) were used to obtain the residue, which was purified by silica gel column chromatography (*n*-hexane/ethyl acetate 100:1, v/v), to afford **2af** as a colorless oil (6 mg, 0.03 mmol, 64% yield, 78% *ee* from (*E*)-**1af**; 5 mg, 0.02 mmol, 46% yield, 76% *ee* from (*Z*)-**1af**).

^{1}H NMR (600 MHz, $CDCl_3$) δ 7.31 (d, J = 4.4 Hz, 4H), 7.25 – 7.23 (m, 1H), 5.65 – 5.63 (m, 1H), 4.51 (d, J = 9.5 Hz, 1H), 3.68 (s, 3H), 1.77 (s, 3H), 1.68 (d, J = 0.8 Hz, 3H) ppm.

^{13}C{^{1}H} NMR (151 MHz, $CDCl_3$) δ 173.7, 139.5, 135.0, 128.6, 127.7, 127.0, 122.0, 52.1, 50.4, 25.8, 18.1 ppm.

See NMR Spectra

HRMS (*m/z*): (ESI) calc'd $C_{13}H_{16}O_2{}^{23}Na$ for $[M+Na]^+$: 227.1043, found: 227.1041.

IR (ATR) ν_{max}: 2969, 2921, 1736, 1229, 1153, 1066, 731 and 698 cm^{-1}.

Chiral HPLC (Chiralpak® IC-3, Hexane/^{i}PrOH = 99:01, 0.9 mL/min) *er* = 11.1:88.9, t_R: 8.6 min (minor), t_R: 8.8 min (major) from **1af**; *er* = 11.8:88.2, t_R: 8.7 min (minor), t_R: 8.9 min (major) from (*Z*)-**1af**.

See HPLC Spectra

$[\alpha]_D^{24}$ = 8.51 (c = 0.01, CH_2Cl_2).

TLC: R_f = 0.69 (*n*-hexane/ethyl acetate 6:1, v/v)

n-hexane/ethyl acetate 6:1

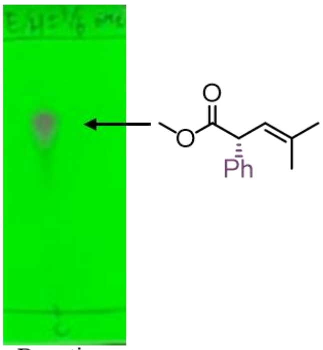

Reaction

[1,1'-Biphenyl]-4-ylmethyl (*R*)-4-methyl-2-phenylpent-3-enoate (2ag)

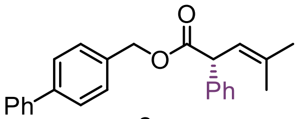

2ag

According to General Procedure E, **1ag** or (*Z*)-**1ag** (20 mg, 0.06 mmol, 1.0 equiv) and (*R*)-**D3** (3 mg, 10 mol%) were used to obtain the residue, which was purified by silica gel column chromatography (*n*-hexane/ethyl acetate 100:1, v/v), to afford **2ag** as a colorless oil (5 mg, 0.01 mmol, 24% yield, 91% *ee* from **1ag**; 7 mg, 0.02 mmol, 33% yield, 90% *ee* from (*Z*)-**1ag**).

^{1}H NMR (600 MHz, $CDCl_3$) δ 7.58 – 7.57 (m, 5H), 7.44 (t, *J* = 7.7 Hz, 3H), 7.34 – 7.31 (m, 6H), 5.69 (d, *J* = 9.4 Hz, 1H), 5.16 (q, *J* = 12.5 Hz, 2H), 4.57 (d, *J* = 9.4 Hz, 1H), 1.77 (s, 3H), 1.68 (s, 3H) ppm.

^{13}C{^{1}H} NMR (151 MHz, $CDCl_3$) δ 173.0, 141.1, 140.7, 139.4, 135.2, 135.0, 128.8, 128.6, 128.4, 127.8, 127.4, 127.2, 127.1, 127.0, 121.9, 66.3, 50.6, 25.8, 18.2 ppm.

See NMR Spectra

HRMS (*m/z*): (ESI) calc'd $C_{25}H_{24}O_2{}^{23}Na$ for $[M+Na]^+$: 379.1669, found: 379.1666.

IR (ATR) ν_{max}: 2960, 1714, 1249, 1139, 824, 761 and 698 cm^{-1}.

Chiral HPLC (Chiralpak® IC-3, Hexane/^{i}PrOH = 93:07, 0.7 mL/min) *er* = 4.7:95.3, t_R: 9.3 min (minor), t_R: 9.7 min (major) form **1ag**; *er* = 4.9:95.1, t_R: 9.4 min (minor), t_R: 9.8 min (major) form (*Z*)-**1ag**.

See HPLC Spectra

$[\alpha]_D^{24}$ = 6.54 (c = 0.01, CH_2Cl_2).

TLC: R_f = 0.68 (*n*-hexane/ethyl acetate 6:1, v/v)

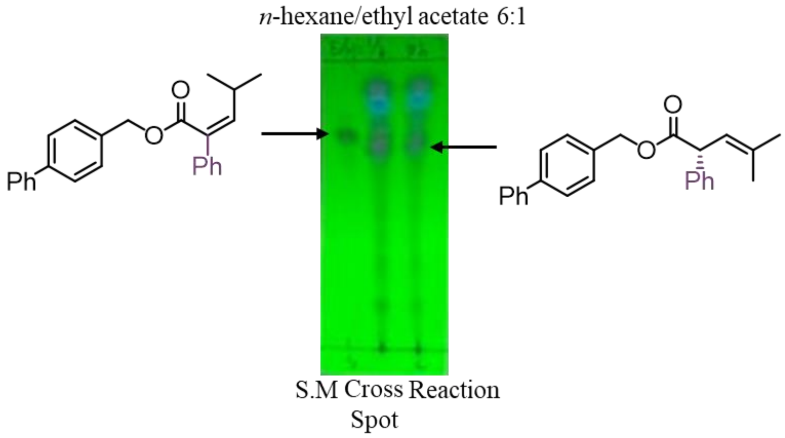

Back to contents / Back to substrate scope table

[1,1'-Biphenyl]-4-ylmethyl (*S*)-2-isopropyl-4-methylpent-3-enoate (2ah)

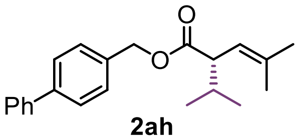

2ah

According to General Procedure E, **1ah** or (*Z*)-**1ah** (20 mg, 0.06 mmol, 1.0 equiv) and (*R*)-**D3** (2 mg, 10 mol%) were used to obtain the residue, which was purified by silica gel column chromatography (*n*-hexane/ethyl acetate 100:1, v/v), to afford **2ah** as a colorless oil (13 mg, 0.04 mmol, 67% yield, 69% *ee* from **1ah**; 13 mg, 0.04 mmol, 65% yield, 77% *ee* from (*Z*)-**1ah**).

^{1}H NMR (600 MHz, $CDCl_3$) δ 7.59 (dd, J = 7.6, 4.0 Hz, 4H), 7.46 – 7.41 (m, 4H), 7.35 (t, J = 7.4 Hz, 1H), 5.19 (d, J = 9.9 Hz, 1H), 5.15 (s, 2H), 3.00 (t, J = 9.4 Hz, 1H), 2.04 – 1.95 (m, 1H), 1.75 (s, 3H), 1.65 (s, 3H), 0.92 (d, J = 6.6 Hz, 3H), 0.87 (d, J = 6.8 Hz, 3H) ppm.

^{13}C{^{1}H} NMR (151 MHz, $CDCl_3$) δ 174.4, 141.0, 140.7, 135.4, 135.3, 128.8, 128.5, 127.4, 127.2, 127.1, 121.7, 65.7, 52.5, 31.5, 25.9, 20.8, 19.7, 18.3 ppm.

See NMR Spectra

HRMS (*m/z*): (ESI) calc'd $C_{22}H_{26}O_2{}^{23}Na$ for $[M+Na]^+$: 345.1825, found: 345.1823.

IR (ATR) ν_{max}: 2958, 2901, 1732, 1385, 908, 732 and 697 cm^{-1}.

Chiral HPLC (Chiralpak® OJ-3, Hexane/^{i}PrOH = 93:07, 0.9 mL/min) *er* = 84.6:15.4, t_R: 13.6 min (major), t_R: 16.6 min (minor) form **1ah**; *er* = 88.4:11.6, t_R: 9.4 min (major), t_R: 9.8 min (minor) form (*Z*)-**1ah**.

See HPLC Spectra

$[\alpha]_D^{24}$ = 7.56 (c = 0.01, CH_2Cl_2).

TLC: R_f = 0.78 (*n*-hexane/ethyl acetate 6:1, v/v)

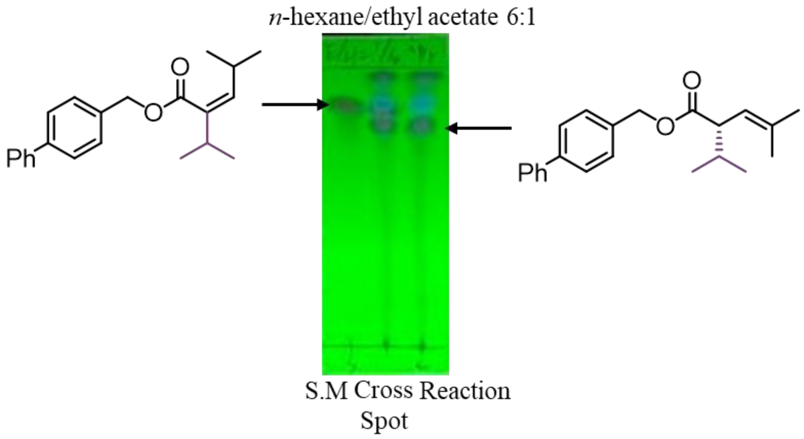

(1*R*,2*S*,5*R*)-2-Isopropyl-5-methylcyclohexyl (*R*)-2-methyl-4,4-diphenylbut-3-enoate (2ai)

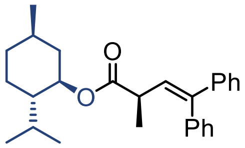

2ai

According to General Procedure E, **1ai** (17 mg, 0.04 mmol, 1.0 equiv) and (*R*)-**B3** (3 mg, 10 mol%) were used to obtain the residue, which was purified by silica gel column chromatography (*n*-hexane/ethyl acetate 100:1, v/v), to afford **2ai** as a colorless oil (14 mg, 0.03 mmol, 82% yield, *dr* = 9:91).

^{1}H NMR (600 MHz, $CDCl_3$) δ 7.39 – 7.27 (m, 5H), 7.24 – 7.18 (m, 5H), 6.10 (dd, J = 19.6, 10.3 Hz, 1H), 4.69 – 4.64 (m, 1H), 3.26 – 3.25 (m, 1H), 2.01 – 1.96 (m, 1H), 1.90 – 1.87 (m, 1H), 1.69 – 1.66 (m, 2H), 1.50 – 1.48 (m, 1H), 1.39 – 1.37 (m, 1H), 1.28 – 1.26 (m, 4H), 1.07 – 1.04 (m, 1H), 0.92 – 0.87 (m, 7H), 0.75 (dd, J = 14.5, 7.0 Hz, 3H) ppm.

^{13}C{^{1}H} NMR (151 MHz, $CDCl_3$) δ 174.4, 143.0, 142.9, 142.0, 139.59, 139.51, 129.8, 129.7, 128.3, 128.12, 128.09, 127.38, 127.35, 127.30, 127.27, 74.3, 47.1, 40.8, 34.3, 31.4, 26.2, 26.1, 23.4, 22.0, 20.8, 18.3, 16.2 ppm.

See NMR Spectra

HRMS (*m/z*): (ESI) calc'd $C_{27}H_{34}O_2{}^{23}Na$ for $[M+Na]^+$: 413.2451, found: 413.2449.

IR (ATR) ν_{max}: 2954, 2926, 2869, 1727, 1445, 1174, 763 and 700 cm^{-1}.

Chiral HPLC (Chiralpak® IC-3, Hexane/^{i}PrOH = 99:01, 0.2 mL/min) *dr* = 9.0:91.0, t_R: 23.6 min (minor), t_R: 24.6 min (major).

See HPLC Spectra

$[\alpha]_D^{24}$ = -50.81 (c = 0.01, CH_2Cl_2).

TLC: R_f = 0.51 (*n*-hexane/ethyl acetate 10:1, v/v)

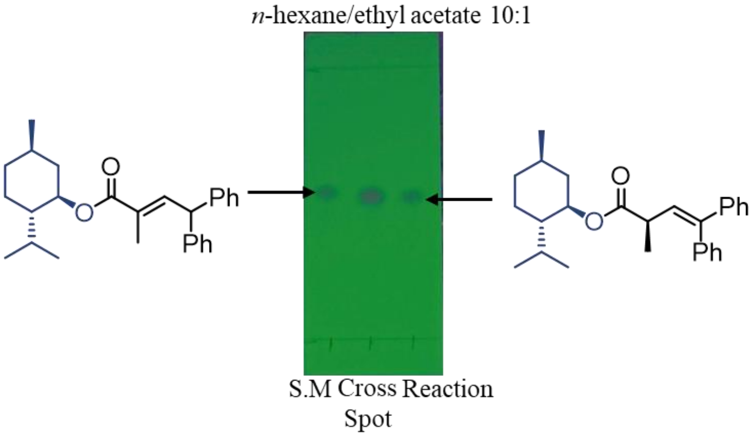

(3*S*,8*S*,9*S*,10*R*,13*R*,14*S*,17*R*)-10,13-Dimethyl-17-((*R*)-6-methylheptan-2-yl)-2,3,4,7,8,9,10,11,12,13,14,15,16,17-tetradecahydro-1*H*-cyclopenta[a]phenanthren-3-yl (*R*)-2-methyl-4,4-diphenylbut-3-enoate (2aj)

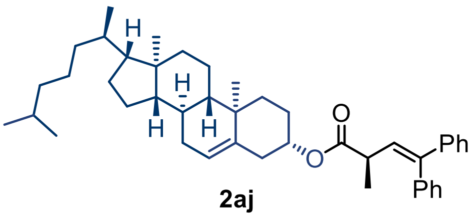

2aj

According to General Procedure E, **1aj** (20 mg, 0.03 mmol, 1.0 equiv) and (*R*)-**B3** (2 mg, 10 mol%) were used to obtain the residue, which was purified by silica gel column chromatography (*n*-hexane/ethyl acetate 100:1, v/v), to afford **2aj** as a colorless oil (14 mg, 0.02 mmol, 70% yield, *dr* = 7:93).

^{1}H NMR (600 MHz, $CDCl_3$) δ 7.40 – 7.27 (m, 5H), 7.26 – 7.20 (m, 5H), 6.11 (d, *J* = 10.2 Hz, 1H), 5.38 (s, 1H), 4.62 – 4.25 (m, 1H), 3.25 – 3.22 (m, 1H), 2.32 – 2.30 (m, 2H), 2.03 – 1.96 (m, 3H), 1.85 – 1.84 (m, 3H), 1.54 – 1.44 (m, 5H), 1.27 (s, 3H), 1.26 (s, 9H), 1.17 – 1.05 (m, 6H), 1.02 (s, 3H), 0.92 (d, *J* = 6.5 Hz, 3H), 0.87 (d, *J* = 2.6 Hz, 3H), 0.86 (d, *J* = 2.7 Hz, 3H), 0.68 (s, 3H) ppm.

^{13}C{^{1}H} NMR (151 MHz, $CDCl_3$) δ 174.3, 142.9, 142.0, 139.5, 129.8, 128.3, 128.11, 128.06, 127.4, 127.31, 127.27, 122.6, 74.1, 56.7, 56.1, 50.0, 42.3, 40.6, 39.7, 39.5, 38.0, 37.0, 36.6, 36.2, 35.8, 31.90, 31.86, 29.7, 29.4, 28.2, 28.0, 27.7, 24.23, 23.8, 22.8, 22.7, 22.5, 21.0, 19.3, 18.7, 18.5, 14.2, 14.1, 11.9 ppm.

See NMR Spectra

HRMS (*m/z*): (ESI) calc'd $C_{44}H_{60}O_2{}^{23}Na$ for $[M+Na]^+$: 643.4486, found: 643.4484.

IR (ATR) ν_{max}: 2923, 2163, 1733, 1376, 1167 and 699 cm^{-1}.

Chiral HPLC (Chiralpak® IC-3, Hexane/^{i}PrOH = 99:01, 0.5 mL/min) *dr* = 7.2:92.8, t_R: 11.0 min (minor), t_R: 13.2 min (major).

See HPLC Spectra

$[\alpha]_D^{24}$ = -61.23 (c = 0.01, CH_2Cl_2).

TLC: R_f = 0.57 (*n*-hexane/ethyl acetate 10:1, v/v)

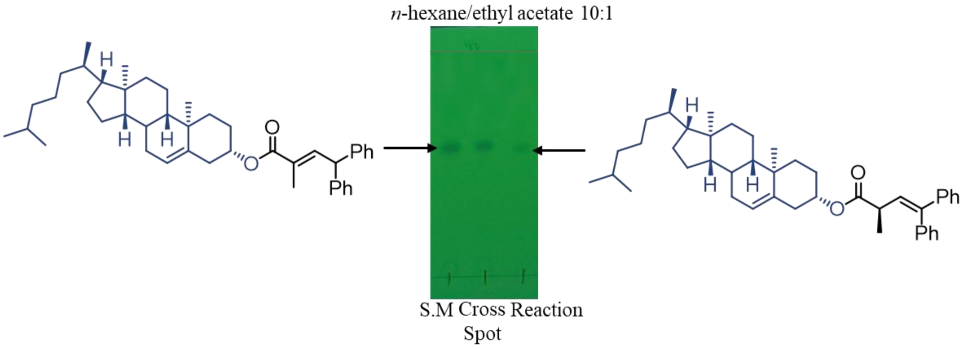

(1*S*,2*R*,4*S*)-1,7,7-Trimethylbicyclo[2.2.1]heptan-2-yl (*R*)-2-methyl-4,4-diphenylbut-3-enoate (2ak)

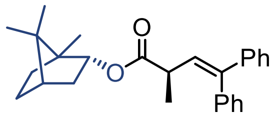

2ak

According to General Procedure E, **1ak** (20 mg, 0.05 mmol, 1.0 equiv) and (*R*)-**B3** (4 mg, 10 mol%) were used to obtain the residue, which was purified by silica gel column chromatography (*n*-hexane/ethyl acetate 100:1, v/v), to afford **2ak** as a colorless oil (7 mg, 0.02 mmol, 37% yield, *dr* = 19:81).

^{1}H NMR (600 MHz, $CDCl_3$) δ 7.40 – 7.27 (m, 5H), 7.25 – 7.22 (m, 5H), 6.09 (dd, J = 10.3, 4.9 Hz, 1H), 4.92 – 4.88 (m, 1H), 3.31 – 3.28 (m, 1H), 2.36 – 2.35 (m, 1H), 1.95 – 1.92 (m, 1H), 1.76 – 1.75 (m, 1H), 1.69 – 1.67 (m, 1H), 1.29 – 1.26 (m, 6H), 0.91 (s, 3H), 0.87 (s, 3H), 0.85 – 0.81 (m, 3H) ppm.

^{13}C{^{1}H} NMR (151 MHz, $CDCl_3$) δ 175.1, 143.0, 142.0, 139.5, 129.77, 129.75, 128.31, 128.30, 128.1, 127.4, 127.3, 80.0, 48.8, 47.9, 44.9, 40.6, 36.9, 36.8, 29.7, 28.1, 27.1, 19.7, 18.8, 18.4, 18.2, 13.6, 13.5 ppm.

See NMR Spectra

HRMS (*m/z*): (ESI) calc'd $C_{27}H_{32}O_2{}^{23}Na$ for $[M+Na]^+$: 411.2295, found: 411.2293.

IR (ATR) ν_{max}: 2954, 2869, 1727, 1495, 1445, 1174, 763 and 700 cm^{-1}.

Chiral HPLC (Chiralpak® IA-3, Hexane/^{i}PrOH = 95:05, 0.5 mL/min) *dr* =18.8:81.2, t_R: 16.5 min (minor), t_R: 22.6 min (major).

See HPLC Spectra

$[\alpha]_D^{24}$ = -49.35 (c = 0.01, CH_2Cl_2).

TLC: R_f = 0.53 (*n*-hexane/ethyl acetate 6:1, v/v)

n-hexane/ethyl acetate 6:1

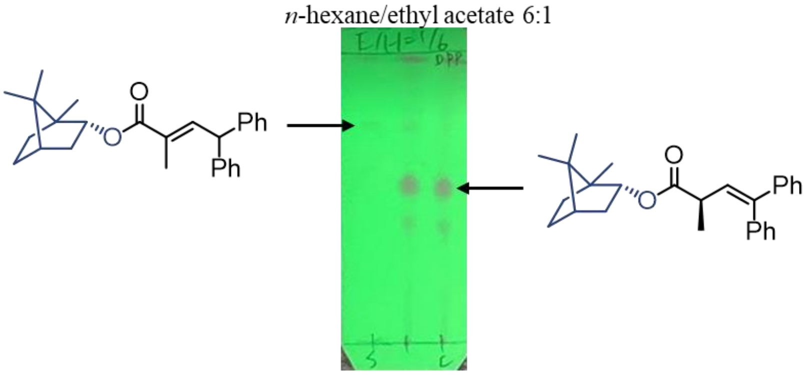

S.M Cross Reaction
Spot

(3a*R*,5*R*,6*S*,6a*R*)-5-((*R*)-2,2-Dimethyl-1,3-dioxolan-4-yl)-2,2-dimethyltetrahydrofuro[2,3-d][1,3]dioxol-6-yl (*R*)-2-methyl-4,4-diphenylbut-3-enoate (2al)

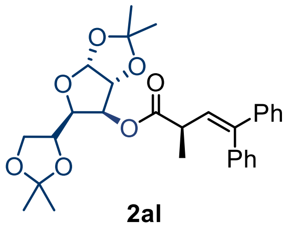

2al

According to General Procedure E, **1al** (30 mg, 0.06 mmol, 1.0 equiv) and (*R*)-**B3** or (*S*)-**B3** (5 mg, 10 mol%) were used to obtain the residue, which was purified by silica gel column chromatography (*n*-hexane/ethyl acetate 100:1, v/v), to afford (*R*)-**2al** as a colorless oil (16 mg, 0.05 mmol, 80% yield, *dr* = 5:95) or (*S*)-**2al** as a colorless oil (12 mg, 0.02 mmol, 40% yield, *dr* = 85:15).

^{1}H NMR (600 MHz, $CDCl_3$) δ 7.40 – 7.33 (m, 4H), 7.25 – 7.18 (m, 6H), 6.07 – 6.05 (m, 1H), 5.84 – 5.84 (m, 1H), 5.30 – 5.29 (m, 1H), 4.42 – 4.42 (m, 1H), 4.21 – 4.17 (m, 2H), 4.00 – 3.97 (m, 2H), 3.33 – 3.30 (m, 1H), 1.52 (s, 3H), 1.39 (d, *J* = 6.2 Hz, 3H), 1.31 – 1.28 (m, 6H), 1.23 (s, 3H) ppm.

^{13}C{^{1}H} NMR (151 MHz, $CDCl_3$) δ 173.2, 143.7, 141.6, 139.3, 129.7, 129.6, 128.4, 128.19, 128.17, 127.54, 127.47, 127.32, 127.29, 127.0, 126.9, 112.3, 109.4, 105.1, 83.3, 80.2, 75.9, 72.3, 67.4, 40.4, 29.7, 26.8, 26.2, 25.1, 17.9 ppm.

See NMR Spectra

HRMS (*m/z*): (ESI) calc'd $C_{29}H_{34}O_7{}^{23}Na$ for $[M+Na]^+$: 517.2197, found: 517.2199.

IR (ATR) ν_{max}: 2956, 2920, 2163, 1456, 1377, 1099, 798 and 614 cm^{-1}.

Chiral HPLC (Chiralpak® IC-3, Hexane/^{i}PrOH = 99:01, 0.3 mL/min) *dr* = 95.1:4.9, t_R: 21.3 min (major), t_R: 21.9 min (minor) for (*R*)-**2ak**; *dr* = 14.8:85.2, t_R: 24.0 min (minor), t_R: 25.0 min (major) for (*S*)-**2ak**.

See HPLC Spectra

$[\alpha]_D^{24}$ = -60.10 (c = 0.01, CH_2Cl_2) for (*R*)-**2ak**; 57.63 (c = 0.01, CH_2Cl_2) for (*S*)-**2ak**.

TLC: R_f = 0.19 (*n*-hexane/ethyl acetate 10:1, v/v)

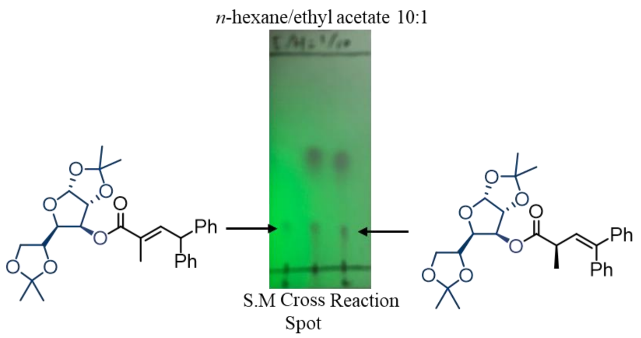

4.3 Characterization data of compound 3

[1,1'-Biphenyl]-4-ylmethyl (*S*)-2,4-dimethylpent-3-enoate-2-*d* ((*S*)-3a)

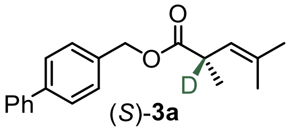

(*S*)-**3a**

According to General Procedure F, **1a** (20 mg, 0.06 mmol, 1.0 equiv) and (*R*)-**D3** (4 mg, 10 mol%) were used to obtain the residue, which was purified by silica gel column chromatography (*n*-hexane/ethyl acetate 100:1, v/v), to afford (*S*)-**3a** as a colorless oil (17 mg, 0.05 mmol, 85% yield, 94% *ee*, 86% D).

^{1}H NMR (400 MHz, $CDCl_3$) δ 7.59 (d, J = 6.8 Hz, 4H), 7.46 – 7.40 (m, 4H), 7.35 (t, J = 7.2 Hz, 1H), 5.17 (d, J = 12.8 Hz, 3H), 3.44 – 3.37 (m, 0.14H), 1.73 (s, 3H), 1.67 (s, 3H), 1.24 (s, 3H) ppm.

^{13}C{^{1}H} NMR (151 MHz, $CDCl_3$) δ 175.3, 141.0, 140.7, 135.3, 134.2, 128.8, 128.3, 127.4, 127.2, 127.1, 123.7, 65.8, 39.0, 38.7 (t, J = 20.4 Hz), 25.7, 17.9 ppm.

See NMR Spectra

HRMS (*m/z*): (ESI) calc'd $C_{20}H_{21}{}^2HO_2{}^{23}Na$ for $[M+Na]^+$: 318.1575, found: 318.1572.

IR (ATR) ν_{max}: 3005, 2253, 1711, 1360, 1221, 912, 731, 648 and 530 cm^{-1}.

Chiral HPLC (Chiralpak® OJ-3, Hexane/^{i}PrOH = 90:10, 0.9 mL/min) *er* = 96.9:3.1, t_R: 15.6 min (major), t_R: 18.6 min (minor).

See HPLC Spectra

$[\alpha]_D^{24}$ = 3.85 (c = 0.01, CH_2Cl_2).

TLC: R_f = 0.83 (*n*-hexane/ethyl acetate 5:1, v/v)

[1,1'-Biphenyl]-4-ylmethyl (*R*)-2,4-dimethylpent-3-enoate-2-*d* ((*R*)-3a)

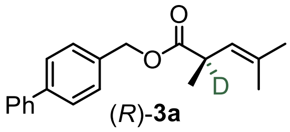

(*R*)-**3a**

According to General Procedure F, **1a** (20 mg, 0.06 mmol, 1.0 equiv) and (*S*)-**D3** (4 mg, 10 mol%) were used to obtain the residue, which was purified by silica gel column chromatography (*n*-hexane/ethyl acetate 100:1, v/v), to afford (*R*)-**3a** as a colorless oil (20 mg, 0.06 mmol, 99% yield, -93% *ee*, 68% D).

^{1}H NMR (400 MHz, $CDCl_3$) δ 7.59 (d, J = 7.4 Hz, 4H), 7.47 – 7.33 (m, 5H), 5.17 (d, J = 9.6 Hz, 3H), 3.45 – 3.35 (m, 0.32H), 1.73 (s, 3H), 1.67 (s, 3H), 1.24 (d, J = 6.0 Hz, 3H) ppm.

^{13}C{^{1}H} NMR (151 MHz, $CDCl_3$) δ 175.3, 141.0, 140.7, 135.3, 134.2, 128.8, 128.3, 127.4, 127.2, 127.1, 123.7, 65.8, 39.0, 38.7 (t, J = 20.4 Hz), 25.7, 17.9 ppm.

See NMR Spectra

HRMS (*m/z*): (ESI) calc'd $C_{20}H_{21}{}^{2}HO_2{}^{23}Na$ for $[M+Na]^+$: 318.1575, found: 318.1572.

IR (ATR) ν_{max}: 3005, 2253, 1711, 1360, 1221, 912, 731, 648 and 530 cm^{-1}.

Chiral HPLC (Chiralpak® OJ-3, Hexane/^{i}PrOH = 90:10, 0.9 mL/min) *er* = 3.6:96.4, t_R: 15.1 min (minor), t_R: 17.7 min (major).

See HPLC Spectra

$[\alpha]_D^{24}$ = -3.80 (c = 0.01, CH_2Cl_2).

TLC: R_f = 0.83 (*n*-hexane/ethyl acetate 5:1, v/v)

[1,1'-Biphenyl]-4-ylmethyl (*S*)-3-cyclobutylidene-2-methylpropanoate-2-*d* (3b)

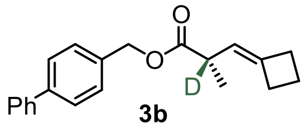

3b

According to General Procedure F, **1d** (20 mg, 0.06 mmol, 1.0 equiv) and (*R*)-**D3** (4 mg, 10 mol%) were used to obtain the residue, which was purified by silica gel column chromatography (*n*-hexane/ethyl acetate 100:1, v/v), to afford **3b** as a colorless oil (16 mg, 0.05 mmol, 81% yield, 75% *ee*, 83% D).

^{1}H NMR (400 MHz, $CDCl_3$) δ 7.59 (d, J = 8.0 Hz, 4H), 7.47 – 7.41 (m, 4H), 7.36 (t, J = 7.3 Hz, 1H), 5.15 (d, J = 10.6 Hz, 3H), 3.20 – 3.12 (m, 0.17H), 2.74 – 2.63 (m, 4H), 1.99 – 1.91 (m, 2H), 1.23 (s, 3H) ppm.

^{13}C{^{1}H} NMR (151 MHz, $CDCl_3$) δ 175.2, 143.1, 141.0, 140.7, 135.3, 128.8, 128.3, 127.4, 127.2, 127.1, 119.2, 65.8, 39.2, 38.8 (t, J = 19.6 Hz), 30.9, 29.3, 17.7, 16.9 ppm.

See NMR Spectra

HRMS (*m/z*): (ESI) calc'd $C_{21}H_{21}{}^{2}HO_{2}{}^{23}Na$ for $[M+Na]^{+}$: 330.1575, found: 330.1572.

IR (ATR) ν_{max}: 2930, 2856, 1707, 1643, 1254, 1103, 761 and 697 cm^{-1}.

Chiral HPLC (Chiralpak® IA-3, Hexane/^{i}PrOH = 99:01, 0.3 mL/min) *er* = 87.4:12.6, t_R: 21.4 min (major), t_R: 22.6 min (minor).

See HPLC Spectra

$[\alpha]_D^{24}$ = 22.19 (c = 0.01, CH_2Cl_2).

TLC: R_f = 0.34 (*n*-hexane/ethyl acetate 10:1, v/v)

[1,1'-Biphenyl]-4-ylmethyl (*S*)-3-cyclopentylidene-2-methylpropanoate-2-*d* (3c)

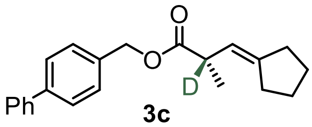

3c

According to General Procedure F, **1e** (20 mg, 0.06 mmol, 1.0 equiv) and (*R*)-**D3** (4 mg, 10 mol%) were used to obtain the residue, which was purified by silica gel column chromatography (*n*-hexane/ethyl acetate 100:1, v/v), to afford **3c** as a colorless oil (15 mg, 0.04 mmol, 74% yield, 91% *ee*, 85% D).

^{1}H NMR (400 MHz, $CDCl_3$) δ 7.59 (dd, J = 7.9, 2.0 Hz, 4H), 7.46 – 7.40 (m, 4H), 7.35 (t, J = 7.3 Hz, 1H), 5.30 (s, 1H), 5.19 – 5.12 (m, 2H), 3.33 – 3.25 (m, 0.15H), 2.27 – 2.17 (m, 4H), 1.68 – 1.59 (m, 4H), 1.25 (d, J = 6.3 Hz, 3H) ppm.

^{13}C{^{1}H} NMR (151 MHz, $CDCl_3$) δ 175.4, 146.1, 141.0, 140.7, 135.3, 128.8, 128.3, 127.4, 127.2, 127.1, 119.1, 65.7, 40.7, 40.3 (t, J = 19.6 Hz), 33.7, 32.4, 29.7, 28.8, 26.2, 17.7 ppm.

See NMR Spectra

HRMS (*m/z*): (ESI) calc'd $C_{22}H_{23}{}^{2}HO_{2}{}^{23}Na$ for $[M+Na]^+$: 344.1731, found: 344.1729.

IR (ATR) ν_{max}: 2932, 2856, 1706, 1643, 1254, 1104, 761 and 697 cm^{-1}.

Chiral HPLC (Chiralpak® IA-3, Hexane/^{i}PrOH = 99:01, 0.3 mL/min) *er* = 95.3:4.7, t_R: 21.0 min (major), t_R: 22.0 min (minor).

See HPLC Spectra

$[\alpha]_D^{24}$ = 34.78 (c = 0.01, CH_2Cl_2).

TLC: R_f = 0.51 (*n*-hexane/ethyl acetate 8:1, v/v)

[1,1'-Biphenyl]-4-ylmethyl (*S*)-2-methyl-3-(tetrahydro-4*H*-pyran-4-ylidene)propanoate-2-*d* (3d)

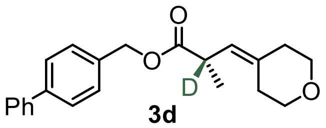

3d

According to General Procedure F, **1g** (20 mg, 0.06 mmol, 1.0 equiv) and (*R*)-**D3** (3 mg, 10 mol%) were used to obtain the residue, which was purified by silica gel column chromatography (*n*-hexane/ethyl acetate 100:1, v/v), to afford **3d** as a colorless oil (15 mg, 0.05 mmol, 75% yield, 95% *ee*, 78% D).

^{1}H NMR (300 MHz, $CDCl_3$) δ 7.60 – 7.57 (m, 4H), 7.47 – 7.33 (m, 5H), 5.25 (s, 1H), 5.20 – 5.11 (m, 2H), 3.73 – 3.54 (m, 4H), 3.46 – 3.41 (m, 0.22H), 2.37 – 2.20 (m, 4H), 1.26 (s, 3H) ppm.

^{13}C{^{1}H} NMR (151 MHz, $CDCl_3$) δ 174.9, 141.2, 140.6, 136.9, 135.1, 128.8, 128.5, 127.4, 127.3, 127.1, 122.3, 69.4, 68.5, 66.0, 37.9, 37.6 (t, J = 20.4 Hz), 36.7, 30.1, 18.1 ppm.

See NMR Spectra

HRMS (*m/z*): (ESI) calc'd $C_{22}H_{23}{}^{2}HO_3{}^{23}Na$ for $[M+Na]^+$: 360.1680, found: 360.1677.

IR (ATR) ν_{max}: 2914, 2849, 1710, 1256, 1016, 762 and 697 cm^{-1}.

Chiral HPLC (Chiralpak® IA-3, Hexane/^{i}PrOH = 99:01, 0.8 mL/min) *er* = 97.4:2.6, t_R: 14.1 min (major), t_R: 15.0 min (minor).

See HPLC Spectra

$[\alpha]_D^{24}$ = 4.41 (c = 0.01, CH_2Cl_2).

TLC: R_f = 0.24 (*n*-hexane/ethyl acetate 10:1, v/v)

tert-Butyl (S)-4-(3-([1,1'-biphenyl]-4-ylmethoxy)-2-methyl-3-oxopropylidene-2-d)piperidine-1-carboxylate (3e)

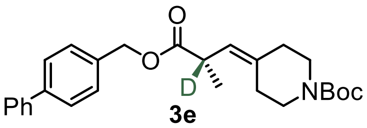

3e

According to General Procedure F, **1h** (20 mg, 0.06 mmol, 1.0 equiv) and (*R*)-**D3** (3 mg, 10 mol%) were used to obtain the residue, which was purified by silica gel column chromatography (*n*-hexane/ethyl acetate 100:1, v/v), to afford **3e** as a colorless oil (13 mg, 0.04 mmol, 65% yield, 95% *ee*, 95% D).

^{1}H NMR (400 MHz, $CDCl_3$) δ 7.58 (d, J = 7.9 Hz, 4H), 7.46 – 7.34 (m, 5H), 5.27 (s, 1H), 5.19 – 5.12 (m, 2H), 3.47 – 3.42 (m, 2.05H), 3.35 – 3.26 (m, 2H), 2.30 – 2.13 (m, 4H), 1.45 (s, 9H), 1.26 (s, 3H) ppm.

^{13}C{^{1}H} NMR (151 MHz, $CDCl_3$) δ 174.9, 154.7, 141.2, 140.6, 137.7, 135.0, 128.8, 128.5, 127.4, 127.3, 127.1, 123.0, 79.5, 66.0, 38.1, 37.8 (t, J = 21.9 Hz), 35.7, 29.7, 28.8, 28.4, 18.1 ppm.

See NMR Spectra

HRMS (*m/z*): (ESI) calc'd $C_{27}H_{32}{}^2HNO_4{}^{23}Na$ for $[M+Na]^+$: 459.2365, found: 459.2362.

IR (ATR) ν_{max}: 2917, 2849, 1733, 1689, 1422, 1240, 1164, 761 and 733 cm^{-1}.

Chiral HPLC (Chiralpak® IC-3, Hexane/^{i}PrOH = 93:07, 0.9 mL/min) *er* = 97.3:2.7, t_R: 25.6 min (major), t_R: 27.2 min (minor).

See HPLC Spectra

$[\alpha]_D^{24}$ = 55.64 (c = 0.01, CH_2Cl_2).

TLC: R_f = 0.56 (*n*-hexane/ethyl acetate 4:1, v/v)

Back to contents / Back to substrate scope table

Benzyl (*S*)-2-methyl-4-phenylpent-3-enoate-2-*d* (3f)

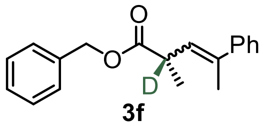

3f

According to General Procedure F, **1k** (20 mg, 0.06 mmol, 1.0 equiv) and (*R*)-**D5** (5 mg, 10 mol%) were used to obtain the residue, which was purified by silica gel column chromatography (*n*-hexane/ethyl acetate 100:1, v/v), to afford **3f** as a colorless oil (16 mg, 0.05 mmol, 79% yield, *E*/*Z* = 1:2.2, *E-isomer* 90% *ee*, 81% D, *Z-isomer* 90% *ee*, 78% D).

^{1}H NMR (400 MHz, $CDCl_3$) *E-isomer*: δ 7.39 – 7.27 (m, 8H), 7.25 – 7.16 (m, 2H), 5.80 (s, 1H), 5.16 (s, 2H), 3.63 – 3.55 (m, 0.19H), 2.09 (m, 3H), 1.34 (s, 3H) ppm. *Z-isomer*: δ 7.39 – 7.27 (m, 8H), 7.25 – 7.16 (m, 2H), 5.53 (s, 1H), 5.12 (s, 2H), 3.27 – 3.19 (m, 0.22H), 2.05 (m, 3H), 1.19 (s, 3H) ppm.

^{13}C{^{1}H} NMR (151 MHz, $CDCl_3$) δ 175.1, 174.7, 143.1, 141.3, 138.9, 137.0, 136.2, 128.52, 128.48, 128.23, 128.20, 128.1, 128.0, 127.9, 127.7, 127.1, 126.9, 126.8, 125.9, 125.8, 66.3, 66.1, 39.8, 39.7, 39.5 (t, *J* = 19.6 Hz), 25.8, 18.4, 17.8 ppm.

See NMR Spectra

HRMS (*m/z*): (ESI) calc'd $C_{19}H_{19}{}^{2}HO_2{}^{23}Na$ for $[M+Na]^+$: 304.1418, found: 304.1417.

IR (ATR) ν_{max}: 2969, 2932, 1732, 1454, 1162, 752 and 699 cm^{-1}.

Chiral HPLC (Chiralpak® IA-3, Hexane/^{i}PrOH = 99:01, 0.7 mL/min) *E-isomer er* = 95.2:4.8, t_R: 12.9 min (major), t_R: 14.2 min (minor), *Z-isomer er* = 95.2:4.8, t_R: 16.8 min (major), t_R: 17.3 min (minor).

See HPLC Spectra

$[\alpha]_D^{24}$ = 59.34 (c = 0.01, CH_2Cl_2).

TLC: R_f = 0.67 (*n*-hexane/ethyl acetate 6:1, v/v)

Back to contents / Back to substrate scope table

Benzyl (*R*)-2-methyl-4,4-diphenylbut-3-enoate-2-*d* (3g)

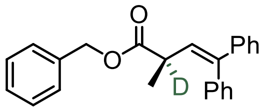

3g

According to General Procedure F, **1o** (21 mg, 0.06 mmol, 1.0 equiv) and (*R*)-**B3** (5 mg, 10 mol%) were used to obtain the residue, which was purified by silica gel column chromatography (*n*-hexane/ethyl acetate 100:1, v/v), to afford **3g** as a colorless oil (17 mg, 0.05 mmol, 82% yield, -93% *ee*, 98% D).

^{1}H NMR (400 MHz, $CDCl_3$) δ 7.36 – 7.27 (m, 9H), 7.25 – 7.16 (m, 6H), 6.12 (s, 1H), 5.17 – 5.10 (m, 2H), 3.38 – 3.30 (m, 0.02H), 1.29 (s, 3H) ppm.

^{13}C{^{1}H} NMR (151 MHz, $CDCl_3$) δ 174.6, 143.3, 141.8, 139.3, 136.1, 129.7, 128.5, 128.3, 128.1, 127.9, 127.5, 127.39, 127.36, 127.3, 66.3, 40.4, 40.1 (t, J = 19.6 Hz), 18.3 ppm.

See NMR Spectra

HRMS (*m/z*): (ESI) calc'd $C_{24}H_{21}{}^{2}HO_{2}{}^{23}Na$ for $[M+Na]^+$: 366.1575, found: 366.1573.

IR (ATR) ν_{max}: 3026, 1712, 1494, 1212, 1127, 699 and 513 cm^{-1}.

Chiral HPLC (Chiralpak® IC-3, Hexane/^{i}PrOH = 93:07, 0.7 mL/min) *er* = 3.5:96.5, t_R: 8.8 min (minor), t_R: 9.2 min (major).

See HPLC Spectra

$[\alpha]_D^{24}$ = -12.87 (c = 0.01, CH_2Cl_2).

TLC: R_f = 0.56 (*n*-hexane/ethyl acetate 6:1, v/v)

4-Methoxybenzyl (*R*)-2-methyl-4,4-diphenylbut-3-enoate-2-*d* (3h)

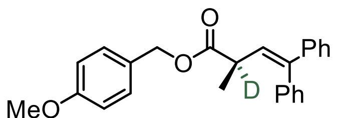

3h

According to General Procedure F, **1s** (16 mg, 0.04 mmol, 1.0 equiv) and (*R*)-**B3** (3 mg, 10 mol%) were used to obtain the residue, which was purified by silica gel column chromatography (*n*-hexane/ethyl acetate 100:1, v/v), to afford **3h** as a colorless oil (15 mg, 0.03 mmol, 77% yield, -92% *ee*, 91% D).

^{1}H NMR (400 MHz, $CDCl_3$) δ 7.35 – 7.27 (m, 5H), 7.24 – 7.15 (m, 7H), 6.88 (d, J = 8.6 Hz, 2H), 6.11 (d, J = 6.4 Hz, 1H), 5.06 (d, J = 2.3 Hz, 2H), 3.81 (s, 3H), 3.34 – 3.27 (m, 0.09H), 1.26 (d, J = 3.3 Hz, 3H) ppm.

^{13}C{^{1}H} NMR (151 MHz, $CDCl_3$) δ 174.7, 159.5, 143.2, 141.8, 139.3, 129.8, 129.7, 128.3, 128.2, 128.1, 127.7, 127.4, 127.3, 113.9, 66.1, 55.3, 40.4, 40.1 (t, J = 19.6 Hz), 18.4 ppm.

See NMR Spectra

HRMS (*m/z*): (ESI) calc'd $C_{20}H_{21}{}^{2}HO_{3}{}^{23}Na$ for $[M+Na]^+$: 334.1632, found: 334.1630.

IR (ATR) ν_{max}: 2934, 1734, 1269, 1154, 867, 765 and 696 cm^{-1}.

Chiral HPLC (Chiralpak® IC-3, Hexane/^{i}PrOH = 93:07, 0.7 mL/min) *er* = 4.0:96.0, t_R: 10.4 min (minor), t_R: 11.3 min (major).

See HPLC Spectra

$[\alpha]_D^{24}$ = -34.81 (c = 0.01, CH_2Cl_2).

TLC: R_f = 0.47 (*n*-hexane/ethyl acetate 10:1, v/v)

Benzyl (*R*)-2-(2,2-diphenylvinyl)pentanoate-2-*d* (3i)

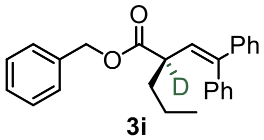

3i

According to General Procedure F, **1w** (27 mg, 0.07 mmol, 1.0 equiv) and (*R*)-**B3** (6 mg, 10 mol%) were used to obtain the residue, which was purified by silica gel column chromatography (*n*-hexane/ethyl acetate 100:1, v/v), to afford **3i** as a colorless oil (20 mg, 0.05 mmol, 75% yield, -82% *ee*, 86% D).

^{1}H NMR (400 MHz, $CDCl_3$) δ 7.36 – 7.27 (m, 10H), 7.25 – 7.15 (m, 5H), 6.07 (d, J = 3.7 Hz, 1H), 5.14 (ABq, J = 12.5 Hz, 2H), 3.28 – 3.22 (m, 0.14H), 1.78 – 1.72 (m, 1H), 1.65 – 1.59 (m, 1H), 1.35 – 1.29 (m, 1H), 1.23 – 1.17 (m, 1H), 0.80 (t, J = 7.3 Hz, 3H) ppm.

^{13}C{^{1}H} NMR (151 MHz, $CDCl_3$) δ 174.2, 144.1, 141.9, 139.4, 136.1, 129.8, 128.5, 128.2, 128.1, 128.0, 127.4, 127.34, 127.27, 126.6, 66.2, 45.9, 45.6 (t, J = 20.4 Hz), 35.2, 20.2, 13.8 ppm.

See NMR Spectra

HRMS (*m/z*): (ESI) calc'd $C_{21}H_{23}{}^{2}HO_2{}^{23}Na$ for $[M+Na]^+$: 332.1839, found: 332.1841.

IR (ATR) ν_{max}: 2965, 1730, 1495, 1217, 1155, 698 and 522 cm^{-1}.

Chiral HPLC (Chiralpak® IC-3, Hexane/^{i}PrOH = 93:07, 0.7 mL/min) *er* = 9.0:91.0, t_R: 8.2 min (minor), t_R: 8.6 min (major).

See HPLC Spectra

$[\alpha]_D^{24}$ = -83.94 (c = 0.01, CH_2Cl_2).

TLC: R_f = 0.60 (*n*-hexane/ethyl acetate 10:1, v/v)

4.4 Characterization data of compound S5

(*E*)-2-Methyl-1,4,4-triphenylbut-2-en-1-one (S5a)

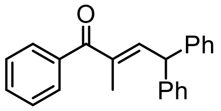

S5a

To a slurry of *N*,*O*-dimethylhydroxylamine hydrochloride (87 mg, 0.89 mmol, 1.2 equiv) and **1v** (200 mg, 0.71 mmol, 1.0 equiv) in 8 mL of THF at -5 °C under nitrogen was added 1.0 M PhMgBr in THF (5.9 mL, 5.92 mmol, 8.3 equiv) over 5 min maintaining the temperature at -5 °C. After putting for l h at -5 °C, the reaction mixture was warmed to room temperature for 20 h. The reaction was added 1.0 M PhMgBr in THF (5.9 mL, 5.92 mmol, 8.3 equiv) in an ice bath, and the reaction mixture was warmed to room temperature for 24 h. After the weinreb amide was totally transferred to phenyl ketone, diluted with EA, and quenched with 1 N HCl in ice bath. The aqueous layer was extracted twice with 10 mL of ethyl acetate, and the combined extracts were dried over Na_2SO_4. The solvent was removed under reduced pressure. The residue was purified by silica gel column chromatography (*n*-hexane/ethyl acetate 100:1, v/v), to afford **S5a** as a colorless oil (124 mg, 0.7 mmol, 56%).

^{1}H NMR (600 MHz, $CDCl_3$) δ 7.85 – 7.83 (m, 2H), 7.57 – 7.53 (m, 1H), 7.40 – 7.37 (m, 2H), 7.27 – 7.23 (m, 4H), 7.20 – 7.16 (m, 2H), 7.06 – 7.04 (m, 4H), 6.17 (d, *J* = 10.9 Hz, 1H), 4.60 (d, *J* = 10.9 Hz, 1H), 2.07 (s, 3H) ppm.

^{13}C{^{1}H} NMR (151 MHz, $CDCl_3$) δ 200.1, 143.2, 136.14, 136.10, 133.5, 132.9, 129.4, 128.6, 128.4, 128.2, 126.4, 50.4, 21.4 ppm.

See NMR Spectra

TLC: R_f = 0.40 (*n*-hexane/ethyl acetate 10:1, v/v)

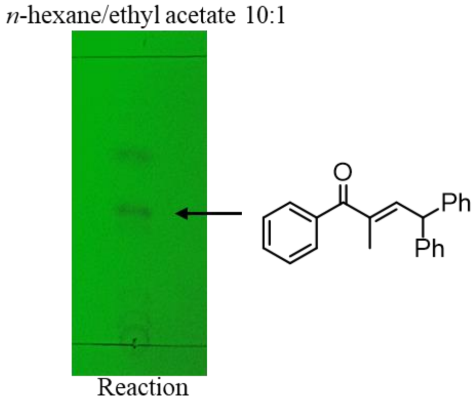

(*S*)-2-Methyl-1,4,4-triphenylbut-3-en-1-one (S5b)

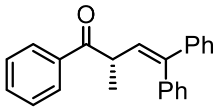

S5b

According to General Procedure E, **S5a** (20 mg, 0.05 mmol, 1.0 equiv) and (*R*)-**D3** (4 mg, 10 mol%) were used to obtain the residue, which was purified by silica gel column chromatography (*n*-hexane/ethyl acetate 100:1, v/v), to afford **S5b** as a yellow oil (10 mg, 0.03 mmol, 48% yield, 58% *ee*).

^{1}H NMR (600 MHz, $CDCl_3$) δ 7.68 – 7.66 (m, 2H), 7.51 – 7.50 (m, 1H), 7.48 – 7.40 (m, 3H), 7.36 – 7.32 (m, 2H), 7.24 – 7.17 (m, 7H), 6.14 (d, J = 10.3 Hz, 1H), 4.32 – 4.24 (dq, J = 13.4, 6.7 Hz, 1H), 1.39 (d, J = 6.7 Hz, 3H) ppm.

^{13}C{^{1}H} NMR (151 MHz, $CDCl_3$) δ 174.2, 144.1, 141.9, 139.4, 136.1, 129.8, 128.5, 128.2, 128.1, 128.0, 127.4, 127.34, 127.27, 126.6, 66.2, 45.9, 45.6 (t, J = 20.4 Hz), 35.2, 20.2, 13.8 ppm.

See NMR Spectra

Chiral HPLC (Chiralpak® OD-3, Hexane/^{i}PrOH = 97:03, 0.9 mL/min) *er* =21.2:78.8, t_R: 7.7 min (minor), t_R: 8.0 min (major).

See HPLC Spectra

TLC: R_f = 0.43 (*n*-hexane/ethyl acetate 10:1, v/v)

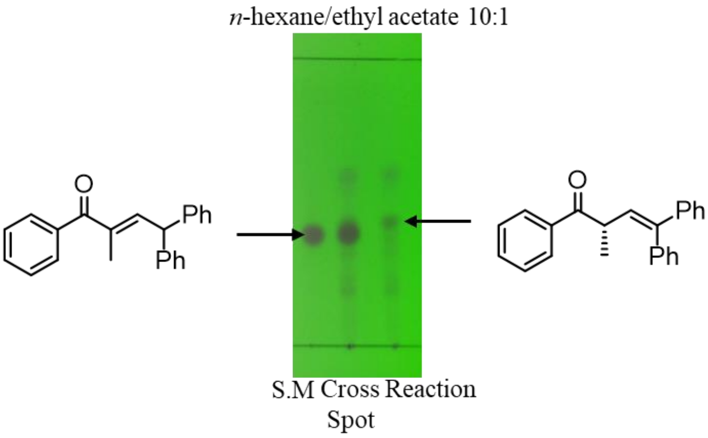

5. Synthetic Application

5.1 Functionalization group interconversion and formal synthesis

Ethyl (*E*)-2-methyl-4-phenylpent-2-enoate (S1k)

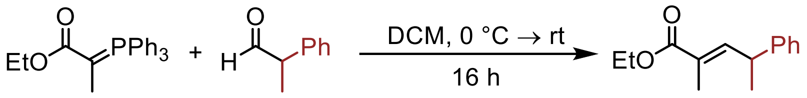

1.0 equiv 3.0 equiv **S1k**

In an oven-dried round bottom flask, ethyl 2-(triphenylphosphoranylidene) propanoate (0.90 g, 2.5 mmol, 1.0 equiv) was dissolved in dry DCM (24.8 mL). 2-Phenylpropanal (1.00 g, 7.5 mmol, 3.0 equiv) was then added to the flask, which was stirred at room temperature for 16 h. The solvent was removed under reduced pressure. The crude product was purified by column chromatography (*n*-hexane/ethyl acetate 50:1, v/v) to obtain the desired product **S1k** as a colorless oil (1.01 g, 4.6 mmol, 62%).

*All characterization data for **S1k**[7] are consistent with the respective reported literature.*

Back to contents

(*R*)-Ethyl 2-methyl-4-phenylpent-3-enoate (4)

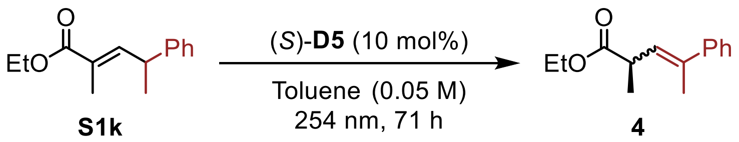

In a quartz tube, **S1k** (86 mg, 0.4 mmol, 1.0 equiv) and (*S*)-**D5** (26 mg, 10 mol%) were dissolved in degassed toluene (7.8 mL) under nitrogen atmosphere. After irradiated at 254 nm for 71 h, toluene was removed under reduced pressure. The crude product was purified by column chromatography (*n*-hexane/ethyl acetate 100:1, v/v) to afford compound **4** as a colorless oil (55 mg, 0.3 mmol, 64% yield, –76% *ee* for *E*-isomer, *E*/*Z* = 1:1.3).

^{1}H NMR (300 MHz, $CDCl_3$) *E-isomer*: δ 7.40 – 7.29 (m, 4H), 7.24 – 7.18 (m, 1H), 5.50 (d, *J* = 9.9 Hz, 1H), 4.19 – 4.08 (m, 2H), 3.18 – 3.12 (m, 1H), 2.07 (d, *J* = 14.3 Hz, 3H), 1.33 – 1.26 (m, 3H), 1.25 – 1.15 (m, 3H) ppm; *Z-isomer*: δ 7.40 – 7.29 (m, 4H), 7.24 – 7.18 (m, 1H), 5.78 (d, *J* = 9.1 Hz, 1H), 4.19 – 4.08 (m, 2H), 3.53 – 3.48 (m, 1H), 2.07 (d, *J* = 14.3 Hz, 3H), 1.33 – 1.26 (m, 3H), 1.25 – 1.15 (m, 3H) ppm.

^{13}C{^{1}H} NMR (101 MHz, $CDCl_3$) δ 175.3, 175.0, 143.2, 141.4, 138.5, 136.6, 128.21, 128.18, 127.8, 127.1, 127.0, 126.8, 126.2, 125.8, 60.5, 60.4, 39.74, 39.67, 31.6, 25.8, 22.6, 18.5, 17.9, 16.1, 14.2, 14.1 ppm.

See NMR Spectra

HRMS (*m/z*): (ESI) calc'd $C_{14}H_{18}O_2{}^{23}Na$ for $[M+Na]^+$: 241.1199, found: 241.1197.

IR (ATR) ν_{max}: 2977, 2933, 1730, 1446, 1375, 1177, 1045, 764 and 701 cm^{-1}.

Chiral HPLC (Chiralpak® IC-3, Hexane/^{i}PrOH = 97:03, 0.5 mL/min) *er* = 12.1:87.9, t_R: 11.4 min (minor), t_R: 11.7 min (major) for *E*-isomer.

Note: *The peaks for Z-isomer were not separatable.*

See HPLC Spectra

$[\alpha]_D^{24}$ = -34.86 (c = 0.01, CH_2Cl_2).

TLC: R_f = 0.78 (*n*-hexane/ethyl acetate 6:1, v/v)

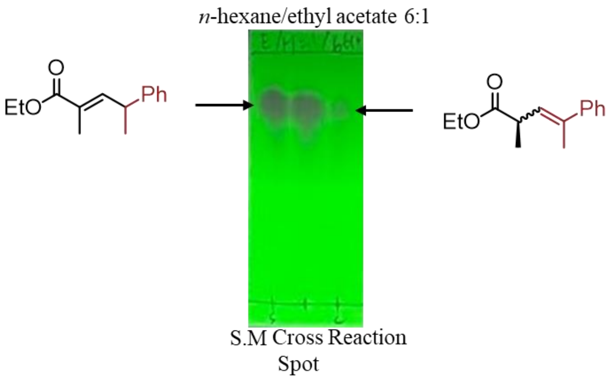

(*R*)-2-Methyl-4-phenylpent-3-enoic acid (5)

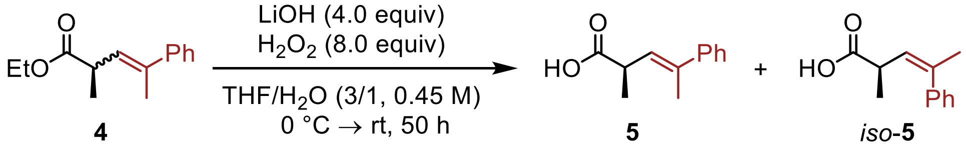

To a 0 °C solution of lithium hydroxide (8.8 mg, 0.37 mmol, 4.0 equiv) and H_2O (0.05 mL) was added 35% H_2O_2 (0.05 mL, 0.73 mmol, 8.0 equiv). Then, compound **4** (20.0 mg, 0.09 mmol, 1.0 equiv) in THF (0.15 mL) was added dropwise. The mixture was stirred at 0 °C for 2 h, and then the ice/water bath was removed and the mixture was stirred for an additional 50 h at room temperature. A solution of saturated sodium sulfite (0.4 mL) was added to the reaction mixture followed by water, ether, and 1.0 M HCl (2.0 mL) (pH approx. 1). The layers were separated, and the aqueous layer was extracted with ether (3 × 2 mL). The combined organic layers were washed with saturated $NaHCO_3$ (3 × 2 mL). Then, 1.0 M HCl was added to the combined basic aqueous layers until the pH reached approximately 1. The acidic aqueous solution was extracted with ether, and the organic layer was dried over Na_2SO_4, filtered, and concentrated under reduced pressure. The crude was purified by silica column chromatography (*n*-hexane/ethyl acetate 6:1, v/v, with 2% AcOH) to afford an *E*/*Z* mixture of compound **5** and *iso*-**5** as a pale-yellow oil (4.0 mg, 0.02 mmol, 25% yield, –76% *ee*).

The *E*/*Z* mixture of compound **5** and *iso*-**5** was separated by HPLC (Luna® CN, Hexane/^{i}PrOH = 99:01, 0.9 mL/min) to give (*E*)-2-methyl-4-phenylpent-3-enoic acid **5** (1.9 mg, 0.01 mmol, 11% yield, –76% *ee*, t_R = 9.4 min) and (*Z*)-2-methyl-4-phenylpent-3-enoic acid *iso*-**5** (2.1 mg, 0.01 mmol, 12% yield, –76% *ee*, t_R = 8.5 min).

Chiral HPLC for 5 (Chiralpak® OJ-3, Hexane/^{i}PrOH = 90:10, 0.5 mL/min) *er* = 12.2:87.8, t_R: 13.7 min (minor), t_R: 15.3 min (major).

Chiral HPLC for *iso*-5 (Chiralpak® OJ-3, Hexane/^{i}PrOH = 90:10, 0.5 mL/min) *er* = 88.1:11.9, t_R: 11.1 min (major), t_R: 16.1 min (minor).

See HPLC Spectra

$[\alpha]_D^{24}$ for 5= -36.78 (c = 0.01, CH_2Cl_2).

$[\alpha]_D^{24}$ for *iso*-5 = -34.97 (c = 0.01, CH_2Cl_2).

All the other characterization data for ***5***[8] *are consistent with the respective reported literature.*

(*R*)-2-Methyl-4,4-diphenylbut-3-en-1-ol (7)

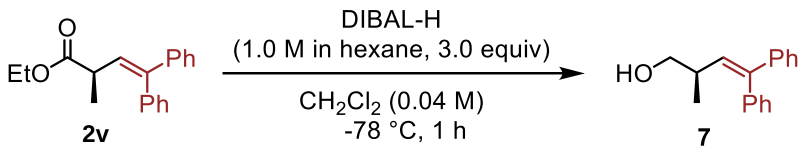

To a solution of **2v** (20 mg, 0.07 mmol, 1.0 equiv, –85% *ee*) in CH_2Cl_2 (2.0 mL) was added dropwise a 1.0 M solution of DIBAL-H in hexane (0.2 mL, 0.21 mmol, 3.0 equiv) at -78 ºC. The reaction mixture was stirred at -78 ºC for 1h and quenched with saturated potassium sodium tartrate solution (2.0 mL). The reaction was warm to rt and extracted with ether (2 mL x 3). The organic phases were dried over Na_2SO_4 and the solvent evaporated under reduced pressure. The residue was purified by column chromatography (*n*-hexane/ethyl acetate 6:1) to afford **7** as a white solid (9 mg, 0.04 mmol, 53% yield, –85% *ee*).

^{1}H NMR (400 MHz, $CDCl_3$) δ 7.42 – 7.27 (m, 7H), 7.24 – 7.20 (m, 3H), 5.90 (d, *J* = 10.2 Hz, 1H), 3.54 (d, *J* = 6.7 Hz, 2H), 2.67 – 2.57 (m, 1H), 1.05 (d, *J* = 6.7 Hz, 3H) ppm.

^{13}C{^{1}H} NMR (101 MHz, $CDCl_3$) δ 143.4, 142.2, 140.0, 131.7, 129.7, 128.3, 128.2, 128.1, 127.2, 127.1, 67.9, 36.9, 17.3 ppm.

See NMR Spectra

HRMS (*m/z*): (ESI) calc'd $C_{17}H_{18}O^{23}Na$ for $[M+Na]^+$: 261.1250, found: 261.1248.

IR (ATR) ν_{max}: 3342, 2923, 2853, 1494, 1444, 1030, 762 and 698 cm^{-1}.

Chiral HPLC (Chiralpak® IC-3, Hexane/^{i}PrOH = 90:10, 0.9 mL/min) *er* = 7.5:92.5, t_R: 7.5 min (minor), t_R: 8.2 min (major).

See HPLC Spectra

$[\alpha]_D^{24}$ = -57.63 (c = 0.01, CH_2Cl_2).

Melting point: 87 – 90 °C

TLC: R_f = 0.39 (*n*-hexane/ethyl acetate 4:1, v/v)

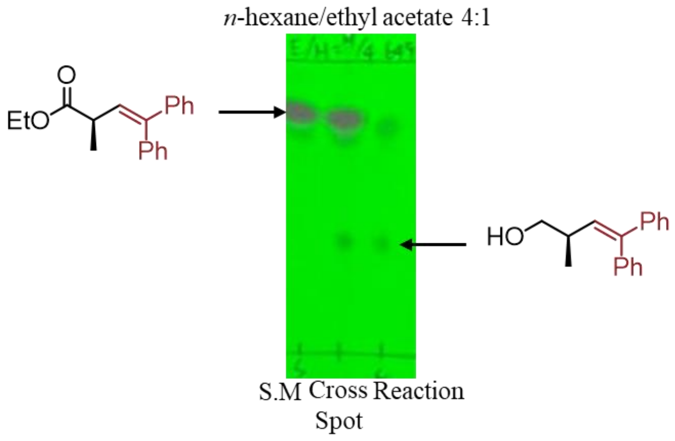

Back to contents

[1,1'-Biphenyl]-4-ylmethyl (*E*)-2-methyl-4-phenylpent-2-enoate (S4)

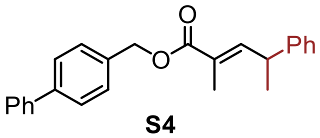

S4

According to General Procedure D, **S3k** (0.10 g, 0.5 mmol, 1.0 equiv), [1,1'-biphenyl]-4-ylmethanol (120 mg, 0.7 mmol, 1.5 equiv), DCC (130 mg, 0.6 mmol, 1.2 equiv), and DMAP (13 mg, 0.1 mmol, 0.2 equiv) were used to obtain the crude residue, which was purified by silica gel column chromatography (*n*-hexane/ethyl acetate 100:1, v/v), to afford **S4** as a pale-yellow oil (121 mg, 0.4 mmol, 71%).

^{1}H NMR (400 MHz, $CDCl_3$) δ 7.64 – 7.62 (m, 4H), 7.50 – 7.46 (m, 4H), 7.40 – 7.33 (m, 3H), 7.29 – 7.25 (m, 3H), 7.01 (d, J = 9.9 Hz, 1H), 5.30 – 5.23 (m, 2H), 3.89 – 3.81 (m, 1H), 2.01 (s, 3H), 1.45 (d, J = 6.9 Hz, 3H) ppm.

^{13}C{^{1}H} NMR (101 MHz, $CDCl_3$) δ 168.0, 146.7, 146.5, 144.4, 141.0, 140.6, 135.3, 128.7, 128.6, 127.2, 127.1, 126.5, 126.4, 66.0, 38.7, 21.3, 12.6 ppm.

See NMR Spectra

HRMS (*m/z*): (ESI) calc'd $C_{25}H_{24}O_2{}^{23}Na$ for $[M+Na]^+$: 379.1669, found: 379.1668.

IR (ATR) ν_{max}: 2965, 2925, 1707, 1234, 1019, 761 and 698 cm^{-1}.

TLC: R_f = 0.47 (*n*-hexane/ethyl acetate 10:1, v/v)

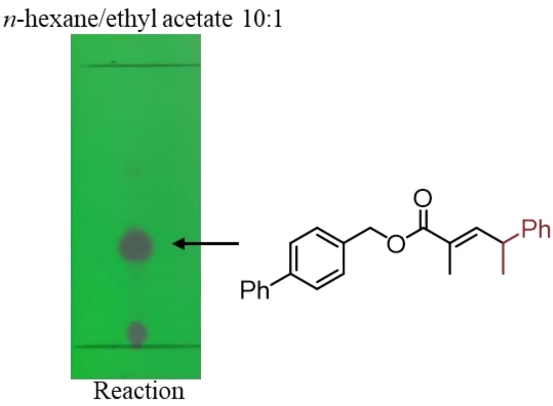

Back to contents

[1,1'-Biphenyl]-4-ylmethyl (*S*)-2-methyl-4-phenylpent-3-enoate (8)

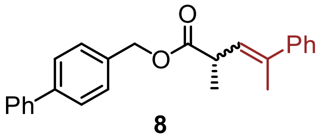

8

According to General Procedure E, **S4** (59 mg, 0.17 mmol, 1.0 equiv) and (*R*)-**D5** (9 mg, 10 mol%) were used to obtain the crude residue, which was purified by silica gel column chromatography (*n*-hexane/ethyl acetate 100:1, v/v), to afford **8** as a yellow oil (20 mg, 0.06 mmol, *E*/*Z* = 1:4.9, 34% yield, 91% *ee* for *E*-isomer).

^{1}H NMR (300 MHz, $CDCl_3$) *E-isomer*: δ 7.61 – 7.58 (m, 4H), 7.47 – 7.29 (m, 8H), 7.19 – 7.17 (m, 2H), 5.80 (d, *J* = 9.1 Hz, 1H), 5.19 – 5.15 (m, 2H), 3.65 – 3.55 (m, 1H), 2.10 – 2.05 (m, 3H), 1.37 – 1.20 (m, 3H) ppm; *Z-isomer*: δ 7.61 – 7.58 (m, 4H), 7.47 – 7.29 (m, 8H), 7.19 – 7.17 (m, 2H), 5.53 (d, *J* = 10.0 Hz, 1H), 5.19 – 5.15 (m, 2H), 3.30 – 3.20 (m, 1H), 2.10 – 2.05 (m, 3H), 1.37 – 1.20 (m, 3H) ppm.

^{13}C{^{1}H} NMR (101 MHz, $CDCl_3$) δ 175.1, 141.4, 141.0, 140.7, 138.9, 135.2, 128.8, 128.7, 128.4, 128.3, 128.2, 127.9, 127.3, 127.2, 127.1, 126.0, 125.9, 66.1, 65.9, 39.81, 39.78, 25.80, 25.79, 18.53, 18.47 ppm.

See NMR Spectra

HRMS (*m/z*): (ESI) calc'd $C_{25}H_{24}O_2{}^{23}Na$ for $[M+Na]^+$: 379.1669, found: 379.1668.

IR (ATR) ν_{max}: 2968, 2930, 1731,1488, 1160, 761 and 699 cm^{-1}.

Chiral HPLC (Chiralpak® IC-3, Hexane/^{i}PrOH = 97:03, 0.5 mL/min) *er* = 95.6:4.4, t_R: 16.3 min (major), t_R: 17.0 min (minor) for *E*-isomer.

Note: *The peaks for Z-isomer were not separatable.*

See HPLC Spectra

$[\alpha]_D^{24}$ = 50.83 (c = 0.01, CH_2Cl_2).

TLC: R_f = 0.57 (*n*-hexane/ethyl acetate 6:1, v/v)

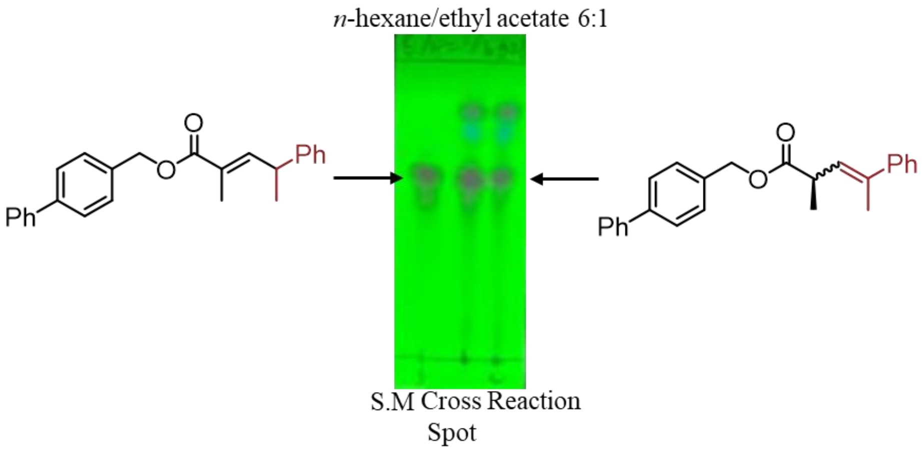

[1,1'-Biphenyl]-4-ylmethyl (2*S*,4*R*)-2-methyl-4-phenylpentanoate (9)

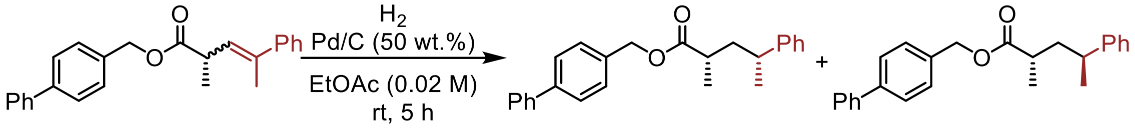

A solution of Palladium on carbon (4 mg, 50 wt.%) in EtOAc (0.5 mL) was added a *E*/*Z* mixture (1:4.9) of **8** (8 mg, 0.02 mmol) in EtOAc (0.5 mL) under hydrogen and stirred at rt for 6 h. Then the solution was filtered through a celite pad and concentrated under reduced pressure to afford **9** as a colorless oil (8 mg, 0.02 mmol, >99% yield, *syn*/*anti* = 1.3:1, 89% *ee* for major isomer).

^{1}H NMR (300 MHz, $CDCl_3$) *syn*-**9**: δ 7.60 – 7.47 (m, 4H), 7.44 – 7.30 (m, 6H), 7.20 – 7.10 (m, 4H), 5.08 – 5.02 (m, 2H), 2.75 – 2.70 (m, 1H), 2.43 – 2.33 (m, 1H), 2.11 – 1.99 (m, 2H), 1.27 – 1.21 (m, 3H), 1.19 – 1.12 (m, 3H) ppm; *anti*-**9**: δ 7.60 – 7.47 (m, 4H), 7.44 – 7.30 (m, 6H), 7.20 – 7.10 (m, 4H), 5.16 – 5.10 (m, 2H), 2.75 – 2.70 (m, 1H), 2.43 – 2.33 (m, 1H), 2.11 – 1.99 (m, 2H), 1.27 – 1.21 (m, 3H), 1.19 – 1.12 (m, 3H) ppm.

The peaks belong to syn- or anti-isomers were distinguished based on the reference.[9]

^{13}C{^{1}H} NMR (101 MHz, $CDCl_3$) δ 176.7, 176.5, 146.6, 146.4, 146.2, 141.14, 141.09, 140.7, 135.2, 129.5, 128.8, 128.6, 128.5, 128.44, 128.40, 127.4, 127.3, 127.12, 127.07, 127.0, 126.2, 126.1, 65.7, 42.4, 41.9, 41.6, 38.0, 37.6, 29.7, 22.6, 22.5, 18.0, 17.1 ppm.

See NMR Spectra

HRMS (*m/z*): (ESI) calc'd $C_{25}H_{26}O_2{}^{23}Na$ for $[M+Na]^+$: 381.1825, found: 381.1824.

IR (ATR) ν_{max}: 2959, 2925, 1732, 1452, 1160, 761 and 699 cm^{-1}.

Chiral HPLC (Chiralpak® IC-3, Hexane/^{i}PrOH = 97:03, 0.9 mL/min) *er* = 94.4:5.6, t_R: 9.4 min (major), t_R: 10.1 min (minor) for major isomer.

Note: The peaks for minor isomer were not separatable.

See HPLC Spectra

$[\alpha]_D^{24}$ = 47.62 (c = 0.01, CH_2Cl_2).

TLC: R_f = 0.79 (*n*-hexane/ethyl acetate 6:1, v/v)

n-hexane/ethyl acetate 6:1

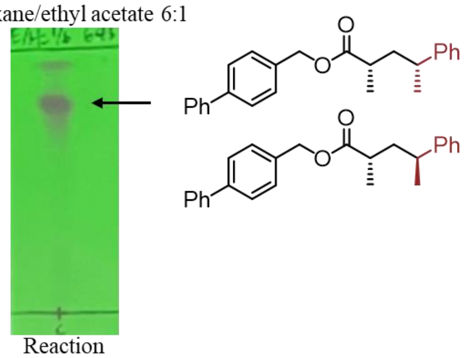

Reaction

5.2 Gram-scale photoflow chemistry

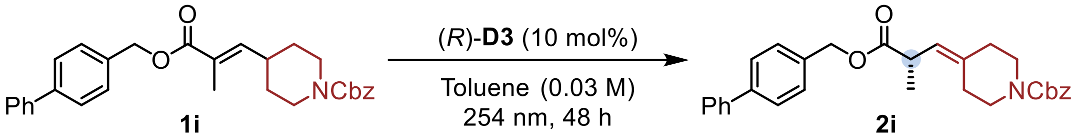

In a round-bottom flask with septum, **1i** (1.41 g, 3.0 mmol, 1.0 equiv) and (*R*)-**D3** (170.0 mg, 10 mol%) were dissolved in degassed toluene (95 mL) under nitrogen condition and stirred for 10 min (Fig. A). On the other hand, the flow reactor (tube: FEP, 1/8) was purged by the nitrogen balloon (Fig. B). After purging, the solution was transferred to a syringe and injected to the reactor through the syringe pump at 5.0 mL/h (Fig. C). Subsequently, the 254 nm Hg-lamps and cooling fan were turned on (Fig. D and E). The photoflow reactor was allowed to continue to flow until all reaction mixture had been collected to the round bottom flask (Fig. F). After the reaction finished, ethyl acetate (10 mL) was injected to wash the reactor. The residue was concentrated under reduced pressure and purified by silica gel column chromatography (*n*-hexane/ethyl acetate 50:1, v/v) to afford **2i** as a colorless oil (1.01 g, 2.2 mmol, 72% yield, 92% *ee*) (Fig. G).

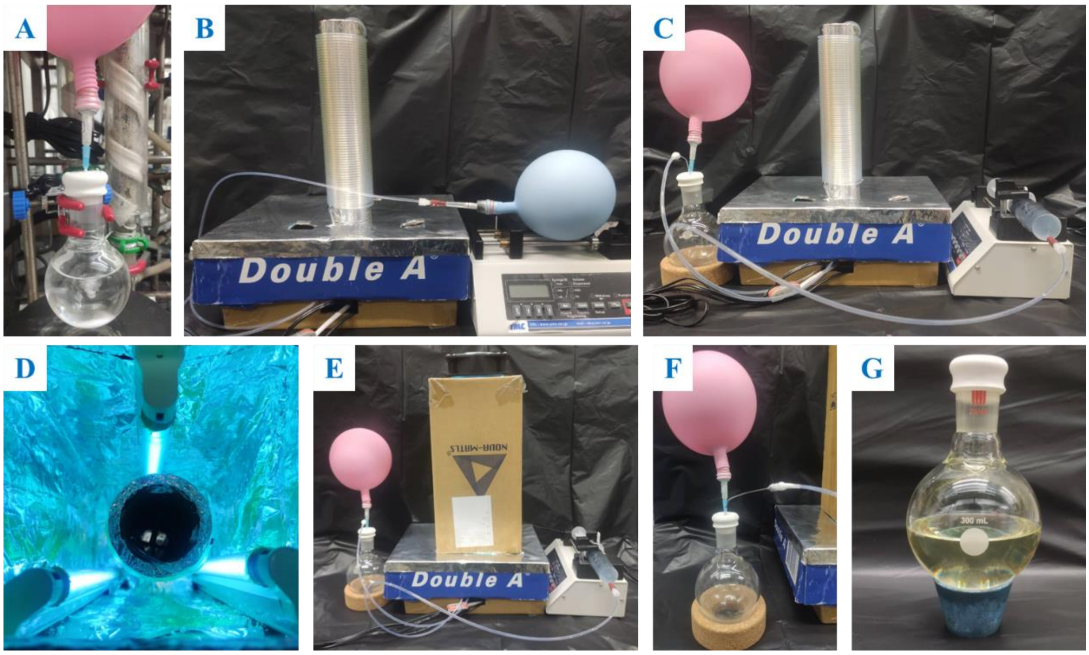

Figure S5.1 Home-made gram-scale reactor.

Productivity and residence time calculations:

$$\text{Productivity} = \text{Yield} \times \text{Flow rate} \times \text{Concentration}$$
$$= 0.72 \times 5.0\,\text{mL/h} \times 0.03\,\text{mmol/mL} = 0.108\,\text{mmol/h}$$

$$\text{Residence time } (t_r) = \frac{\text{Reaction scale in mmol}}{\text{Productivity}} = \frac{3.0\,\text{mmol}}{0.108\,\text{mmol/h}} = 27.8\,\text{h}$$

6. Mechanism Studies

6.1 Orthogonal *in situ* reaction monitoring with infrared spectroscopy

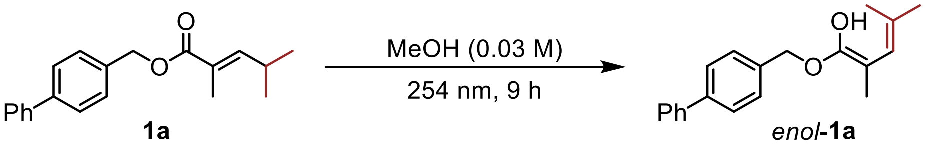

In a quartz tube, **1a** (180 mg, 0.6 mmol, 1.0 equiv) was dissolved in MeOH (20 mL) under nitrogen atmosphere, which was then irradiated at 254 nm for 9 h. During the irradiation process, a peristaltic pump is used for circulation and monitored by React IR.

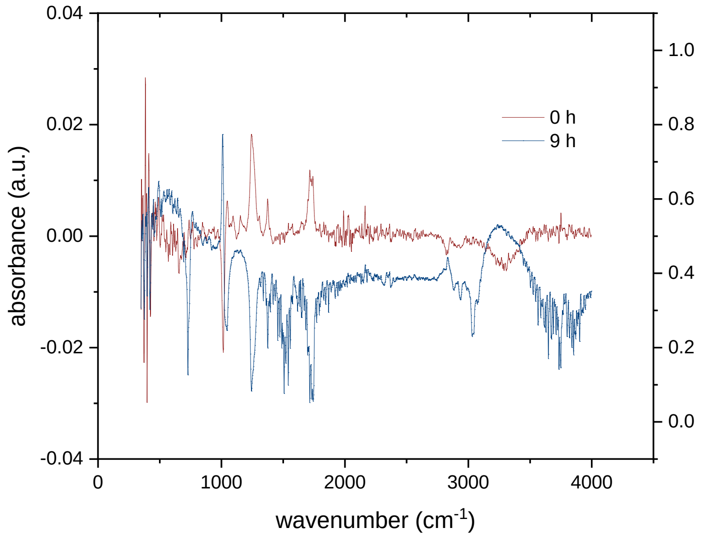

Figure S6.1 The 2D-plot for initial and final result of React IR.

6.2 NMR monitoring studies

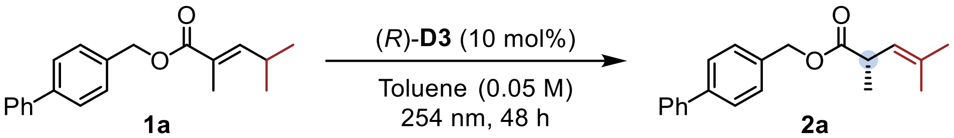

In a round bottom flask, **1a** (110 mg, 0.4 mmol, 1.0 equiv) and (*R*)-**D3** (21 mg, 10 mol%) were dissolved in degassed toluene (5.5 mL) under nitrogen condition. The reaction mixture was divided into 11 aliquots and transferred into quartz tube with septum. The aliquots were irradiated at 254 nm for 0, 0.5, 1, 1.5, 2, 3, 4, 6, 12, 24 and 36 h. Following the irradiation steps, toluene was removed under reduced pressure. The ^{1}H NMR yield of **1a**, (*Z*)-**1a** and **2a** was determined by using tetrachloroethane as internal standard, and the enantiomeric excess of **2a** was determined by chiral high-performance liquid chromatography.

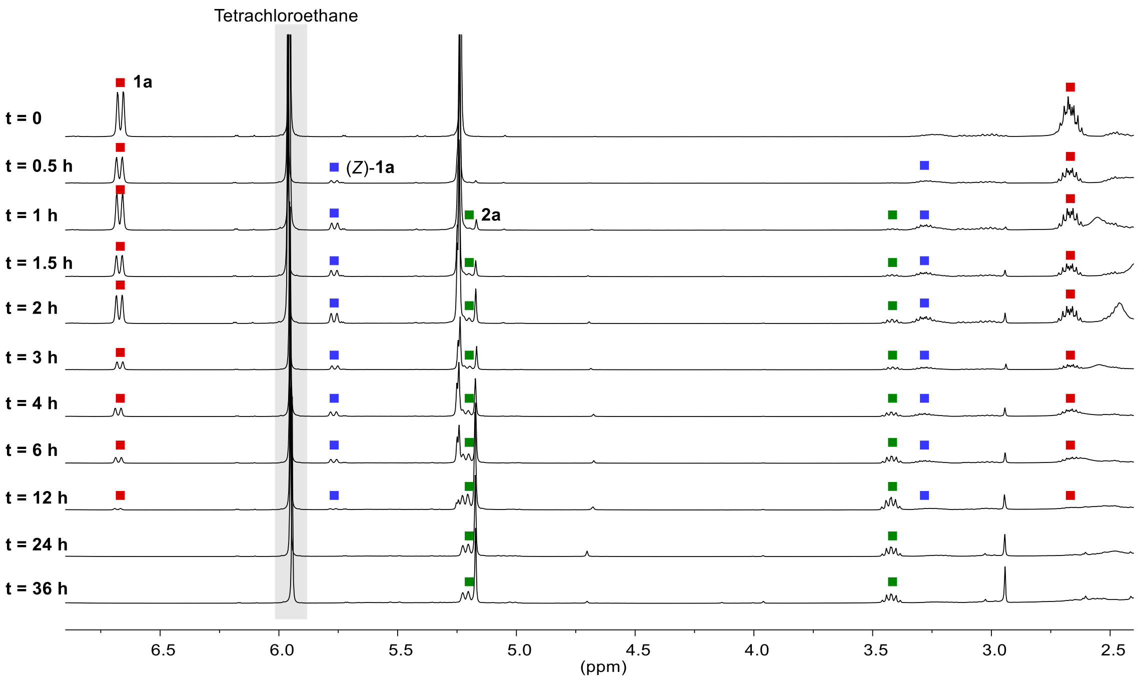

Figure S6.2 ^{1}H NMR spectra of the reaction mixture at different time point.

reaction time (h)	yield of **1a** (%)	yield of (*Z*)-**1a** (%)	yield of **2a** (%)	*ee* ratio of **2a** (%)
0	100	0	0	-
0.5	99	1	0	-
1	85	14	0	94
1.5	77	21	1	94
2	69	23	10	94
3	54	25	23	94
4	42	23	30	94
6	29	19	51	94
12	0	1	80	94
24	0	0	86	94
36	0	0	87	94

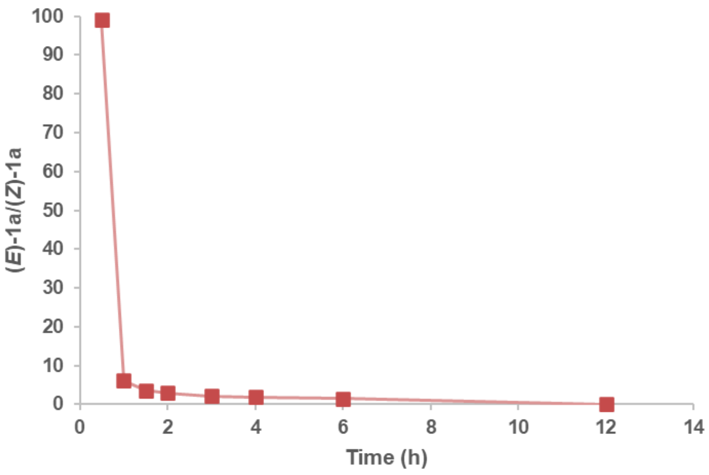

Figure S6.3 The ratio of *E*/*Z*-isomers of the reaction.

6.3 Light on-off experiments

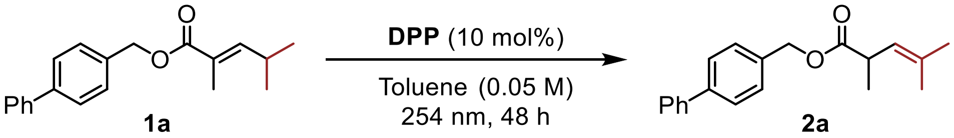

In a round bottom flask, **1a** (60 mg, 0.2 mmol, 1.0 equiv) and **DPP** (5 mg, 10 mol%) were dissolved in toluene (3 mL) under nitrogen atmosphere. The reaction mixture was divided into 6 aliquots and transferred into quartz tube with septum. The aliquots were reacted for 0, 6, 9, 15, 18 and 24 h. Following the irradiation steps, toluene was removed under reduced pressure. The ^{1}H NMR yield of **1a**, (*Z*)-**1a** and **2a** was determined by using tetrachloroethane as internal standard.

reaction time (h)	yield of **1a** (%)	yield of (*Z*)-**1a** (%)	yield of **2a** (%)
0	100	0	0
6	16	10	42
9	16	10	42
15	0	1	83
18	0	1	84
24	0	0	94

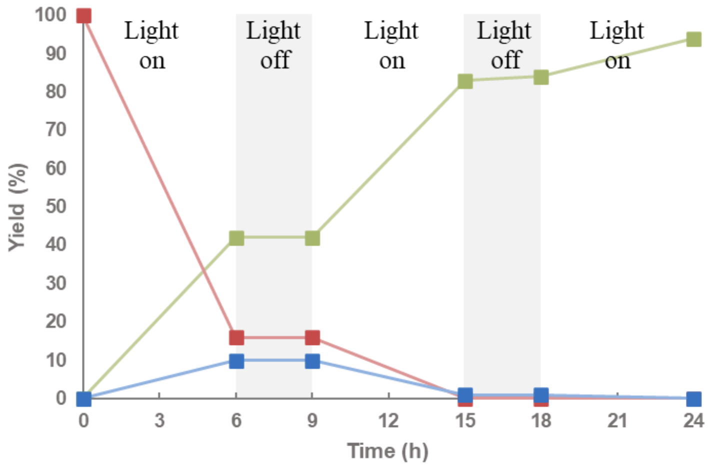

Figure S6.4 Light on-off experiment (red line: **1a**, blue line: (*Z*)-**1a**, green line: **2a**).

Figure S6.4 showed that the amounts of 1a, (Z)-1a, and photodeconjugated 2a remain constant under the lights off phase, pointing out that all reactions halted in the absence of light and no photo-ketene hemiacetal can be generated from 1a and (Z)-1a.

Back to contents

6.4 Reversible test of the *E*/*Z* isomerization

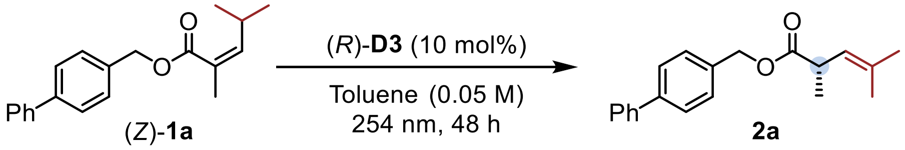

In a round bottom flask, (*Z*)-**1a** (60 mg, 0.2 mmol, 1.0 equiv) and (*R*)-**D3** (12 mg, 10 mol%) were dissolved in toluene (3 mL) under nitrogen atmosphere. The reaction mixture was divided into 6 aliquots and transferred into quartz tube with septum. The aliquots were irradiated at 254 nm for 0, 1, 2, 3, 6 and 48 h. Following the irradiation steps, toluene was removed under reduced pressure. The ^{1}H NMR yield of (*Z*)-**1a**, **1a** and **2a** was determined by using tetrachloroethane as internal standard.

reaction time (h)	yield of (*Z*)-**1a** (%)	yield of **1a** (%)	yield of **2a** (%)	*ee* ratio of **2a** (%)
0	100	0	0	-
1	68	4	32	94
2	21	5	63	94
3	3	1	90	94
6	0	0	92	94
48	0	0	92	94

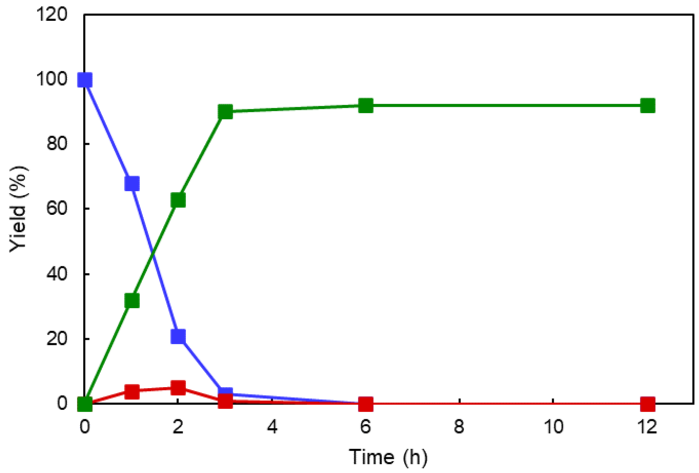

Figure S6.5 Reaction monitoring of asymmetric Norrish type II reaction of (*Z*)-**1a** (red line: **1a**, blue line: (*Z*)-**1a**, green line: **2a**).

Back to contents

6.5 Irreversible test of the keto-enol tautomerization

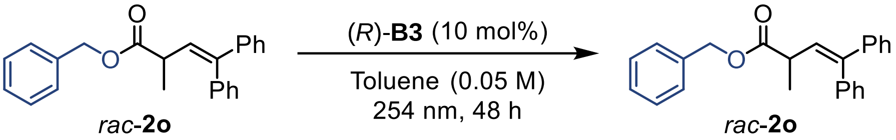

In a quartz tube, *rac*-**2o** (17 mg, 0.05 mmol, 1.0 equiv, 0% *ee*) and (*R*)-**B3** (4 mg, 10 mol%) were dissolved in toluene (1.0 mL) under nitrogen atmosphere. The reaction mixture was irradiated at 254 nm for 48 h. Following the irradiation steps, toluene was removed under reduced pressure. The crude was purified by silica gel column chromatography (*n*-hexane/ethyl acetate 100:1, v/v), to afford *rac*-**2o** (16 mg, 0.05 mmol, 94% yield, 0% *ee*).

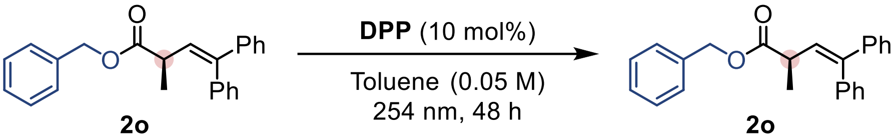

In a quartz tube, (*R*)-**2o** (17 mg, 0.05 mmol, 1.0 equiv, -90% *ee*) and **DPP** (1 mg, 10 mol%) were dissolved in toluene (1.0 mL) under nitrogen atmosphere. The reaction mixture was irradiated at 254 nm for 48 h. Following the irradiation steps, toluene was removed under reduced pressure. The crude was purified by silica gel column chromatography (*n*-hexane/ethyl acetate 100:1, v/v), to afford (*R*)-**2o** (16 mg, 0.05 mmol, 96% yield, -90% *ee*).

6.6 Control experiments

Background reaction test

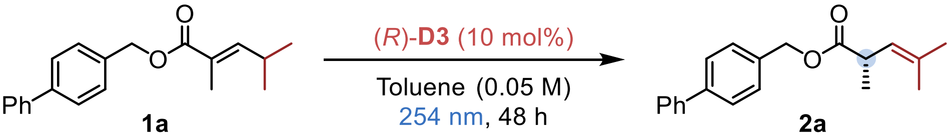

In a quartz tube, **1a** (15 mg, 0.05 mmol, 1.0 equiv) and (*R*)-**D3** (3 mg, 10 mol%) were dissolved in toluene (1 mL) under nitrogen atmosphere. The reaction mixture was irradiated at 254 nm for 48 h. Following the irradiation steps, toluene was removed under reduced pressure. The ^{1}H NMR yield of **1a** and **2a** was determined by using tetrachloroethane as internal standard, and the enantiomeric excess of **2a** was determined by chiral high-performance liquid chromatography.

Entry	Variation from condition	Yield of **1a** (%)	Yield of **2a** (%)	*ee* of **2a** (%)
1	No (*R*)-**D3**	0	74	0
2	No light	>99	0	NA

Effect of oxygen

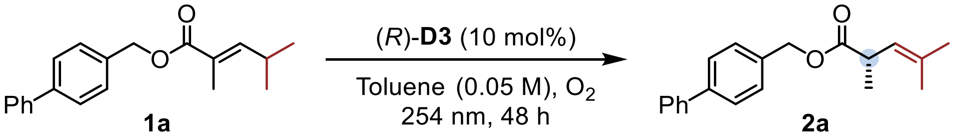

In a quartz tube, **1a** (15 mg, 0.05 mmol, 1.0 equiv), (*R*)-**D3** (3 mg, 10 mol%) were dissolved in toluene (1.0 mL). The reaction mixture was purged with oxygen. Then, it was irradiated at 254 nm for 48 h. Following the irradiation steps, toluene was removed under reduced pressure. The crude was purified by silica gel column chromatography (*n*-hexane/ethyl acetate 100:1, v/v) to afford **2a** (11 mg, 0.04 mmol, 72% yield, 60% *ee*).

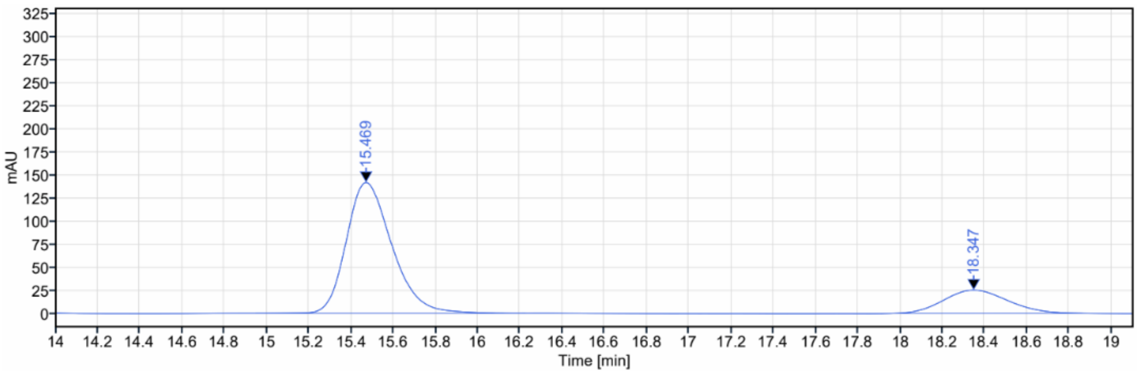

Signal: DAD1A,Sig=250,4 Ref=off

RT [min]	Type	Width [min]	Area	Height	Area%	Name
15.469	MM m	0.23	2128.35	141.91	79.95	
18.347	MM m	0.33	533.66	25.24	20.05	

A decrease in the yield and enantioselectivity of the photodeconjugated product 2a was observed when nitrogen atmosphere was changed to oxygen, from 89% yield, 94% ee to 72% yield, 60% ee. This 22% yield difference could possibly point out that there is a triplet state process existing in the mechanistic scenario that was quenched by oxygen as an efficient triplet quencher[10-13], either from then E/Z isomerization step or Norrish type II (1,5-HAT) process as a minor photoenolization pathway.

6.7 *E*/*Z* isomerization of unsymmetric product

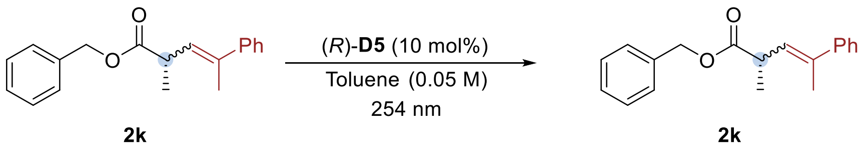

In a quartz tube, **2a** (20 mg, 0.05 mmol, 1.0 equiv), (*R*)-**D5** (4 mg, 10 mol%) were dissolved in toluene-d$_8$ (1.0 mL) under nitrogen condition. The reaction mixture was irradiated at 254 nm for 0, 1, 2, 4, 8, 16 and 48 h. Following the irradiation steps, the *E*/*Z* ratio of **2k** was determined by ^{1}H NMR.

Time (h)	E/Z
0	1/0.43
1	1/1.02
2	1/1.42
4	1/1.92
8	1/2.28
16	1/2.91
48	1/3.80

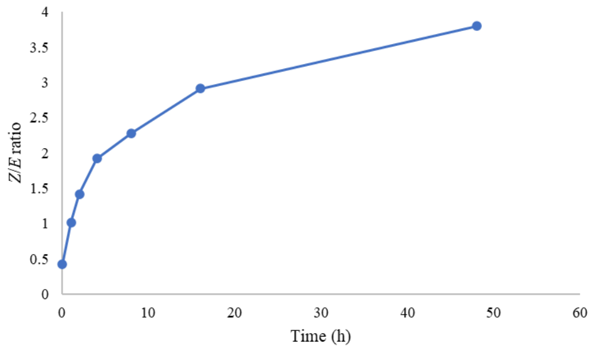

Figure S6.6 *E*/*Z* isomerization of **2k**

This experiment showed that the π-bonding at the β,γ-position was able to be excited and induced E/Z isomerization under 254 nm irradiation.

6.8 Effect of water

Effect of water for (*S*)-D3

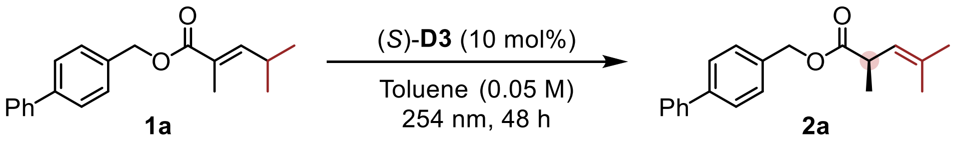

In a quartz tube, **1a** (15 mg, 0.05 mmol, 1.0 equiv) and (*S*)-**D3** (3 mg, 10 mol%) were dissolved in toluene (1.0 mL) under nitrogen atmosphere. The reaction mixture was irradiated at 254 nm for 48 h. Following the irradiation steps, toluene was removed under reduced pressure. The crude was purified by silica gel column chromatography (*n*-hexane/ethyl acetate 100:1, v/v) to afford **2a** (13 mg, 0.04 mmol, 87% yield, 0% *ee*).

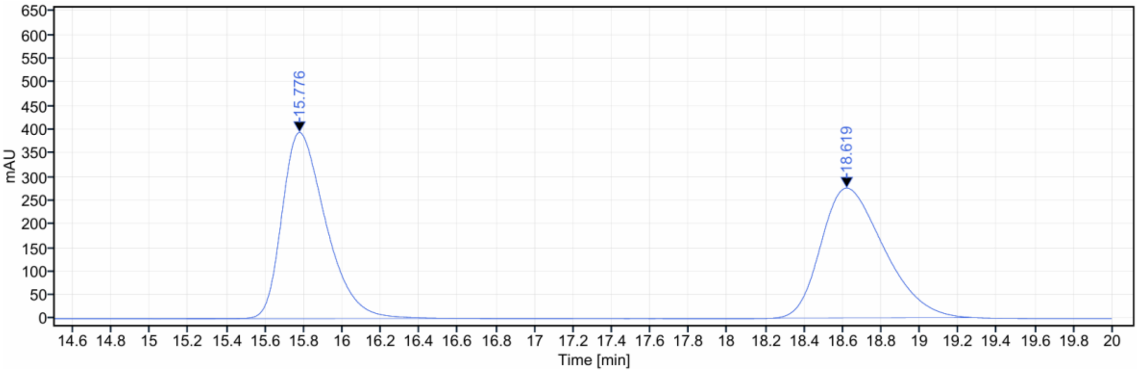

Signal:	DAD1A,Sig=250,4 Ref=off					
RT [min]	Type	Width [min]	Area	Height	Area%	Name
15.776	MM m	0.24	6235.77	393.70	50.00	
18.619	MM m	0.35	6234.78	274.59	50.00	

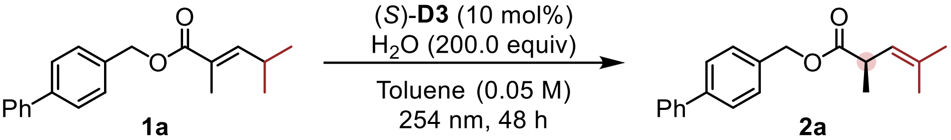

In a quartz tube, **1a** (15 mg, 0.05 mmol, 1.0 equiv) and (*S*)-**D3** (3 mg, 10 mol%) were dissolved in toluene (1 mL) under nitrogen atmosphere. The mixture was then adding deionized water (0.2 mL, 10.0 mmol, 200.0 equiv). The reaction mixture was irradiated at 254 nm for 48 h. Following the irradiation steps, toluene was removed under reduced pressure. The crude was purified by silica gel column chromatography (*n*-hexane/ethyl acetate 100:1, v/v) to afford **2a** (13 mg, 0.04 mmol, 85%, -93% *ee*).

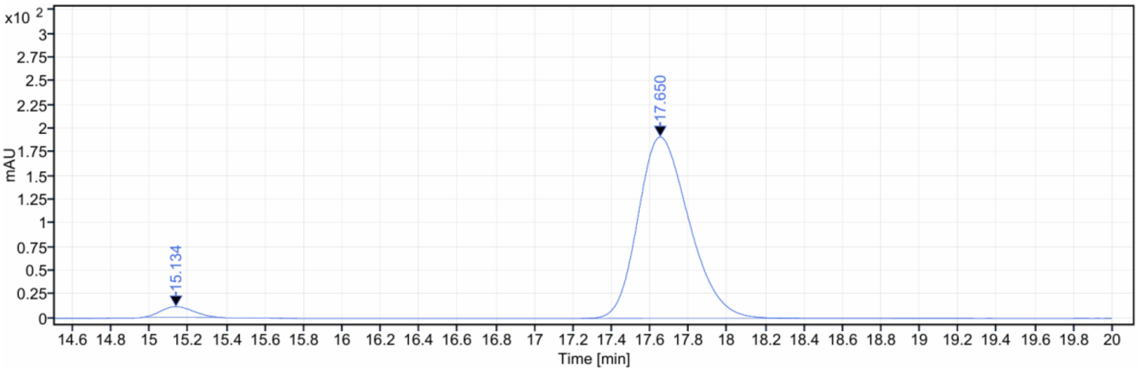

Signal:	DAD1A,Sig=250,4 Ref=off					
RT [min]	Type	Width [min]	Area	Height	Area%	Name
15.134	MM m	0.18	131.87	11.27	3.61	
17.650	MM m	0.28	3523.35	191.50	96.39	

These experiments showed that additional water is necessary for the enantioselectivity of the reaction when using (S)-D3.

Correlation of water loading to *ee* ratio

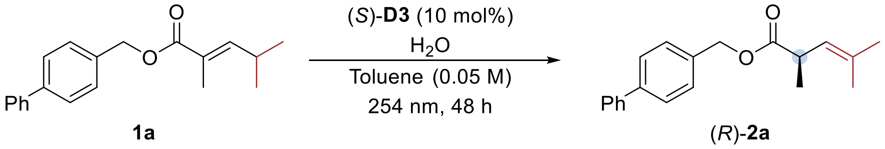

In a quartz tube, **1a** (15 mg, 0.05 mmol, 1.0 equiv) and (*S*)-**D3** (3 mg, 10 mol%) were dissolved in toluene (1 mL) under nitrogen atmosphere. The mixture was then adding deionized water. The reaction mixture was irradiated at 254 nm for 48 h. Following the irradiation steps, toluene was removed under reduced pressure. The crude was purified by silica gel column chromatography (*n*-hexane/ethyl acetate 100:1, v/v) to afford **2a**.

equiv. of H_2O	yield (%)	*ee* (%)
0	90	1
10	87	29
50	87	62
100	86	82
200	89	94

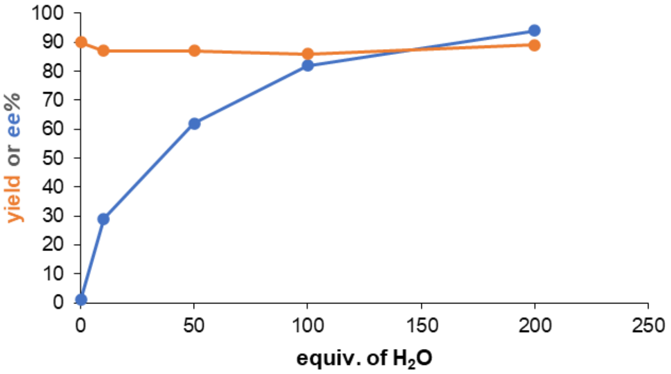

Figure S6.7 Yields or *ee* ratio correlates to water loadings (orange line: yield%, blue line: *ee*%).

This experiment showed that the addition of water has no effect on yield, but has an effect on ee ratio. As the amount of water increases, so does the ee ratio.

Isotope labelling experiment

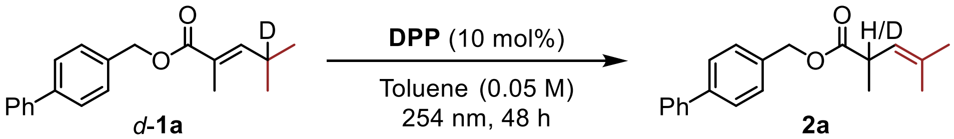

In a quartz tube, *d*-**1a** (15 mg, 0.05 mmol, 1.0 equiv) and **DPP** (1 mg, 10 mol%) were dissolved in toluene (1 mL) under nitrogen atmosphere. The reaction mixture was irradiated at 254 nm for 48 h. Following the irradiation steps, toluene was removed under reduced pressure. The crude was purified by silica gel column chromatography (*n*-hexane/ethyl acetate 100:1, v/v) to afford **2a** (13 mg, 0.04 mmol, 85% yield, 10% D).

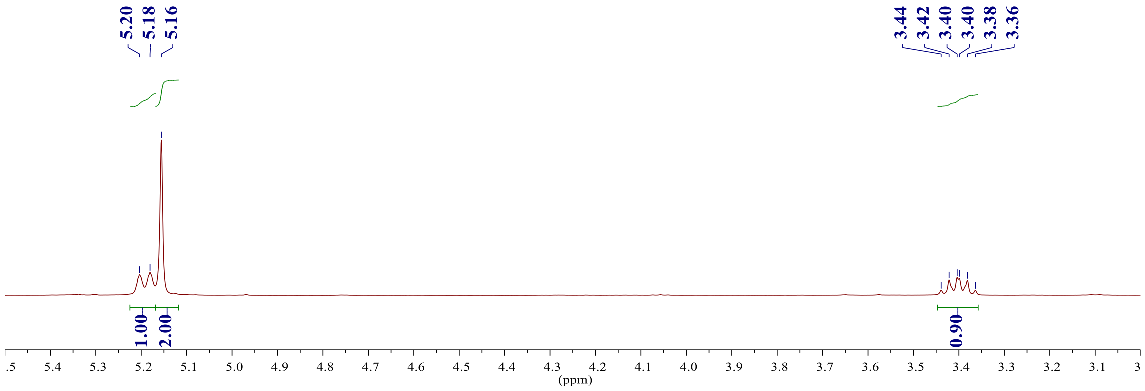

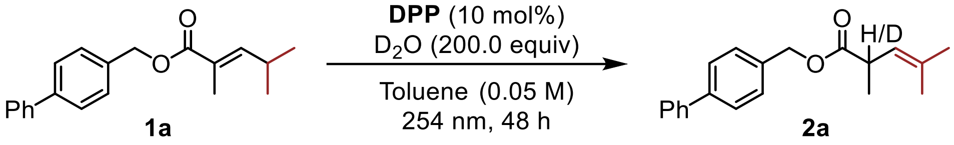

In a quartz tube, **1a** (15 mg, 0.05 mmol, 1.0 equiv) and **DPP** (1 mg, 10 mol%) were dissolved in toluene under nitrogen atmosphere. The reaction mixture was added D_2O and irradiated at 254 nm for 48 h. Following the irradiation steps, toluene was removed under reduced pressure. The crude was purified by silica gel column chromatography (*n*-hexane/ethyl acetate 100:1, v/v) to afford **2a** (13 mg, 0.04 mmol, 87% yield, 79% D).

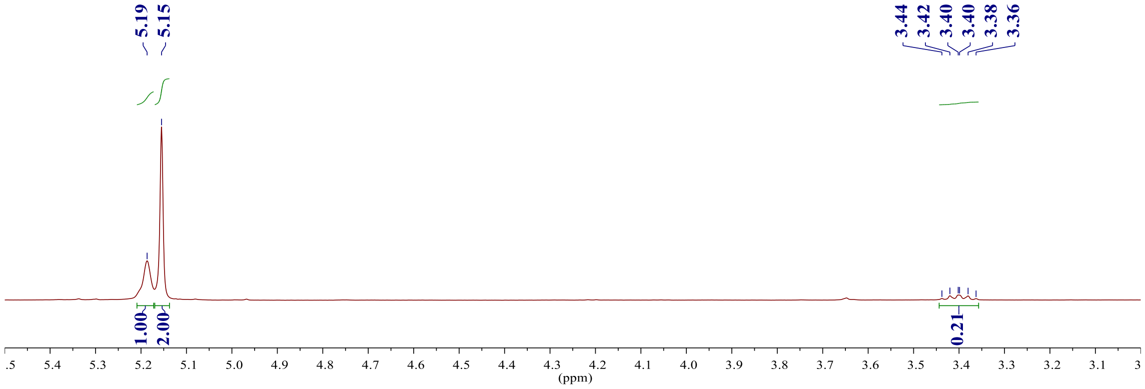

These isotope labelling experiments showed that the proton for the tautomerization/protonation step of the photo-ketene hemiacetal to the photodeconjugated product 2a was from an external proton source rather than directly from the substrate itself.

Alcohol as external proton source

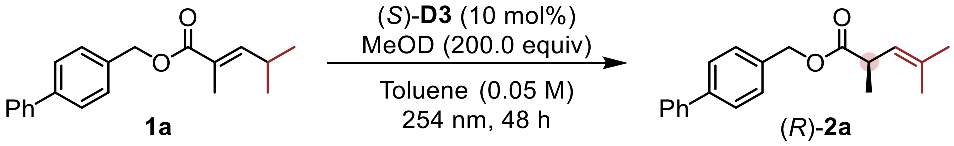

In a quartz tube, **1a** (15 mg, 0.05 mmol, 1.0 equiv) and (*S*)-**D3** (3 mg, 10 mol%) were dissolved in toluene (1 mL) under nitrogen atmosphere. The mixture was then adding methanol-d_4 (0.4 mL, 10.0 mmol, 200.0 equiv). The reaction mixture was

irradiated at 254 nm for 48 h. Following the irradiation steps, toluene was removed under reduced pressure. The crude was purified by silica gel column chromatography (*n*-hexane/ethyl acetate 100:1, v/v) to afford **2a** (12 mg, 0.05 mmol, 78% yield, 0% *ee*, 12% D).

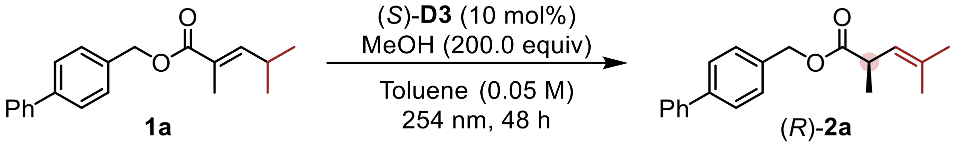

In a quartz tube, **1a** (15 mg, 0.05 mmol, 1.0 equiv) and (*S*)-**D3** (3 mg, 10 mol%) were dissolved in toluene (1 mL) under nitrogen atmosphere. The mixture was then adding methanol (0.4 mL, 10.0 mmol, 200.0 equiv). The reaction mixture was irradiated at 254 nm for 48 h. Following the irradiation steps, toluene was removed under reduced pressure. The crude was purified by silica gel column chromatography (*n*-hexane/ethyl acetate 100:1, v/v) to afford **2a** (12 mg, 0.05 mmol, 79% yield, 0% *ee*).

These results showed that water cannot be replaced with methanol as an external proton source.

KIE experiment of 1,5-HAT step

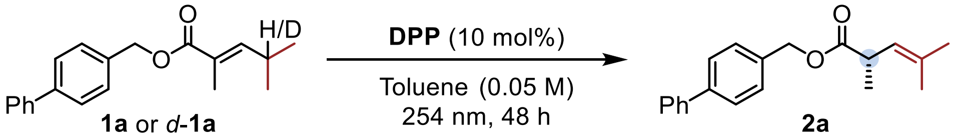

In a round bottom flask, **1a** or *d*-**1a** (40 mg, 0.1 mmol, 1.0 equiv) and **DPP** (3 mg, 10 mol%) were dissolved in toluene (2 mL) under nitrogen atmosphere. The reaction mixture was divided into 4 aliquots and transferred into quartz tube with septum. The aliquots were irradiated at 254 nm for 0, 1, 2 and 3 h. Following the irradiation steps, toluene was removed under reduced pressure. The ^{1}H NMR yield of **2a** was determined by using tetrachloroethane as internal standard.

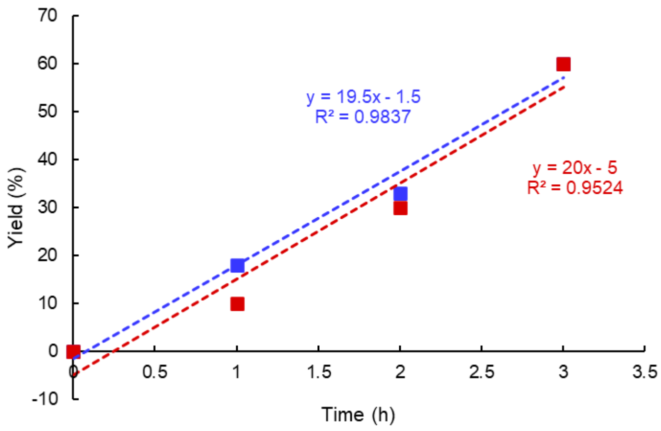

Figure S6.8 KIE experiment of 1,5-HAT step (red line: **1a** as starting material, blue line: *d*-**1a** as starting material)

The result of k_H/k_D is 1.03, which means the 1,5-HAT step is not a rate-determining step.

KIE experiment of formal 1,3-protonation step

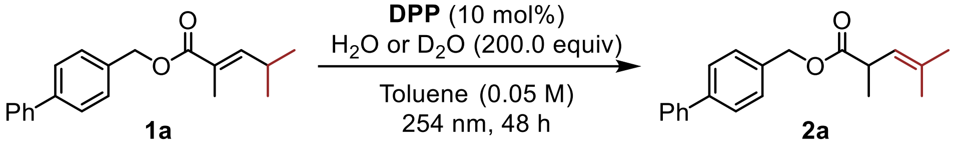

In a round bottom flask, **1a** (40 mg, 0.1 mmol, 1.0 equiv) and **DPP** (3 mg, 10 mol%) were dissolved in toluene (2.0 mL) under nitrogen atmosphere. The reaction mixture was added H_2O or D_2O and divided into 4 quartz tubes with septum. The aliquots were irradiated at 254 nm for 0, 1, 2 and 3 h. Following the irradiation steps, toluene was removed under reduced pressure. The ^1H NMR yield of **2a** was determined by using tetrachloroethane as internal standard.

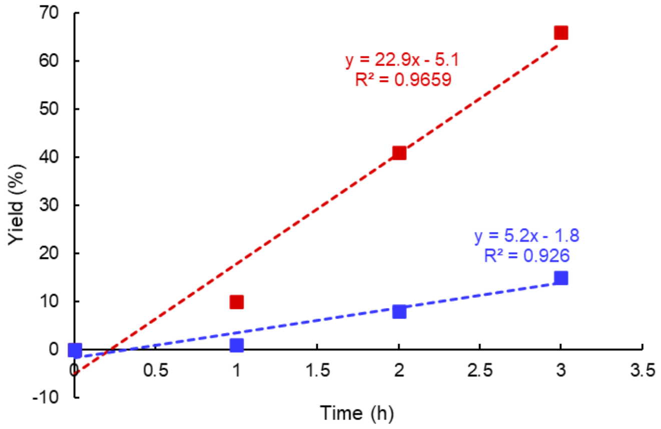

Figure S6.9 KIE experiment of formal 1,3-protonation step (red line: reaction with H_2O, blue line: reaction with D_2O)

The result of k_H/k_D is 4.40, which means the formal 1,3-protonation step is a rate-determining step.

DFT calculation

To probe the potential of having an energetically favorable alternative reaction pathway with the participation of water in the transition state for the protonation step, DFT calculations were perform.

The calculations were carried out with the Gaussian 16 program.[14] All DFT calculations were conducted with the hybrid exchange correlation-functional B3LYP[15–17] in combination with the 6-31++G(d,p) basis set.[18–21] To include solvent effects (toluene and water), polarizable continuum model (PCM)[22] was implemented; particularly, the solvation model density (SMD) variant of the integral equation formalism variant PCM (IEFPCM) was applied, recommended for the calculations of ΔG.[23] Vibrational

frequencies were computed analytically within the harmonic approximation and were used to estimate the Gibbs free energies at 298 K.

Five different pathways for the protonation of the photo-ketene hemiacetal were probed, namely, (1, **Path a**) intramolecular 1,3-proton transfer (uncatalyzed/background reaction), (2, **Path b**) water as an external proton source, (3, **Path c**) DMP as an external proton source, (4, **Path d**) DMP as an external proton source with spectator water, and (5, **Path e**) DMP-water as a single proton shuttle system (Figure S6.8). The initial states (IS), transition states (TS), and products (P) for these plausible protonation pathways were searched in gaseous and solution phase (toluene). Their corresponding reaction energies, activation barriers and related Gibbs free energies were calculated, which were tabulated in the following table.

Table S6.1 Computed reaction energies ΔE_r and activation barriers ΔE_a as well as the Gibbs free energies ΔG_r of reaction and activation ΔG_a in kJ/mol for the different proton transfer mechanisms using different solvent models.

		**Path a**	**Path b**	**Path c**	**Path d**	**Path e**
Gas phase	ΔE_a (ΔG_a)	187 (189)	82 (90)	6 (13)	7 (12)	17 (25)
	ΔE_r (ΔG_r)	-81 (-82)	-82 (-88)	-90 (-94)	-82 (-89)	-83 (-90)
Toluene	ΔE_a (ΔG_a)	184 (184)	87 (95)	10 (14)	6 (14)	--
	ΔE_r (ΔG_r)	-86 (-86)	-83 (-87)	-89 (-92)	-84 (-87)	--

All reactions were found to be exothermic. In the gaseous phase calculations, the presence of water (**Path b**) significantly reduced the activation energy (ΔE_a) by more than 100 kJ/mol when compared to the uncatalyzed reaction (intramolecular 1,3-proton transfer, **Path a**), while that of the pathways involving DMP (**Paths c-e**) gave much lower ΔE_a values of 6 to 17 kJ/mol. A similar trend for the ΔG values were observed. Therefore, the most probable pathway for the protonation step clearly involve DMP. Further, a 10-membered TS involving DMP-water as a proton shuttle system (**Path e**), where water acts as a proton donor and DMP as the proton acceptor to the photo-ketene hemiacetal, was located slightly higher in energy than the TS in **Path c**. However, this same TS was not located under implicit toluene solvation, leading us to rule out **Path e** as a viable protonation pathway. Further, we considered the possibility of water as a spectator

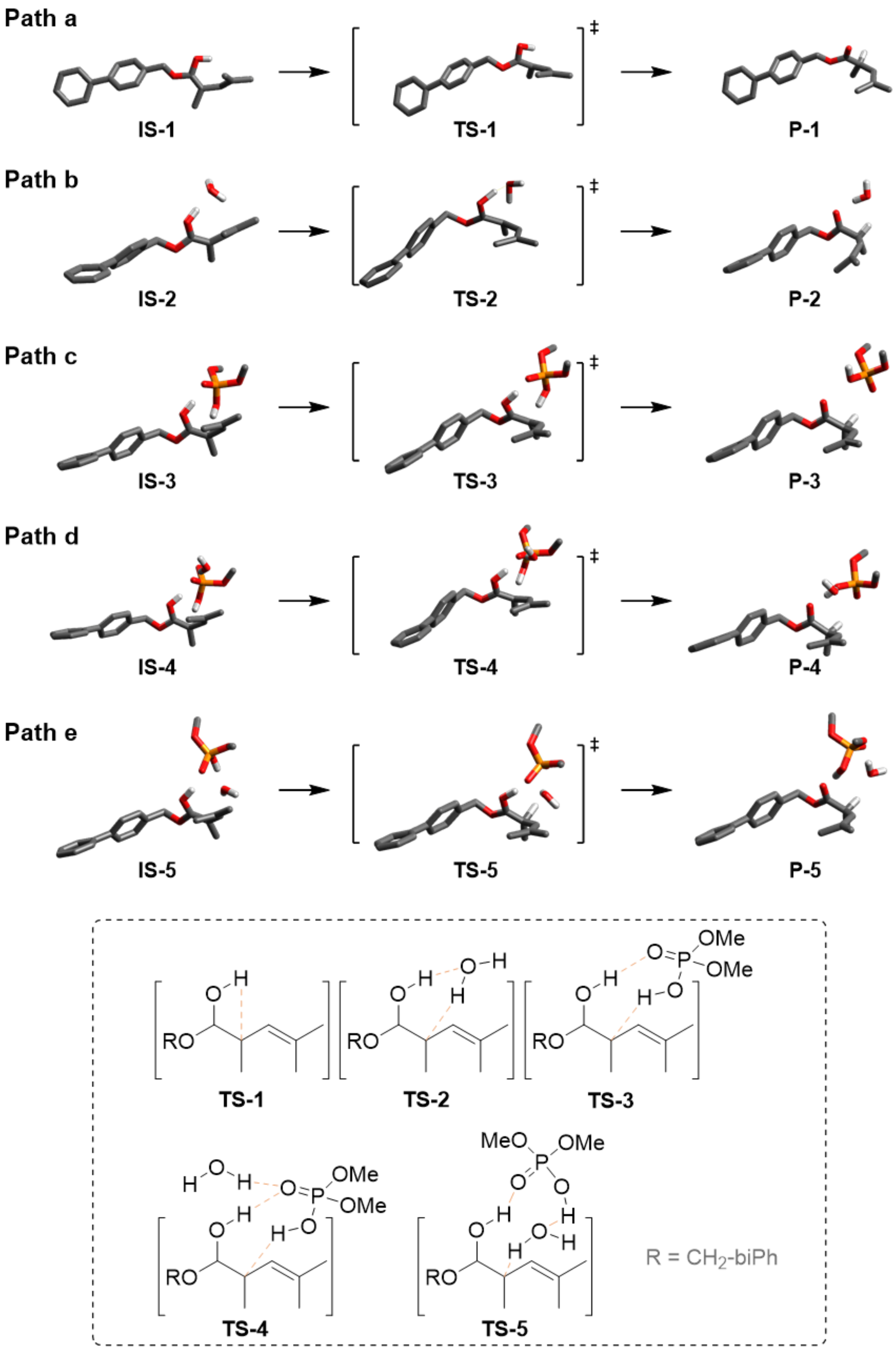

Figure S6.10 Optimized initial state (enol form), transition state (TS), and product state (keto form) of the considered protonation pathways. The non-reacting hydrogen atoms were removed for clarity. Black: C; red: O; white: H; orange: P.

in the proton transfer step with DMP (**Path d**), which gave a 7 kJ/mol ΔE_a value. When considering the ΔG_a values, the barrier for **Paths c** and **d** were comparable, with the latter 1 kJ/mol more favorable.

In our calculations under toluene, similar trends from gaseous phase calculations were observed. Nevertheless, a 4 kJ/mol difference in ΔE_a was now obtained from **Paths c** and **d**, favoring DMP with spectator water, an increase to the 1 kJ/mol difference in the results from gaseous phase calculations. However, ΔG_a values for both pathways were calculated to be 14 kJ/mol, which indicated that both pathways have similar probability. Given the small difference obtained in the calculated barriers, the current methods applied were not necessarily able to explain the preference of DMP with spectator water as the active proton shuttle system over DMP alone. To understand the effect of water on the reaction under study, a larger model that considers bulk water molecules and their dynamic behavior is probably needed.

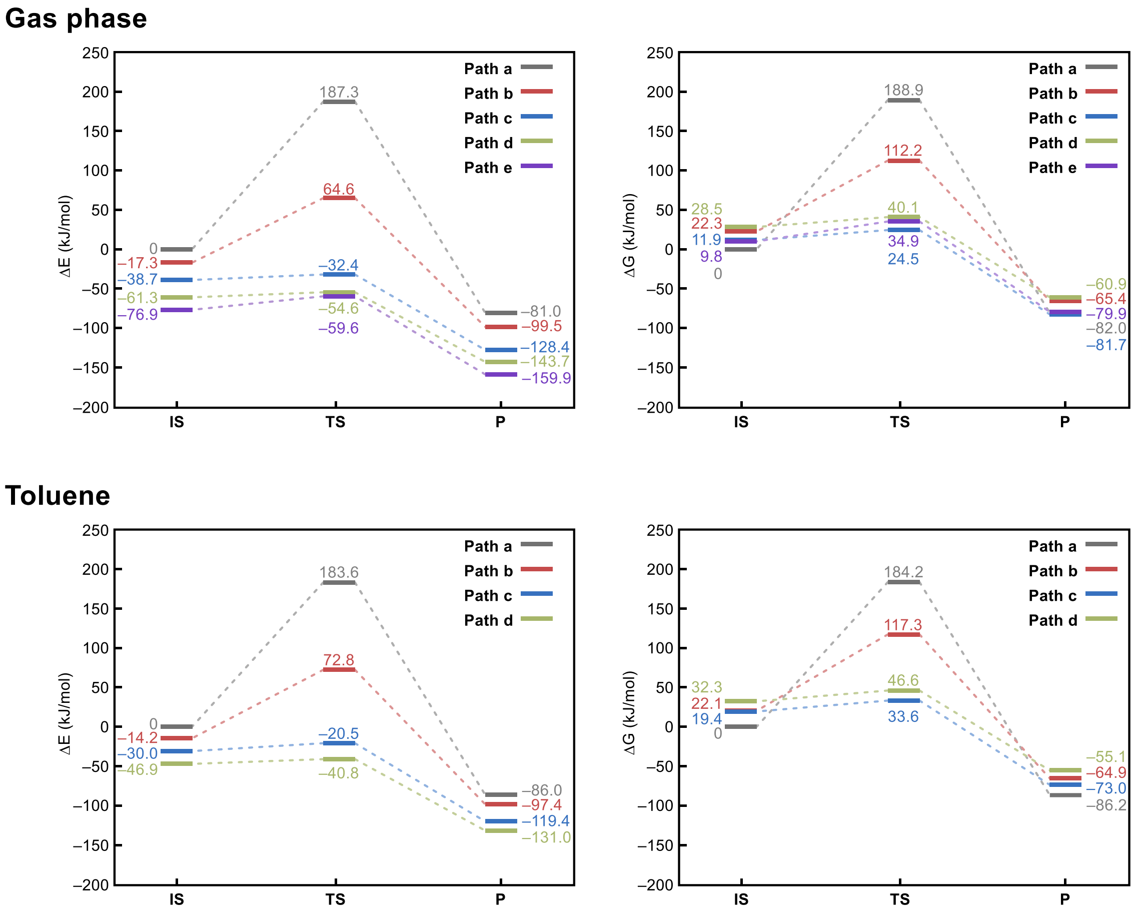

Figure S6.11 The energy and Gibbs free energy profile diagrams for the computed reaction pathways in gaseous and toluene.

DFT Optimized Coordinates

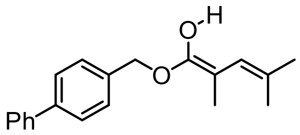

IS-1 (PCM = toluene)

Gibbs free energy = -925.572466 Hartree

C	6.967458	-0.678903	-0.273829	C	-5.860037	-0.342975	1.513410
C	7.425160	0.636559	-0.155342	H	7.673458	-1.489083	-0.434533
C	5.603711	-0.961368	-0.175504	H	8.486221	0.856459	-0.231635
C	4.666154	0.064723	0.043015	H	6.848188	2.694248	0.146630
C	6.505470	1.666620	0.061983	H	4.436533	2.196950	0.304439
C	5.141780	1.383775	0.159503	H	5.264886	-1.990863	-0.246618
C	3.212856	-0.235735	0.146878	H	3.211667	-1.731972	-1.411854
C	2.612304	-1.207510	-0.673630	H	0.808338	-2.240128	-1.224064
C	1.250445	-1.490359	-0.572624	H	2.835700	1.177354	1.737759
C	0.441398	-0.811920	0.348344	H	0.427089	0.688641	1.896664
C	2.396509	0.440726	1.071582	H	-1.406737	-0.924059	1.453395
C	1.034674	0.157219	1.168256	H	-1.244595	-2.139384	0.162604
C	-1.031121	-1.103610	0.438684	H	-2.131924	2.194817	-0.674277
O	-1.726899	-0.210682	-0.477392	H	-3.519487	2.697318	-1.653996
C	-3.081151	-0.246713	-0.451616	H	-3.560548	2.916268	0.094461
O	-3.563996	-1.524570	-0.411550	H	-8.096018	-0.768070	-0.163112
H	4.529693	-1.478438	-0.506749	H	-8.254428	0.806759	0.604468
C	-3.841479	0.867266	-0.550950	H	-7.890660	0.703792	-1.131946
C	-3.222618	2.237948	-0.700182	H	-6.493248	0.099194	2.293777
C	-5.312549	0.781757	-0.686701	H	-4.814691	-0.171902	1.781184
C	-6.213986	0.248294	0.170215	H	-6.049203	-1.425517	1.545411
C	-7.688064	0.252063	-0.156063	H	-5.715183	1.262484	-1.583039

IS-1 (gas phase)

Gibbs free energy = -925.55197 Hartree

C	6.967458	-0.678903	-0.273829	C	5.603711	-0.961368	-0.175504
C	7.425160	0.636559	-0.155342	C	4.666154	0.064723	0.043015

C	6.505470	1.666620	0.061983	H	8.486221	0.856459	-0.231635
C	5.141780	1.383775	0.159503	H	6.848188	2.694248	0.146630
C	3.212856	-0.235735	0.146878	H	4.436533	2.196950	0.304439
C	2.612304	-1.207510	-0.673630	H	5.264886	-1.990863	-0.246618
C	1.250445	-1.490359	-0.572624	H	3.211667	-1.731972	-1.411854
C	0.441398	-0.811920	0.348344	H	0.808338	-2.240128	-1.224064
C	2.396509	0.440726	1.071582	H	2.835700	1.177354	1.737759
C	1.034674	0.157219	1.168256	H	0.427089	0.688641	1.896664
C	-1.031121	-1.103610	0.438684	H	-1.406737	-0.924059	1.453395
O	-1.726899	-0.210682	-0.477392	H	-1.244595	-2.139384	0.162604
C	-3.081151	-0.246713	-0.451616	H	-2.131924	2.194817	-0.674277
O	-3.563996	-1.524570	-0.411550	H	-3.519487	2.697318	-1.653996
H	4.529693	-1.478438	-0.506749	H	-3.560548	2.916268	0.094461
C	-3.841479	0.867266	-0.550950	H	-8.096018	-0.768070	-0.163112
C	-3.222618	2.237948	-0.700182	H	-8.254428	0.806759	0.604468
C	-5.312549	0.781757	-0.686701	H	-7.890660	0.703792	-1.131946
C	-6.213986	0.248294	0.170215	H	-6.493248	0.099194	2.293777
C	-7.688064	0.252063	-0.156063	H	-4.814691	-0.171902	1.781184
C	-5.860037	-0.342975	1.513410	H	-6.049203	-1.425517	1.545411
H	7.673458	-1.489083	-0.434533	H	-5.715183	1.262484	-1.583039

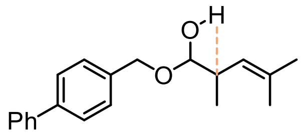

TS-1 (PCM = toluene)

Gibbs free energy = -925.502323 Hartree

C	-6.798772	-0.620737	0.283483	C	-3.082118	-0.067398	-0.304207
C	-7.208553	0.641616	0.722123	C	-2.453103	-1.285518	0.012460
C	-5.462531	-0.850751	-0.049085	C	-1.119142	-1.512544	-0.321986
C	-4.506065	0.176182	0.049633	C	-0.368011	-0.529940	-0.981556
C	-6.269208	1.671657	0.825271	C	-2.323655	0.912673	-0.969734
C	-4.933059	1.441365	0.492851	C	-0.989158	0.684355	-1.301832

C	1.072364	-0.767779	-1.324995
O	1.901379	-0.320621	-0.185812
C	3.135134	-0.761257	-0.100251
O	3.680090	-1.479842	-1.037125
H	4.364166	-1.724045	-0.010689
C	3.966990	-0.703131	1.070994
C	3.298543	-0.739066	2.437551
C	5.272073	0.006237	1.019564
C	5.747041	0.943073	0.167311
C	7.168955	1.438238	0.289714
C	4.959905	1.530303	-0.976722
H	-7.521433	-1.426835	0.191268
H	-8.248268	0.820617	0.980696
H	-6.574168	2.654598	1.173624
H	-4.209603	2.244287	0.600645
H	-5.163406	-1.830339	-0.410458
H	-3.007250	-2.054711	0.541980
H	-0.654745	-2.460160	-0.060569
H	-2.788761	1.852217	-1.251980
H	-0.427681	1.454913	-1.824229
H	1.385154	-0.188160	-2.196294
H	1.292833	-1.822700	-1.500894
H	2.445515	-1.425521	2.461251
H	4.017520	-1.079570	3.191478
H	2.942322	0.251681	2.756510
H	7.751612	1.205839	-0.614143
H	7.201661	2.530651	0.406515
H	7.687392	0.988697	1.142914
H	5.251922	2.573101	-1.151363
H	3.881477	1.517857	-0.788321
H	5.134393	0.987787	-1.918001
H	5.941827	-0.311973	1.820025

TS-1 (gas phase)

Gibbs free energy = -925.480026 Hartree

C	6.804380	-0.636555	-0.255400
C	7.222134	0.626465	-0.683610
C	5.464895	-0.862790	0.065182
C	4.514029	0.168238	-0.035802
C	6.288104	1.660750	-0.788520
C	4.948711	1.433753	-0.468226
C	3.087324	-0.072555	0.304364
C	2.449111	-1.273134	-0.054819
C	1.112084	-1.498848	0.267216
C	0.366970	-0.532626	0.956110
C	2.335436	0.891649	0.998792
C	0.997863	0.664465	1.318372
C	-1.077780	-0.769893	1.284067
O	-1.897062	-0.259821	0.171164
C	-3.118663	-0.730286	0.033150
O	-3.646297	-1.541209	0.900801
H	-4.313874	-1.718787	-0.157317
C	-3.940358	-0.595960	-1.136754
C	-3.266120	-0.489404	-2.496632
C	-5.275204	0.048354	-1.031827
C	-5.789066	0.879063	-0.097694
C	-7.230179	1.324829	-0.179326
C	-5.021570	1.384237	1.098378
H	7.523351	-1.445461	-0.161724

H	8.264290	0.802764	-0.933057
H	6.599893	2.643968	-1.129223
H	4.227698	2.238447	-0.578518
H	5.157301	-1.841818	0.420817
H	2.999357	-2.027179	-0.609372
H	0.640040	-2.432739	-0.027636
H	2.810132	1.815917	1.313820
H	0.440666	1.422037	1.863689
H	-1.386506	-0.229049	2.181775
H	-1.313307	-1.828316	1.413382
H	-2.379659	-1.127781	-2.567124
H	-3.965512	-0.804393	-3.278989
H	-2.960027	0.540057	-2.731681
H	-7.805662	0.976597	0.690795
H	-7.307120	2.420903	-0.184435
H	-7.724495	0.943562	-1.078264
H	-5.377123	2.377779	1.395571
H	-3.949059	1.463567	0.892037
H	-5.137634	0.723028	1.969806
H	-5.928480	-0.223370	-1.862194

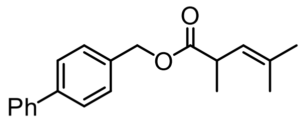

P-1 (PCM = toluene)

Gibbs free energy = -925.605287 Hartree

C	-6.749217	-0.328713	0.266513
C	-7.053945	1.024912	0.436182
C	-5.430816	-0.731106	0.044761
C	-4.387022	0.210536	-0.011514
C	-6.027351	1.972087	0.381903
C	-4.709032	1.569211	0.161009
C	-2.981702	-0.217544	-0.245060
C	-2.474382	-1.392382	0.338976
C	-1.157284	-1.793190	0.117823
C	-0.300893	-1.034685	-0.691530
C	-2.119242	0.537529	-1.060374
C	-0.802323	0.134671	-1.278855
C	1.123444	-1.458854	-0.915307
O	1.946502	-0.862657	0.133837
C	3.269106	-1.127435	0.078347
O	3.767544	-1.818245	-0.791468
H	4.386534	-1.423760	1.791565
C	4.053674	-0.528107	1.249154
C	3.219958	0.316578	2.231039
C	5.322361	0.187140	0.799185
C	5.441740	1.170937	-0.108161
C	6.788722	1.783396	-0.408986
C	4.302139	1.739700	-0.917080
H	-7.539819	-1.073415	0.297857
H	-8.079726	1.338004	0.608555
H	-6.250511	3.026362	0.520863
H	-3.918314	2.313694	0.145378
H	-5.212300	-1.783772	-0.109048
H	-3.110157	-1.987566	0.987713
H	-0.787990	-2.702233	0.586294

H	-2.489397	1.433888	-1.549078	H	7.045529	1.659084	-1.469864
H	-0.159970	0.730526	-1.922608	H	6.782474	2.864243	-0.212940
H	1.501406	-1.119697	-1.883385	H	7.587063	1.332418	0.187918
H	1.237580	-2.544989	-0.863739	H	4.277351	2.833546	-0.825924
H	2.372298	-0.248120	2.629799	H	3.321724	1.362312	-0.620496
H	3.853793	0.622713	3.069672	H	4.441664	1.517474	-1.983893
H	2.833510	1.222503	1.755961	H	6.227270	-0.134075	1.312309

P-1 (gas phase)

Gibbs free energy = -925.583201 Hartree

C	-6.749152	-0.351962	0.259879	C	4.317529	1.715705	-0.926121
C	-7.063222	0.997559	0.442348	H	-7.534715	-1.101980	0.282334
C	-5.427921	-0.742574	0.035973	H	-8.091172	1.301836	0.616133
C	-4.391282	0.206578	-0.009220	H	-6.274478	3.003214	0.548643
C	-6.043482	1.952274	0.399254	H	-3.935395	2.309114	0.170920
C	-4.722390	1.560648	0.176579	H	-5.200254	-1.791514	-0.129791
C	-2.983936	-0.209159	-0.246127	H	-3.090714	-1.969929	0.999235
C	-2.462991	-1.375165	0.342409	H	-0.763505	-2.667859	0.588261
C	-1.143977	-1.765400	0.116327	H	-2.516280	1.437554	-1.561891
C	-0.298486	-1.004276	-0.701472	H	-0.179581	0.754491	-1.942180
C	-2.133083	0.548655	-1.069759	H	1.508317	-1.062575	-1.891471
C	-0.813618	0.156727	-1.292121	H	1.251252	-2.503526	-0.897871
C	1.128490	-1.417457	-0.929446	H	2.365052	-0.250347	2.627076
O	1.944557	-0.835372	0.128953	H	3.851695	0.609325	3.082224
C	3.266976	-1.122131	0.079195	H	2.841875	1.220493	1.765740
O	3.758294	-1.813774	-0.790237	H	7.066886	1.617087	-1.460428
H	4.381486	-1.433433	1.792741	H	6.799505	2.839248	-0.220365
C	4.052246	-0.533330	1.255408	H	7.594401	1.309285	0.204923
C	3.219734	0.311032	2.239968	H	4.304391	2.811095	-0.857756
C	5.324253	0.177623	0.810103	H	3.334815	1.352837	-0.620060
C	5.451614	1.152078	-0.104650	H	4.454818	1.467357	-1.987076
C	6.803254	1.756435	-0.403439	H	6.225463	-0.141765	1.330407

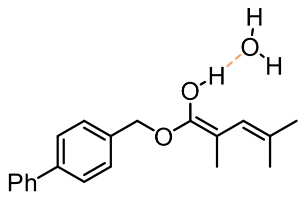

IS-2 (PCM = toluene)

Gibbs free energy = -1001.999936 Hartree

C	7.230111	-0.413991	-0.803720	H	7.250571	2.446952	1.035905
C	7.735638	0.728327	-0.176373	H	4.850388	1.897171	1.197948
C	5.873874	-0.730131	-0.703653	H	5.499248	-1.633574	-1.176084
C	4.991734	0.088931	0.024665	H	3.353044	-0.917280	-1.914340
C	6.871239	1.551556	0.551122	H	0.964099	-1.488664	-1.730774
C	5.514905	1.235660	0.649777	H	3.364467	0.328712	2.205439
C	3.546590	-0.247203	0.130609	H	0.969085	-0.219522	2.374154
C	2.842092	-0.774209	-0.966719	H	-0.932129	-1.550689	1.450361
C	1.487679	-1.090939	-0.864707	H	-0.953172	-2.009544	-0.266965
C	0.790257	-0.894093	0.334481	H	-2.749650	2.421042	-1.821430
C	2.842233	-0.051670	1.332469	H	-1.675436	1.035204	-2.055429
C	1.488328	-0.370584	1.431015	H	-3.195590	1.158748	-2.966302
C	-0.674815	-1.219950	0.441257	H	-7.854397	0.443170	0.422713
O	-1.427817	-0.017345	0.123263	H	-7.649723	2.135246	-0.004680
C	-2.779423	-0.138180	0.022856	H	-7.531044	0.892126	-1.265812
O	-3.290988	-0.984012	0.950206	H	-5.930386	2.195721	1.838711
C	-3.462958	0.583105	-0.902865	H	-4.310906	1.523636	1.539531
C	-2.723312	1.335264	-1.989907	H	-5.614638	0.488887	2.135643
C	-4.933289	0.697534	-0.945645	H	-5.359110	0.596094	-1.948834
C	-5.799442	1.022273	0.044351	H	-4.142353	-1.379371	0.656719
C	-7.282731	1.123299	-0.224549	O	-5.609423	-2.269769	0.138351
C	-5.381870	1.323801	1.459876	H	-5.509371	-3.070460	-0.395382
H	7.893550	-1.066179	-1.365152	H	-5.962681	-1.585369	-0.452935
H	8.790927	0.974148	-0.253599				

Back to contents

IS-2 (gas phase)

Gibbs free energy = -1001.974630 Hartree

C	6.326552	-0.050857	-0.323067	H	5.466585	2.840893	1.246665
C	6.479084	1.207780	0.264888	H	3.239200	1.783750	1.129133
C	5.068409	-0.652571	-0.380298	H	4.967641	-1.642078	-0.816490
C	3.934612	-0.009236	0.147299	H	2.852364	-1.442827	-1.907636
C	5.361543	1.858711	0.794572	H	0.638700	-2.518739	-2.000231
C	4.103584	1.256841	0.735817	H	1.991233	-0.027494	2.062345
C	2.594079	-0.648455	0.084031	H	-0.243116	-1.068601	1.946759
C	2.187648	-1.369962	-1.052063	H	-1.658492	-2.863158	0.870700
C	0.930768	-1.971100	-1.107347	H	-1.329406	-3.339944	-0.820441
C	0.036723	-1.870397	-0.033172	H	-3.674986	-0.249861	-2.543813
C	1.693604	-0.554048	1.160504	H	4.863213	-1.374106	-1.871154
C	0.436427	-1.153539	1.104153	H	-5.304874	0.318693	-2.159663
C	-1.323053	-2.514128	-0.106922	H	-1.458807	3.814077	-0.070207
O	-2.331837	-1.592747	-0.634766	H	-2.485068	4.230387	-1.438949
C	-3.058971	-0.883955	0.245646	H	-3.204159	4.029189	0.172304
O	-2.910846	-0.981930	1.456977	H	-1.726090	2.057771	-2.749013
C	-4.085925	0.042279	-0.400371	H	-1.730006	0.505897	-1.888011
C	-4.497150	-0.342864	-1.832619	H	-0.622206	1.792617	-1.405692
C	-3.668592	1.506272	-0.230339	H	-4.298450	2.059631	0.462235
C	-2.651979	2.170227	-0.807448	H	-4.187994	-0.080991	2.541486
C	-2.447892	3.639141	-0.514080	O	-4.961509	0.401258	2.891029
C	-1.639189	1.584031	-1.761641	H	-4.965123	-0.081682	0.240994
H	7.189606	-0.570457	-0.729627	H	-5.099428	0.073376	3.787007
H	7.457942	1.675990	0.310121				

Back to contents

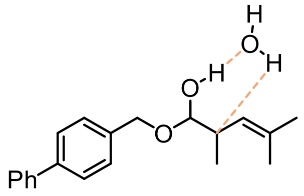

TS-2 (PCM = toluene)

Gibbs free energy = -1002.033065 Hartree

C	6.586713	0.022114	-0.588385	H	5.817113	3.066148	0.719604
C	6.772685	1.334523	-0.144614	H	3.589348	2.014470	0.845551
C	5.327990	-0.576443	-0.508211	H	5.204011	-1.604566	-0.835701
C	4.226032	0.123406	0.016712	H	3.045180	-1.555492	-1.786302
C	5.687241	2.042406	0.379474	H	0.835079	-2.626947	-1.633914
C	4.428441	1.443874	0.458436	H	2.375178	0.352504	2.013842
C	2.884448	-0.513671	0.100764	H	0.142992	-0.682108	2.135539
C	2.423608	-1.370945	-0.915304	H	-1.392286	-2.446167	1.361476
C	1.168167	-1.970981	-0.833194	H	-1.019476	-3.370317	-0.118648
C	0.325948	-1.730713	0.261701	H	-3.728617	-0.808079	-2.726355
C	2.037848	-0.282198	1.199771	H	-4.263703	-2.187675	-1.751493
C	0.778966	-0.877804	1.277182	H	-5.395558	-0.888374	-2.151647
C	-1.032703	-2.376927	0.334761	H	-4.150906	4.096030	0.494169
O	-2.021316	-1.648635	-0.466642	H	-3.913626	4.204396	-1.245692
C	-2.840295	-0.773105	0.120751	H	-5.410685	3.497428	-0.601572
O	-2.645471	-0.479482	1.365697	H	-1.734532	3.097310	-0.759595
C	-4.001292	-0.351319	-0.596372	H	-1.682585	1.332796	-0.634704
C	-4.356886	-1.104078	-1.874917	H	-1.906451	2.316495	0.813346
C	-4.405160	1.088226	-0.604062	H	-5.471318	1.231900	-0.793698
C	-3.666620	2.208236	-0.448084	H	-3.754304	-0.430010	1.811270
C	-4.328292	3.566632	-0.453093	O	-4.976166	-0.642732	1.822569
C	-2.172705	2.230829	-0.249429	H	-4.804435	-0.651308	0.628355
H	7.424480	-0.541216	-0.990129	H	-5.478142	0.156973	2.036761
H	7.752051	1.800233	-0.206784				

TS-2 (gas phase)

Gibbs free energy = -1001.940414 Hartree

C	6.613448	-0.006776	-0.419470	H	5.735711	3.169494	0.420967
C	6.759022	1.355317	-0.142583	H	3.511187	2.105311	0.540214
C	5.357078	-0.609956	-0.341761	H	5.261289	-1.674506	-0.534440
C	4.218088	0.134730	0.012985	H	3.148490	-1.735482	-1.662001
C	5.636171	2.108049	0.212130	H	0.938506	-2.814098	-1.511107
C	4.380004	1.504324	0.288273	H	2.266885	0.550765	1.873345
C	2.879663	-0.506941	0.093727	H	0.033487	-0.496151	1.989331
C	2.480082	-1.471474	-0.847905	H	-1.389577	-2.448730	1.353403
C	1.225955	-2.075093	-0.767142	H	-1.005701	-3.383767	-0.120338
C	0.327356	-1.734019	0.252469	H	-3.737157	-0.827241	-2.731861
C	1.974661	-0.170990	1.116349	H	-4.252329	-2.204686	-1.745597
C	0.719173	-0.772217	1.194026	H	-5.398012	-0.913908	-2.140075
C	-1.028606	-2.387524	0.326225	H	-4.197930	4.072057	0.520284
O	-2.018894	-1.672328	-0.477616	H	-3.922282	4.197387	-1.212675
C	-2.826464	-0.781420	0.111018	H	-5.426604	3.471235	-0.608780
O	-2.613347	-0.475303	1.348119	H	-1.747768	3.120421	-0.659640
C	-3.989195	-0.362378	-0.603154	H	-1.686400	1.350325	-0.640243
C	-4.355577	-1.123247	-1.874072	H	-1.932783	2.238486	0.861149
C	-4.402397	1.072089	-0.608447	H	-5.465641	1.209605	-0.819135
C	-3.676094	2.196235	-0.428793	H	-3.739529	-0.422790	1.816688
C	-4.348940	3.549647	-0.435070	O	-4.936421	-0.622379	1.833047
C	-2.184674	2.222167	-0.208048	H	-4.787849	-0.652858	0.647035
H	7.480503	-0.603929	-0.687461	H	-5.426205	0.185126	2.040501
H	7.736476	1.824870	-0.202501				

Back to contents

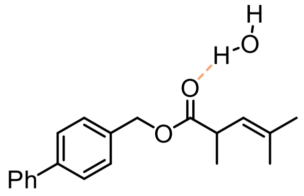

P-2 (PCM = toluene)

Gibbs free energy = -1001.963654 Hartree

C	6.448963	0.094997	-0.447436	H	5.502970	3.213032	0.528845
C	6.562009	1.451694	-0.130396	H	3.319574	2.075078	0.691512
C	5.215321	-0.550972	-0.347023	H	5.147768	-1.610864	-0.574238
C	4.066701	0.144939	0.072171	H	3.008172	-1.739215	-1.602731
C	5.429404	2.156116	0.287865	H	0.837257	-2.881730	-1.413215
C	4.195911	1.510008	0.387613	H	2.152833	0.468616	1.992702
C	2.750793	-0.540589	0.178116	H	-0.037458	-0.644920	2.152151
C	2.354341	-1.503296	-0.768444	H	-1.482554	-2.526508	1.496020
C	1.120808	-2.144414	-0.665992	H	-1.040823	-3.557826	0.107461
C	0.237757	-1.843014	0.380728	H	-2.856226	-0.470783	-2.737596
C	1.864185	-0.248580	1.230147	H	-3.661702	-2.020339	-2.430103
C	0.628068	-0.887090	1.328552	H	-4.607535	-0.607534	-2.934729
C	-1.097967	-2.531588	0.475711	H	-3.451149	3.909509	0.209264
O	-2.092668	-1.906639	-0.402103	H	-3.696884	4.011849	-1.532504
C	-2.937715	-1.007494	0.126462	H	-5.020920	3.460903	-0.482353
O	-2.901515	-0.675694	1.303472	H	-1.636539	2.479396	-1.848601
C	-3.995853	-0.520050	-0.864169	H	-1.624798	0.845223	-1.178693
C	-3.757542	-0.935767	-2.328392	H	-1.418578	2.244404	-0.117304
C	-4.304843	0.964605	-0.715640	H	-5.356165	1.194486	-0.554660
C	-3.448026	1.996980	-0.790607	H	-4.139456	0.376511	2.325654
C	-3.939747	3.416445	-0.641876	O	-4.745570	0.790272	2.969083
C	-1.959494	1.867933	-0.995746	H	-4.893694	-1.054202	-0.520071
H	7.324082	-0.465145	-0.765229	H	-4.570352	0.337005	3.803499
H	7.521751	1.954344	-0.208567				

P-2 (gas phase)

Gibbs free energy = -1002.008071 Hartree

C	7.061800	0.075397	0.234321
C	7.237448	1.366247	-0.271804
C	5.779971	-0.463005	0.358883
C	4.644738	0.274781	-0.021834
C	6.118708	2.112178	-0.652996
C	4.837499	1.571962	-0.530013
C	3.279983	-0.299838	0.107576
C	3.028404	-1.646898	-0.198537
C	1.744051	-2.181228	-0.079136
C	0.673024	-1.391873	0.353363
C	2.197548	0.489993	0.539344
C	0.917502	-0.045102	0.661283
C	-0.710601	-1.977564	0.490960
O	-1.612983	-1.421483	-0.498185
C	-2.706426	-0.726613	-0.086352
O	-2.459280	0.028630	1.013713
C	-3.862605	-0.797237	-0.796704
C	-4.026924	-1.842852	-1.881407
C	-5.024961	0.086389	-0.597657
C	-5.076699	1.437978	-0.502121
C	-6.398534	2.155774	-0.352335
C	-3.861098	2.325944	-0.567644
H	7.923122	-0.510805	0.542315
H	8.234534	1.785934	-0.368146
H	6.243148	3.113347	-1.056106
H	3.978138	2.151754	-0.853819
H	5.654779	-1.457276	0.777687
H	3.836518	-2.275092	-0.561637
H	1.572882	-3.223556	-0.337634
H	2.368096	1.528695	0.807182
H	0.096552	0.579451	1.000906
H	-1.113490	-1.794613	1.489389
H	-0.685584	-3.056644	0.306920
H	-3.995511	-1.394127	-2.883437
H	-3.245701	-2.603535	-1.833046
H	-5.002551	-2.339242	-1.788604
H	-6.421545	2.770752	0.557996
H	-6.561243	2.844389	-1.192291
H	-7.243358	1.460354	-0.315881
H	-4.079418	3.224252	-1.157868
H	-3.006785	1.810697	-1.011851
H	-3.555318	2.667416	0.430911
H	-5.988172	-0.432176	-0.641313
H	-3.270443	0.170083	1.551824
O	-4.739033	0.368523	2.537451
H	-5.038675	-0.351028	3.107536
H	-5.326565	0.379005	1.761988

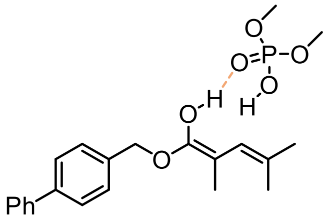

IS-3 (PCM = toluene)

Gibbs free energy = -1648.279972 Hartree

C	-8.310333	-1.588020	0.394329	H	-6.385679	-2.373934	-0.152072
C	-9.063718	-0.444398	0.674586	H	-4.277867	-1.871377	0.823995
C	-6.952776	-1.478972	0.086969	H	-1.886444	-1.682285	0.265170
C	-6.316977	-0.224322	0.053616	H	-5.086545	1.683698	-1.463853
C	-8.445757	0.809130	0.644999	H	-2.690100	1.881897	-1.996208
C	-7.088058	0.917550	0.338444	H	-0.497885	0.715046	-2.200387
C	-4.870628	-0.106861	-0.273486	H	-0.157668	-0.734498	-1.232096
C	-3.940910	-1.052904	0.194741	H	0.877874	2.482179	2.569237
C	-2.585893	-0.941904	-0.115541	H	0.311248	0.836786	2.255246
C	-2.112353	0.115616	-0.903446	H	1.846523	1.112069	3.105519
C	-4.390133	0.952282	-1.064675	H	5.987971	3.115873	-0.396149
C	-3.034673	1.059770	-1.373761	H	5.528370	4.323394	0.798696
C	-0.648846	0.243944	-1.226489	H	5.861927	2.654902	1.312680
O	-0.036151	1.074590	-0.196500	H	3.575235	4.778613	-0.705883
C	1.312068	1.195093	-0.193548	H	2.171280	3.690444	-0.662574
O	1.866588	1.047666	-1.412255	H	3.515049	3.367615	-1.762083
C	1.963156	1.470995	0.978451	H	3.960725	1.503809	1.833522
C	1.201809	1.469138	2.292432	H	2.750914	0.605072	-1.364996
C	3.360024	1.953698	1.039331	O	5.366228	-1.517284	0.745264
C	3.954854	2.923099	0.308964	O	3.918980	-3.096289	-0.675232
C	5.408453	3.265931	0.525869	P	4.037673	-1.586159	-0.158639
C	3.259288	3.728977	-0.756140	O	3.999089	-0.621803	-1.298173
H	-8.781266	-2.567159	0.405782	O	2.878050	-1.441332	0.928163
H	-10.120069	-0.529059	0.913282	H	2.500530	-0.521139	0.966549
H	-9.018554	1.704685	0.869791	C	3.890216	-4.214965	0.244004
H	-6.615697	1.895531	0.342321	H	2.987661	-4.176098	0.859288

H	4.780094	-4.216744	0.879688
H	3.877638	-5.111119	-0.377274
C	6.662785	-1.414944	0.106818
H	7.390723	-1.363353	0.917384
H	6.714974	-0.509187	-0.501826
H	6.862089	-2.295264	-0.512087

IS-3 (gas phase)

Gibbs free energy = -1648.25367 Hartree

C	-8.472608	-0.783402	-0.489259
C	-8.797710	0.495135	-0.950677
C	-7.148847	-1.106453	-0.186365
C	-6.120387	-0.158900	-0.335691
C	-7.786163	1.447063	-1.105264
C	-6.462985	1.123485	-0.800526
C	-4.711051	-0.501676	-0.010376
C	-4.396561	-1.292938	1.106786
C	-3.071958	-1.611883	1.408493
C	-2.019823	-1.154101	0.606673
C	-3.649274	-0.044251	-0.812759
C	-2.327232	-0.363807	-0.510842
C	-0.591593	-1.503832	0.943435
O	0.087601	-0.392714	1.591702
C	1.059983	0.283283	0.936959
O	0.946611	0.238885	-0.404292
C	2.016473	0.947811	1.657449
C	2.075620	0.791930	3.167618
C	2.900784	1.979989	1.075822
C	2.583503	3.003101	0.252751
C	3.650575	3.951118	-0.239011
C	1.188803	3.292677	-0.237026
H	-9.248972	-1.534555	-0.373705
H	-9.827453	0.746721	-1.187135
H	-8.027873	2.446887	-1.454849
H	-5.689784	1.879561	-0.900512
H	-6.905319	-2.111114	0.146745
H	-5.191786	-1.640166	1.759844
H	-2.854215	-2.217046	2.285335
H	-3.864973	0.546612	-1.698172
H	-1.522720	-0.002975	-1.144479
H	-0.034279	-1.796604	0.052396
H	-0.561450	-2.317203	1.673809
H	1.531794	1.601182	3.673337
H	1.644637	-0.155027	3.501089
H	3.114837	0.844116	3.515373
H	3.713726	3.935913	-1.336168
H	3.418464	4.986664	0.044037
H	4.637518	3.699026	0.160727
H	1.011372	4.374708	-0.263358
H	0.427077	2.830049	0.394625
H	1.037376	2.916054	-1.258060
H	3.937431	1.933613	1.418397
H	1.829988	0.221191	-0.854612
O	5.744164	-0.147614	-0.571969
O	4.745062	-2.411526	-1.253613
P	4.358428	-0.960971	-0.695200
O	3.328444	-0.301609	-1.548948
O	3.970398	-1.218424	0.828339
H	3.290478	-0.572602	1.165474
C	5.660314	-3.272781	-0.541678
H	5.243529	-3.541738	0.432073

H	6.628556	-2.780875	-0.411620	H	7.151406	1.072725	-1.391317
H	5.774311	-4.163088	-1.160309	H	5.547891	1.170111	-2.184140
C	6.290399	0.502689	-1.741141	H	6.612203	-0.239094	-2.478791

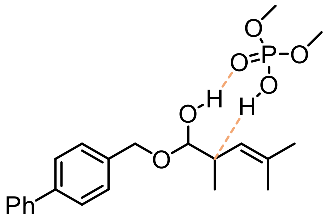

TS-3 (PCM = toluene)

Gibbs free energy = -1648.274594 Hartree

C	-8.282614	-0.308862	-0.129319	H	-9.139088	-0.802607	0.321513
C	-8.472168	0.767067	-1.001282	H	-9.474624	1.120065	-1.225829
C	-6.994181	-0.762582	0.159016	H	-7.494652	2.227357	-2.254491
C	-5.865845	-0.150580	-0.417059	H	-5.217469	1.436549	-1.732481
C	-7.360626	1.384812	-1.581660	H	-6.863671	-1.614364	0.820113
C	-6.072287	0.931364	-1.292448	H	-4.888039	-1.023246	1.980626
C	-4.492949	-0.633096	-0.108810	H	-2.624279	-1.847533	2.480887
C	-4.148875	-1.063666	1.186140	H	-3.740963	-0.377012	-2.117746
C	-2.864315	-1.522143	1.471583	H	-1.461519	-1.152107	-1.598998
C	-1.875972	-1.557682	0.477228	H	0.059396	-2.335191	-0.097925
C	-3.499367	-0.678095	-1.102763	H	-0.524289	-2.898292	1.489175
C	-2.211192	-1.128584	-0.813502	H	1.217871	0.831462	3.673425
C	-0.490683	-2.052587	0.799485	H	1.979384	-0.756963	3.461467
O	0.284361	-1.050867	1.543894	H	2.978524	0.696752	3.607267
C	1.107516	-0.220853	0.912572	H	2.173624	4.385514	-0.741668
O	1.029940	-0.222578	-0.396806	H	1.889649	5.013434	0.878638
C	2.047176	0.503565	1.673136	H	3.419763	4.189019	0.505464
C	2.047496	0.300286	3.188134	H	-0.260936	3.710698	0.800482
C	2.471686	1.869034	1.223081	H	-0.245275	1.956913	1.036027
C	1.713067	2.863816	0.720738	H	-0.002551	2.641889	-0.576336
C	2.340617	4.178841	0.324751	H	3.529853	2.084222	1.376646
C	0.226038	2.777887	0.489798	H	1.924252	0.007637	-0.858270

O	5.733360	0.473536	-0.740473	H	5.715585	-3.006380	0.222076
O	4.963204	-1.892110	-1.383748	H	6.938796	-2.072944	-0.693653
P	4.457240	-0.517131	-0.706269	H	6.187705	-3.519515	-1.424183
O	3.292194	0.010079	-1.512671	C	6.164996	1.036497	-1.998493
O	4.192499	-0.776999	0.802187	H	7.022575	1.670613	-1.767585
H	3.199223	-0.257689	1.209770	H	5.366900	1.637310	-2.442731
C	6.019533	-2.662154	-0.770426	H	6.468876	0.249172	-2.696104

TS-3 (gas phase)

Gibbs free energy = -1648.248881 Hartree

C	-8.290720	-0.371888	-0.288078	H	-9.152348	-0.962566	0.009971
C	-8.470976	0.851929	-0.938376	H	-9.471670	1.222249	-1.140686
C	-7.004411	-0.847645	-0.028662	H	-7.480817	2.551951	-1.824694
C	-5.869963	-0.110448	-0.412329	H	-5.206446	1.718027	-1.349388
C	-7.352842	1.595192	-1.326349	H	-6.877595	-1.812012	0.454539
C	-6.066906	1.119255	-1.065591	H	-4.971006	-1.376462	1.831841
C	-4.499998	-0.615655	-0.134007	H	-2.705866	-2.235217	2.279597
C	-4.198497	-1.263628	1.077140	H	-3.676283	0.007403	-2.030037
C	-2.914524	-1.741219	1.333748	H	-1.396161	-0.804501	-1.554073
C	-1.886910	-1.582363	0.393940	H	0.047916	-2.323992	-0.226878
C	-3.466015	-0.464051	-1.074706	H	-0.539041	-2.981133	1.325831
C	-2.180098	-0.936075	-0.814511	H	1.212668	0.650051	3.695471
C	-0.501834	-2.097005	0.686493	H	1.938767	-0.945003	3.421104
O	0.273779	-1.141274	1.482850	H	2.970819	0.478068	3.637576
C	1.110085	-0.291708	0.890588	H	2.245347	4.352138	-0.573420
O	1.038959	-0.235234	-0.415091	H	1.940029	4.926519	1.063108
C	2.052027	0.388011	1.690851	H	3.468667	4.101641	0.686186
C	2.034639	0.120898	3.196515	H	-0.218871	3.648734	0.899148
C	2.492868	1.766823	1.302628	H	-0.217071	1.890341	1.106113
C	1.749306	2.785081	0.828638	H	0.047869	2.596922	-0.491827
C	2.393107	4.108199	0.487642	H	3.551775	1.963451	1.474032
C	0.263780	2.717558	0.578515	H	1.943527	0.007341	-0.873582

O	5.696639	0.546525	-0.707820	H	5.745789	-2.960402	0.106968
O	5.011093	-1.805825	-1.472575	H	6.973475	-1.991907	0.762360
P	4.453908	-0.488817	-0.723191	H	6.243940	-3.421749	-1.549872
O	3.278957	0.050656	-1.506401	C	6.063856	1.224165	-1.925619
O	4.197064	-0.842952	0.764253	H	6.862590	1.917055	-1.657462
H	3.196639	-0.348899	1.204825	H	5.208193	1.771741	-2.329005
C	6.061520	-2.587580	-0.871118	H	6.429645	0.510223	-2.670993

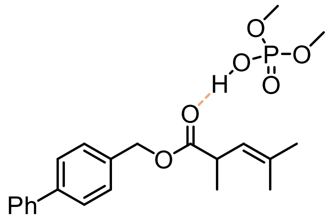

P-3 (PCM = toluene)

Gibbs free energy = -1648.315194 Hartree

C	-8.083190	0.695014	0.387201	C	1.981058	-2.527315	-0.252840
C	-8.160638	0.228653	1.702652	C	0.948014	-3.031532	0.443307
C	-6.841373	0.860804	-0.228853	C	1.189265	-3.946426	1.620031
C	-5.648823	0.563605	0.455916	C	-0.505618	-2.745860	0.160348
C	-6.984159	-0.070495	2.395744	H	-8.990272	0.939222	-0.158830
C	-5.742610	0.094772	1.779160	H	-9.126726	0.099766	2.182213
C	-4.325106	0.737410	-0.199905	H	-7.031900	-0.441718	3.415767
C	-4.141884	0.425732	-1.559755	H	-4.838221	-0.163769	2.322102
C	-2.900774	0.590378	-2.172487	H	-6.795937	1.246346	-1.243208
C	-1.798120	1.065467	-1.447922	H	-4.973032	0.034140	-2.138563
C	-3.217535	1.221930	0.518897	H	-2.785780	0.339486	-3.224275
C	-1.974672	1.380510	-0.093602	H	-3.333549	1.498761	1.562485
C	-0.459069	1.228730	-2.116170	H	-1.135360	1.757680	0.483258
O	0.271722	-0.044603	-2.193158	H	0.167613	1.955674	-1.598877
C	1.151479	-0.338447	-1.234458	H	-0.570378	1.515373	-3.163406
O	1.340720	0.406340	-0.272279	H	0.549011	-2.767652	-2.729008
C	1.940011	-1.618851	-1.478105	H	1.658368	-1.731984	-3.648798
C	1.568420	-2.373494	-2.767129	H	2.251497	-3.219448	-2.890897

H	0.750416	-3.527763	2.536008	O	3.460586	0.459640	1.306068
H	0.711468	-4.922921	1.462325	O	4.987321	-0.091257	-0.775384
H	2.255645	-4.111666	1.799280	H	2.964330	-1.237154	-1.597493
H	-1.066067	-3.684515	0.058725	C	5.935468	2.829983	-0.477178
H	-0.671806	-2.163623	-0.747553	H	5.827346	2.472085	-1.504440
H	-0.961247	-2.199221	0.996903	H	6.936144	2.587122	-0.104481
H	2.984871	-2.811026	0.056385	H	5.785758	3.910433	-0.441904
H	2.645744	0.390014	0.728120	C	5.942273	0.761758	2.919501
O	5.945948	0.228008	1.577575	H	6.842601	0.375667	3.400255
O	4.916008	2.256774	0.369826	H	5.056675	0.421251	3.462592
P	4.820462	0.644717	0.506256	H	5.972927	1.855512	2.904171

P-3 (gas phase)

Gibbs free energy = -1648.289326 Hartree

C	-8.034668	0.152294	-0.806773	C	1.988349	0.824486	2.257882
C	-8.068843	1.541394	-0.956323	C	0.948753	1.661763	2.406150
C	-6.813760	-0.510794	-0.672791	C	1.179761	3.134165	2.652971
C	-5.600254	0.199754	-0.684188	C	-0.503899	1.259044	2.333190
C	-6.870733	2.260750	-0.970426	H	-8.959116	-0.418227	-0.803681
C	-5.650351	1.597020	-0.835387	H	-9.018606	2.057565	-1.060921
C	-4.299868	-0.505469	-0.538543	H	-6.885501	3.341678	-1.077539
C	-4.155592	-1.583216	0.353357	H	-4.727798	2.169912	-0.822998
C	-2.936679	-2.246351	0.484832	H	-6.799379	-1.593044	-0.582911
C	-1.819159	-1.852434	-0.264051	H	-4.999678	-1.891648	0.962857
C	-3.176621	-0.118840	-1.290914	H	-2.850524	-3.074730	1.183886
C	-1.955826	-0.778577	-1.154474	H	-3.265873	0.691417	-2.008322
C	-0.504431	-2.570590	-0.109154	H	-1.100405	-0.461463	-1.743280
O	0.264950	-2.061148	1.030084	H	0.110749	-2.484107	-1.005571
C	1.143667	-1.075930	0.810716	H	-0.655051	-3.622301	0.140540
O	1.297231	-0.582421	-0.305254	H	0.614634	-1.265434	3.667911
C	1.960060	-0.684178	2.033465	H	1.723467	-2.550816	3.156249
C	1.628011	-1.472695	3.313819	H	2.327407	-1.177169	4.101123

H	0.715522	3.739680	1.862935	O	3.346280	0.892457	-1.144002
H	0.721932	3.449680	3.600104	O	4.884625	-0.509380	0.480659
H	2.244596	3.379655	2.688137	H	2.982402	-0.948939	1.727016
H	-1.033414	1.576465	3.241052	C	6.164070	-1.563584	-2.094730
H	-0.657284	0.184851	2.218841	H	6.070140	-2.242984	-1.243735
H	-0.999955	1.759070	1.490952	H	7.119677	-1.031699	-2.036575
H	2.990868	1.240603	2.327083	H	6.111672	-2.121311	-3.030921
H	2.560740	0.376778	-0.806114	C	5.737243	2.415433	-1.967632
O	5.766372	1.493225	-0.861080	H	6.541635	3.129547	-1.786401
O	5.063792	-0.633554	-2.120764	H	4.775563	2.933876	-2.003649
P	4.752599	0.244526	-0.792452	H	5.911223	1.891113	-2.912403

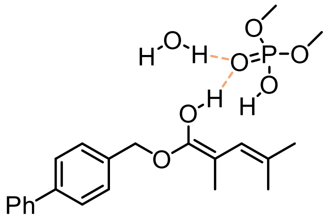

IS-4 (PCM = toluene)

Gibbs free energy = -1724.710941 Hartree

C	-8.551499	-1.629171	0.257946	C	1.049644	1.230874	-0.061818
C	-9.319534	-0.492993	0.528541	O	1.658437	1.046083	-1.250999
C	-7.188554	-1.507882	-0.019463	C	1.651556	1.530492	1.130465
C	-6.562209	-0.248145	-0.032061	C	0.832327	1.568725	2.408434
C	-8.710993	0.765449	0.519332	C	3.048839	2.004654	1.240058
C	-7.347946	0.886179	0.242534	C	3.684608	2.949768	0.511987
C	-5.109950	-0.117556	-0.326129	C	5.130804	3.284343	0.783418
C	-4.183331	-1.057158	0.160789	C	3.044846	3.736662	-0.601265
C	-2.822308	-0.932529	-0.115919	H	-9.014934	-2.611928	0.253780
C	-2.339793	0.132545	-0.888020	H	-10.380013	-0.587187	0.744227
C	-4.620468	0.948450	-1.102490	H	-9.295470	1.655164	0.737070
C	-3.258992	1.069560	-1.378345	H	-6.883252	1.867674	0.262199
C	-0.869695	0.276705	-1.170989	H	-6.609395	-2.397024	-0.251293
O	-0.295528	1.116912	-0.125322	H	-4.527952	-1.880786	0.779034

H	-2.125427	-1.667645	0.279456
H	-5.313895	1.674991	-1.515531
H	-2.907105	1.897268	-1.989179
H	-0.696570	0.749805	-2.140457
H	-0.367234	-0.695544	-1.161161
H	0.507828	2.591547	2.645210
H	-0.062337	0.944146	2.347060
H	1.436710	1.225630	3.257548
H	5.747753	3.103151	-0.107898
H	5.249350	4.347731	1.032358
H	5.542745	2.691406	1.606069
H	3.360487	4.786425	-0.553117
H	1.953422	3.701991	-0.562691
H	3.353056	3.358877	-1.586211
H	3.609636	1.570339	2.071078
H	2.556909	0.655602	-1.142934
O	5.040459	-1.421474	1.192659
O	3.737797	-3.059655	-0.306844
P	3.799266	-1.541346	0.184795
O	3.845211	-0.608428	-0.988233
O	2.543012	-1.371353	1.146905
H	2.174568	-0.444259	1.158002
C	3.651382	-4.163666	0.628962
H	2.705844	-4.121808	1.175363
H	4.493172	-4.144945	1.326334
H	3.691587	-5.069366	0.023324
C	6.391711	-1.300082	0.673216
H	7.045888	-1.327929	1.545175
H	6.507854	-0.349492	0.148722
H	6.630551	-2.131326	0.004131
O	5.792313	-0.520459	-3.071269
H	5.102926	-0.552599	-2.380849
H	5.348015	-0.784062	-3.887036

IS-4 (gas phase)

Gibbs free energy = -1724.678545 Hartree

C	-8.657373	-0.596394	-0.742294
C	-8.954797	0.753178	-0.950706
C	-7.348183	-0.993491	-0.465265
C	-6.307149	-0.051063	-0.388248
C	-7.930438	1.701194	-0.878389
C	-6.621888	1.303269	-0.599634
C	-4.913402	-0.473577	-0.090854
C	-4.645947	-1.476312	0.855654
C	-3.335795	-1.869728	1.131712
C	-2.251842	-1.278272	0.472636
C	-3.819550	0.118090	-0.749671
C	-2.512157	-0.276231	-0.474004
C	-0.839856	-1.711065	0.778782
O	-0.164771	-0.746775	1.634341
C	0.857277	-0.007033	1.148345
O	0.783834	0.205368	-0.183005
C	1.817520	0.465938	2.001018
C	1.823464	0.026760	3.454983
C	2.771151	1.538387	1.640995
C	2.537159	2.711544	1.012730
C	3.671336	3.659261	0.707516
C	1.176247	3.170791	0.558707
H	-9.443688	-1.343461	-0.804756
H	-9.973165	1.062557	-1.166969

H	-8.151017	2.754132	-1.029767
H	-5.839384	2.052237	-0.520757
H	-7.125309	-2.047935	-0.330755
H	-5.466945	-1.933894	1.399681
H	-3.154944	-2.640329	1.877321
H	-3.998211	0.877356	-1.505413
H	-1.682691	0.191496	-0.995763
H	-0.260255	-1.849334	-0.135196
H	-0.843297	-2.643716	1.349983
H	1.314280	0.761279	4.093145
H	1.328015	-0.936476	3.597152
H	2.853638	-0.052037	3.823625
H	3.791527	3.775447	-0.377716
H	3.471512	4.657278	1.119809
H	4.621192	3.301807	1.117294
H	1.048719	4.239358	0.770590
H	0.368285	2.620262	1.045333
H	1.058058	3.046305	-0.526367
H	3.792954	1.368189	1.988625
H	1.676478	0.309503	-0.588780
O	5.589193	-0.325456	-0.249617
O	4.522481	-2.271316	-1.544625
P	4.184940	-0.980666	-0.660440
O	3.242601	-0.073213	-1.390638
O	3.653650	-1.566574	0.717998
H	3.004380	-0.969270	1.185602
C	5.321031	-3.354964	-1.017794
H	4.804368	-3.828174	-0.179354
H	6.300693	-2.990235	-0.696320
H	5.437119	-4.064442	-1.837055
C	6.372926	0.423942	-1.215033
H	7.237703	0.792792	-0.663598
H	5.794908	1.257152	-1.619872
H	6.703428	-0.234284	-2.024371
O	3.896010	2.348562	-2.695352
H	3.567002	1.507004	-2.325357
H	3.548177	2.399307	-3.593315

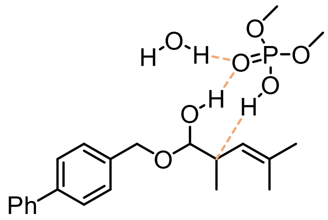

TS-4 (PCM = toluene)

Gibbs free energy = -1724.705496 Hartree

C	-8.587718	-1.652301	0.166900
C	-9.319349	-0.537172	0.585347
C	-7.229521	-1.531055	-0.132703
C	-6.572518	-0.292114	-0.020708
C	-8.679632	0.700239	0.701794
C	-7.321481	0.821203	0.402244
C	-5.125655	-0.162137	-0.340423
C	-4.211392	-1.171979	0.011838
C	-2.856358	-1.051045	-0.291533
C	-2.368260	0.083386	-0.954328
C	-4.629940	0.971999	-1.008404
C	-3.273606	1.091901	-1.308878

C	-0.906354	0.219447	-1.267794	H	4.553115	4.796430	0.915252
O	-0.252748	0.830729	-0.099328	H	5.279920	3.227006	1.326263
C	1.068460	0.942110	-0.079445	H	2.441953	4.854933	-0.448335
O	1.669007	0.699203	-1.224835	H	1.343262	3.467682	-0.442073
C	1.716454	1.204747	1.142009	H	2.693026	3.533825	-1.586553
C	0.849052	1.362853	2.390191	H	3.729674	1.646464	1.873517
C	2.989348	1.996182	1.153249	H	2.624393	0.369853	-1.110237
C	3.301657	3.097923	0.441658	O	5.356117	-1.197354	1.142993
C	4.654274	3.750089	0.596835	O	3.972007	-2.932751	-0.159124
C	2.389273	3.764478	-0.556069	P	3.986723	-1.393564	0.316616
H	-9.076120	-2.617419	0.064473	O	3.921183	-0.523789	-0.925212
H	-10.376144	-0.631435	0.818234	O	2.852241	-1.176477	1.351739
H	-9.235759	1.572256	1.034692	H	2.281781	-0.132922	1.251942
H	-6.831773	1.783491	0.519885	C	3.968639	-4.002716	0.812623
H	-6.679068	-2.401041	-0.478904	H	3.070947	-3.951133	1.434880
H	-4.560885	-2.050845	0.545260	H	4.862549	-3.959383	1.442261
H	-2.169416	-1.842741	-0.002783	H	3.968966	-4.930439	0.238472
H	-5.314617	1.755714	-1.318208	C	6.625125	-1.168541	0.447374
H	-2.917319	1.974316	-1.834655	H	7.391293	-1.135586	1.223455
H	-0.735498	0.867059	-2.130091	H	6.693780	-0.276670	-0.180004
H	-0.436093	-0.749351	-1.451347	H	6.760270	-2.065223	-0.164876
H	0.368084	2.348990	2.430699	O	5.572745	-0.583427	-3.230589
H	0.065714	0.601564	2.446657	H	5.005169	-0.567872	-2.434554
H	1.477047	1.268818	3.282535	H	5.122027	-1.171819	-3.849346
H	5.191471	3.764880	-0.361567				

TS-4 (gas phase)

Gibbs free energy = -1724.674103 Hartree

C	-8.450638	-0.145635	-0.409710	C	-7.434052	2.018263	-0.743951
C	-8.581207	1.227799	-0.632764	C	-6.168390	1.440522	-0.632484
C	-7.184599	-0.722842	-0.299195	C	-4.673078	-0.553729	-0.288444
C	-6.021534	0.060007	-0.408341	C	-4.417903	-1.564586	0.655338

Back to contents

C	-3.153797	-2.141462	0.763088
C	-2.100665	-1.724873	-0.062642
C	-3.613768	-0.144135	-1.117364
C	-2.347856	-0.717403	-1.004901
C	-0.737341	-2.350346	0.070246
O	0.045457	-1.716992	1.137658
C	0.922737	-0.758888	0.859224
O	0.889654	-0.299137	-0.371088
C	1.864895	-0.397797	1.842192
C	1.806551	-1.126117	3.185011
C	2.381545	1.007347	1.906495
C	1.723918	2.166100	1.705154
C	2.460432	3.482505	1.770996
C	0.258442	2.274033	1.368287
H	-9.335193	-0.771252	-0.330500
H	-9.566125	1.677179	-0.718943
H	-7.523552	3.088322	-0.908329
H	-5.285169	2.069371	-0.695151
H	-7.096052	-1.795132	-0.151046
H	-5.210693	-1.886701	1.323726
H	-2.980711	-2.918006	1.504161
H	-3.788739	0.614936	-1.873831
H	-1.544195	-0.382892	-1.653804
H	-0.172688	-2.298081	-0.860482
H	-0.811721	-3.388227	0.400159
H	0.995957	-0.745118	3.818846
H	1.664695	-2.203313	3.061509
H	2.746544	-0.965899	3.722560
H	2.464093	3.963786	0.784267
H	1.972763	4.173126	2.471445
H	3.500612	3.350466	2.082943
H	-0.196825	3.101851	1.924965
H	-0.300640	1.364280	1.600672
H	0.116621	2.491754	0.301175
H	3.433955	1.077621	2.182802
H	1.782869	0.092354	-0.681745
O	5.576498	0.427637	-0.205800
O	4.885380	-1.407892	-1.864103
P	4.321725	-0.460823	-0.686386
O	3.193951	0.376503	-1.263715
O	3.953348	-1.345732	0.529233
H	2.974413	-0.985031	1.133982
C	5.874315	-2.414700	-1.568061
H	5.486353	-3.122385	-0.830745
H	6.793613	-1.955231	-1.191595
H	6.075377	-2.925879	-2.510268
C	6.125306	1.456283	-1.061632
H	6.984039	1.860830	-0.524579
H	5.387946	2.242660	-1.239649
H	6.455443	1.027906	-2.013605
O	3.088337	3.140471	-1.814582
H	3.084302	2.174162	-1.664838
H	2.805972	3.263957	-2.728396

Back to contents

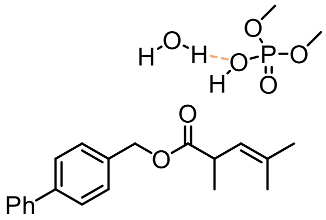

P-4 (PCM = toluene)

Gibbs free energy = -1724.744223 Hartree

C	-8.197490	0.773690	-0.064481	H	-7.030342	-0.354876	-1.473104
C	-8.179189	1.644670	1.028677	H	-5.221942	-1.808933	-1.035705
C	-7.001430	0.302323	-0.608828	H	-3.122044	-2.631002	-2.020798
C	-5.760009	0.690320	-0.072222	H	-3.417900	2.053209	-0.430082
C	-6.953281	2.039183	1.572244	H	-1.305029	1.211265	-1.369032
C	-5.757432	1.566999	1.028216	H	-0.138611	-0.482031	-2.718167
C	-4.485733	0.187148	-0.651637	H	-0.963881	-2.003128	-3.155948
C	-4.374057	-1.135641	-1.118658	H	0.311138	-4.020411	0.603483
C	-3.181970	-1.604100	-1.668128	H	1.307105	-4.282298	-0.842035
C	-2.057740	-0.771103	-1.763078	H	2.009319	-4.485797	0.772031
C	-3.356614	1.019228	-0.755877	H	1.004148	-0.022511	4.073995
C	-2.161741	0.547379	-1.299040	H	0.890256	-1.678226	4.662275
C	-0.773427	-1.289433	-2.352515	H	2.442819	-1.062524	4.057387
O	-0.005457	-2.082075	-1.379211	H	-1.043411	-2.027188	2.999875
C	0.941131	-1.478530	-0.662970	H	-0.760152	-1.933708	1.259784
O	1.163937	-0.270817	-0.767345	H	-0.870588	-0.455887	2.225802
C	1.757015	-2.416026	0.216496	H	2.970908	-1.864649	1.972976
C	1.310290	-3.888555	0.178632	H	2.433386	0.539213	-0.270493
C	1.936073	-1.891672	1.637739	O	5.445350	0.714885	1.108564
C	0.986903	-1.511312	2.510011	O	5.280508	1.582676	-1.302405
C	1.360819	-1.046280	3.896932	P	4.652035	0.503665	-0.275855
C	-0.492565	-1.486986	2.218914	O	3.209911	1.146560	-0.045110
H	-9.143927	0.466931	-0.501176	O	4.703134	-0.915592	-0.711673
H	-9.109650	2.011984	1.452015	H	2.751826	-2.353015	-0.247629
H	-6.926458	2.709396	2.427020	C	6.597696	1.345667	-1.849228
H	-4.813443	1.864385	1.475372	H	6.611179	0.415461	-2.423449

H	7.349317	1.303372	-1.054435
H	6.808110	2.190398	-2.506867
C	5.448810	1.989203	1.791780
H	6.136895	1.878104	2.631149
H	4.447874	2.225202	2.162282
H	5.799476	2.787590	1.131149
O	2.620176	4.003616	-0.347816
H	2.812998	3.055259	-0.246995
H	3.023268	4.248384	-1.190760

P-4 (gas phase)

Gibbs free energy = -1724.712598 Hartree

C	-8.157109	0.534622	-0.553029
C	-8.117864	1.901619	-0.265095
C	-6.972329	-0.194844	-0.664409
C	-5.722541	0.425677	-0.489661
C	-6.882945	2.531885	-0.089358
C	-5.698647	1.801613	-0.200021
C	-4.460587	-0.351144	-0.605660
C	-4.363196	-1.656949	-0.092460
C	-3.180385	-2.385333	-0.206039
C	-2.052979	-1.833003	-0.829636
C	-3.328109	0.194841	-1.235197
C	-2.143180	-0.532066	-1.343087
C	-0.776786	-2.624511	-0.939964
O	0.023158	-2.541013	0.286797
C	0.961341	-1.594116	0.371828
O	1.144529	-0.783873	-0.537559
C	1.806347	-1.669655	1.634227
C	1.340733	-2.723494	2.656710
C	2.057367	-0.307663	2.269331
C	1.163527	0.643133	2.587493
C	1.614164	1.942721	3.210984
C	-0.321504	0.548329	2.336675
H	-9.110944	0.036059	-0.700428
H	-9.039511	2.469491	-0.178489
H	-6.840582	3.592024	0.143810
H	-4.746505	2.297542	-0.035921
H	-7.015330	-1.251123	-0.913424
H	-5.214797	-2.097629	0.417079
H	-3.129818	-3.391667	0.202560
H	-3.382873	1.190266	-1.665584
H	-1.279780	-0.089897	-1.831074
H	-0.162046	-2.286913	-1.775020
H	-0.979556	-3.692788	-1.033308
H	0.358858	-2.475097	3.069374
H	1.286587	-3.721166	2.212362
H	2.055475	-2.752198	3.484064
H	1.406343	2.783130	2.536209
H	1.076968	2.135724	4.148962
H	2.686989	1.940316	3.422795
H	-0.875980	0.765550	3.258512
H	-0.647846	-0.430700	1.980177
H	-0.633789	1.297696	1.597787
H	3.103057	-0.118936	2.502113
H	2.392679	0.213115	-0.667659
O	5.519935	1.205433	0.069817
O	5.079658	0.204647	-2.250034
P	4.590571	0.150858	-0.707476
O	3.158503	0.852859	-0.815783

O	4.633163	-1.191701	-0.076515	H	6.468364	2.995729	0.245547
H	2.781511	-1.986767	1.234619	H	4.727822	3.133003	-0.138871
C	6.257347	-0.537489	-2.627042	H	5.894215	2.642784	-1.409991
H	6.153909	-1.587017	-2.340265	O	2.196817	3.505886	-0.115167
H	7.150519	-0.112887	-2.156829	H	2.467674	2.605949	-0.368499
H	6.335691	-0.448707	-3.711335	H	1.775501	3.879611	-0.898326
C	5.649424	2.583279	-0.345135				

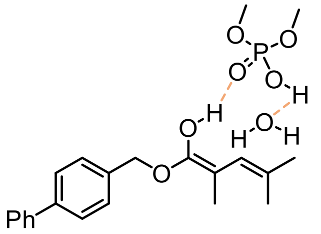

IS-5 (gas phase)

Gibbs free energy = -1724.685645 Hartree

C	8.433415	-1.147539	0.113229	C	-2.603169	2.693567	0.363751
C	8.648703	-0.792527	1.447723	C	-2.376027	2.641881	1.693571
C	7.165596	-1.012229	-0.454920	C	-3.489228	2.918454	2.674454
C	6.084778	-0.517314	0.296625	C	-1.043604	2.305426	2.310662
C	7.583929	-0.300404	2.207576	H	9.251238	-1.539699	-0.484871
C	6.316940	-0.163850	1.637958	H	9.634831	-0.898190	1.890418
C	4.735341	-0.369727	-0.309050	H	7.741037	-0.012895	3.243407
C	4.578684	0.089388	-1.627570	H	5.503739	0.243773	2.231267
C	3.310143	0.226543	-2.191891	H	7.005201	-1.315544	-1.485404
C	2.157697	-0.088525	-1.461620	H	5.454375	0.365254	-2.207691
C	3.574115	-0.686383	0.419564	H	3.215559	0.593362	-3.211266
C	2.307549	-0.548281	-0.144974	H	3.666205	-1.069475	1.431670
C	0.791474	0.071276	-2.083320	H	1.424213	-0.797109	0.434944
O	0.173918	1.330589	-1.706024	H	0.132427	-0.753810	-1.809329
C	-0.833903	1.352351	-0.795175	H	0.877279	0.120882	-3.172590
O	-0.873403	0.259703	-0.015982	H	-0.914762	4.378346	-1.388869
C	-1.660915	2.439917	-0.745677	H	-1.058036	3.203194	-2.699427
C	-1.525007	3.549943	-1.773798	H	-2.509816	3.971548	-2.012855

H	-3.674260	2.042224	3.311678	H	-4.089030	-0.589196	-1.817733
H	-3.225821	3.744104	3.349398	C	-4.751916	-4.144583	-0.167280
H	-4.425583	3.173241	2.168548	H	-4.585355	-4.133668	-1.247213
H	-0.851070	2.949954	3.177377	H	-5.813858	-3.994173	0.048345
H	-0.220802	2.420869	1.601528	H	-4.417111	-5.092386	0.255063
H	-1.023765	1.267753	2.671807	C	-6.011167	-0.945671	1.967977
H	-3.597890	3.019791	0.049674	H	-7.060104	-0.648388	1.989139
H	-1.771746	0.055129	0.356514	H	-5.378317	-0.129217	2.322953
O	-5.712042	-1.247413	0.588228	H	-5.864713	-1.830199	2.596072
O	-3.954731	-3.123640	0.469384	O	-3.744723	0.881267	-2.393140
P	-4.190952	-1.568901	0.156081	H	-3.541169	0.982381	-3.331383
O	-3.186362	-0.718671	0.858810	H	-3.016266	1.325481	-1.895414
O	-4.248022	-1.500597	-1.423012				

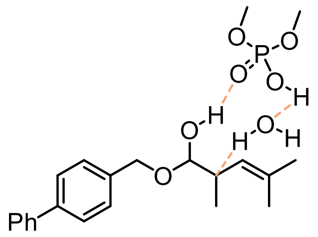

TS-5 (gas phase)

Gibbs free energy = -1724.676082 Hartree

C	8.287387	-0.817832	-0.080030	C	0.713309	0.679121	-2.315205
C	8.356854	-1.267014	1.241538	O	-0.001011	1.699787	-1.547601
C	7.053351	-0.505678	-0.652732	C	-0.898759	1.340105	-0.621814
C	5.861706	-0.634400	0.082817	O	-0.973056	0.064392	-0.381221
C	7.180757	-1.400506	1.984620	C	-1.721096	2.352666	-0.087463
C	5.947273	-1.087032	1.411547	C	-1.412068	3.801996	-0.475353
C	4.547517	-0.300461	-0.526209	C	-2.310551	2.185858	1.280214
C	4.404248	0.808953	-1.378372	C	-1.693533	1.781765	2.406552
C	3.171952	1.116459	-1.952460	C	-2.459549	1.667336	3.702517
C	2.040258	0.331990	-1.690888	C	-0.240603	1.387007	2.489278
C	3.409643	-1.086162	-0.271527	H	9.194089	-0.718836	-0.670167
C	2.175831	-0.774284	-0.841231	H	9.316795	-1.510440	1.687331

H	7.223323	-1.740426	3.015528
H	5.043055	-1.170870	2.007222
H	7.010298	-0.182015	-1.688586
H	5.260222	1.446976	-1.576825
H	3.086325	1.982228	-2.604725
H	3.496332	-1.964842	0.360577
H	1.309620	-1.392618	-0.627015
H	0.075346	-0.198511	-2.420840
H	0.854179	1.152749	-3.288988
H	-0.546289	4.193816	0.073974
H	-1.206088	3.910442	-1.544463
H	-2.269830	4.434834	-0.224959
H	-2.439800	0.633937	4.074987
H	-2.007707	2.291843	4.484829
H	-3.505665	1.964267	3.585140
H	0.216223	1.800045	3.397007
H	0.341616	1.731880	1.631414
H	-0.133532	0.295104	2.545890
H	-3.359357	2.473230	1.364951
H	-1.853366	-0.310737	0.080478
O	-5.515309	-1.347931	0.850881
O	-4.164778	-2.772081	-0.808147
P	-4.276107	-1.280930	-0.188746
O	-2.985612	-0.993332	0.549350
O	-4.720875	-0.326227	-1.303880
H	-4.340788	0.854080	-1.365815
C	-5.208354	-3.268245	-1.666417
H	-5.322595	-2.622146	-2.541001
H	-6.159332	-3.327136	-1.126779
H	-4.898298	-4.267603	-1.975680
C	-5.354348	-2.048548	2.097809
H	-6.283342	-1.901909	2.650908
H	-4.511993	-1.637459	2.660271
H	-5.193749	-3.117958	1.924215
O	-3.957674	1.951994	-1.436216
H	-3.910662	2.239546	-2.358098
H	-2.904258	2.081125	-0.908050

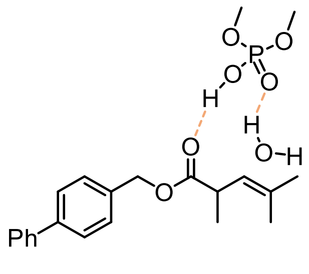

P-5 (gas phase)

Gibbs free energy = -1724.719797 Hartree

C	7.966306	-1.050073	-0.157861
C	7.991025	-1.598680	1.127262
C	6.758623	-0.638112	-0.723591
C	5.549158	-0.763970	-0.017146
C	6.796765	-1.730860	1.841014
C	5.589728	-1.317426	1.275054
C	4.263270	-0.322821	-0.618381
C	4.188440	0.851410	-1.388712

C	2.983033	1.260193	-1.956686	H	3.118002	-1.994164	0.127721
C	1.810416	0.515199	-1.769832	H	0.978828	-1.244870	-0.848111
C	3.084695	-1.068295	-0.438753	H	-0.175815	0.136992	-2.536756
C	1.877725	-0.655549	-1.002082	H	0.680722	1.491971	-3.326274
C	0.511812	0.969355	-2.383215	H	-0.198973	4.204162	0.209451
O	-0.158523	1.976566	-1.556610	H	-1.168587	4.317713	-1.272334
C	-1.047576	1.560300	-0.649453	H	-1.849873	4.833417	0.281579
O	-1.274943	0.364099	-0.471637	H	-1.224052	0.877356	4.239206
C	-1.781367	2.687843	0.056452	H	-0.993097	2.590967	4.575942
C	-1.205899	4.093963	-0.203223	H	-2.582696	2.001385	4.045333
C	-2.011042	2.407566	1.534932	H	0.963818	2.591704	2.880776
C	-1.094427	2.109966	2.470411	H	0.676115	2.192993	1.183868
C	-1.504257	1.885924	3.906737	H	0.698693	0.916518	2.406501
C	0.382964	1.948974	2.206579	H	-3.048423	2.490125	1.852733
H	8.887114	-0.950822	-0.725659	H	-2.441262	-0.377955	0.388998
H	8.930452	-1.919692	1.567571	O	-5.333867	-2.117609	1.006883
H	6.804935	-2.147867	2.844097	O	-3.603531	-2.728309	-0.804613
H	4.672050	-1.402236	1.849628	P	-4.312773	-1.479345	-0.056171
H	6.749460	-0.237030	-1.732851	O	-3.152161	-0.911143	0.854338
H	5.077506	1.459156	-1.527821	O	-5.021235	-0.558424	-0.987935
H	2.951070	2.173680	-2.545560	H	-4.733737	1.158251	-1.443227
C	-4.250157	-3.322416	-1.948715	H	-4.191689	-2.448433	2.724051
H	-4.494116	-2.556719	-2.688860	H	-4.359716	-3.861296	1.637267
H	-5.162345	-3.847248	-1.646567	O	-4.506820	2.088931	-1.656453
H	-3.536093	-4.035874	-2.362254	H	-5.343582	2.518028	-1.868311
C	-4.868619	-2.988334	2.057259	H	-2.769062	2.646756	-0.429378
H	-5.759121	-3.304604	2.601733				

7. X-ray

All single X-ray diffraction data were accumulated using Rigaku XtaLAB Synergy R, DW system, HyPix-Arc 150 diffractometer with Cu Kα radiation (λ = 1.54184 Å). The data collection was executed using the CrysAlisPro (Rigaku, V1.171.42.96a, 2023) program. Cell refinement and data reduction were made with CrysAlisPro (Rigaku, V1.171.42.96a, 2023) program. The structure was determined using the Olex2/ ShelXL program refined using full-matrix least squares. All non-hydrogen atoms were refined anisotropically, whereas hydrogen atoms placed at calculated positions and included in final stage of refinement with fixed parameters.

X-Ray crystallographic data for 2j (CCDC number 2338352).

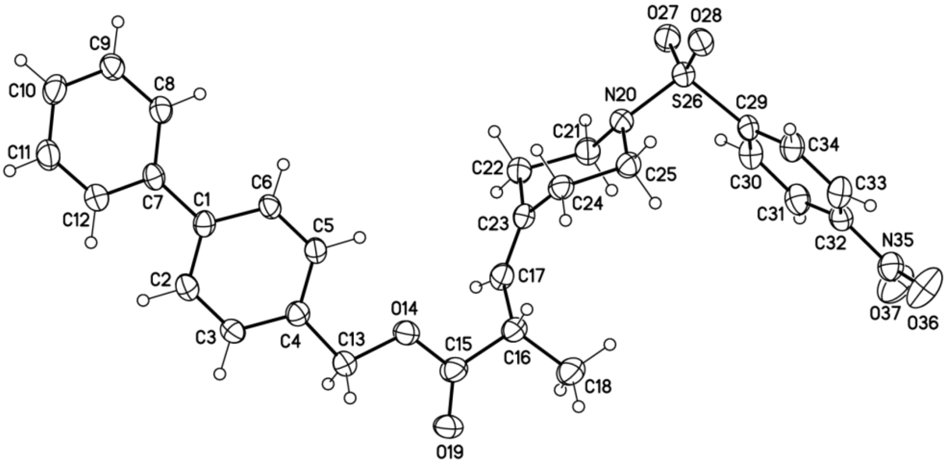

Figure S7.1 ORTEP drawing of (50% ellipsoids; hydrogen atoms not shown for clarity).

Table S7.1 Crystal data and structure refinement for 240203lt_auto.

Identification code	240203lt_auto
Empirical formula	$C_{28}H_{28}N_2O_6S$
Formula weight	520.58
Temperature/K	100.00(10)
Crystal system	monoclinic
Space group	$P2_1$
a/Å	8.00935(6)
b/Å	21.71113(19)
c/Å	15.03384(11)
α/°	90

β/°	103.2966(7)
γ/°	90
Volume/Å^3	2544.18(4)
Z	4
ρ_{calc}g/cm^3	1.359
μ/mm^{-1}	1.521
F(000)	1096.0
Crystal size/mm^3	0.11 × 0.09 × 0.06
Radiation	Cu Kα (λ = 1.54184)
2Θ range for data collection/°	6.04 to 134.14
Index ranges	-9 ≤ h ≤ 9, -22 ≤ k ≤ 25, -17 ≤ l ≤ 17
Reflections collected	60304
Independent reflections	8703 [R_{int} = 0.0267, R_{sigma} = 0.0221]
Data/restraints/parameters	8703/1/670
Goodness-of-fit on F^2	1.053
Final R indexes [I>=2σ (I)]	R_1 = 0.0309, wR_2 = 0.0777
Final R indexes [all data]	R_1 = 0.0333, wR_2 = 0.0789
Largest diff. peak/hole / e Å^{-3}	0.26/-0.40
Flack parameter	0.001(4)

Table S7.2 Fractional Atomic Coordinates (×10^4) and Equivalent Isotropic Displacement Parameters (Å^2×10^3) for 240203lt_auto. U_{eq} is defined as 1/3 of the trace of the orthogonalised U_{IJ} tensor.

**Atom**	***x***	***y***	***z***	**U(eq)**
S26	331.0(9)	5566.8(3)	3042.3(4)	29.79(17)
O14	8490(3)	3313.8(10)	5084.7(13)	36.1(5)
O19	8970(3)	2901.6(12)	3804.8(14)	47.8(6)
O27	-1299(3)	5334.7(10)	3128.7(14)	37.2(5)
O28	919(3)	6157.0(10)	3402.4(13)	36.4(5)

O36	1660(3)	5798.4(13)	-1150.7(15)	57.2(7)
O37	-256(4)	5091.2(13)	-1383.3(16)	56.1(7)
N20	1771(3)	5061.5(11)	3510.4(15)	26.6(5)
N35	654(3)	5462.7(13)	-889.3(16)	34.3(6)
C1	9718(3)	3117.5(13)	8494.9(18)	24.6(6)
C2	10457(4)	2605.4(14)	8166.8(19)	28.8(6)
C3	10416(4)	2538.0(13)	7253.6(19)	28.3(6)
C4	9617(3)	2973.8(14)	6618.4(19)	25.9(6)
C5	8867(3)	3484.5(13)	6931.0(18)	25.5(6)
C6	8941(3)	3560.0(13)	7854.9(18)	24.4(6)
C7	9733(3)	3171.3(13)	9481.9(18)	25.7(6)
C8	8440(4)	3486.0(13)	9783.5(18)	27.3(6)
C9	8419(4)	3505.9(14)	10698.9(19)	30.6(6)
C10	9706(4)	3212.0(15)	11347.3(19)	34.8(7)
C11	11010(4)	2915.8(15)	11060(2)	35.6(7)
C12	11025(4)	2896.3(14)	10148.9(19)	31.0(7)
C13	9613(4)	2869.5(15)	5634.1(19)	32.2(7)
C15	8262(4)	3273.1(15)	4174.7(18)	32.9(7)
C16	7037(4)	3765.6(14)	3707.8(18)	30.0(6)
C17	5307(4)	3687.8(14)	3915.7(18)	29.7(6)
C18	6846(4)	3760.0(17)	2674(2)	39.7(8)
C21	1321(4)	4403.1(15)	3368.8(18)	31.0(6)
C22	2473(4)	4022.2(14)	4114.9(19)	31.3(6)
C23	4315(4)	4139.6(14)	4092.3(17)	27.2(6)
C24	4781(4)	4812.9(14)	4195.1(18)	29.3(6)
C25	3566(3)	5199.2(15)	3474.1(18)	29.8(6)
C29	338(3)	5570.3(14)	1861.5(17)	27.4(6)

C30	-550(4)	5111.9(14)	1302.5(19)	32.4(7)
C31	-441(4)	5074.7(14)	398.9(19)	32.9(7)
C32	542(3)	5505.2(14)	75.2(18)	28.7(6)
C33	1421(4)	5966.1(15)	615.7(19)	33.2(7)
C34	1329(4)	5998.4(14)	1523.8(18)	30.8(7)
S63	14553.6(9)	4457.6(3)	6805.3(4)	28.50(17)
O51	6710(2)	6937.2(10)	4657.5(12)	32.0(5)
O56	5579(3)	6974.6(12)	5893.4(15)	47.9(6)
O64	14204(3)	3844.5(9)	6465.8(13)	32.5(5)
O65	16071(3)	4771.0(10)	6703.0(14)	35.8(5)
O73	13112(3)	4228.4(12)	10967.3(14)	49.3(6)
O74	14994(3)	4956.6(11)	11206.1(14)	49.0(6)
N57	12914(3)	4887.3(11)	6328.6(15)	25.5(5)
N72	14111(3)	4572.0(12)	10714.2(16)	33.6(6)
C38	5264(3)	6881.5(13)	1249.1(18)	25.5(6)
C39	5932(3)	6430.9(13)	1894.8(18)	26.5(6)
C40	5891(3)	6495.3(13)	2803.5(18)	26.6(6)
C41	5170(4)	7020.2(14)	3105.4(19)	28.3(6)
C42	4519(4)	7475.8(14)	2470.6(19)	32.0(7)
C43	4560(4)	7406.9(14)	1560(2)	30.7(7)
C44	5286(4)	6822.5(13)	267.0(19)	28.3(6)
C45	3982(4)	7083.0(14)	-413(2)	31.9(7)
C46	4002(4)	7046.1(15)	-1330(2)	34.8(7)
C47	5343(4)	6745.1(15)	-1595(2)	35.5(7)
C48	6642(4)	6485.5(14)	-933.1(19)	32.8(7)
C49	6619(4)	6518.5(13)	-16.0(19)	29.3(6)
C50	5048(4)	7096.8(16)	4079(2)	34.1(7)

C52	6788(4)	6872.7(14)	5554.9(19)	30.9(7)
C53	8532(4)	6644.3(13)	6051.0(17)	27.4(6)
C54	8587(4)	5960.6(13)	5881.1(17)	25.9(6)
C55	8875(4)	6788.0(17)	7072(2)	42.5(8)
C58	11207(3)	4632.3(13)	6358.8(18)	27.2(6)
C59	9832(3)	4951.3(13)	5633.6(18)	26.6(6)
C60	9931(3)	5639.9(13)	5762.4(16)	24.0(6)
C61	11693(3)	5896.7(13)	5794.8(18)	25.8(6)
C62	13043(3)	5555.5(14)	6517.5(17)	27.5(6)
C66	14554(3)	4450.6(15)	7983.1(18)	27.3(6)
C67	13632(4)	4002.5(14)	8329.3(18)	29.2(6)
C68	13494(4)	4038.1(14)	9228.8(18)	29.8(7)
C69	14286(4)	4521.7(15)	9759.7(18)	30.0(6)
C70	15210(4)	4968.3(15)	9432.0(19)	34.6(7)
C71	15348(4)	4931.9(15)	8532.3(19)	33.9(7)

Table S7.3 Anisotropic Displacement Parameters ($Å^2×10^3$) for 240203lt_auto. The Anisotropic displacement factor exponent takes the form: -$2\pi^2[h^2a^{*2}U_{11}+2hka^*b^*U_{12}+…]$.

**Atom**	**U_{11}**	**U_{22}**	**U_{33}**	**U_{23}**	**U_{13}**	**U_{12}**
S26	31.2(4)	32.8(4)	25.6(3)	5.1(3)	6.9(3)	8.0(3)
O14	40.1(12)	42.5(14)	24.5(10)	-0.6(9)	4.7(8)	16.4(10)
O19	58.0(15)	55.6(16)	31.3(11)	-0.3(10)	13.4(10)	29.8(12)
O27	28.4(10)	48.1(14)	37.2(11)	12.2(9)	11.9(8)	9.2(9)
O28	46.2(13)	32.5(13)	29.4(10)	1.7(9)	6.3(9)	9.7(10)
O36	60.5(16)	80(2)	33.1(12)	-1.7(12)	15.5(11)	-25.6(15)
O37	73.9(17)	59.7(18)	34.8(13)	-13.1(12)	12.7(12)	-20.1(14)
N20	25.2(12)	28.9(14)	26.4(12)	3.3(9)	7.0(9)	3.1(10)
N35	33.6(14)	40.9(17)	26.2(12)	2.2(11)	2.2(10)	3.4(12)

Back to contents

C1	23.2(14)	24.5(16)	25.4(13)	3.9(11)	4.4(10)	-3.4(11)
C2	30.3(15)	24.1(17)	31.3(15)	4.9(11)	5.6(12)	2.3(12)
C3	30.2(15)	23.8(16)	31.6(15)	0.8(11)	8.5(12)	3.9(12)
C4	25.2(14)	25.3(16)	27.3(14)	1.5(11)	6.5(11)	-1.2(11)
C5	23.9(14)	25.9(16)	26.1(14)	5.4(11)	4.6(11)	0.8(11)
C6	25.1(14)	19.7(16)	28.6(14)	3.9(11)	6.9(11)	1.1(11)
C7	28.4(14)	18.6(16)	29.6(14)	4.5(11)	5.8(11)	-2.2(11)
C8	31.7(15)	22.6(16)	26.0(14)	2.1(11)	3.6(11)	-0.5(12)
C9	37.3(16)	24.0(17)	30.1(15)	1.1(12)	6.9(12)	2.6(13)
C10	47.4(18)	33.6(19)	22.3(14)	2.2(12)	5.8(12)	-0.2(14)
C11	39.5(17)	34.2(19)	30.7(15)	7.4(13)	3.0(13)	4.8(14)
C12	33.7(16)	28.1(18)	31.3(15)	4.3(12)	7.6(12)	3.2(13)
C13	35.5(16)	33.3(18)	27.6(15)	4.3(12)	7.2(12)	10.6(13)
C15	31.2(15)	42.5(19)	24.5(14)	-3.1(13)	5.3(12)	6.0(13)
C16	31.4(15)	34.9(17)	24.3(13)	-0.5(12)	7.4(11)	3.6(12)
C17	32.6(15)	32.2(17)	24.4(14)	2.7(11)	6.5(11)	0.2(12)
C18	39.7(17)	52(2)	28.1(15)	1.1(14)	8.9(13)	8.2(15)
C21	30.8(15)	31.5(17)	30.3(14)	3.1(12)	6.4(11)	0.6(13)
C22	31.4(15)	32.6(18)	31.3(15)	6.1(12)	9.7(12)	3.9(13)
C23	30.3(15)	31.5(17)	19.1(13)	3.1(11)	4.4(11)	4.6(12)
C24	25.7(14)	37.8(19)	24.3(14)	0.2(12)	5.4(11)	3.3(12)
C25	27.4(14)	34.7(18)	28.1(14)	3.7(12)	8.1(11)	1.9(12)
C29	26.5(14)	31.3(17)	23.1(13)	6.7(12)	3.2(11)	6.3(13)
C30	32.4(15)	31.4(18)	31.0(15)	6.8(12)	2.6(12)	-2.5(13)
C31	35.5(16)	28.9(18)	30.5(15)	1.1(12)	-0.4(12)	-2.3(13)
C32	27.1(14)	31.0(18)	26.1(14)	2.4(12)	1.9(11)	4.2(12)
C33	31.2(16)	38(2)	28.8(15)	6.3(13)	2.5(12)	-5.0(13)

C34	29.1(16)	33.5(19)	26.7(14)	2.7(12)	0.2(12)	-2.3(13)
S63	26.9(3)	33.7(4)	24.8(3)	6.0(3)	5.7(3)	3.2(3)
O51	30.4(10)	39.4(13)	26.0(10)	-0.3(8)	6.0(8)	4.4(9)
O56	44.2(13)	66.9(18)	37.0(12)	1.8(11)	18.2(10)	20.0(12)
O64	37.4(11)	29.4(12)	30.5(10)	0.8(8)	7.3(8)	7.4(9)
O65	27.1(10)	46.5(14)	34.5(11)	13.0(9)	8.6(8)	1.7(9)
O73	47.2(14)	70.2(19)	31.9(12)	-1.6(11)	11.7(10)	-15.3(12)
O74	72.3(17)	41.7(15)	28.6(11)	-5.9(10)	2.6(11)	-6.9(12)
N57	24.6(11)	25.0(13)	26.1(11)	4.7(9)	4.2(9)	0.0(9)
N72	35.4(14)	35.2(17)	27.3(12)	2.1(11)	1.2(10)	3.4(12)
C38	20.6(13)	24.9(17)	29.5(14)	-0.2(11)	2.4(11)	-3.5(11)
C39	24.7(14)	21.6(16)	32.1(14)	-2.6(11)	4.2(11)	-0.9(11)
C40	24.2(14)	25.3(16)	29.8(14)	2.5(11)	5.4(11)	0.0(11)
C41	24.4(14)	28.3(17)	31.0(14)	-1.8(12)	3.9(11)	0.3(12)
C42	32.3(16)	27.7(17)	34.2(16)	-4.1(12)	3.9(12)	6.6(12)
C43	32.2(16)	25.7(17)	31.3(15)	-0.1(12)	1.4(12)	3.9(12)
C44	28.5(15)	22.5(16)	32.3(15)	-1.3(11)	3.9(12)	-5.9(12)
C45	31.1(15)	27.2(17)	36.2(16)	-0.5(13)	5.3(13)	-1.3(13)
C46	36.1(16)	32.5(18)	32.3(16)	3.4(13)	1.2(13)	-1.0(13)
C47	44.0(18)	32.9(19)	29.7(16)	-1.1(12)	9.0(13)	-5.8(14)
C48	36.5(17)	27.8(18)	35.5(15)	-2.6(12)	11.2(13)	-1.7(13)
C49	31.7(15)	22.6(16)	31.8(14)	0.6(12)	3.6(12)	-1.0(12)
C50	30.3(16)	36.8(19)	34.7(16)	-1.6(13)	6.3(13)	8.9(13)
C52	40.6(17)	26.4(17)	28.0(14)	-3.9(12)	12.9(12)	4.3(13)
C53	32.3(15)	26.7(16)	25.1(14)	-3.9(11)	10.2(11)	0.1(12)
C54	29.5(14)	28.3(16)	19.4(13)	0.8(10)	4.4(11)	-3.6(12)
C55	51.8(19)	47(2)	28.4(16)	-10.4(13)	9.0(14)	2.2(16)

C58	25.7(14)	29.2(17)	26.6(13)	2.0(11)	5.9(11)	-3.2(11)
C59	25.1(13)	29.3(17)	24.5(14)	0.6(11)	3.6(11)	-2.2(11)
C60	27.0(14)	25.8(16)	18.3(12)	2.7(11)	3.2(10)	-1.8(12)
C61	26.2(14)	25.1(15)	26.3(14)	4.6(11)	6.4(11)	-0.2(12)
C62	27.3(14)	28.1(16)	26.1(13)	1.4(12)	3.9(11)	-3.8(12)
C66	24.6(14)	30.7(16)	24.8(13)	5.7(12)	2.2(10)	3.5(12)
C67	31.8(15)	29.0(18)	25.4(14)	-0.9(11)	3.4(11)	-2.4(12)
C68	27.9(15)	33.2(18)	26.9(14)	5.2(12)	3.9(11)	-3.5(12)
C69	28.1(14)	36.6(19)	22.6(13)	4.2(12)	0.5(11)	6.8(13)
C70	38.9(17)	30.5(18)	29.1(15)	2.5(12)	-2.9(12)	-4.6(13)
C71	32.3(16)	34.7(19)	31.0(15)	7.8(12)	-0.6(12)	-6.5(13)

Table S7.4 Bond Lengths for 240203lt_auto.

**Atom**	**Atom**	**Length/Å**	**Atom**	**Atom**	**Length/Å**
S26	O27	1.433(2)	S63	O64	1.430(2)
S26	O28	1.428(2)	S63	O65	1.432(2)
S26	N20	1.630(2)	S63	N57	1.636(2)
S26	C29	1.777(3)	S63	C66	1.771(3)
O14	C13	1.442(3)	O51	C50	1.455(3)
O14	C15	1.341(3)	O51	C52	1.343(3)
O19	C15	1.195(4)	O56	C52	1.214(3)
O36	N35	1.217(3)	O73	N72	1.217(3)
O37	N35	1.218(3)	O74	N72	1.226(3)
N20	C21	1.478(4)	N57	C58	1.486(3)
N20	C25	1.481(3)	N57	C62	1.477(4)
N35	C32	1.476(3)	N72	C69	1.478(3)
C1	C2	1.401(4)	C38	C39	1.395(4)
C1	C6	1.400(4)	C38	C43	1.399(4)

C1	C7	1.486(4)	C38	C44	1.486(4)
C2	C3	1.374(4)	C39	C40	1.381(4)
C3	C4	1.391(4)	C40	C41	1.400(4)
C4	C5	1.393(4)	C41	C42	1.390(4)
C4	C13	1.496(4)	C41	C50	1.498(4)
C5	C6	1.386(4)	C42	C43	1.385(4)
C7	C8	1.400(4)	C44	C45	1.401(4)
C7	C12	1.399(4)	C44	C49	1.402(4)
C8	C9	1.381(4)	C45	C46	1.386(4)
C9	C10	1.399(4)	C46	C47	1.391(4)
C10	C11	1.378(4)	C47	C48	1.384(4)
C11	C12	1.373(4)	C48	C49	1.385(4)
C15	C16	1.510(4)	C52	C53	1.508(4)
C16	C17	1.498(4)	C53	C54	1.509(4)
C16	C18	1.527(4)	C53	C55	1.528(4)
C17	C23	1.327(4)	C54	C60	1.328(4)
C21	C22	1.523(4)	C58	C59	1.526(4)
C22	C23	1.505(4)	C59	C60	1.507(4)
C23	C24	1.508(4)	C60	C61	1.508(4)
C24	C25	1.530(4)	C61	C62	1.536(4)
C29	C30	1.389(4)	C66	C67	1.393(4)
C29	C34	1.392(4)	C66	C71	1.392(4)
C30	C31	1.383(4)	C67	C68	1.384(4)
C31	C32	1.381(4)	C68	C69	1.382(4)
C32	C33	1.376(4)	C69	C70	1.378(4)
C33	C34	1.386(4)	C70	C71	1.385(4)

Table S7.5 Bond Angles for 240203lt_auto.

Atom	Atom	Atom	Angle/°	Atom	Atom	Atom	Angle/°
O27	S26	N20	107.06(12)	O64	S63	O65	120.34(13)
O27	S26	C29	107.73(13)	O64	S63	N57	107.65(12)
O28	S26	O27	120.61(13)	O64	S63	C66	107.70(14)
O28	S26	N20	107.53(12)	O65	S63	N57	107.33(12)
O28	S26	C29	107.35(14)	O65	S63	C66	107.85(13)
N20	S26	C29	105.65(12)	N57	S63	C66	104.98(12)
C15	O14	C13	117.5(2)	C52	O51	C50	116.8(2)
C21	N20	S26	117.63(18)	C58	N57	S63	115.22(18)
C21	N20	C25	113.4(2)	C62	N57	S63	117.51(18)
C25	N20	S26	116.40(19)	C62	N57	C58	112.6(2)
O36	N35	O37	123.2(3)	O73	N72	O74	123.9(2)
O36	N35	C32	118.3(2)	O73	N72	C69	118.3(2)
O37	N35	C32	118.5(3)	O74	N72	C69	117.7(3)
C2	C1	C7	120.5(2)	C39	C38	C43	117.3(3)
C6	C1	C2	117.3(2)	C39	C38	C44	122.7(3)
C6	C1	C7	122.2(2)	C43	C38	C44	120.0(3)
C3	C2	C1	121.4(3)	C40	C39	C38	121.6(3)
C2	C3	C4	121.0(3)	C39	C40	C41	120.6(3)
C3	C4	C5	118.4(3)	C40	C41	C50	122.1(3)
C3	C4	C13	118.1(3)	C42	C41	C40	118.3(3)
C5	C4	C13	123.4(2)	C42	C41	C50	119.6(3)
C6	C5	C4	120.6(2)	C43	C42	C41	120.8(3)
C5	C6	C1	121.1(3)	C42	C43	C38	121.4(3)
C8	C7	C1	121.7(2)	C45	C44	C38	120.8(3)
C12	C7	C1	121.1(3)	C45	C44	C49	117.4(3)
C12	C7	C8	117.2(3)	C49	C44	C38	121.8(2)

C9	C8	C7	121.2(3)	C46	C45	C44	121.7(3)
C8	C9	C10	120.3(3)	C45	C46	C47	119.9(3)
C11	C10	C9	119.0(3)	C48	C47	C46	119.2(3)
C12	C11	C10	120.6(3)	C47	C48	C49	121.0(3)
C11	C12	C7	121.8(3)	C48	C49	C44	120.8(3)
O14	C13	C4	108.9(2)	O51	C50	C41	107.6(2)
O14	C15	C16	110.4(2)	O51	C52	C53	110.8(2)
O19	C15	O14	123.5(3)	O56	C52	O51	123.0(3)
O19	C15	C16	126.1(3)	O56	C52	C53	126.2(3)
C15	C16	C18	111.7(2)	C52	C53	C54	107.5(2)
C17	C16	C15	110.9(2)	C52	C53	C55	111.2(2)
C17	C16	C18	109.3(2)	C54	C53	C55	111.4(2)
C23	C17	C16	125.7(3)	C60	C54	C53	126.5(3)
N20	C21	C22	109.4(2)	N57	C58	C59	109.1(2)
C23	C22	C21	108.8(2)	C60	C59	C58	110.5(2)
C17	C23	C22	121.0(3)	C54	C60	C59	121.1(3)
C17	C23	C24	126.3(3)	C54	C60	C61	125.7(3)
C22	C23	C24	112.5(2)	C59	C60	C61	113.1(2)
C23	C24	C25	110.9(2)	C60	C61	C62	110.3(2)
N20	C25	C24	109.1(2)	N57	C62	C61	109.3(2)
C30	C29	S26	118.9(2)	C67	C66	S63	120.1(2)
C30	C29	C34	120.9(3)	C71	C66	S63	118.8(2)
C34	C29	S26	120.0(2)	C71	C66	C67	120.8(3)
C31	C30	C29	119.8(3)	C68	C67	C66	119.6(3)
C32	C31	C30	118.4(3)	C69	C68	C67	118.5(3)
C31	C32	N35	118.2(3)	C68	C69	N72	118.9(3)
C33	C32	N35	119.2(3)	C70	C69	N72	118.3(3)

C33	C32	C31	122.7(3)	C70	C69	C68	122.8(3)
C32	C33	C34	118.9(3)	C69	C70	C71	118.7(3)
C33	C34	C29	119.3(3)	C70	C71	C66	119.6(3)

Table S7.6 Torsion Angles for 240203lt_auto.

A	B	C	D	Angle/°	A	B	C	D	Angle/°
S26	N20	C21	C22	-158.72(18)	S63	N57	C58	C59	-160.75(18)
S26	N20	C25	C24	161.20(19)	S63	N57	C62	C61	162.01(17)
S26	C29	C30	C31	-174.5(2)	S63	C66	C67	C68	-173.3(2)
S26	C29	C34	C33	175.2(2)	S63	C66	C71	C70	173.3(2)
O14	C15	C16	C17	-61.8(3)	O51	C52	C53	C54	-78.5(3)
O14	C15	C16	C18	176.0(3)	O51	C52	C53	C55	159.3(2)
O19	C15	C16	C17	119.5(3)	O56	C52	C53	C54	99.8(4)
O19	C15	C16	C18	-2.6(5)	O56	C52	C53	C55	-22.4(4)
O27	S26	N20	C21	38.7(2)	O64	S63	N57	C58	48.5(2)
O27	S26	N20	C25	178.20(19)	O64	S63	N57	C62	-175.03(19)
O27	S26	C29	C30	-33.6(3)	O64	S63	C66	C67	-25.1(3)
O27	S26	C29	C34	151.3(2)	O64	S63	C66	C71	161.0(2)
O28	S26	N20	C21	169.70(19)	O65	S63	N57	C58	179.41(18)
O28	S26	N20	C25	-50.8(2)	O65	S63	N57	C62	-44.1(2)
O28	S26	C29	C30	-164.9(2)	O65	S63	C66	C67	-156.4(2)
O28	S26	C29	C34	20.0(3)	O65	S63	C66	C71	29.7(3)
O36	N35	C32	C31	-172.3(3)	O73	N72	C69	C68	-8.5(4)
O36	N35	C32	C33	7.4(4)	O73	N72	C69	C70	170.3(3)
O37	N35	C32	C31	7.3(4)	O74	N72	C69	C68	171.0(3)
O37	N35	C32	C33	-173.1(3)	O74	N72	C69	C70	-10.2(4)
N20	S26	C29	C30	80.6(2)	N57	S63	C66	C67	89.4(3)
N20	S26	C29	C34	-94.5(3)	N57	S63	C66	C71	-84.5(2)

N20	C21	C22	C23	-57.8(3)	N57	C58	C59	C60	-55.5(3)
N35	C32	C33	C34	-179.1(3)	N72	C69	C70	C71	-178.6(3)
C1	C2	C3	C4	-0.9(4)	C38	C39	C40	C41	0.0(4)
C1	C7	C8	C9	176.6(3)	C38	C44	C45	C46	178.4(3)
C1	C7	C12	C11	-176.7(3)	C38	C44	C49	C48	-178.0(3)
C2	C1	C6	C5	1.7(4)	C39	C38	C43	C42	-0.4(4)
C2	C1	C7	C8	-150.5(3)	C39	C38	C44	C45	148.4(3)
C2	C1	C7	C12	28.1(4)	C39	C38	C44	C49	-33.0(4)
C2	C3	C4	C5	0.5(4)	C39	C40	C41	C42	-0.8(4)
C2	C3	C4	C13	-179.7(3)	C39	C40	C41	C50	177.9(3)
C3	C4	C5	C6	1.0(4)	C40	C41	C42	C43	1.0(4)
C3	C4	C13	O14	170.6(2)	C40	C41	C50	O51	47.1(4)
C4	C5	C6	C1	-2.1(4)	C41	C42	C43	C38	-0.4(5)
C5	C4	C13	O14	-9.6(4)	C42	C41	C50	O51	-134.2(3)
C6	C1	C2	C3	-0.2(4)	C43	C38	C39	C40	0.6(4)
C6	C1	C7	C8	27.8(4)	C43	C38	C44	C45	-31.9(4)
C6	C1	C7	C12	-153.6(3)	C43	C38	C44	C49	146.7(3)
C7	C1	C2	C3	178.2(3)	C44	C38	C39	C40	-179.8(3)
C7	C1	C6	C5	-176.7(2)	C44	C38	C43	C42	180.0(3)
C7	C8	C9	C10	0.3(4)	C44	C45	C46	C47	-0.1(5)
C8	C7	C12	C11	1.9(4)	C45	C44	C49	C48	0.7(4)
C8	C9	C10	C11	1.5(5)	C45	C46	C47	C48	0.1(5)
C9	C10	C11	C12	-1.6(5)	C46	C47	C48	C49	0.3(5)
C10	C11	C12	C7	-0.2(5)	C47	C48	C49	C44	-0.7(5)
C12	C7	C8	C9	-2.0(4)	C49	C44	C45	C46	-0.3(4)
C13	O14	C15	O19	-2.0(5)	C50	O51	C52	O56	-4.2(4)
C13	O14	C15	C16	179.3(2)	C50	O51	C52	C53	174.1(2)

C13	C4	C5	C6	-178.8(3)	C50	C41	C42	C43	-177.7(3)
C15	O14	C13	C4	-177.0(2)	C52	O51	C50	C41	-168.8(2)
C15	C16	C17	C23	139.5(3)	C52	C53	C54	C60	143.3(3)
C16	C17	C23	C22	168.2(2)	C53	C54	C60	C59	177.6(2)
C16	C17	C23	C24	-5.3(5)	C53	C54	C60	C61	1.4(4)
C17	C23	C24	C25	119.0(3)	C54	C60	C61	C62	123.1(3)
C18	C16	C17	C23	-96.9(3)	C55	C53	C54	C60	-94.6(3)
C21	N20	C25	C24	-57.7(3)	C58	N57	C62	C61	-60.5(3)
C21	C22	C23	C17	-117.7(3)	C58	C59	C60	C54	-122.8(3)
C21	C22	C23	C24	56.7(3)	C58	C59	C60	C61	53.8(3)
C22	C23	C24	C25	-55.0(3)	C59	C60	C61	C62	-53.3(3)
C23	C24	C25	N20	53.2(3)	C60	C61	C62	N57	55.0(3)
C25	N20	C21	C22	60.7(3)	C62	N57	C58	C59	60.7(3)
C29	S26	N20	C21	-75.9(2)	C66	S63	N57	C58	-66.0(2)
C29	S26	N20	C25	63.6(2)	C66	S63	N57	C62	70.4(2)
C29	C30	C31	C32	-0.9(4)	C66	C67	C68	C69	-0.1(4)
C30	C29	C34	C33	0.2(4)	C67	C66	C71	C70	-0.5(4)
C30	C31	C32	N35	180.0(3)	C67	C68	C69	N72	178.5(2)
C30	C31	C32	C33	0.3(5)	C67	C68	C69	C70	-0.2(4)
C31	C32	C33	C34	0.5(5)	C68	C69	C70	C71	0.1(5)
C32	C33	C34	C29	-0.8(4)	C69	C70	C71	C66	0.2(4)
C34	C29	C30	C31	0.6(4)	C71	C66	C67	C68	0.4(4)

Table S7.7 Hydrogen Atom Coordinates (Å×10^4) and Isotropic Displacement Parameters (Å^2×10^3) for 240203lt_auto.

**Atom**	***x***	***y***	***z***	**U(eq)**
H2	10996.59	2298.1	8584.97	35
H3	10942.26	2188.67	7052.7	34

H5	8299.64	3783.95	6506.83	31
H6	8456.08	3918.19	8057.27	29
H8	7561.87	3689.47	9350.8	33
H9	7527.16	3720.12	10889.27	37
H10	9680.79	3216.49	11975.98	42
H11	11906.07	2723.46	11495.6	43
H12	11938.82	2690.35	9966.59	37
H13A	10790.68	2915.29	5540.44	39
H13B	9210.33	2447.01	5451.79	39
H16	7505.01	4175.72	3946.38	36
H17	4883.89	3279.48	3921.58	36
H18A	6329.81	3369.5	2422.91	60
H18B	7978.14	3804.64	2536.17	60
H18C	6108.17	4102.3	2398.51	60
H21A	1469.76	4269.74	2762.16	37
H21B	104.54	4340.35	3386.91	37
H22A	2277.16	4138.32	4719.53	38
H22B	2204.16	3579.06	4013.33	38
H24A	5974.12	4870.01	4130.76	35
H24B	4719.92	4954.66	4812.67	35
H25A	3806	5642.64	3593	36
H25B	3742.5	5101.37	2859.15	36
H30	-1230.67	4824.77	1539.78	39
H31	-1029.27	4760.37	10.16	40
H33	2079.24	6257.03	370.41	40
H34	1936.19	6309.6	1911.71	37
H39	6427.5	6070.83	1704.6	32

H40	6356.38	6180.61	3227.27	32
H42	4040.77	7838.55	2663.55	38
H43	4101.7	7723.13	1137.19	37
H45	3061.24	7290.16	-239.77	38
H46	3101.46	7226.14	-1777.83	42
H47	5366.25	6718.15	-2222.89	43
H48	7563.05	6281.49	-1110.34	39
H49	7517.28	6332.81	426.51	35
H50A	4751.43	7527.82	4192.04	41
H50B	4147.56	6823.21	4212.05	41
H53	9426.74	6851.21	5789.36	33
H54	7557.79	5737.1	5856.54	31
H55A	8743.22	7231.28	7158.14	64
H55B	10045.63	6662.58	7369.15	64
H55C	8055.6	6562.3	7343.46	64
H58A	10979.39	4700.74	6971.3	33
H58B	11185.36	4183.33	6241.77	33
H59A	9994.99	4846.06	5018.39	32
H59B	8684.09	4803.7	5676.92	32
H61A	11715.78	6340.98	5945.24	31
H61B	11964.45	5851.19	5188.15	31
H62A	14205.17	5703.48	6501.19	33
H62B	12851.65	5638.25	7133.86	33
H67	13100.03	3674.28	7950.1	35
H68	12869.33	3736.61	9475.81	36
H70	15740.46	5294.72	9815.43	42
H71	15982.26	5233.61	8291.35	41

Back to contents

CheckCIF for 2j (CCDC number 2338352)

Datablock: 240203lt_auto

Bond precision:	C-C = 0.0041 A		Wavelength=1.54184
Cell:	a=8.00935(6)	b=21.71113(19)	c=15.03384(11)
	alpha=90	beta=103.2966(7)	gamma=90
Temperature:	100 K		

	Calculated	Reported
Volume	2544.18(4)	2544.18(4)
Space group	P 21	P 1 21 1
Hall group	P 2yb	P 2yb
Moiety formula	C28 H28 N2 O6 S	C28 H28 N2 O6 S
Sum formula	C28 H28 N2 O6 S	C28 H28 N2 O6 S
Mr	520.58	520.58
Dx,g cm-3	1.359	1.359
Z	4	4
Mu (mm-1)	1.521	1.521
F000	1096.0	1096.0
F000'	1100.64	
h,k,lmax	9,25,17	9,25,17
Nref	9091[4677]	8703
Tmin,Tmax	0.849,0.913	0.925,1.000
Tmin'	0.846	

Correction method= # Reported T Limits: Tmin=0.925 Tmax=1.000 AbsCorr = MULTI-SCAN

Data completeness= 1.86/0.96	Theta(max)= 67.070
R(reflections)= 0.0309(8233)	wR2(reflections)= 0.0789(8703)
S = 1.053	Npar= 670

Back to contents

The following ALERTS were generated. Each ALERT has the format
test-name_ALERT_alert-type_alert-level.
Click on the hyperlinks for more details of the test.

Alert level C

PLAT089_ALERT_3_C Poor Data / Parameter Ratio (Zmax < 18) .... 6.98 Note
PLAT340_ALERT_3_C Low Bond Precision on C-C Bonds .... 0.00409 Ang.

Alert level G

PLAT111_ALERT_2_G ADDSYM Detects New (Pseudo) Centre of Symmetry. 90 %Fit
PLAT113_ALERT_2_G ADDSYM Suggests Possible Pseudo/New Space Group P21/c Check
Check Model Parameter Symmetry for Reflection Data Support
PLAT142_ALERT_4_G s.u. on b - Axis Small or Missing .... 0.00019 Ang.
PLAT143_ALERT_4_G s.u. on c - Axis Small or Missing .... 0.00011 Ang.
PLAT909_ALERT_3_G Percentage of I>2sig(I) Data at Theta(Max) Still 88% Note
PLAT969_ALERT_5_G The 'Henn et al.' R-Factor-gap value .... 3.89 Note
Predicted wR2: Based on SigI**2 2.02 or SHELX Weight 7.79
PLAT978_ALERT_2_G Number C-C Bonds with Positive Residual Density. 12 Info

0 **ALERT level A** = Most likely a serious problem - resolve or explain
0 **ALERT level B** = A potentially serious problem, consider carefully
2 **ALERT level C** = Check. Ensure it is not caused by an omission or oversight
7 **ALERT level G** = General information/check it is not something unexpected

0 ALERT type 1 CIF construction/syntax error, inconsistent or missing data
3 ALERT type 2 Indicator that the structure model may be wrong or deficient
3 ALERT type 3 Indicator that the structure quality may be low
2 ALERT type 4 Improvement, methodology, query or suggestion
1 ALERT type 5 Informative message, check

It is advisable to attempt to resolve as many as possible of the alerts in all categories. Often the minor alerts point to easily fixed oversights, errors and omissions in your CIF or refinement strategy, so attention to these fine details can be worthwhile. In order to resolve some of the more serious problems it may be necessary to carry out additional measurements or structure refinements. However, the purpose of your study may justify the reported deviations and the more serious of these should normally be commented upon in the discussion or experimental section of a paper or in the "special_details" fields of the CIF. checkCIF was carefully designed to identify outliers and unusual parameters, but every test has its limitations and alerts that are not important in a particular case may appear. Conversely, the absence of alerts does not guarantee there are no aspects of the results needing attention. It is up to the individual to critically assess their own results and, if necessary, seek expert advice.

Back to contents

Publication of your CIF in IUCr journals

A basic structural check has been run on your CIF. These basic checks will be run on all CIFs submitted for publication in IUCr journals (*Acta Crystallographica*, *Journal of Applied Crystallography*, *Journal of Synchrotron Radiation*); however, if you intend to submit to *Acta Crystallographica Section C* or *E* or *IUCrData*, you should make sure that full publication checks are run on the final version of your CIF prior to submission.

Publication of your CIF in other journals

Please refer to the *Notes for Authors* of the relevant journal for any special instructions relating to CIF submission.

PLATON version of 06/01/2024; check.def file version of 05/01/2024

Datablock 240203lt_auto - ellipsoid plot

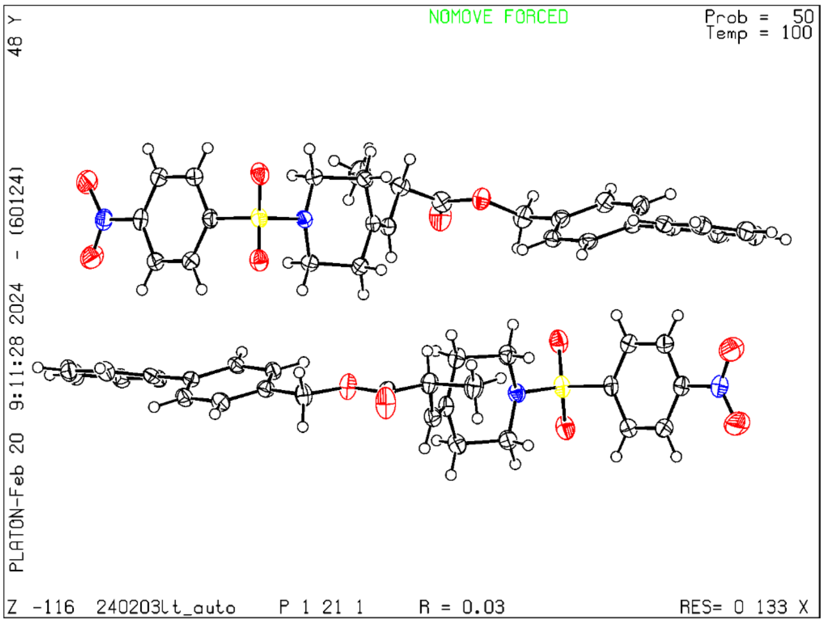

8. References

1. Xiao, Q.; Young, K.; Zakarian, A. An efficient synthesis of the fully elaborated isoindolinone unit of muironolide A. *Org. Lett.* **2013**, *15*, 3314–3317.
2. Gemma, S.; Kunjir, S.; Coccone, S. S.; Brindisi, M.; Moretti, V.; Brogi, S.; Novellino, E.; Basilico, N.; Parapini, S.; Taramelli, D.; Campiani, G.; Butini, S. Synthesis and antiplasmodial activity of bicyclic dioxanes as simplified dihydroplakortin analogues. *J. Med. Chem.* **2011**, *54*, 5949–5953.
3. Xavier, T.; Condon, S.; Pichon, C.; Le Gall, E.; Presset, M. Synthesis of *α,β*-disubstituted acrylates via Galat reaction. *Org. Lett.* **2019**, *21*, 6135–6139.
4. Huang, D. S.; Hartwig, J. F. Palladium-Catalyzed *γ*-Arylation of *α,β*-Unsaturated Esters from Silyl Ketene Acetals. *Angew. Chem., Int. Ed.* **2010**, *49*, 5757–5761.
5. Kapferer, T.; Brückner, R. Asymmetric Dihydroxylation of *β,γ*-Unsaturated Carboxylic Esters with Trisubstituted C═C Bonds—Enantioselective Syntheses of Trisubstituted Γ-Butyrolactones. *Eur. J. Org. Chem.* **2006**, *2006*, 2119–2133.
6. Henin, F.; Mortezaei, R.; Muzart, J.; PETE, J. P.; Piva, O. Photodeconjugaison enantioelective d'esters et de lactones conjuguees en presence d'ephedrine. *Tetrahedron* **1989**, *45*, 6171–6196.
7. Adam, W.; Albert, R.; Grau, N. D.; Hasemann, L.; Nestler, B.; Peters, E. M.; Peters, K.; Prechtl, F.; Schnering, H. G. V. Synthesis of *α*-methylene *β*-lactones, novel heterocycles. *J. Org. Chem.* **1991**, *56*, 5778–5781.
8. Garnier, J. M.; Robin, S.; Guillot, R.; Rousseau, G. Preparation of enantiopure 3,5,5-trialkyl-*γ*-butyrolactones by diastereospecific 5-endo halo lactonizations. *Tetrahedron: Asymmetry* **2007**, *18*, 1434–1442.
9. Morack, T.; Onneken, C.; Nakakohara, H.; Mück-Lichtenfeld, C.; Gilmour, R. Enantiodivergent prenylation via deconjugative isomerization. *ACS Catalysis*, **2021**, *11*, 11929–11937.
10. Patterson, L. K.; Porter, G.; Topp, M. R. Oxygen quenching of singlet and triplet states. *Chem. Phys. Lett.*, **1970**, *7*, 612–614.
11. Gijzeman, O. L. J.; Kaufman, F.; Porter, G. Oxygen quenching of aromatic triplet states in solution. Part 1. *J. Chem. Soc., Faraday Trans. 2*, **1973**, *69*, 708–720.
12. Redmond, R. W.; Gamlin, J. N. A compilation of singlet oxygen yields from biologically relevant molecules. *Photochem. Photobiol.*, **1999**, *70*, 391–475.
13. DeRosa, M. C.; Crutchley, R. J. Photosensitized singlet oxygen and its applications. *Coord. Chem. Rev.*, **2002**, *233*, 351–371.
14. Frisch, M. J.; Trucks, G. W.; Schlegel, H. B.; Scuseria, G. E.; Robb, M. A.; Cheeseman, J. R.; Scalmani, G.; Barone, V.; Petersson, G. A.; Nakatsuji, H.; ,et al. *Gaussian 16*, Revision 16.A.03; Gaussian Inc.: Wallingford, CT, 2016.
15. Becke, A. D. Density-functional thermochemistry. III. The role of exact exchange. *J. Chem. Phys.* **1993**, *98*, 5648–5652.
16. Lee, C.; Yang, W.; Parr, R. G. Development of the Colle-Salvetti correlation-energy formula into a functional of the electron density. *Phys. Rev. B*, **1988**, *37*, 785.
17. Vosko, S. H.; Wilk, L.; Nusair, M. Accurate spin-dependent electron liquid correlation energies for local spin density calculations: a critical analysis. *Can. J. Phys.*, **1980**, *58*, 1200–1211.

18. Krishnan, R.; Binkley, J. S.; Seeger, R.; Pople, J. A. Self-consistent molecular orbital methods. XX. A basis set for correlated wave functions. *J. Chem. Phys.*, **1980**, *72*, 650–654.
19. McLean, A. D.; Chandler, G. S. Contracted Gaussian basis sets for molecular calculations. I. Second row atoms, Z= 11–18. *J. Chem. Phys.*, **1980**, *72*, 5639–5648.
20. Frisch, M. J.; Pople, J. A.; Binkley, J. S. Self-consistent molecular orbital methods 25. Supplementary functions for Gaussian basis sets. *J. Chem. Phys.*, **1984**, *80*, 3265–3269.
21. Clark, T.; Chandrasekhar, J.; Spitznagel, G. W.; Schleyer, P. V. R. Efficient diffuse function-augmented basis sets for anion calculations. III. The 3-21+ G basis set for first-row elements, Li–F. *J. Comput. Chem.*, **1983**, *4*, 294–301.
22. Tomasi, J.; Mennucci, B.; Cammi, R. Quantum mechanical continuum solvation models. *Chem. Rev.*, **2005**, *105*, 2999–3094.
23. Marenich, A. V.; Cramer, C. J.; Truhlar, D. G. Universal solvation model based on solute electron density and on a continuum model of the solvent defined by the bulk dielectric constant and atomic surface tensions. *J. Phys. Chem. B*, **2009**, *113*, 6378–6396.

[Back to contents](#)

9. NMR Spectra

^{1}H NMR (400 MHz, $CDCl_3$) of **S1d**, See procedure

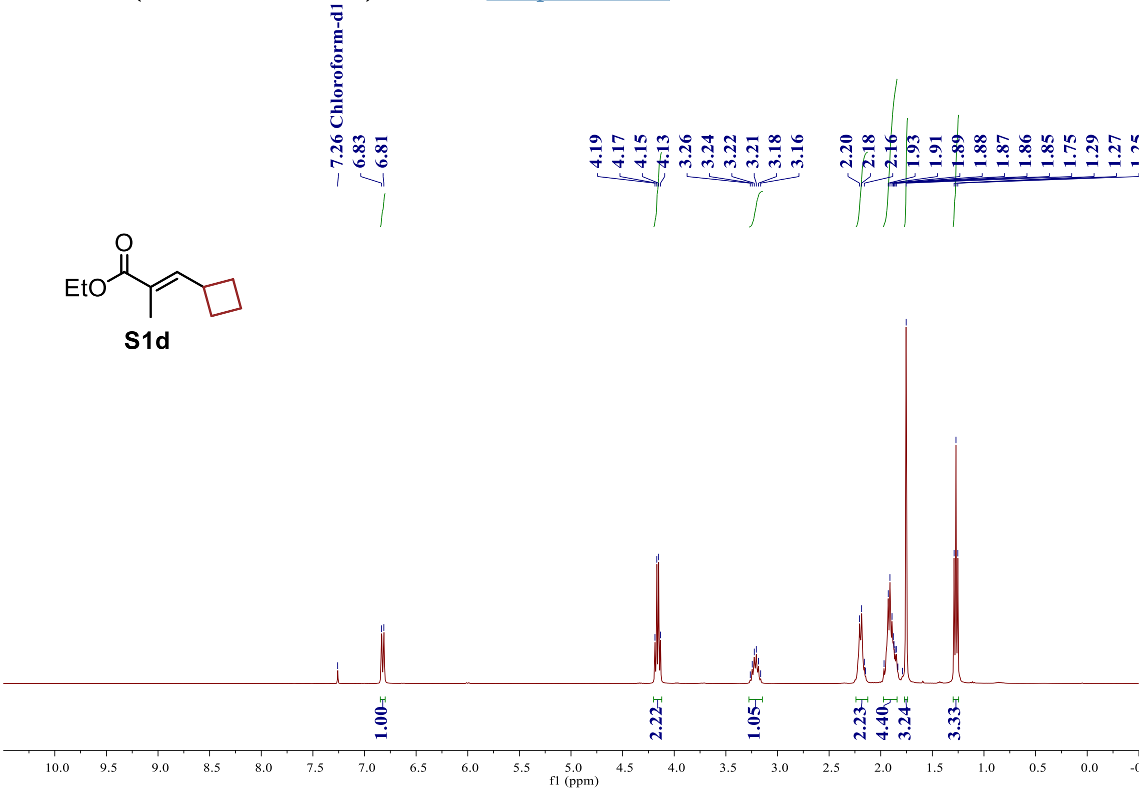

^{13}C{^{1}H} NMR (101 MHz, $CDCl_3$) of **S1d**

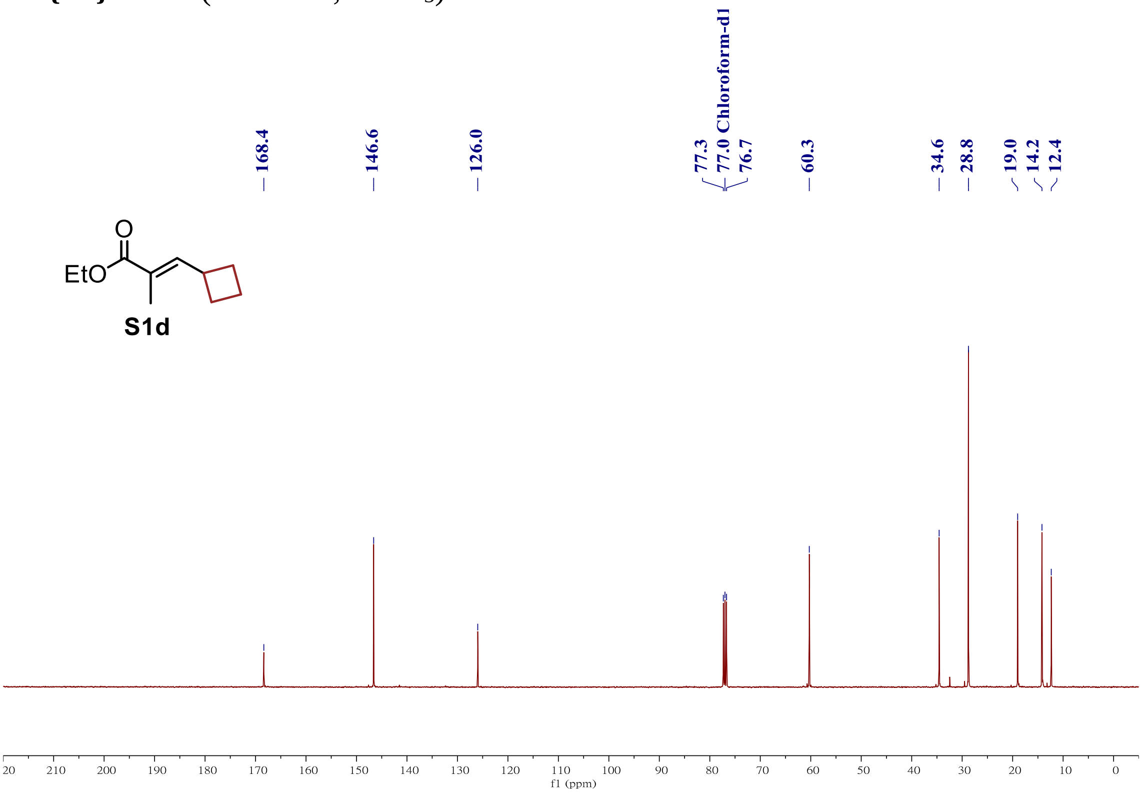

Back to contents

^{1}H NMR (400 MHz, $CDCl_3$) of **S1g**, See procedure

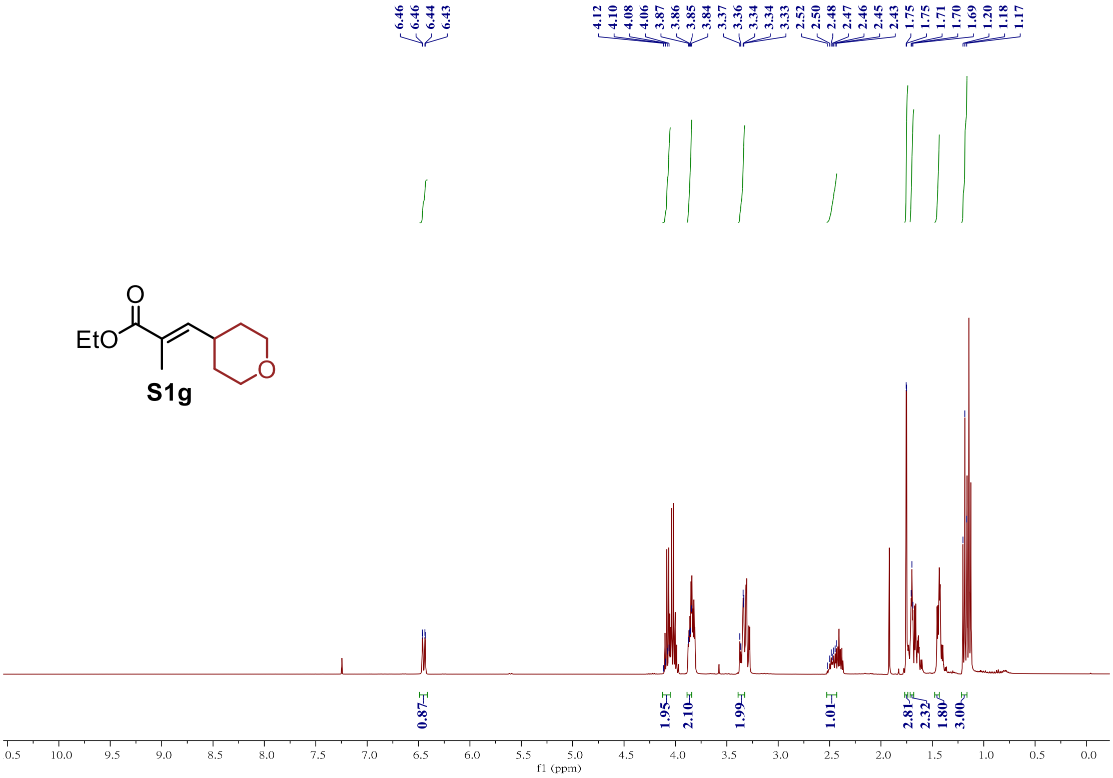

^{13}C{^{1}H} NMR (101 MHz, $CDCl_3$) of **S1g**

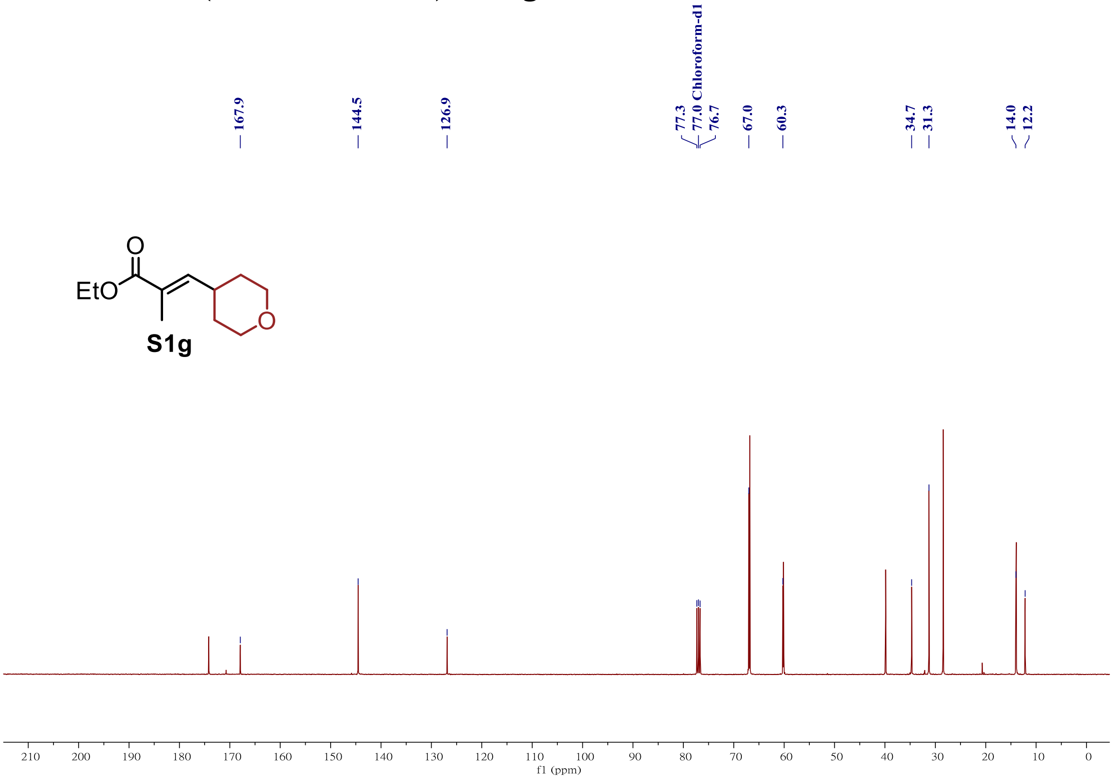

[Back to contents]

^{1}H NMR (300 MHz, $CDCl_3$) of **S1h**, See procedure

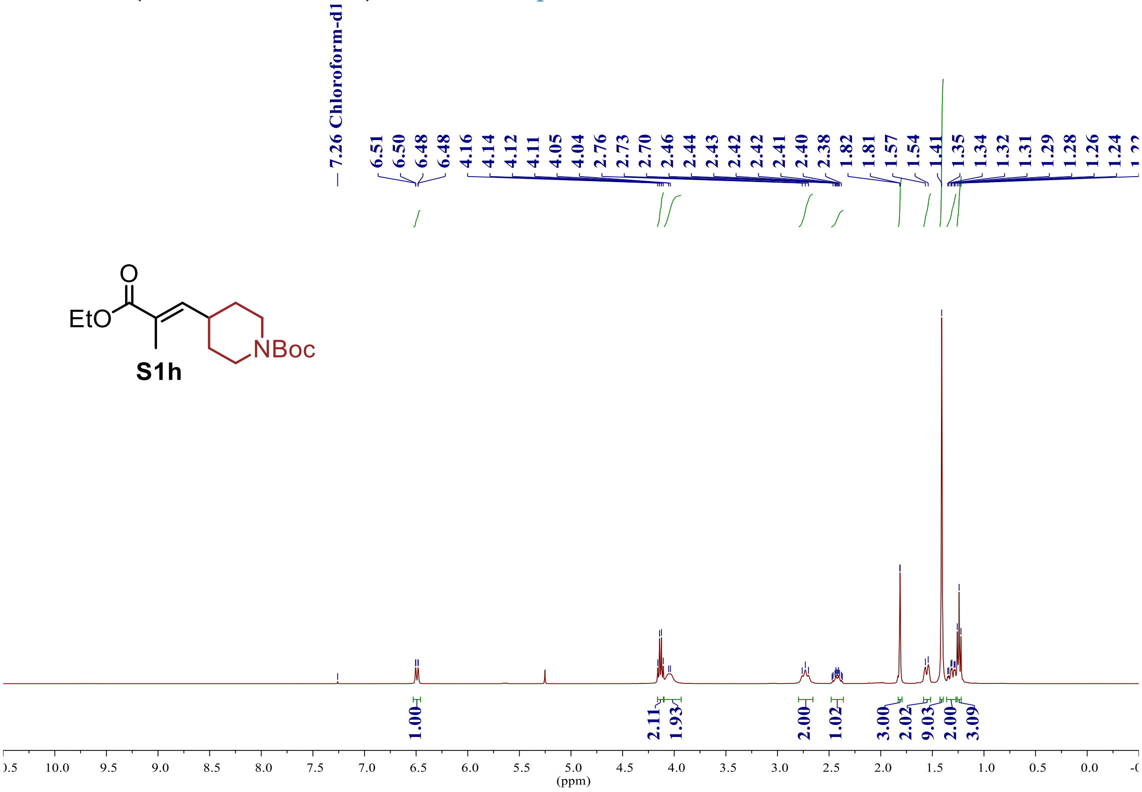

^{13}C{^{1}H} NMR (101 MHz, $CDCl_3$) of **S1h**

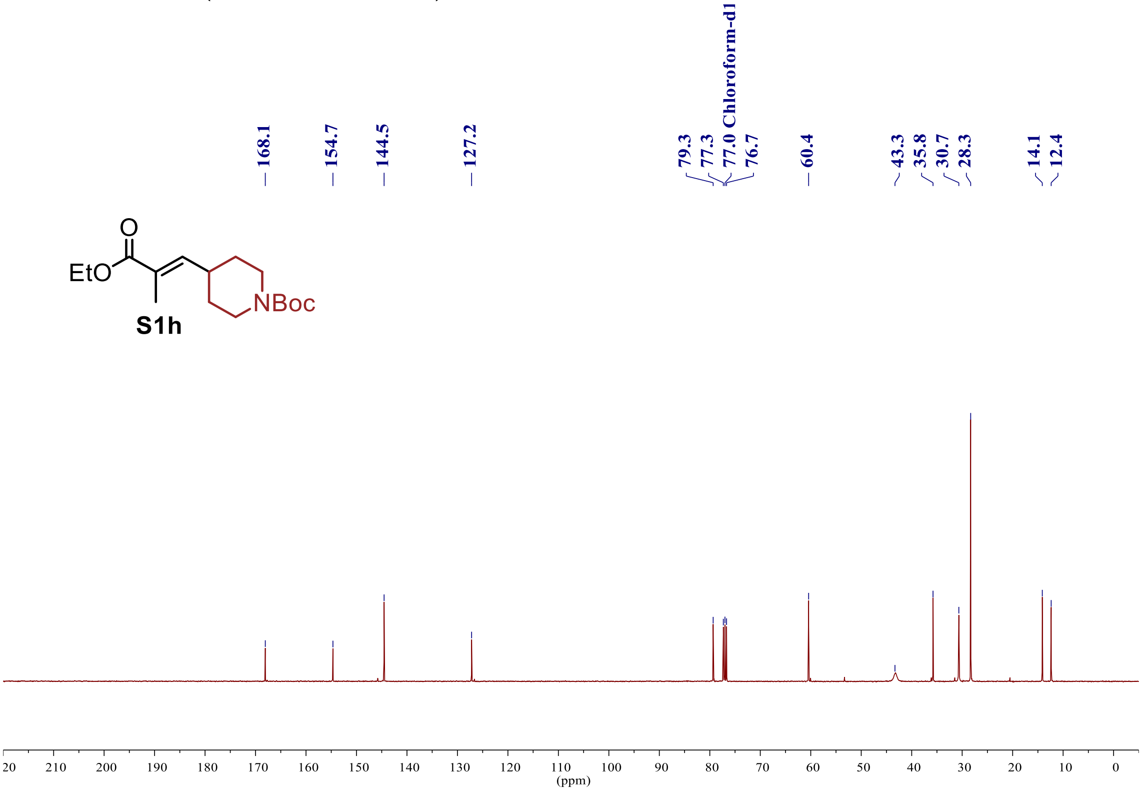

Back to contents

^{1}H NMR (400 MHz, $CDCl_3$) of **S1l**, See procedure

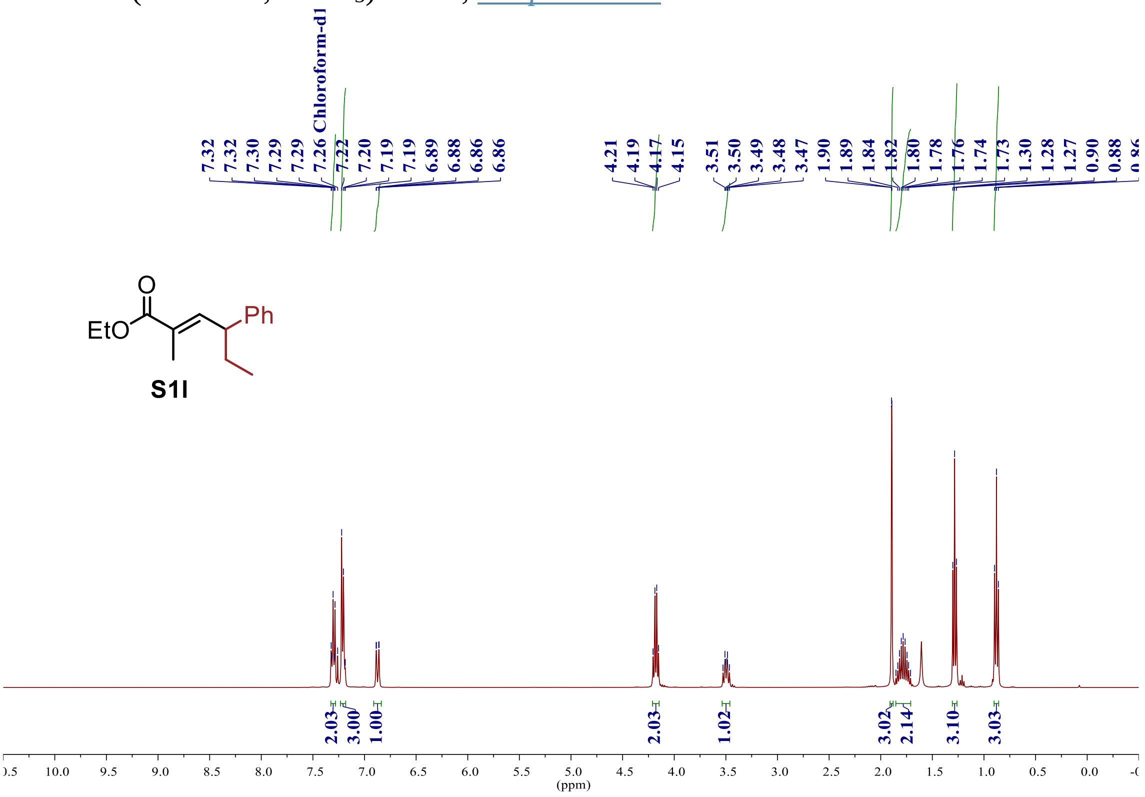

^{13}C{^{1}H} NMR (101 MHz, $CDCl_3$) of **S1l**

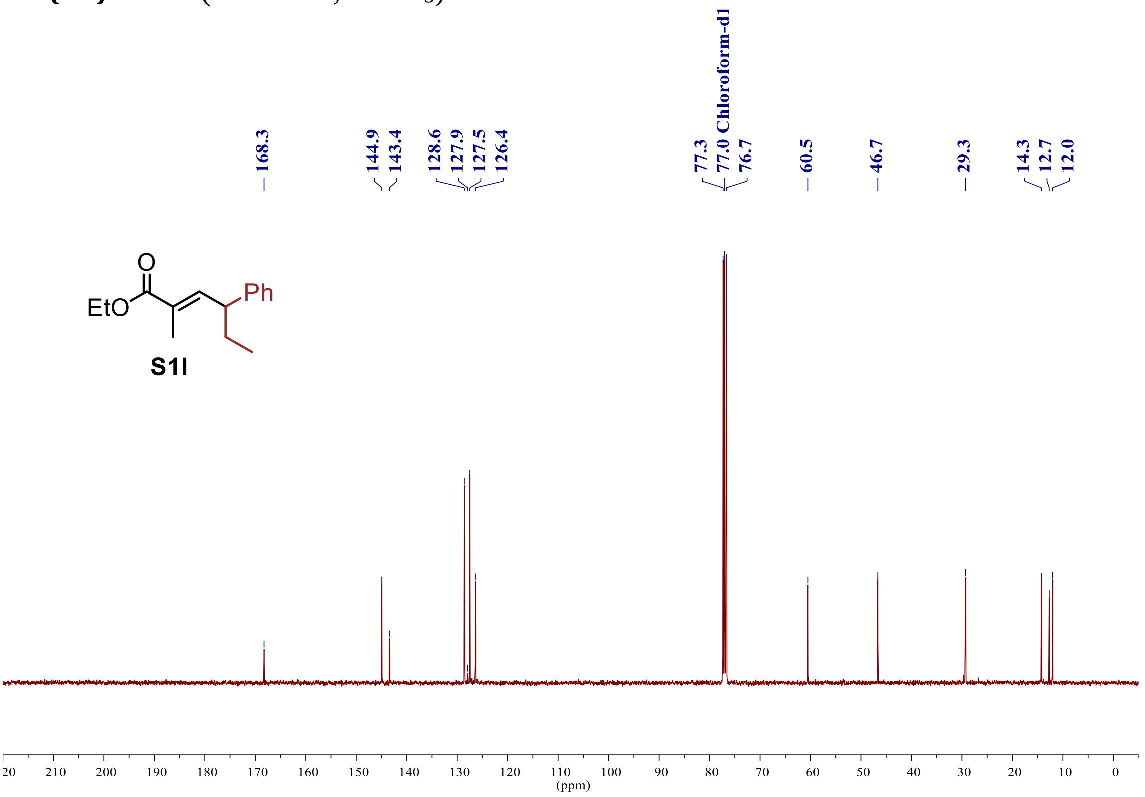

[Back to contents]

^{1}H NMR (300 MHz, $CDCl_3$) of **S1m**, See procedure

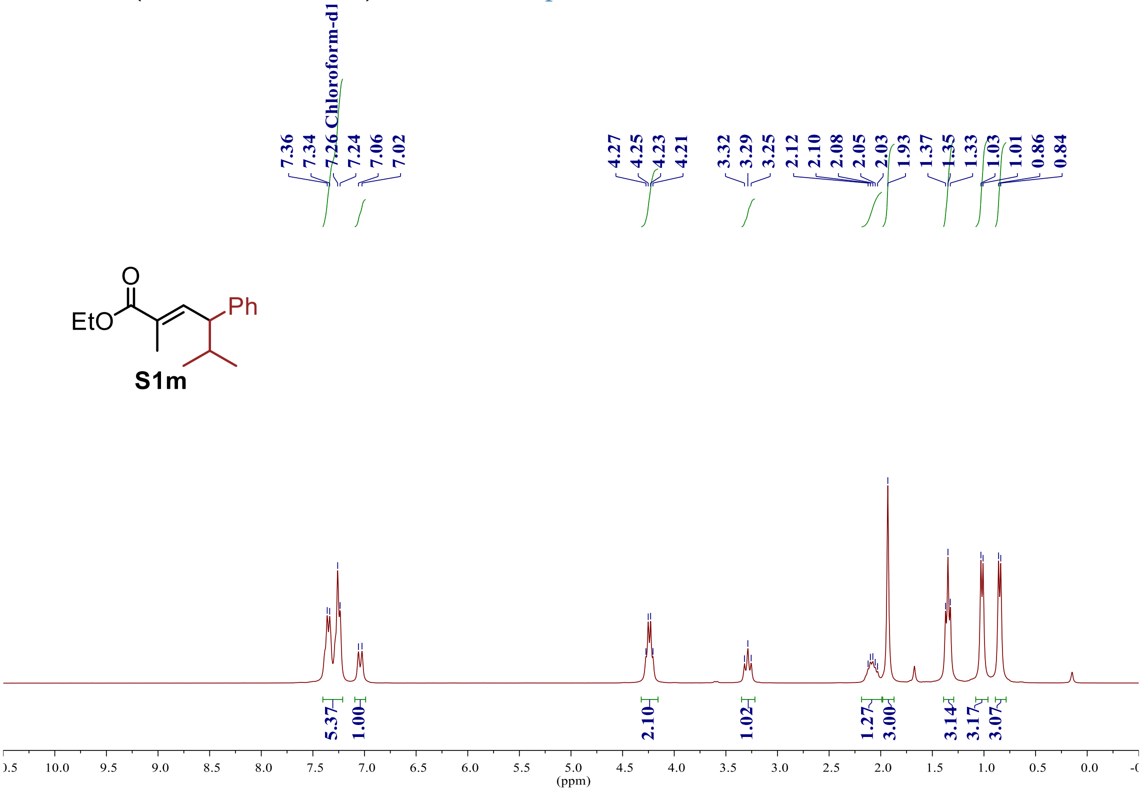

^{13}C{^{1}H} NMR (101 MHz, $CDCl_3$) of **S1m**

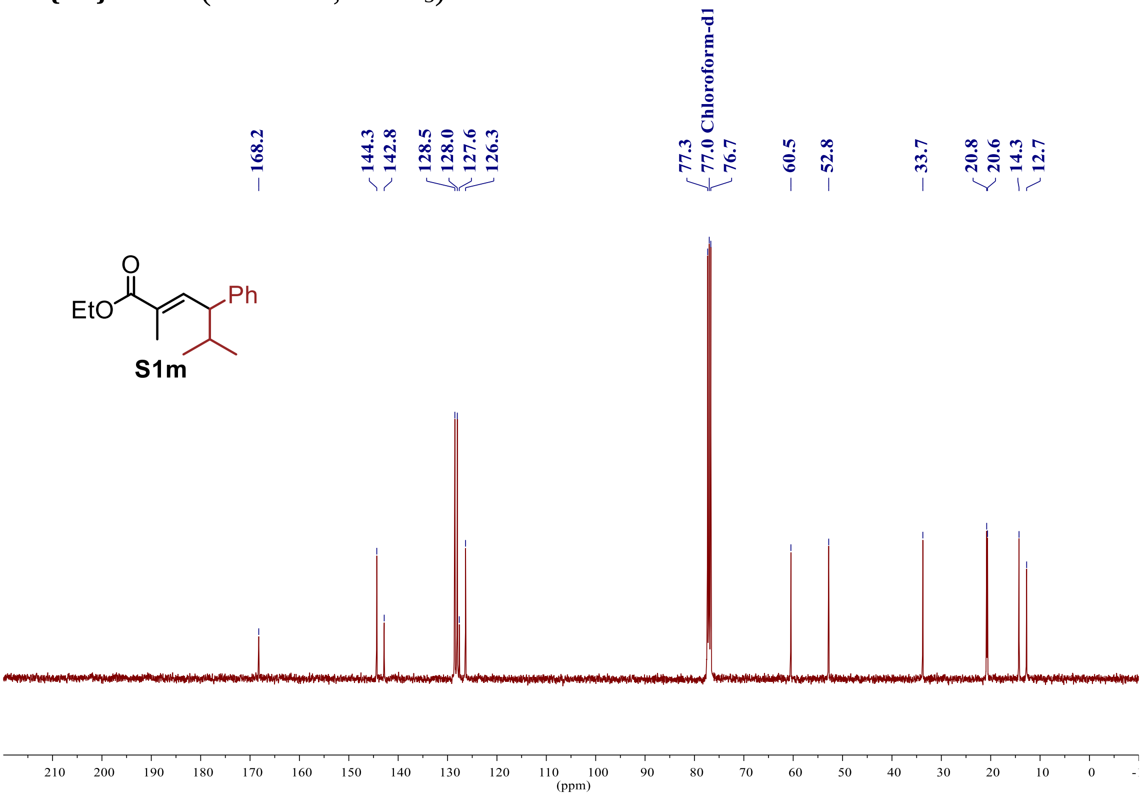

Back to contents

^{1}H NMR (400 MHz, $CDCl_3$) of **S1n**, See procedure

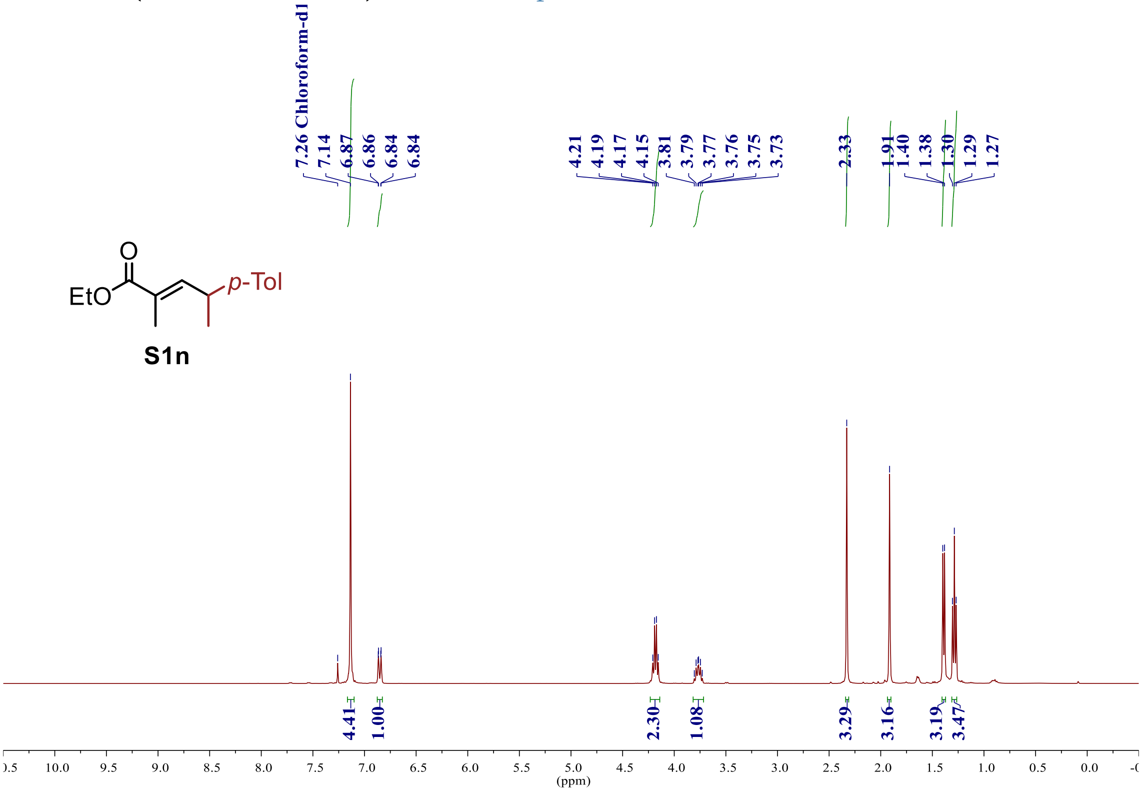

^{13}C{^{1}H} NMR (101 MHz, $CDCl_3$) of **S1n**

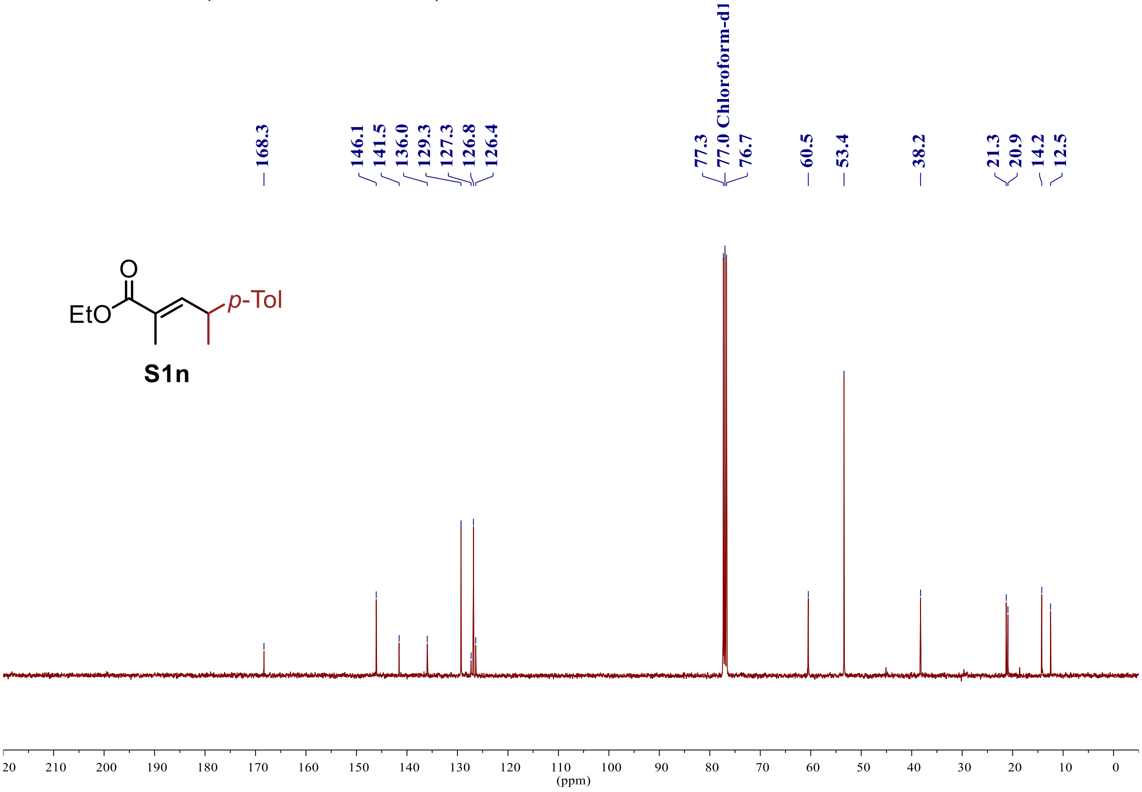

Back to contents

^{1}H NMR (300 MHz, $CDCl_3$) of **1v**, See procedure

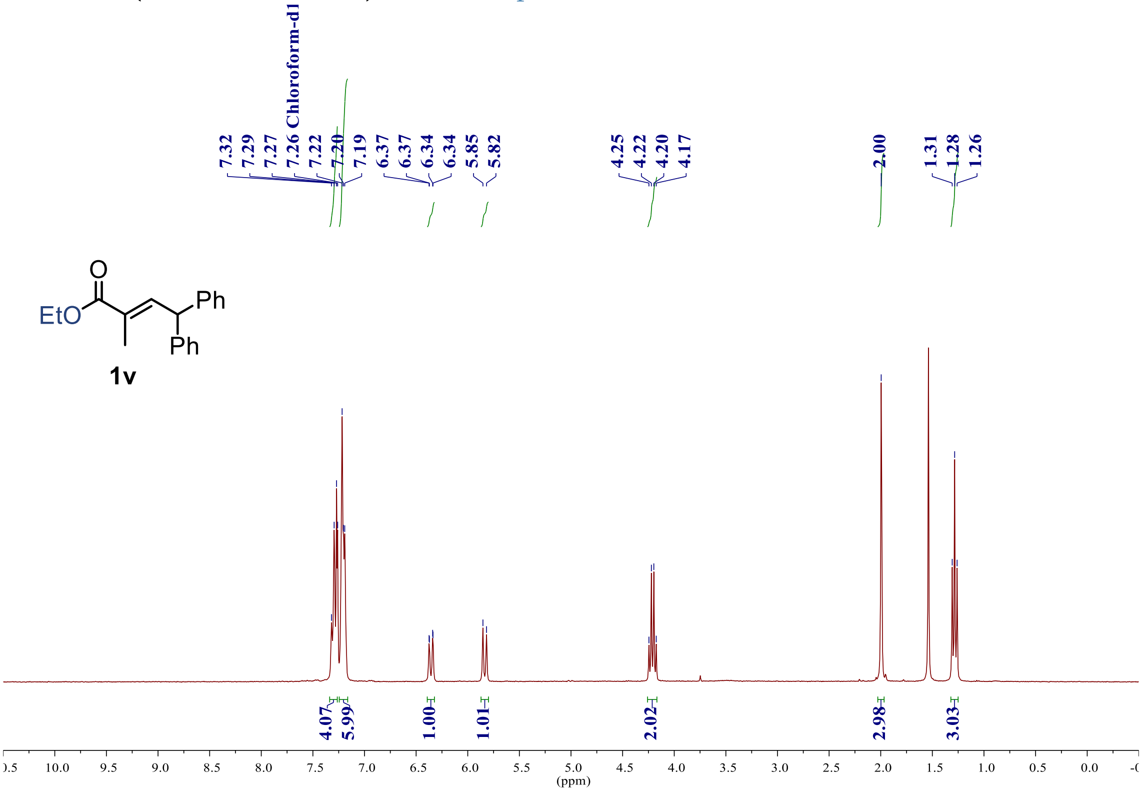

^{13}C{^{1}H} NMR (101 MHz, $CDCl_3$) of **1v**

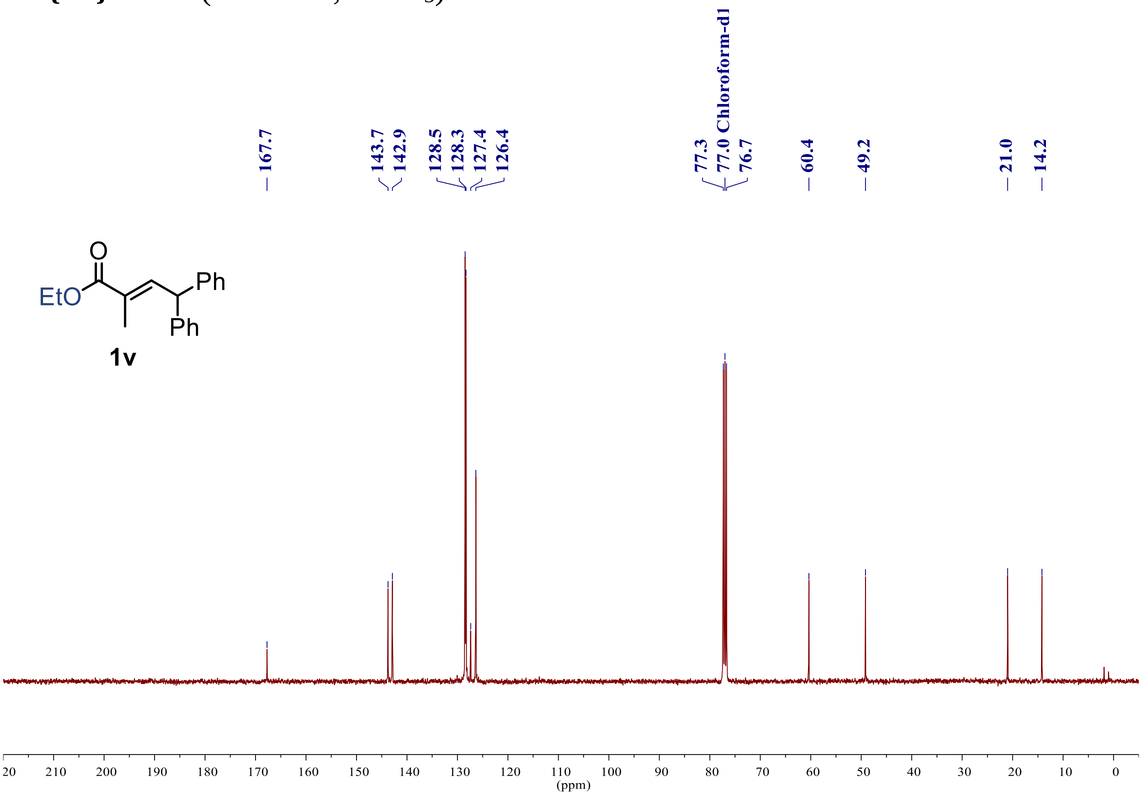

[Back to contents](#)

^{1}H NMR (400 MHz, $CDCl_3$) of **S2a**, [See procedure](#)

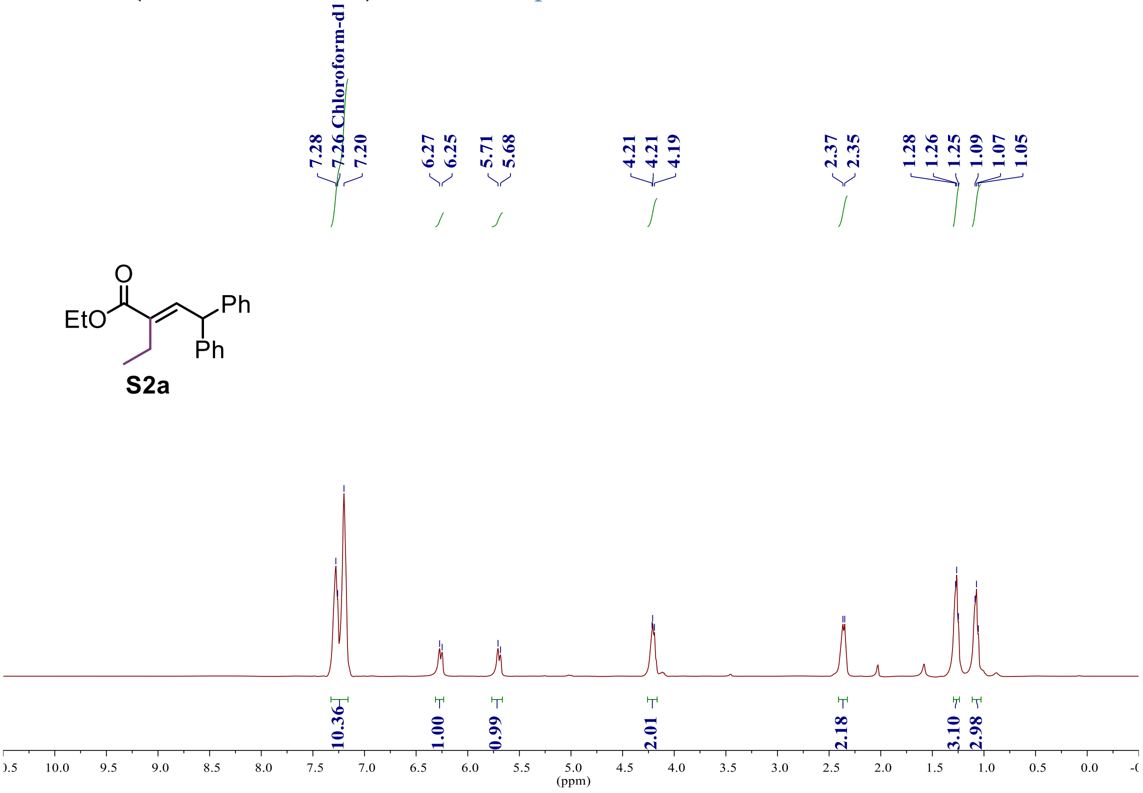

^{13}C{^{1}H} NMR (101 MHz, $CDCl_3$) of **S2a**

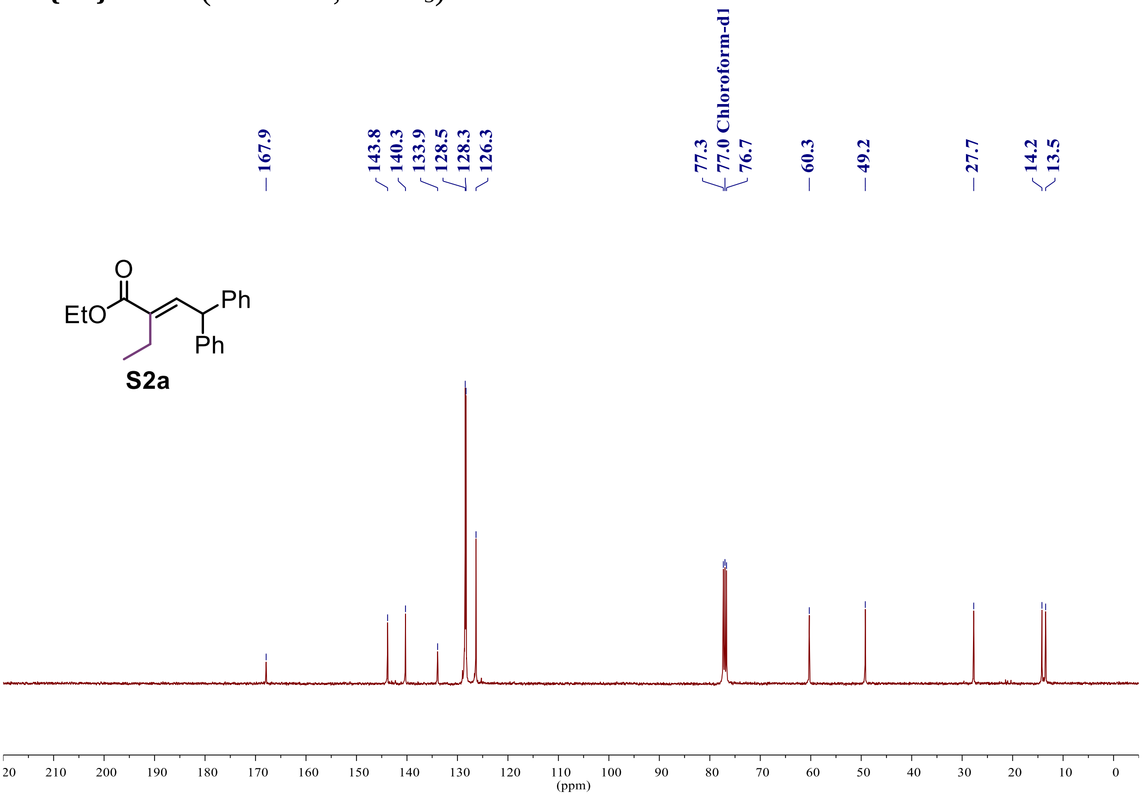

Back to contents

^{1}H NMR (400 MHz, $CDCl_3$) of **S2b**, See procedure

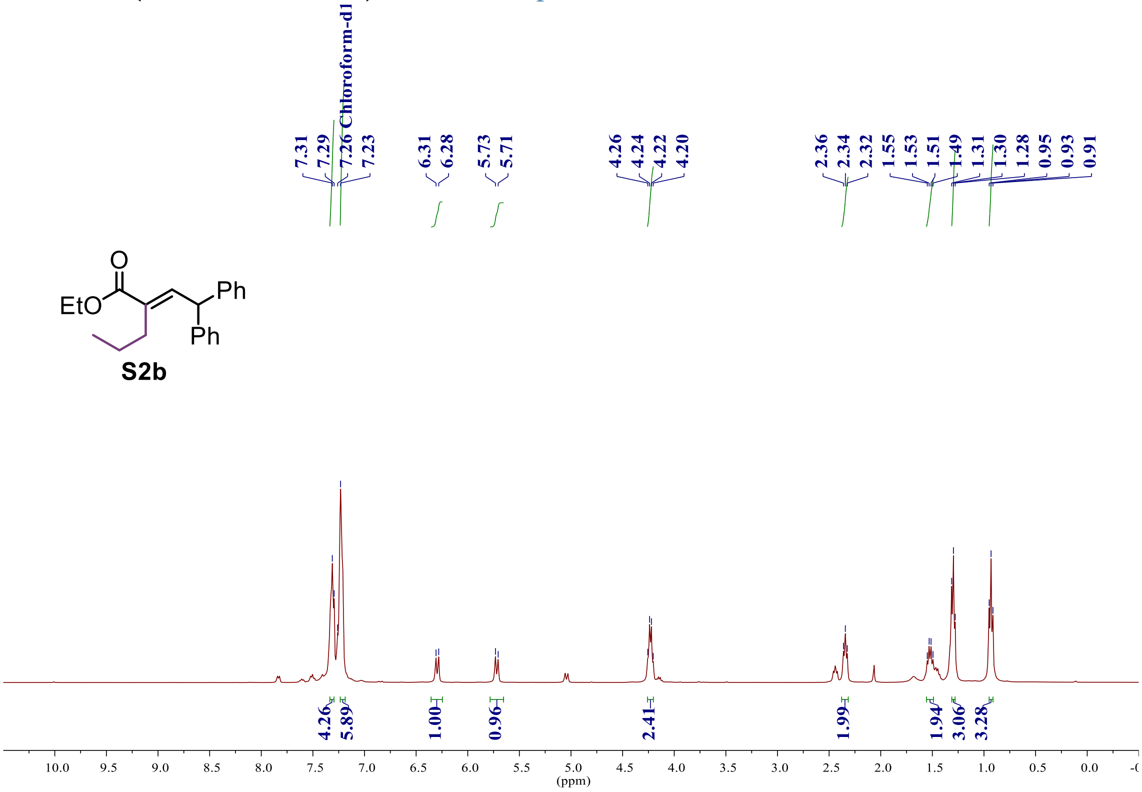

^{13}C{^{1}H} NMR (101 MHz, $CDCl_3$) of **S2b**

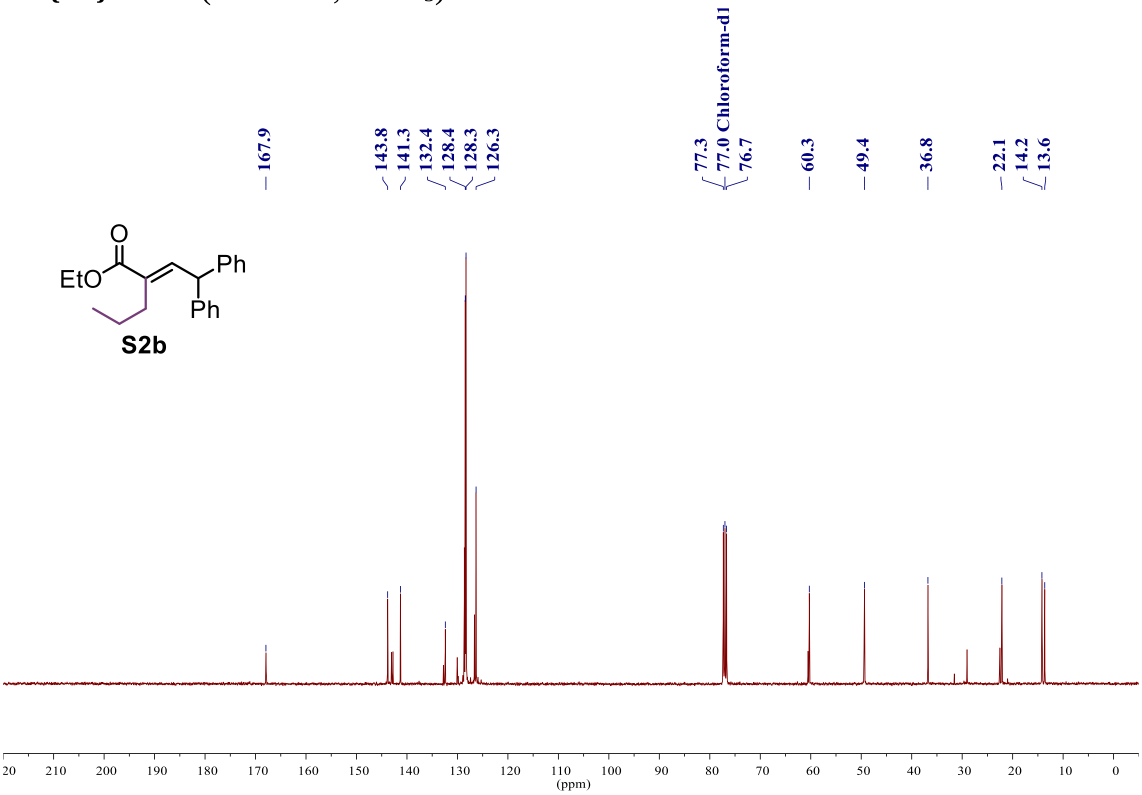

^{1}H NMR (400 MHz, $CDCl_3$) of **S2c**, See procedure

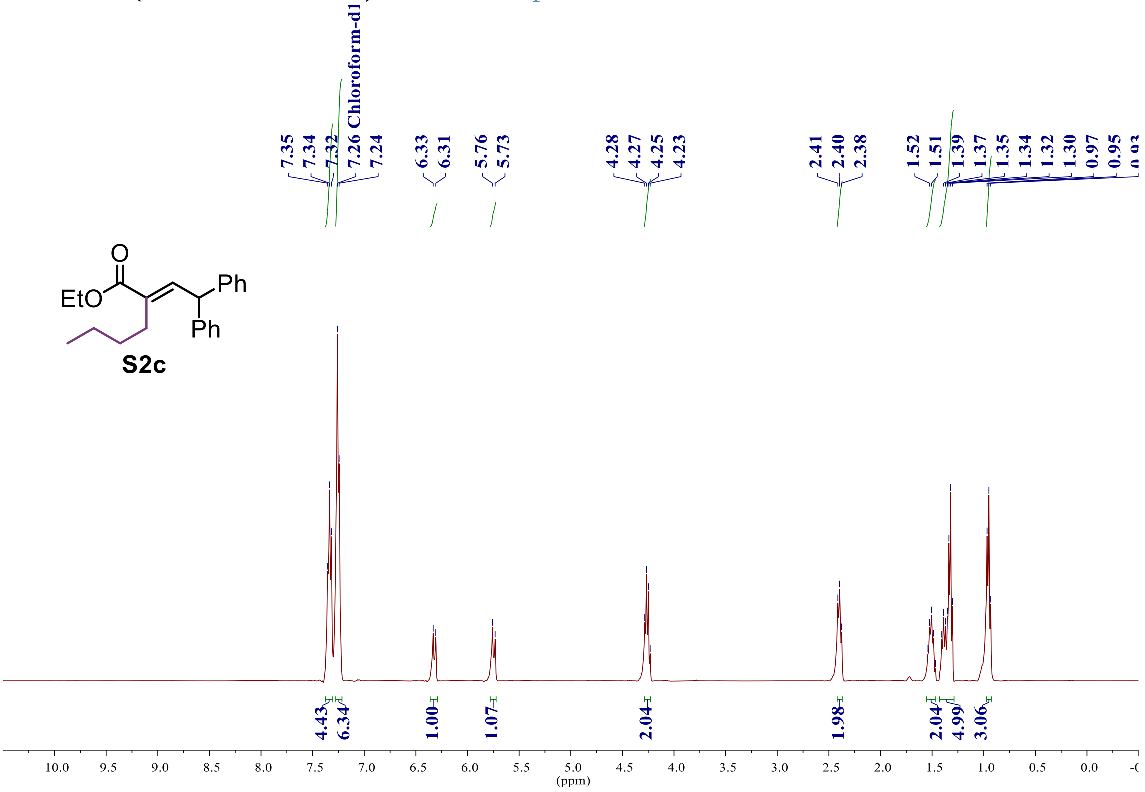

^{13}C{^{1}H} NMR (101 MHz, $CDCl_3$) of **S2c**

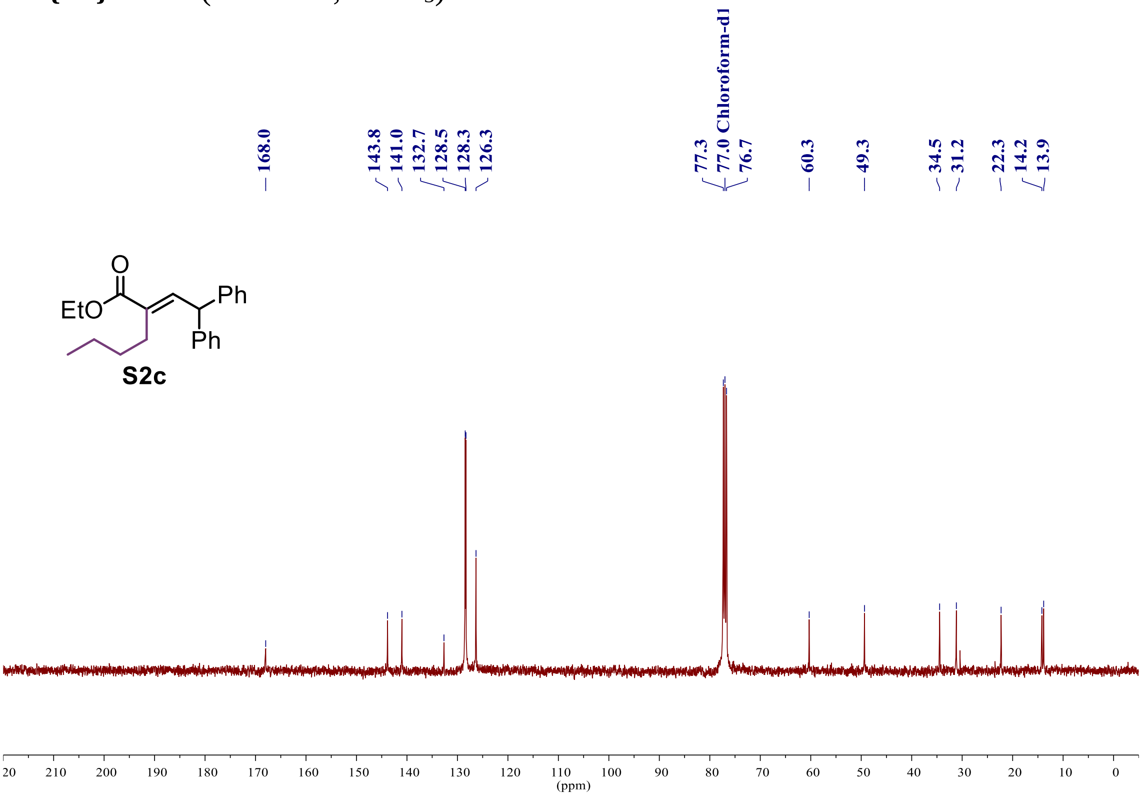

Back to contents

^{1}H NMR (400 MHz, $CDCl_3$) of **S2d**, See procedure

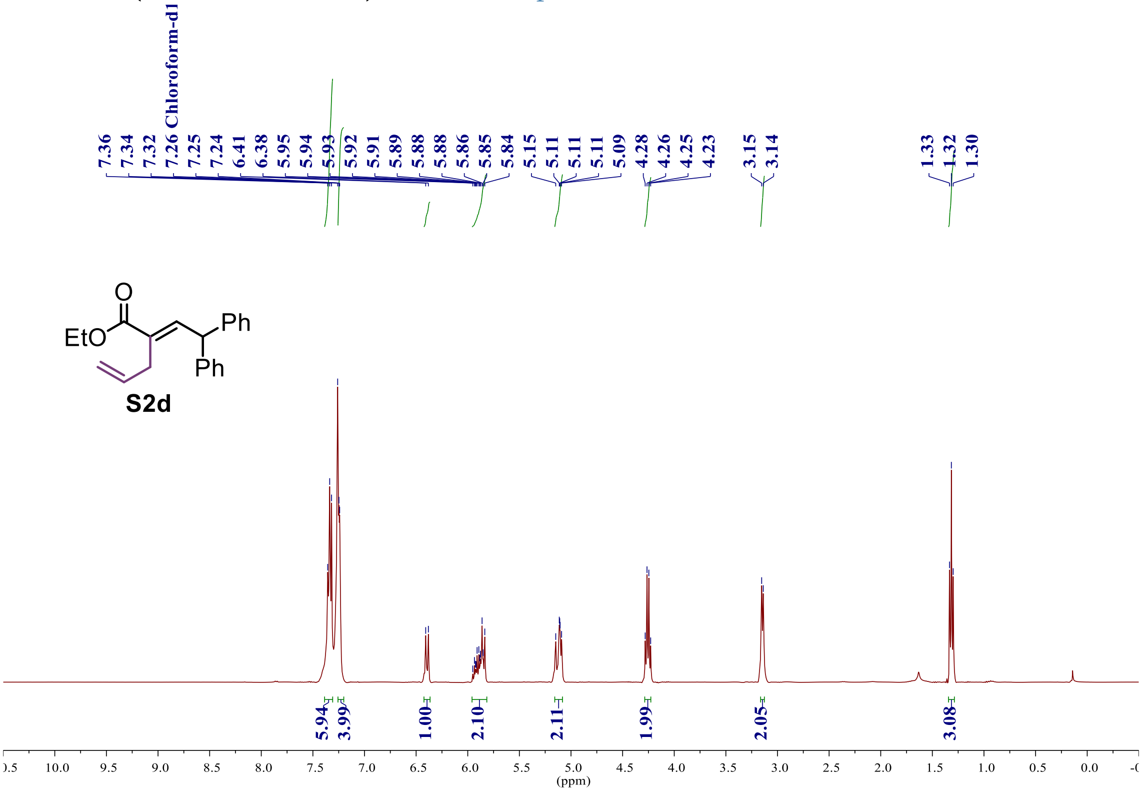

^{13}C{^{1}H} NMR (101 MHz, $CDCl_3$) of **S2d**

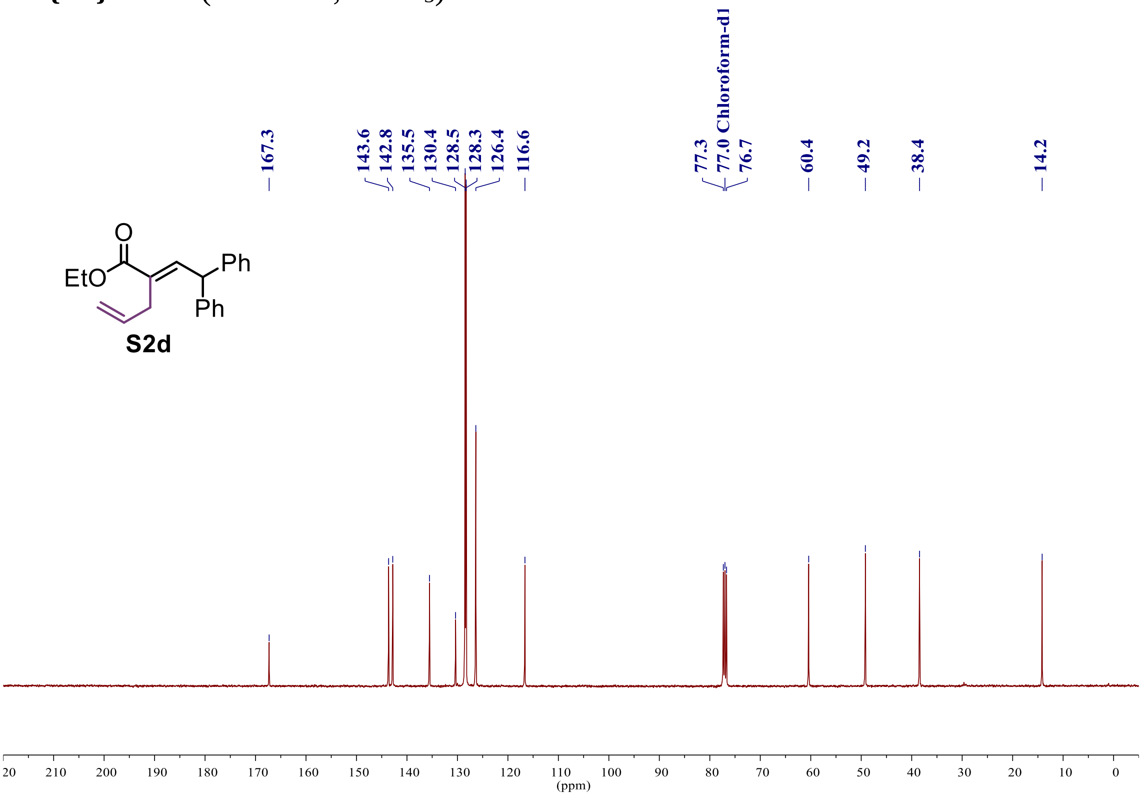

Back to contents

^{1}H NMR (300 MHz, $CDCl_3$) of **S2e**, See procedure

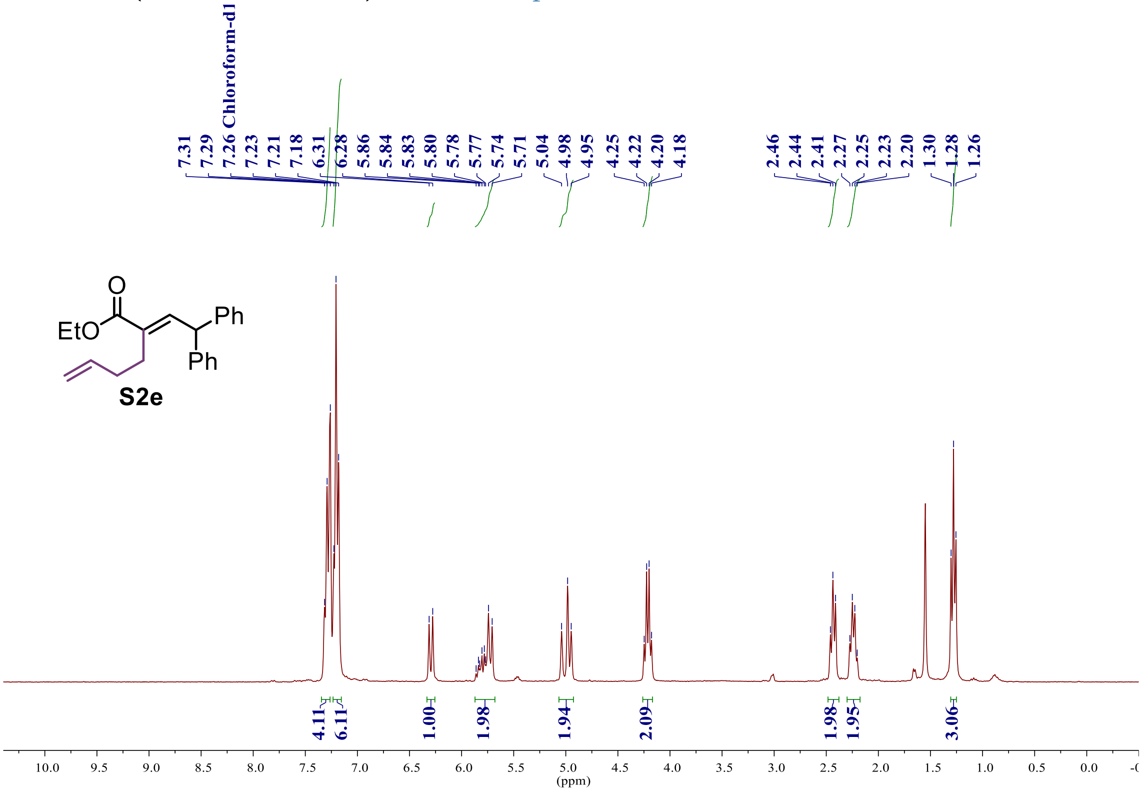

^{13}C{^{1}H} NMR (101 MHz, $CDCl_3$) of **S2e**

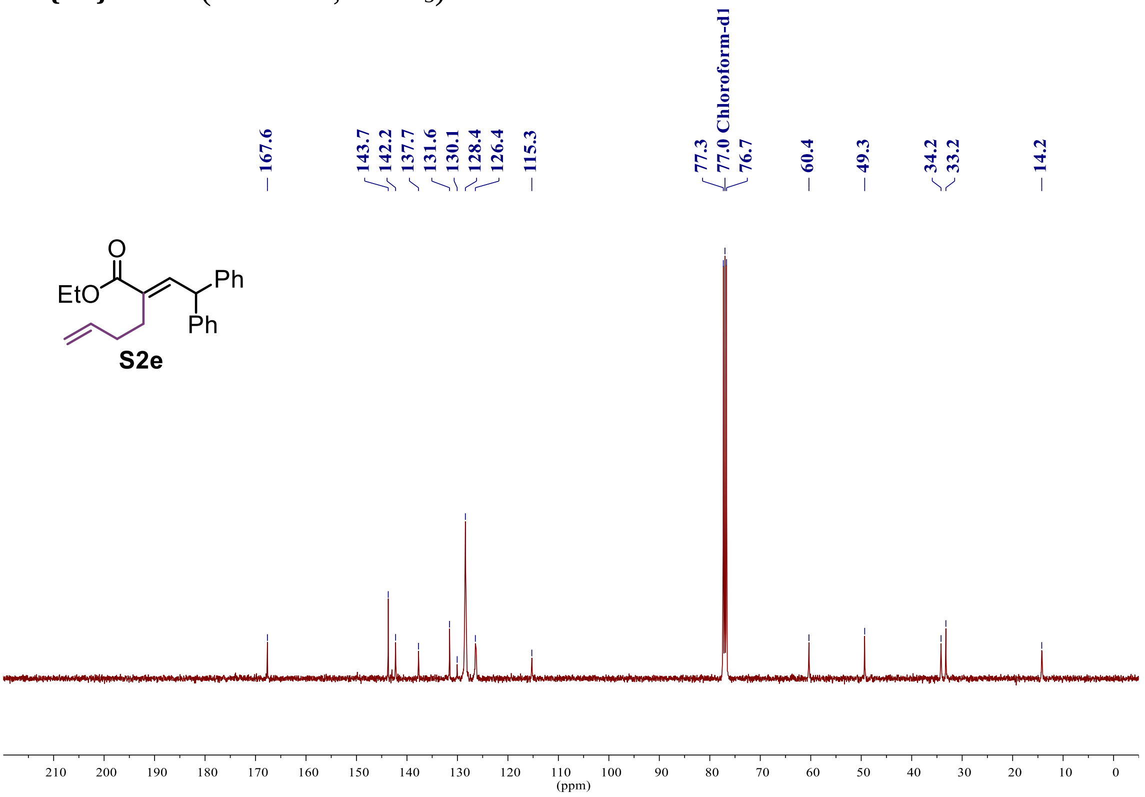

Back to contents

^{1}H NMR (400 MHz, $CDCl_3$) of **S3b**, See procedure

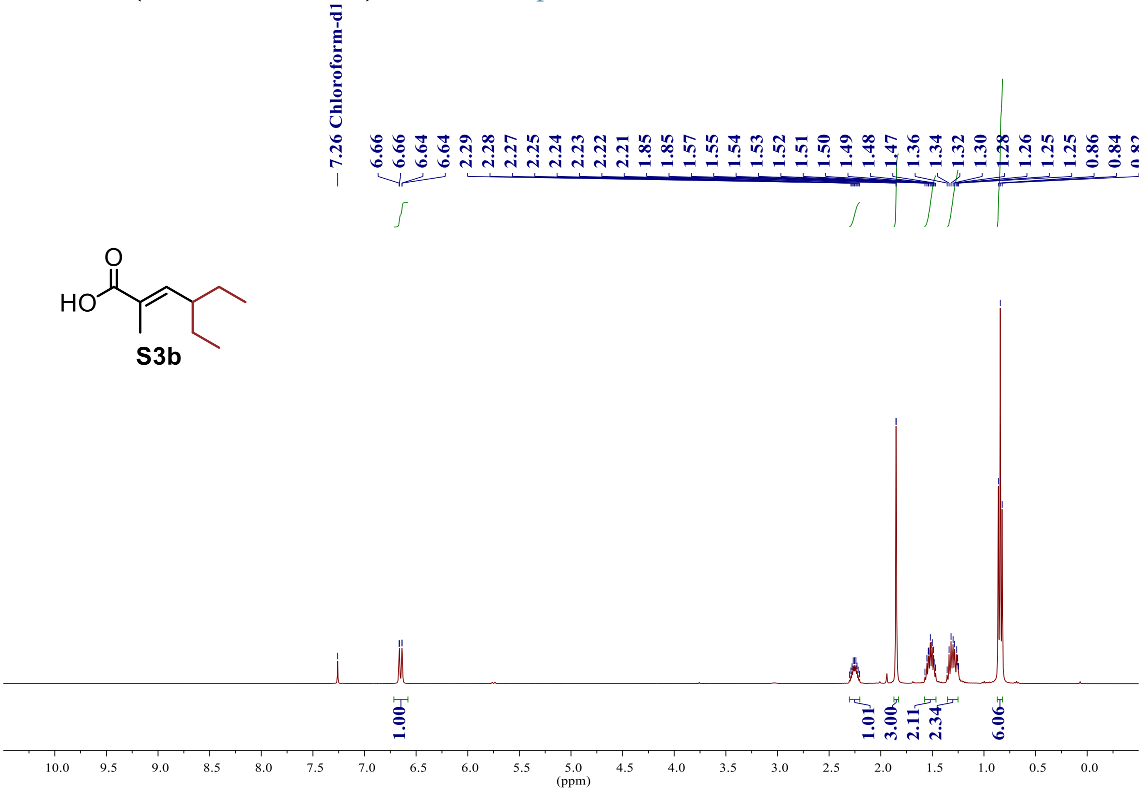

^{13}C{^{1}H} NMR (101 MHz, $CDCl_3$) of **S3b**

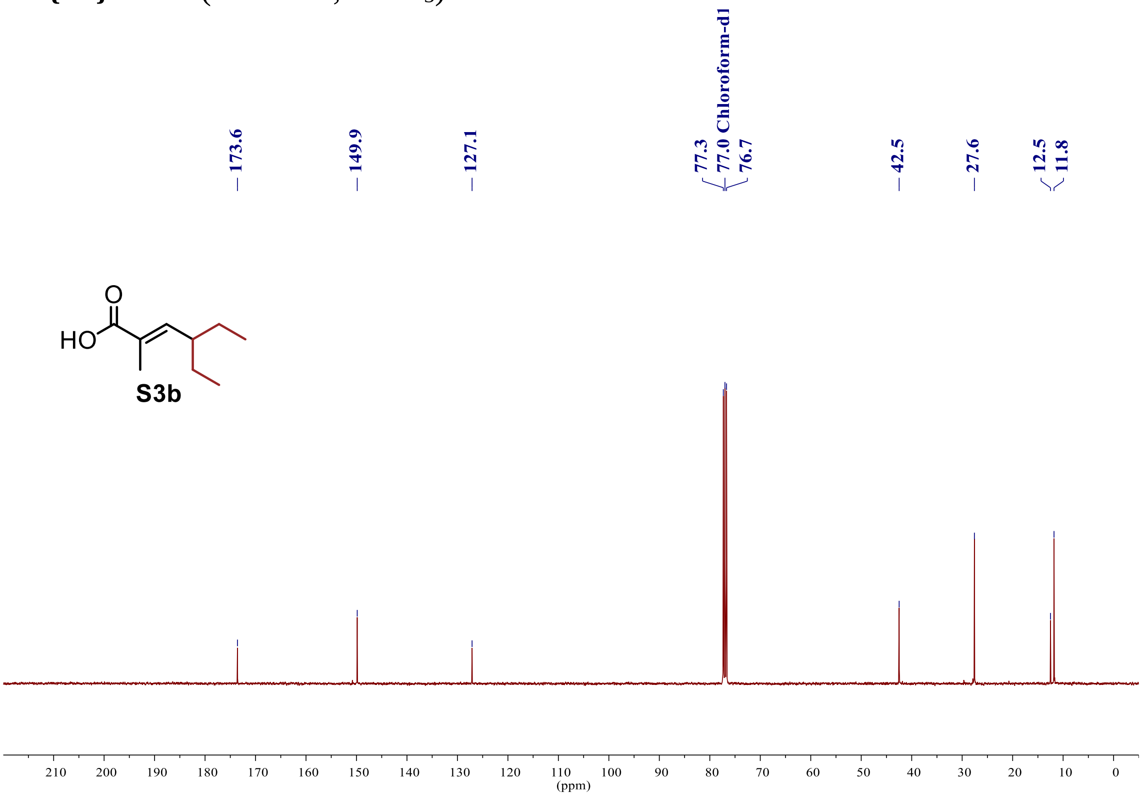

Back to contents

^{1}H NMR (400 MHz, $CDCl_3$) of **S3d**, See procedure

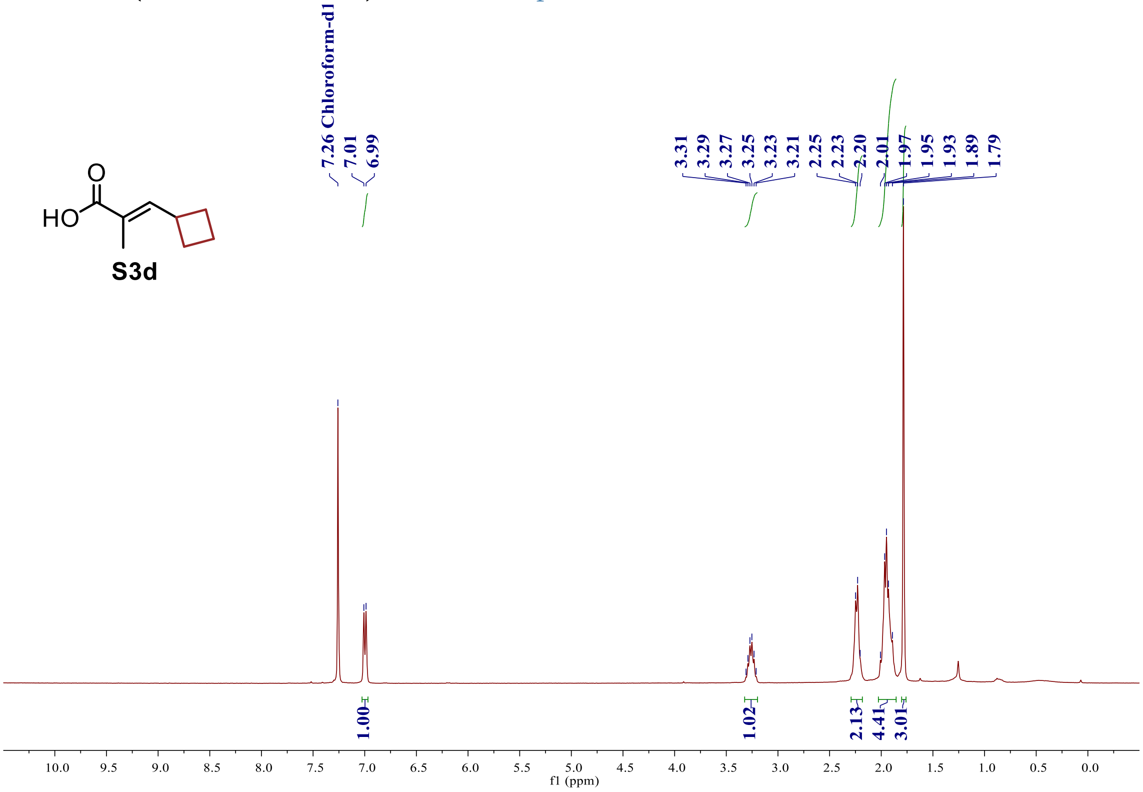

^{13}C{^{1}H} NMR (101 MHz, $CDCl_3$) of **S3d**

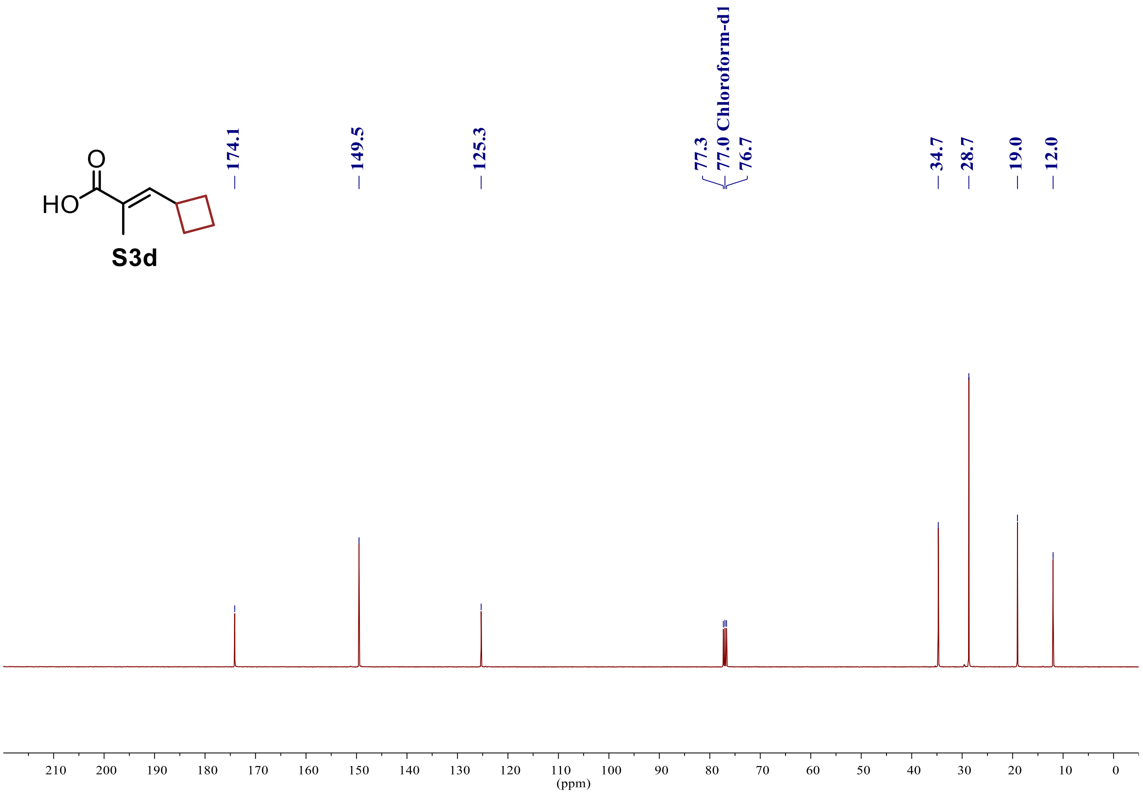

Back to contents

¹H NMR (300 MHz, $CDCl_3$) of **S3e**, See procedure

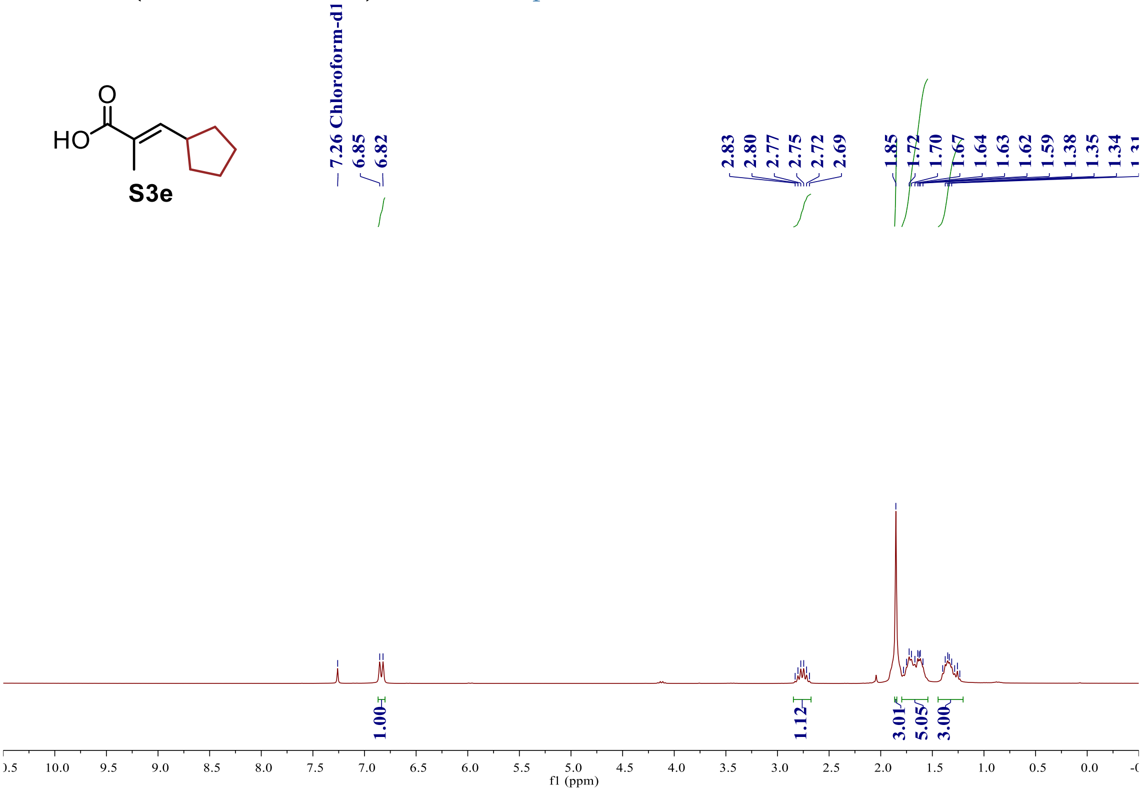

$^{13}C\{^1H\}$ NMR (101 MHz, $CDCl_3$) of **S3e**

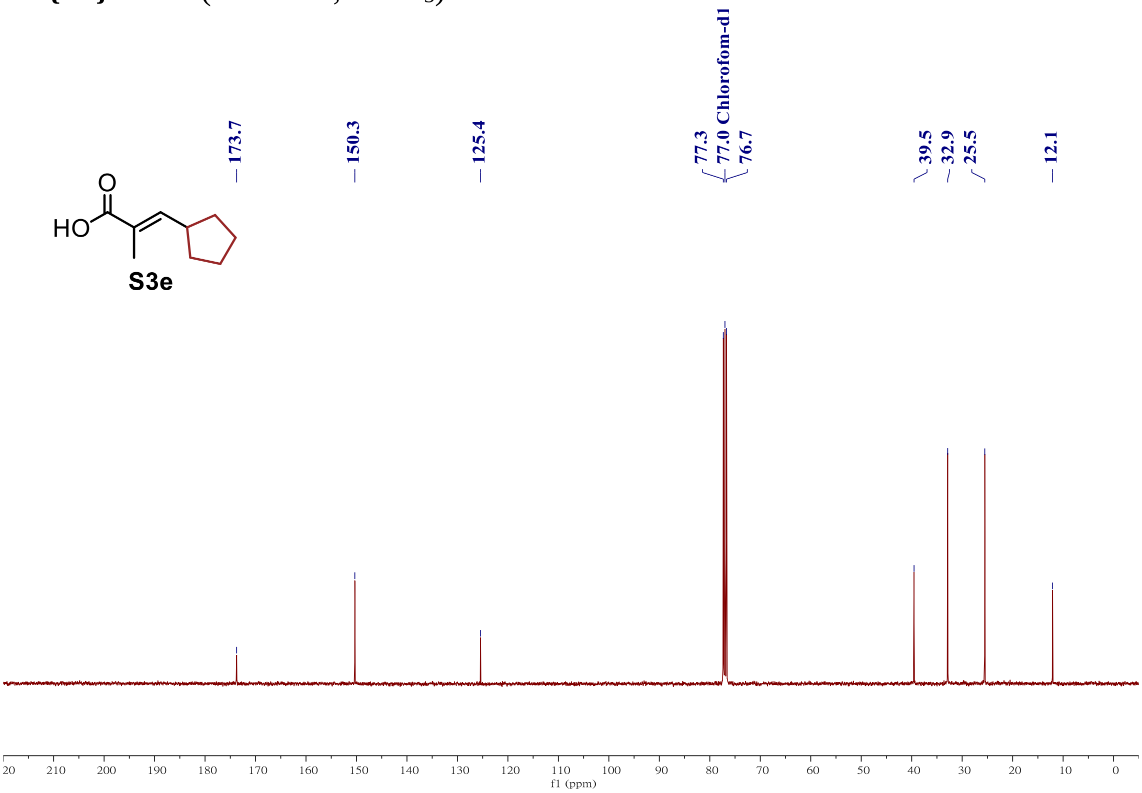

Back to contents

^{1}H NMR (300 MHz, $CDCl_3$) of **S3g**, See procedure

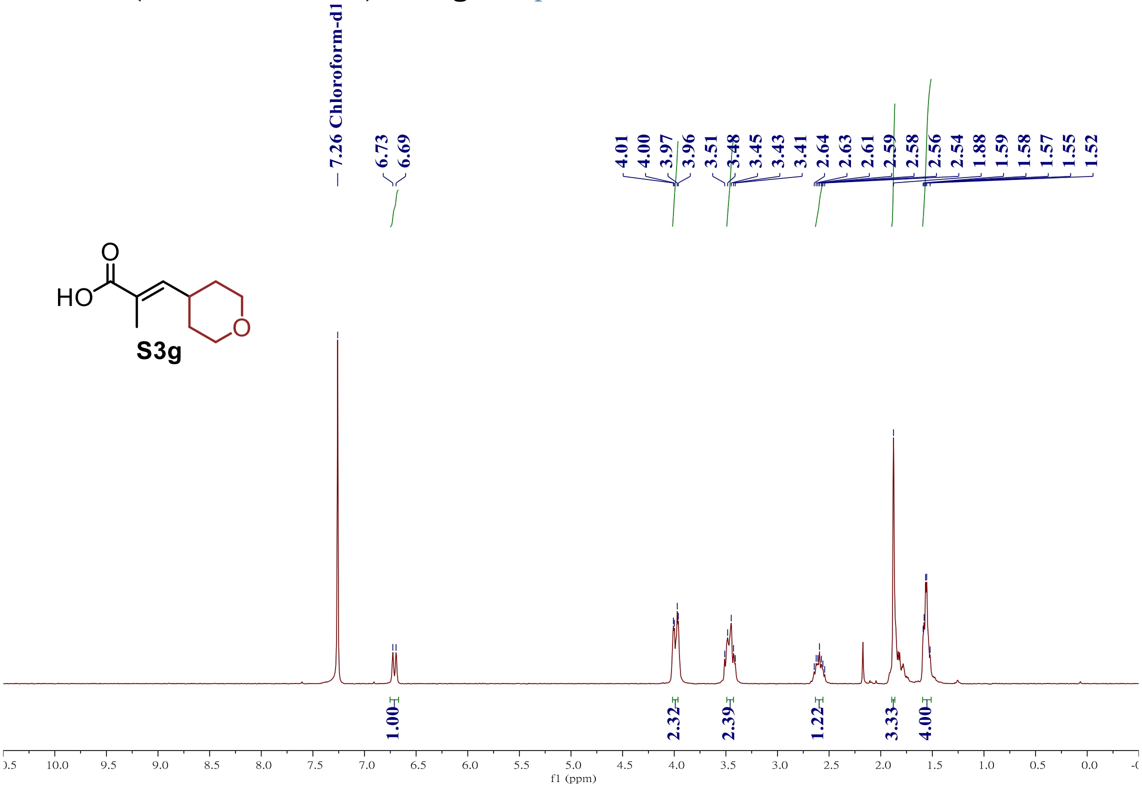

^{13}C{^{1}H} NMR (101 MHz, $CDCl_3$) of **S3g**

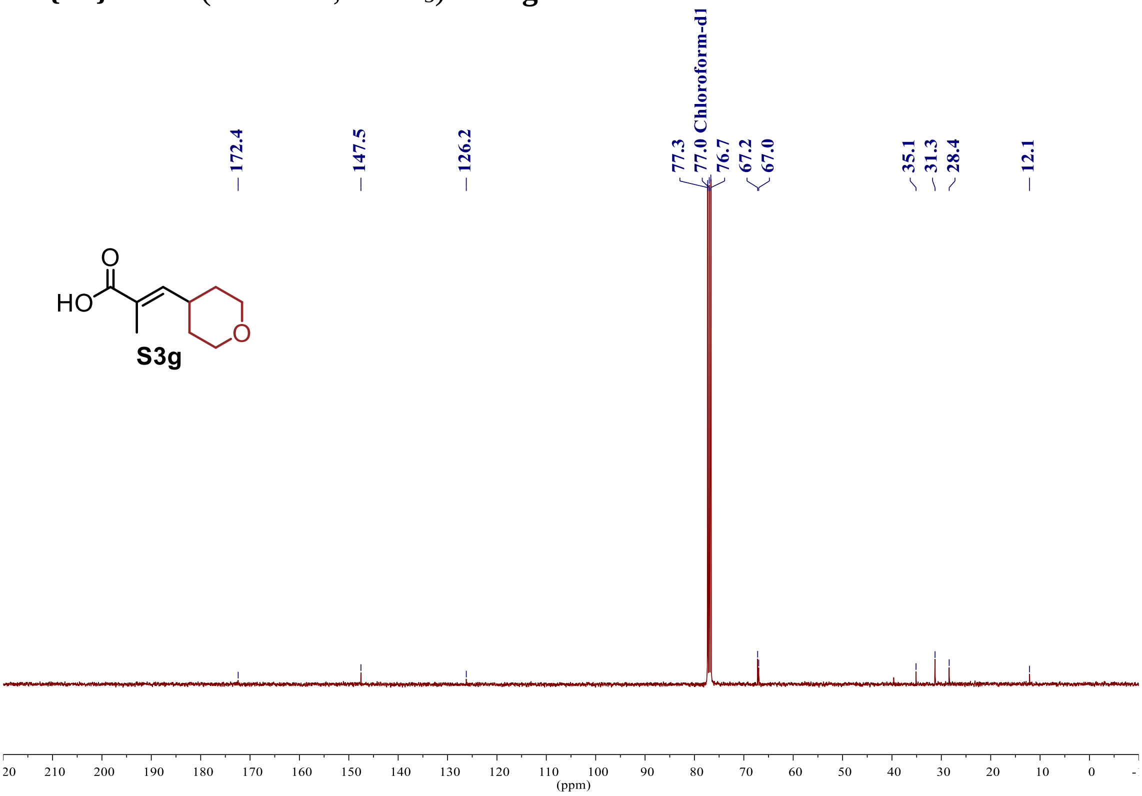

Back to contents

^{1}H NMR (300 MHz, $CDCl_3$) of **S3h**, See procedure

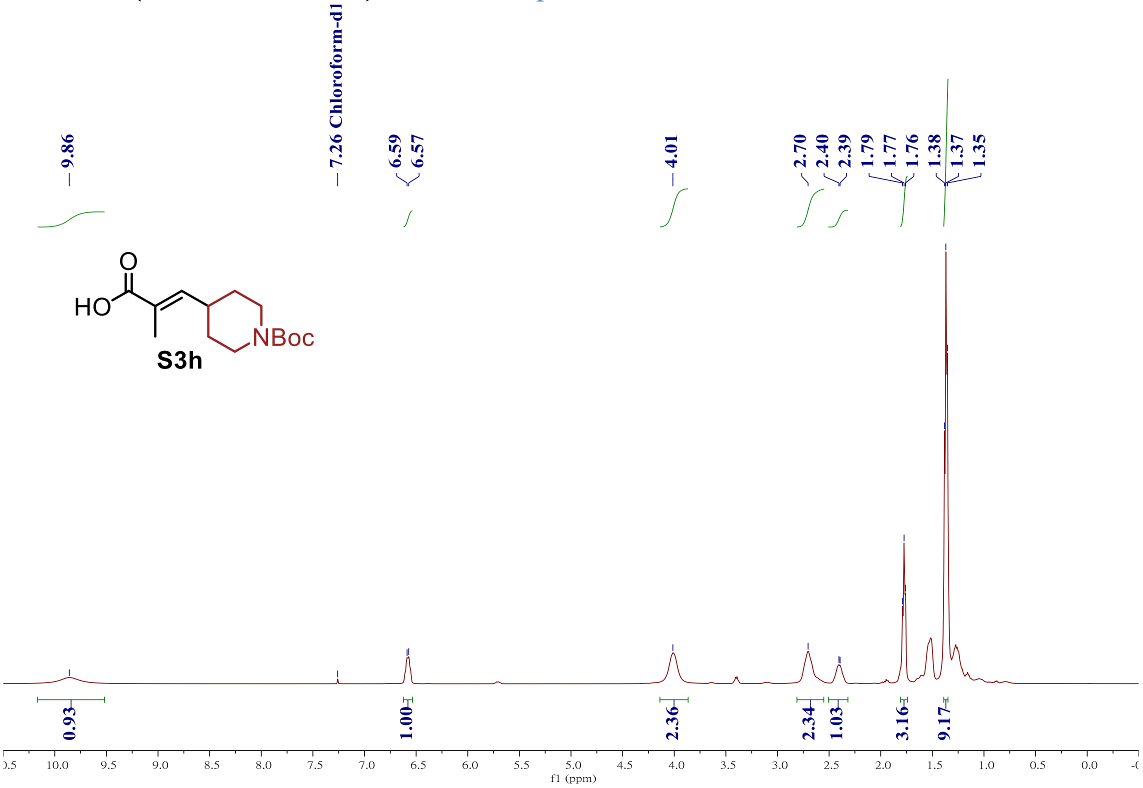

^{13}C{^{1}H} NMR (101 MHz, $CDCl_3$) of **S3h**

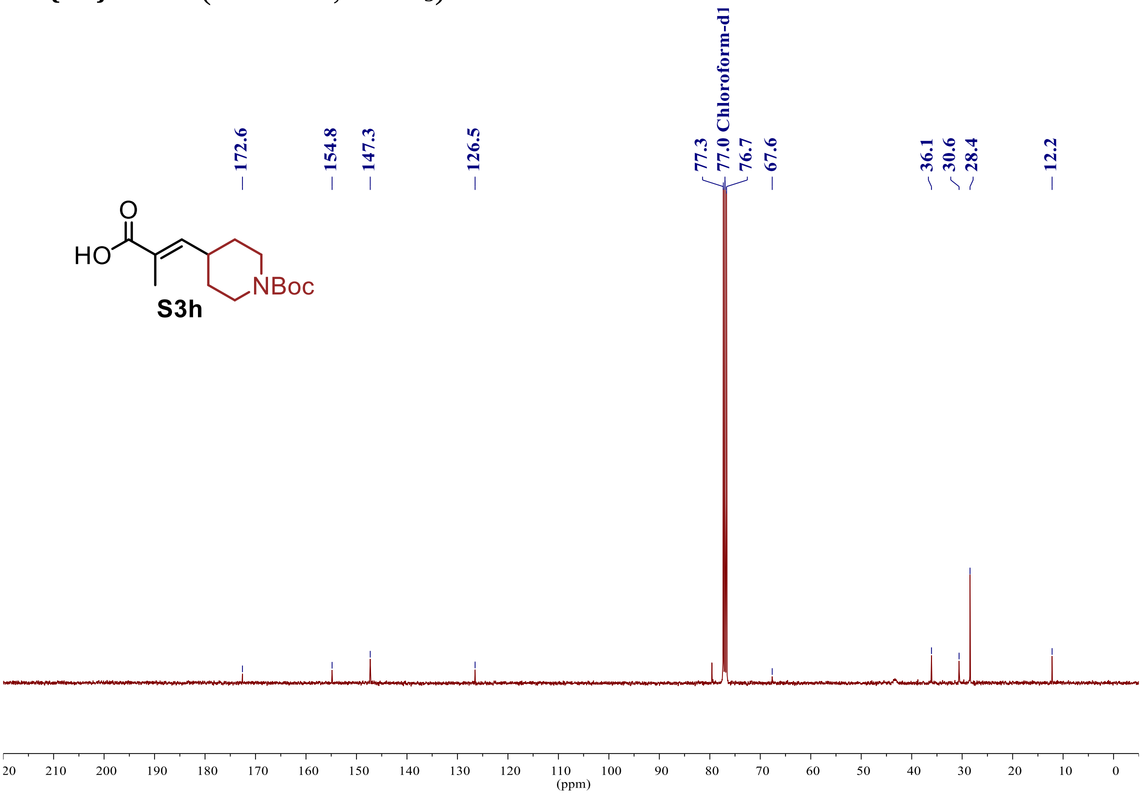

Back to contents

^{1}H NMR (400 MHz, $CDCl_3$) of **S3l**, See procedure

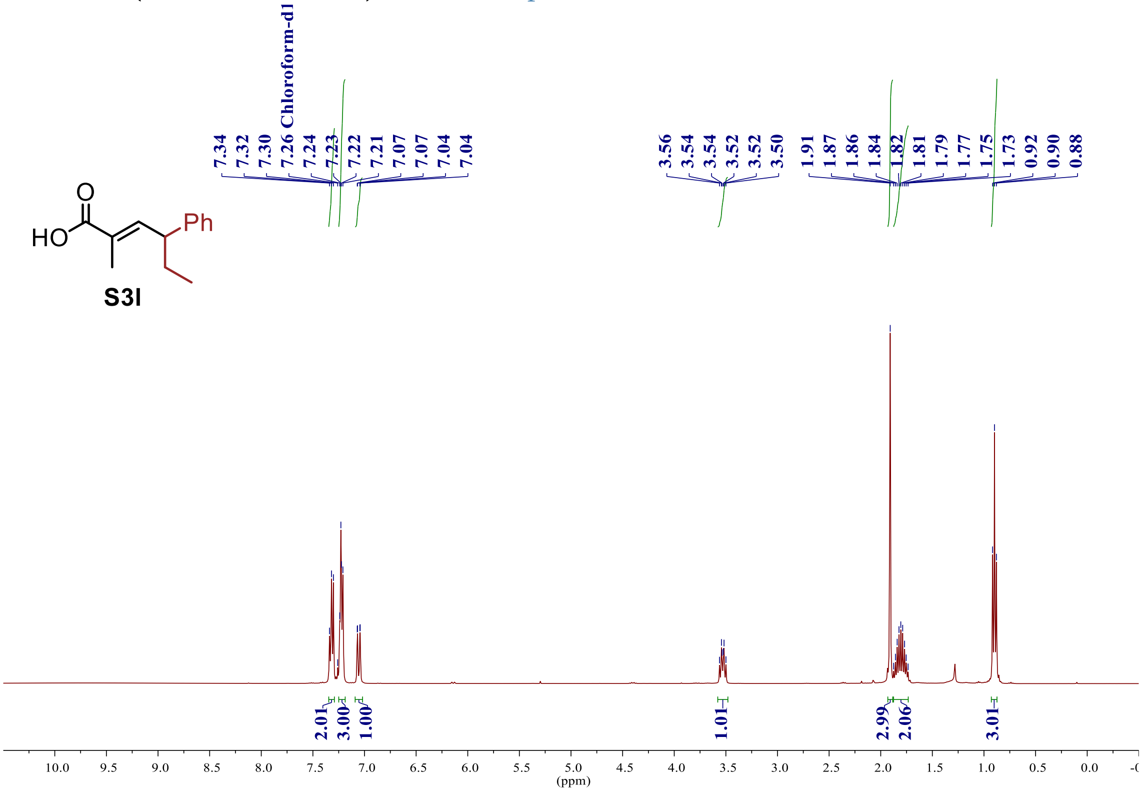

S3l

^{13}C{^{1}H} NMR (101 MHz, $CDCl_3$) of **S3l**

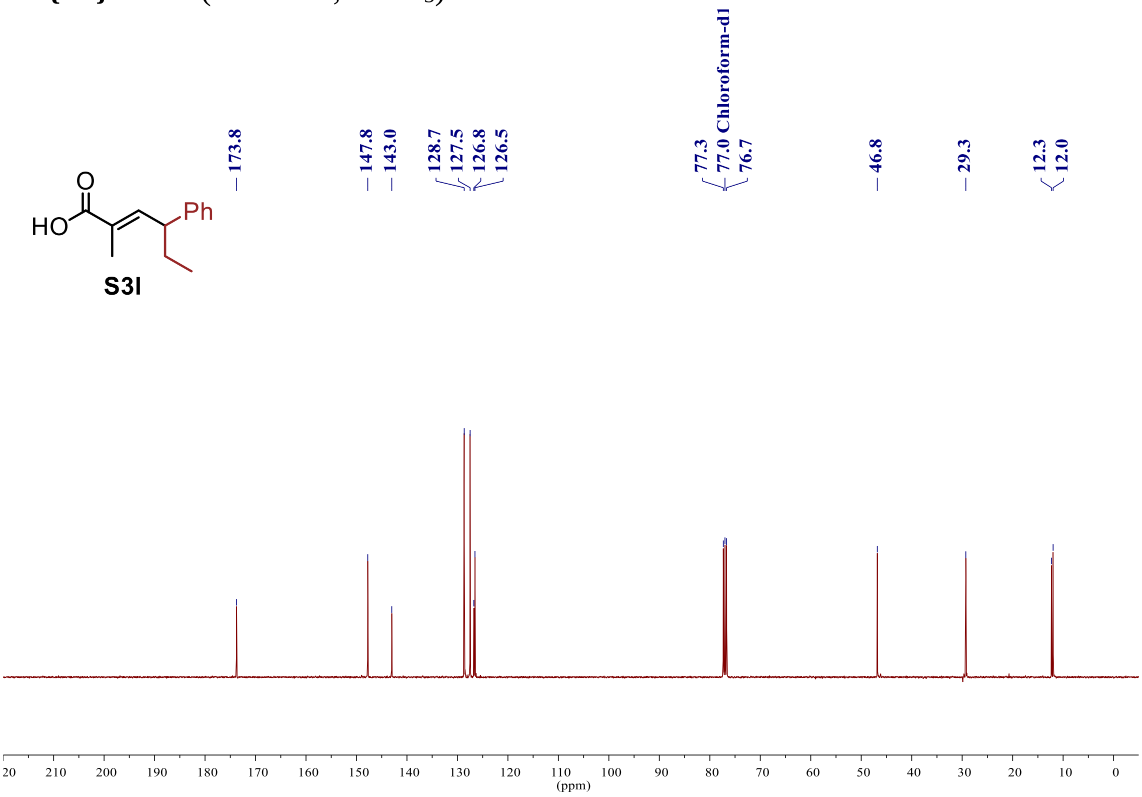

S3l

[Back to contents](#)

^{1}H NMR (300 MHz, $CDCl_3$) of **S3m**, See procedure

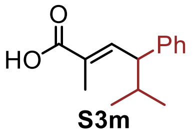

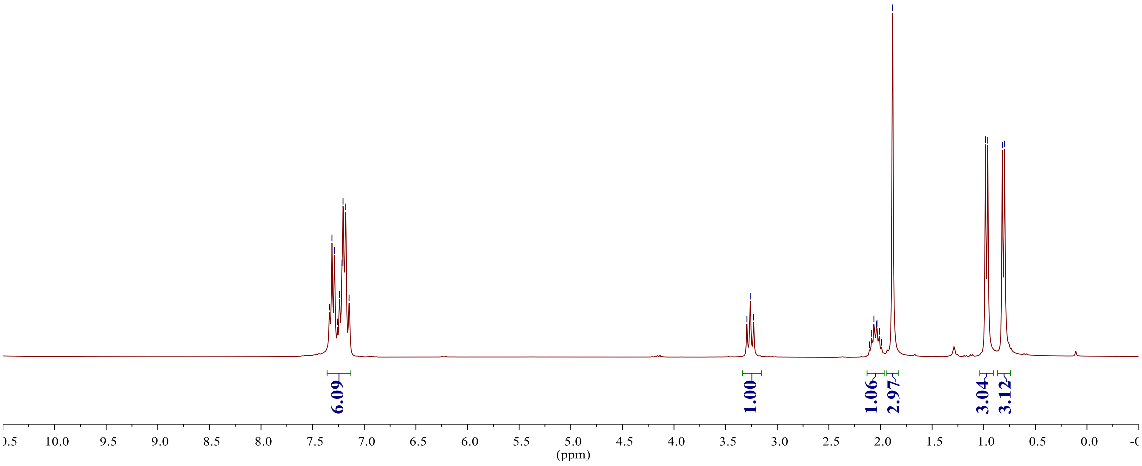

^{13}C{^{1}H} NMR (101 MHz, $CDCl_3$) of **S3m**

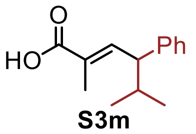

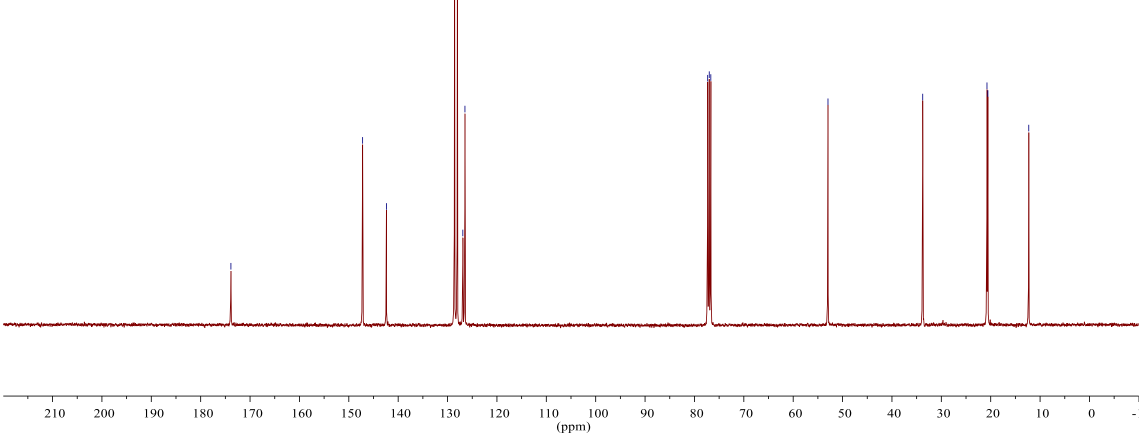

Back to contents

^{1}H NMR (600 MHz, $CDCl_3$) of **S3n**, See procedure

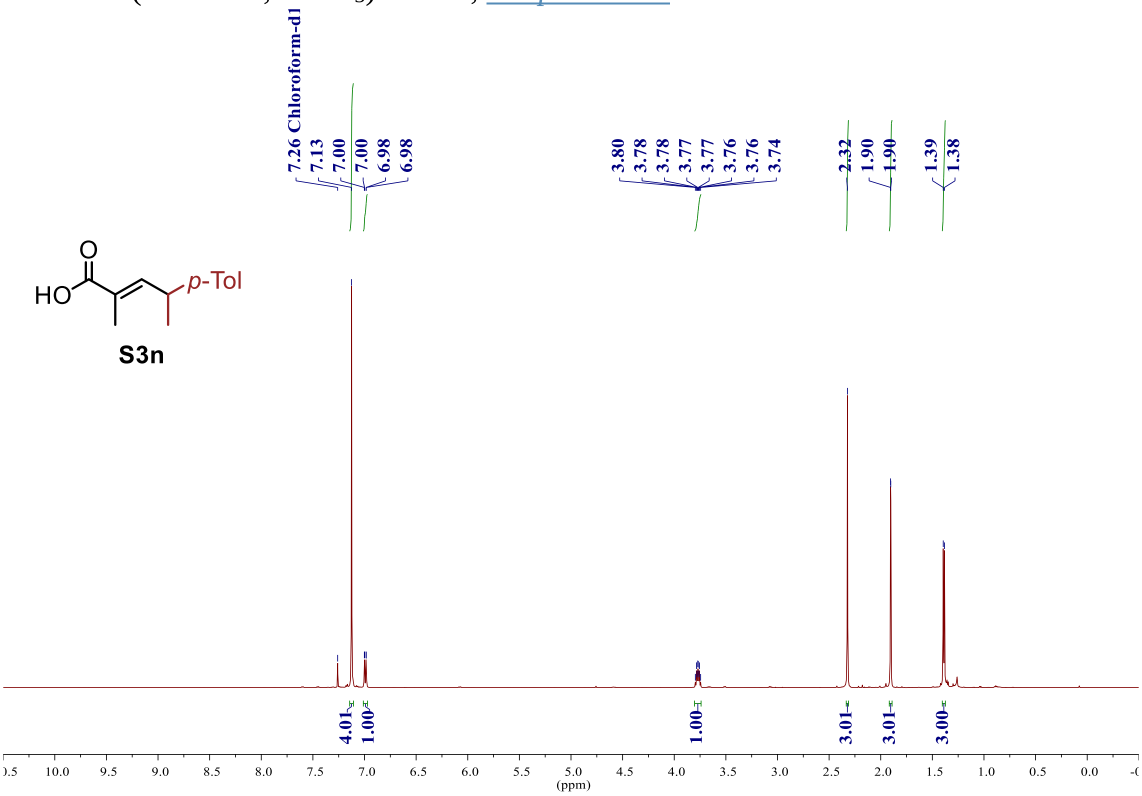

$^{13}C\{^1H\}$ NMR (151 MHz, $CDCl_3$) of **S3n**

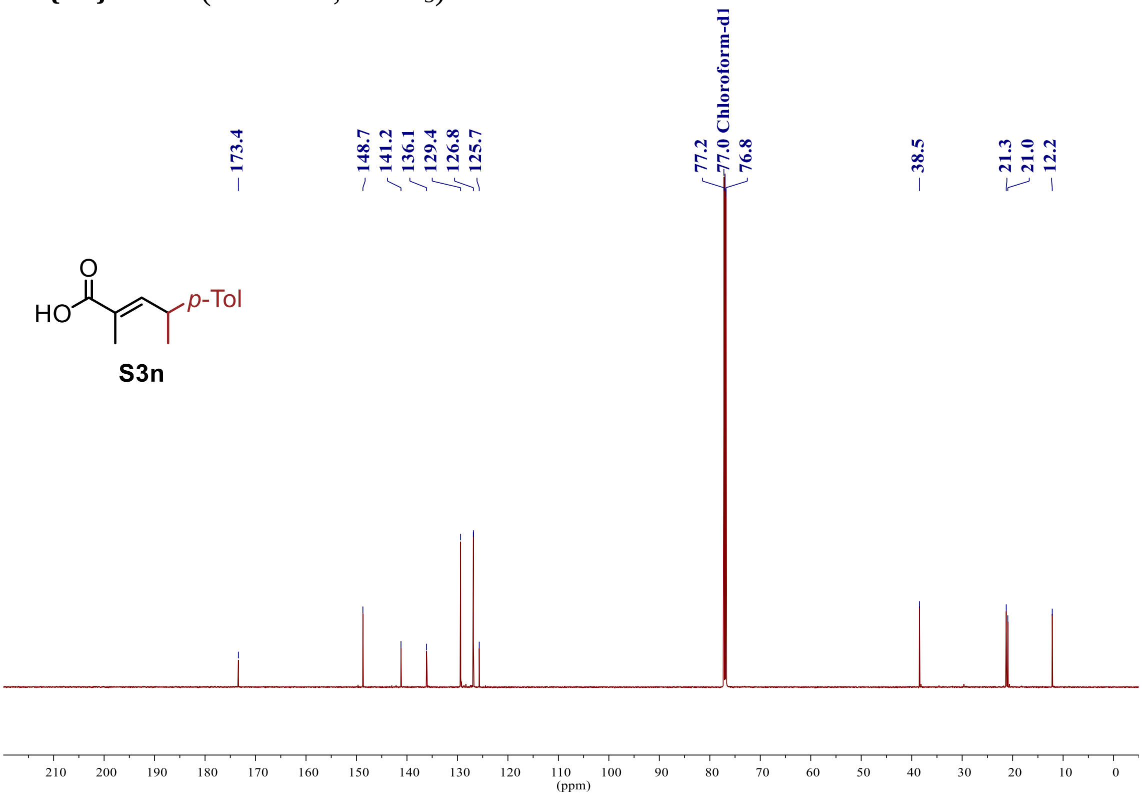

Back to contents

^{1}H NMR (600 MHz, $CDCl_3$) of **S3o**, See procedure

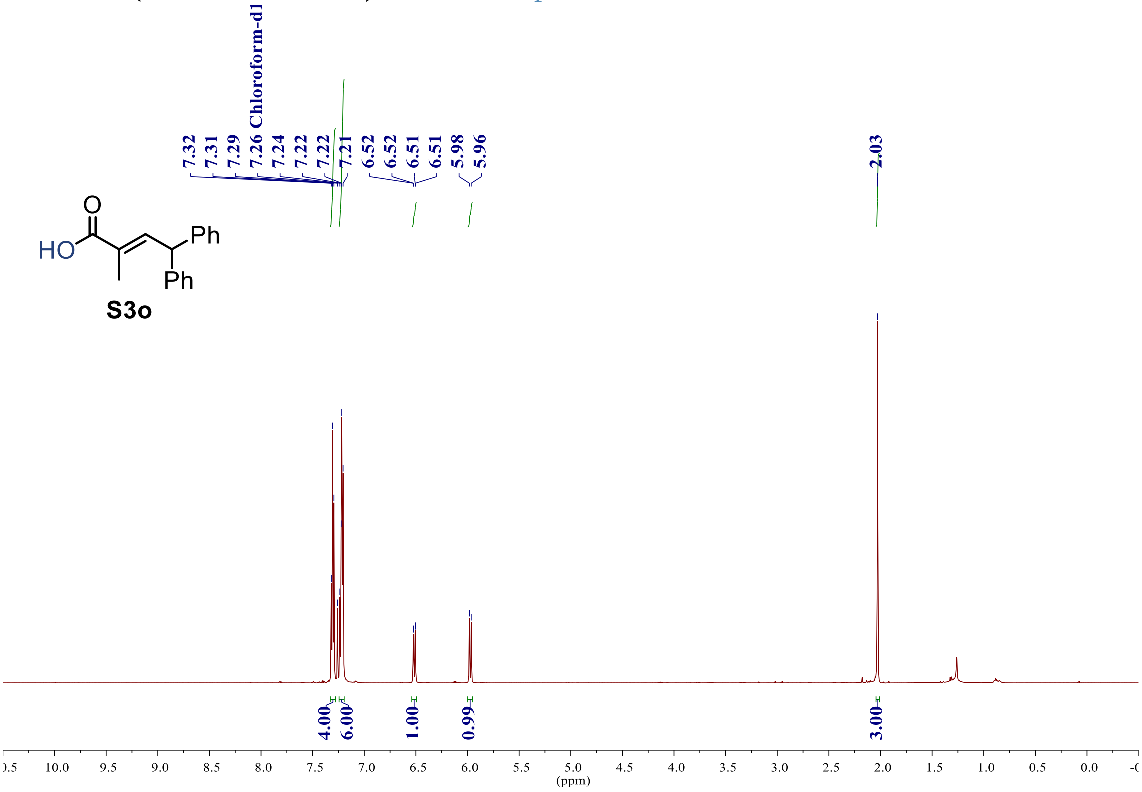

^{13}C{^{1}H} NMR (151 MHz, $CDCl_3$) of **S3o**

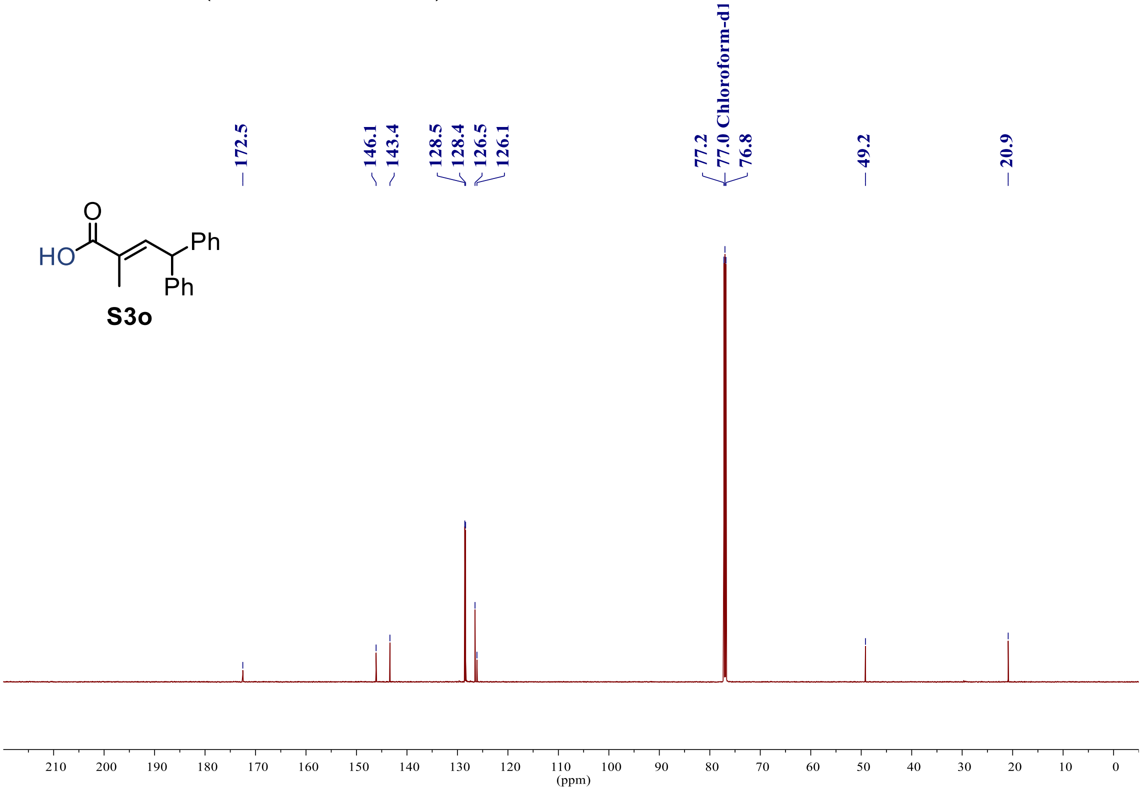

Back to contents

^{1}H NMR (300 MHz, $CDCl_3$) of **S3p**, See procedure

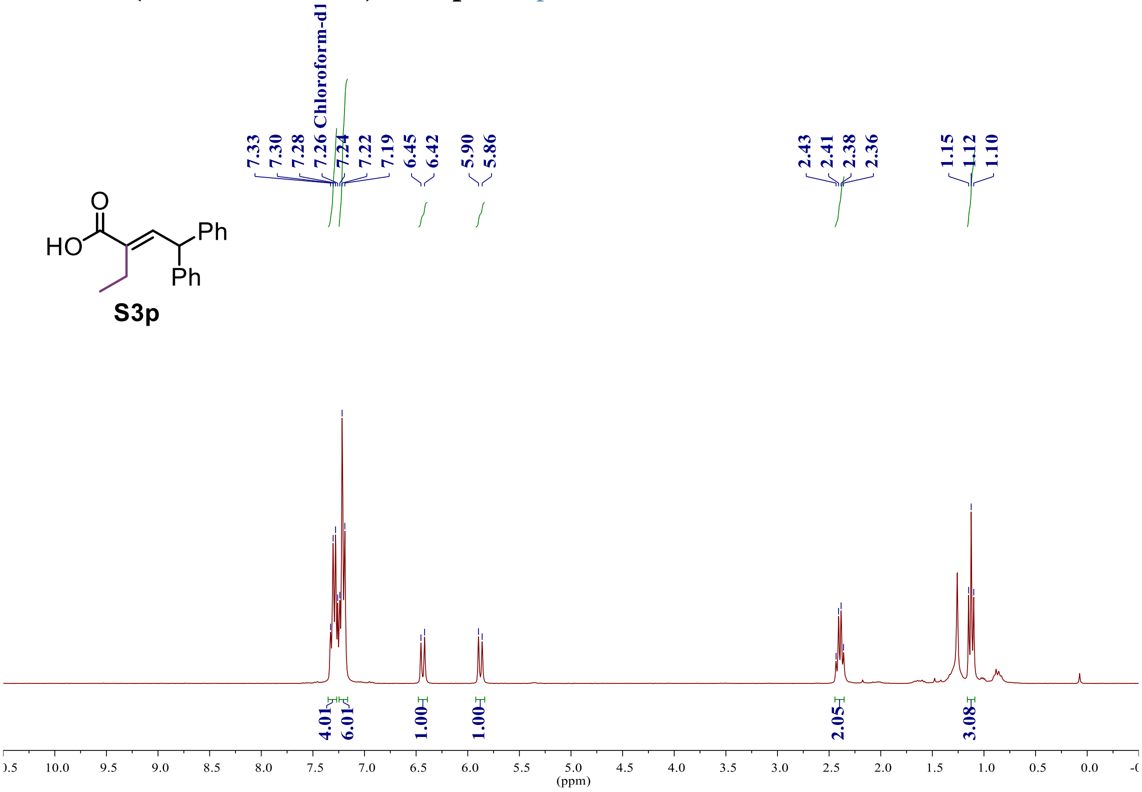

^{13}C{^{1}H} NMR (101 MHz, $CDCl_3$) of **S3p**

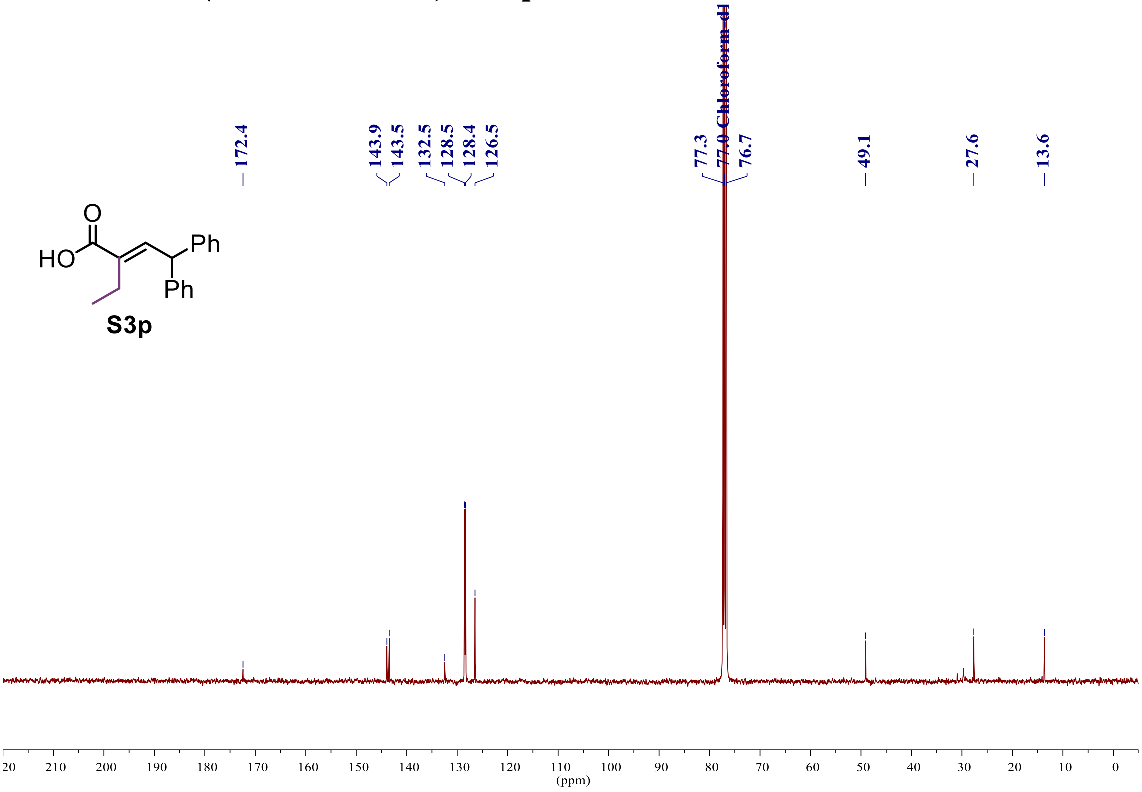

Back to contents

^{1}H NMR (400 MHz, $CDCl_3$) of **S3q**, See procedure

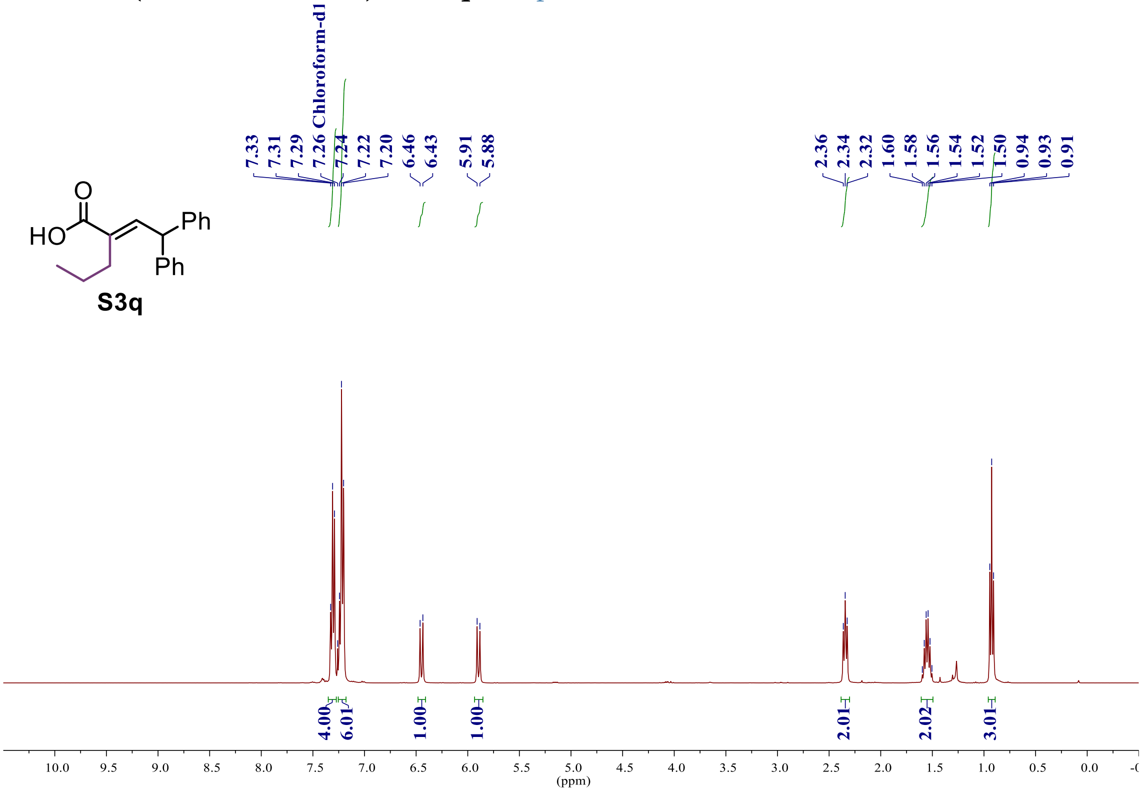

^{13}C{^{1}H} NMR (101 MHz, $CDCl_3$) of **S3q**

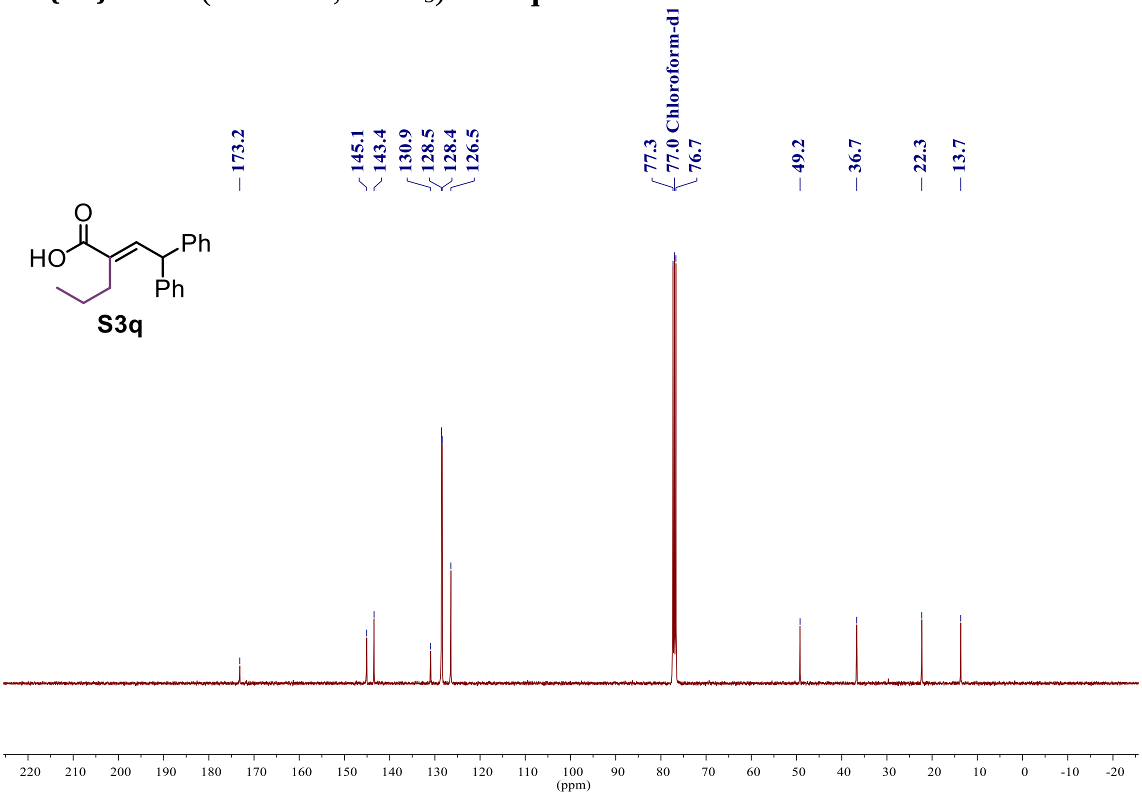

Back to contents

^{1}H NMR (400 MHz, $CDCl_3$) of **S3r**, See procedure

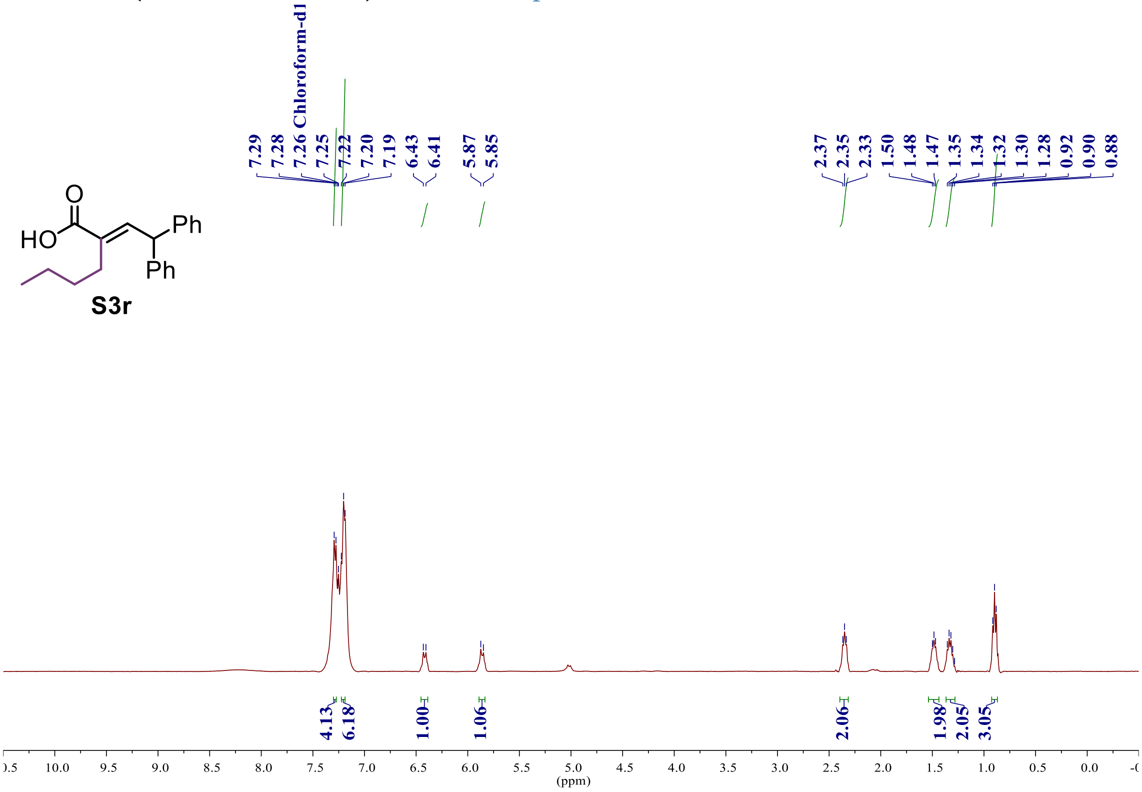

^{13}C{^{1}H} NMR (101 MHz, $CDCl_3$) of **S3r**

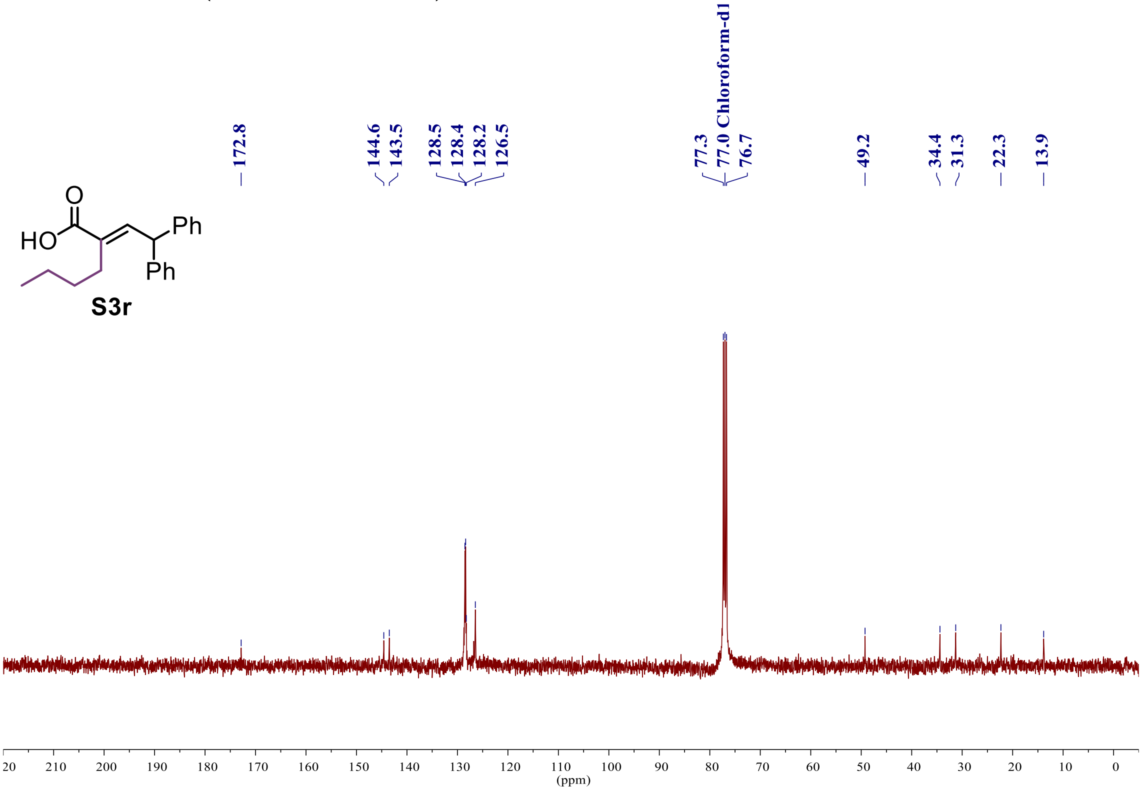

Back to contents

^{1}H NMR (400 MHz, $CDCl_3$) of **S3s**, See procedure

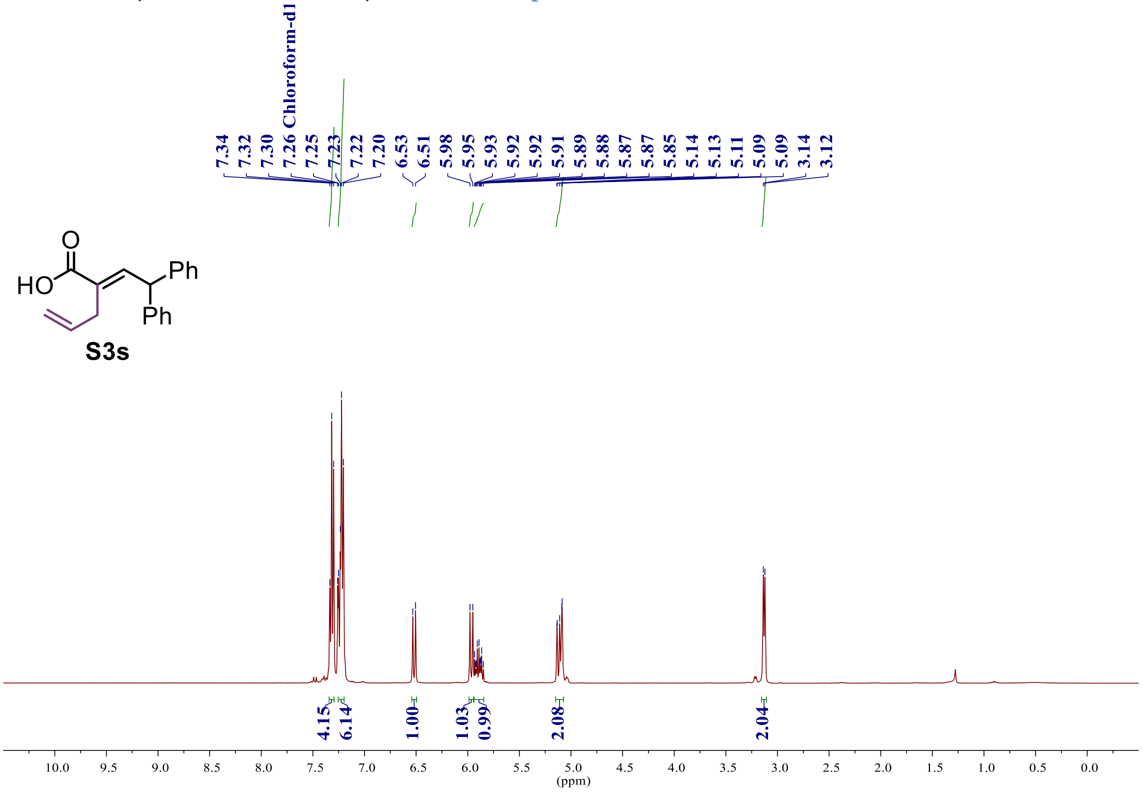

$^{13}C\{^1H\}$ NMR (101 MHz, $CDCl_3$) of **S3s**

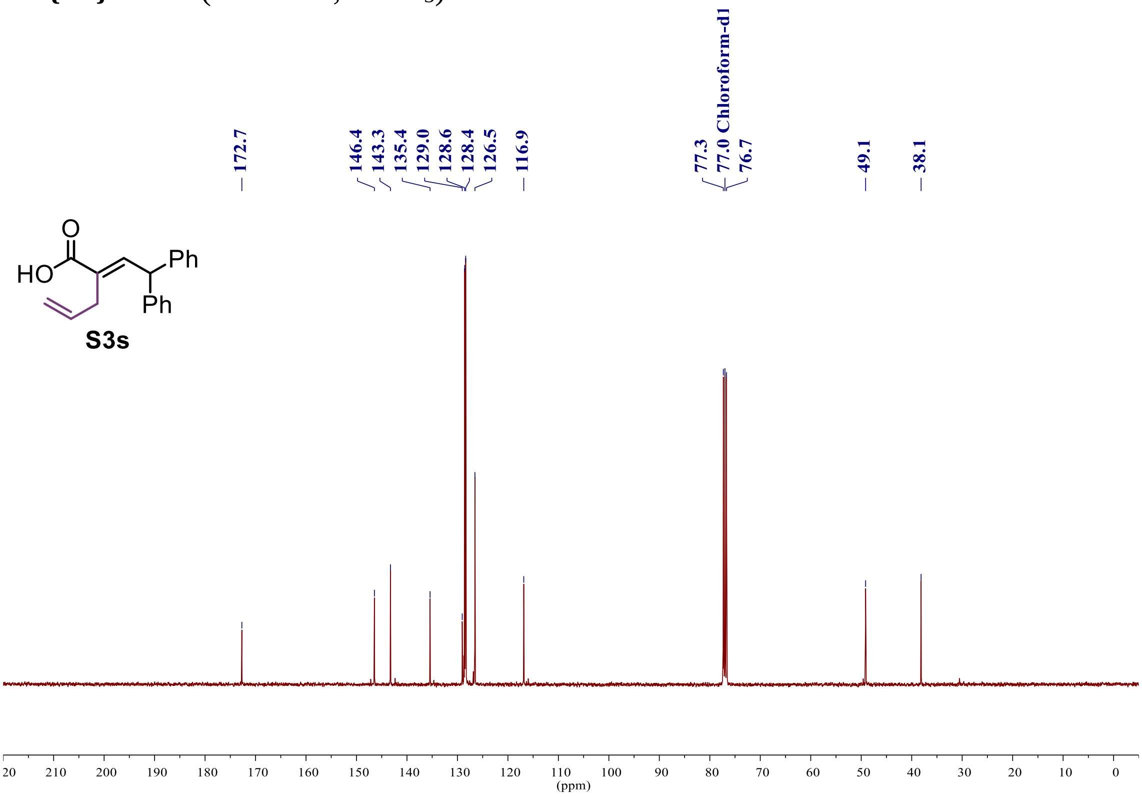

^{1}H NMR (300 MHz, $CDCl_3$) of **S3t**, [See procedure](#)

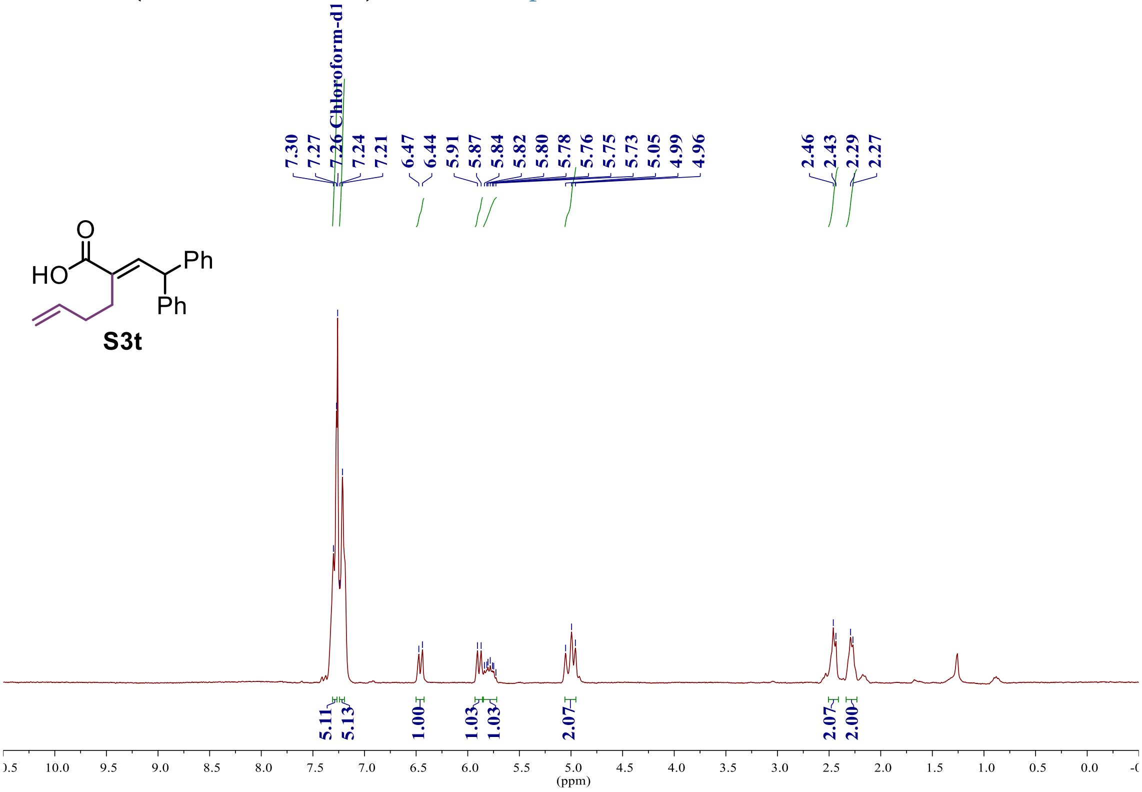

^{13}C{^{1}H} NMR (101 MHz, $CDCl_3$) of **S3t**

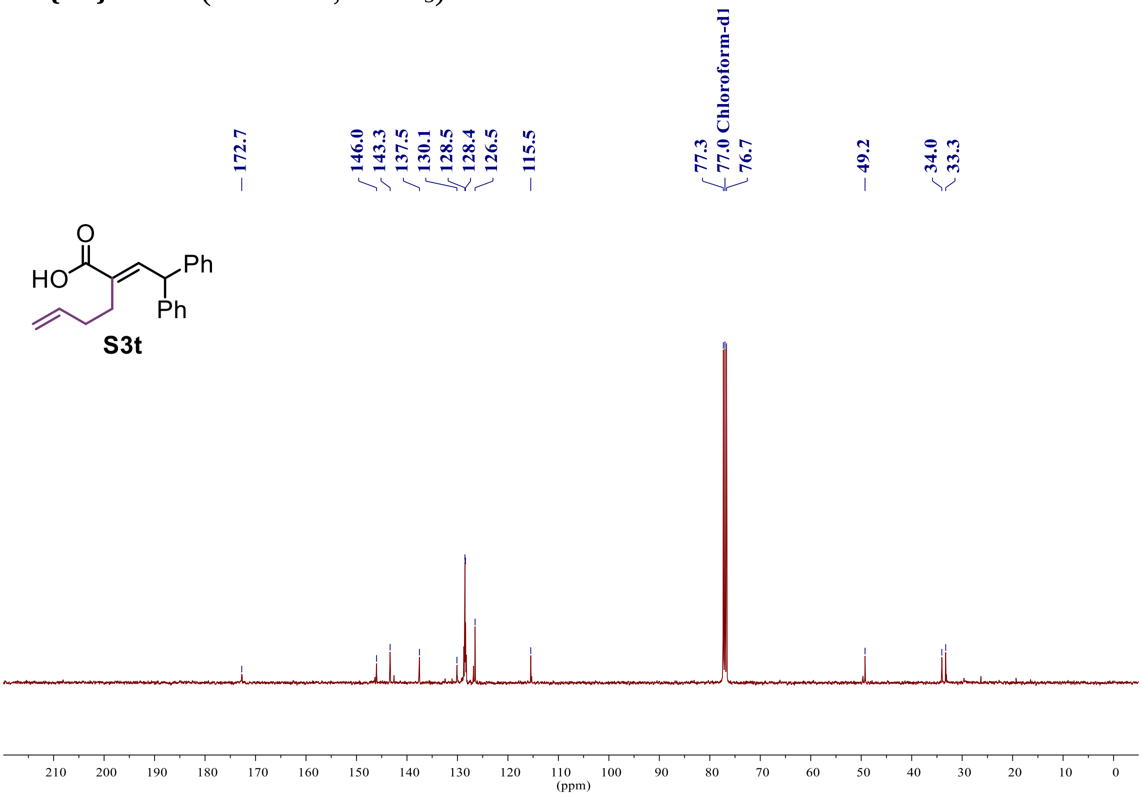

Back to contents

^{1}H NMR (400 MHz, $CDCl_3$) of **1a**, See procedure

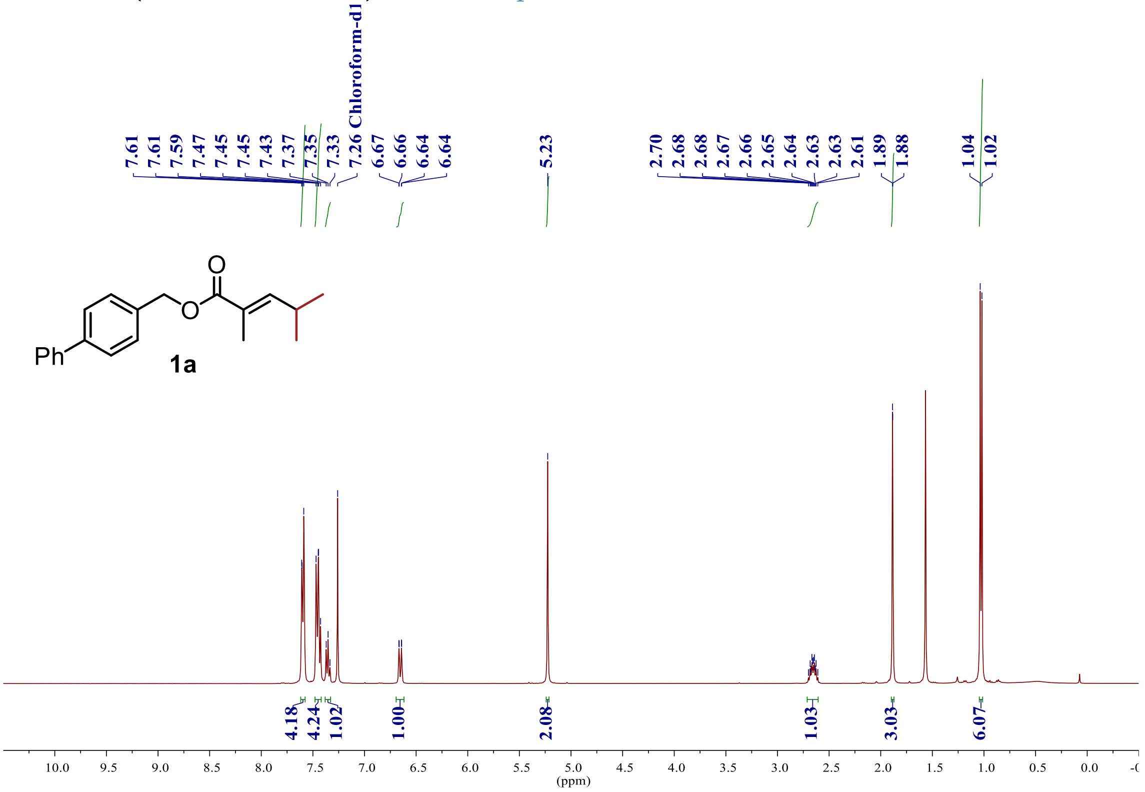

^{13}C{^{1}H} NMR (101 MHz, $CDCl_3$) of **1a**

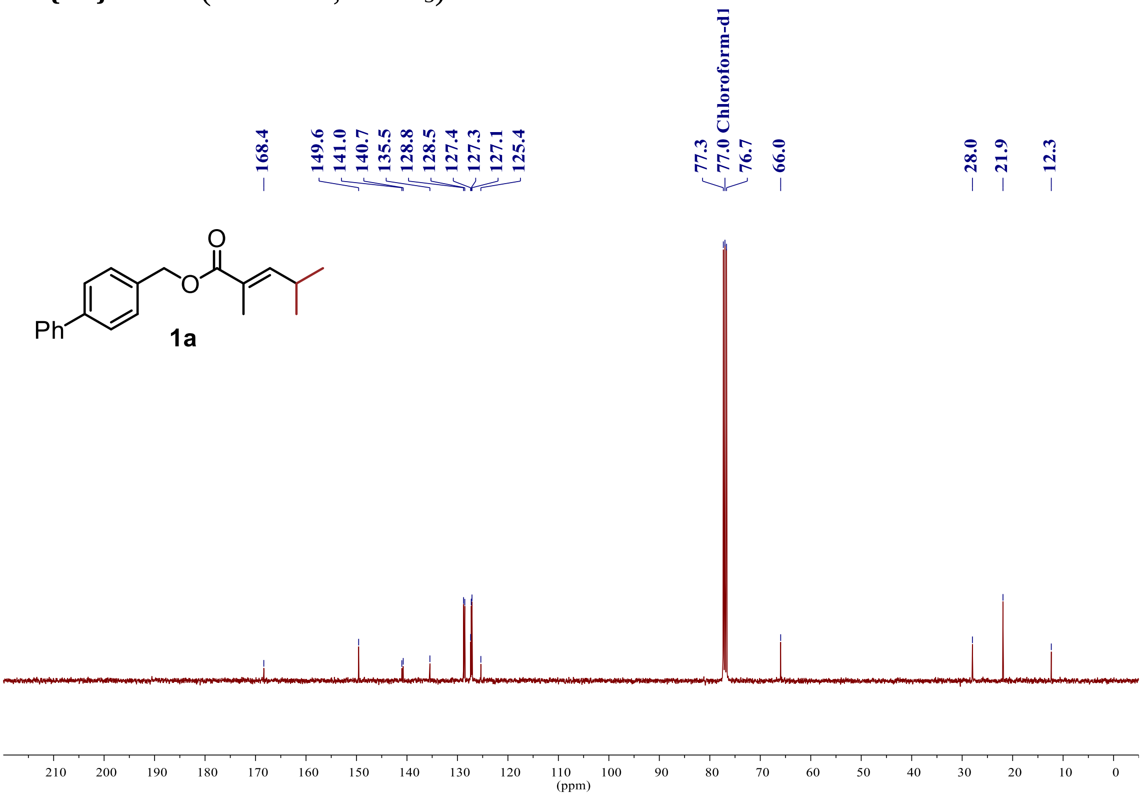

Back to contents

^{1}H NMR (400 MHz, $CDCl_3$) of **1b**, See procedure

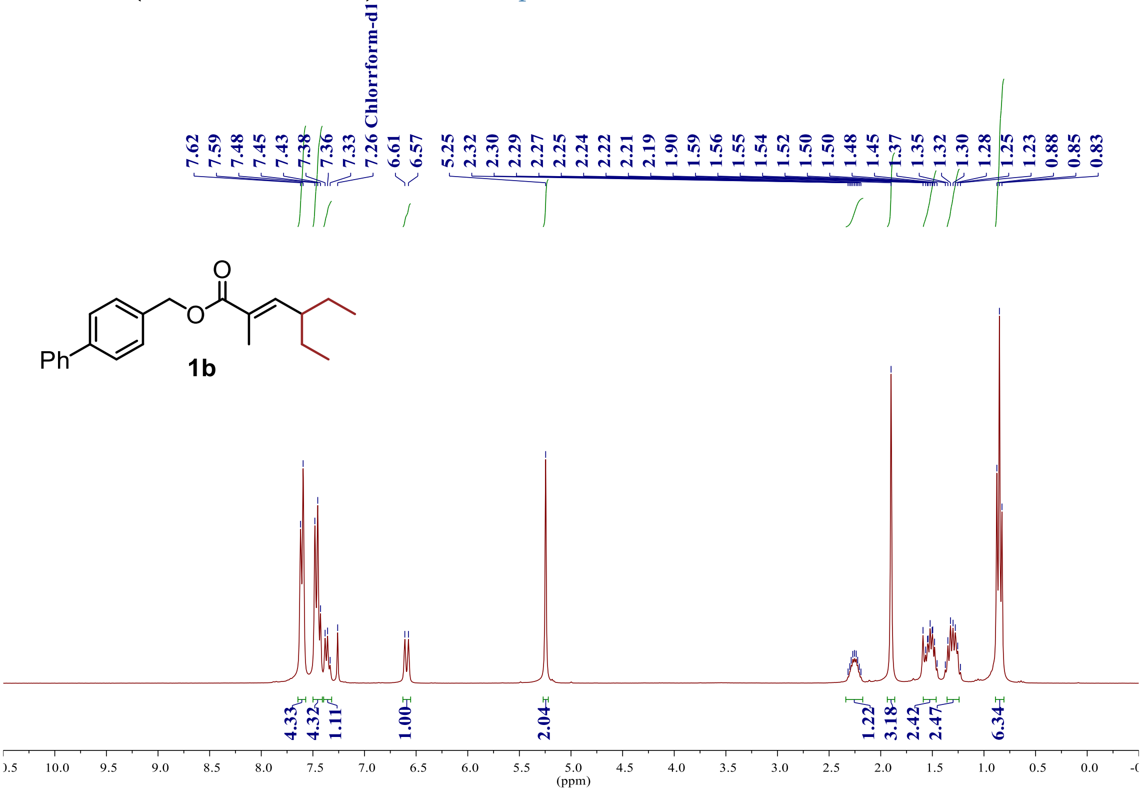

^{13}C{^{1}H} NMR (101 MHz, $CDCl_3$) of **1b**

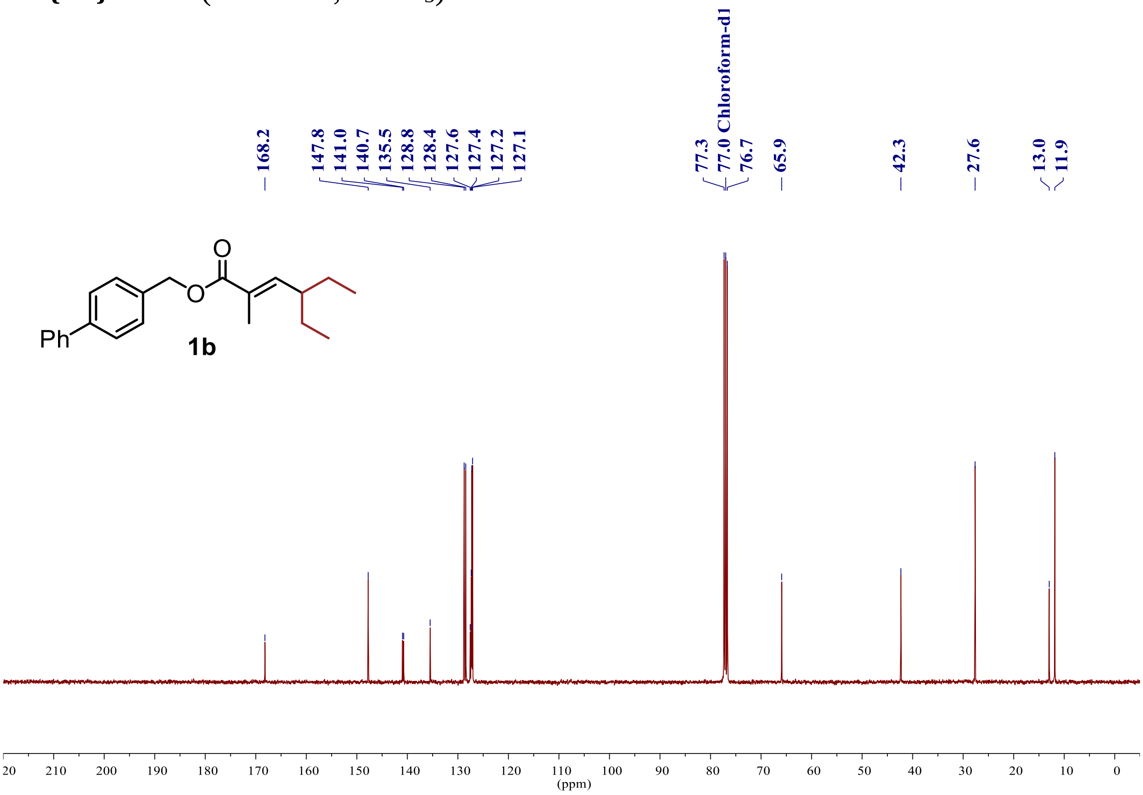

Back to contents

^{1}H NMR (400 MHz, $CDCl_3$) of **1c**, See procedure

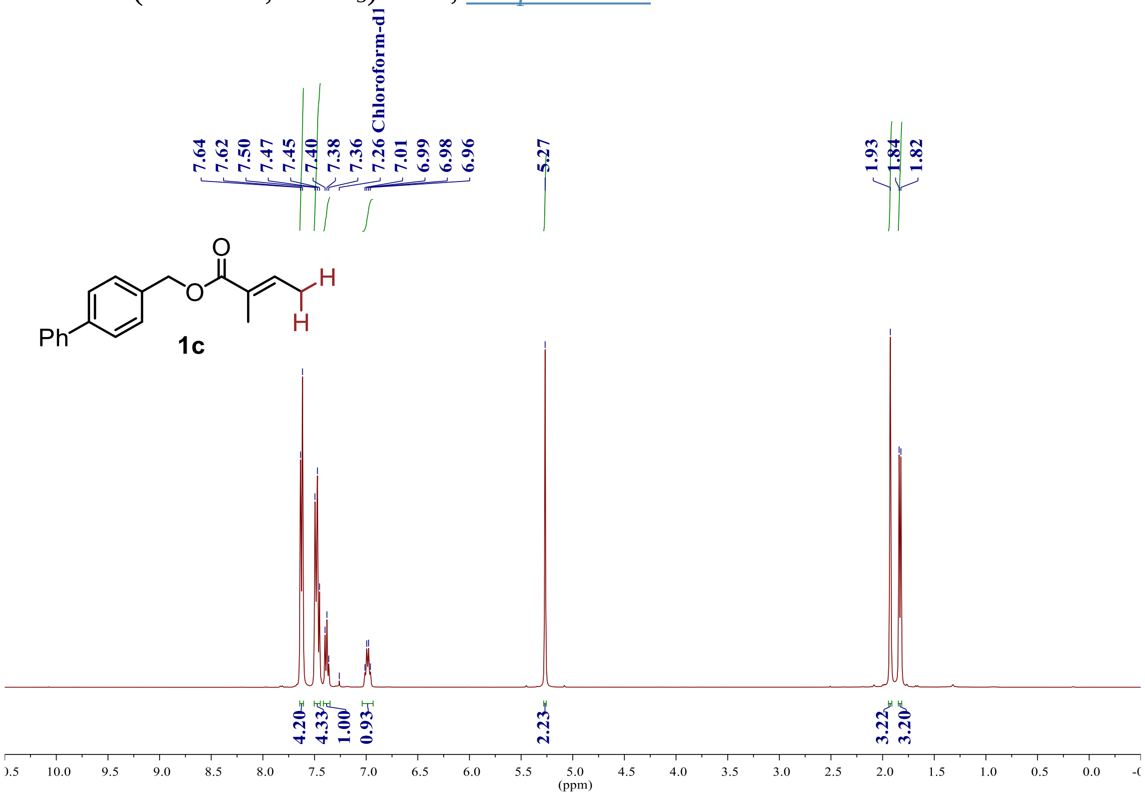

^{13}C{^{1}H} NMR (101 MHz, $CDCl_3$) of **1c**

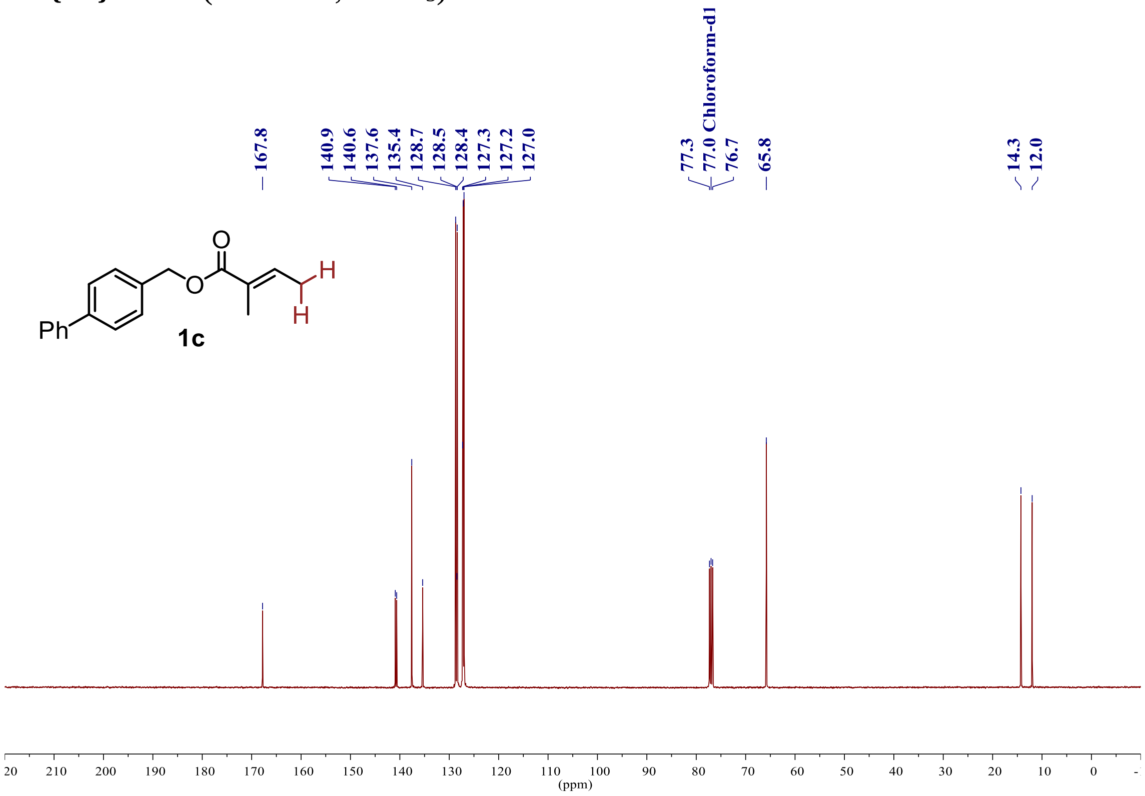

Back to contents

^{1}H NMR (300 MHz, $CDCl_3$) of **1d**, See procedure

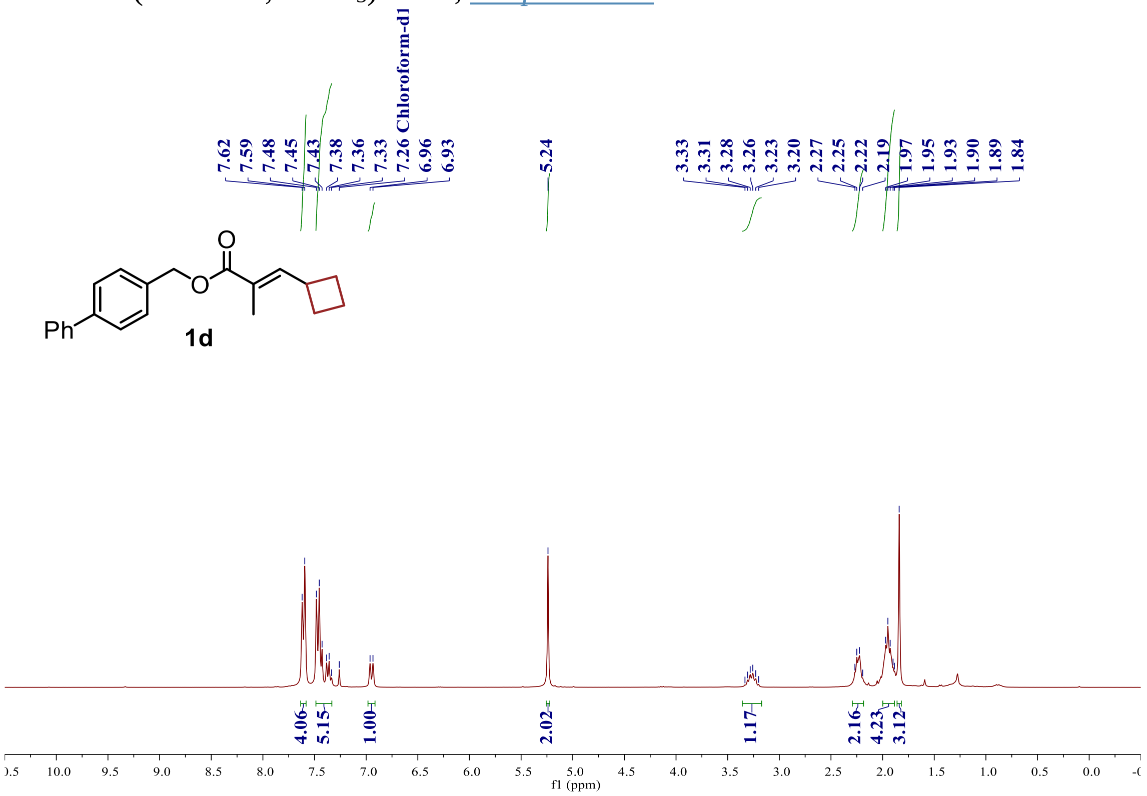

^{13}C{^{1}H} NMR (101 MHz, $CDCl_3$) of **1d**

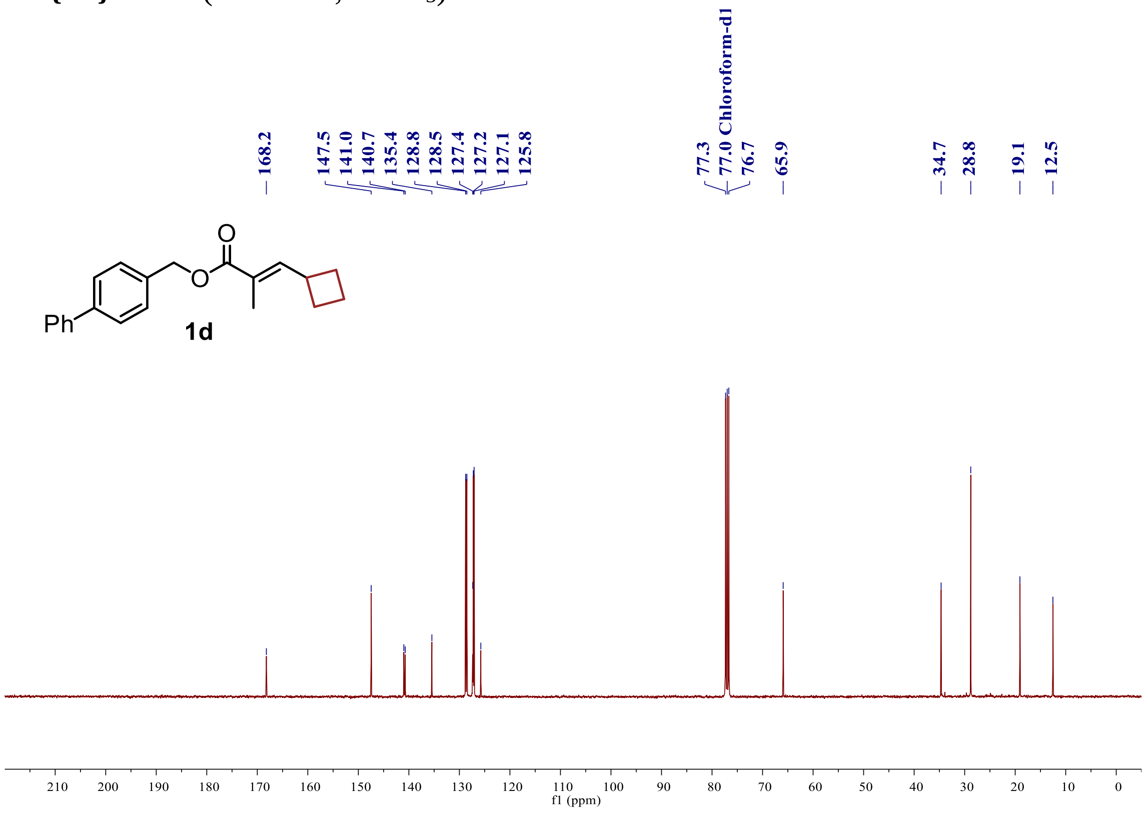

Back to contents

¹H NMR (400 MHz, $CDCl_3$) of **1e**, See procedure

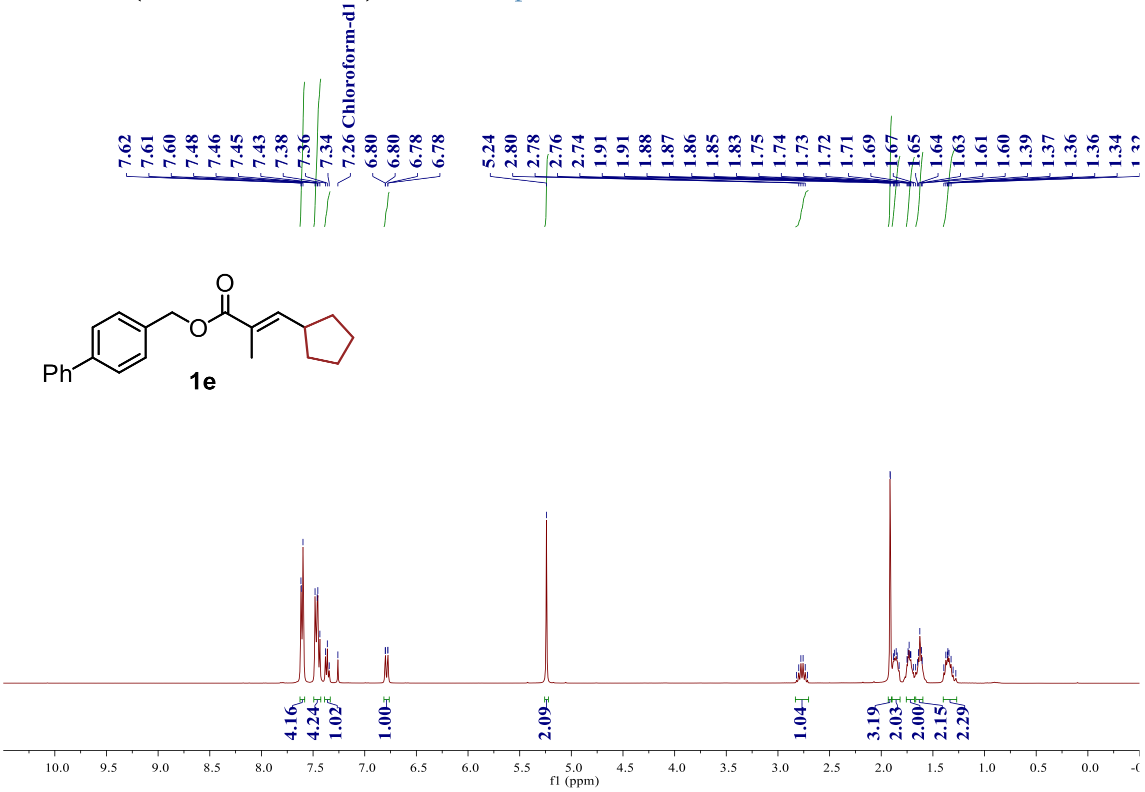

¹³C{¹H} NMR (101 MHz, $CDCl_3$) of **1e**

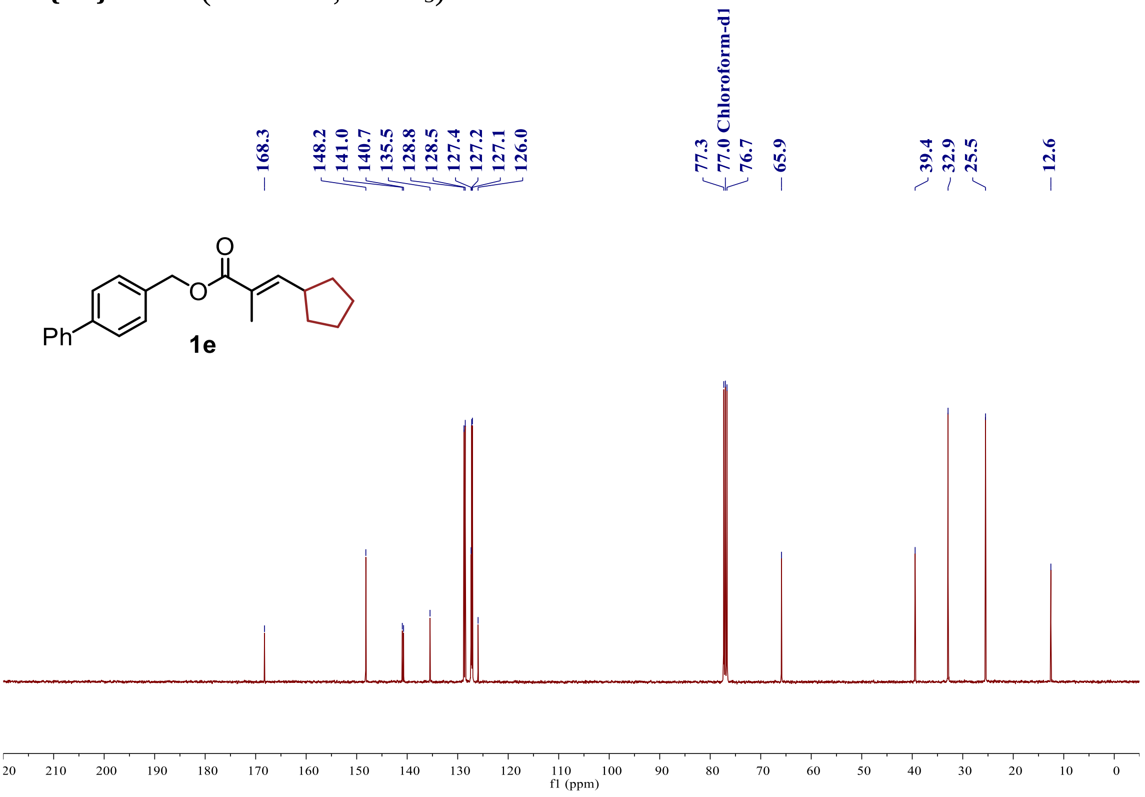

Back to contents

^{1}H NMR (400 MHz, $CDCl_3$) of **1f**, See procedure

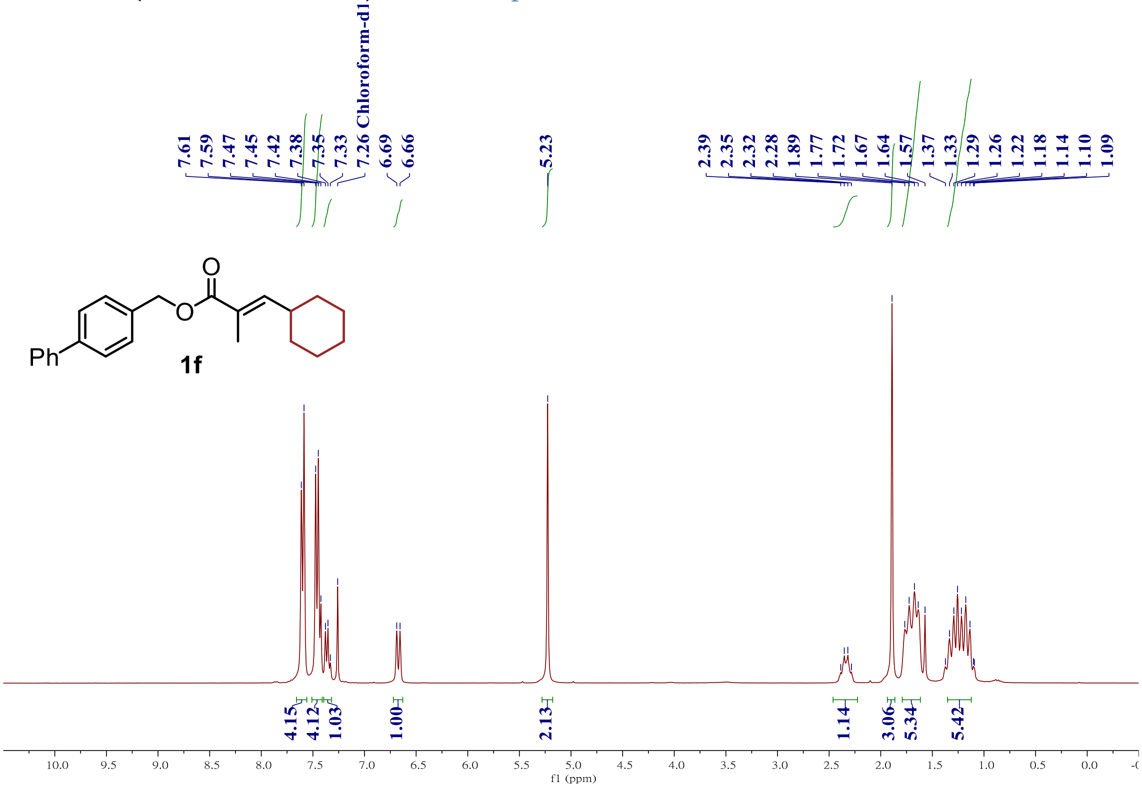

$^{13}C\{^{1}H\}$ NMR (101 MHz, $CDCl_3$) of **1f**

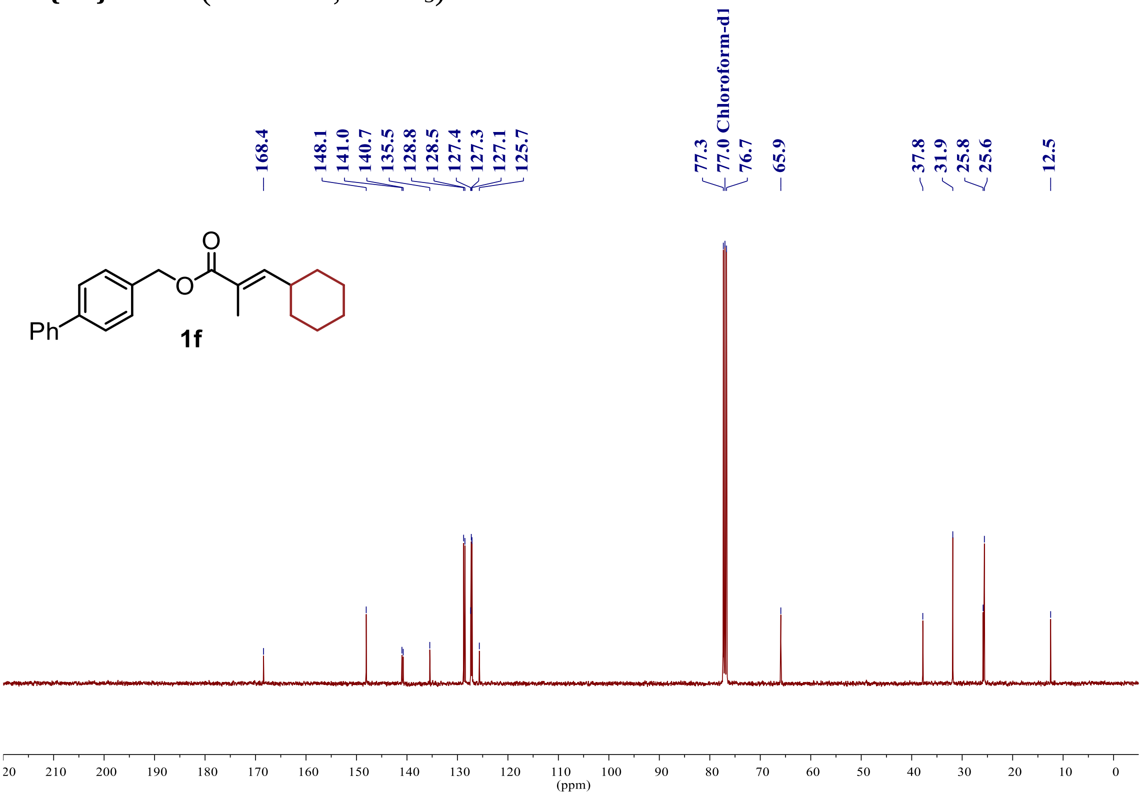

Back to contents

¹H NMR (600 MHz, $CDCl_3$) of **1g**, See procedure

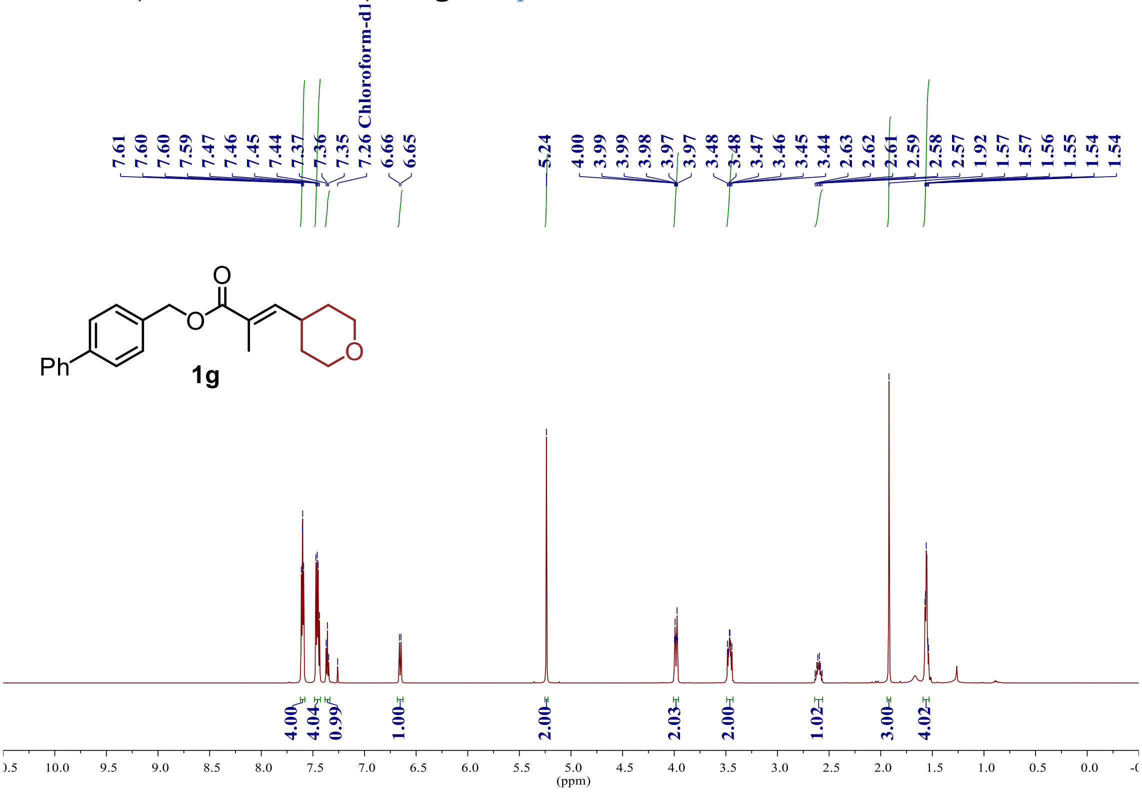

¹³C{¹H} NMR (151 MHz, $CDCl_3$) of **1g**

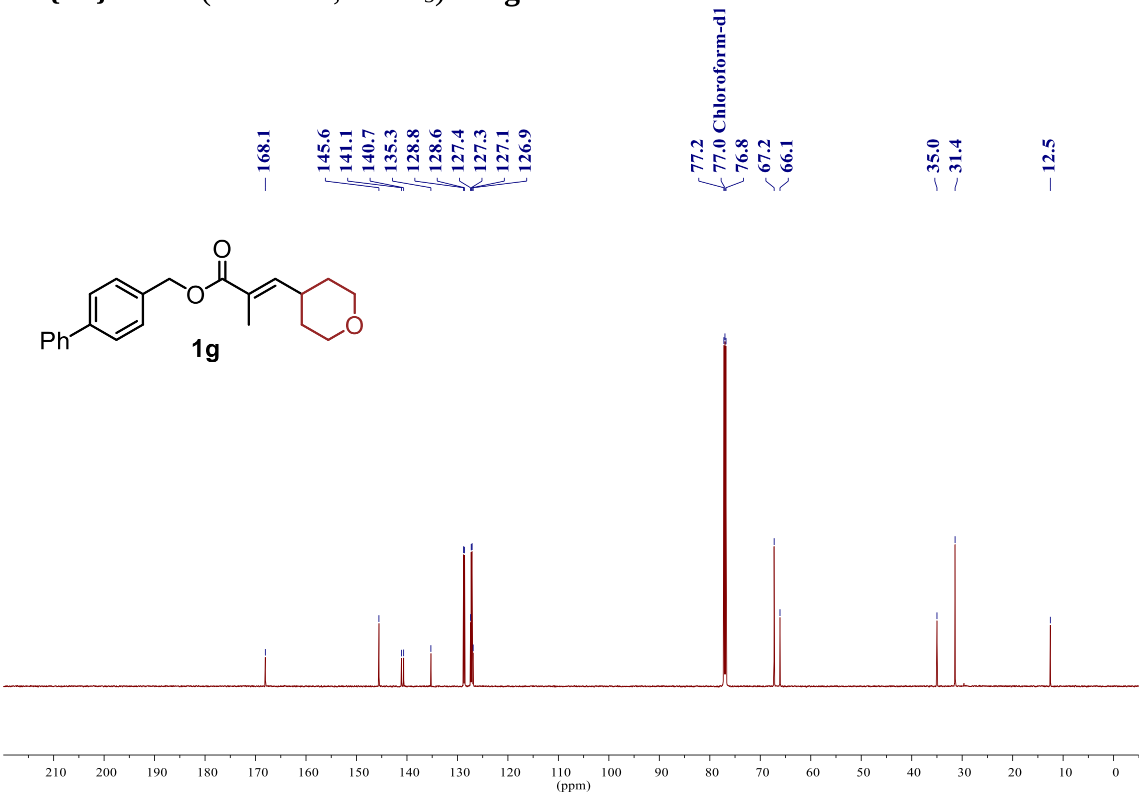

[Back to contents](#)

^{1}H NMR (600 MHz, $CDCl_3$) of **1h**, See procedure

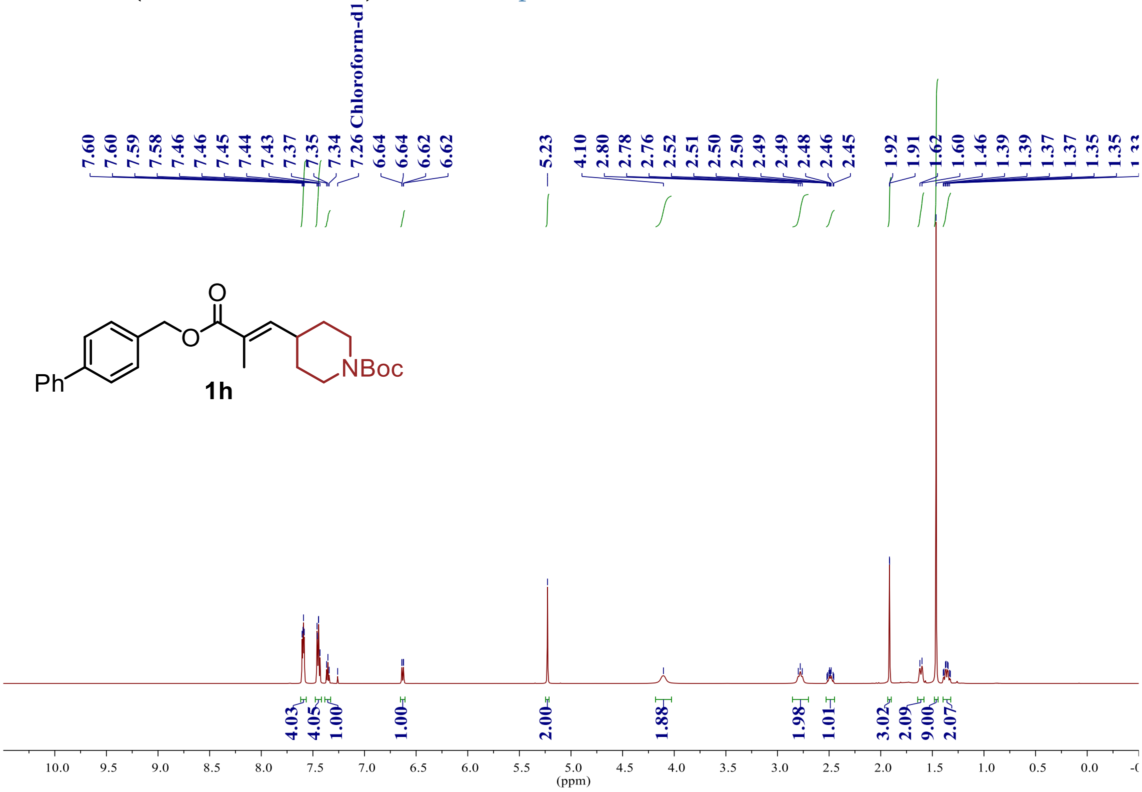

^{13}C{^{1}H} NMR (151 MHz, $CDCl_3$) of **1h**

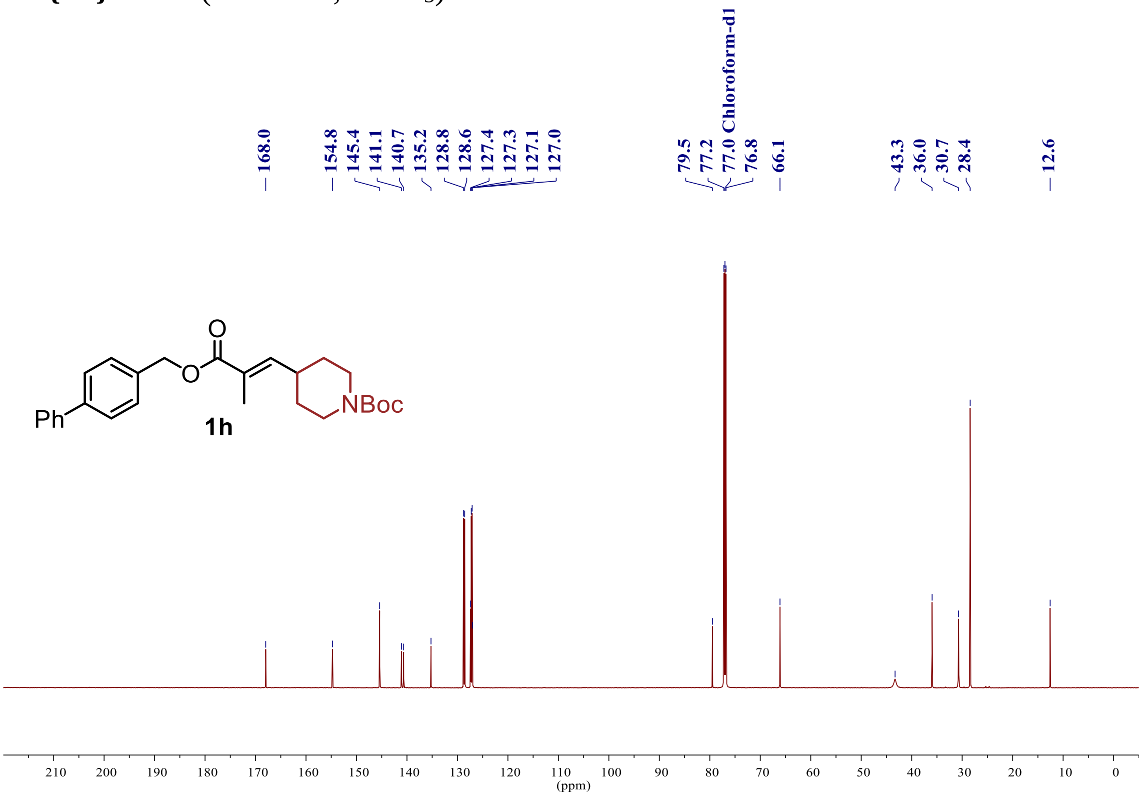

[Back to contents](#)

^{1}H NMR (400 MHz, $CDCl_3$) of **1i**, See procedure

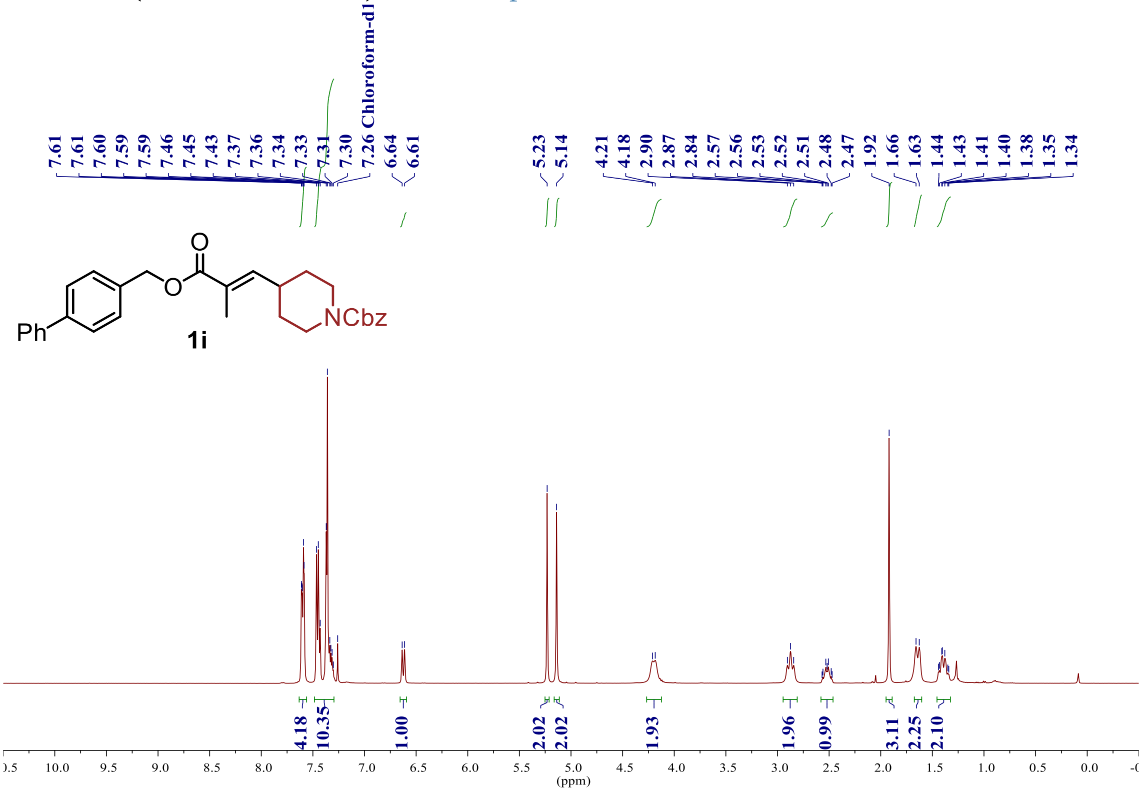

^{13}C{^{1}H} NMR (101 MHz, $CDCl_3$) of **1i**

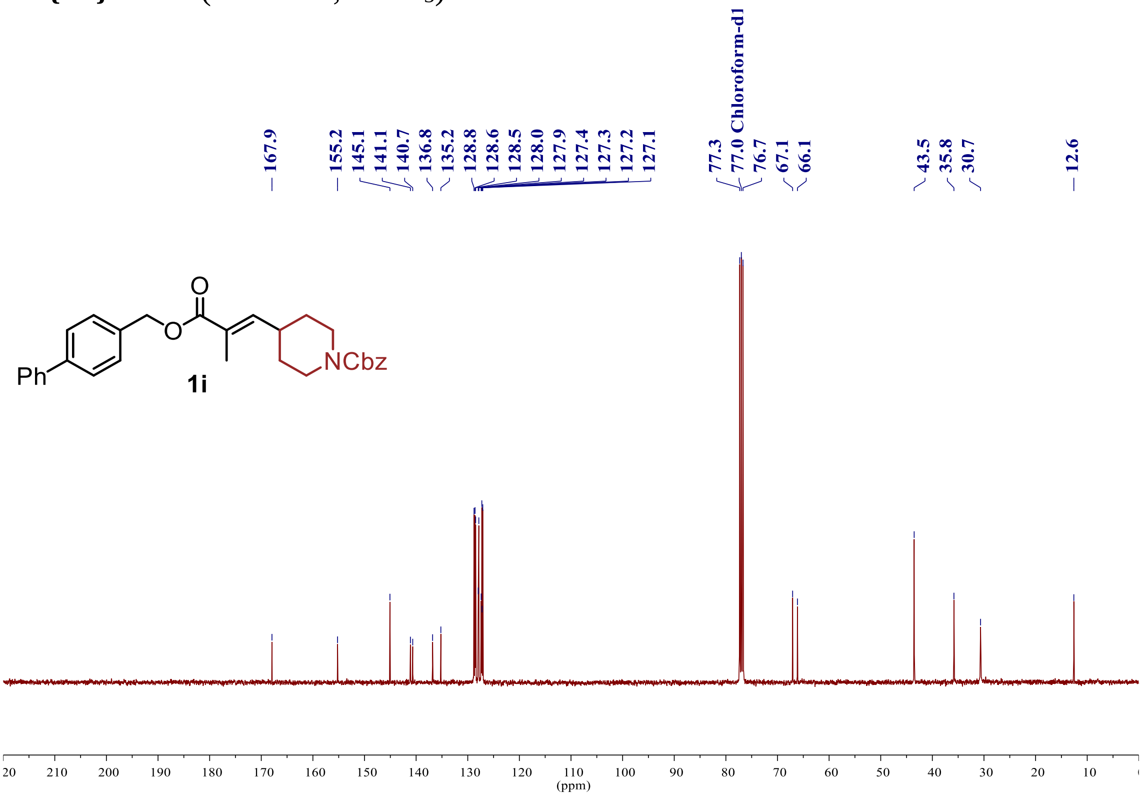

Back to contents

^{1}H NMR (600 MHz, $CDCl_3$) of **1k**, See procedure

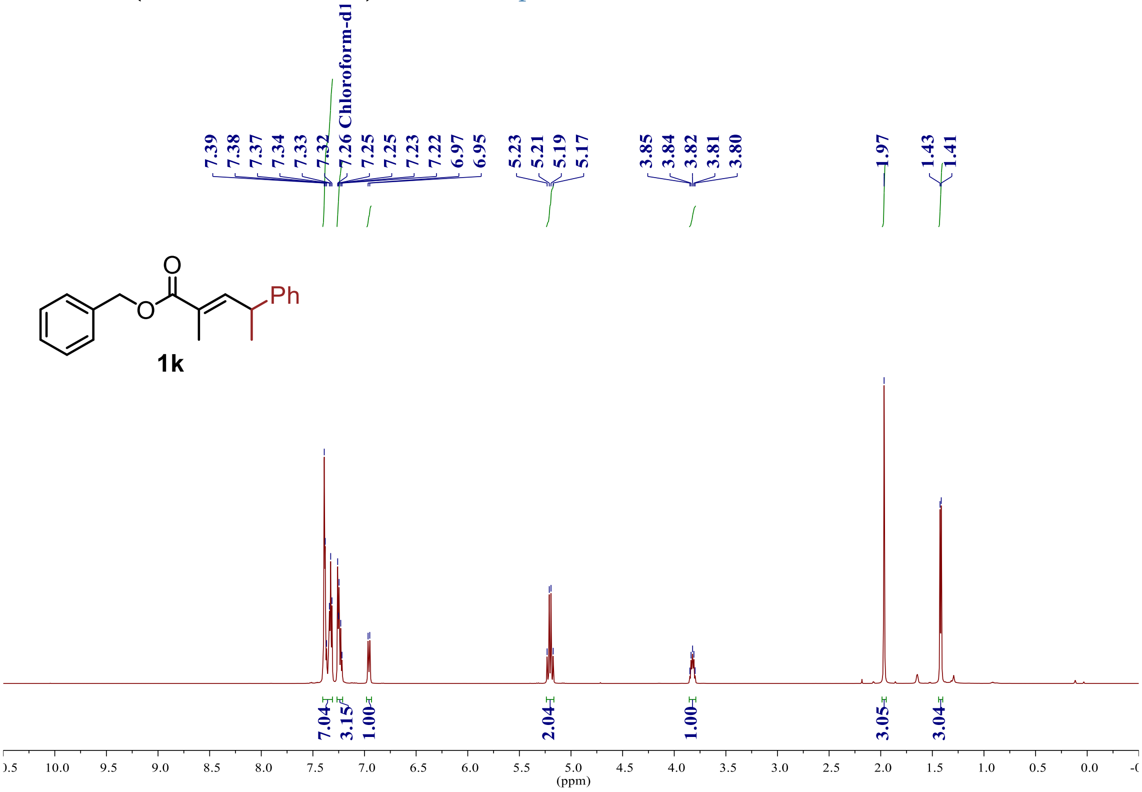

$^{13}C\{^1H\}$ NMR (151 MHz, $CDCl_3$) of **1k**

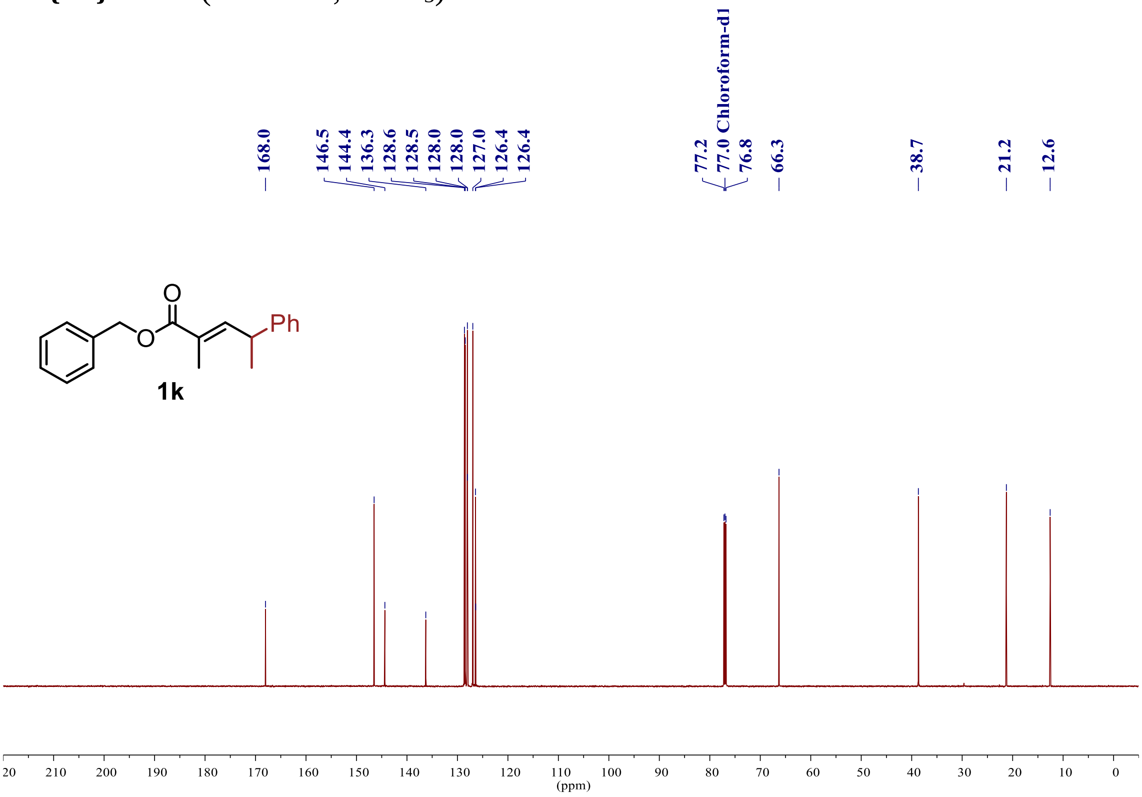

Back to contents

¹H NMR (600 MHz, $CDCl_3$) of **1l**, See procedure

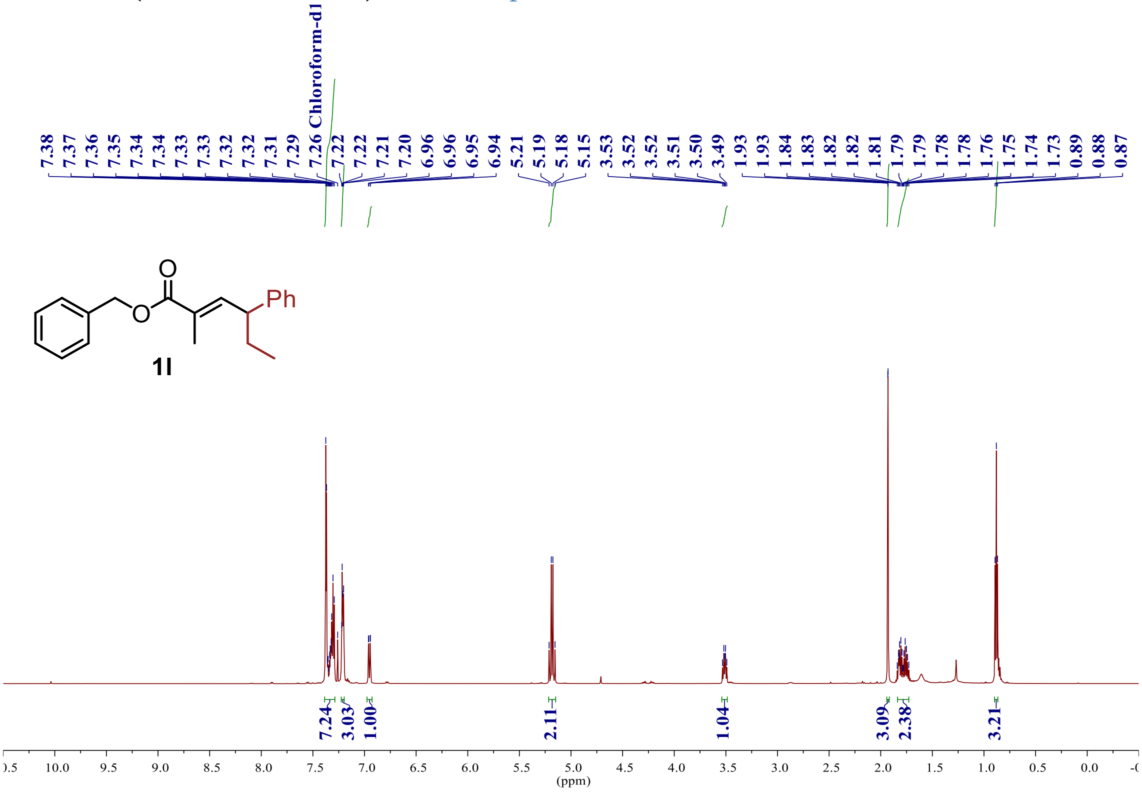

¹³C{¹H} NMR (151 MHz, $CDCl_3$) of **1l**

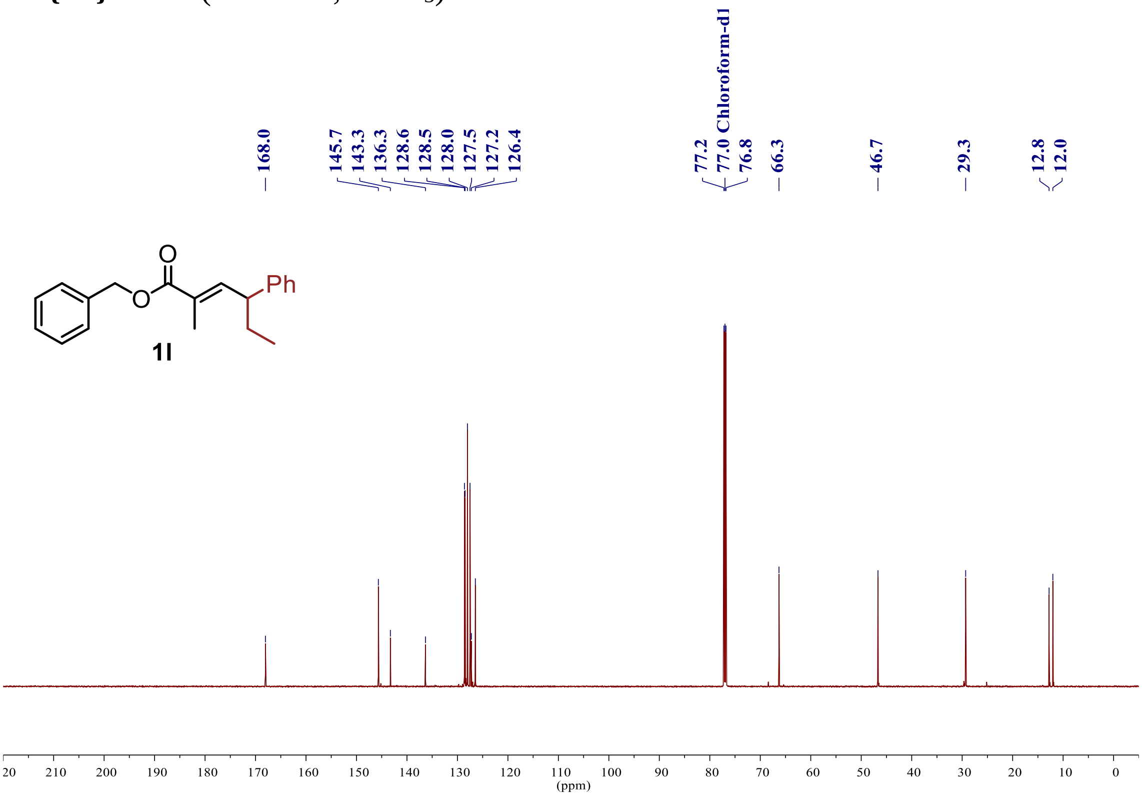

Back to contents

^{1}H NMR (600 MHz, $CDCl_3$) of **1m**, See procedure

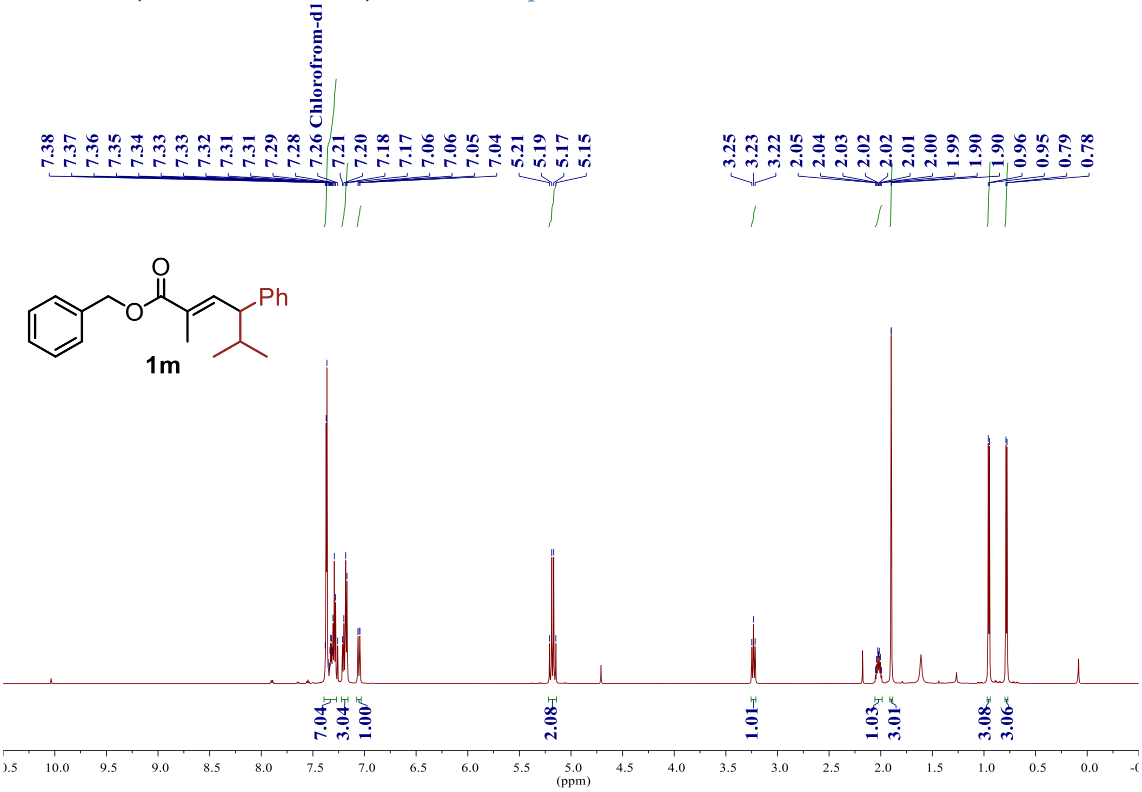

$^{13}C\{^1H\}$ NMR (151 MHz, $CDCl_3$) of **1m**

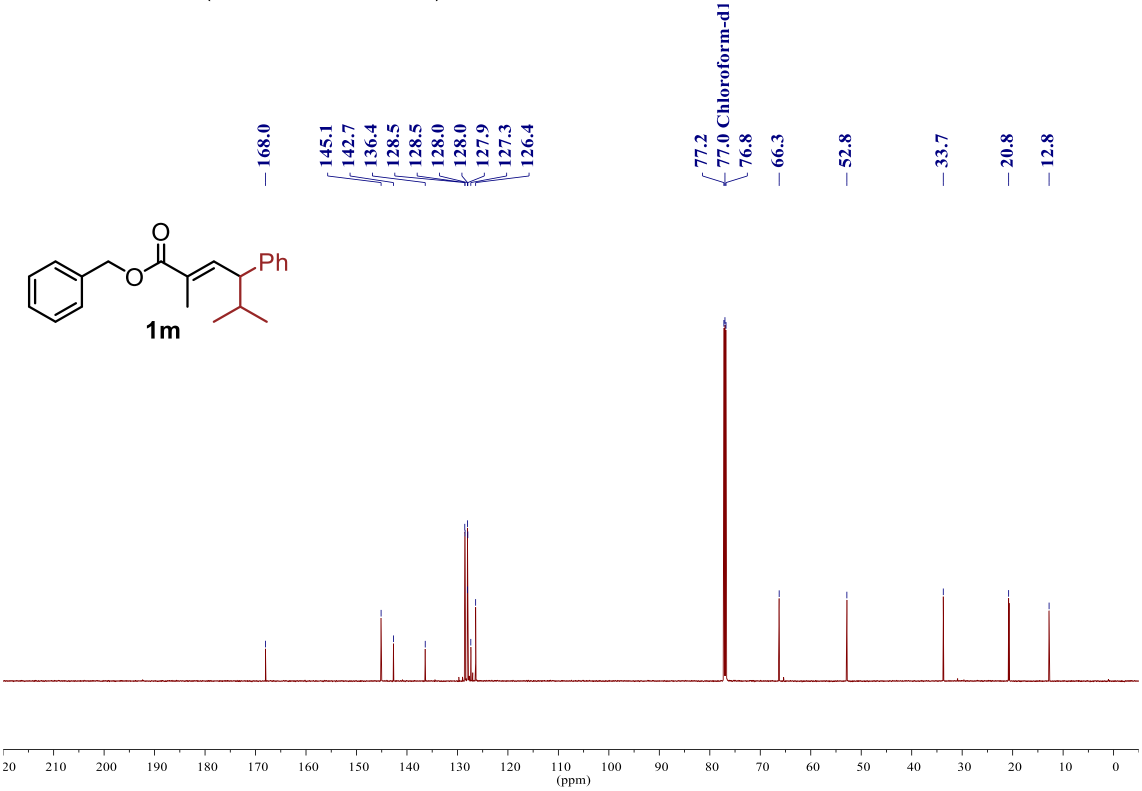

Back to contents

^{1}H NMR (600 MHz, $CDCl_3$) of **1n**, See procedure

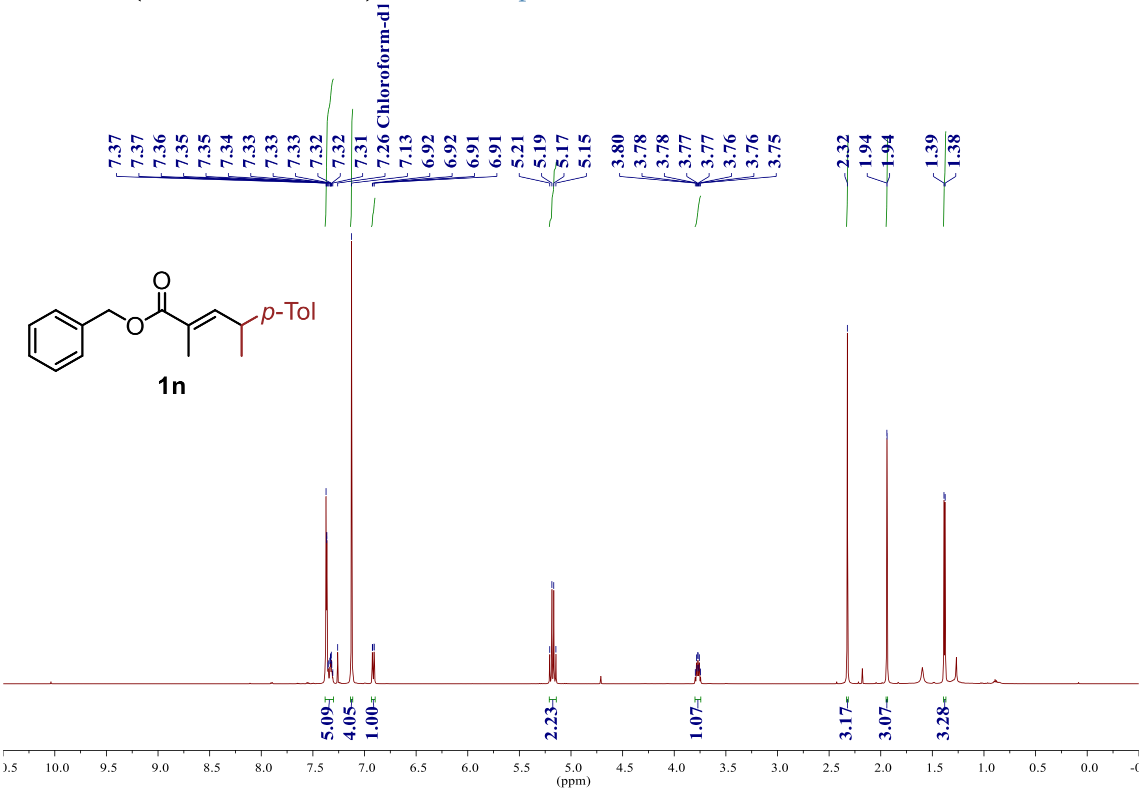

^{13}C{^{1}H} NMR (151 MHz, $CDCl_3$) of **1n**

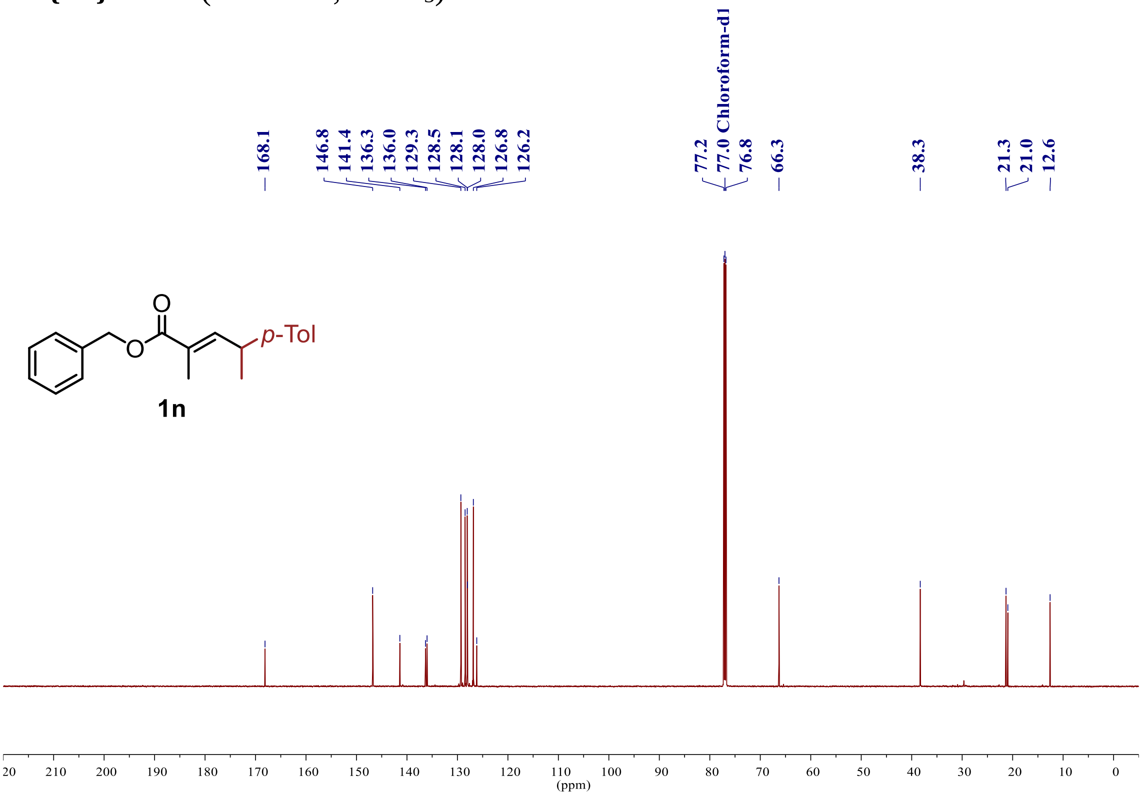

Back to contents

^{1}H NMR (600 MHz, $CDCl_3$) of **1n'**, See procedure

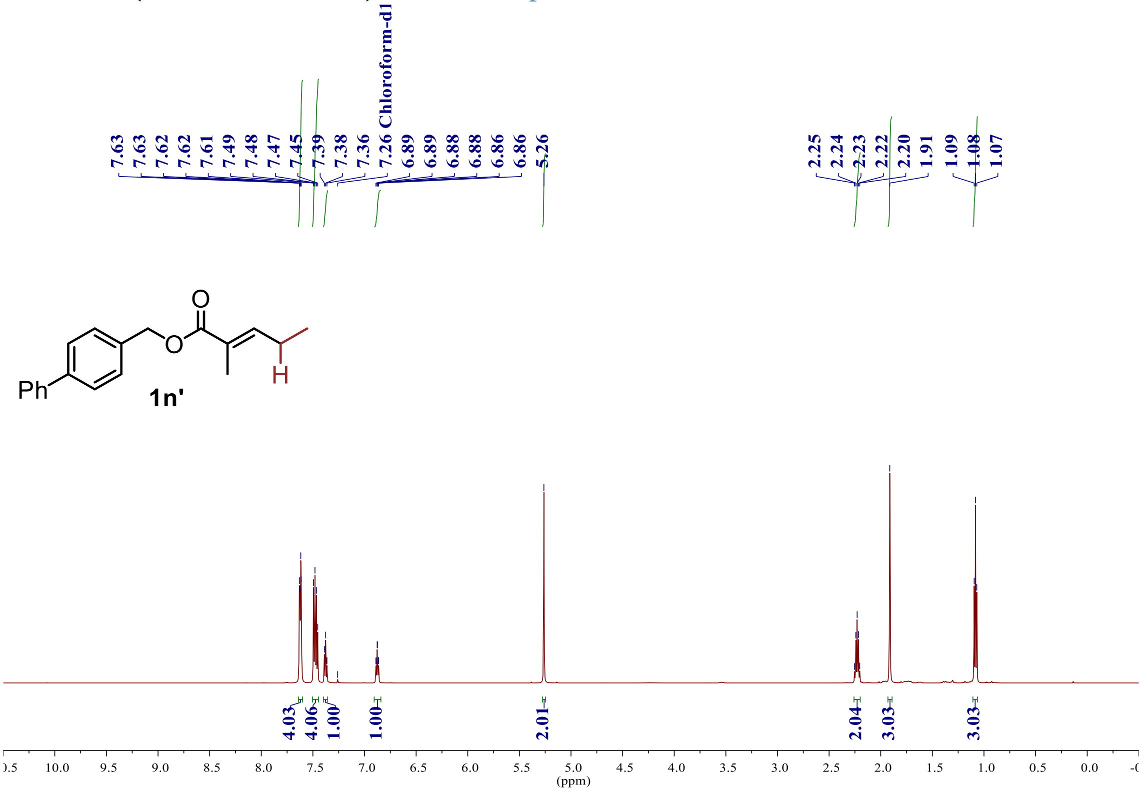

^{13}C{^{1}H} NMR (151 MHz, $CDCl_3$) of **1n'**

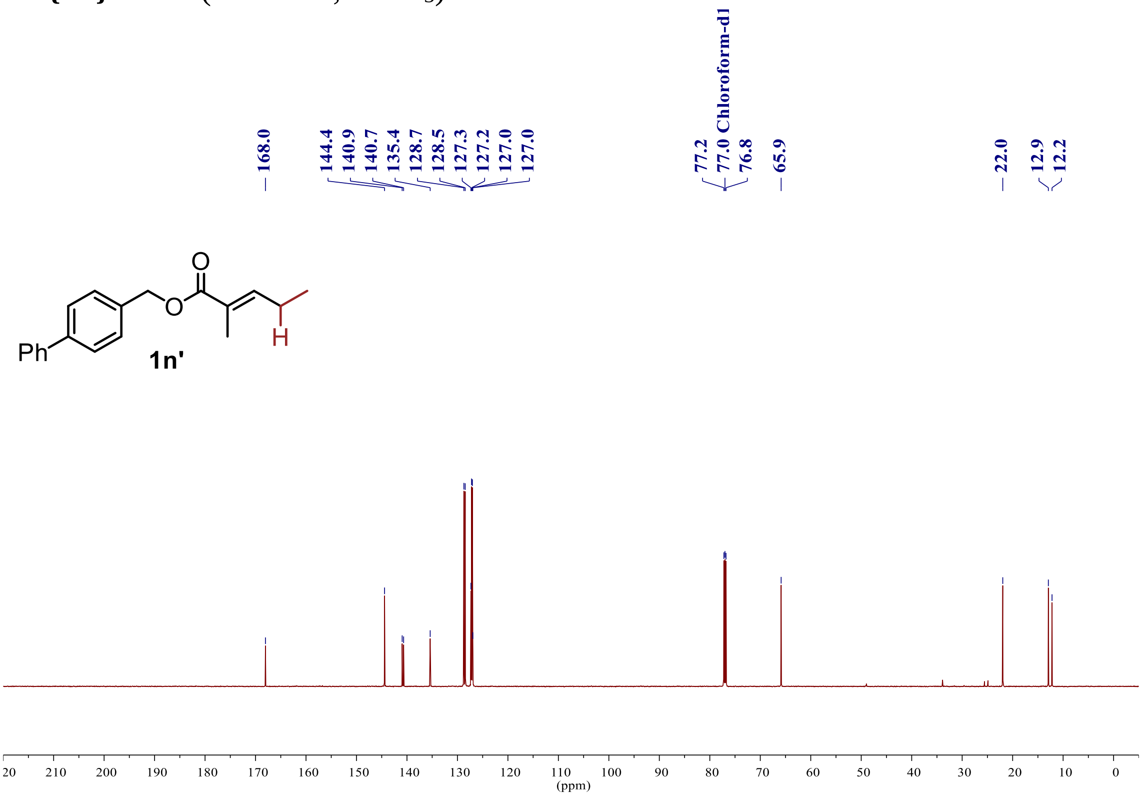

Back to contents

^{1}H NMR (400 MHz, $CDCl_3$) of **1o**, See procedure

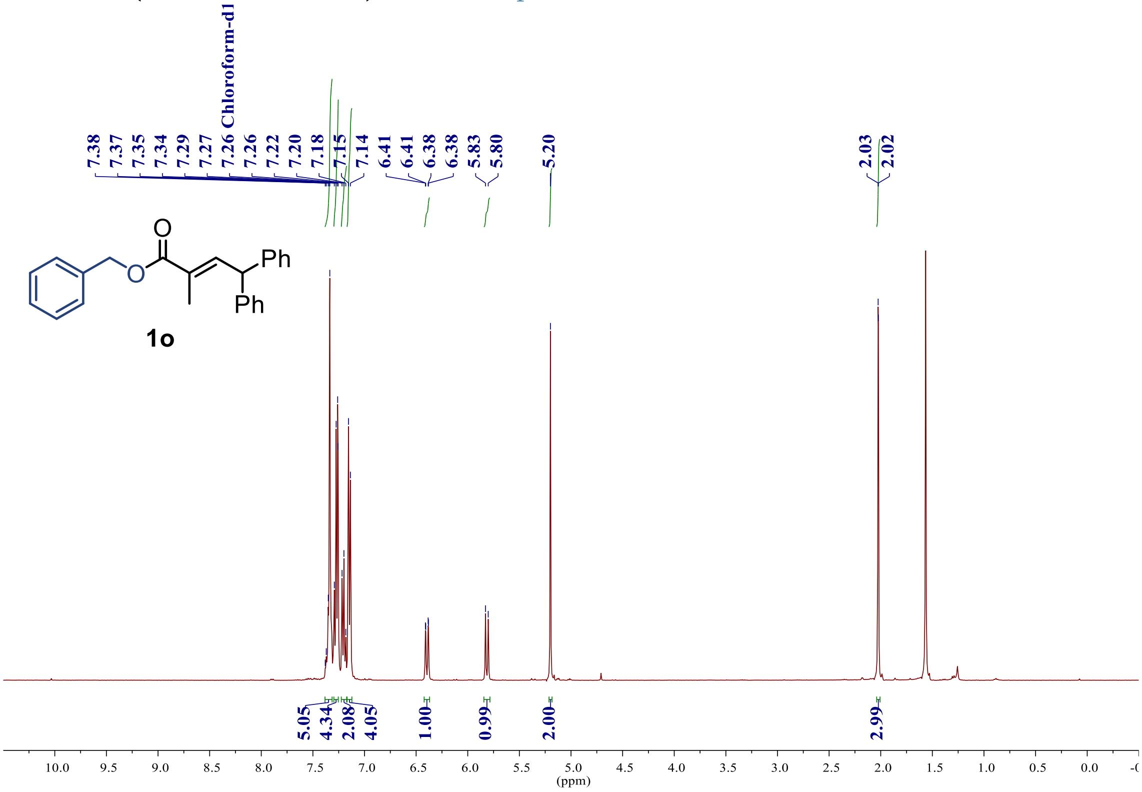

^{13}C{^{1}H} NMR (101 MHz, $CDCl_3$) of **1o**

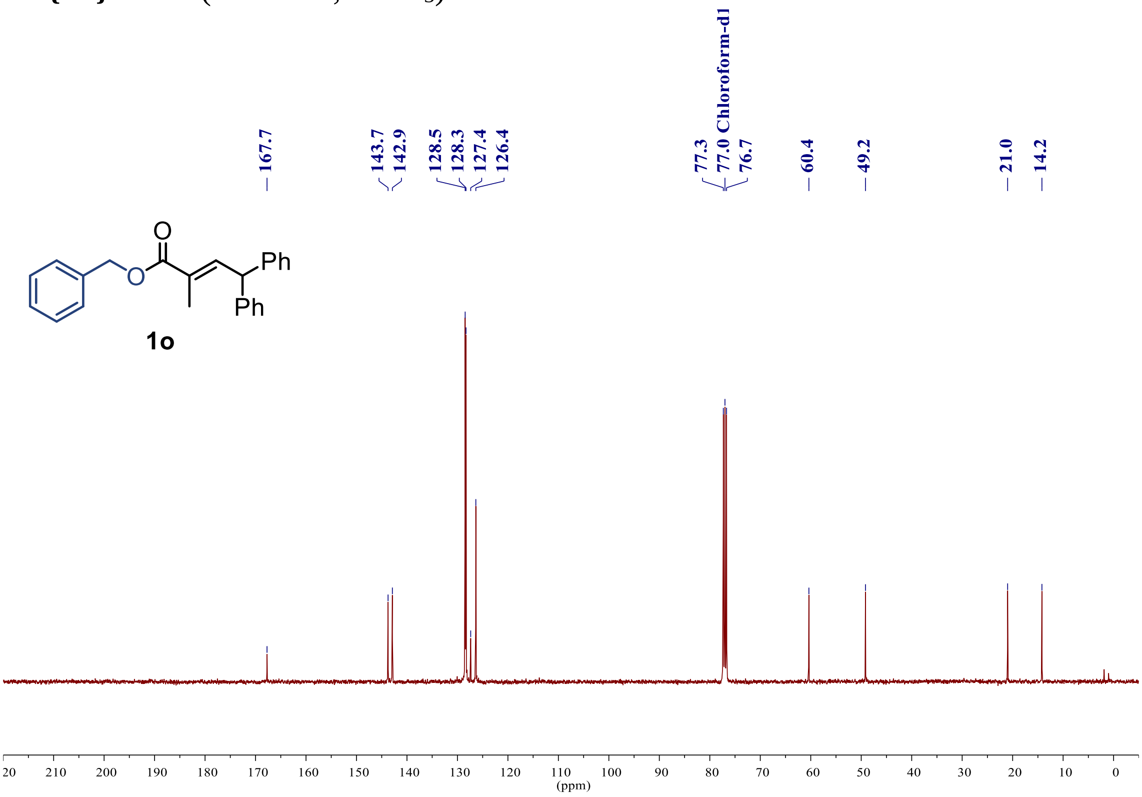

Back to contents

¹H NMR (300 MHz, $CDCl_3$) of **1p**, See procedure

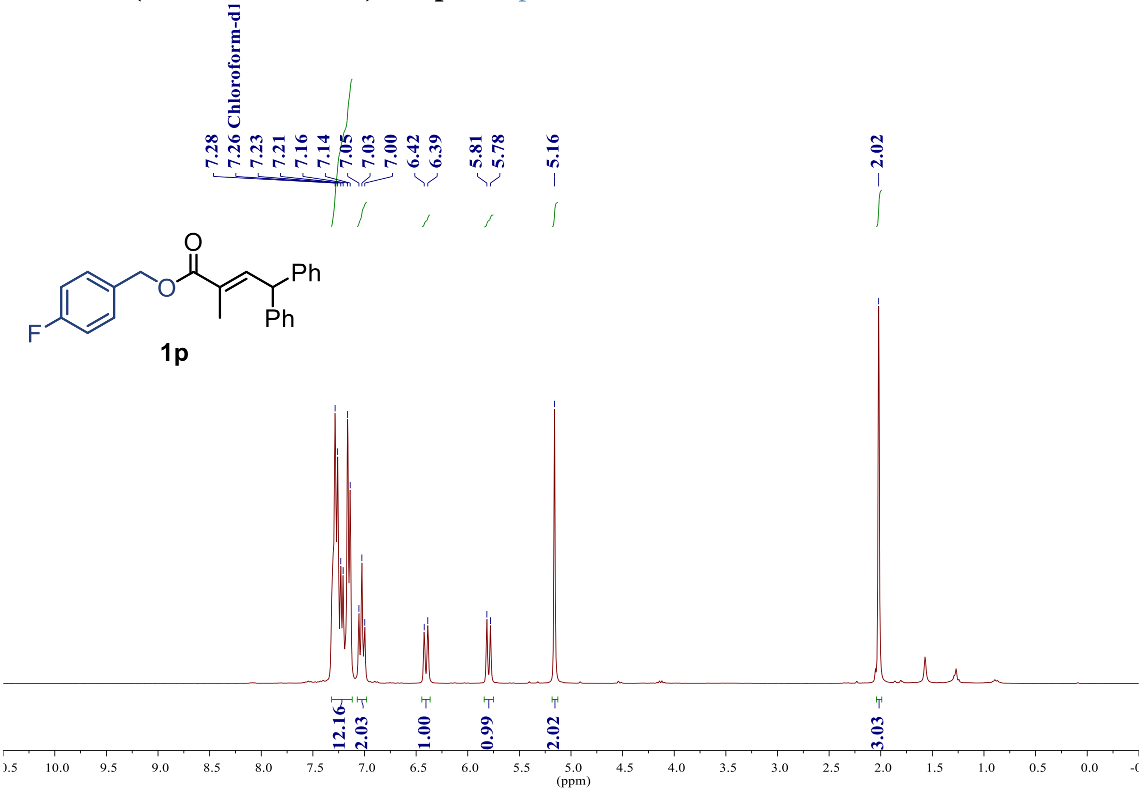

$^{13}C\{^1H\}$ NMR (101 MHz, $CDCl_3$) of **1p**

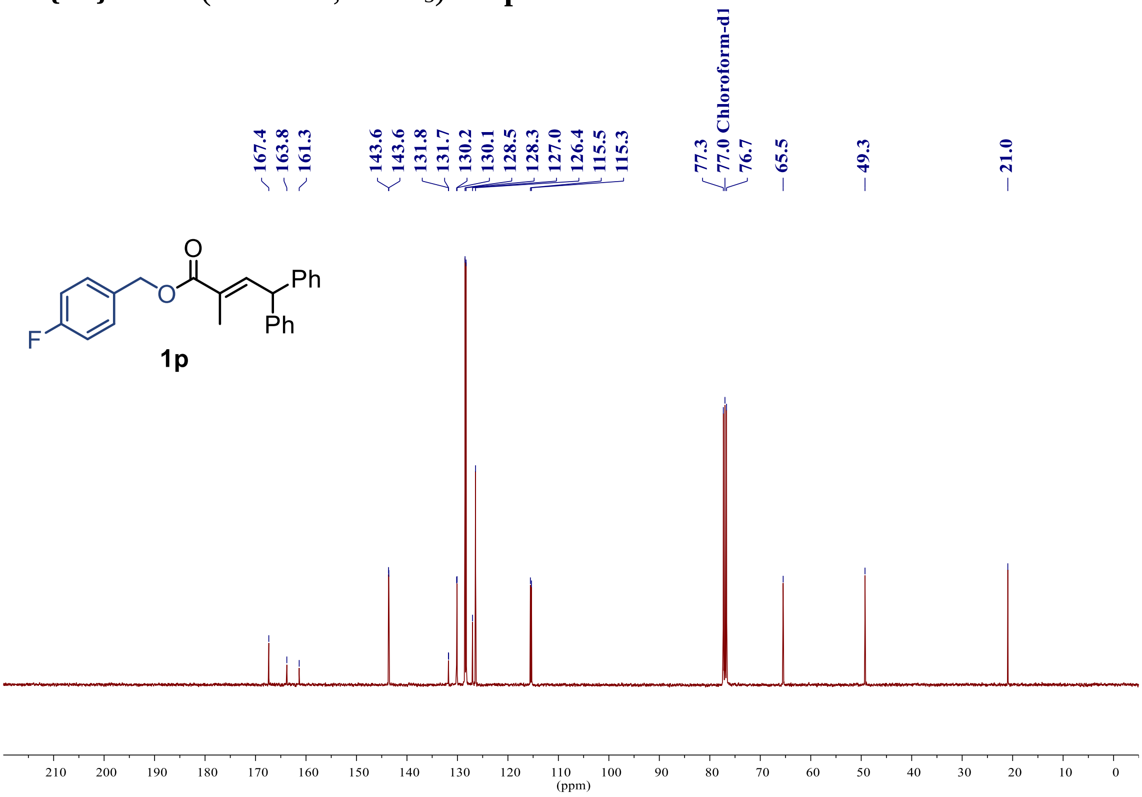

Back to contents

^{19}F NMR (282 MHz, $CDCl_3$) of **1p**

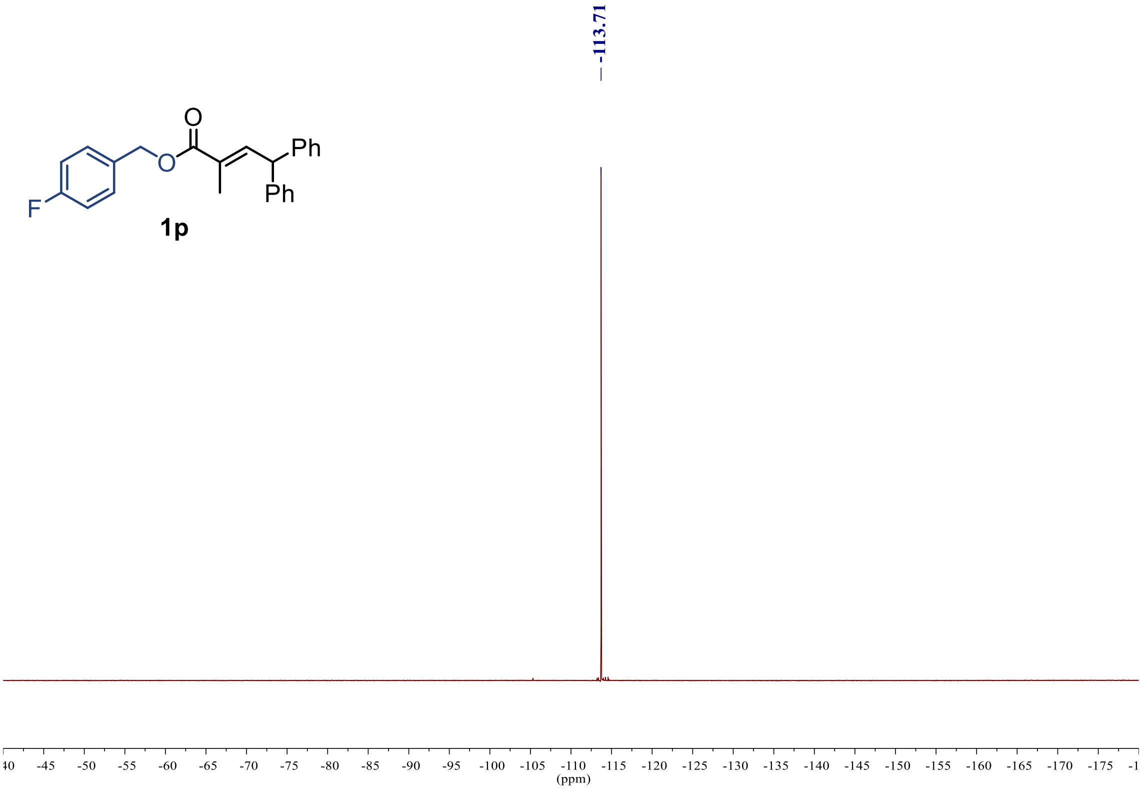

1p

^{1}H NMR (300 MHz, $CDCl_3$) of **1q**, See procedure

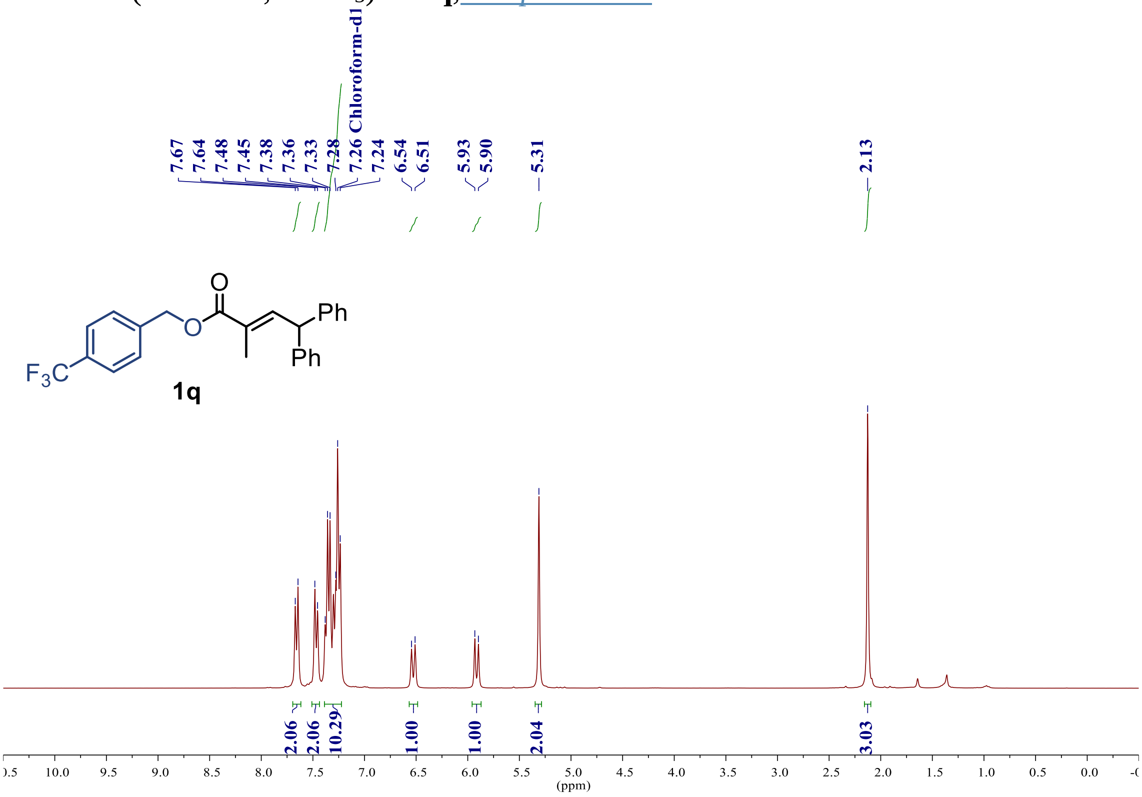

1q

Back to contents

^{13}C{^{1}H} NMR (101 MHz, $CDCl_3$) of **1q**

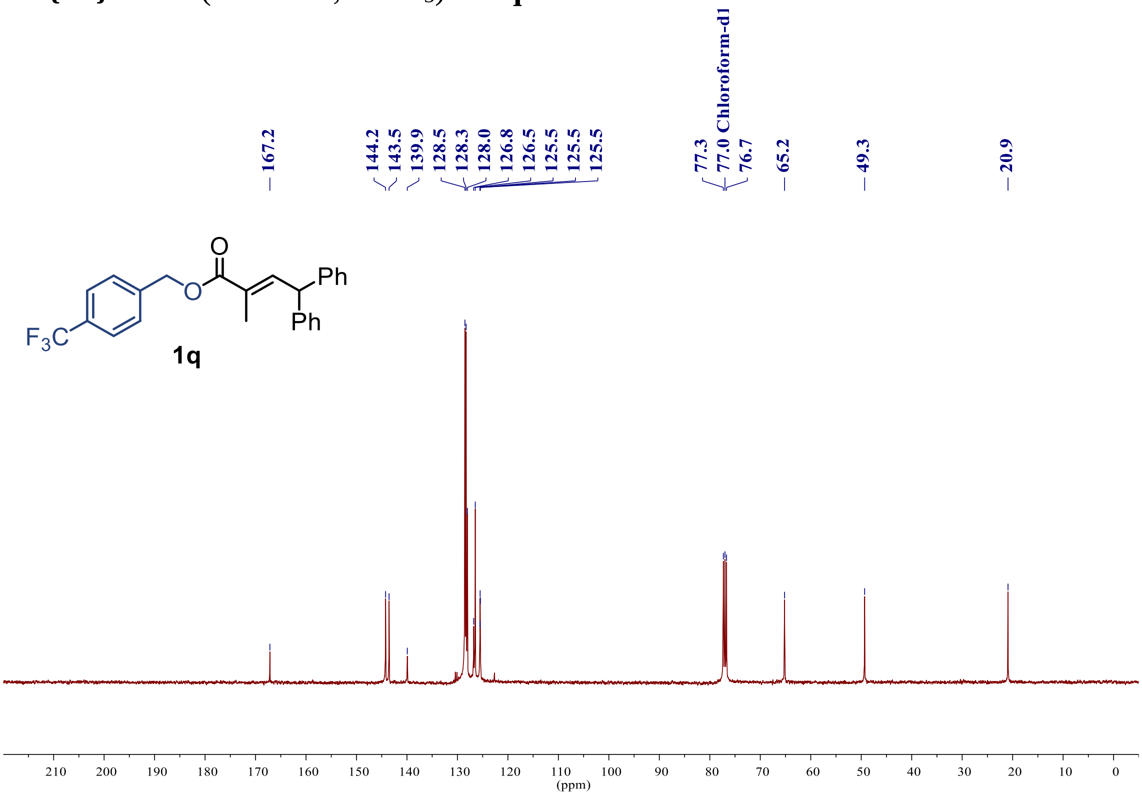

^{19}F NMR (282 MHz, $CDCl_3$) of **1q**

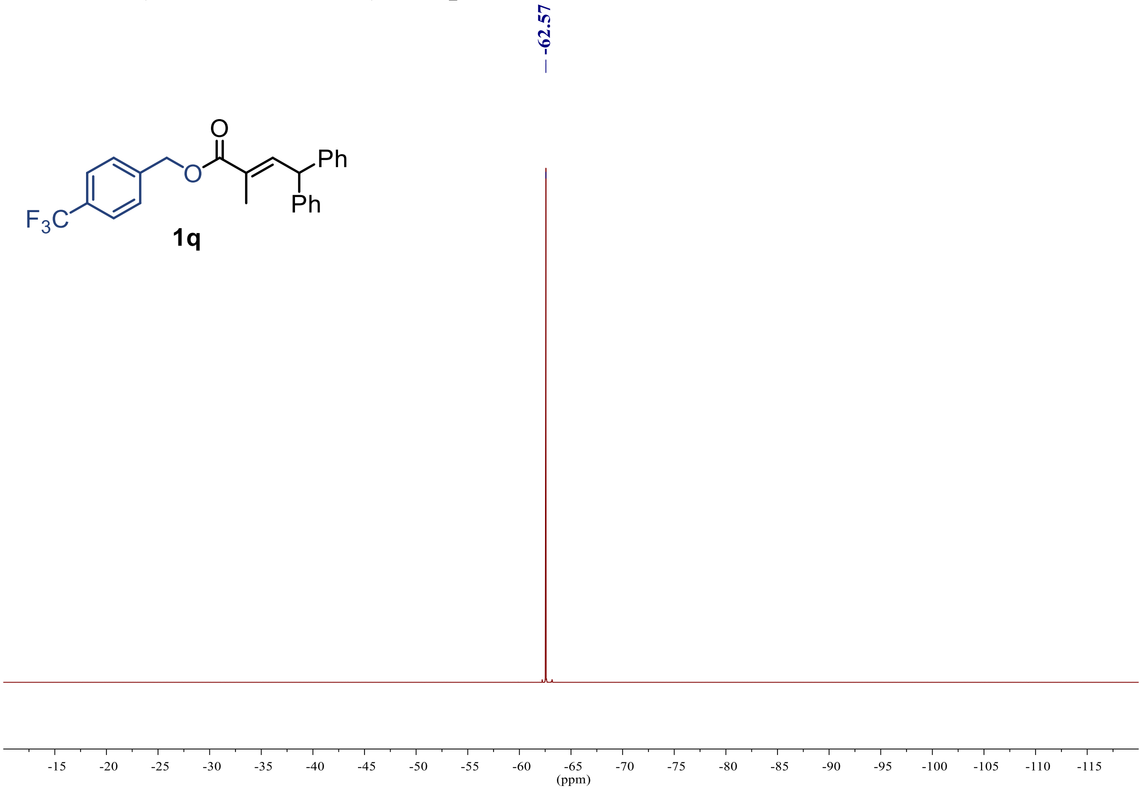

Back to contents

^{1}H NMR (400 MHz, $CDCl_3$) of **1r**, See procedure

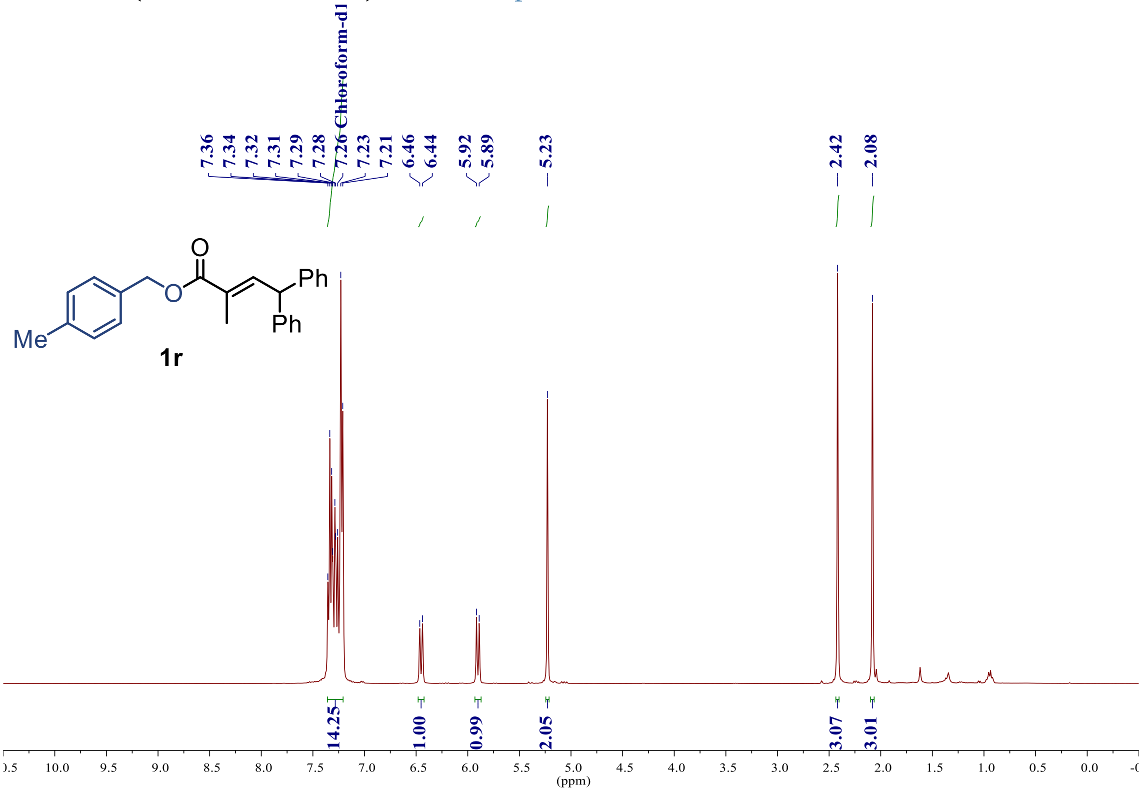

^{13}C{^{1}H} NMR (101 MHz, $CDCl_3$) of **1r**

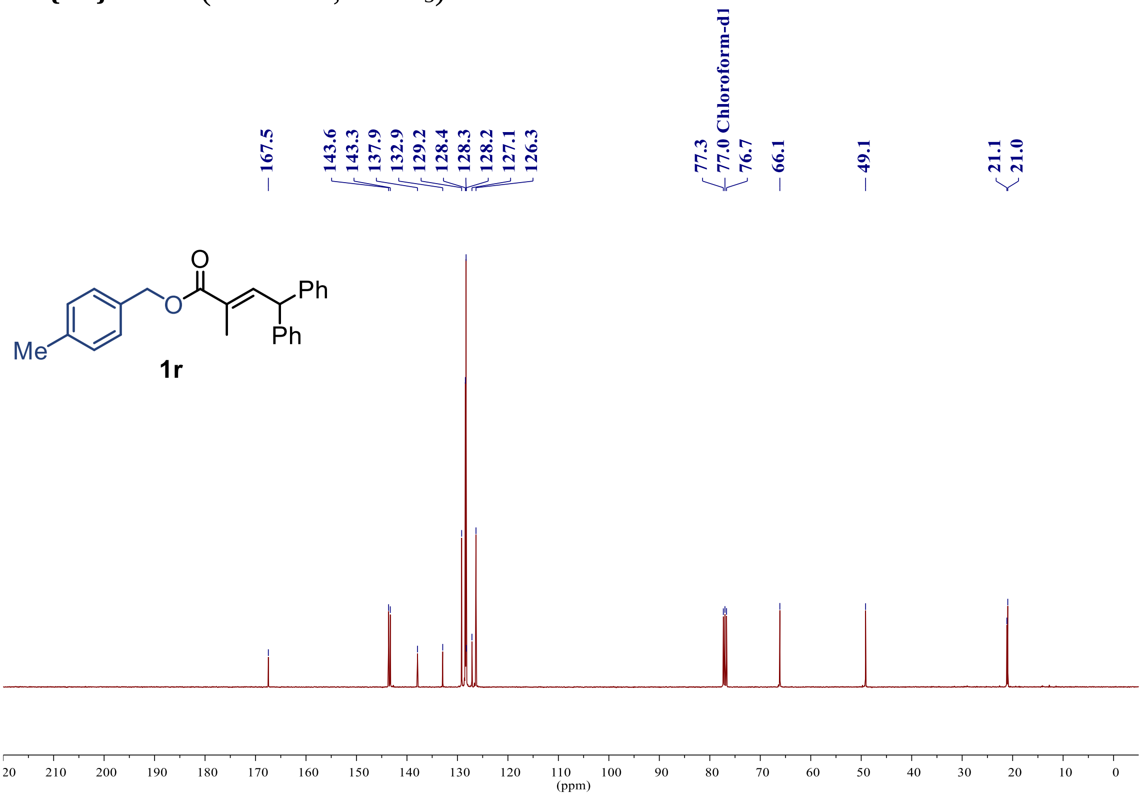

[Back to contents]

^{1}H NMR (300 MHz, $CDCl_3$) of **1s**, See procedure

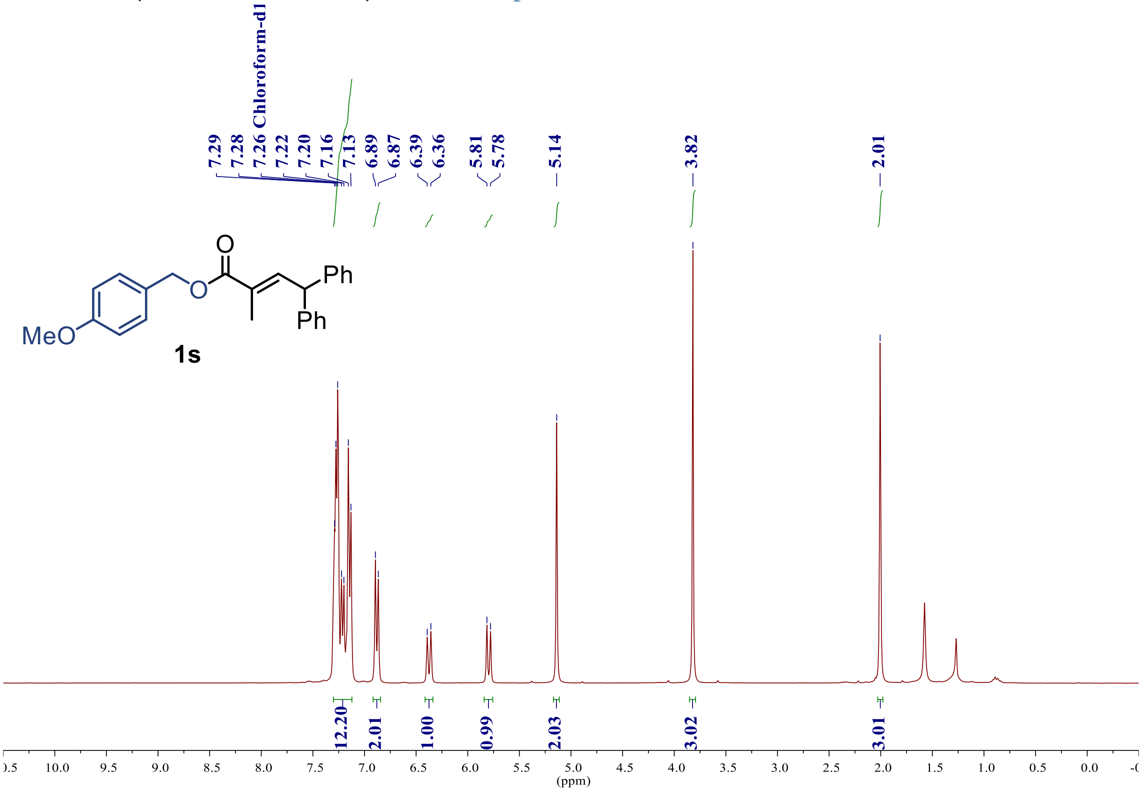

^{13}C{^{1}H} NMR (101 MHz, $CDCl_3$) of **1s**

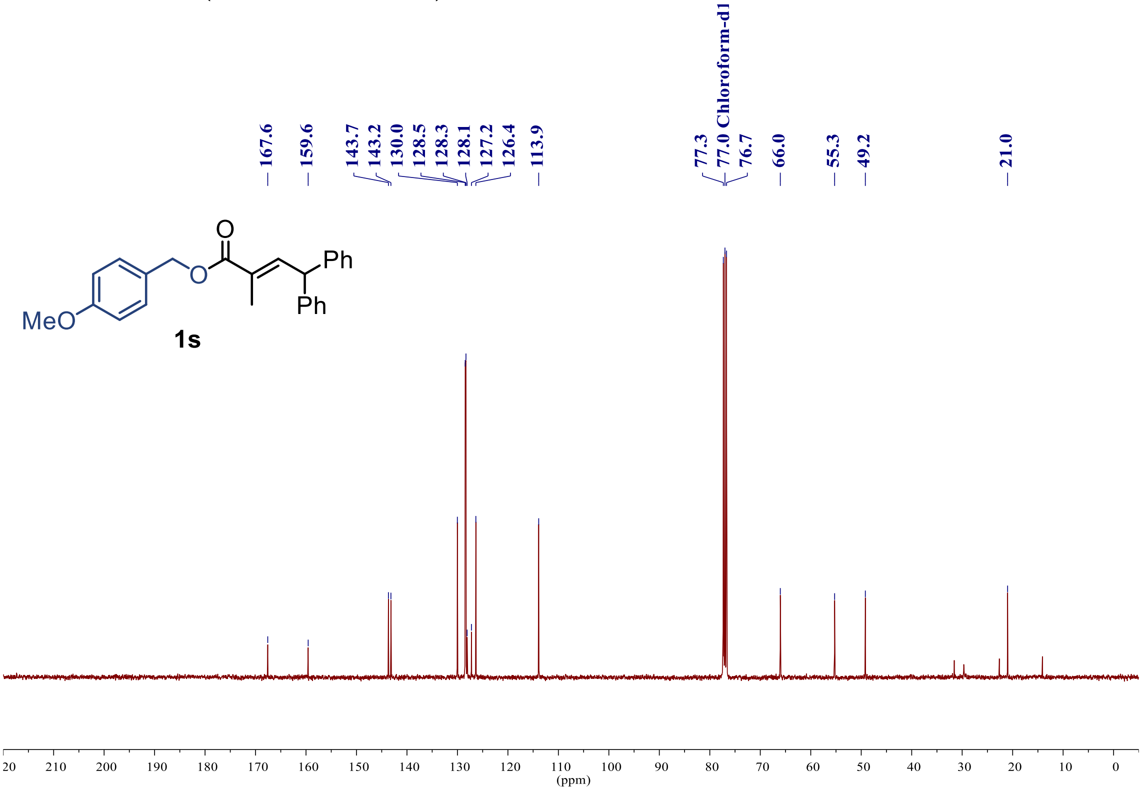

Back to contents

^{1}H NMR (300 MHz, $CDCl_3$) of **1t**, See procedure

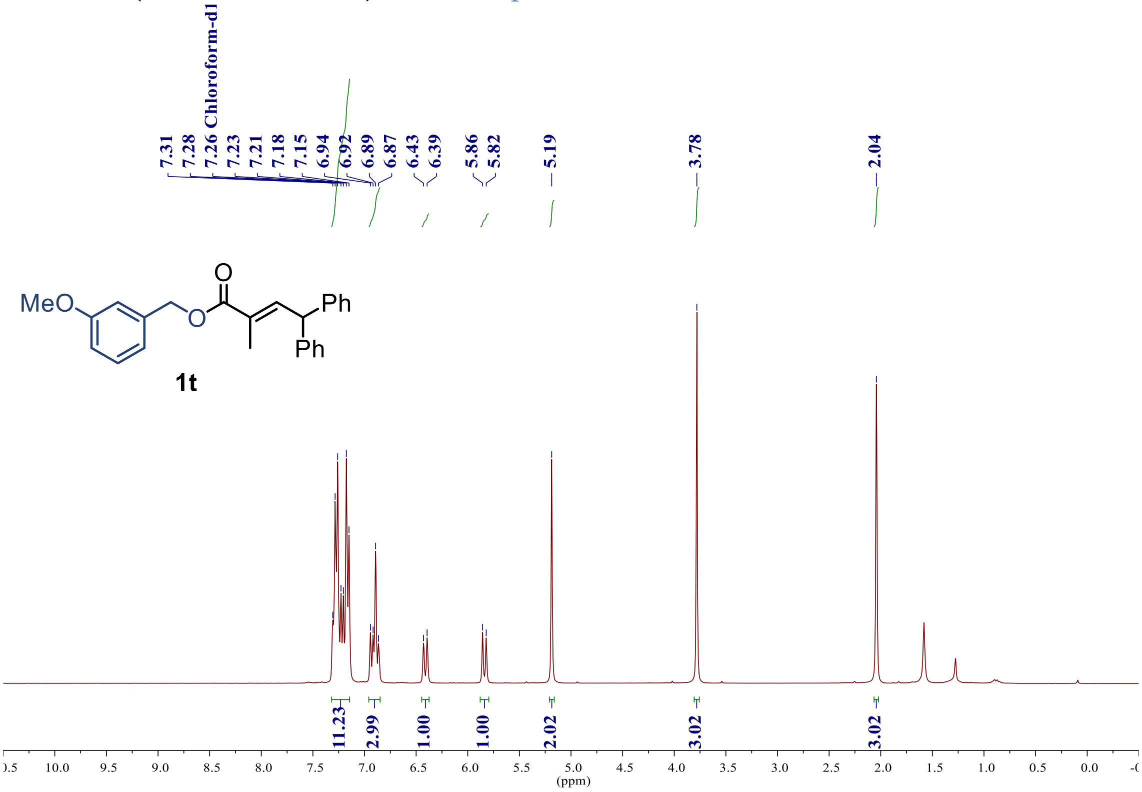

^{13}C{^{1}H} NMR (101 MHz, $CDCl_3$) of **1t**

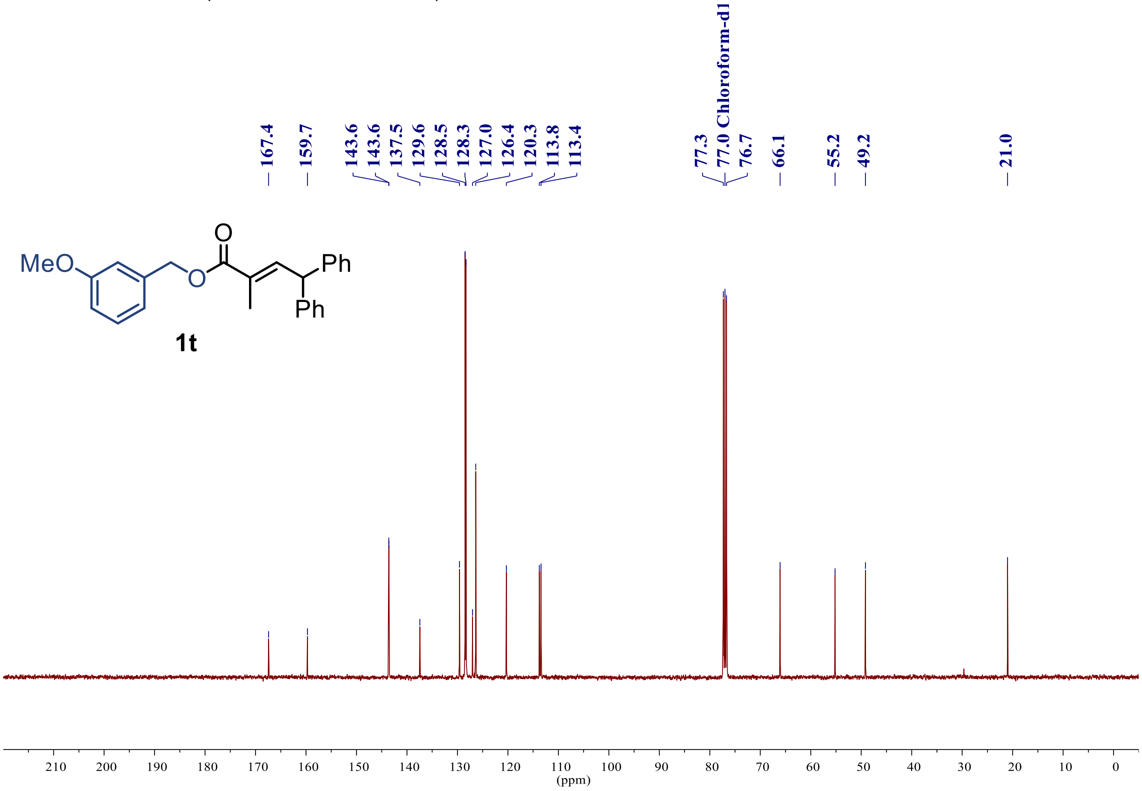

Back to contents

^{1}H NMR (400 MHz, $CDCl_3$) of **1u**, See procedure

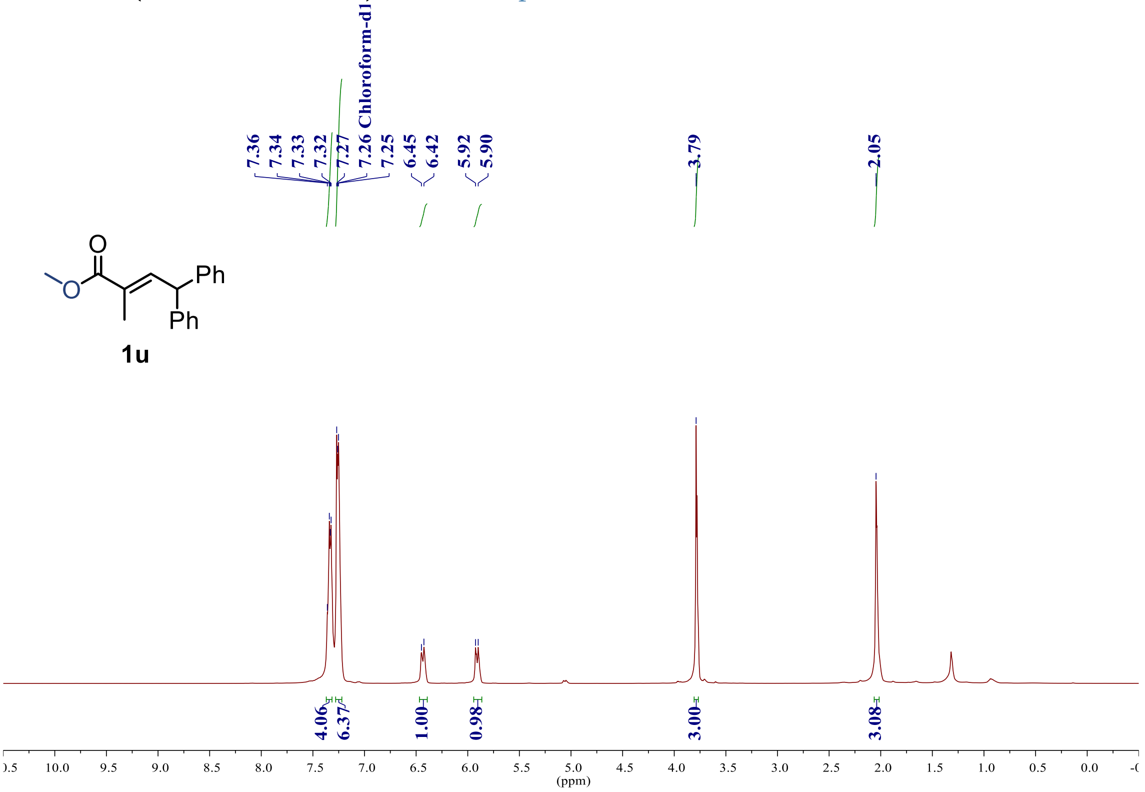

^{13}C{^{1}H} NMR (101 MHz, $CDCl_3$) of **1u**

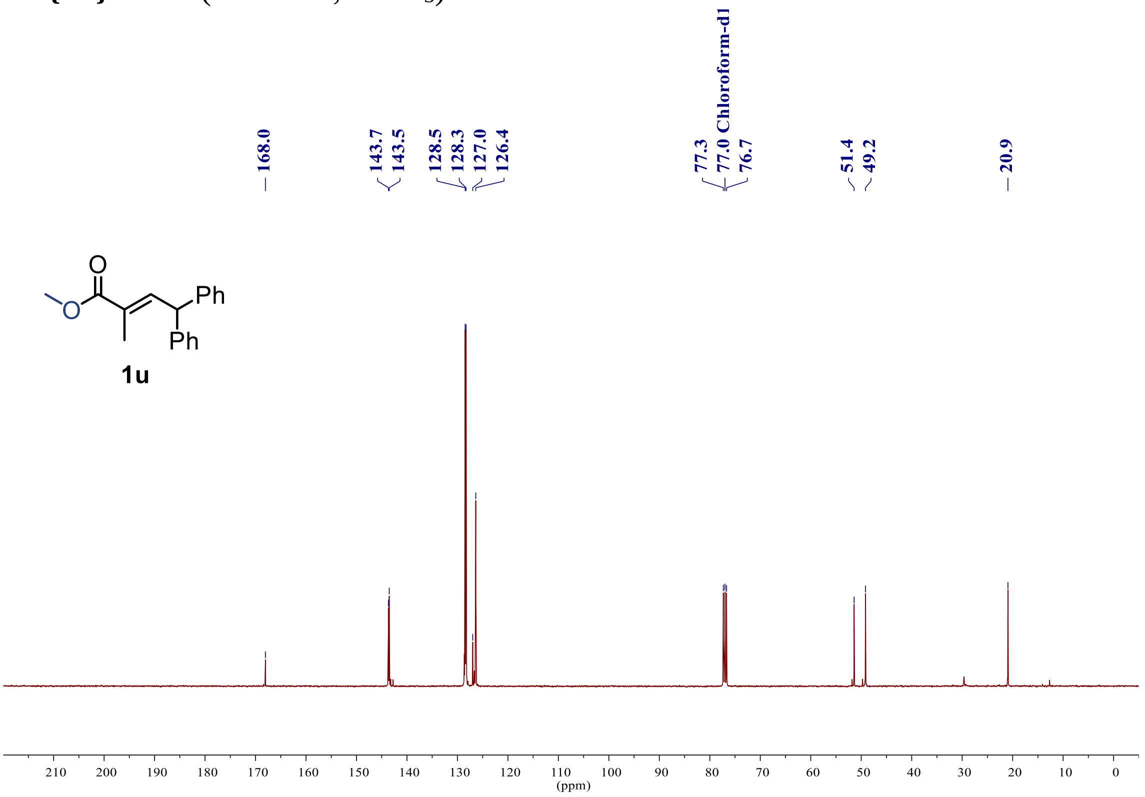

Back to contents

^{1}H NMR (300 MHz, $CDCl_3$) of **1w**, See procedure

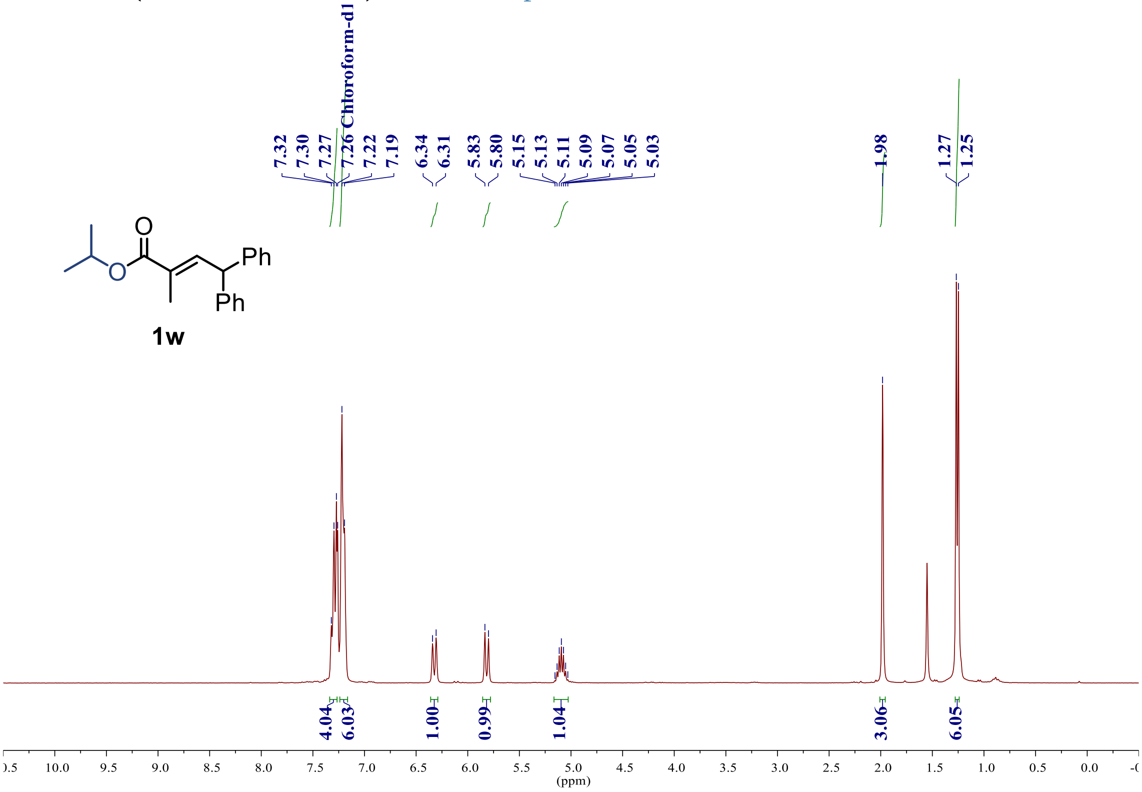

^{13}C{^{1}H} NMR (101 MHz, $CDCl_3$) of **1w**

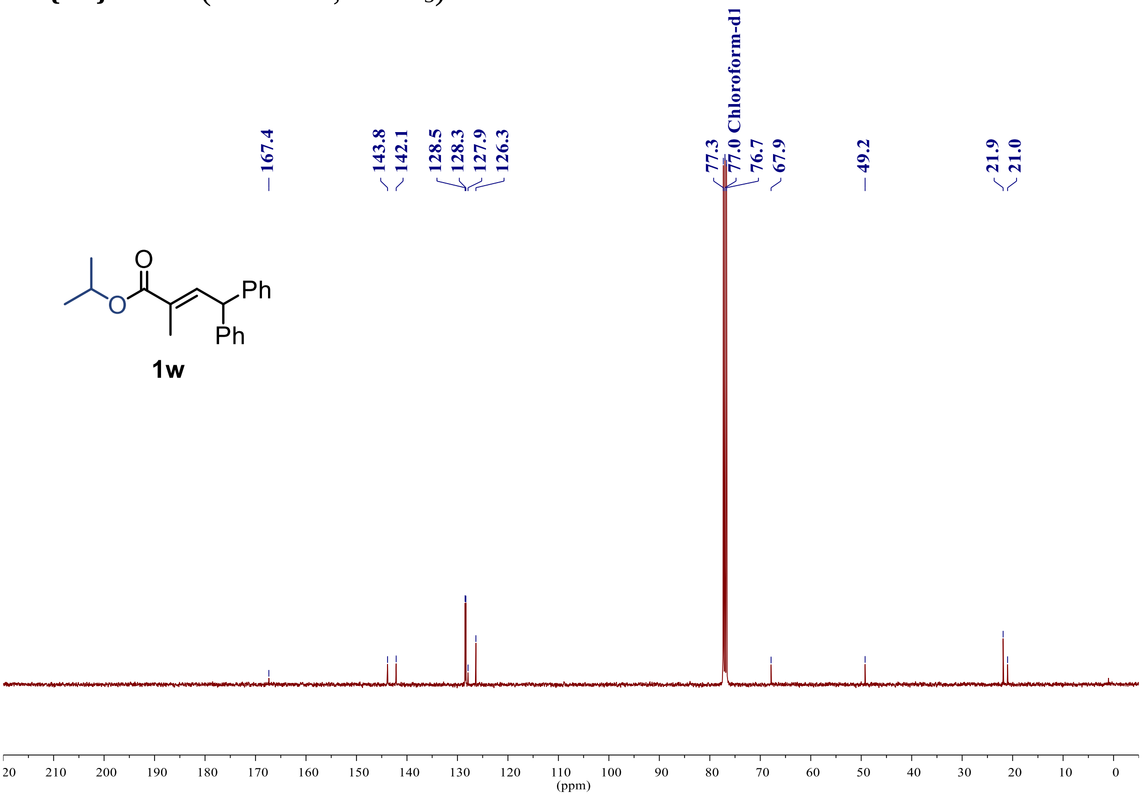

Back to contents

^{1}H NMR (300 MHz, $CDCl_3$) of **1x**, See procedure

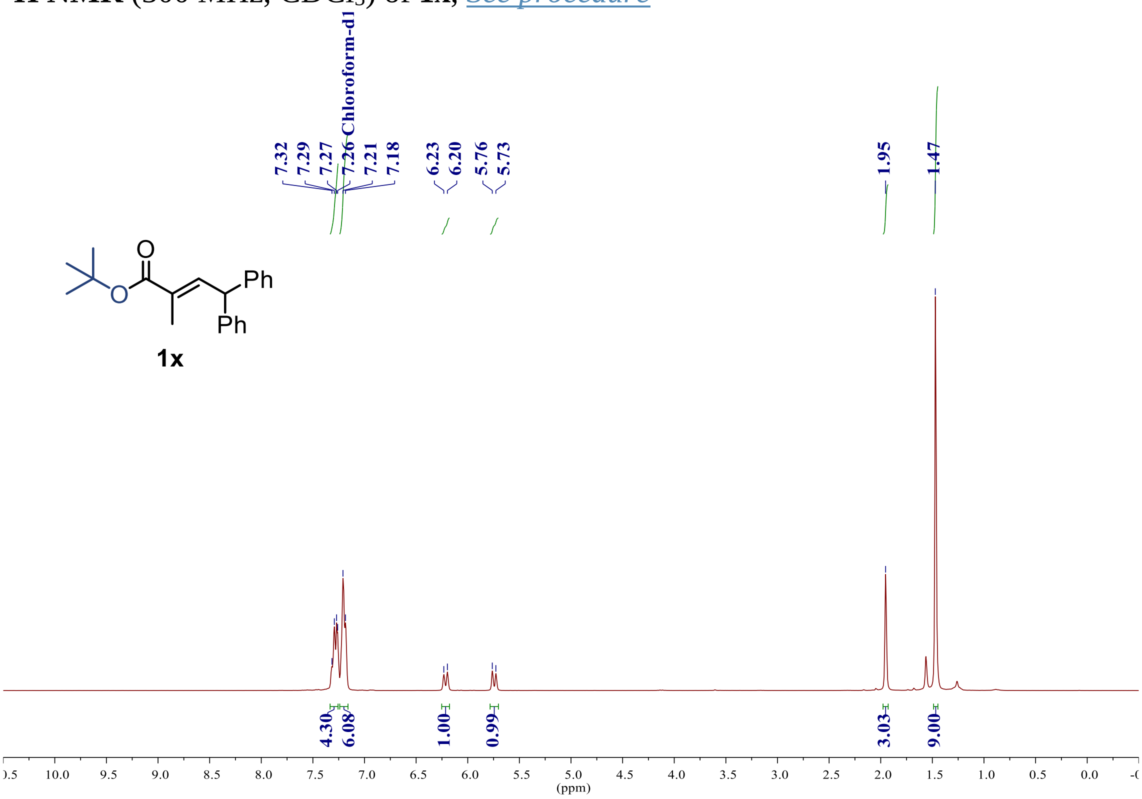

^{13}C{^{1}H} NMR (101 MHz, $CDCl_3$) of **1x**

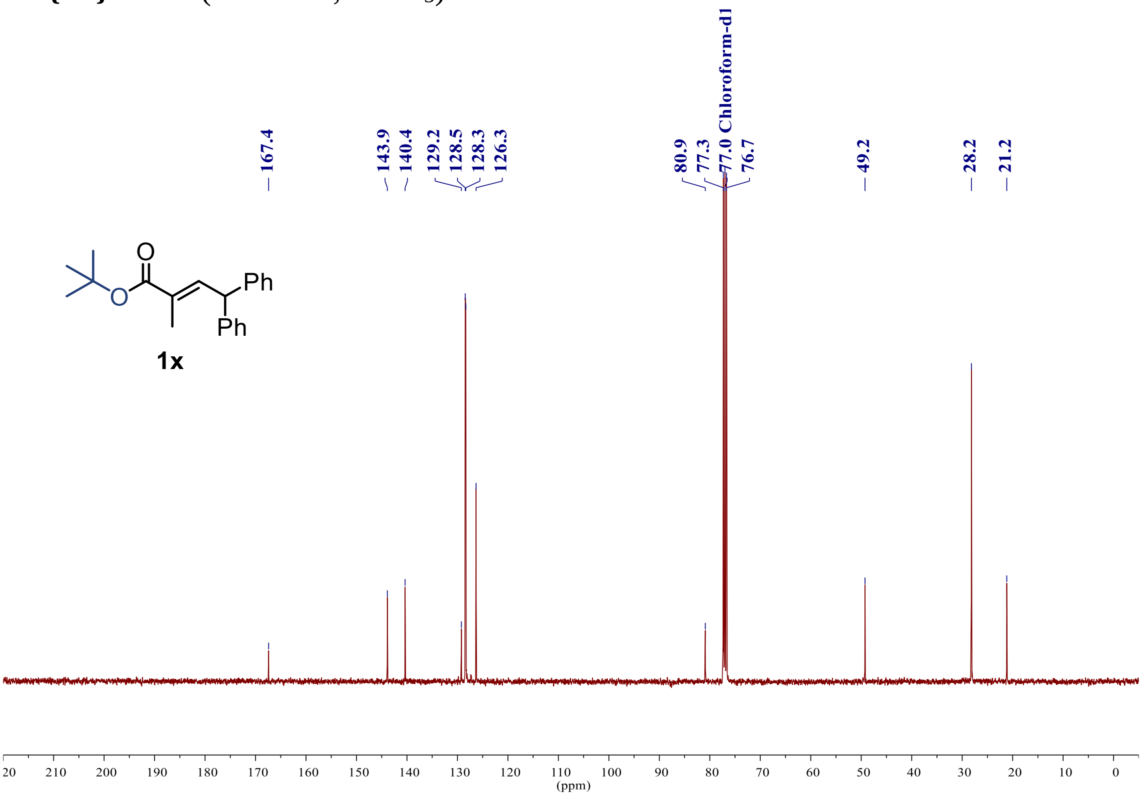

Back to contents

^{1}H NMR (400 MHz, $CDCl_3$) of **1y**, See procedure

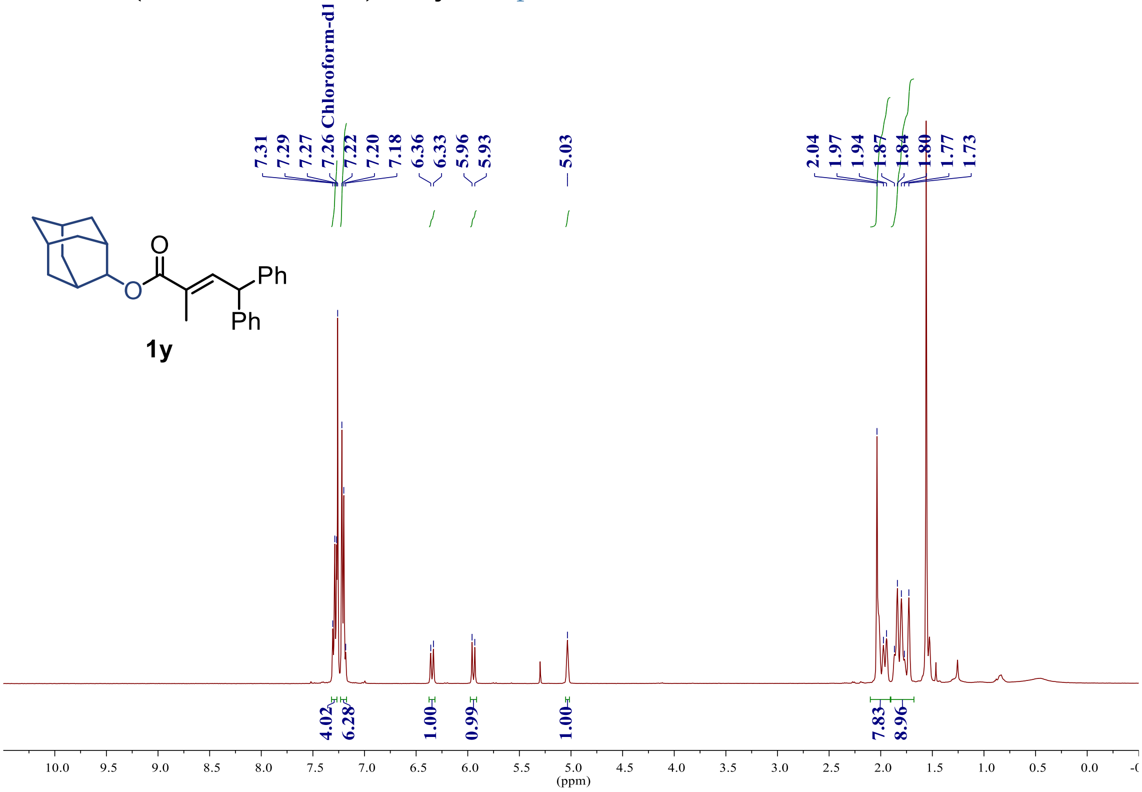

^{13}C{^{1}H} NMR (101 MHz, $CDCl_3$) of **1y**

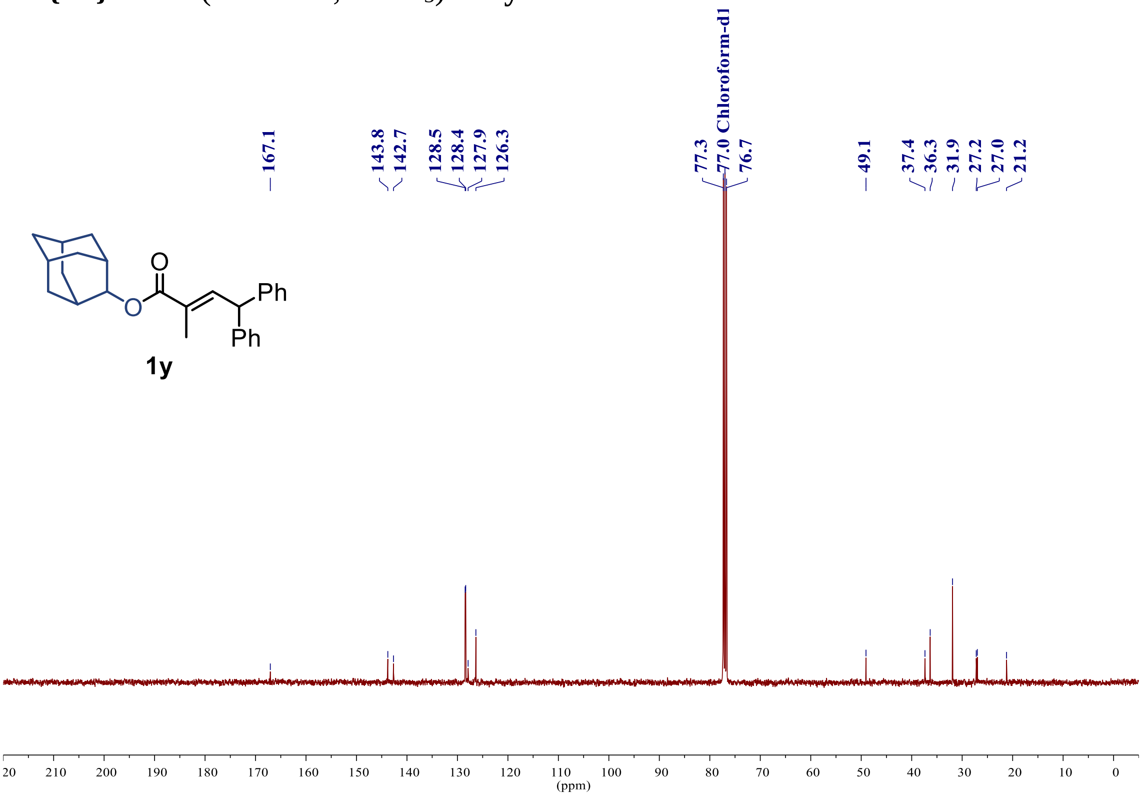

Back to contents

^{1}H NMR (400 MHz, $CDCl_3$) of **1z**, See procedure

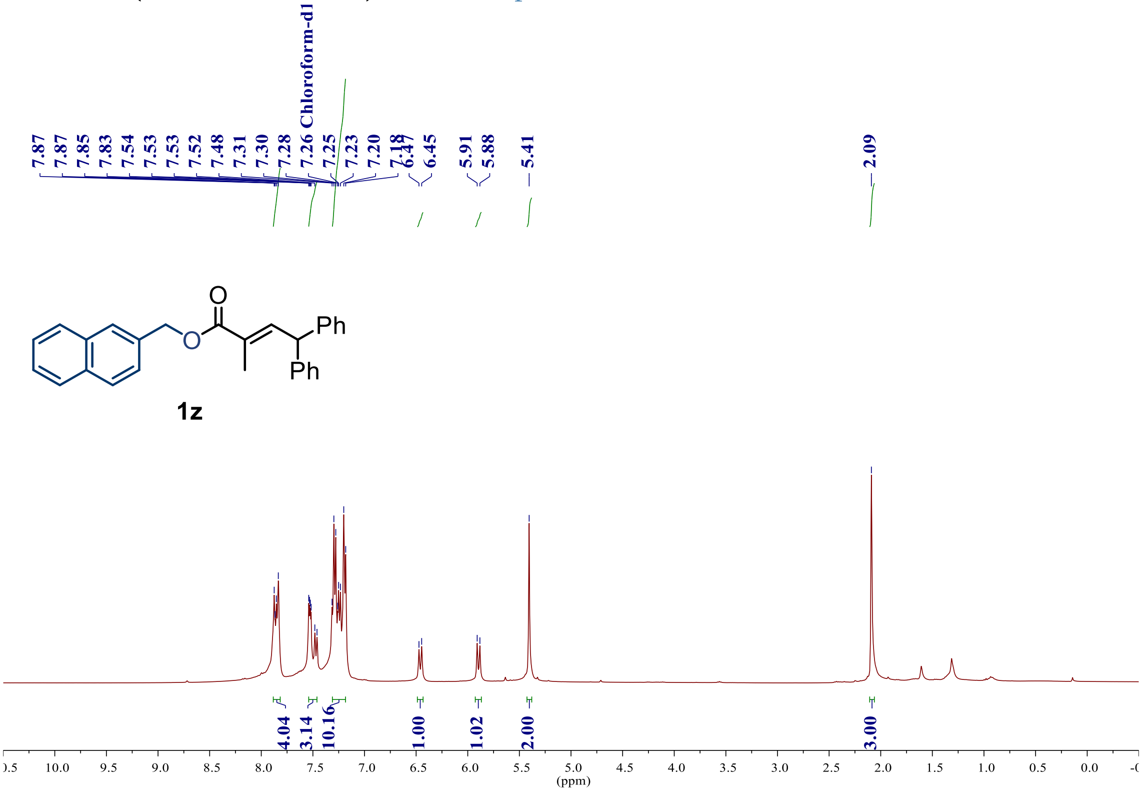

^{13}C{^{1}H} NMR (101 MHz, $CDCl_3$) of **1z**

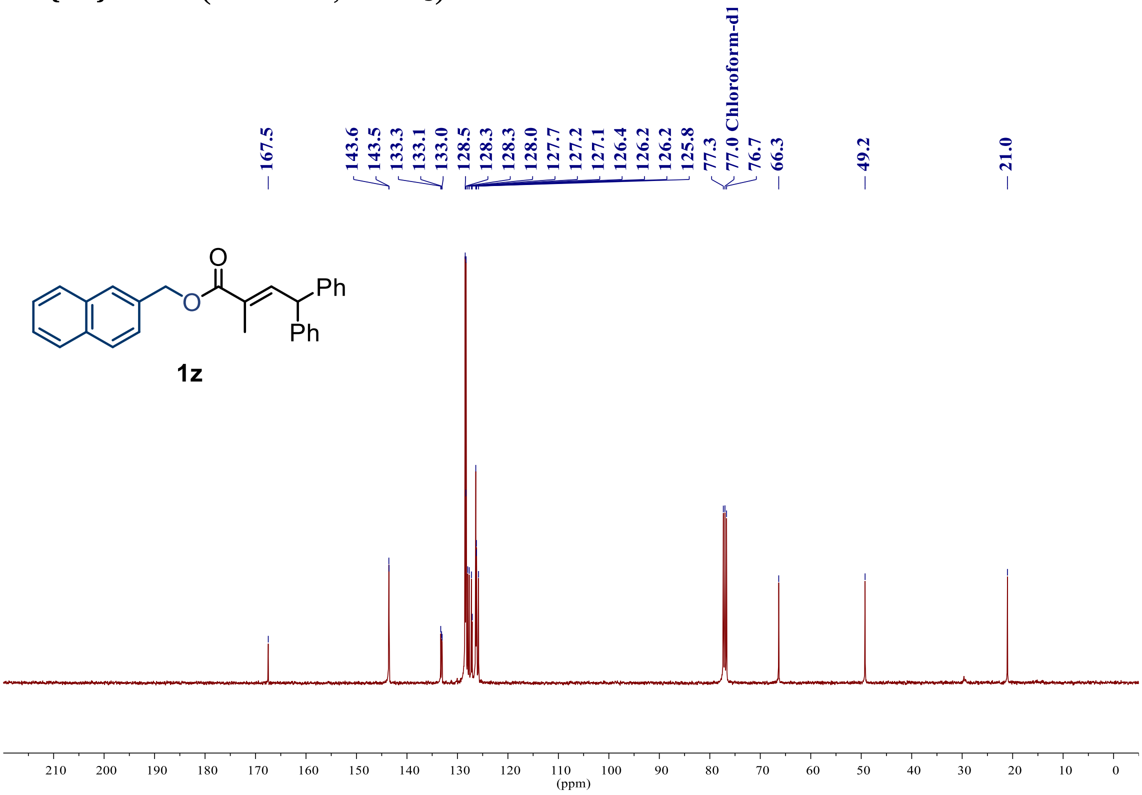

Back to contents

^{1}H NMR (500 MHz, $CDCl_3$) of **1aa**, See procedure

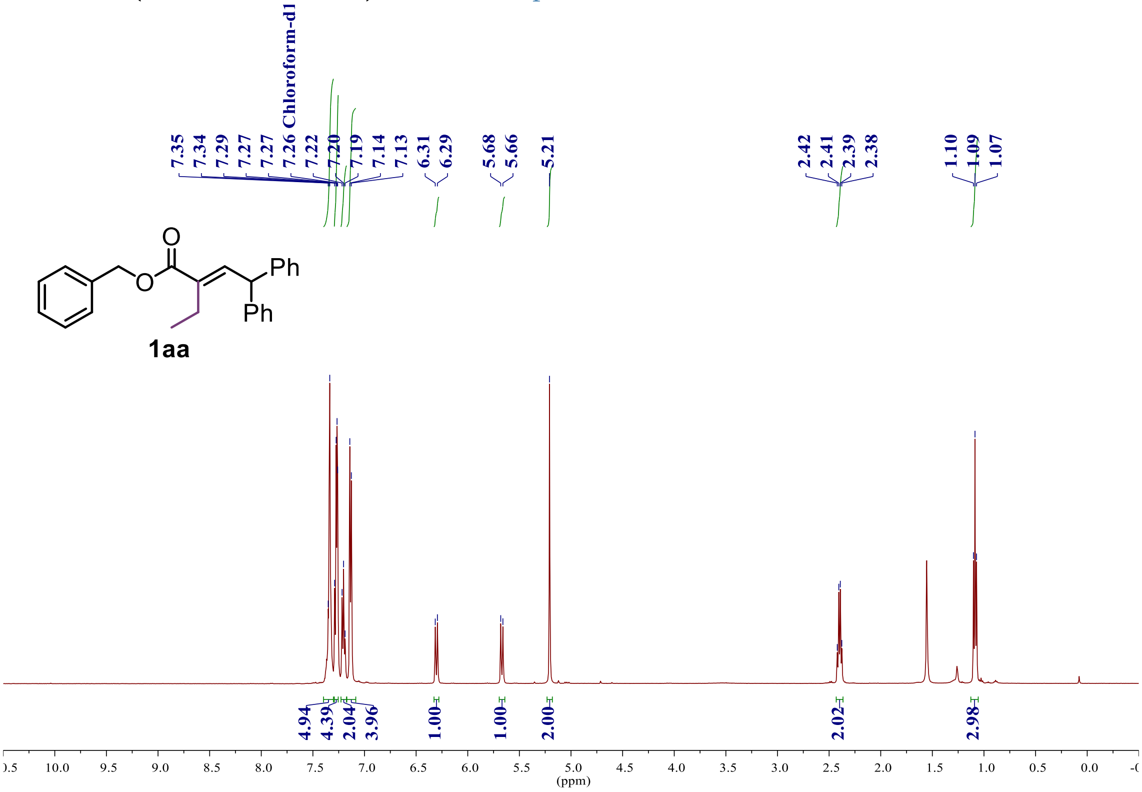

^{13}C{^{1}H} NMR (101 MHz, $CDCl_3$) of **1aa**

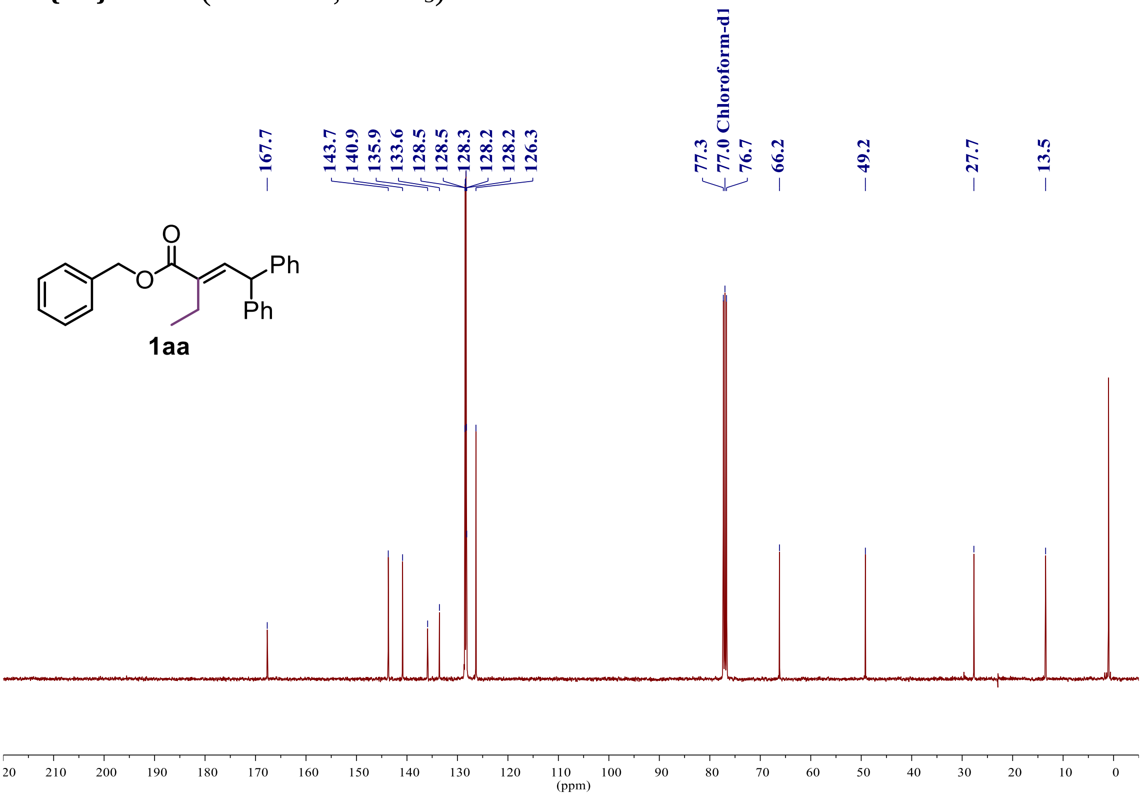

Back to contents

^{1}H NMR (400 MHz, $CDCl_3$) of **1ab**, See procedure

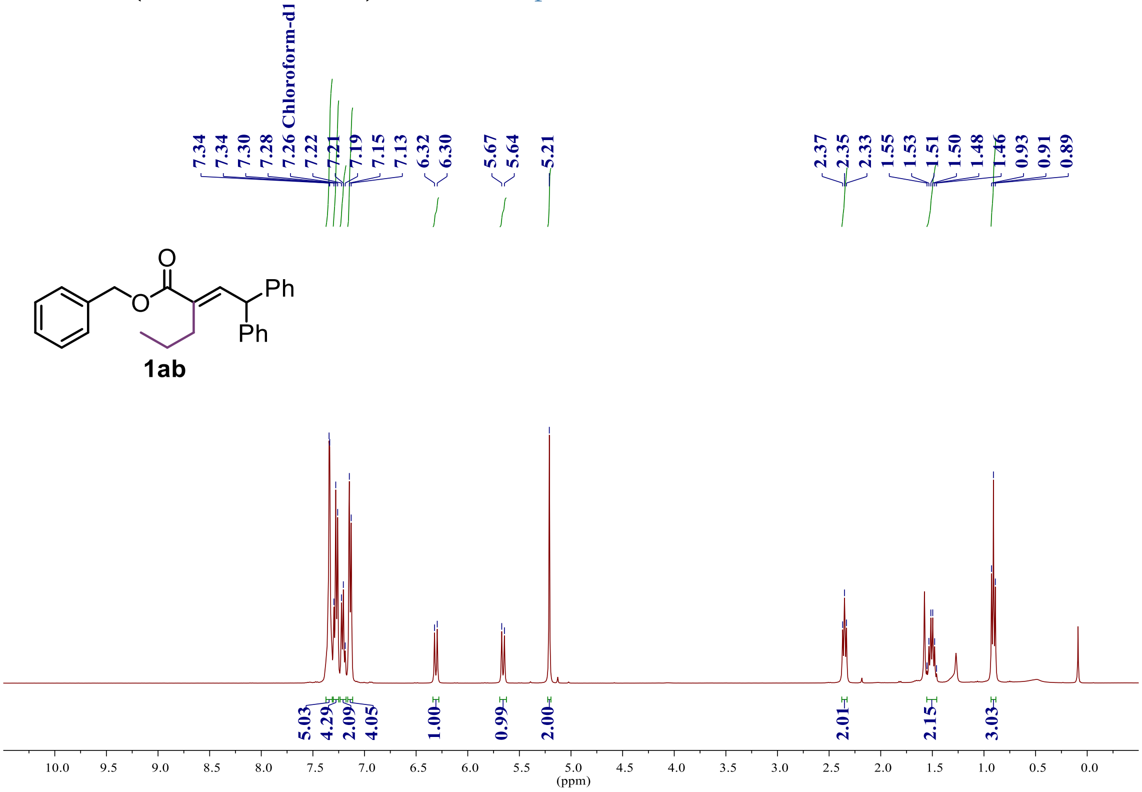

^{13}C{^{1}H} NMR (101 MHz, $CDCl_3$) of **1ab**

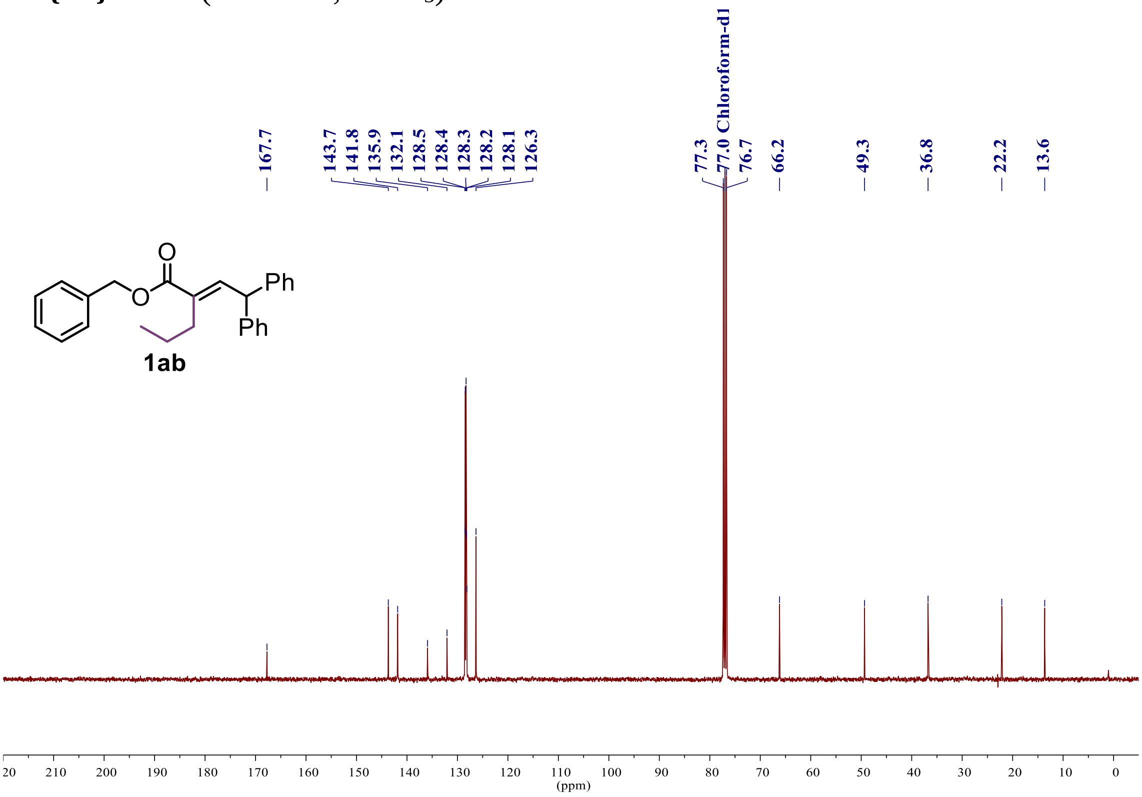

Back to contents

^{1}H NMR (400 MHz, $CDCl_3$) of **1ac**, See procedure

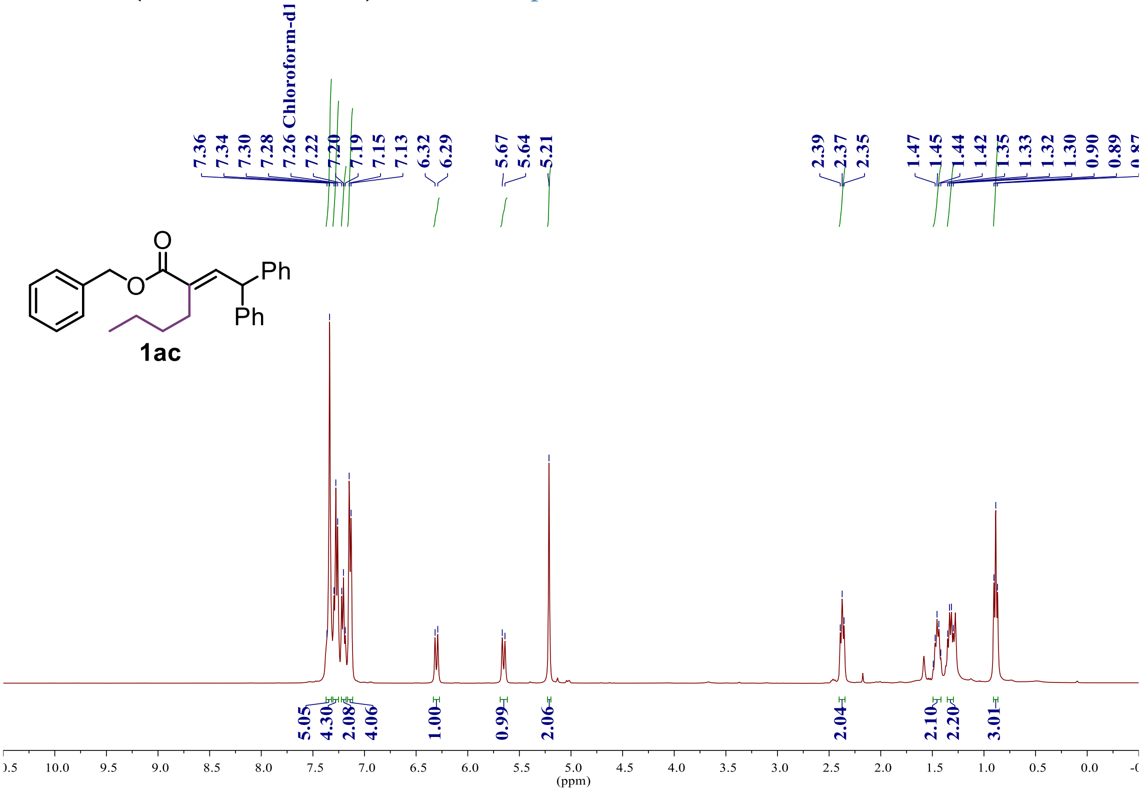

^{13}C{^{1}H} NMR (101 MHz, $CDCl_3$) of **1ac**

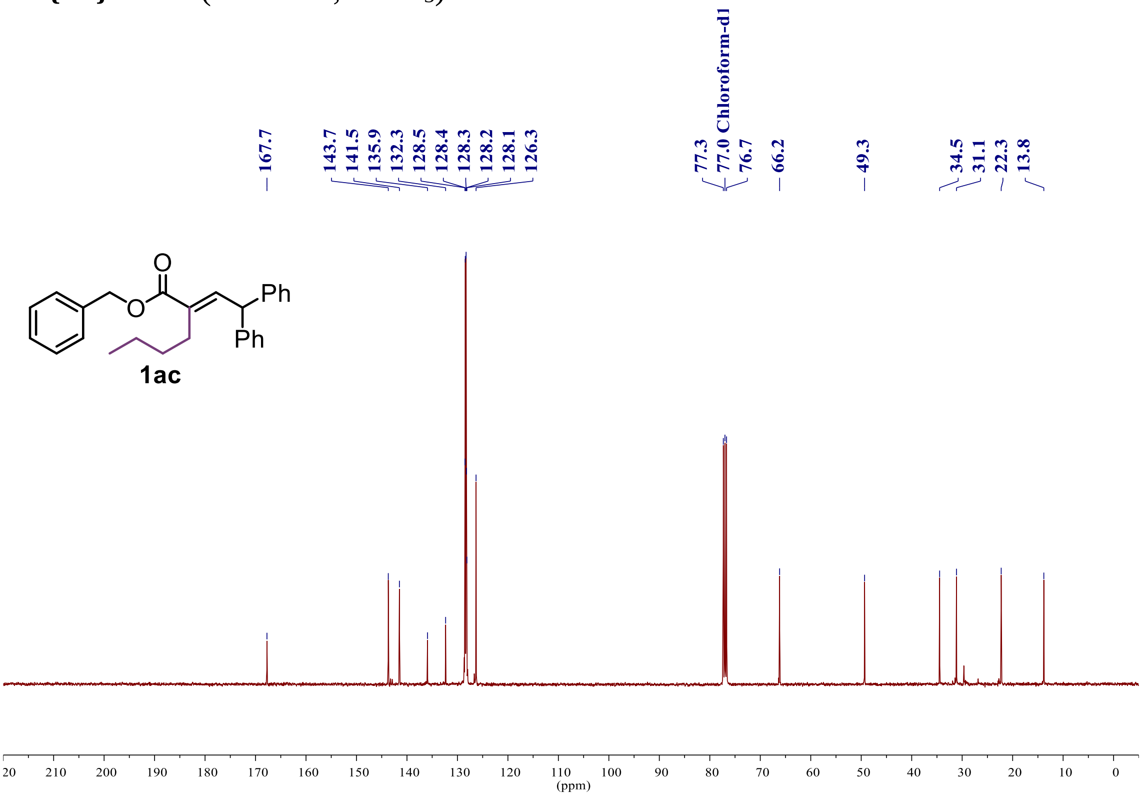

Back to contents

¹H NMR (300 MHz, $CDCl_3$) of **1ad**, See procedure

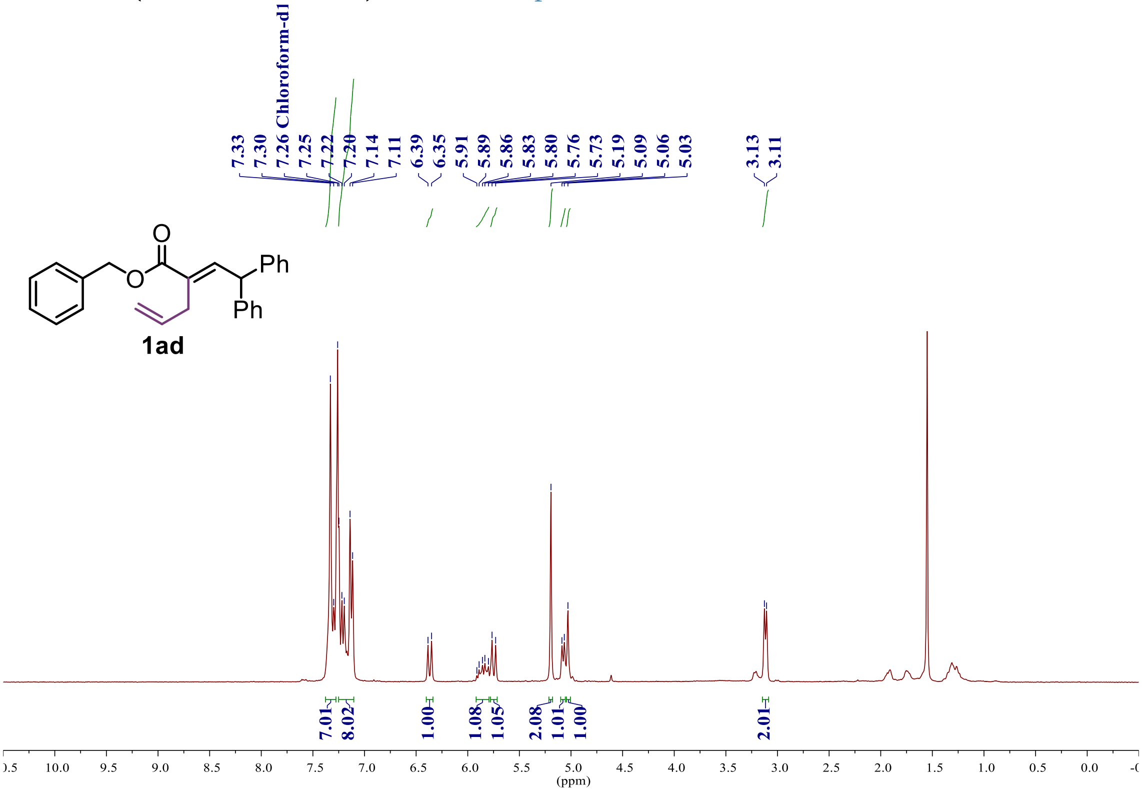

¹³C{¹H} NMR (101 MHz, $CDCl_3$) of **1ad**

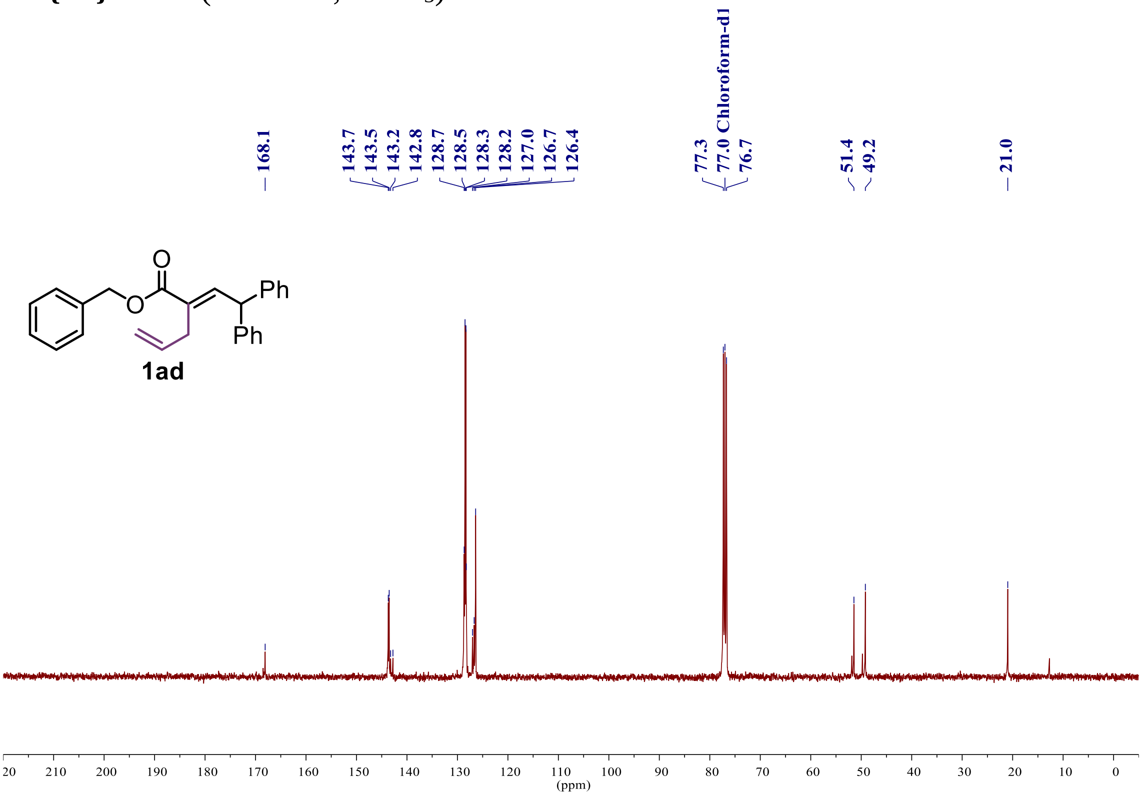

[Back to contents](#)

^{1}H NMR (400 MHz, $CDCl_3$) of **1ae**, [See procedure](#)

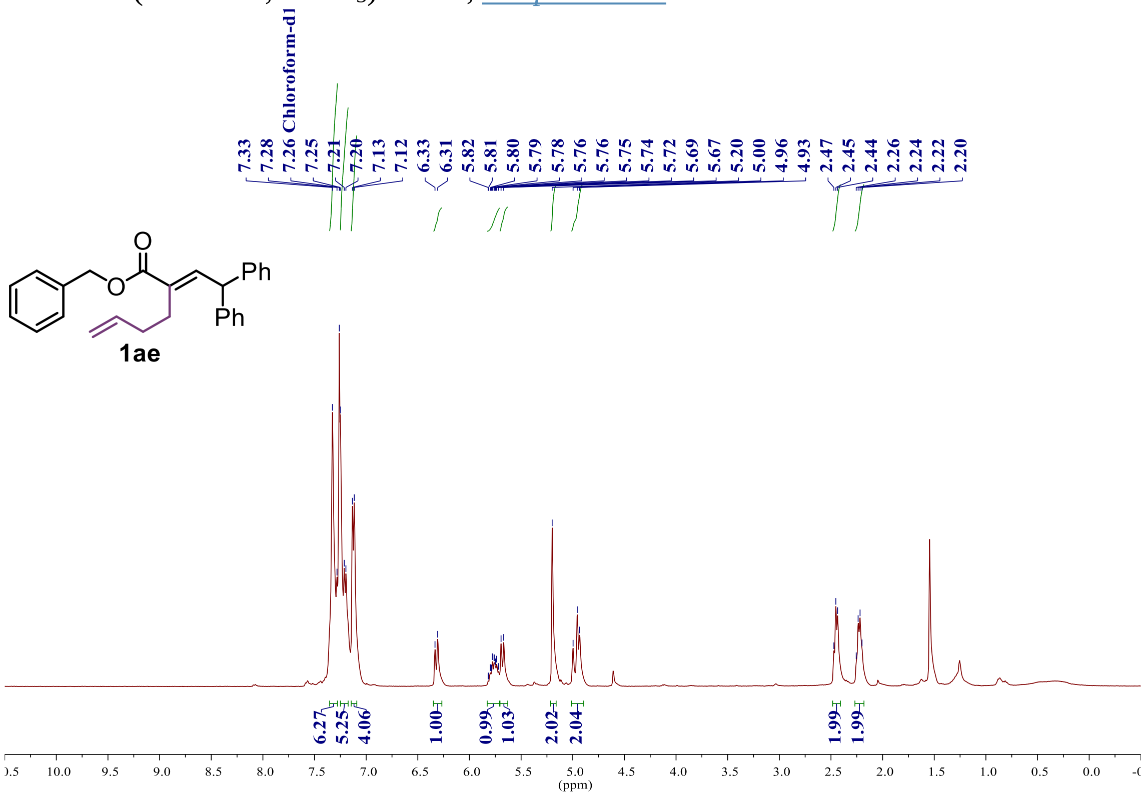

^{13}C{^{1}H} NMR (101 MHz, $CDCl_3$) of **1ae**

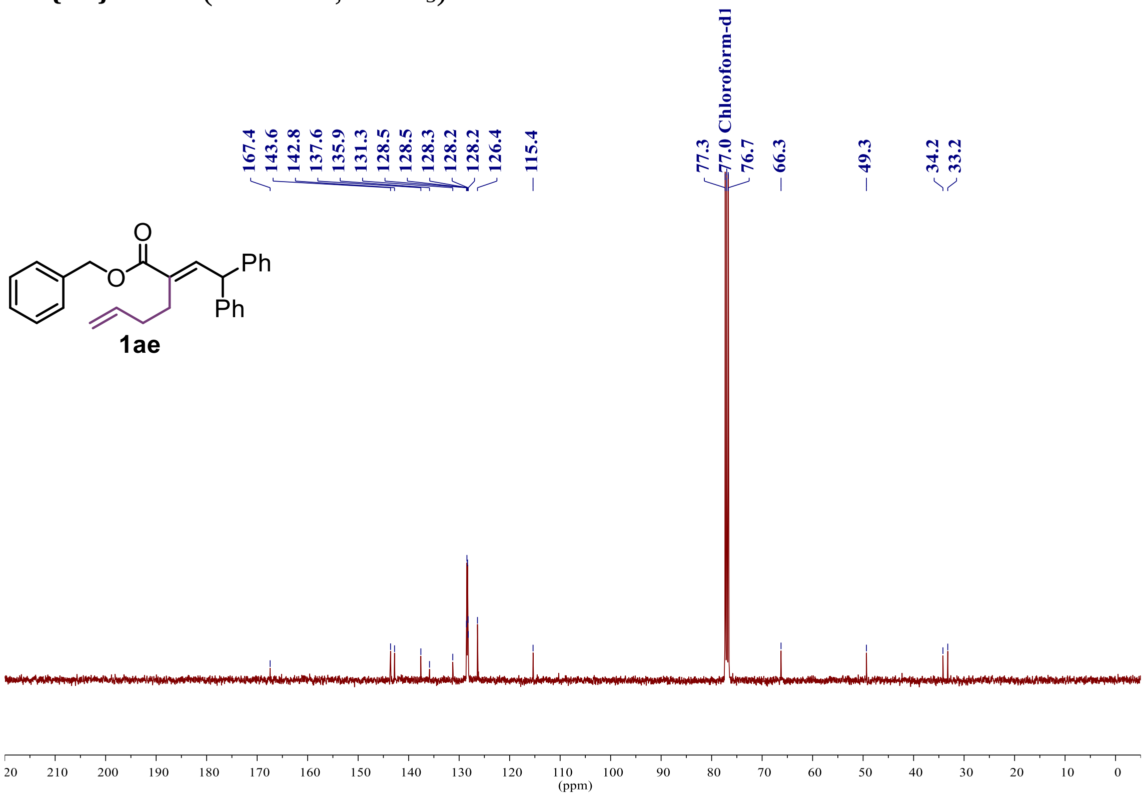

^{1}H NMR (600 MHz, $CDCl_3$) of **1af**, See procedure

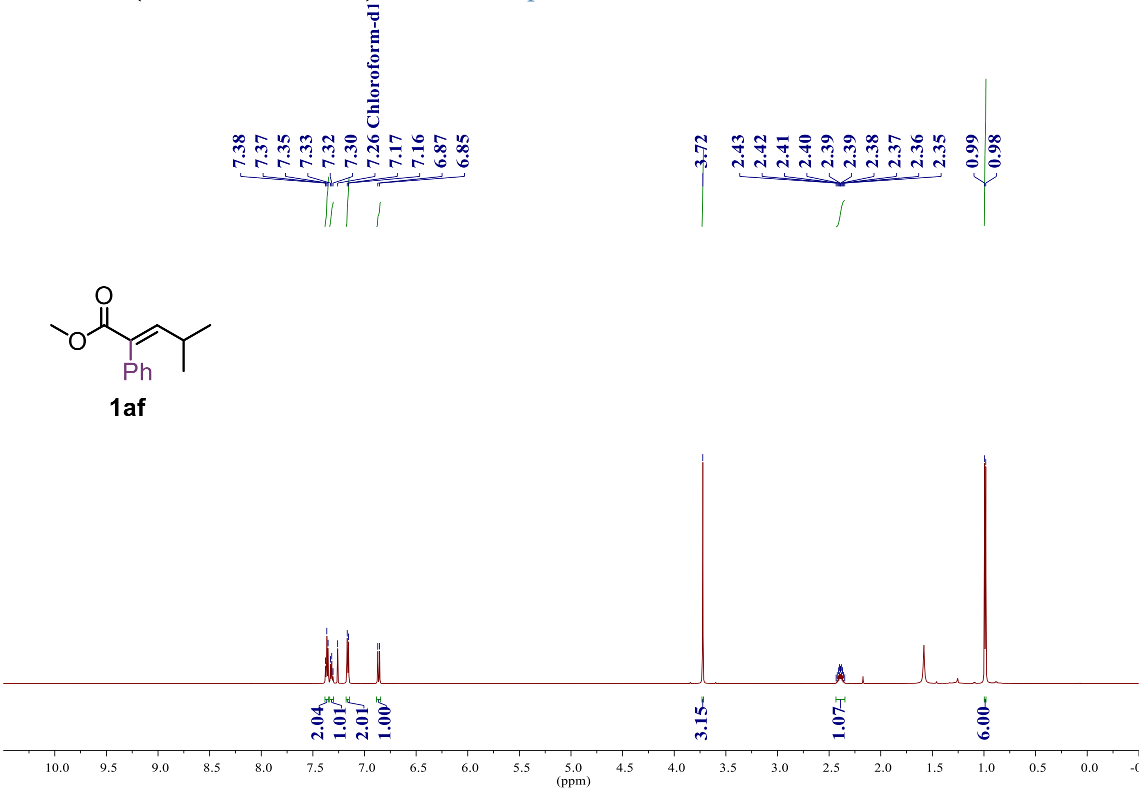

^{13}C{^{1}H} NMR (151 MHz, $CDCl_3$) of **1af**

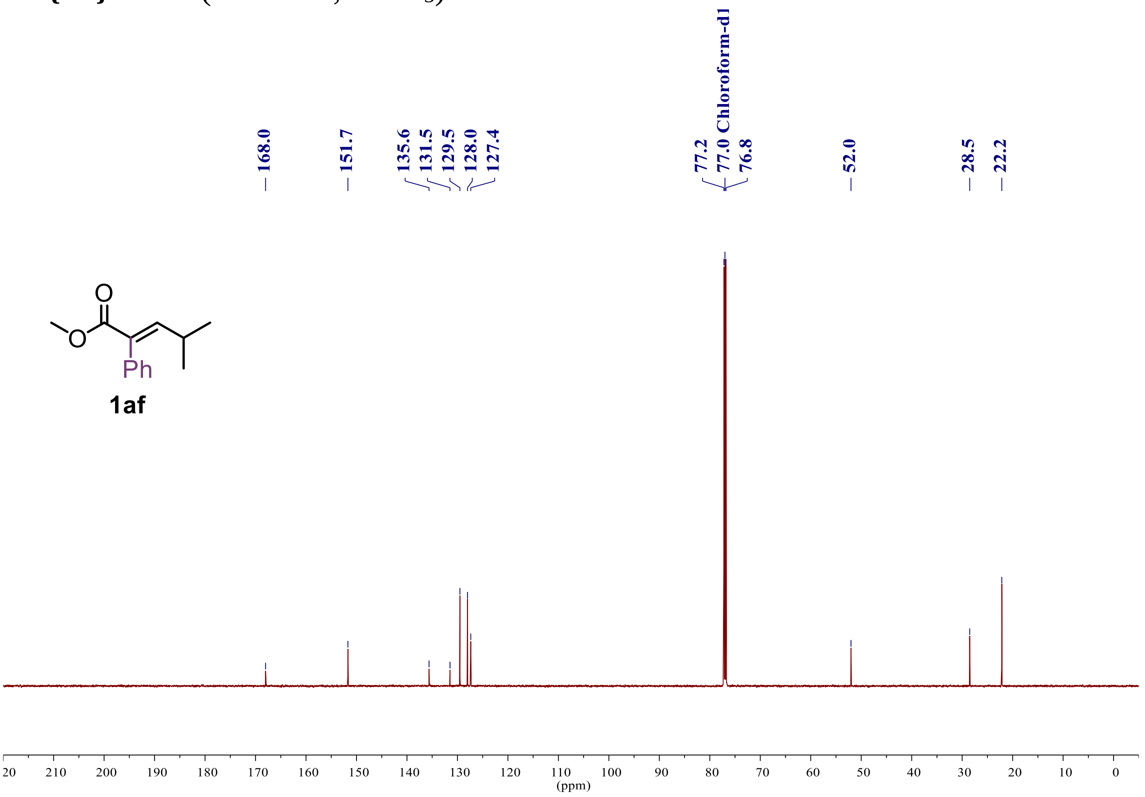

Back to contents

^{1}H NMR (600 MHz, $CDCl_3$) of (*Z*)-**1af**, See procedure

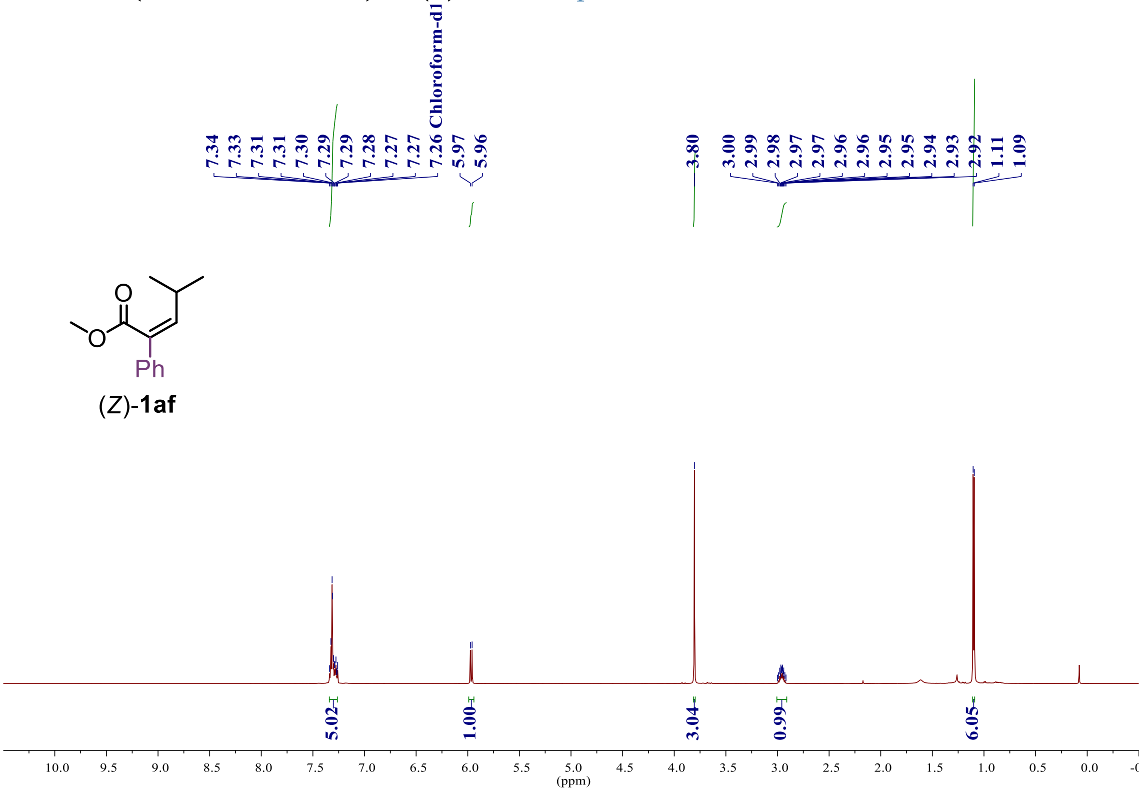

^{13}C{^{1}H} NMR (151 MHz, $CDCl_3$) of (*Z*)-**1af**

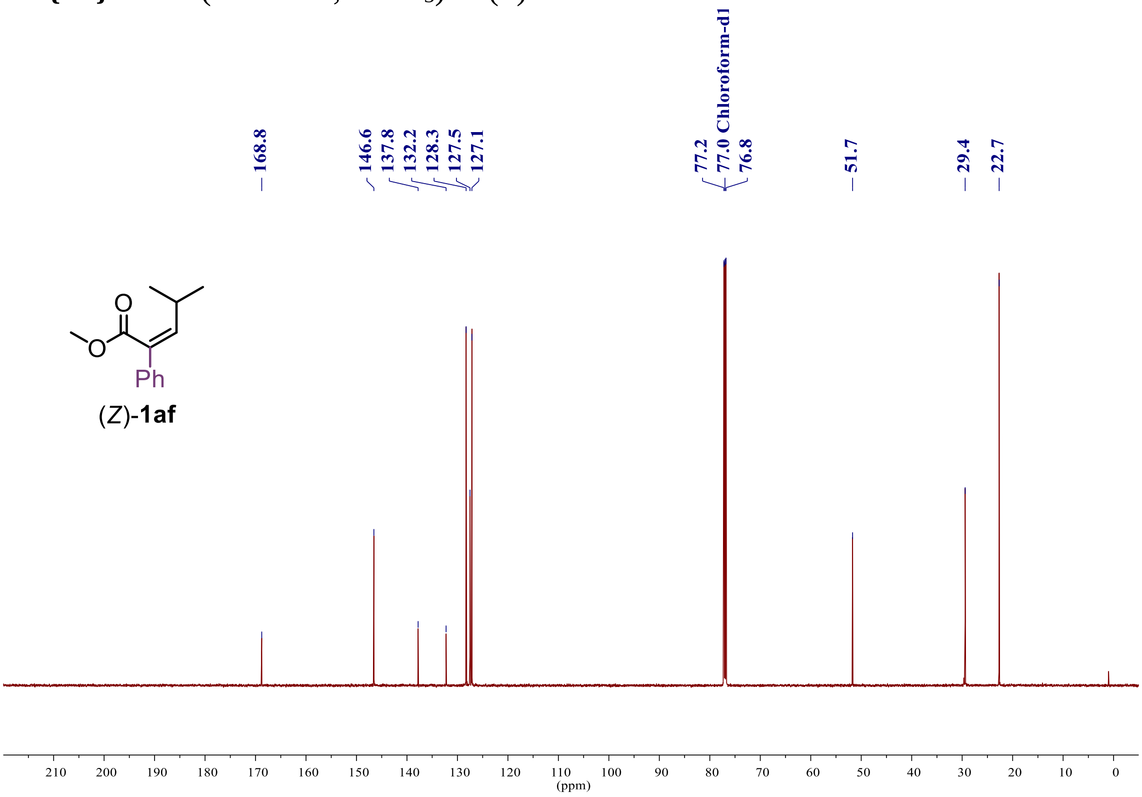

^{1}H NMR (600 MHz, $CDCl_3$) of **1ag**, See procedure

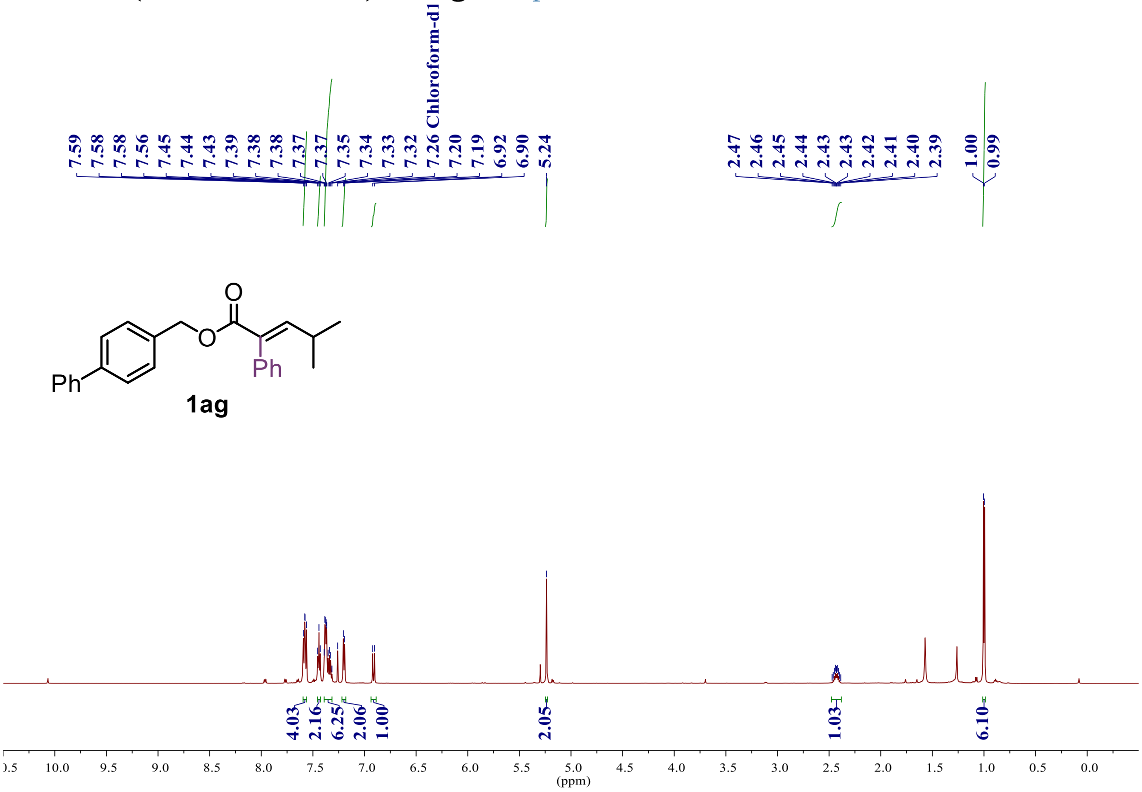

^{13}C{^{1}H} NMR (151 MHz, $CDCl_3$) of **1ag**

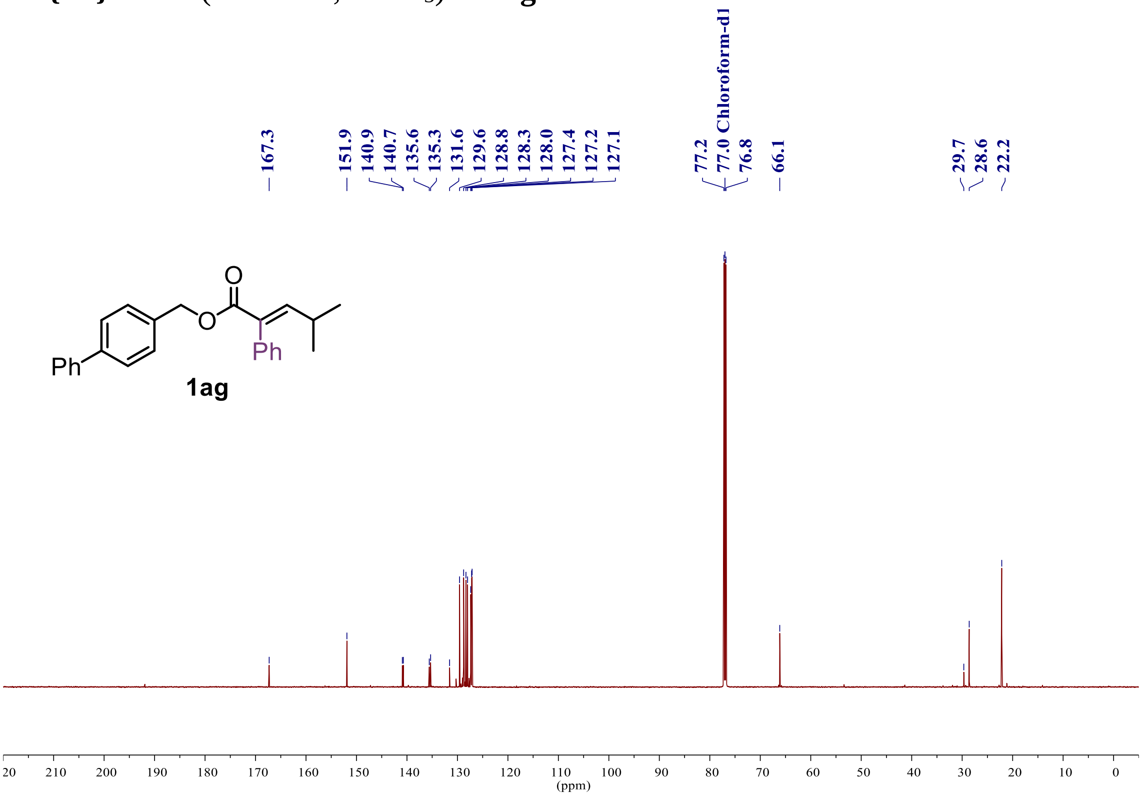

Back to contents

^{1}H NMR (600 MHz, $CDCl_3$) of (*Z*)-**1ag**, See procedure

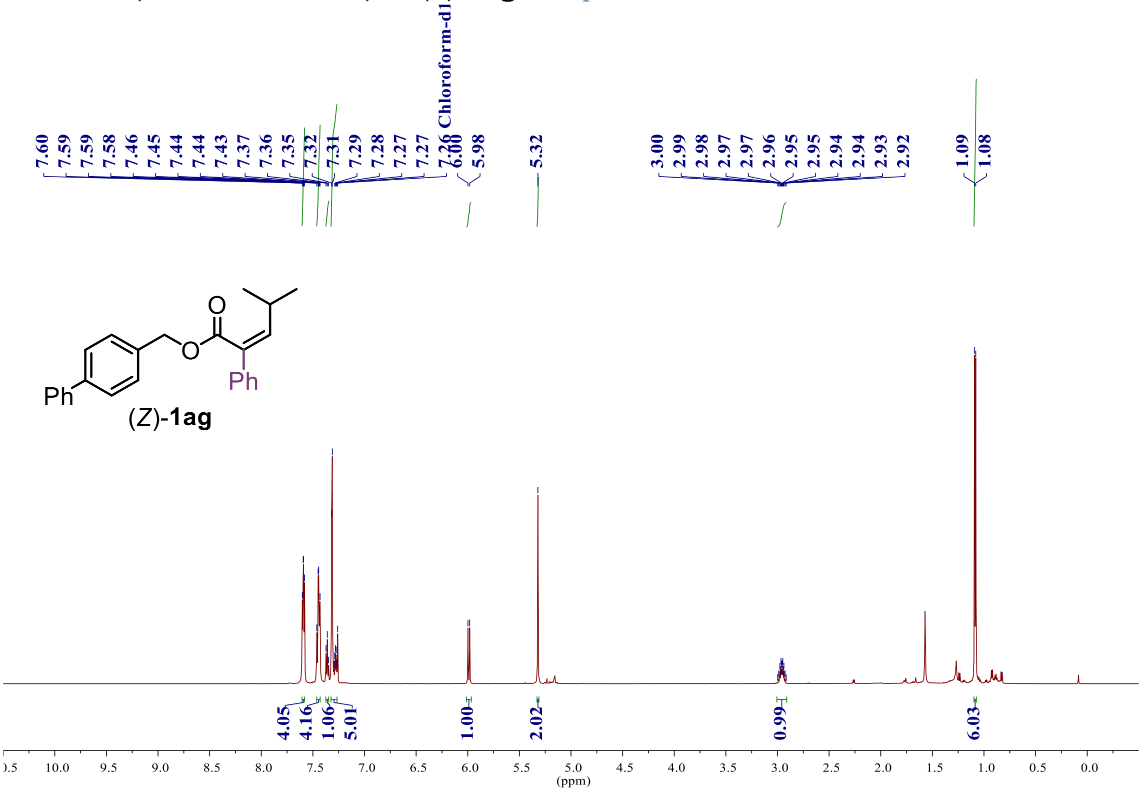

^{13}C{^{1}H} NMR (151 MHz, $CDCl_3$) of (*Z*)-**1ag**

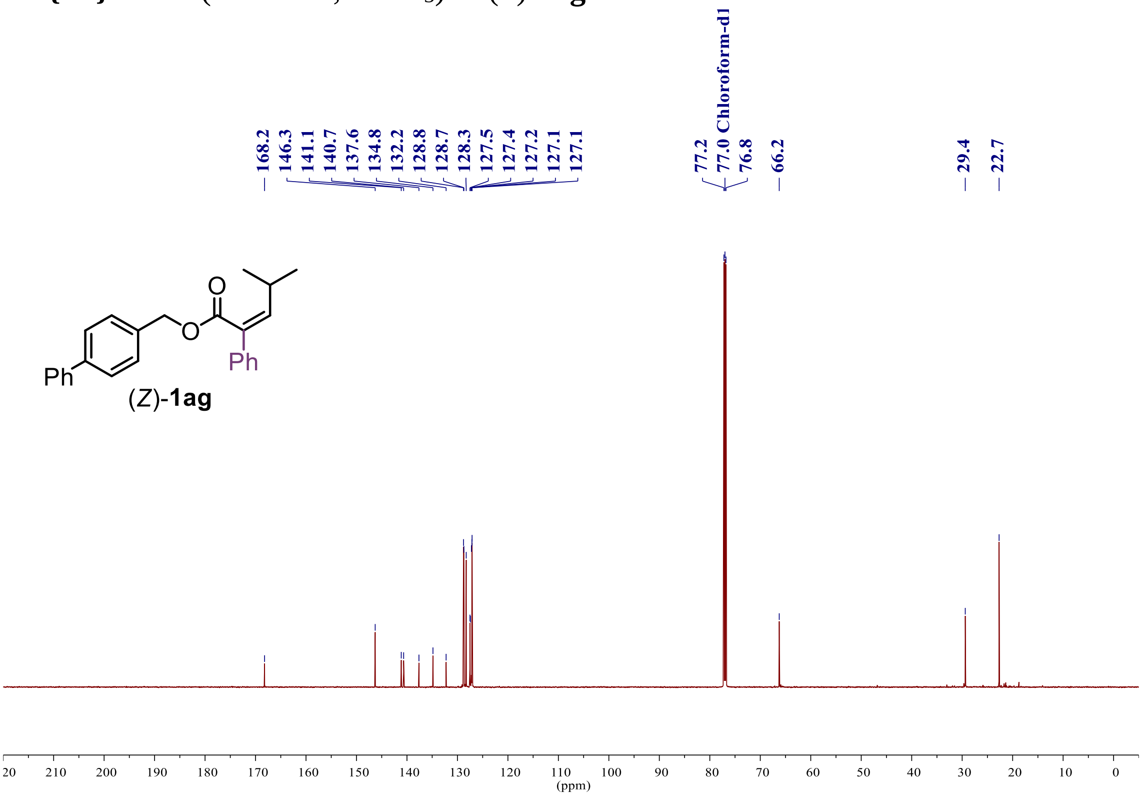

^{1}H NMR (600 MHz, $CDCl_3$) of **1ah**, [See procedure](#)

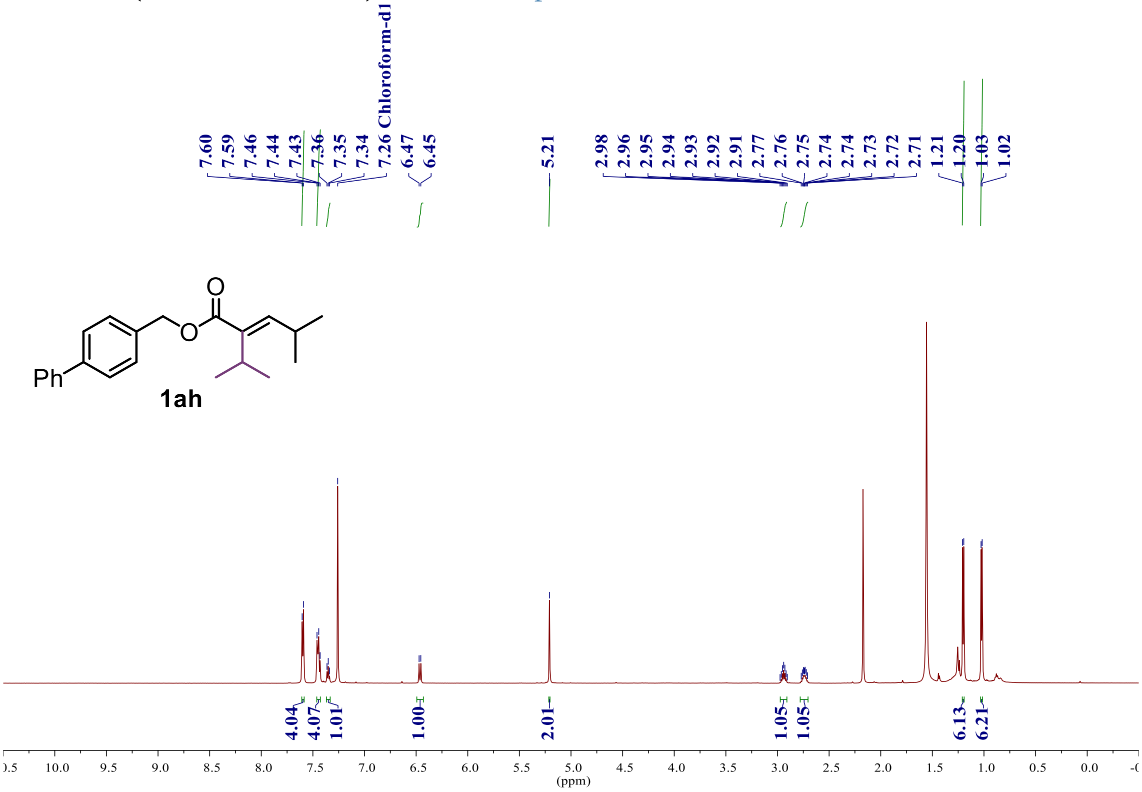

^{13}C{^{1}H} NMR (151 MHz, $CDCl_3$) of **1ah**

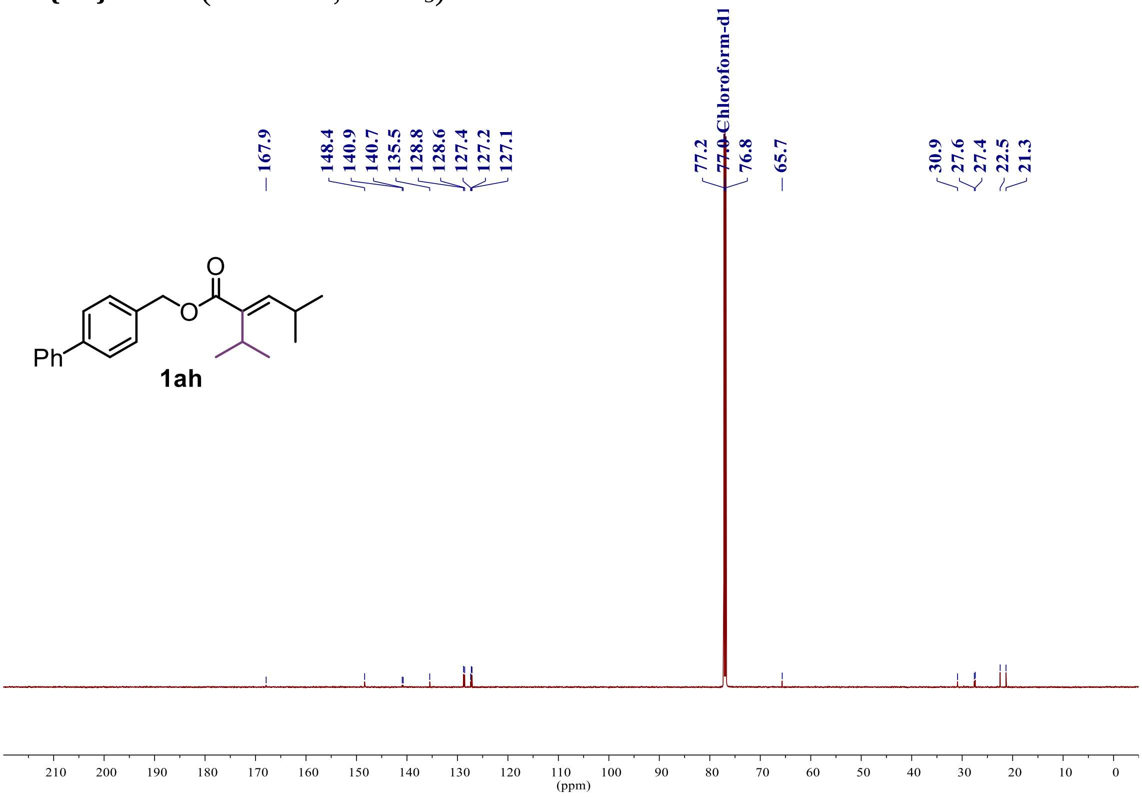

Back to contents

^{1}H NMR (600 MHz, $CDCl_3$) of (*Z*)-**1ah**, See procedure

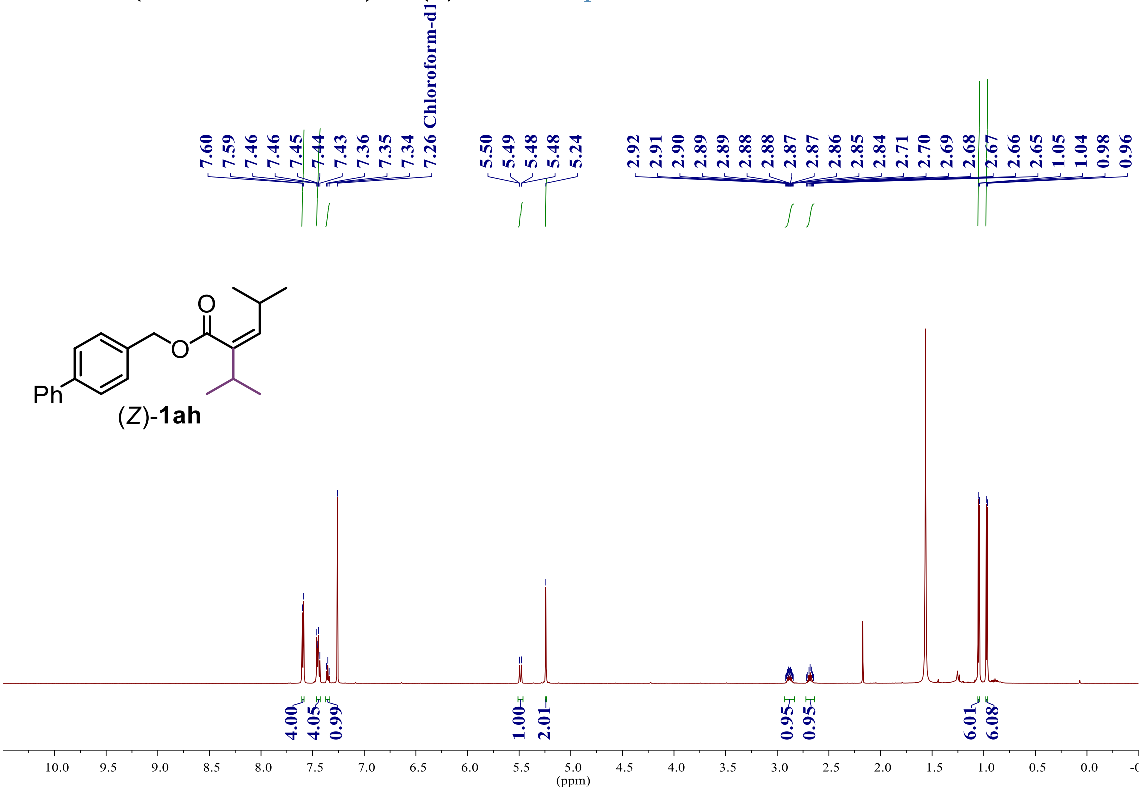

$^{13}C\{^1H\}$ NMR (151 MHz, $CDCl_3$) of (*Z*)-**1ah**

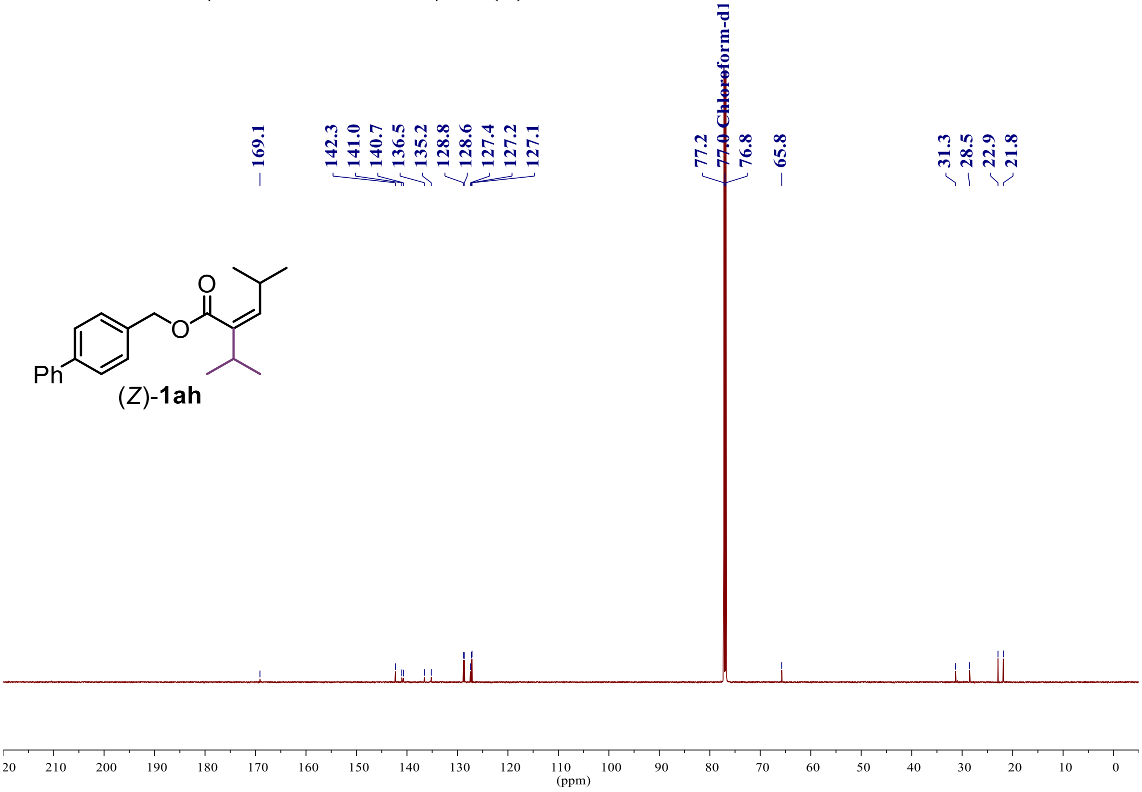

Back to contents

^{1}H NMR (300 MHz, $CDCl_3$) of **1ai**, See procedure

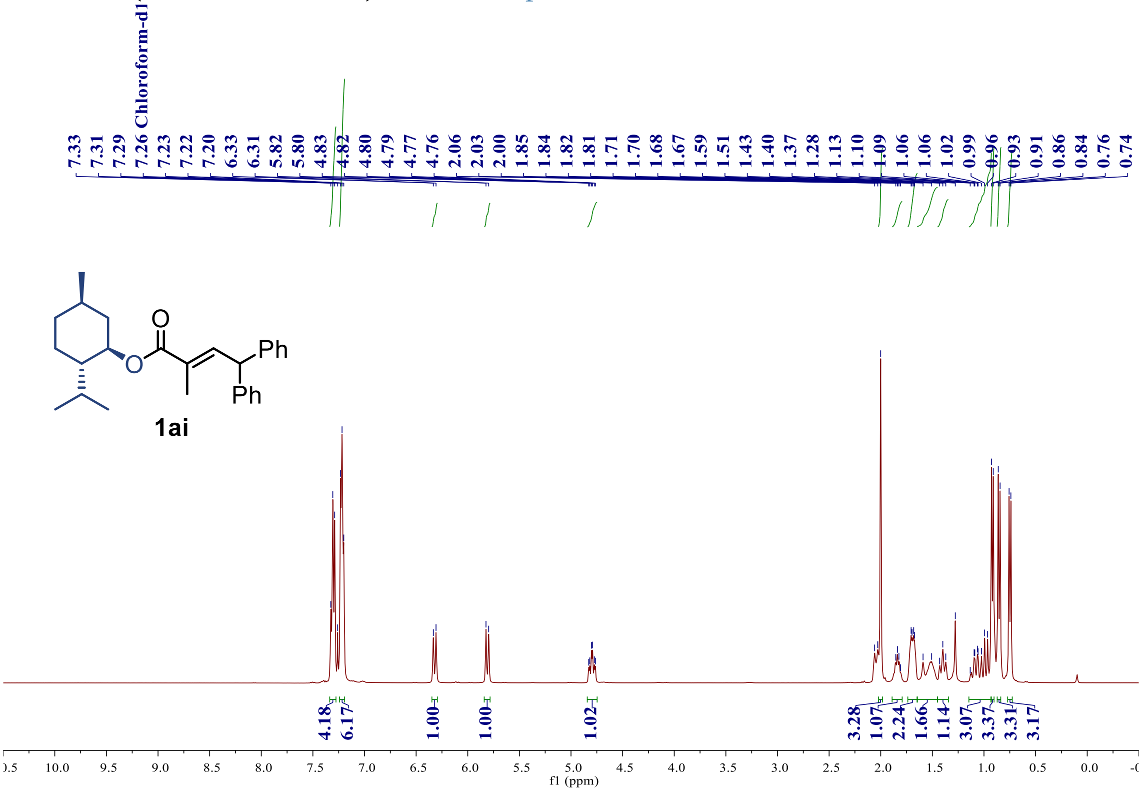

^{13}C{^{1}H} NMR (101 MHz, $CDCl_3$) of **1ai**

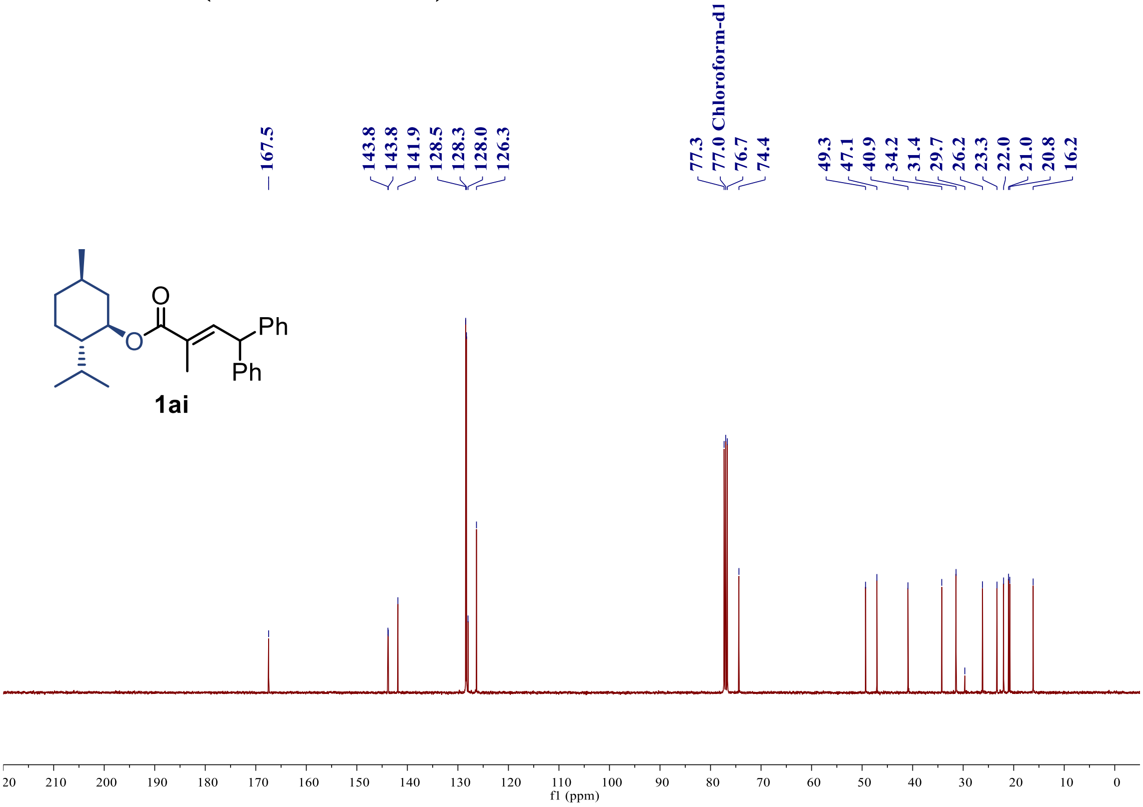

Back to contents

^{1}H NMR (400 MHz, $CDCl_3$) of **1aj**, See procedure

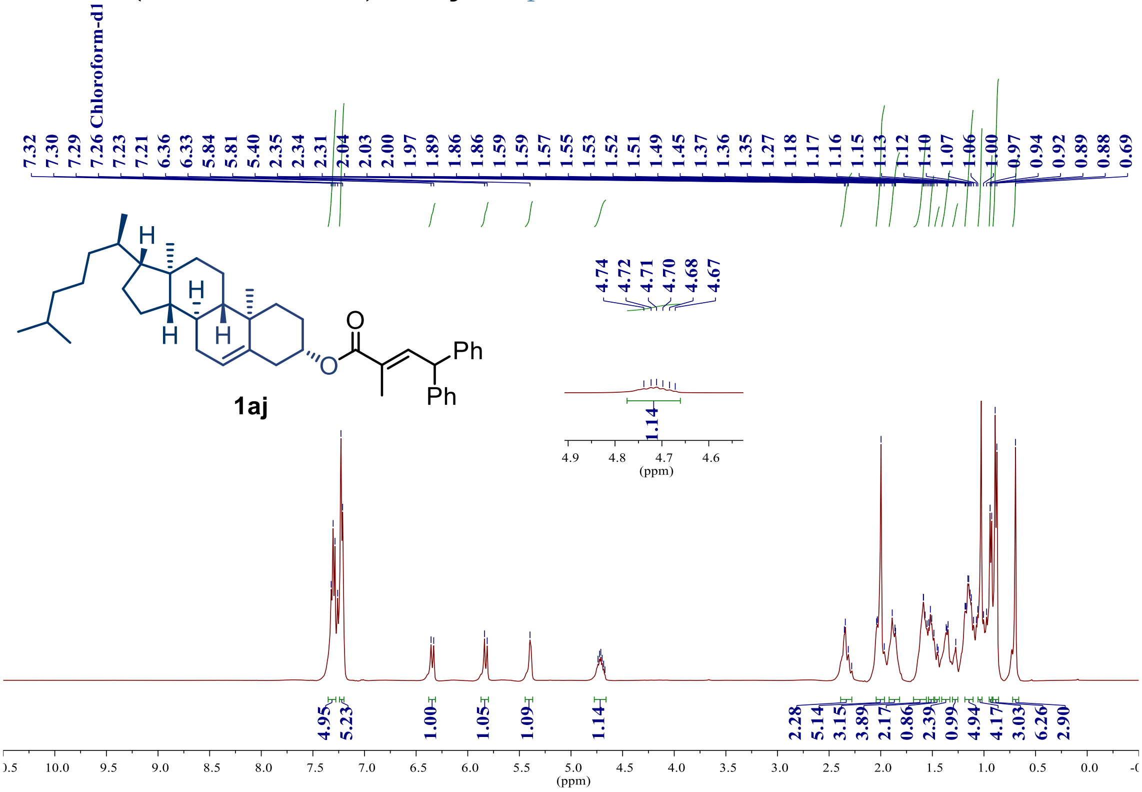

^{13}C{^{1}H} NMR (101 MHz, $CDCl_3$) of **1aj**

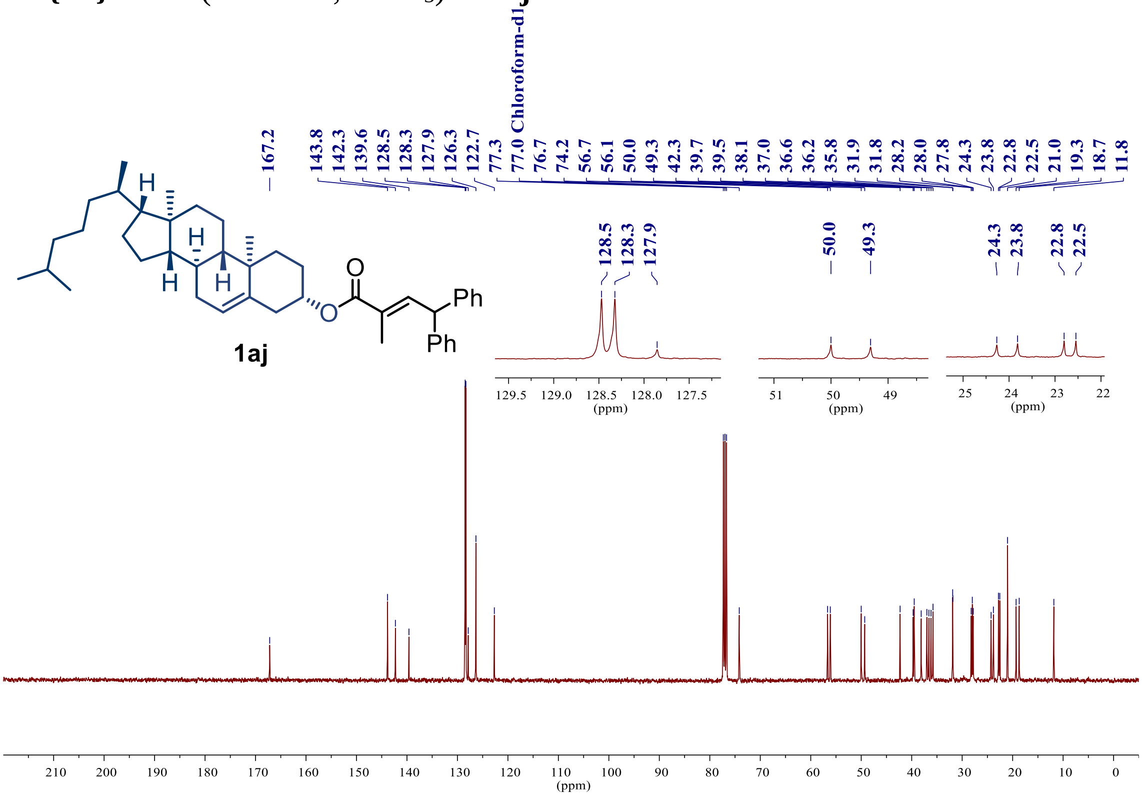

Back to contents

^{1}H NMR (300 MHz, $CDCl_3$) of **1ak**, See procedure

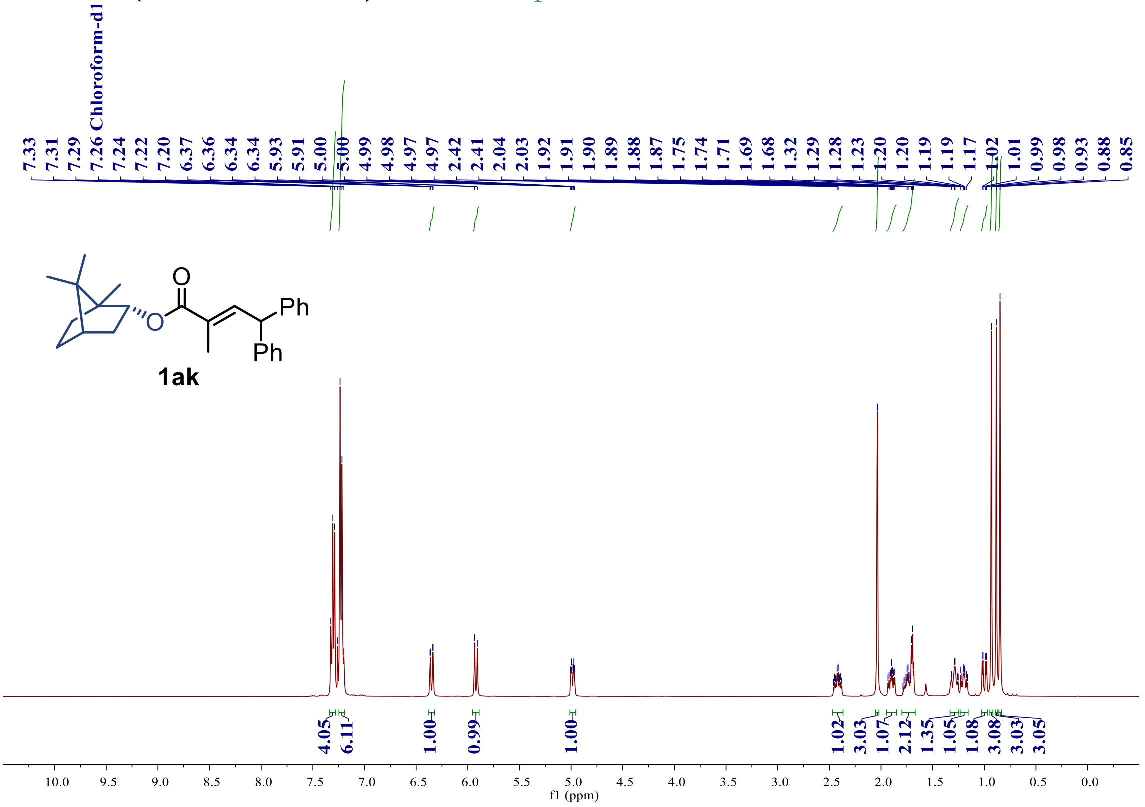

^{13}C{^{1}H} NMR (101 MHz, $CDCl_3$) of **1ak**

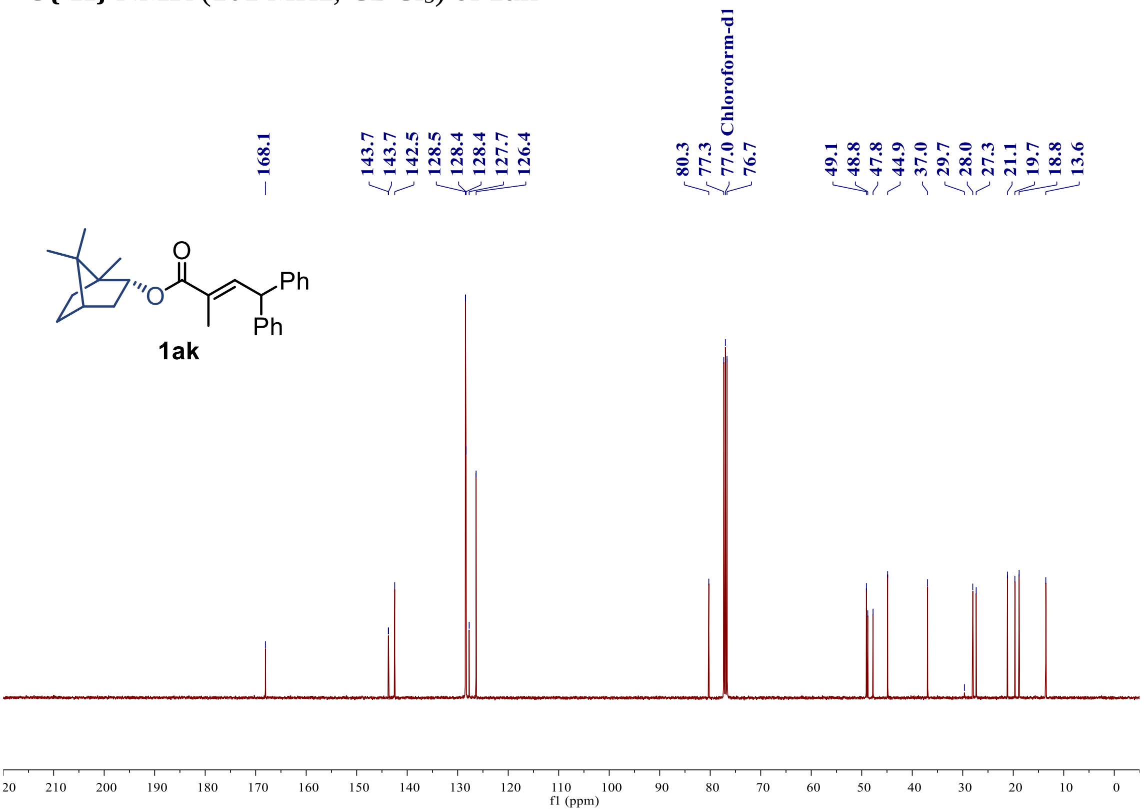

Back to contents

^{1}H NMR (300 MHz, $CDCl_3$) of **1al**, See procedure

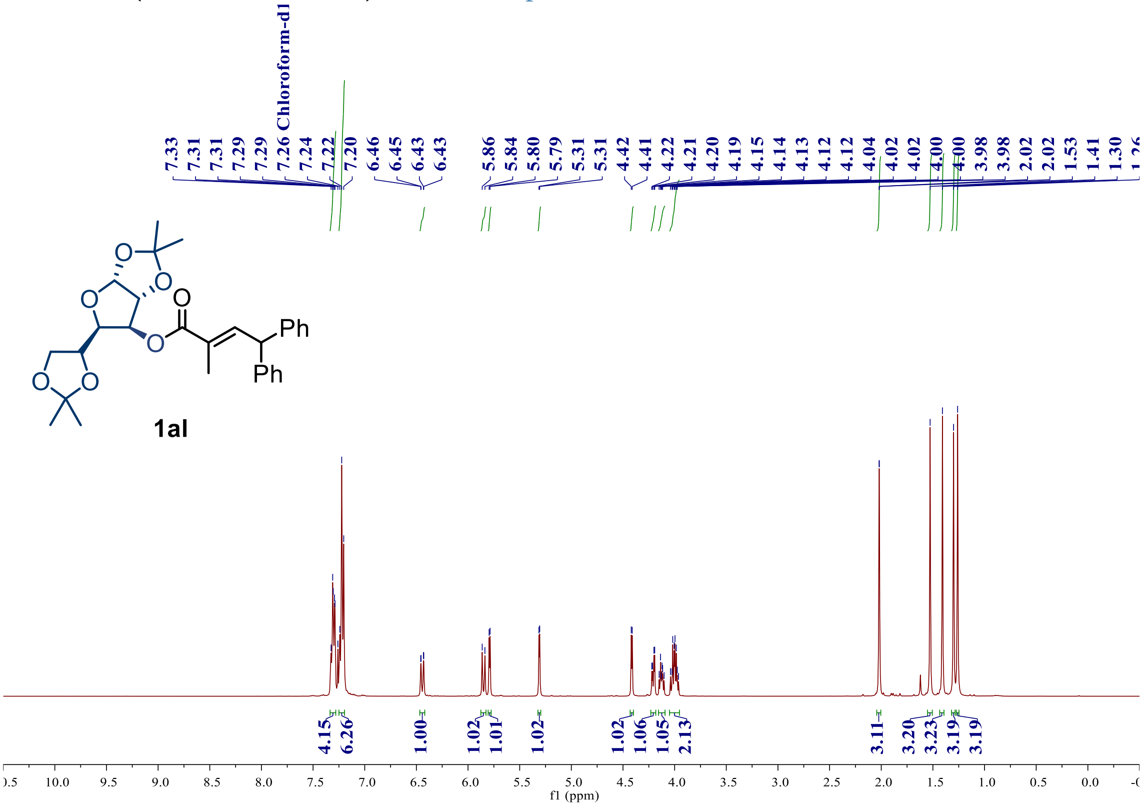

^{13}C{^{1}H} NMR (101 MHz, $CDCl_3$) of **1al**

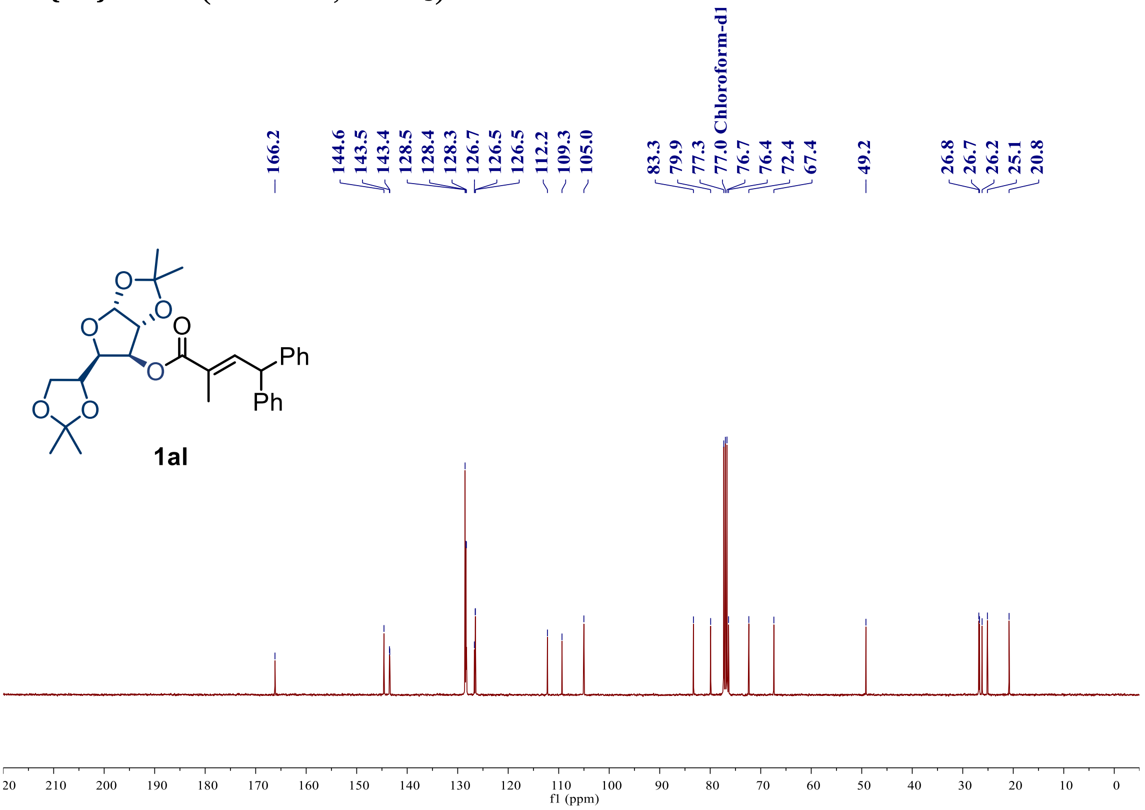

[Back to contents]

^{1}H NMR (400 MHz, $CDCl_3$) of **2a**, See procedure

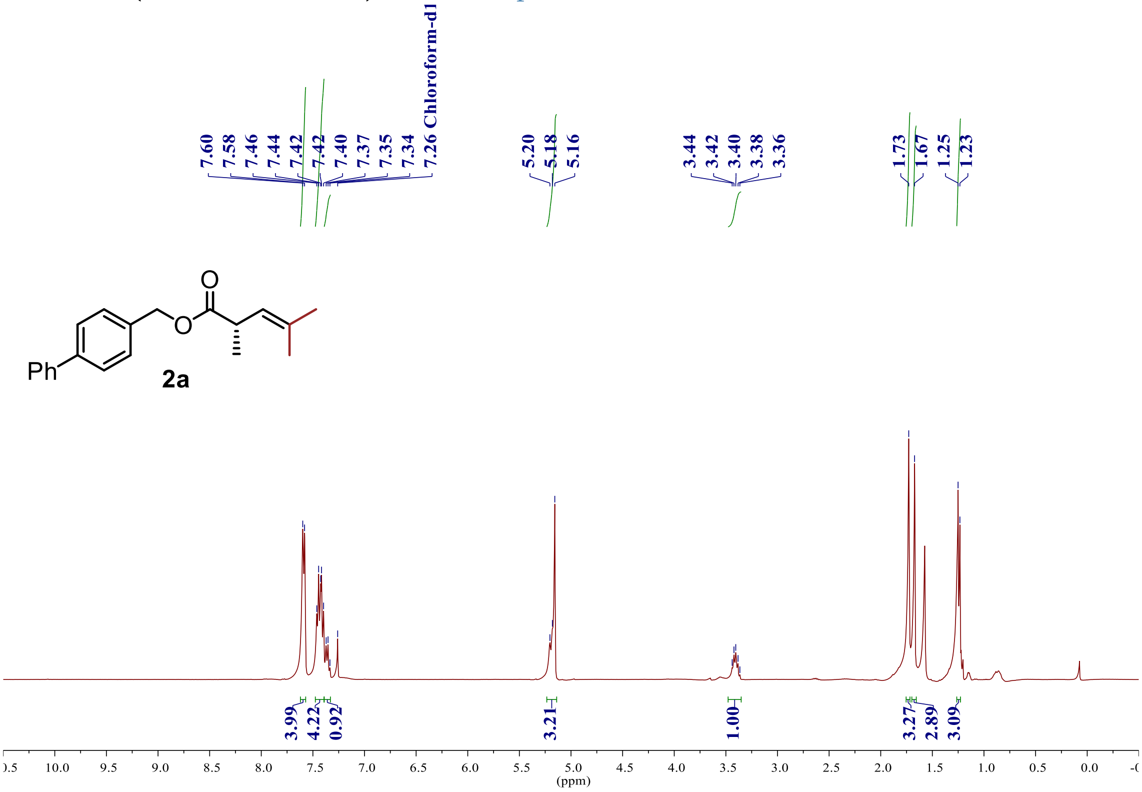

^{13}C{^{1}H} NMR (101 MHz, $CDCl_3$) of **2a**

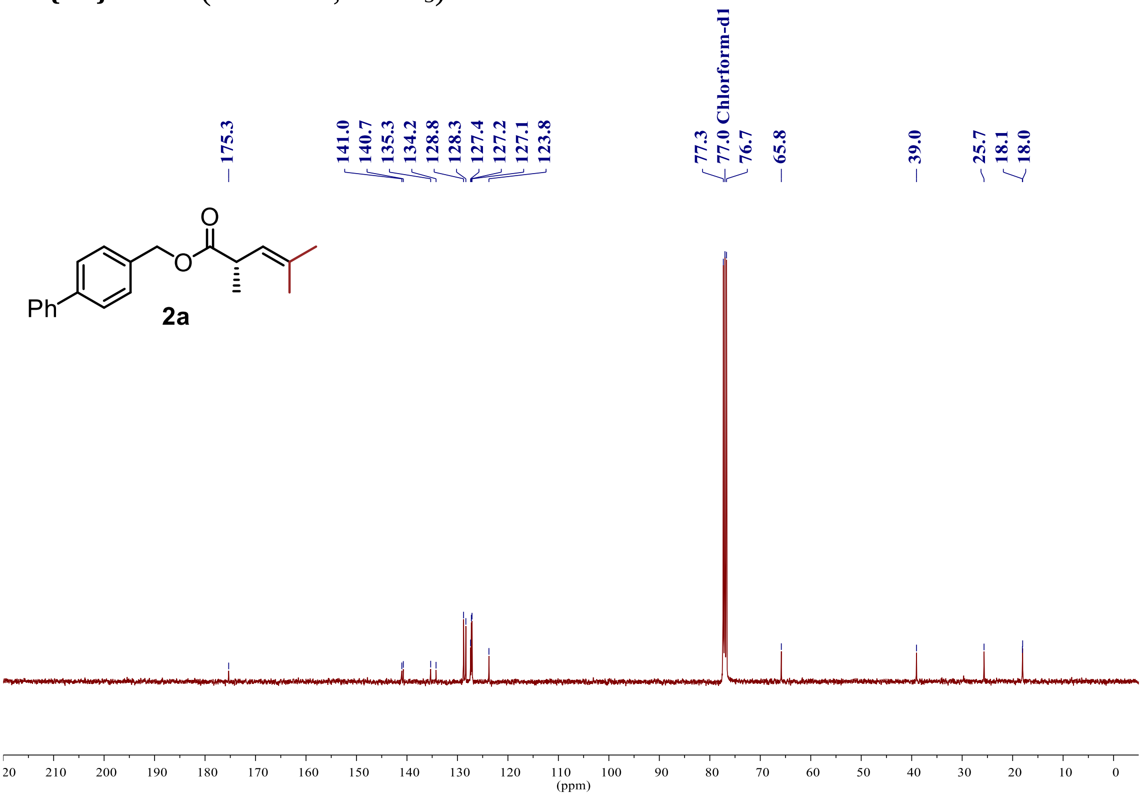

Back to contents

^{1}H NMR (600 MHz, $CDCl_3$) of **2b**, See procedure

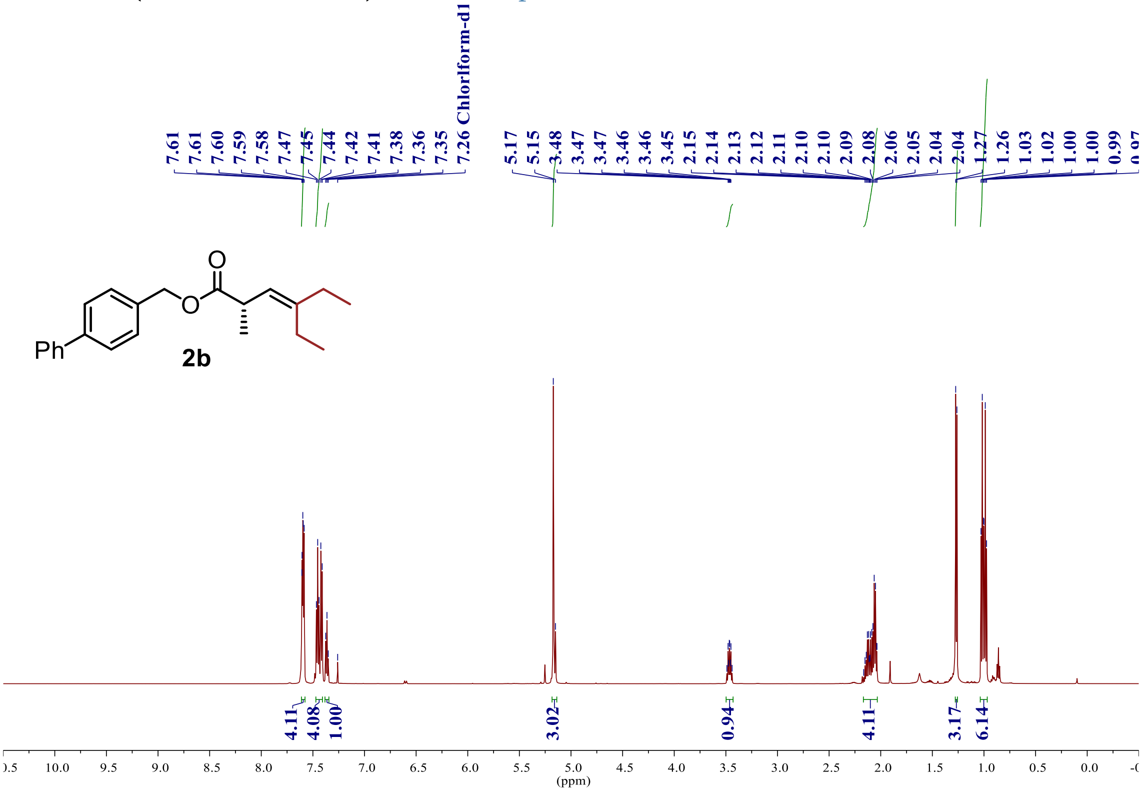

^{13}C{^{1}H} NMR (151 MHz, $CDCl_3$) of **2b**

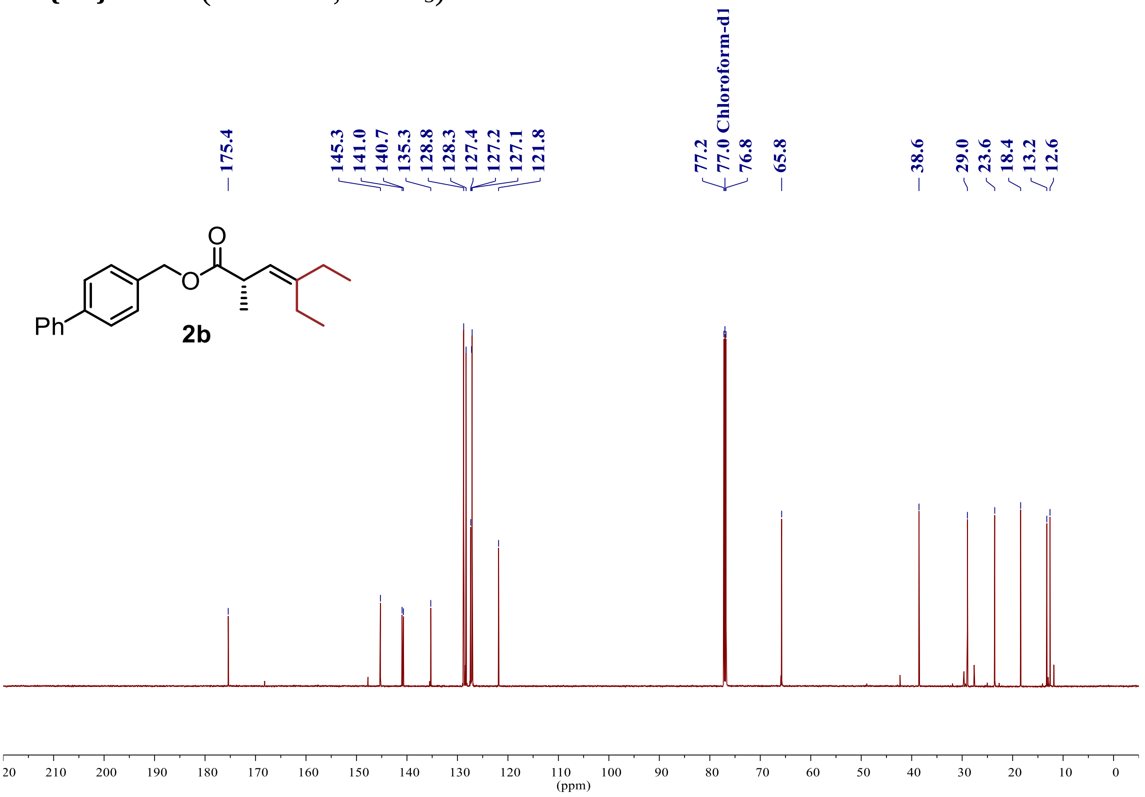

Back to contents

^{1}H NMR (400 MHz, $CDCl_3$) of **2c**, See procedure

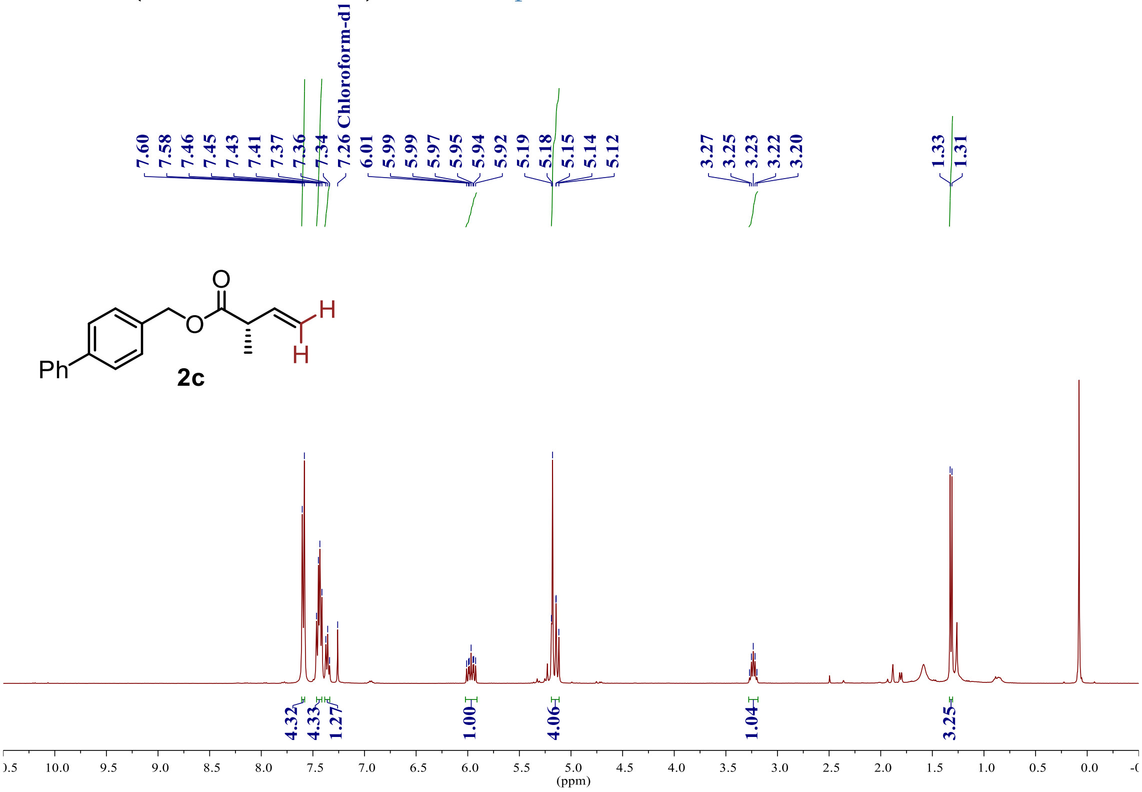

^{13}C{^{1}H} NMR (101 MHz, $CDCl_3$) of **2c**

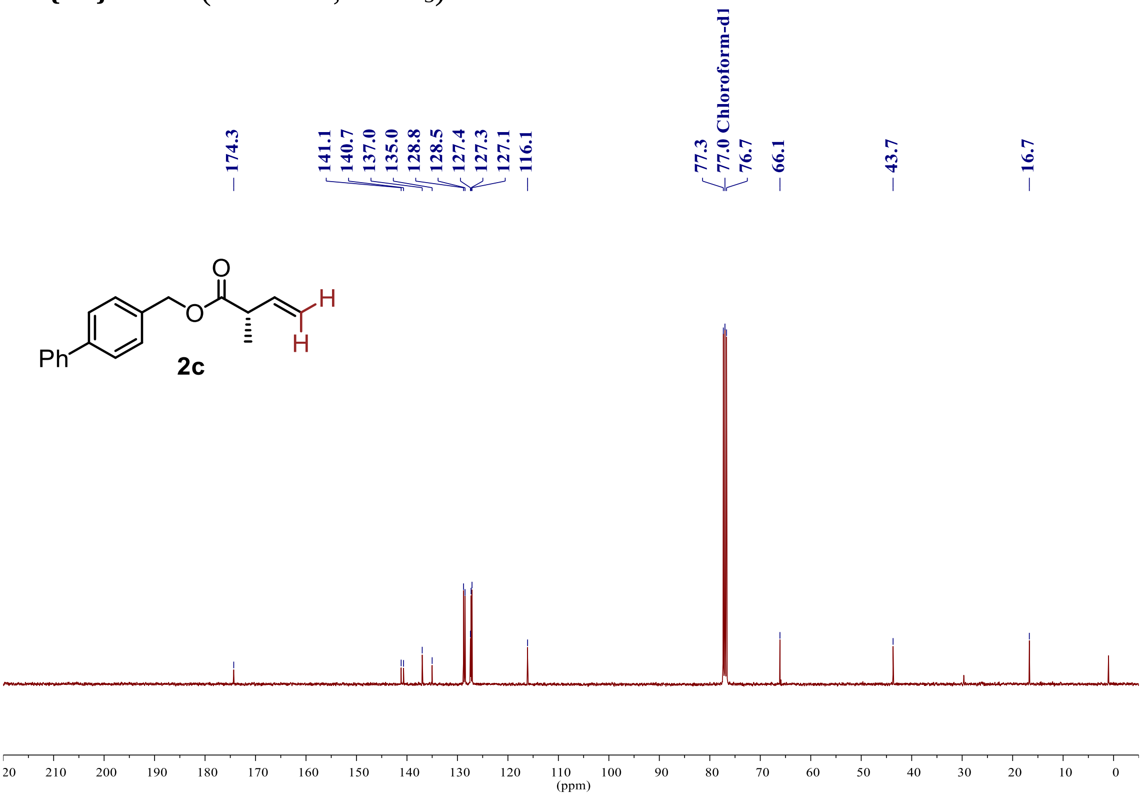

Back to contents

^{1}H NMR (600 MHz, $CDCl_3$) of **2d**, See procedure

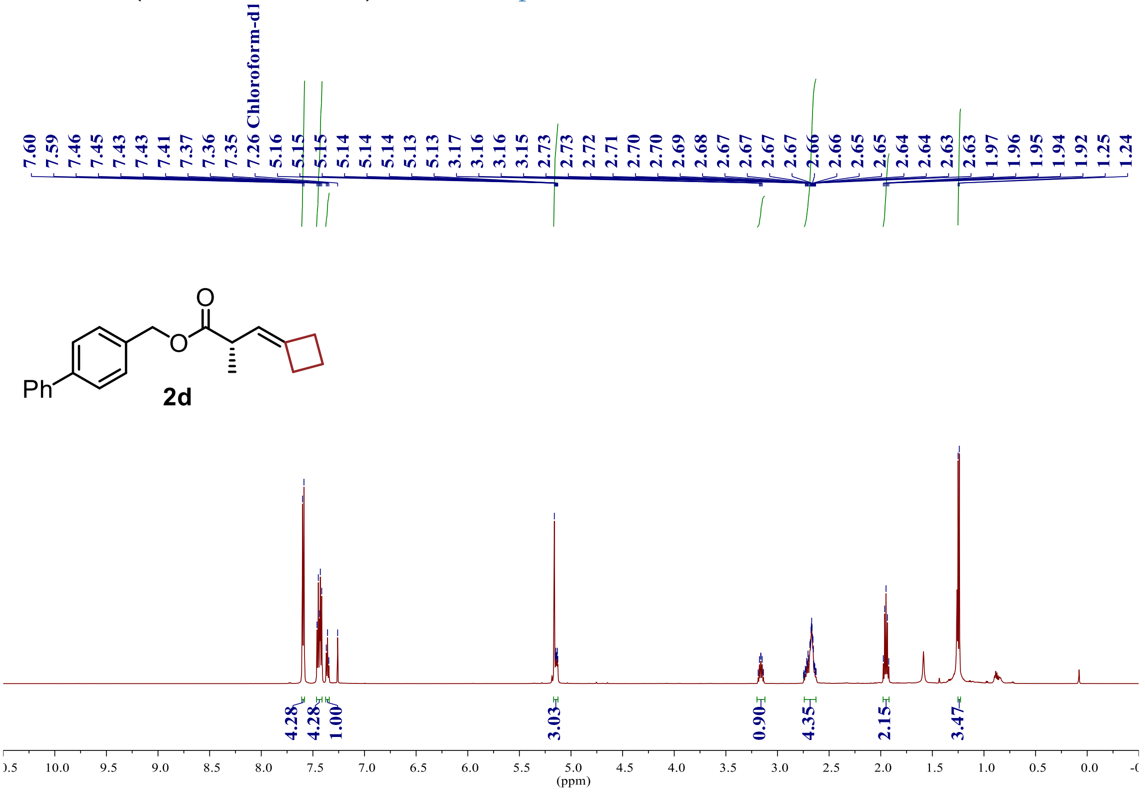

^{13}C{^{1}H} NMR (151 MHz, $CDCl_3$) of **2d**

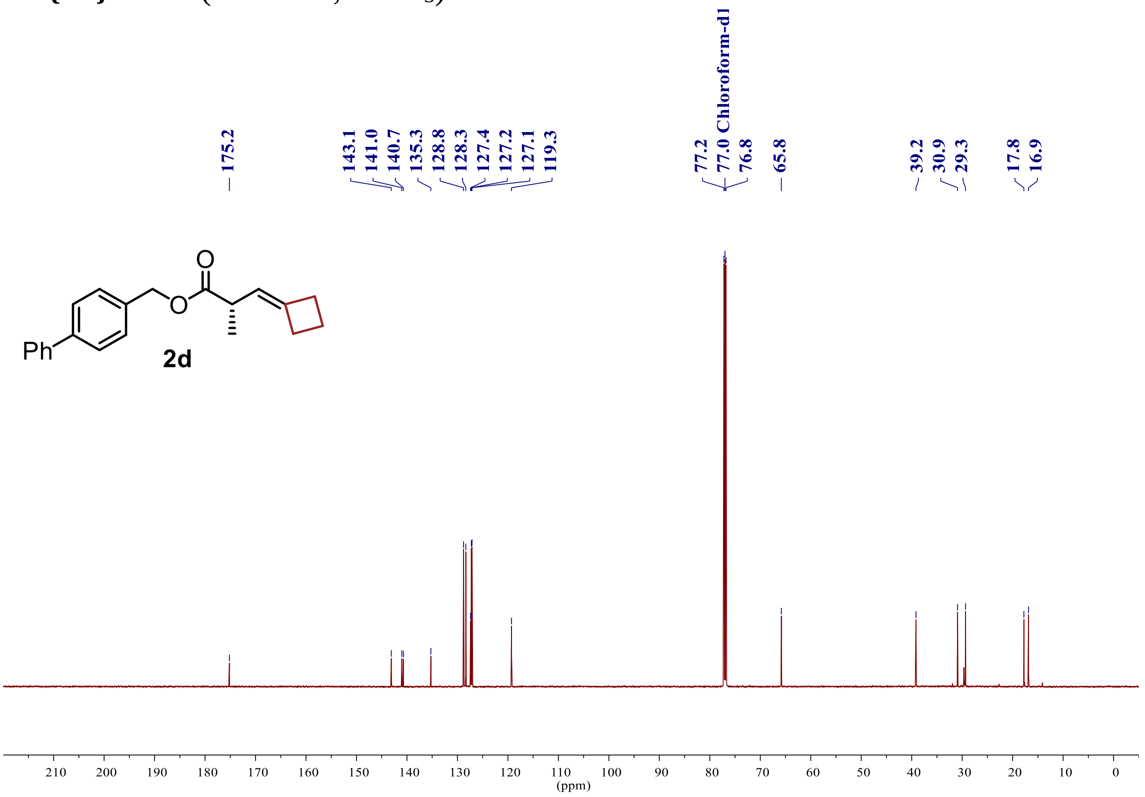

Back to contents

^{1}H NMR (600 MHz, $CDCl_3$) of **2e**, See procedure

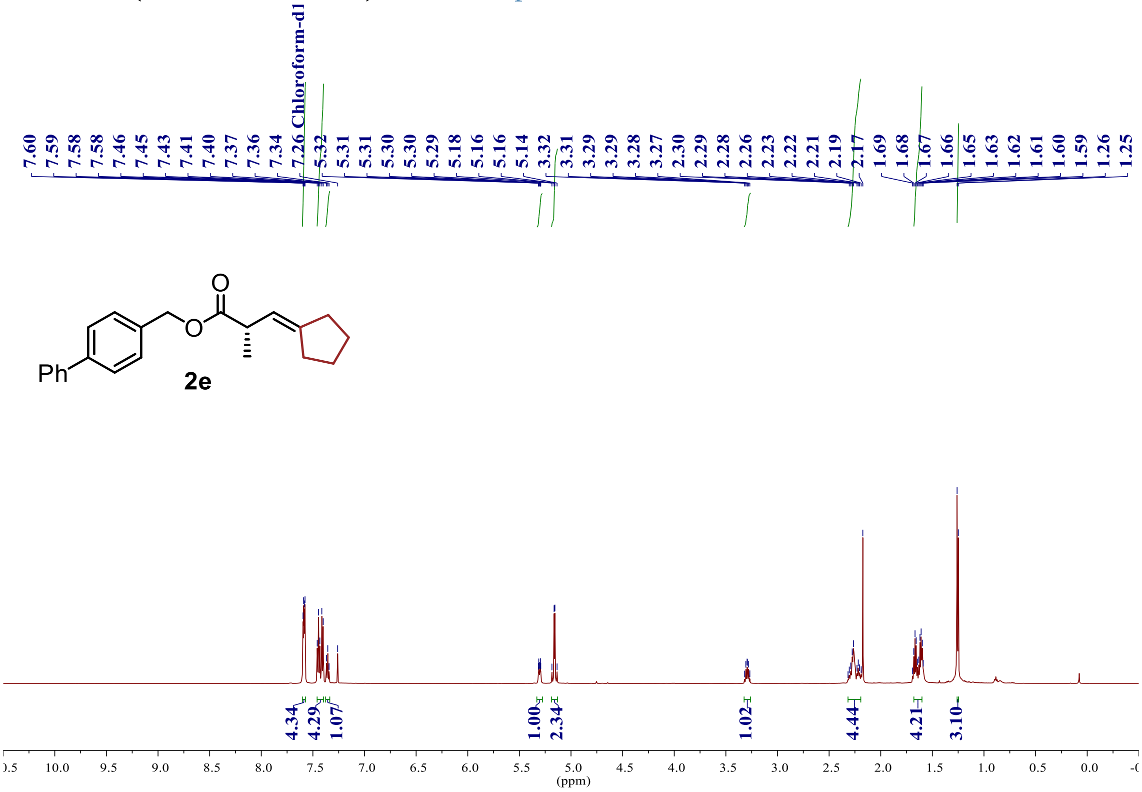

^{13}C{^{1}H} NMR (151 MHz, $CDCl_3$) of **2e**

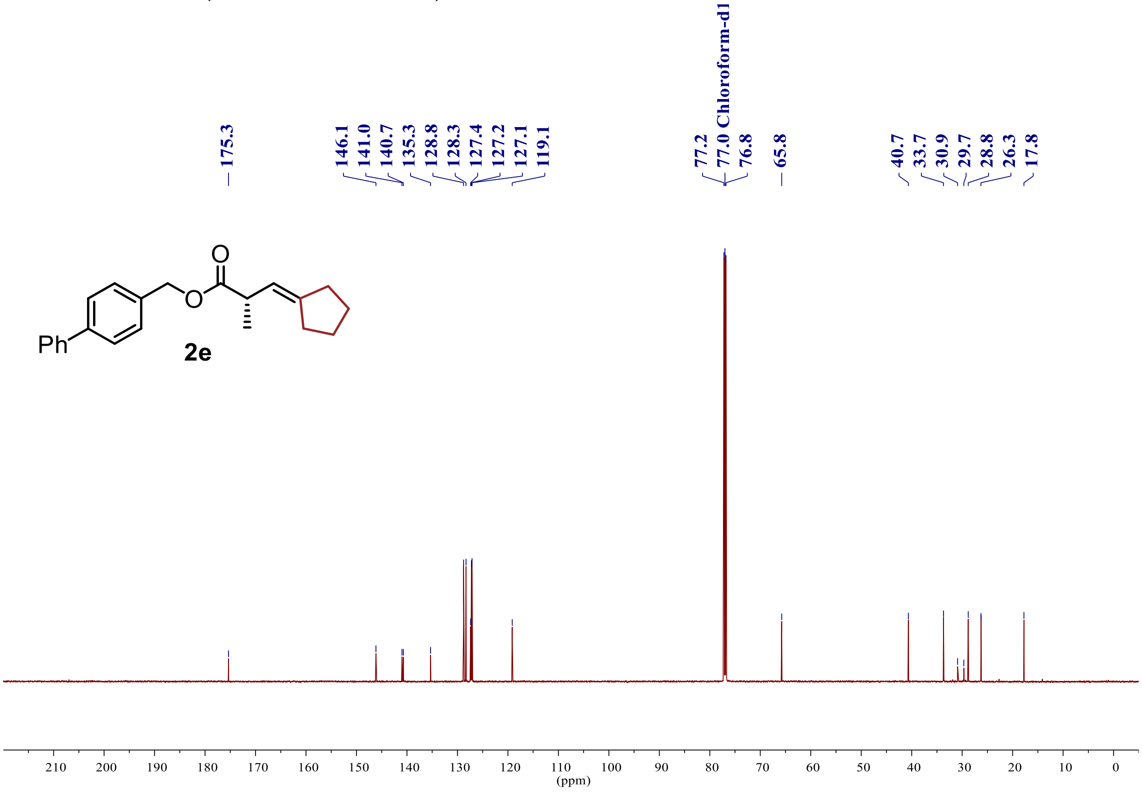

^{1}H NMR (600 MHz, $CDCl_3$) of **2f**, See procedure

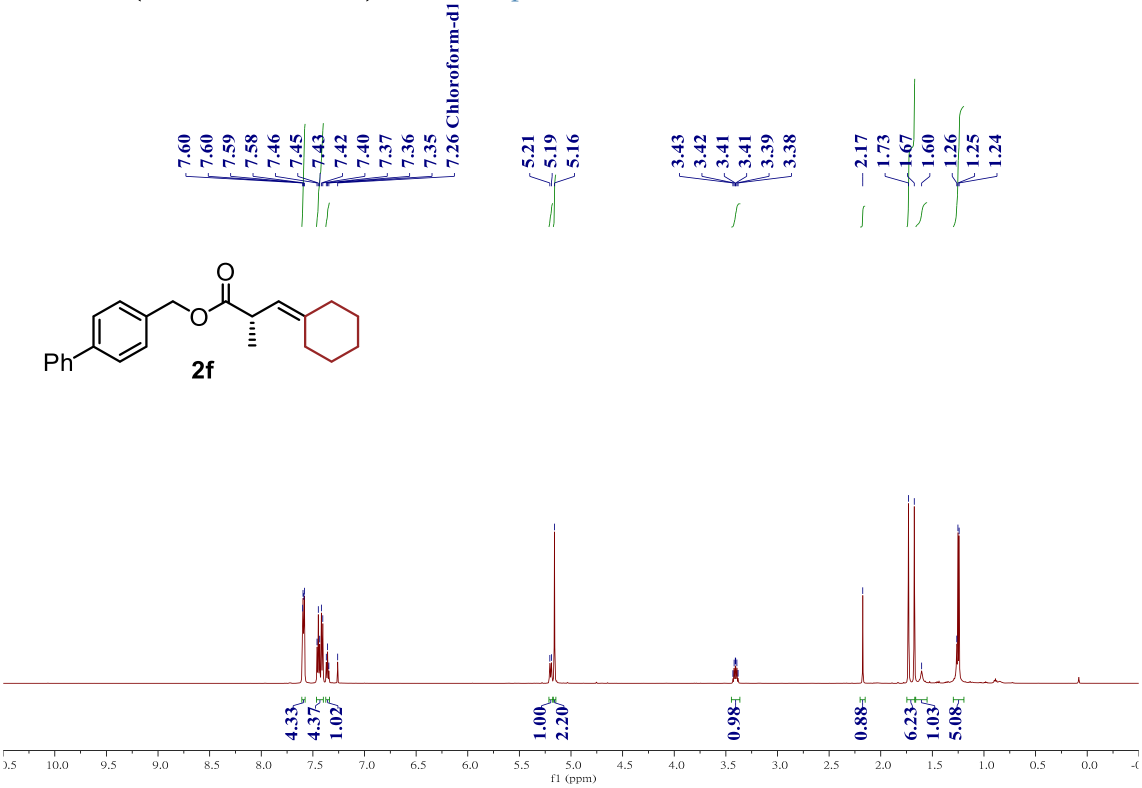

^{13}C{^{1}H} NMR (151 MHz, $CDCl_3$) of **2f**

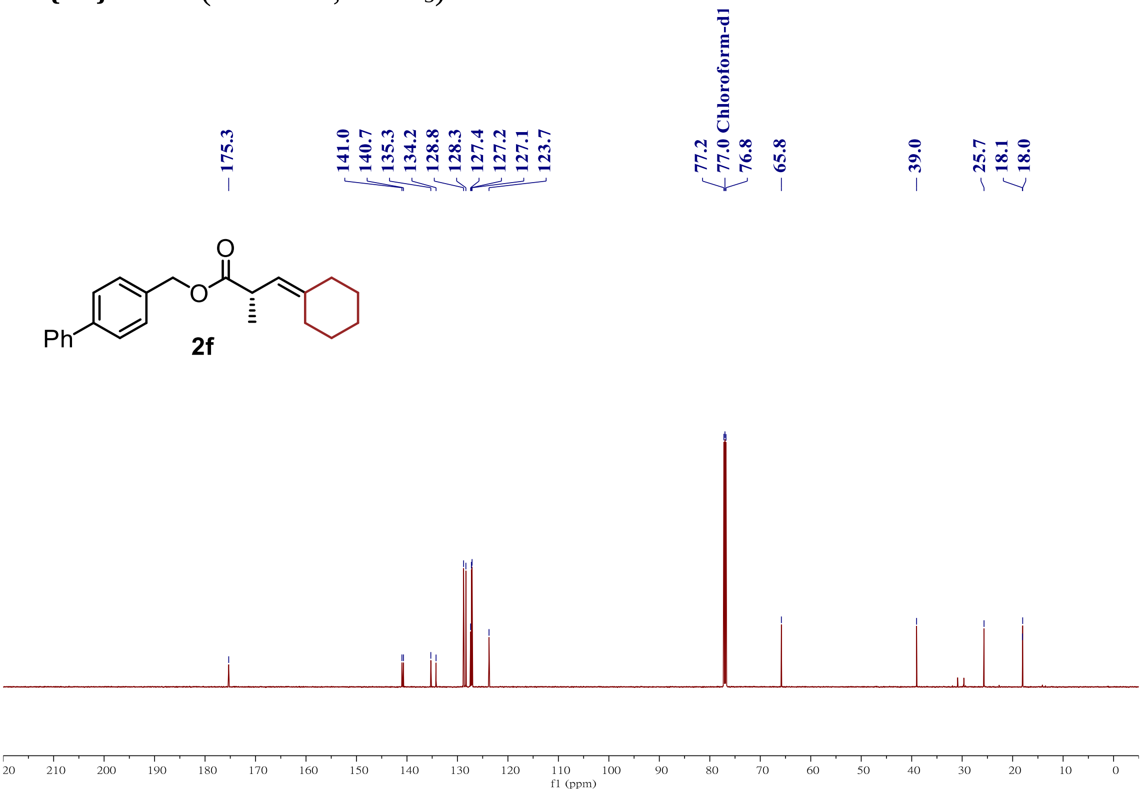

^{1}H NMR (600 MHz, $CDCl_3$) of **2g**, See procedure

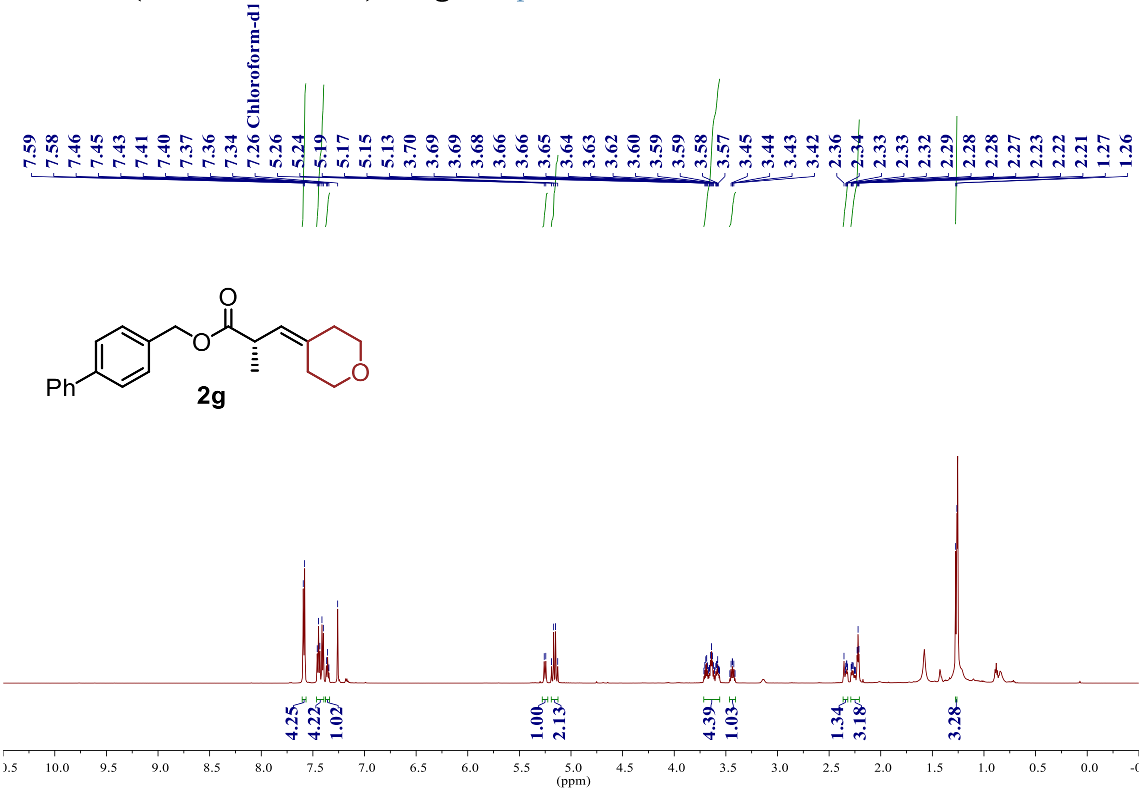

^{13}C{^{1}H} NMR (151 MHz, $CDCl_3$) of **2g**

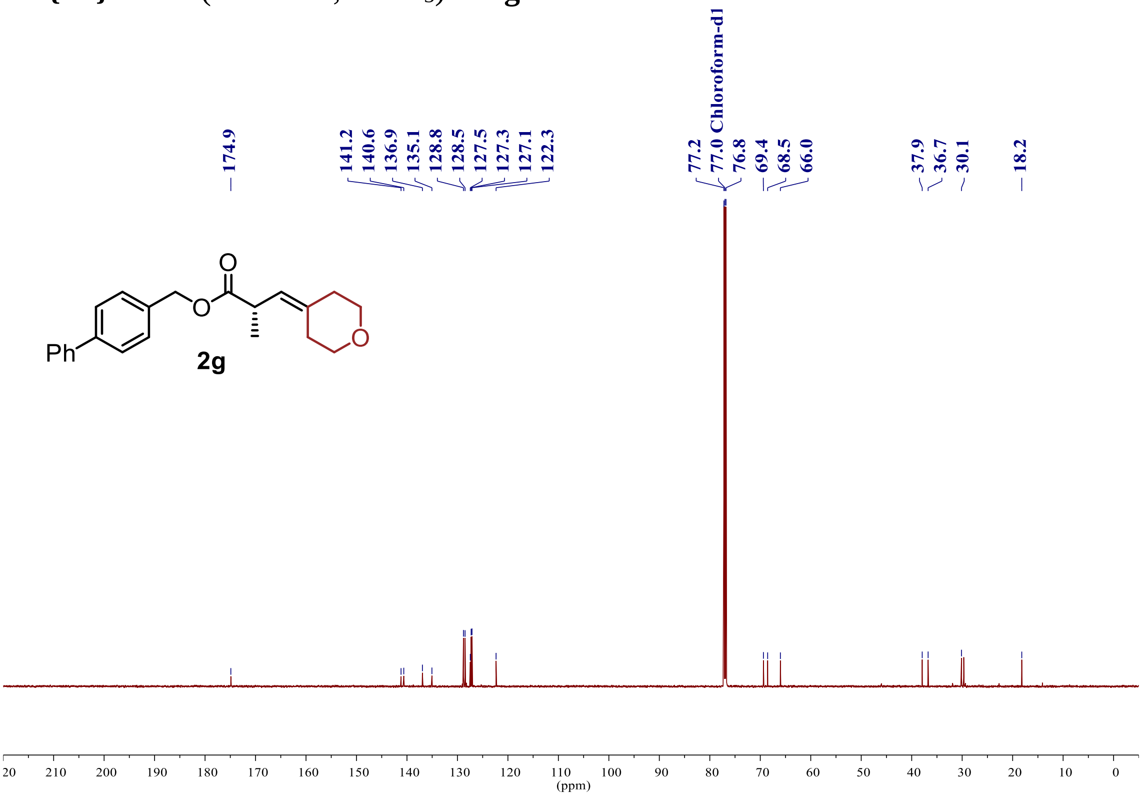

[Back to contents](#)

^{1}**H NMR** (400 MHz, $CDCl_3$) of **2h**, See procedure

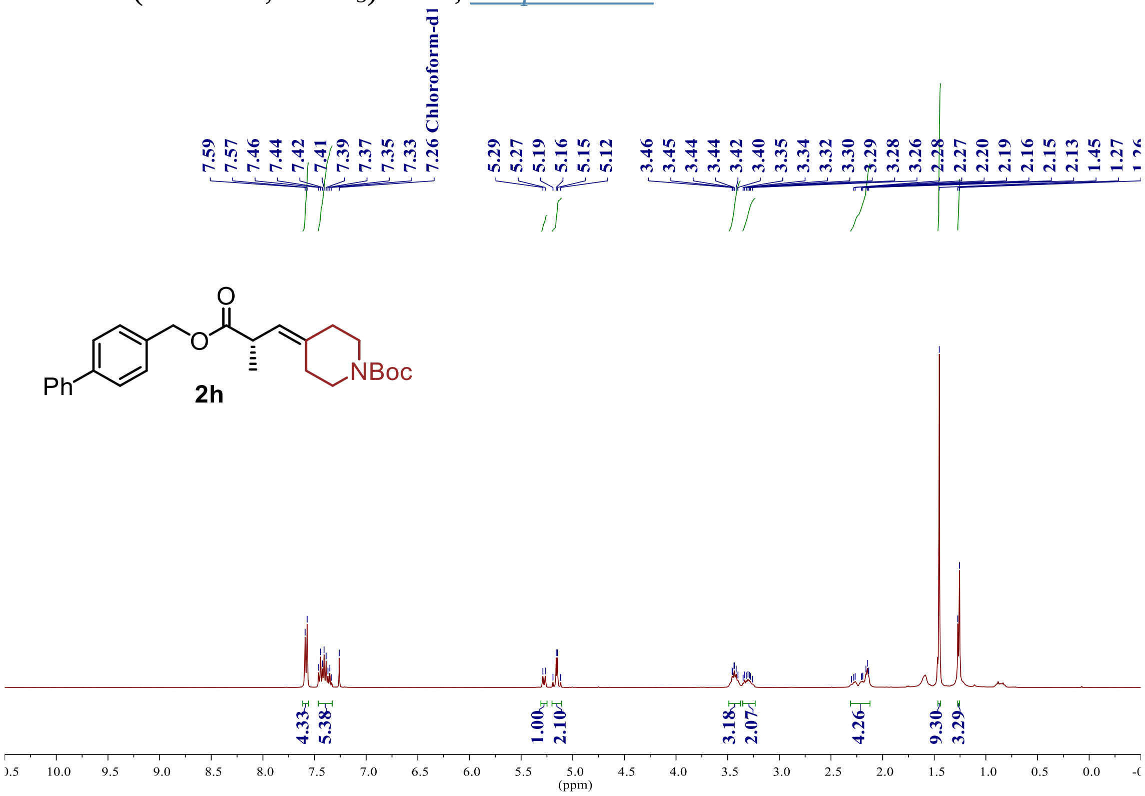

^{13}**C{^{1}H} NMR** (101 MHz, $CDCl_3$) of **2h**

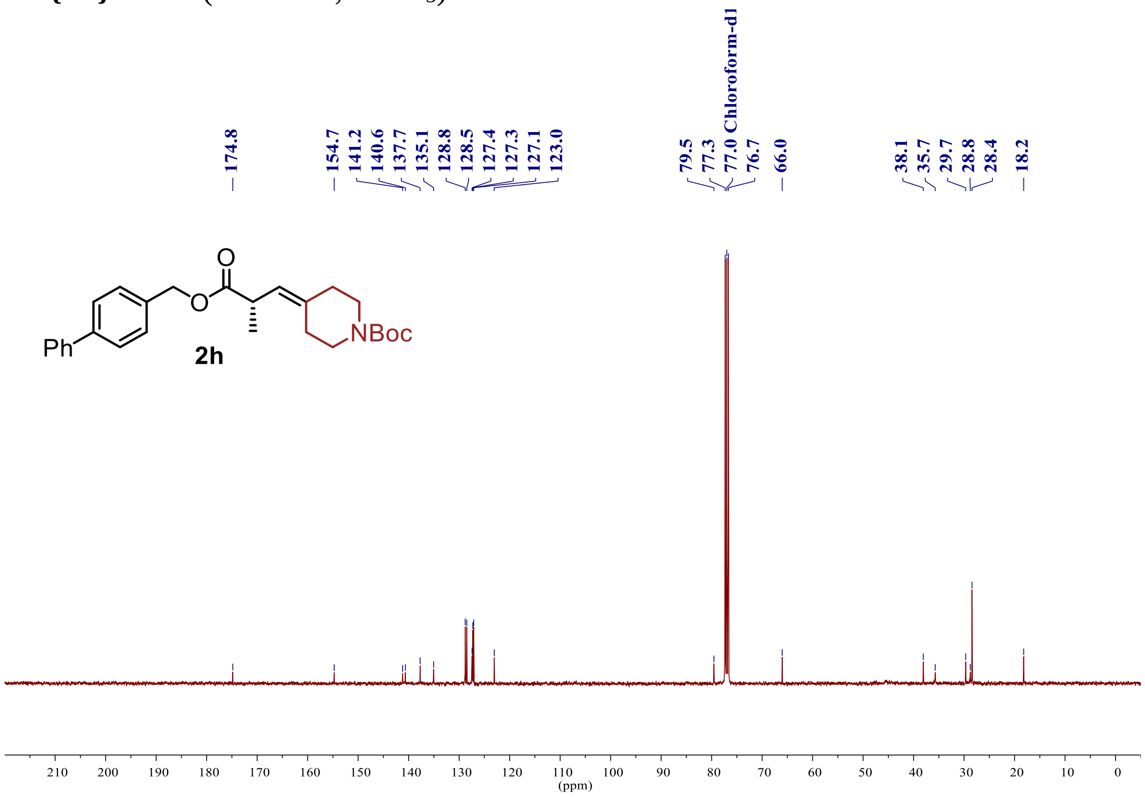

Back to contents

^{1}H NMR (400 MHz, $CDCl_3$) of **2i**, See procedure

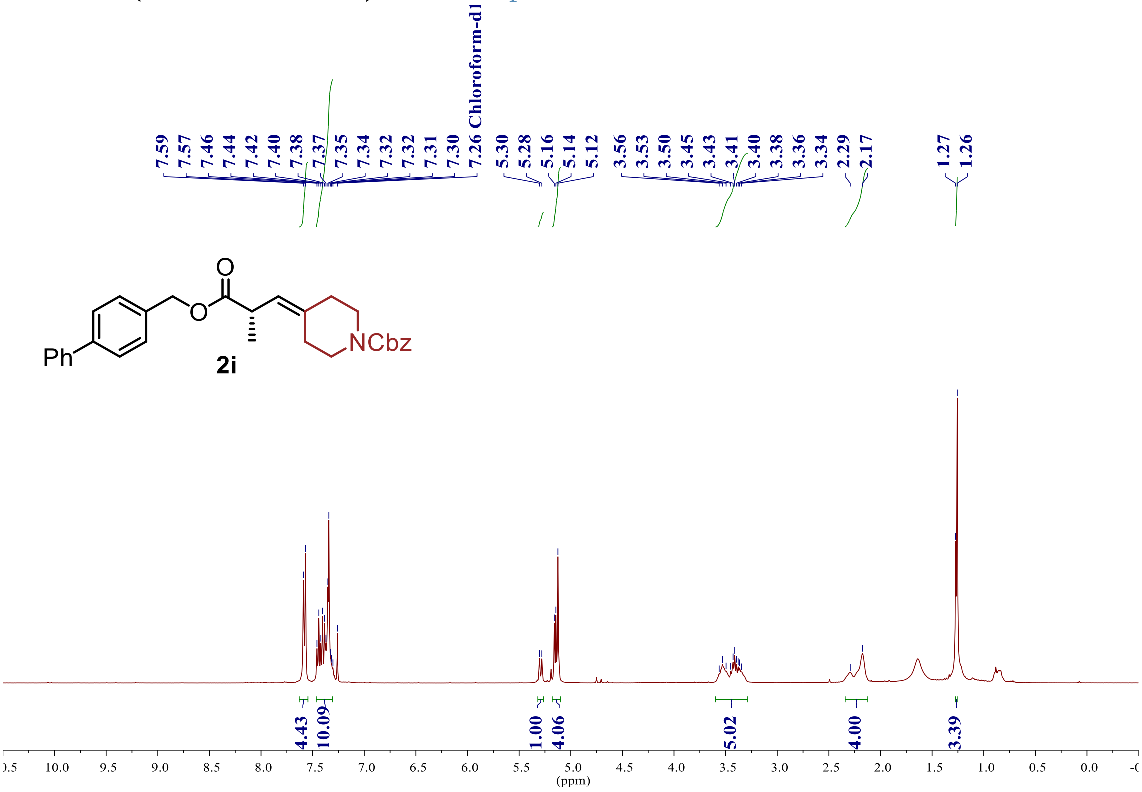

^{13}C{^{1}H} NMR (101 MHz, $CDCl_3$) of **2i**

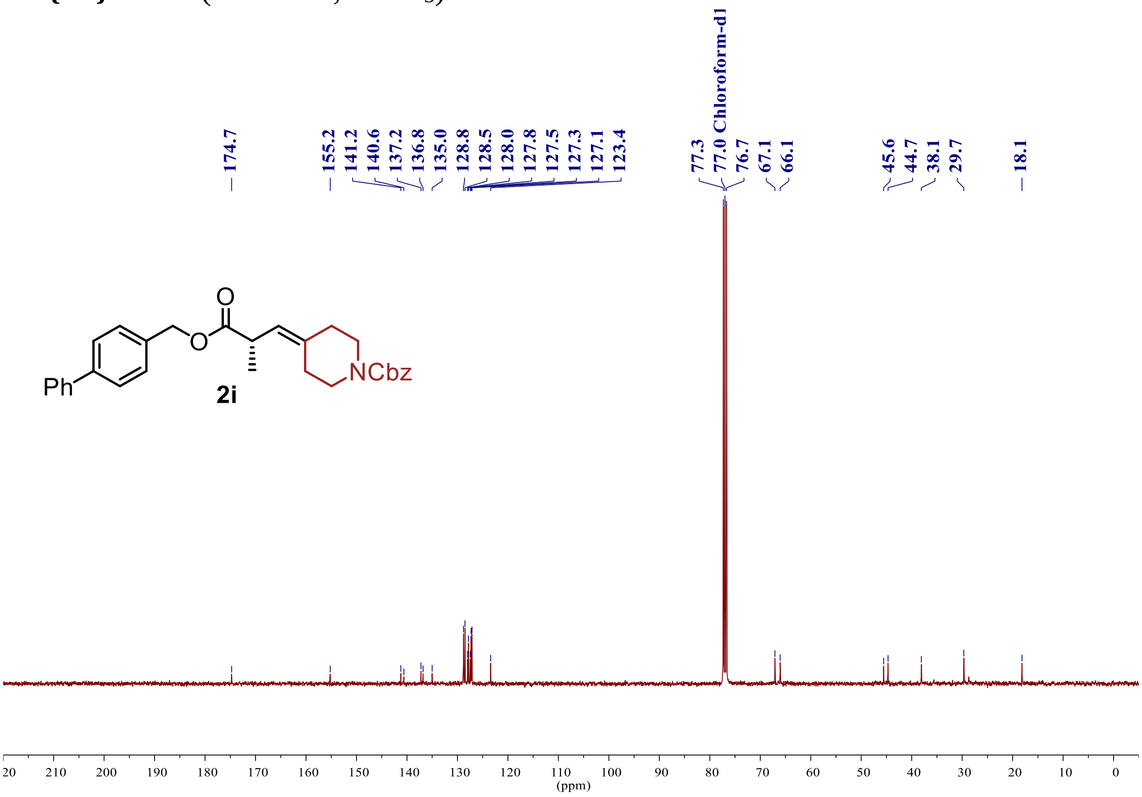

Back to contents

¹H NMR (400 MHz, $CDCl_3$) of **2j**, See procedure

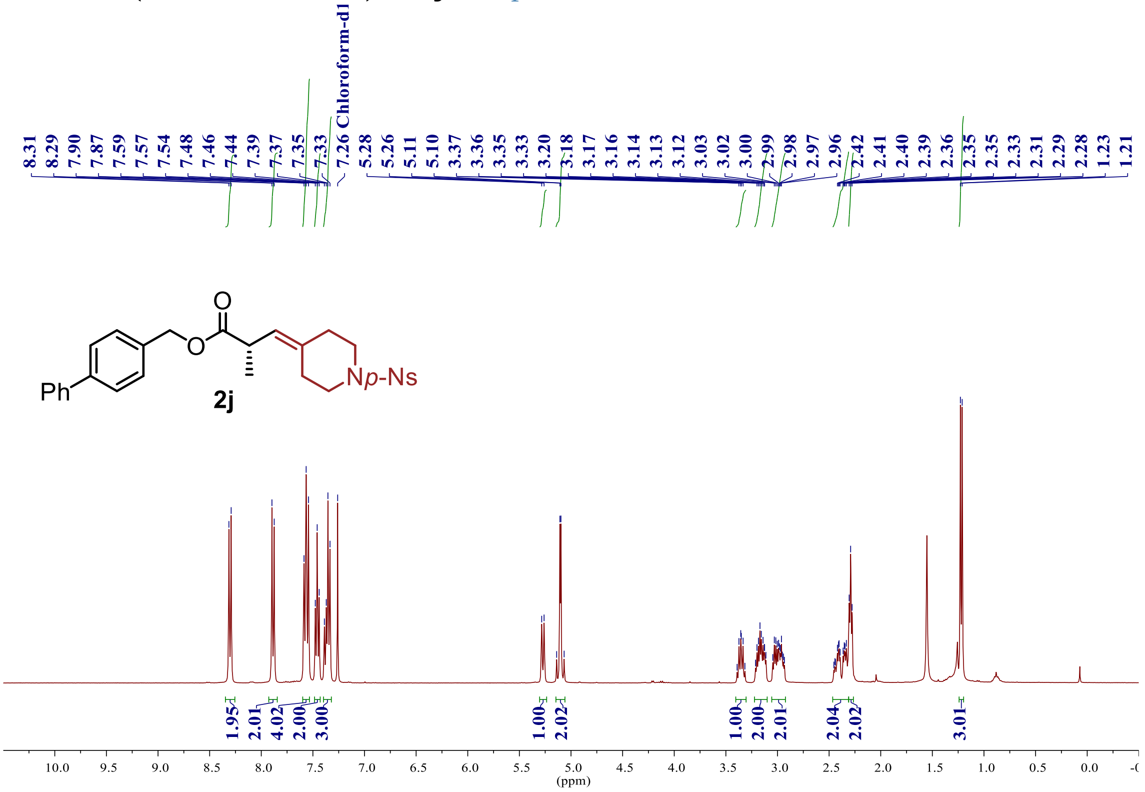

¹³C{¹H} NMR (101 MHz, $CDCl_3$) of **2j**

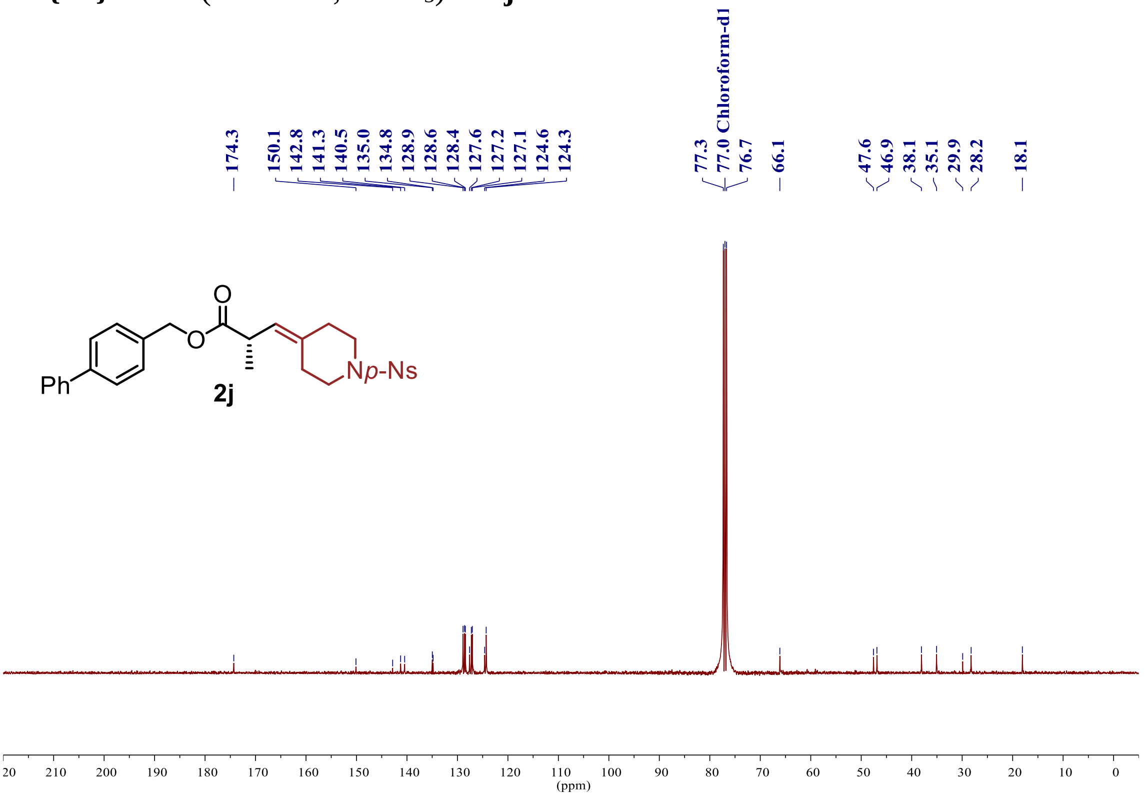

[Back to contents](#)

¹H NMR (600 MHz, $CDCl_3$) of **2k**, See procedure

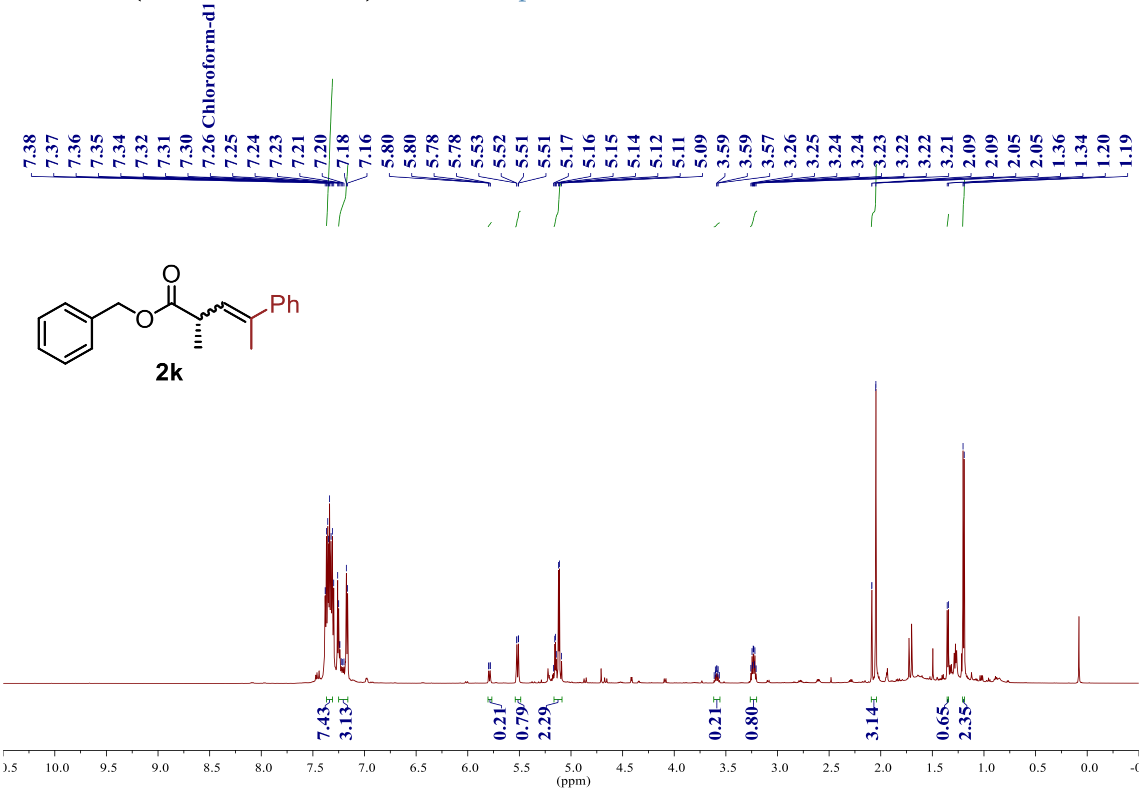

$^{13}C\{^{1}H\}$ NMR (151 MHz, $CDCl_3$) of **2k**

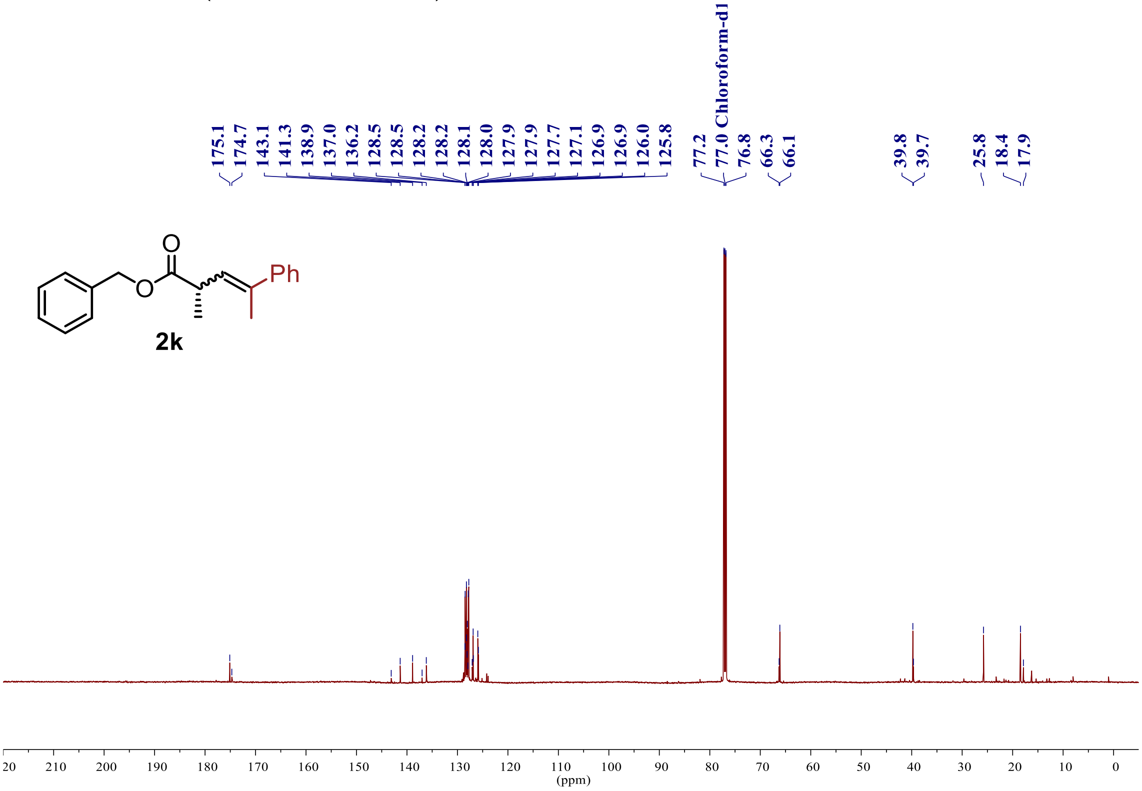

[Back to contents](#)

^{1}H NMR (600 MHz, $CDCl_3$) of **2l**, [See procedure](#)

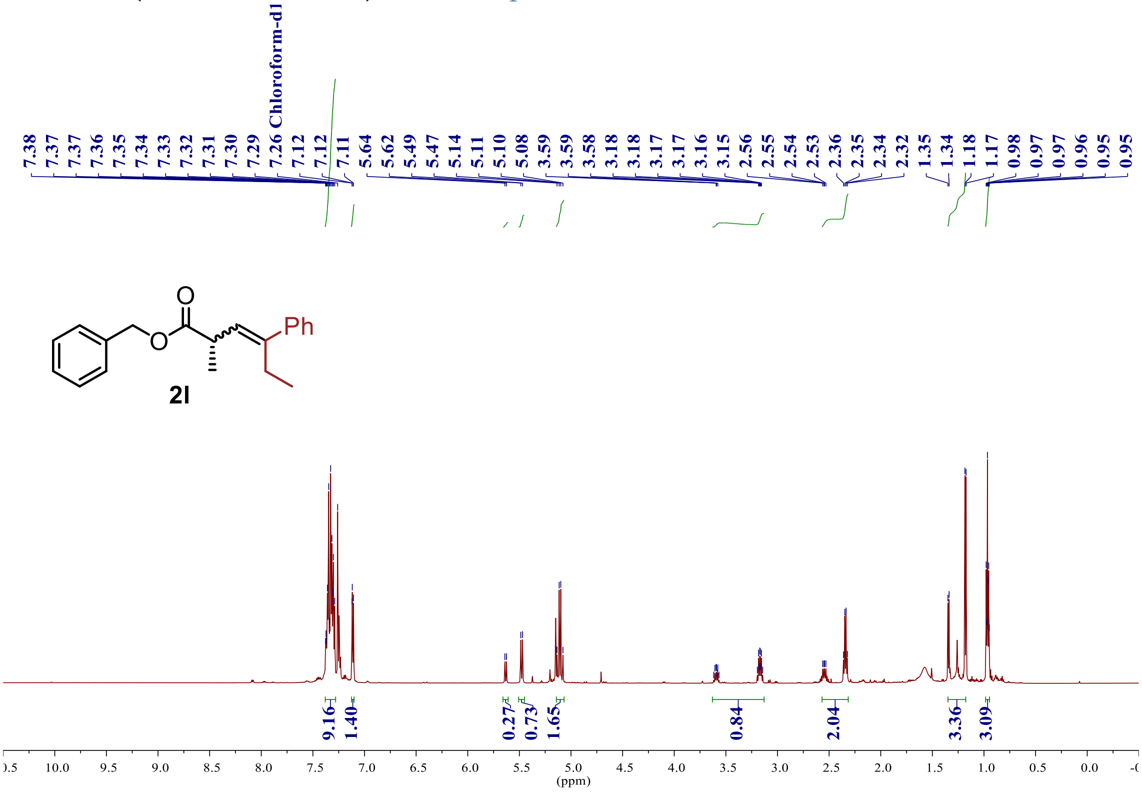

^{13}C{^{1}H} NMR (151 MHz, $CDCl_3$) of **2l**

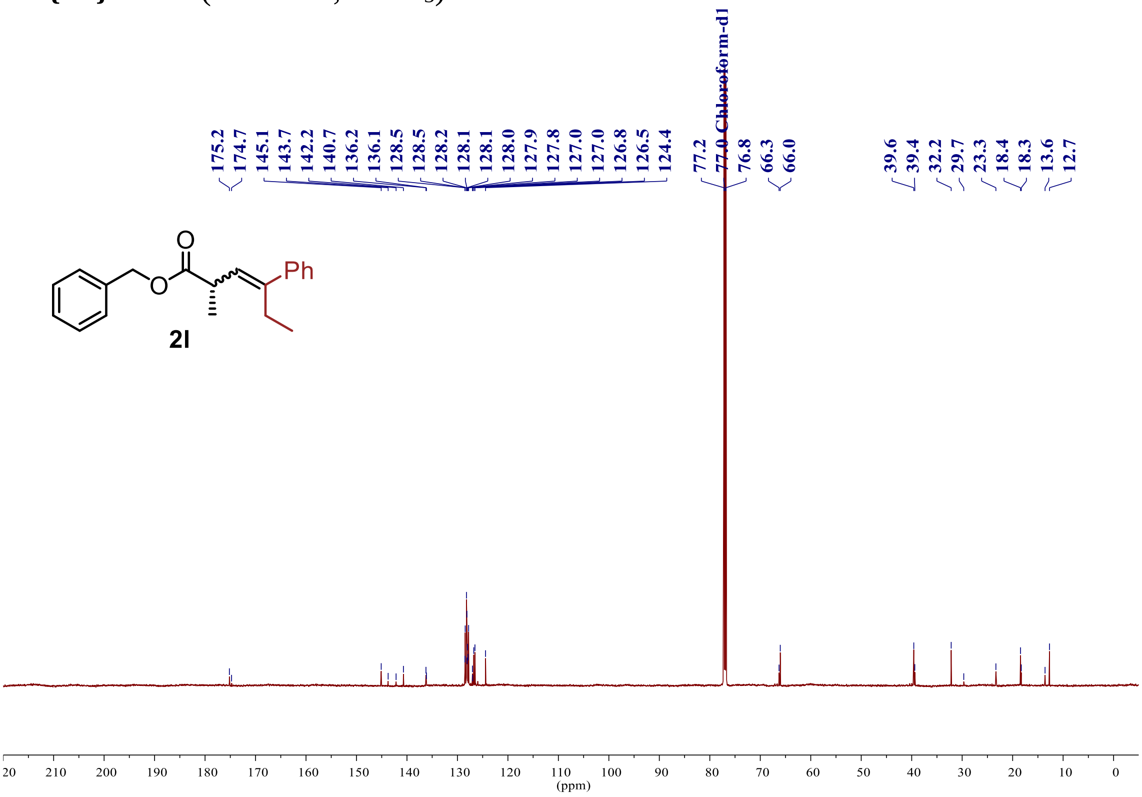

Back to contents

^{1}H NMR (600 MHz, $CDCl_3$) of **2m**, See procedure

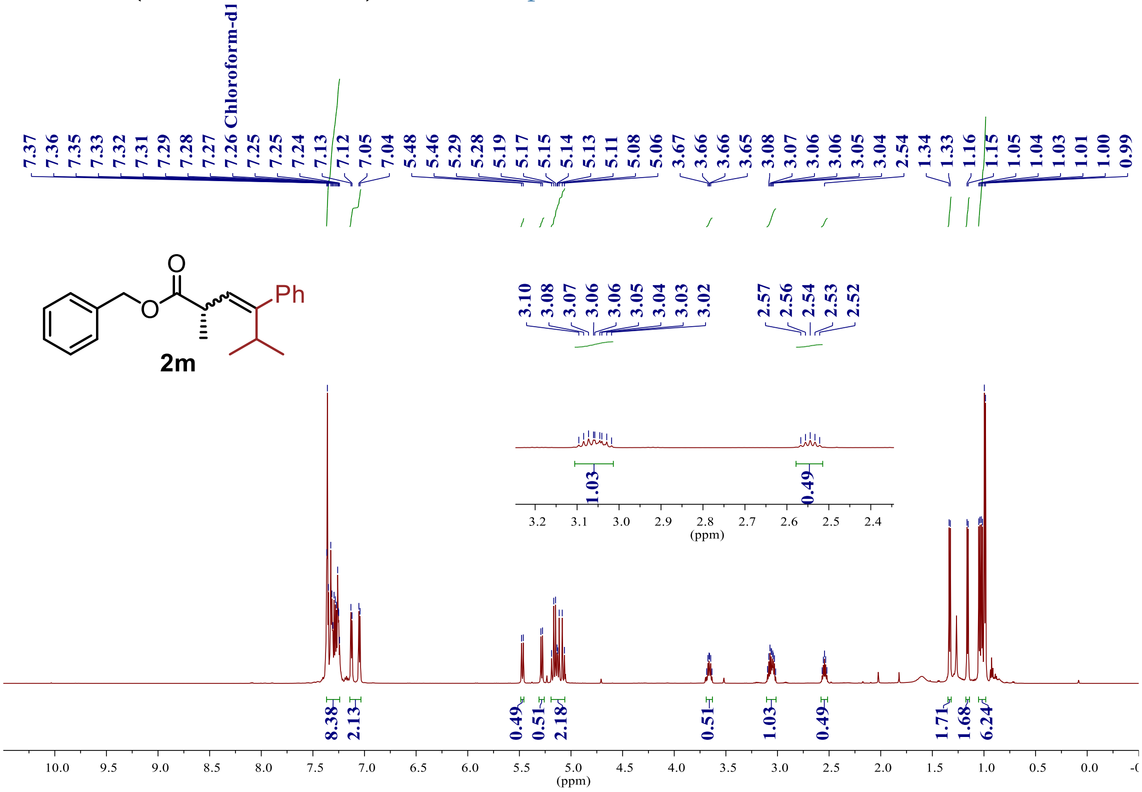

^{13}C{^{1}H} NMR (151 MHz, $CDCl_3$) of **2m**

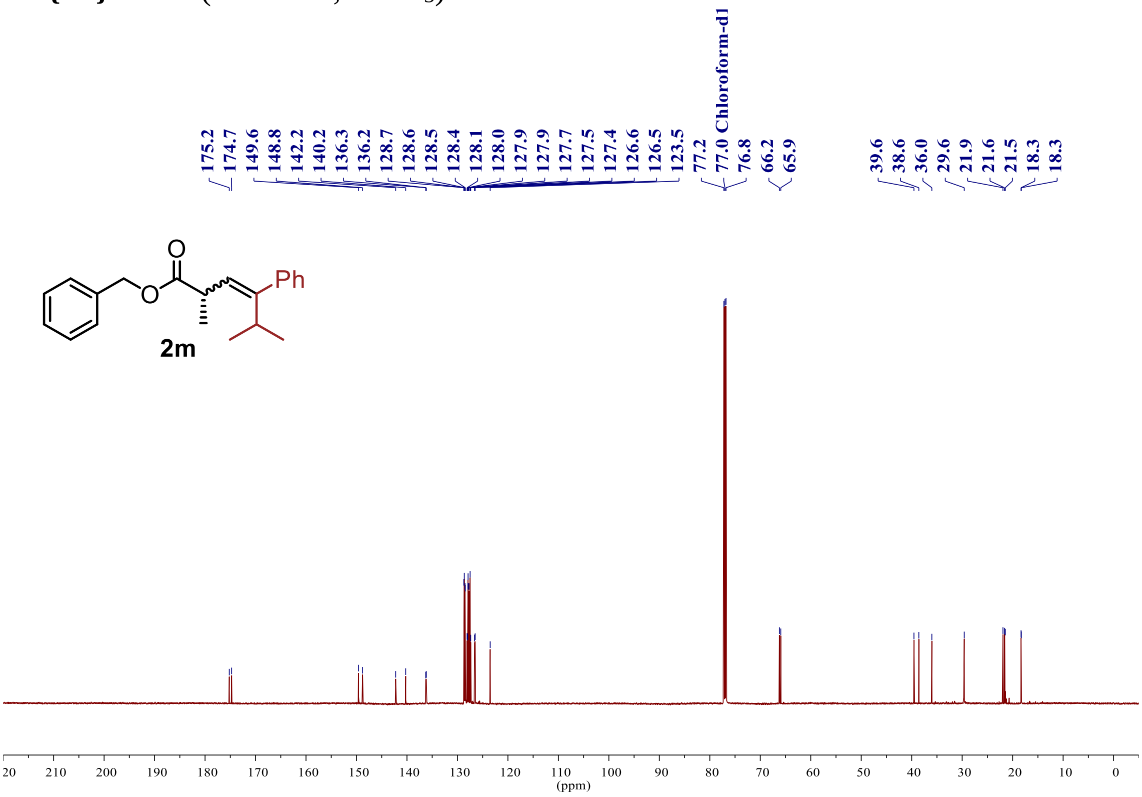

Back to contents

^{1}H NMR (600 MHz, $CDCl_3$) of **2n**, See procedure

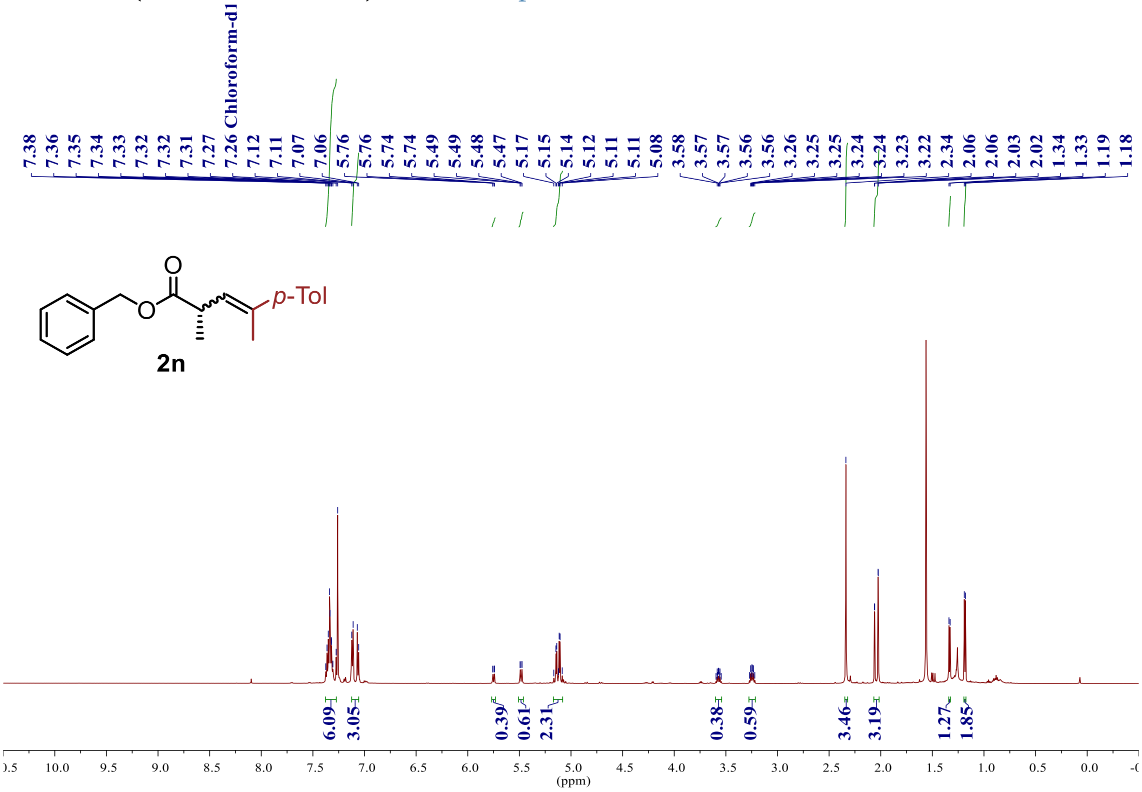

^{13}C{^{1}H} NMR (101 MHz, $CDCl_3$) of **2n**

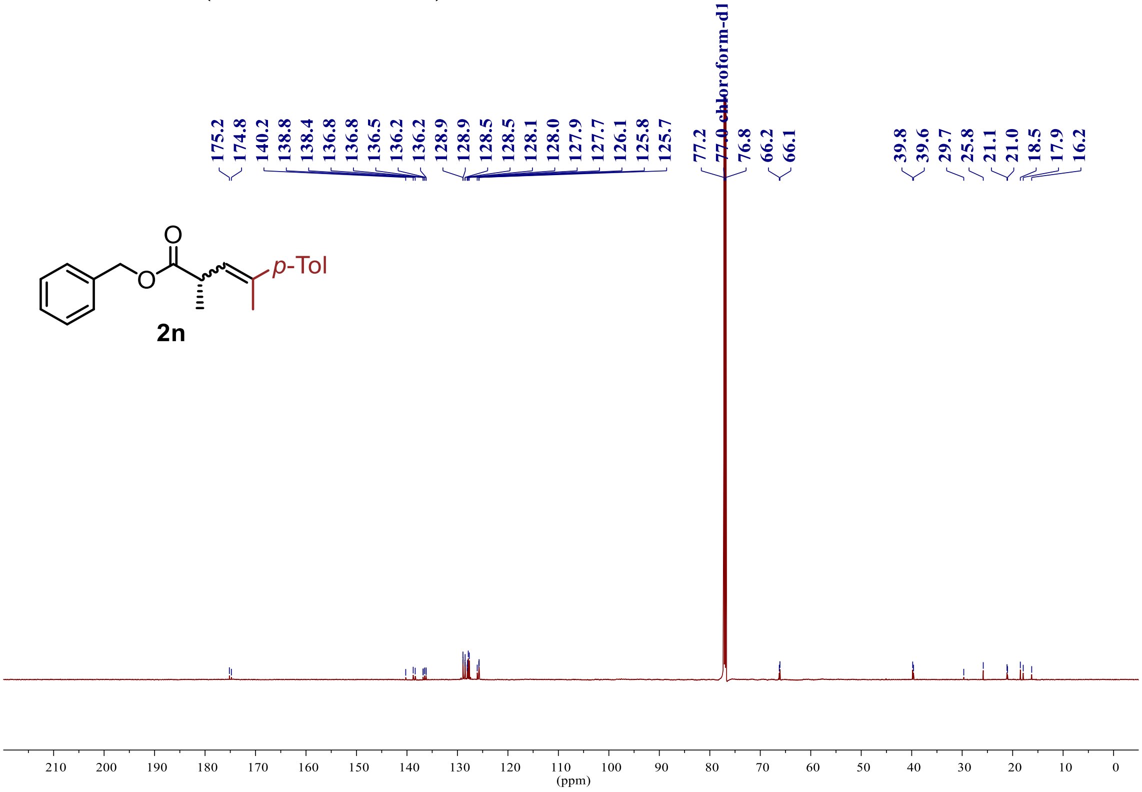

Back to contents

¹H NMR (600 MHz, $CDCl_3$) of **2n'**, See procedure

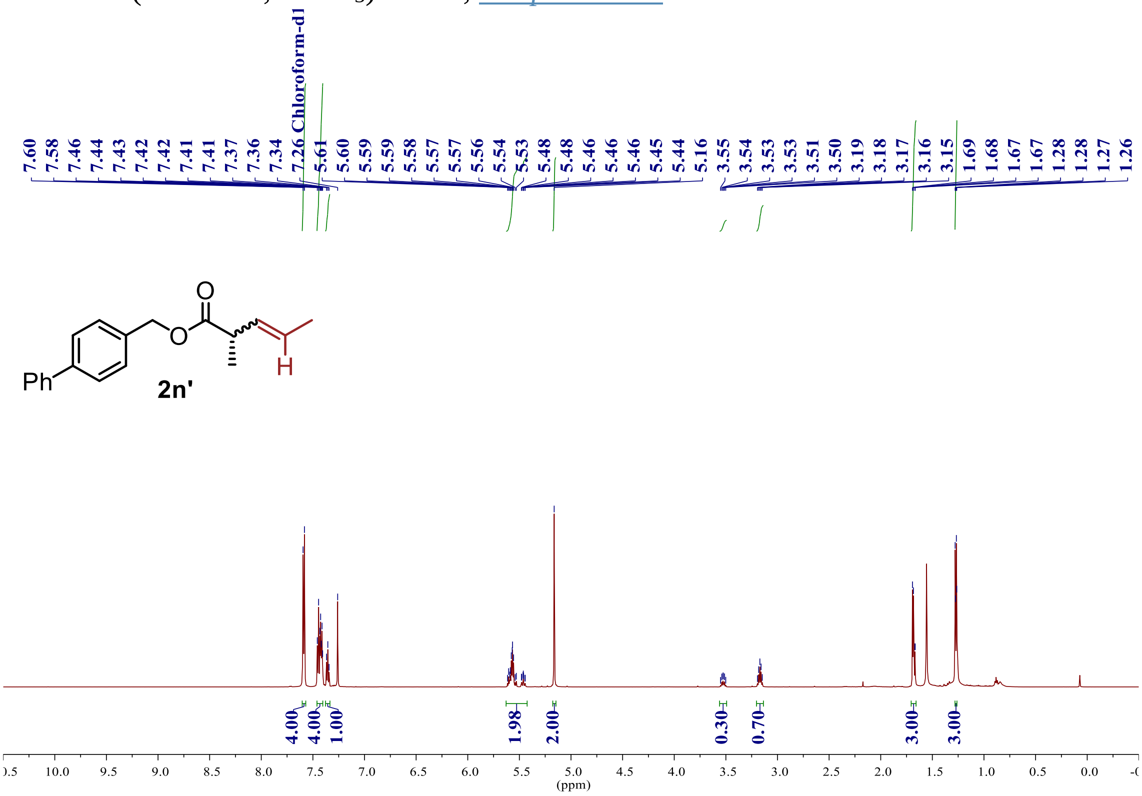

¹³C{¹H} NMR (151 MHz, $CDCl_3$) of **2n'**

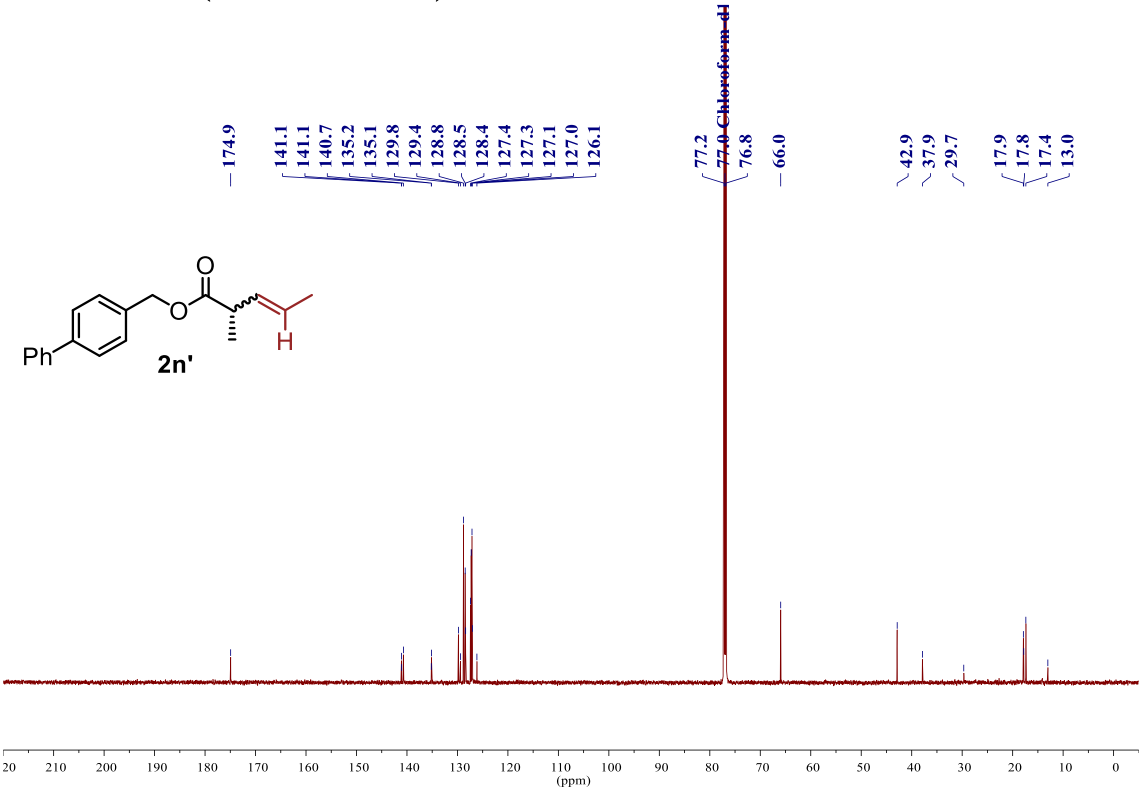

Back to contents

^{1}H NMR (400 MHz, $CDCl_3$) of **2o**, See procedure

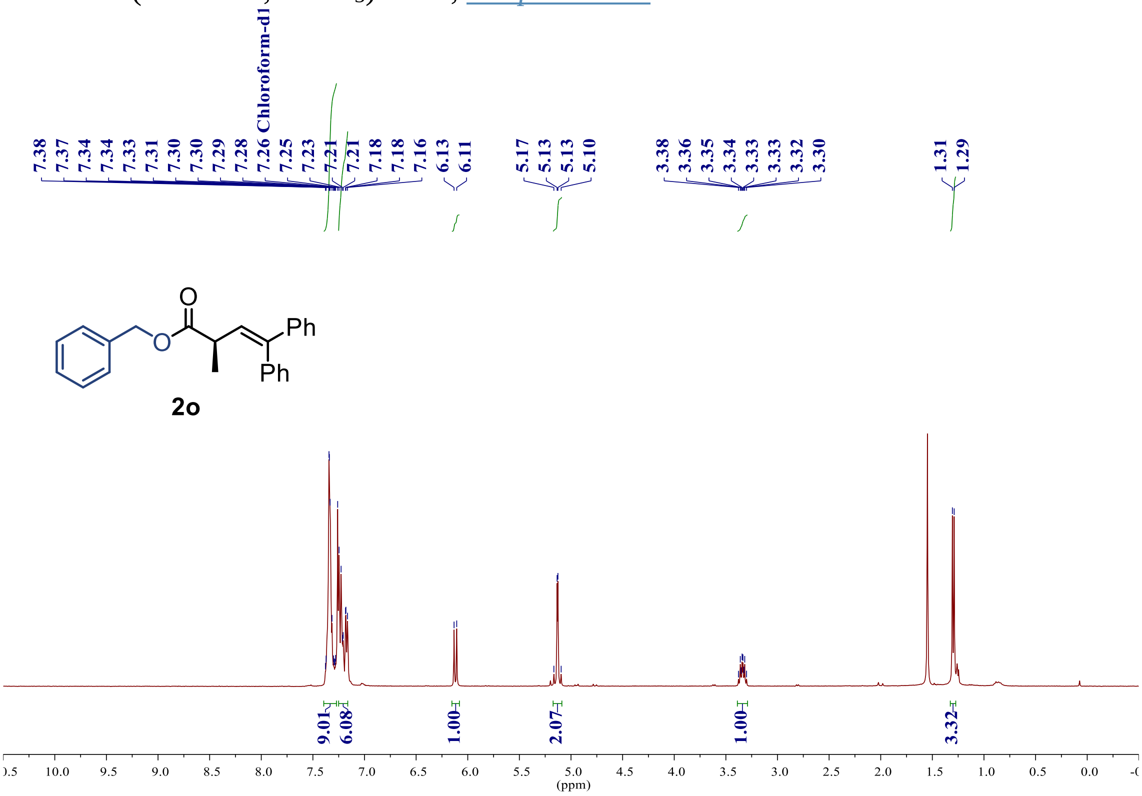

^{13}C{^{1}H} NMR (101 MHz, $CDCl_3$) of **2o**

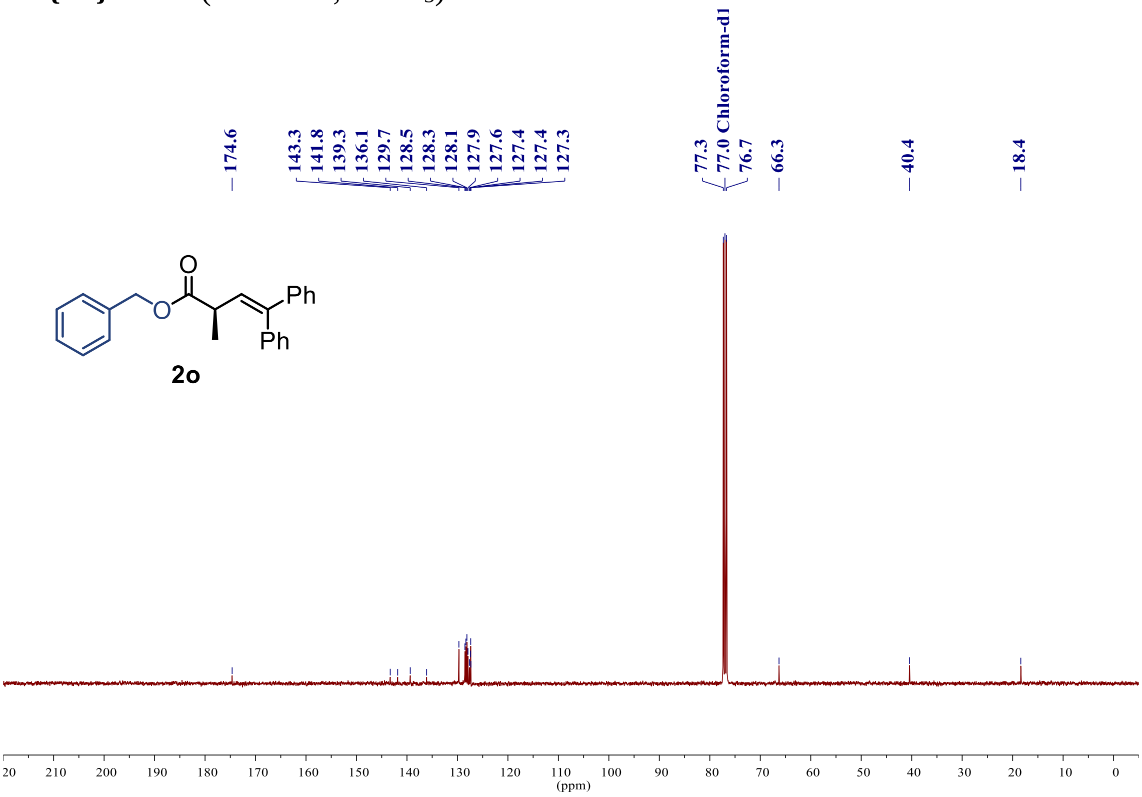

[Back to contents](#)

^{1}H NMR (400 MHz, $CDCl_3$) of **2p**, See procedure

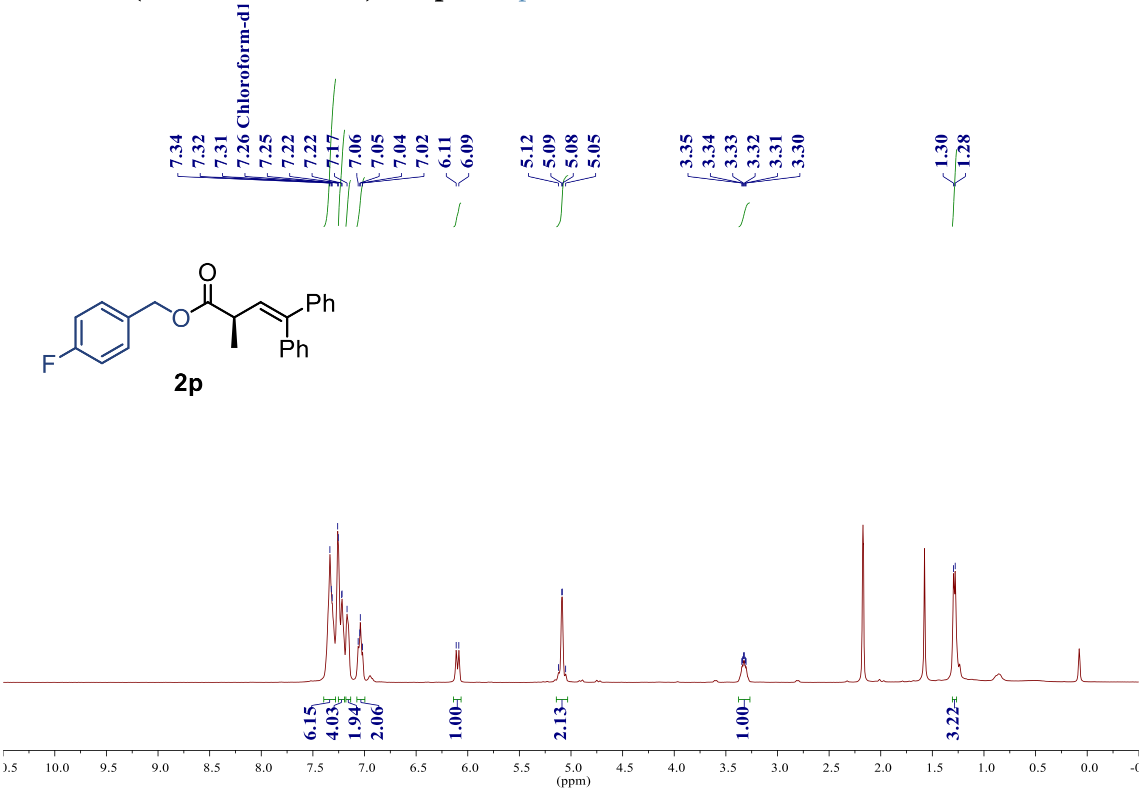

^{13}C{^{1}H} NMR (101 MHz, $CDCl_3$) of **2p**

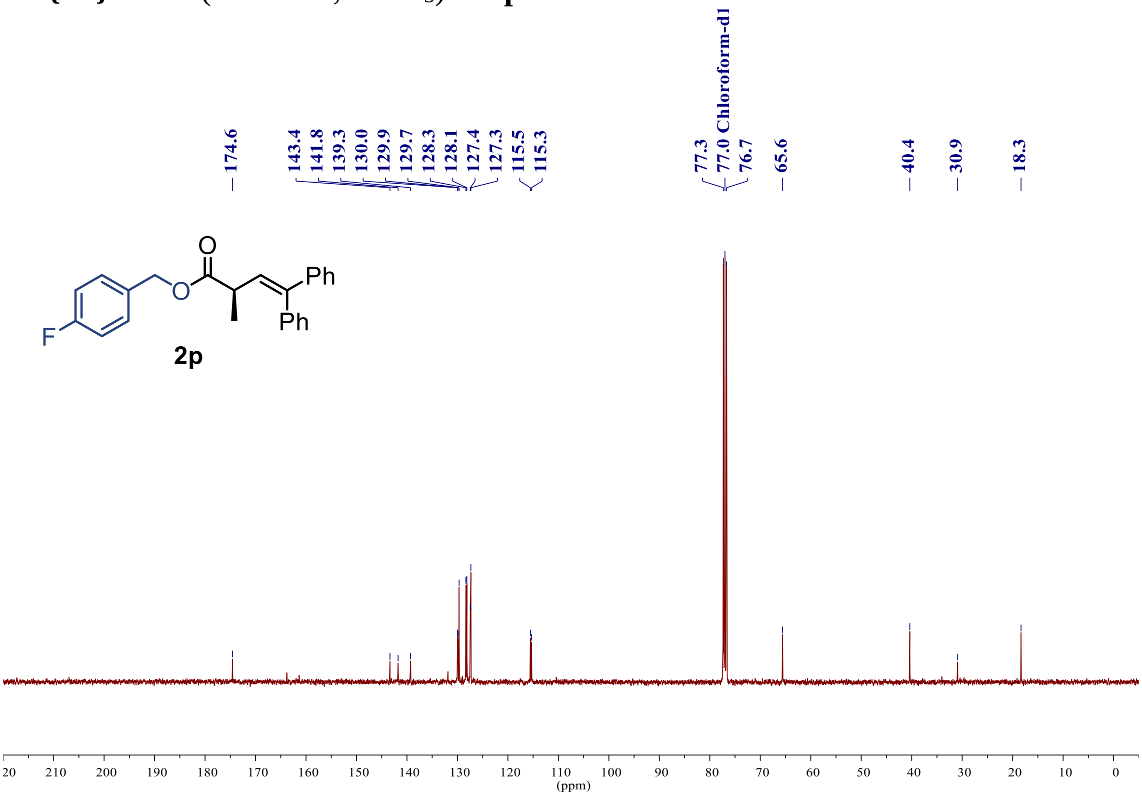

Back to contents

^{19}F NMR (282 MHz, $CDCl_3$) of **2p**

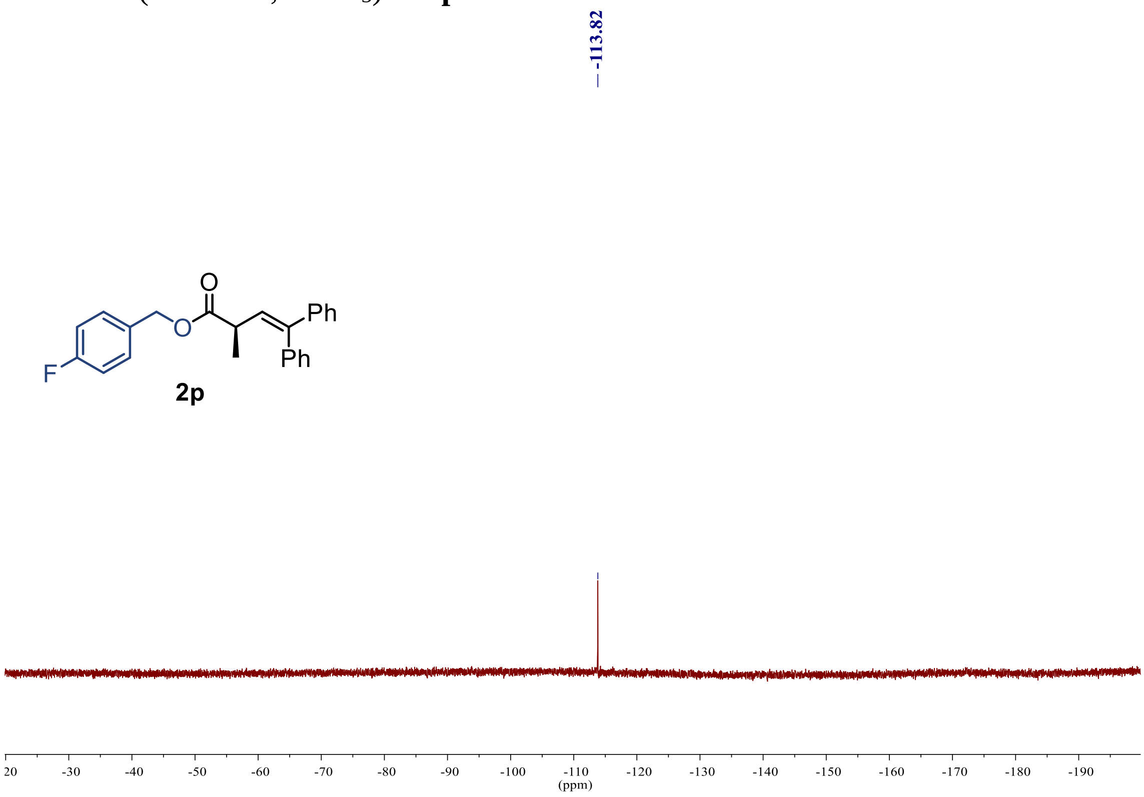

^{1}H NMR (400 MHz, $CDCl_3$) of **2q**, See procedure

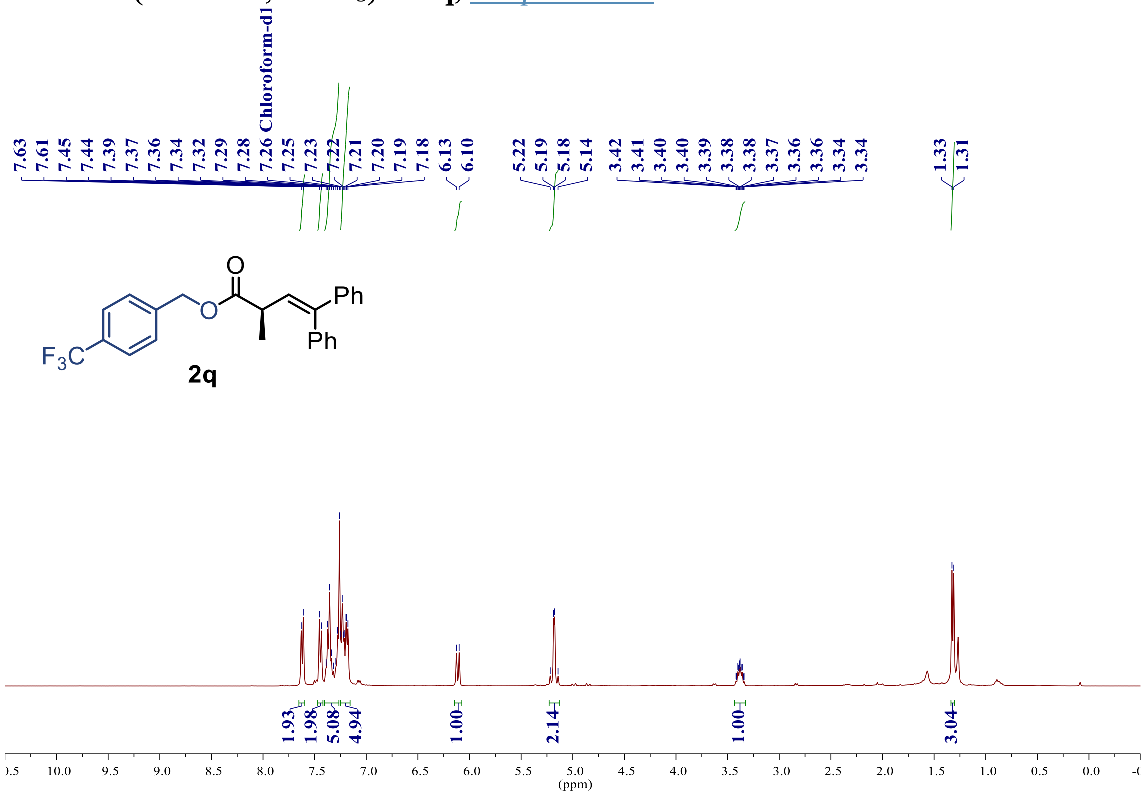

Back to contents

$^{13}C\{^{1}H\}$ NMR (101 MHz, $CDCl_3$) of **2q**

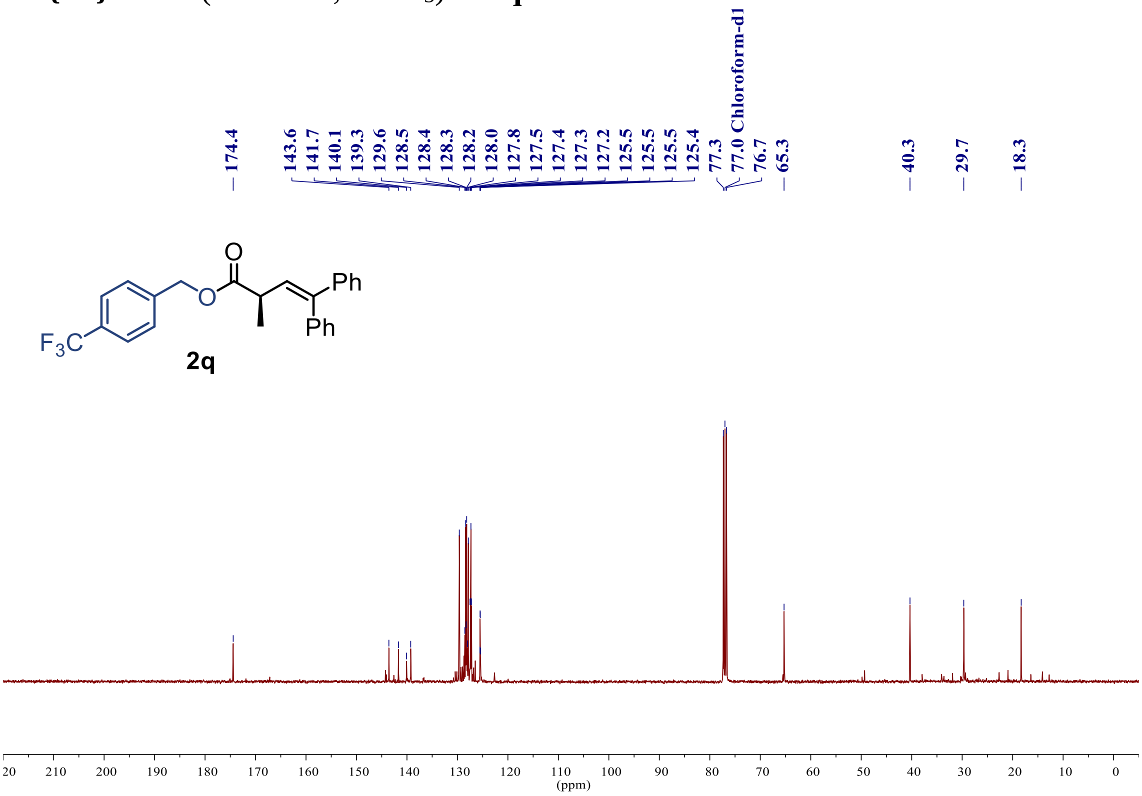

^{19}F NMR (282 MHz, $CDCl_3$) of **2q**

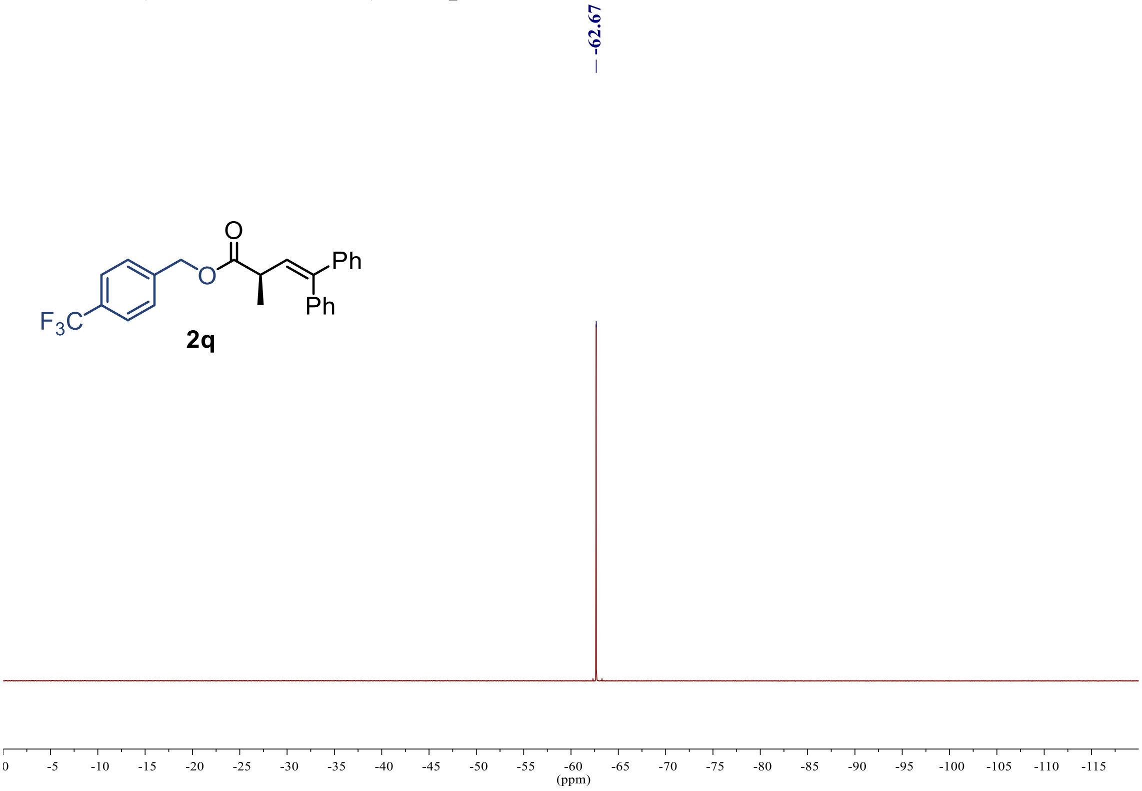

Back to contents

^{1}**H NMR** (400 MHz, $CDCl_3$) of **2r**, See procedure

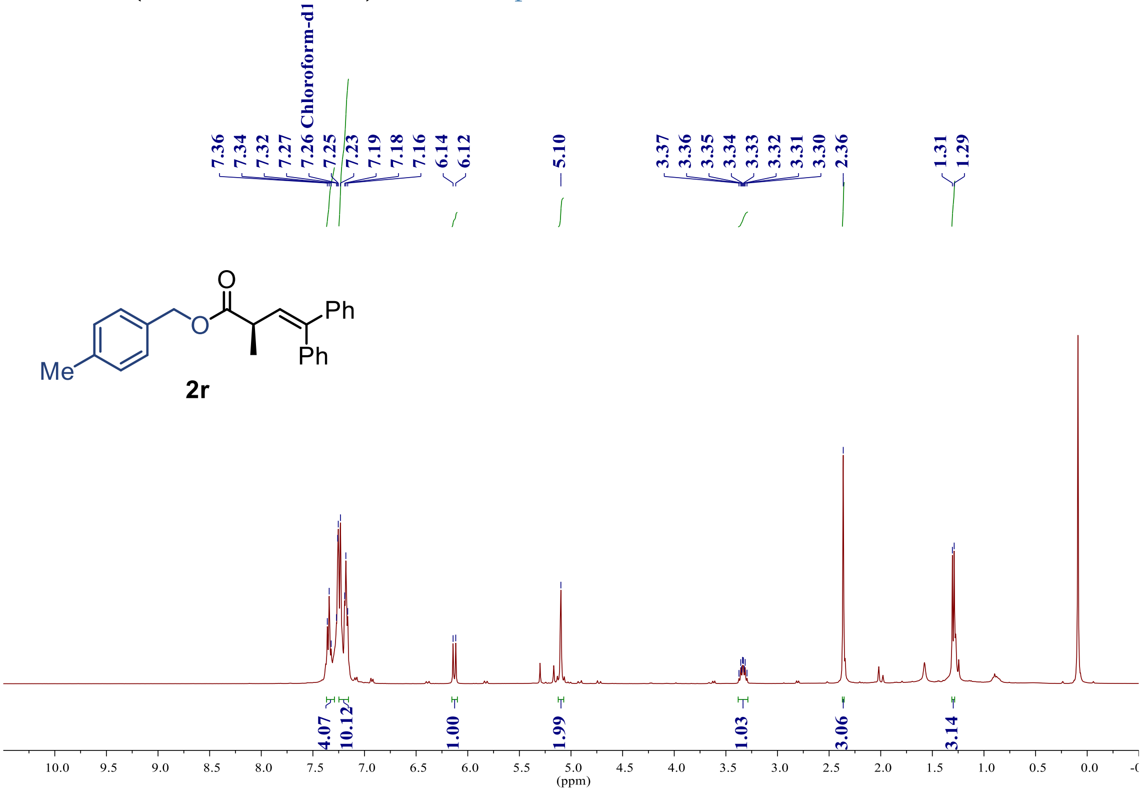

^{13}**C{^{1}H} NMR** (101 MHz, $CDCl_3$) of **2r**

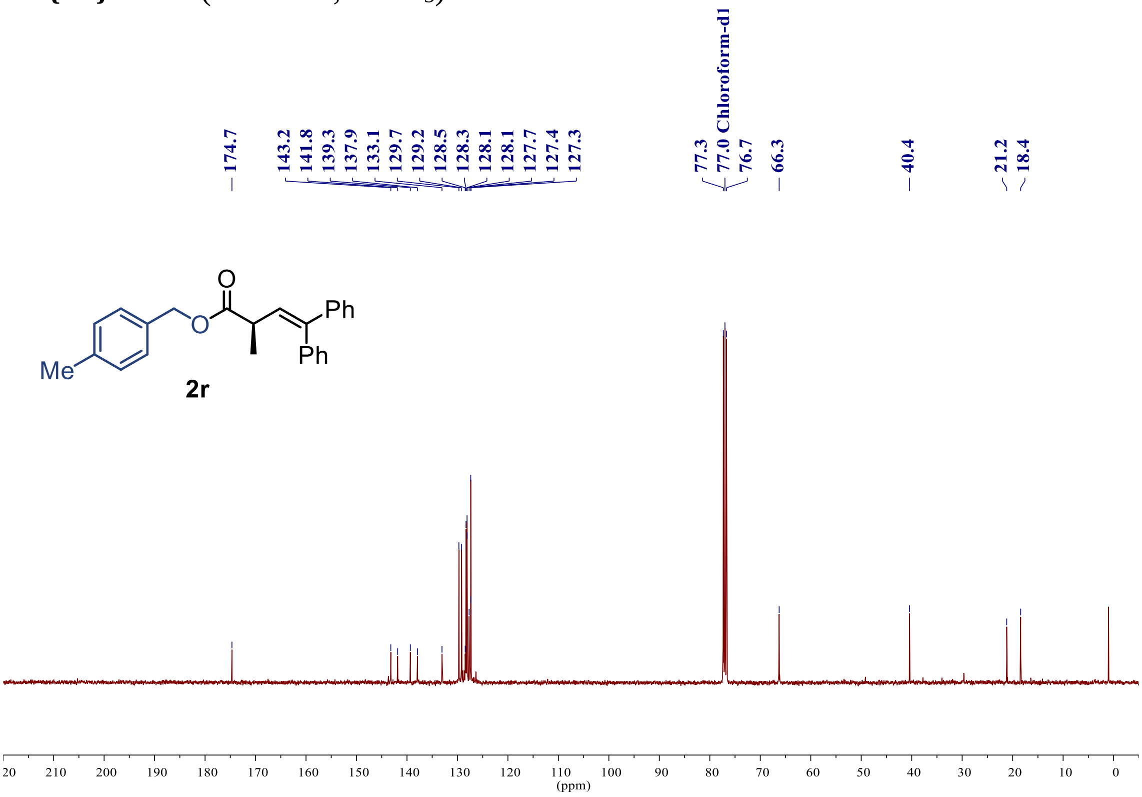

Back to contents

¹H NMR (400 MHz, $CDCl_3$) of **2s**, See procedure

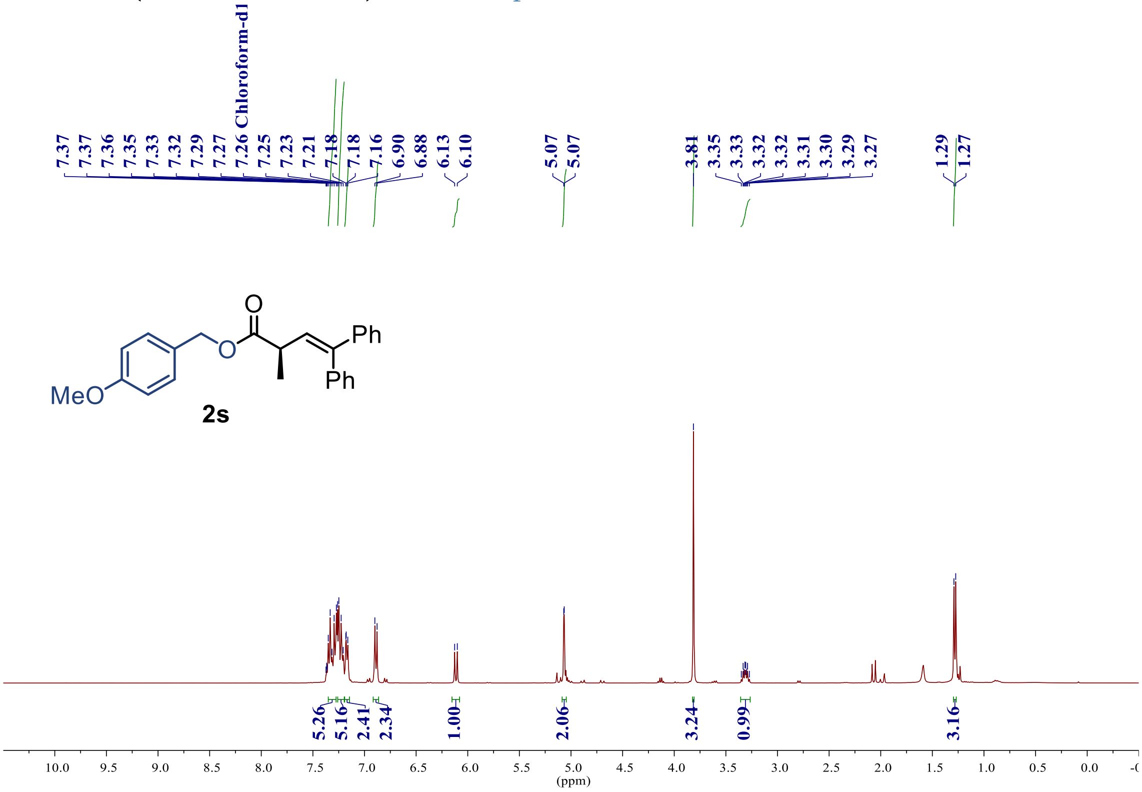

¹³C{¹H} NMR (101 MHz, $CDCl_3$) of **2s**

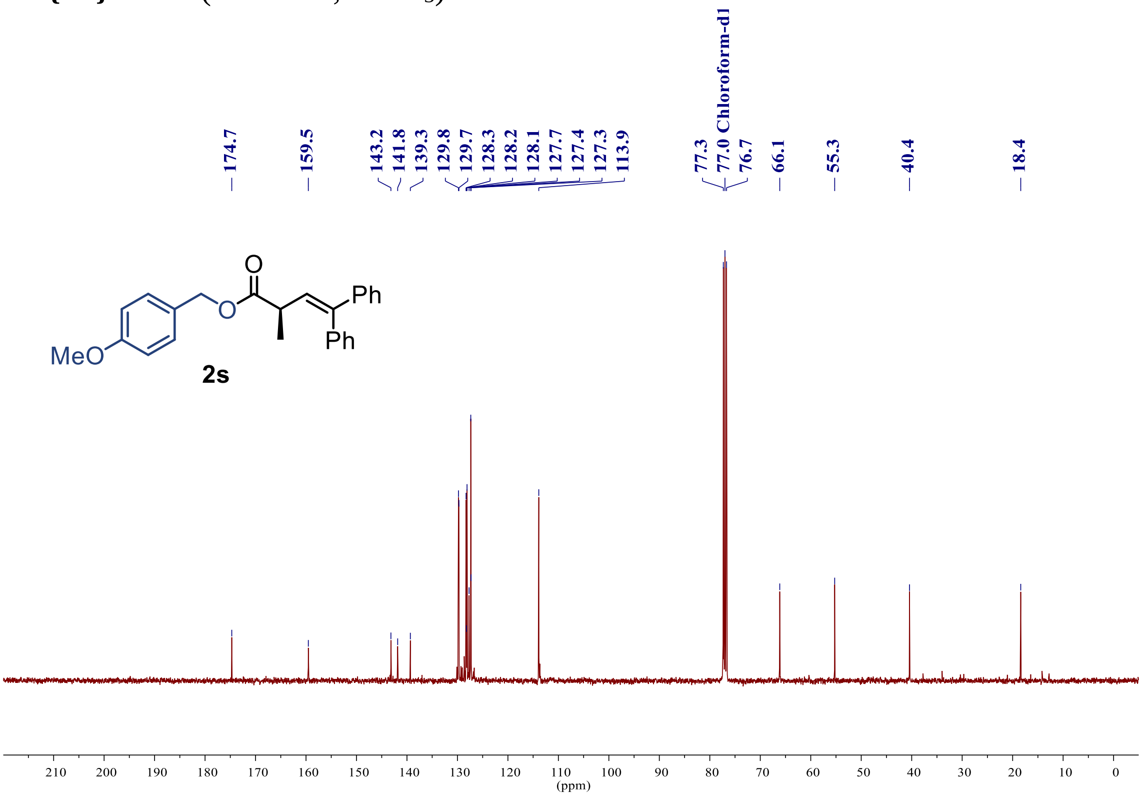

Back to contents

^{1}H NMR (400 MHz, $CDCl_3$) of **2t**, See procedure

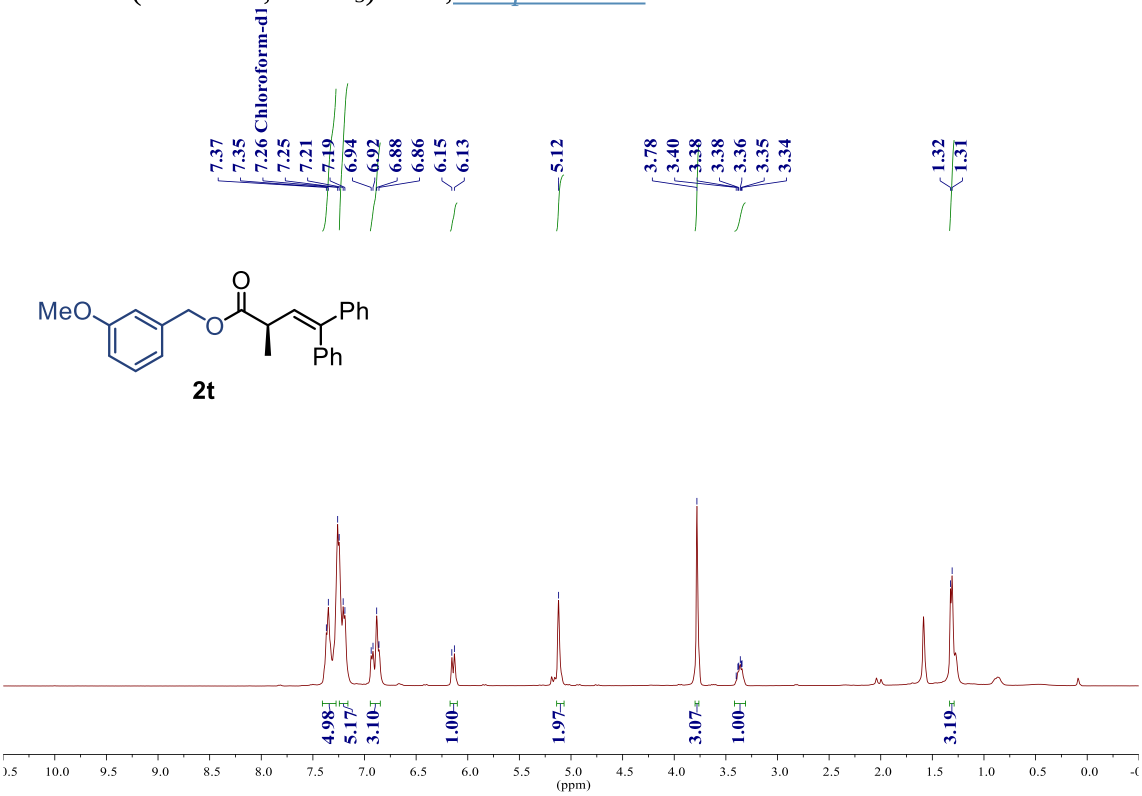

^{13}C{^{1}H} NMR (101 MHz, $CDCl_3$) of **2t**

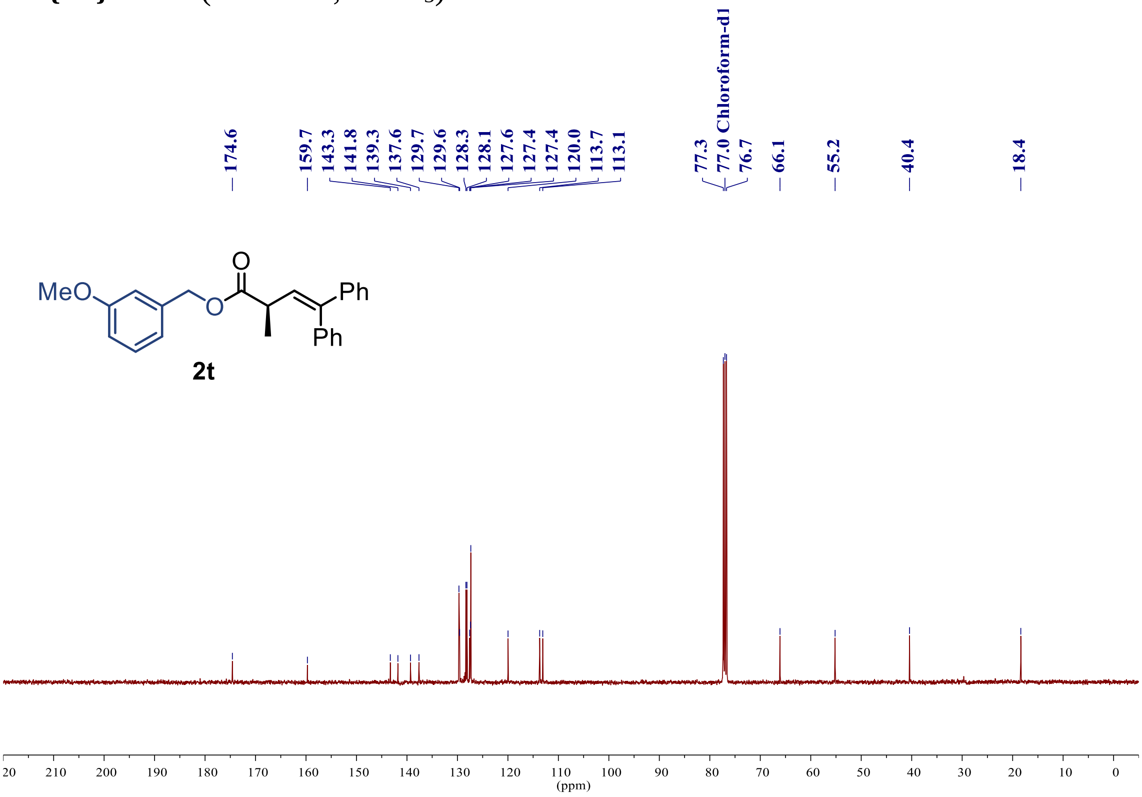

Back to contents

^{1}H NMR (300 MHz, $CDCl_3$) of **2u**, See procedure

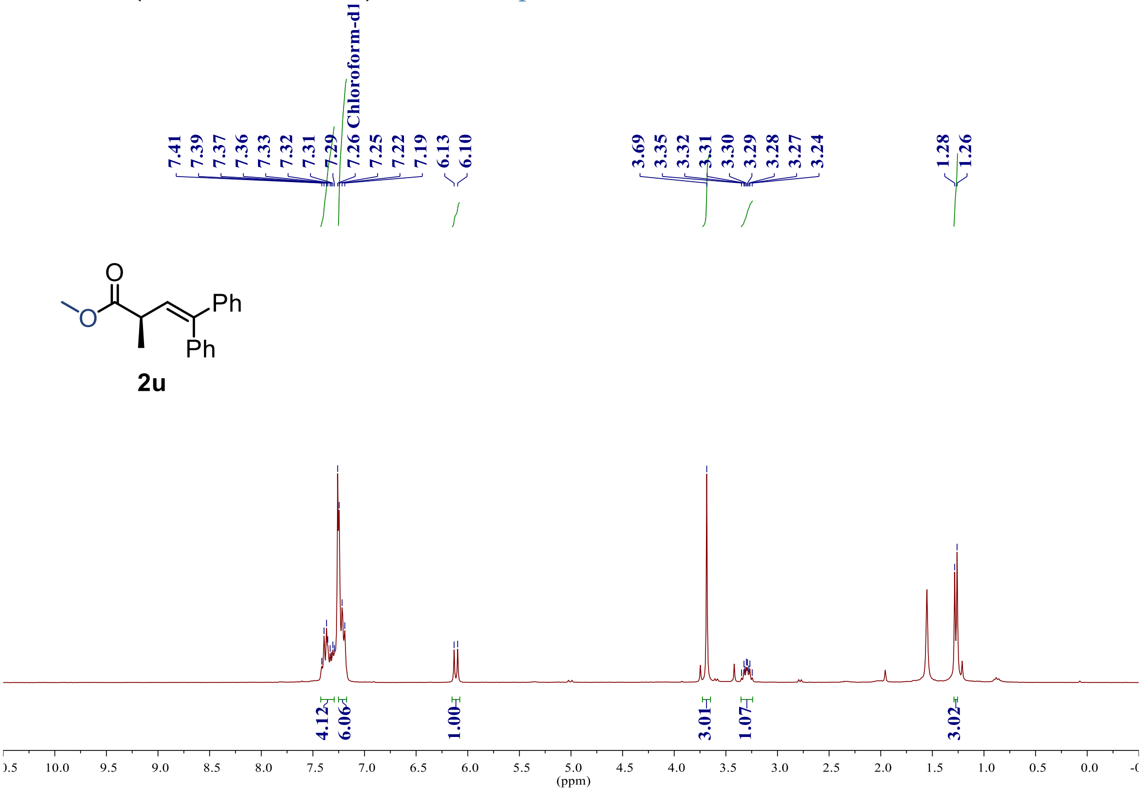

$^{13}C\{^1H\}$ NMR (101 MHz, $CDCl_3$) of **2u**

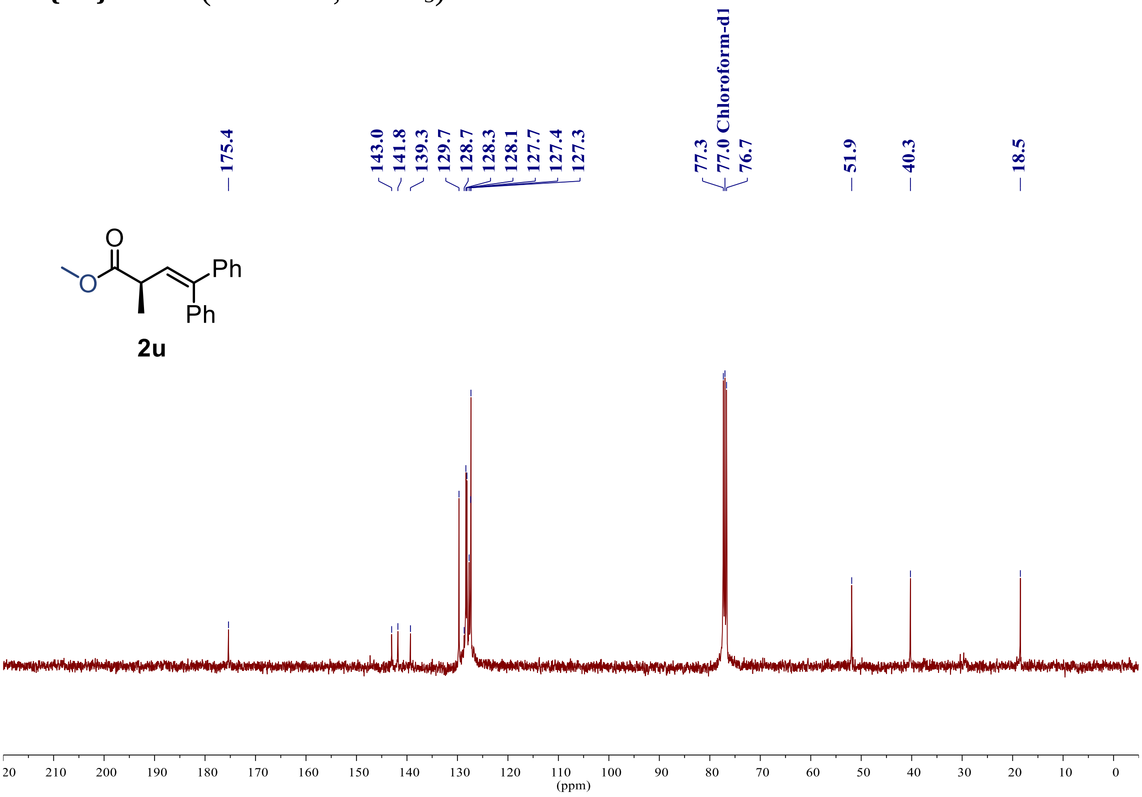

[Back to contents](#)

^{1}H NMR (400 MHz, $CDCl_3$) of **2v**, See procedure

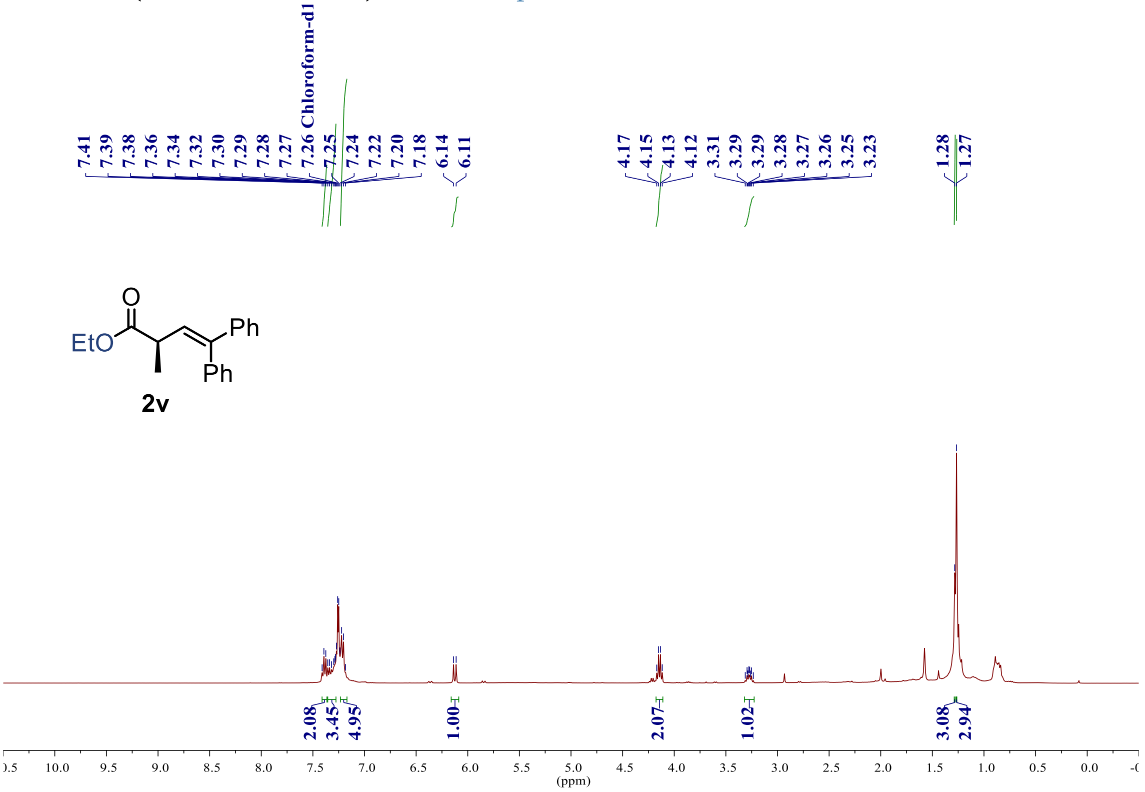

^{13}C{^{1}H} NMR (101 MHz, $CDCl_3$) of **2v**

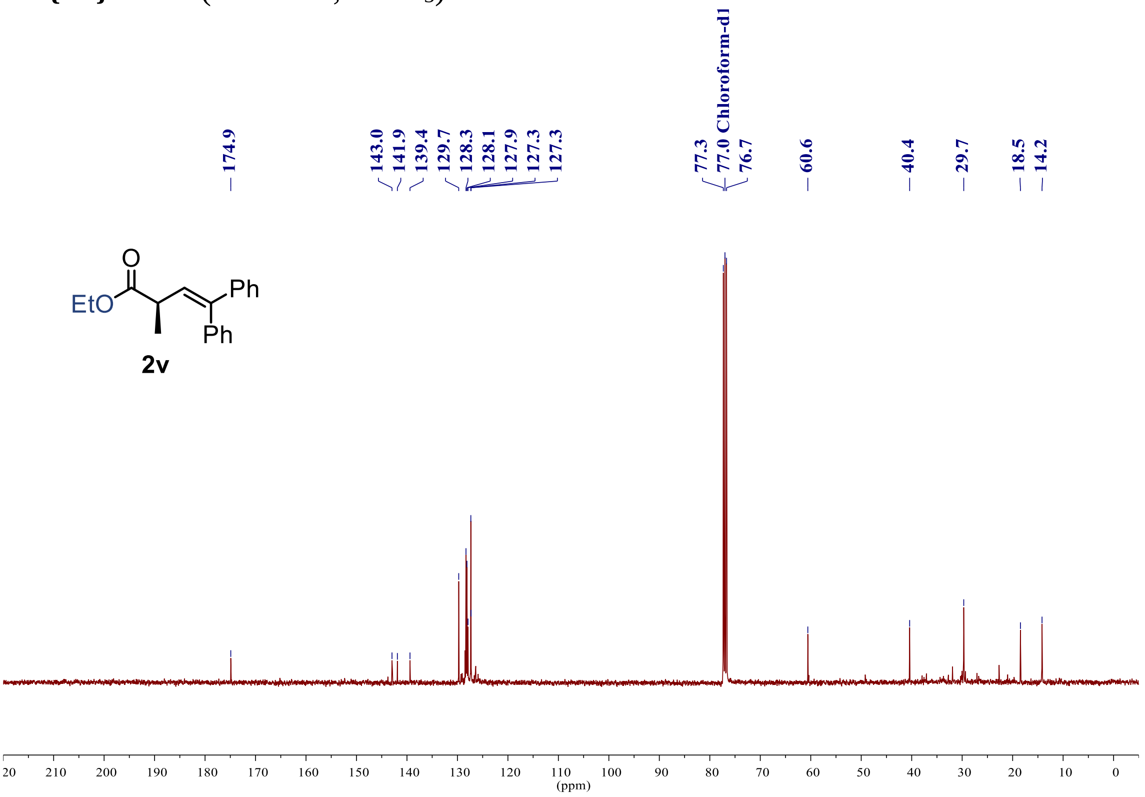

Back to contents

^{1}H NMR (300 MHz, $CDCl_3$) of **2w**, See procedure

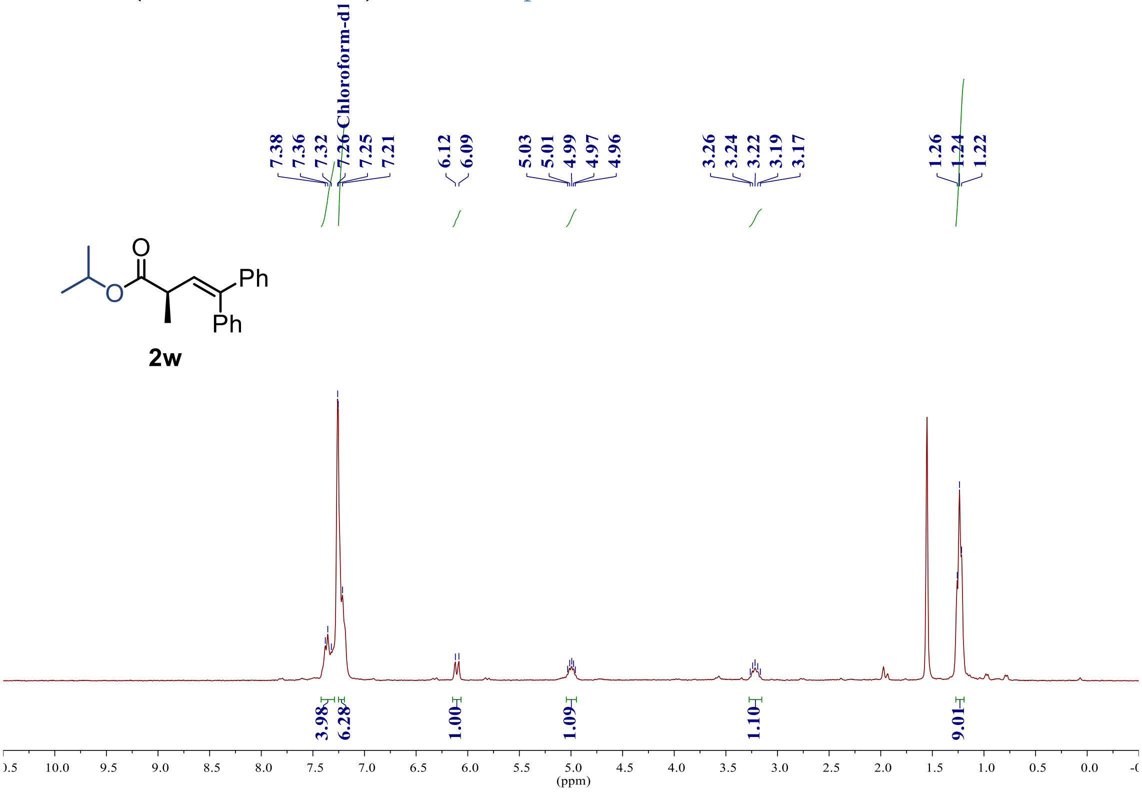

$^{13}C\{^1H\}$ NMR (101 MHz, $CDCl_3$) of **2w**

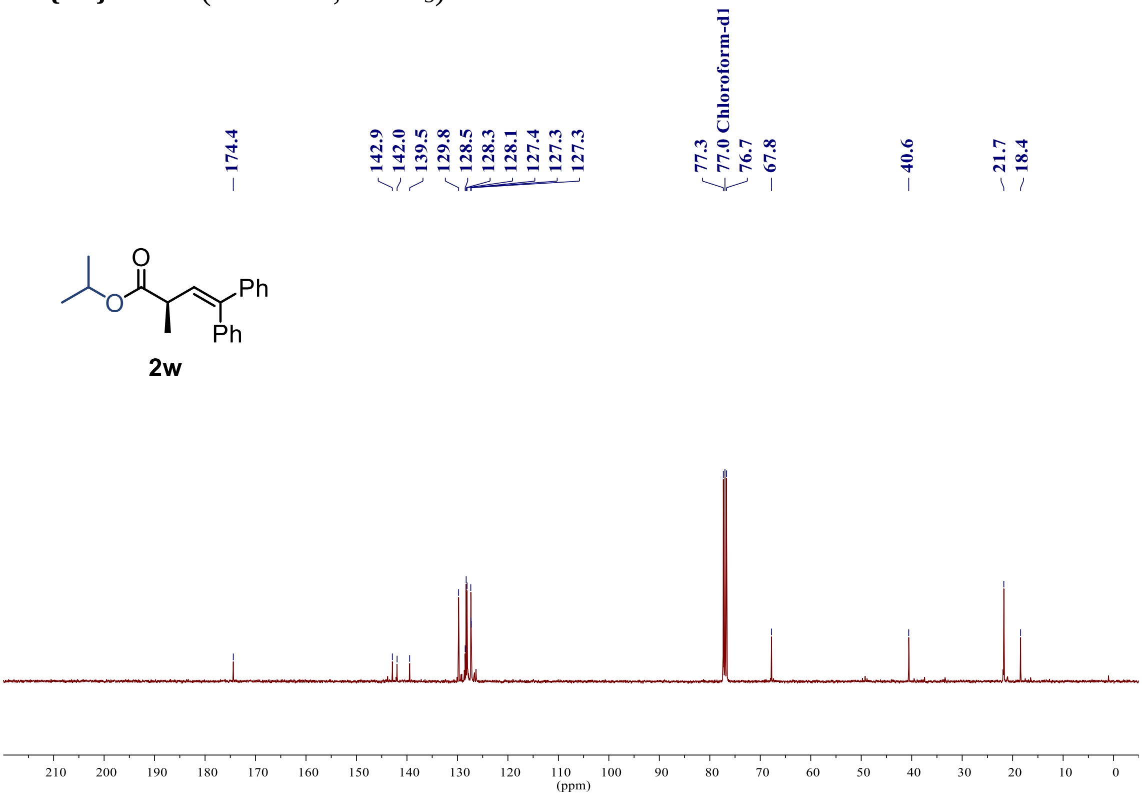

Back to contents

^{1}H NMR (300 MHz, $CDCl_3$) of **2x**, See procedure

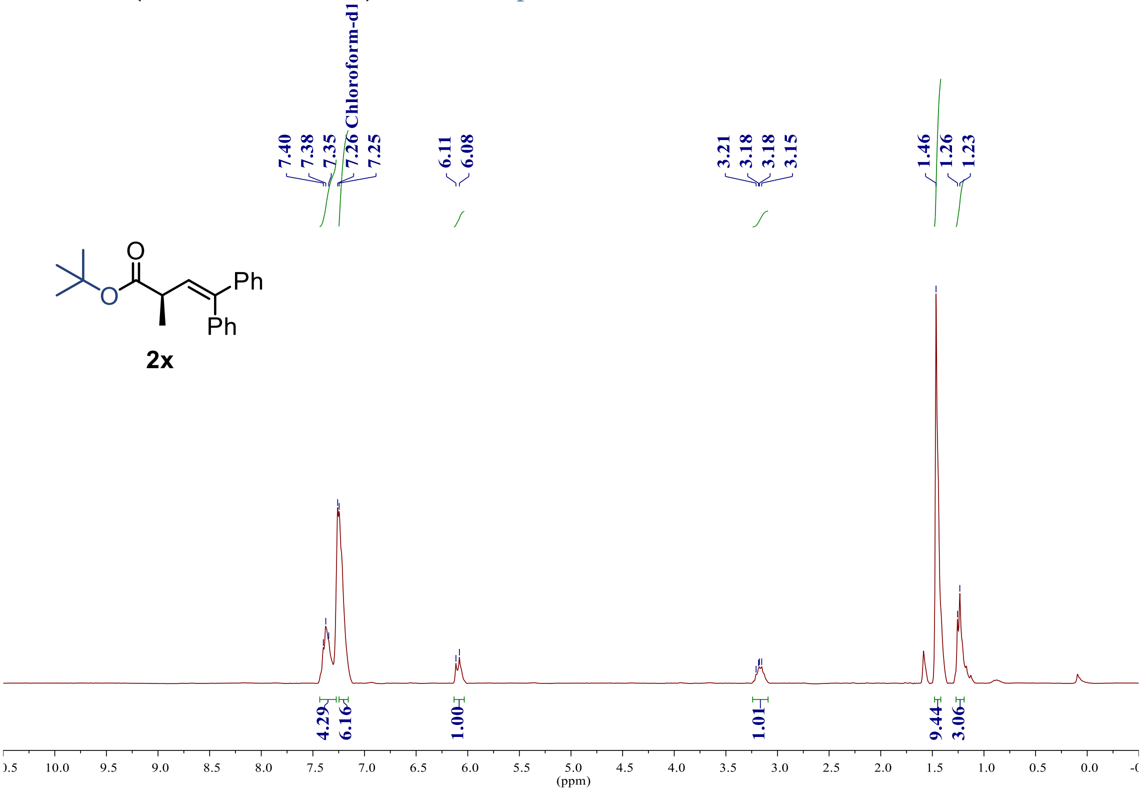

^{13}C{^{1}H} NMR (101 MHz, $CDCl_3$) of **2x**

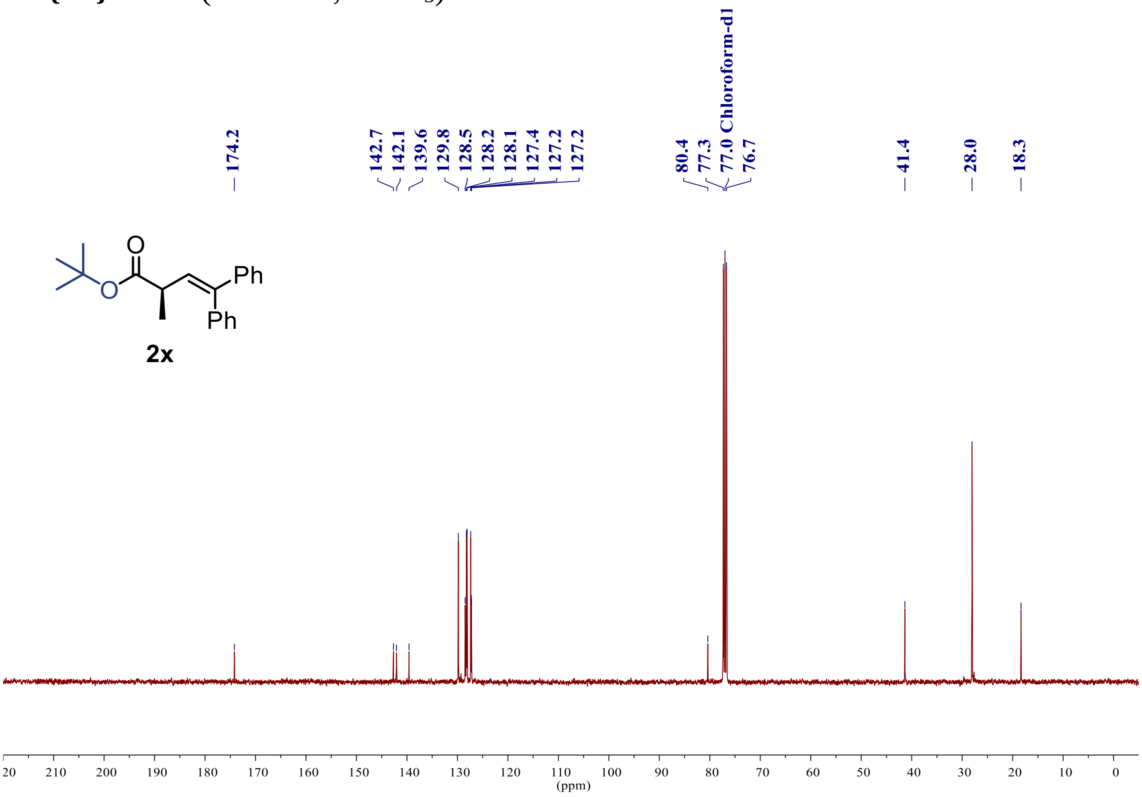

Back to contents

¹H NMR (400 MHz, $CDCl_3$) of **2y**, See procedure

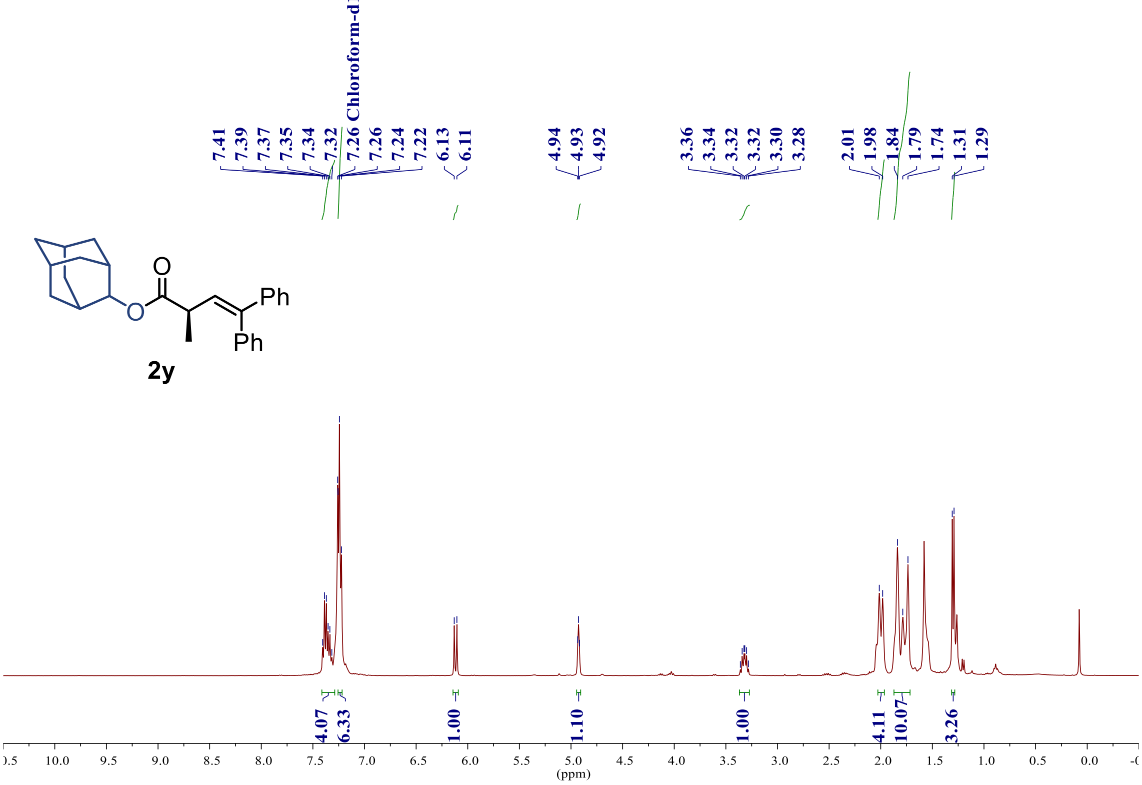

¹³C{¹H} NMR (101 MHz, $CDCl_3$) of **2y**

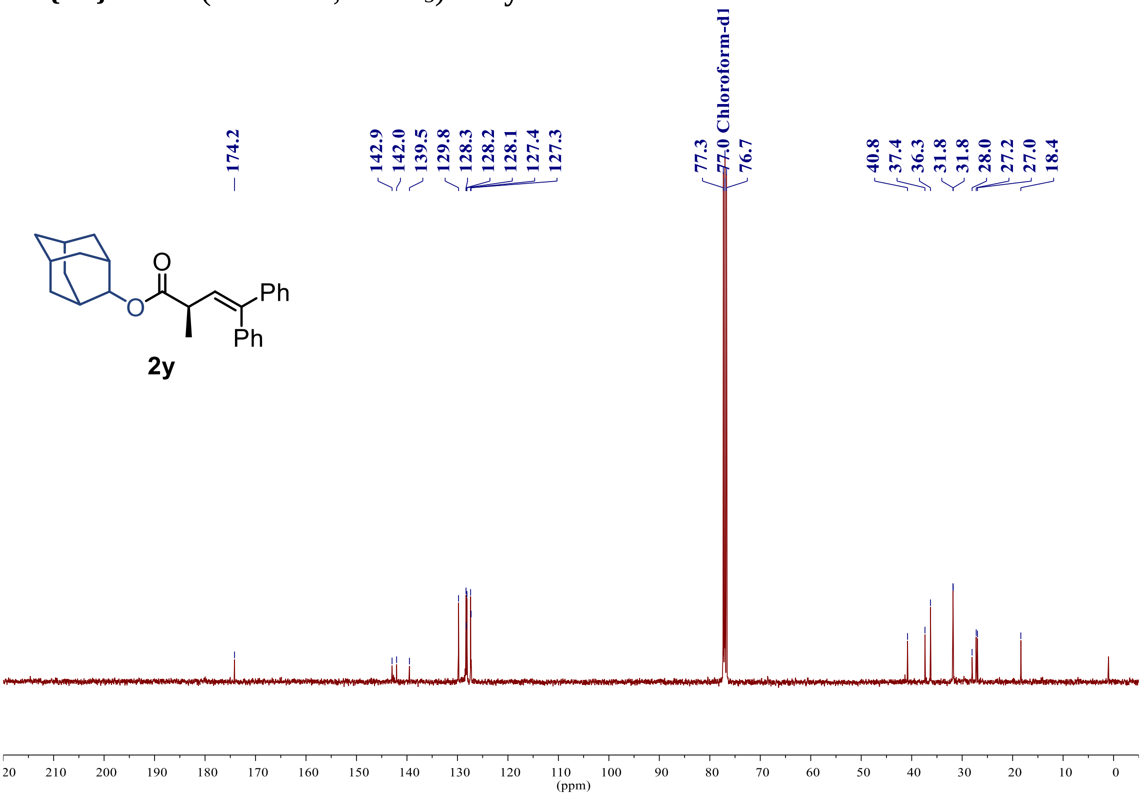

[Back to contents](#)

^{1}H NMR (400 MHz, $CDCl_3$) of **2z**, See procedure

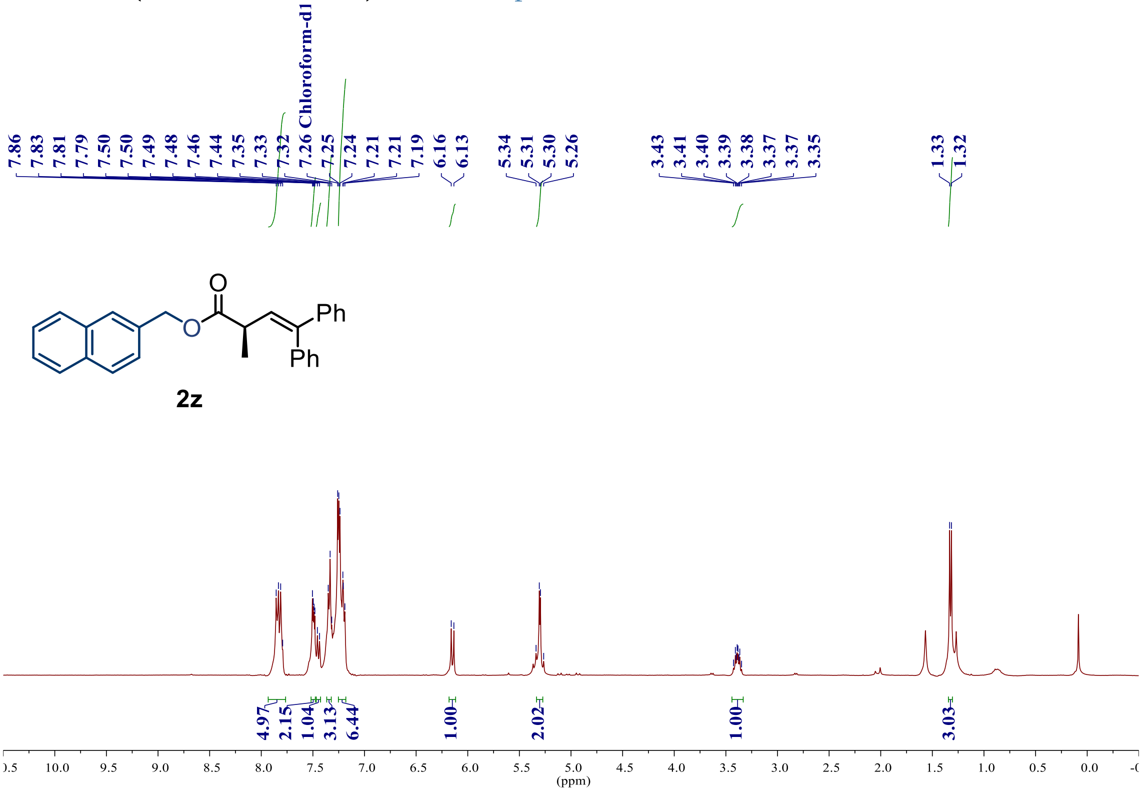

^{13}C{^{1}H} NMR (101 MHz, $CDCl_3$) of **2z**

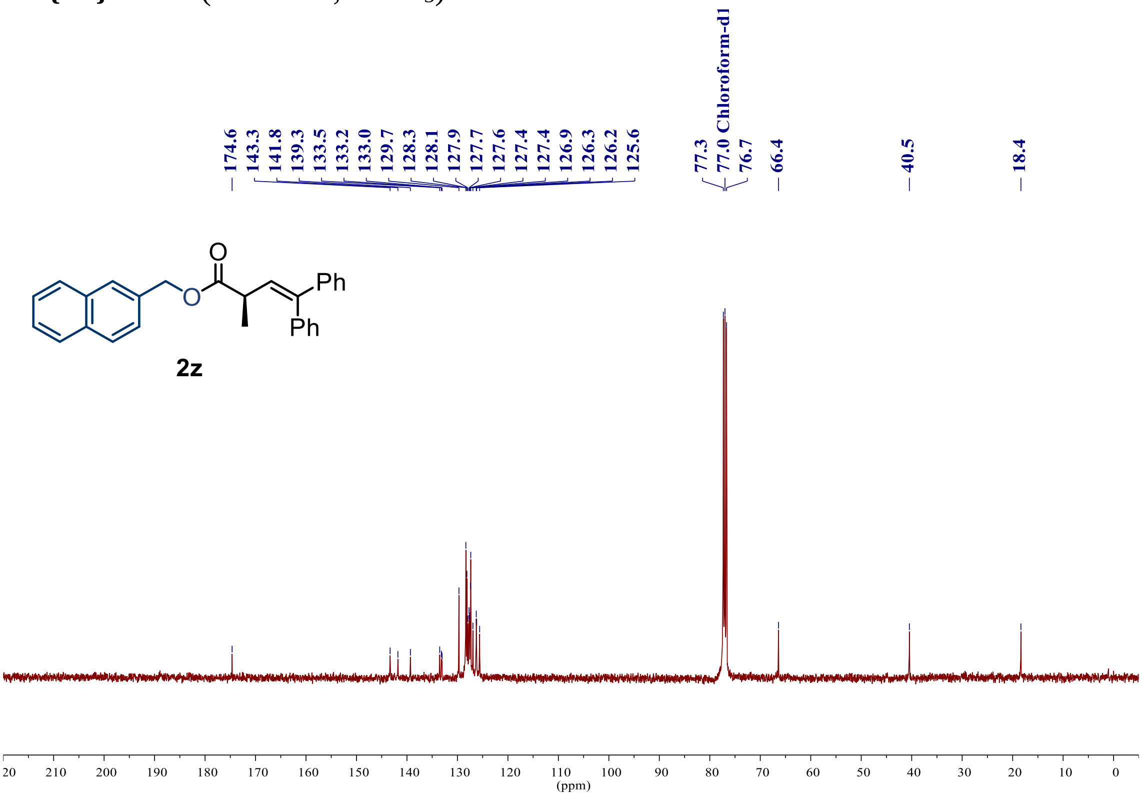

Back to contents

^{1}H NMR (400 MHz, $CDCl_3$) of **2aa**, See procedure

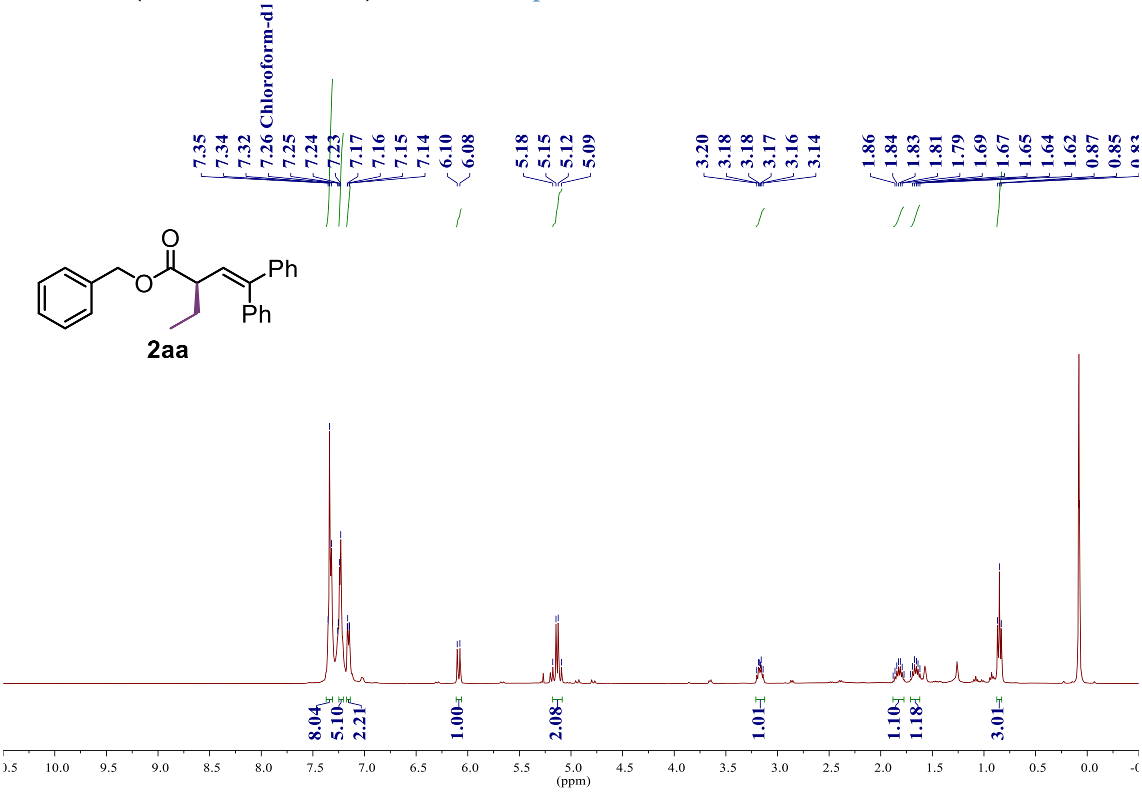

^{13}C{^{1}H} NMR (101 MHz, $CDCl_3$) of **2aa**

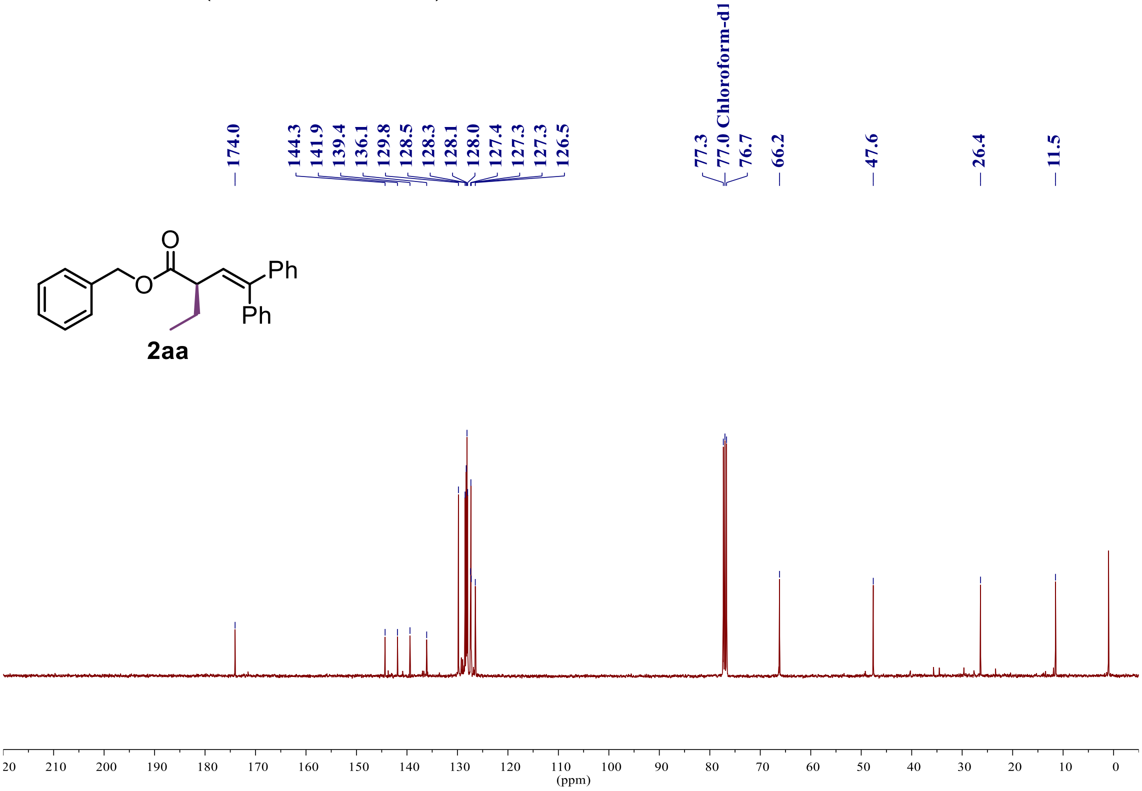

Back to contents

^{1}H NMR (400 MHz, $CDCl_3$) of **2ab**, See procedure

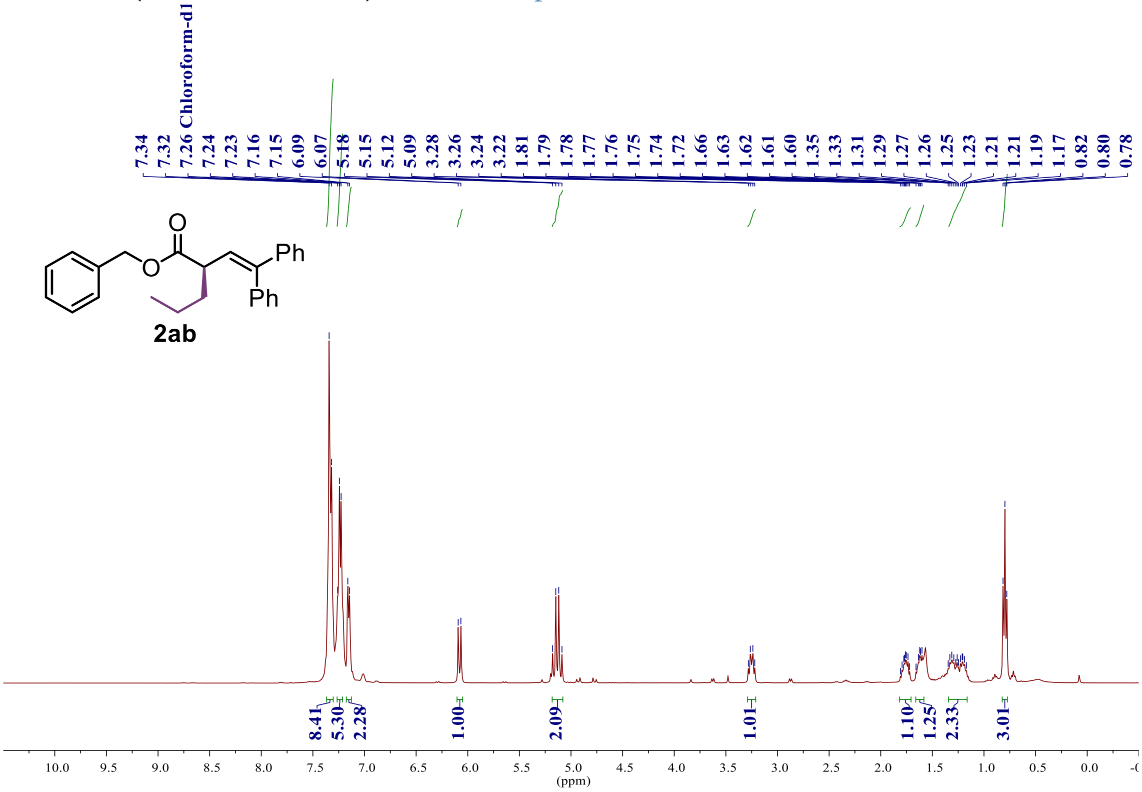

^{13}C{^{1}H} NMR (101 MHz, $CDCl_3$) of **2ab**

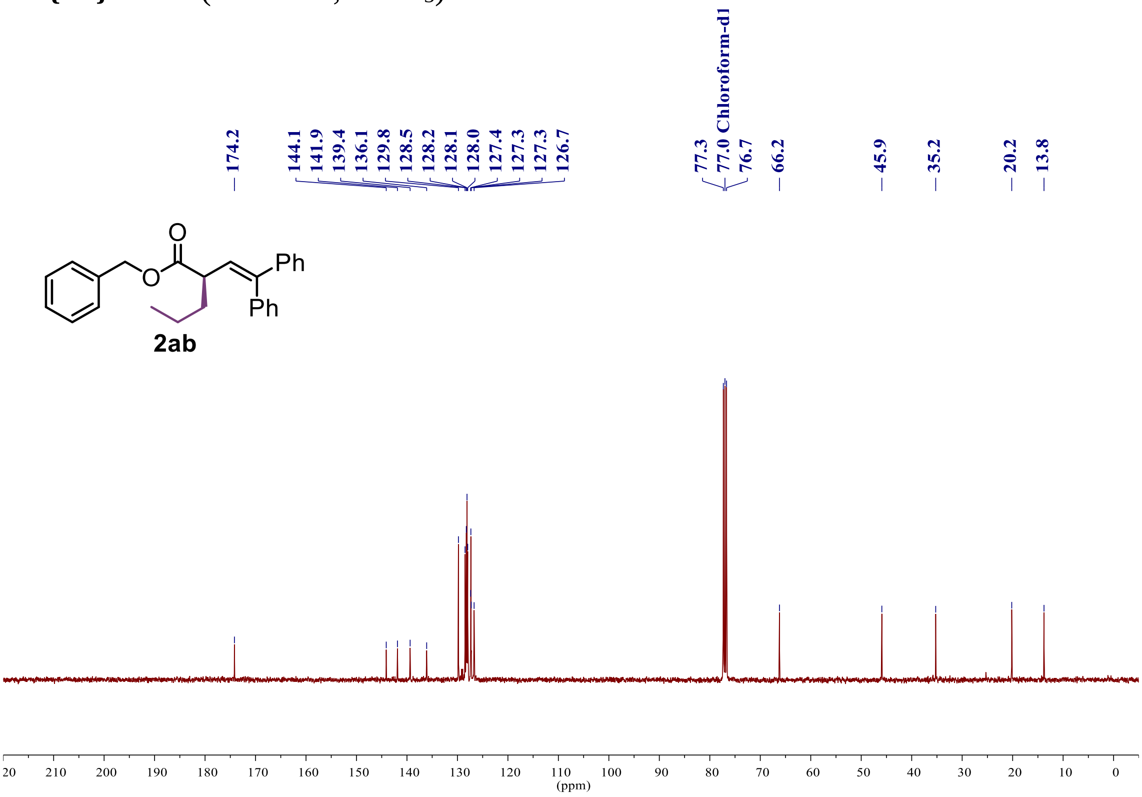

Back to contents

^{1}H NMR (400 MHz, $CDCl_3$) of **2ac**, See procedure

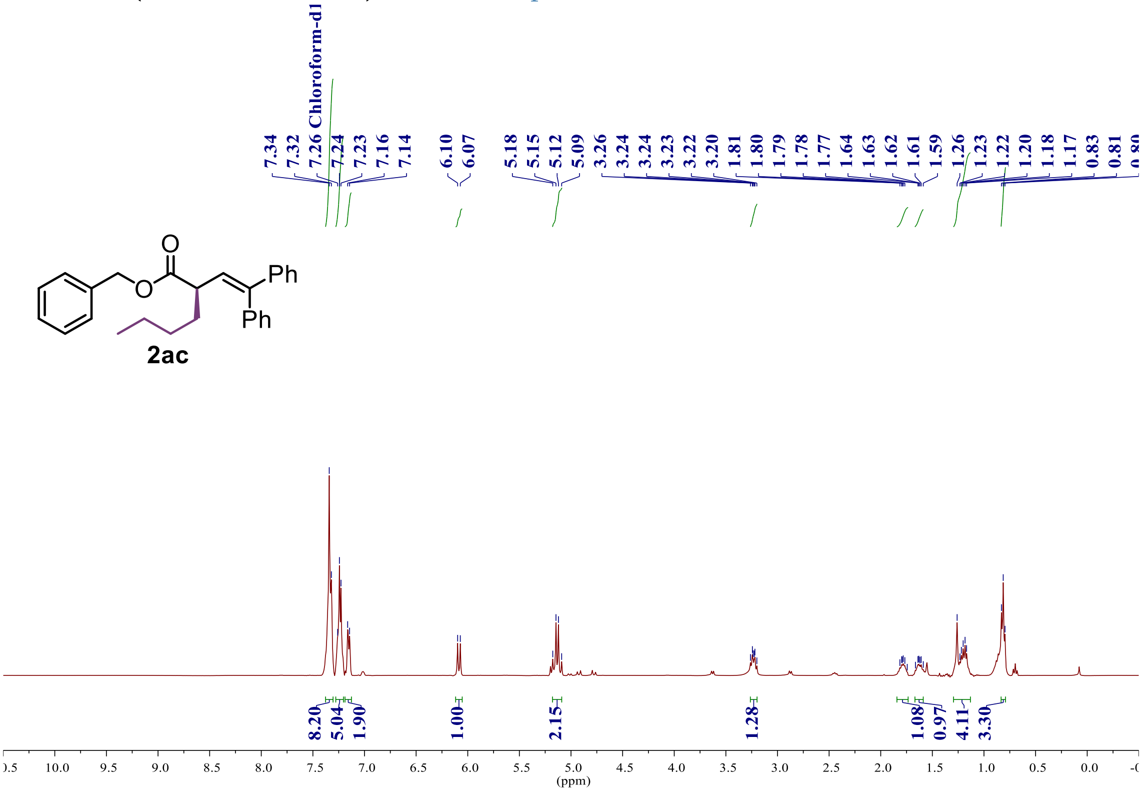

^{13}C{^{1}H} NMR (101 MHz, $CDCl_3$) of **2ac**

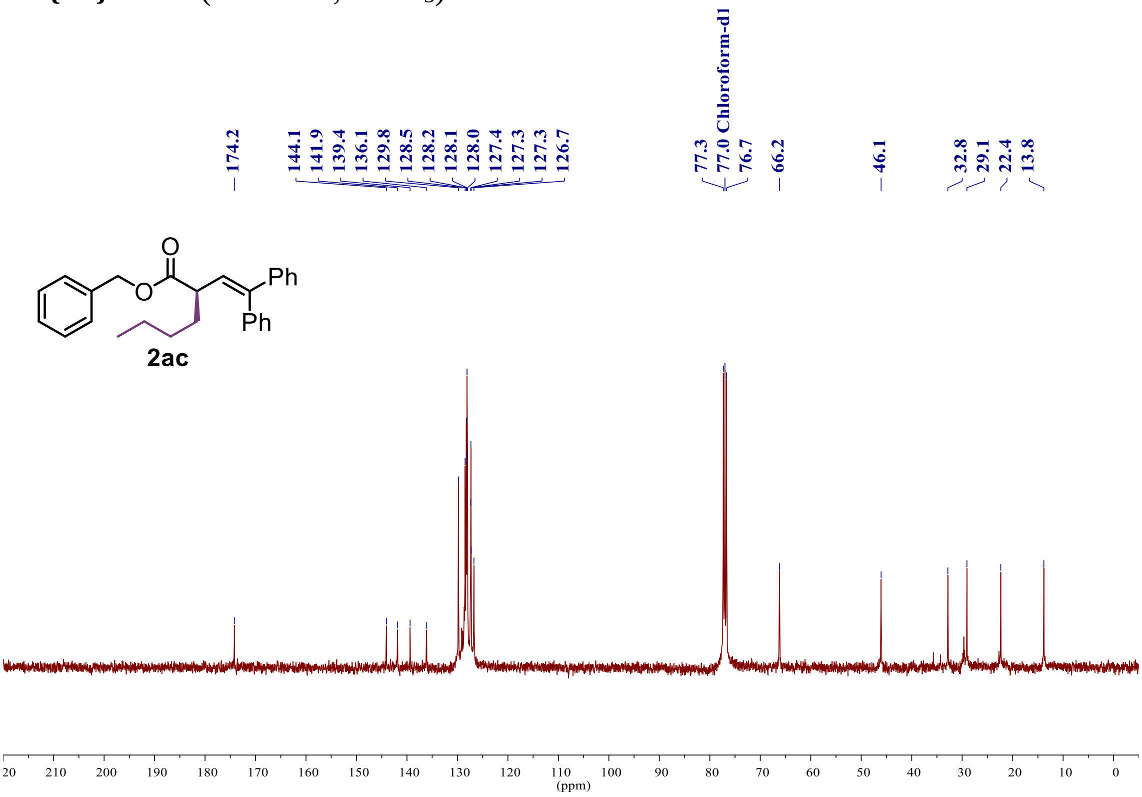

Back to contents

^{1}H NMR (400 MHz, $CDCl_3$) of **2ad**, See procedure

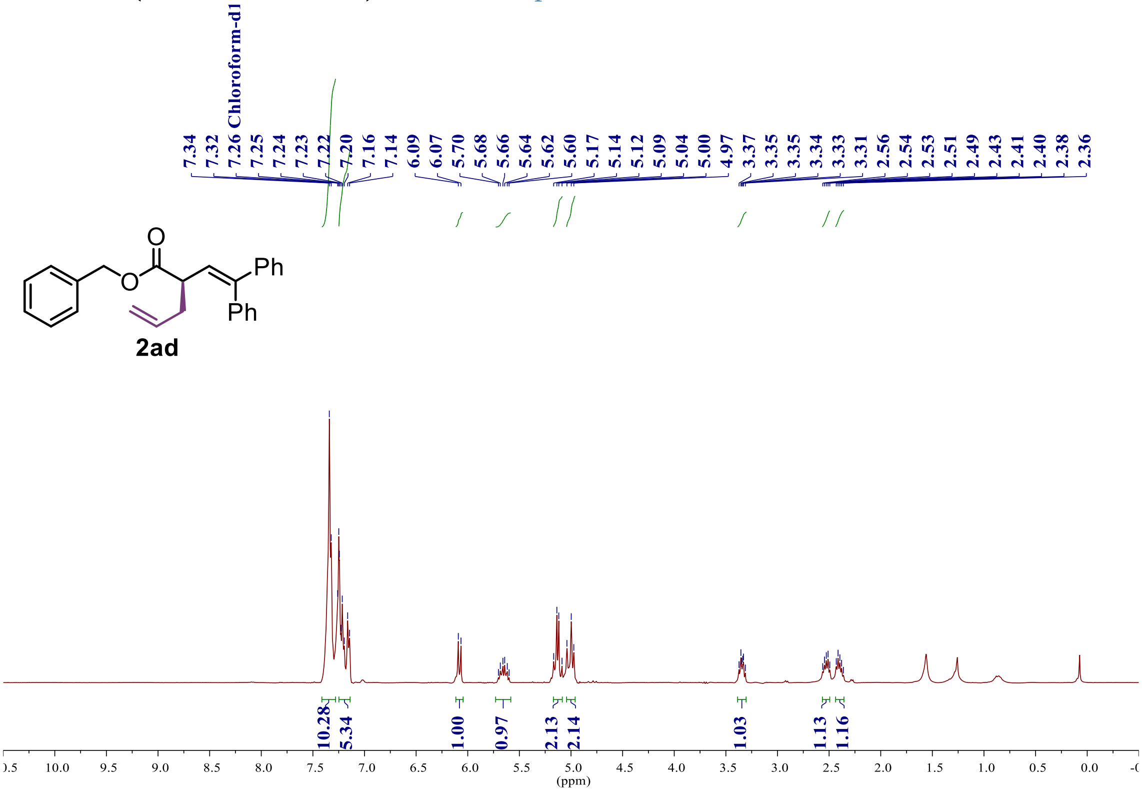

^{13}C{^{1}H} NMR (101 MHz, $CDCl_3$) of **2ad**

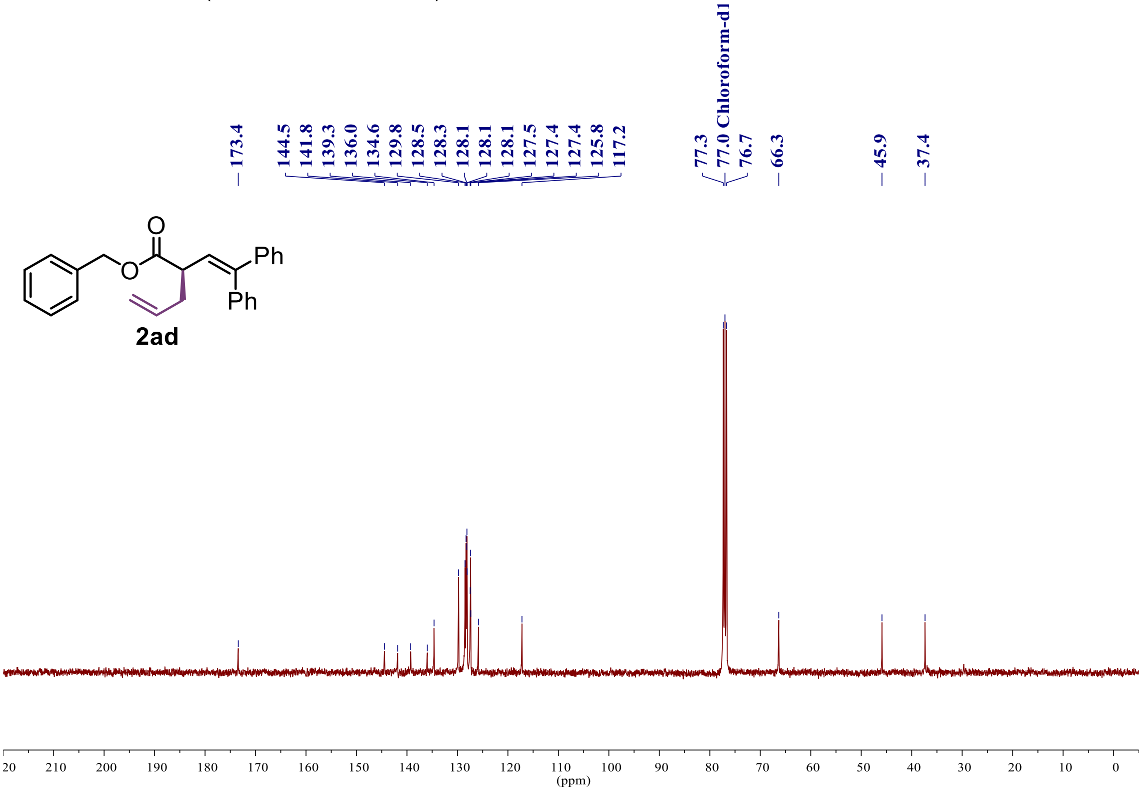

Back to contents

¹H NMR (400 MHz, $CDCl_3$) of **2ae**, See procedure

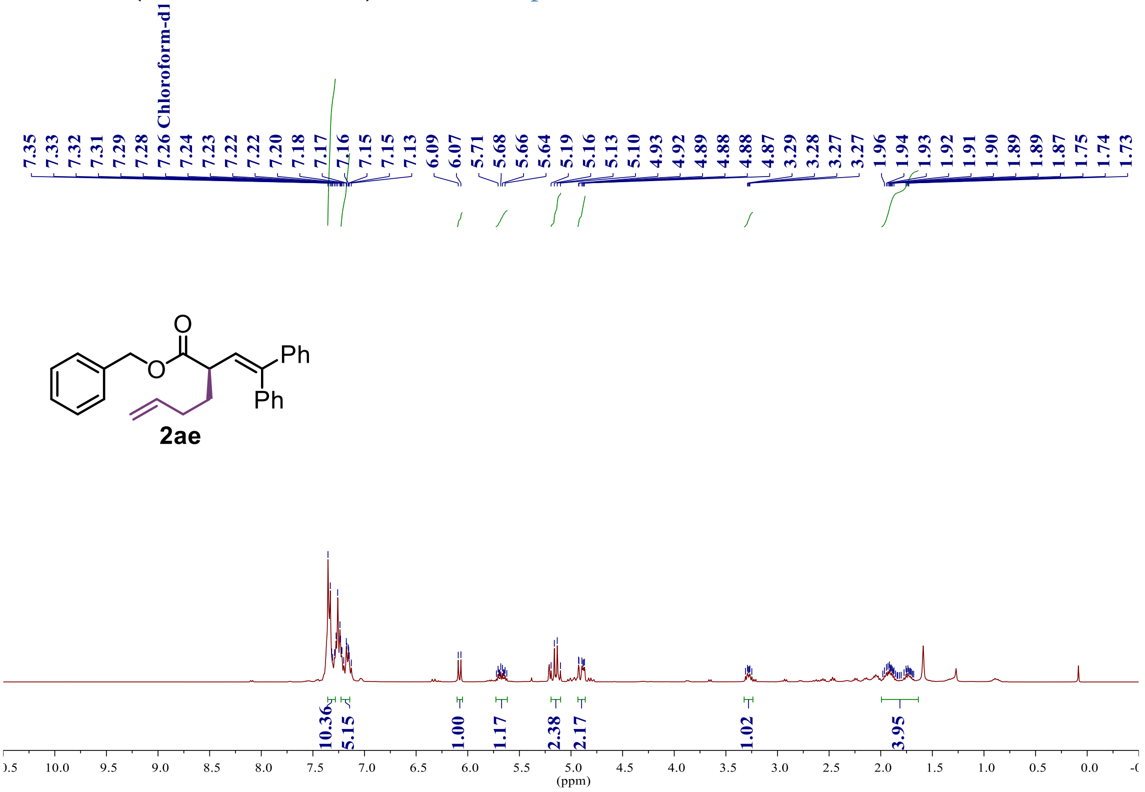

¹³C{¹H} NMR (151 MHz, $CDCl_3$) of **2ae**

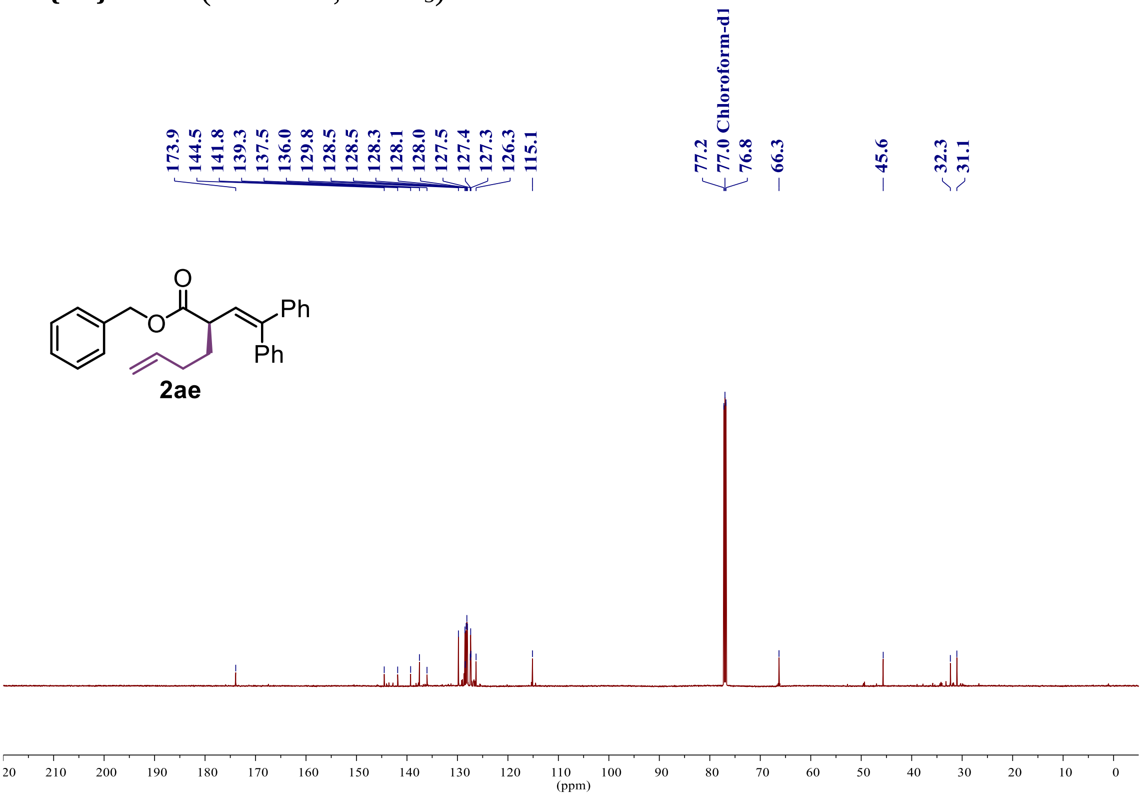

[Back to contents](#)

¹H NMR (600 MHz, $CDCl_3$) of **2af**, See procedure

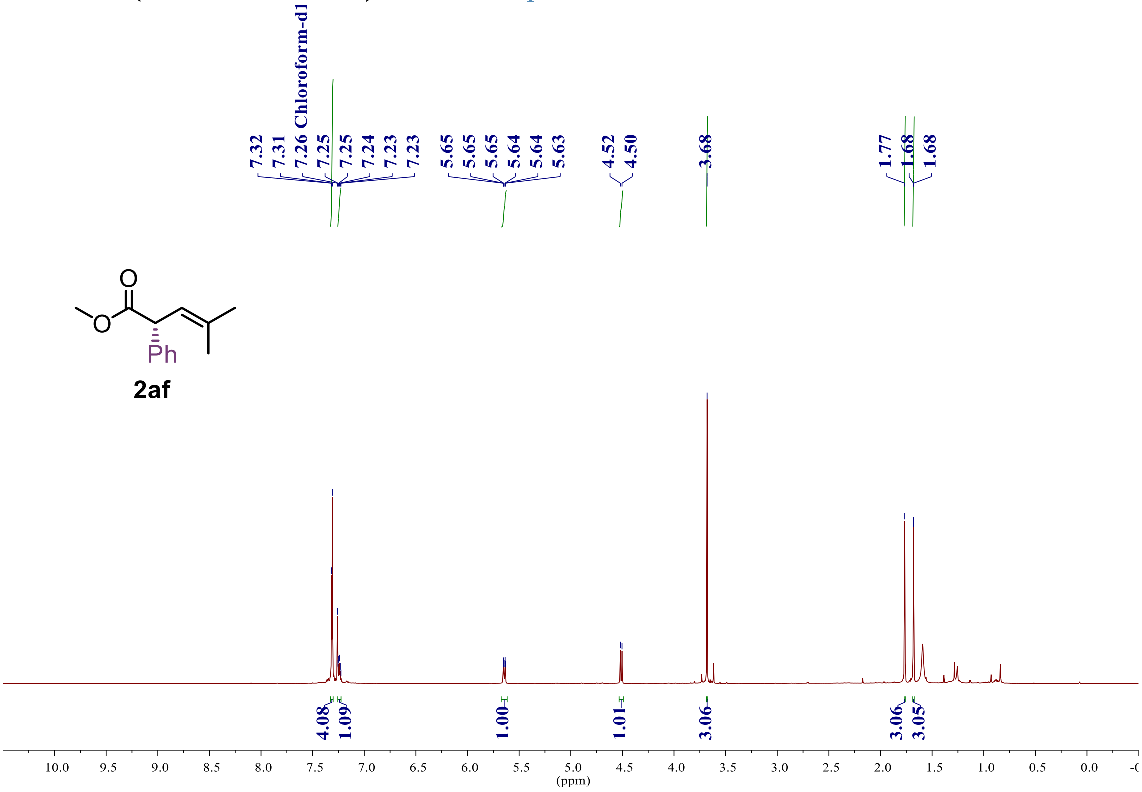

¹³C{¹H} NMR (151 MHz, $CDCl_3$) of **2af**

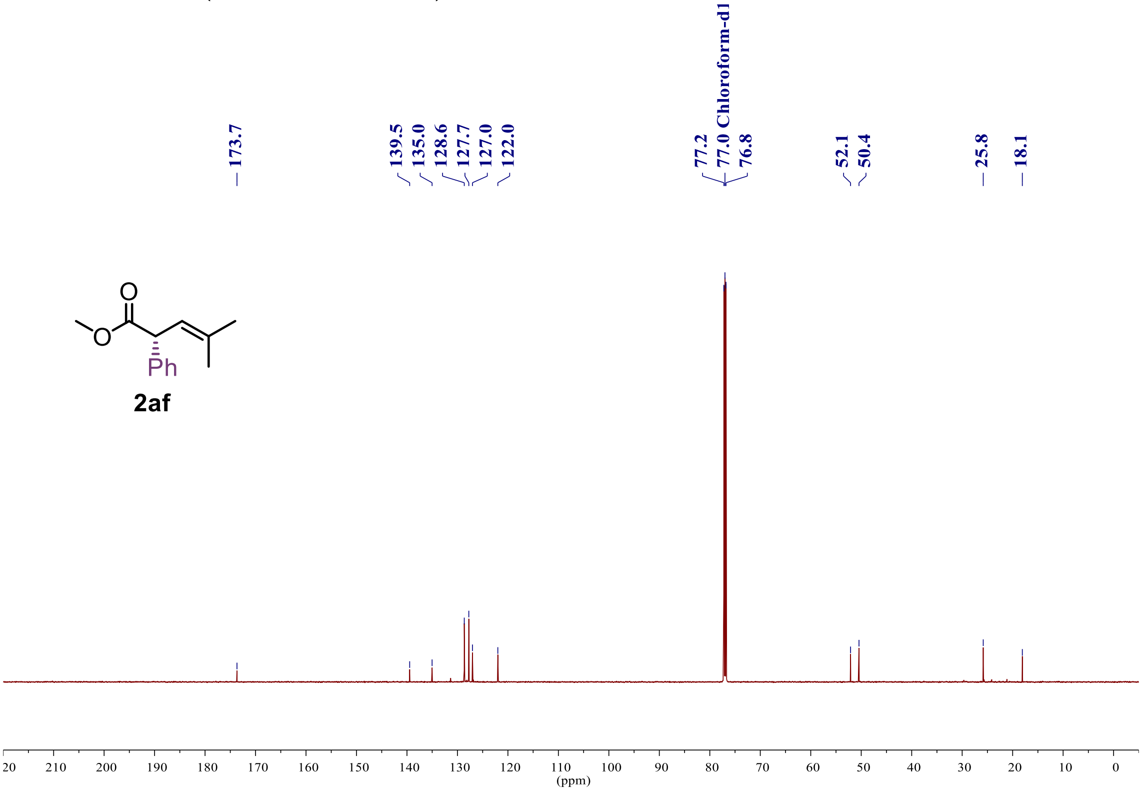

Back to contents

^{1}H NMR (600 MHz, $CDCl_3$) of **2ag**, See procedure

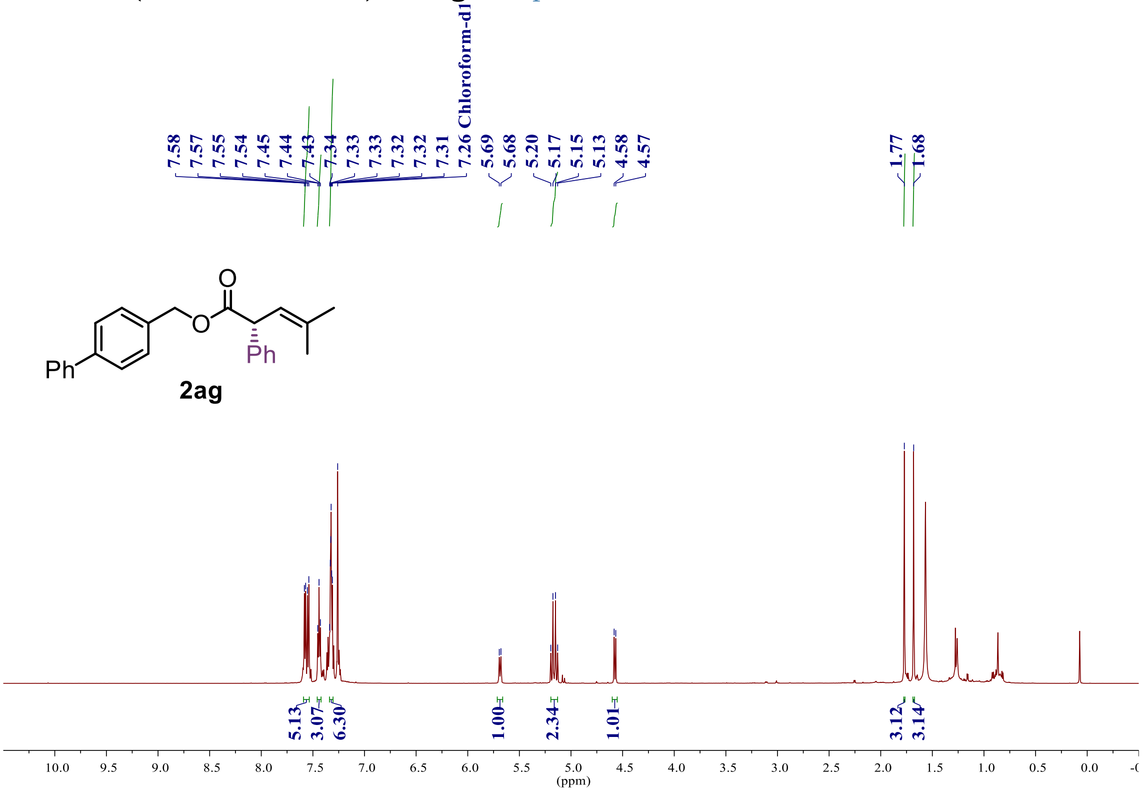

^{13}C{^{1}H} NMR (151 MHz, $CDCl_3$) of **2ag**

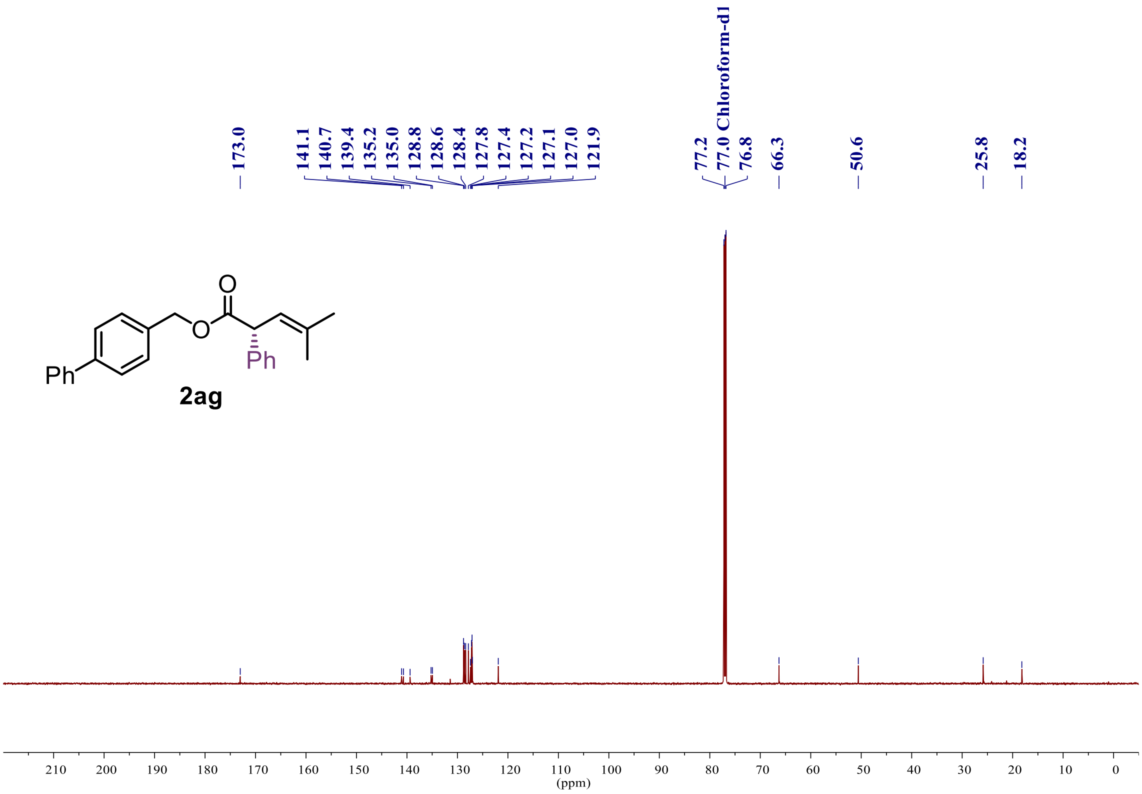

[Back to contents](#)

^{1}H NMR (600 MHz, $CDCl_3$) of **2ah**, See procedure

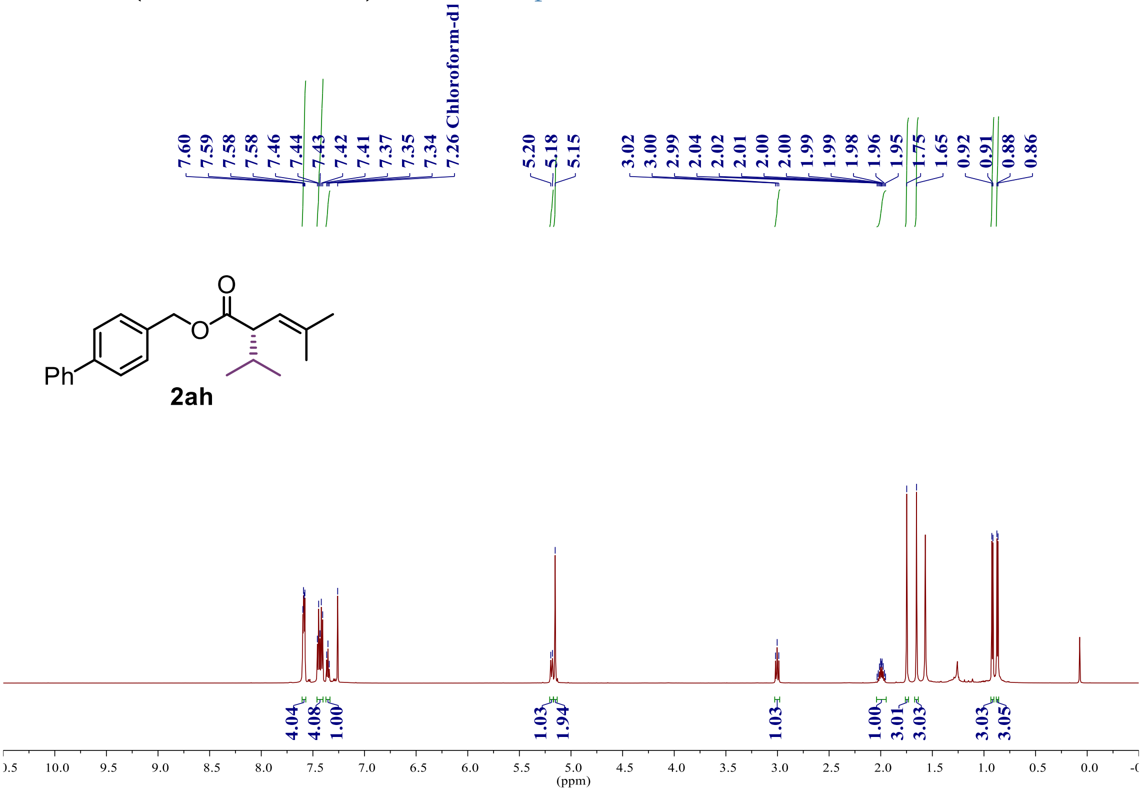

^{13}C{^{1}H} NMR (151 MHz, $CDCl_3$) of **2ah**

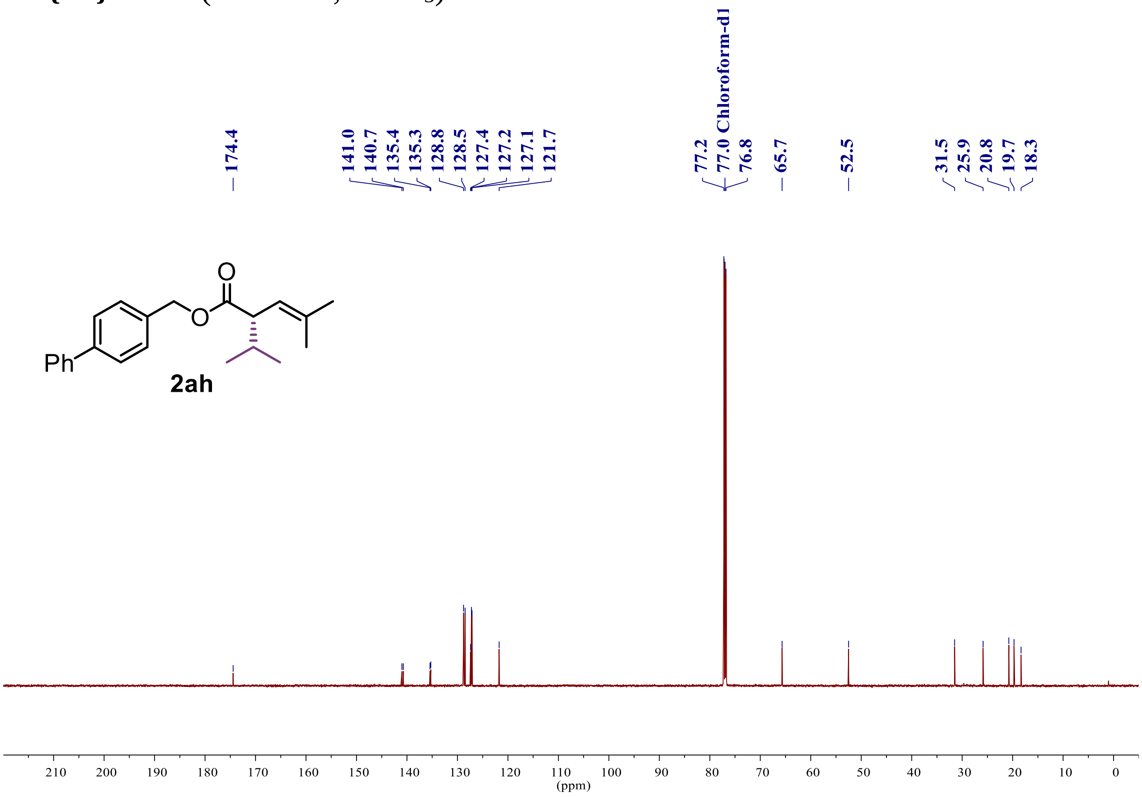

Back to contents

¹H NMR (600 MHz, $CDCl_3$) of **2ai**, See procedure

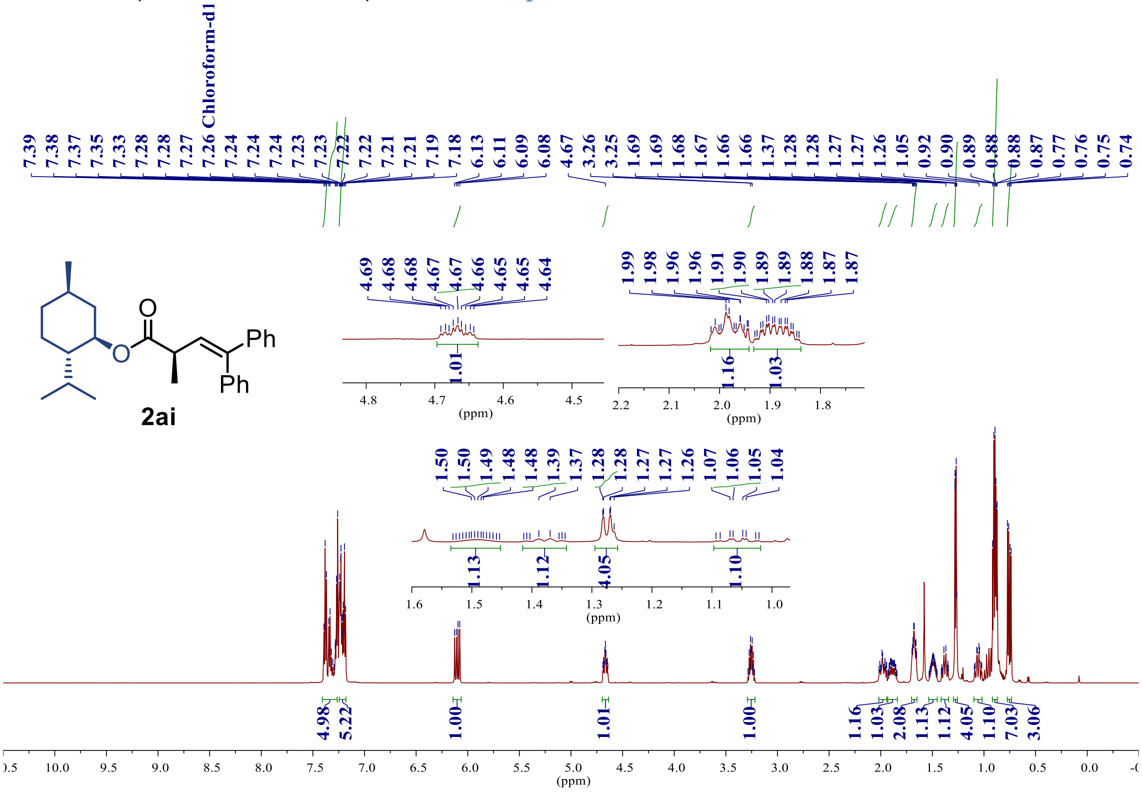

¹³C{¹H} NMR (151 MHz, $CDCl_3$) of **2ai**

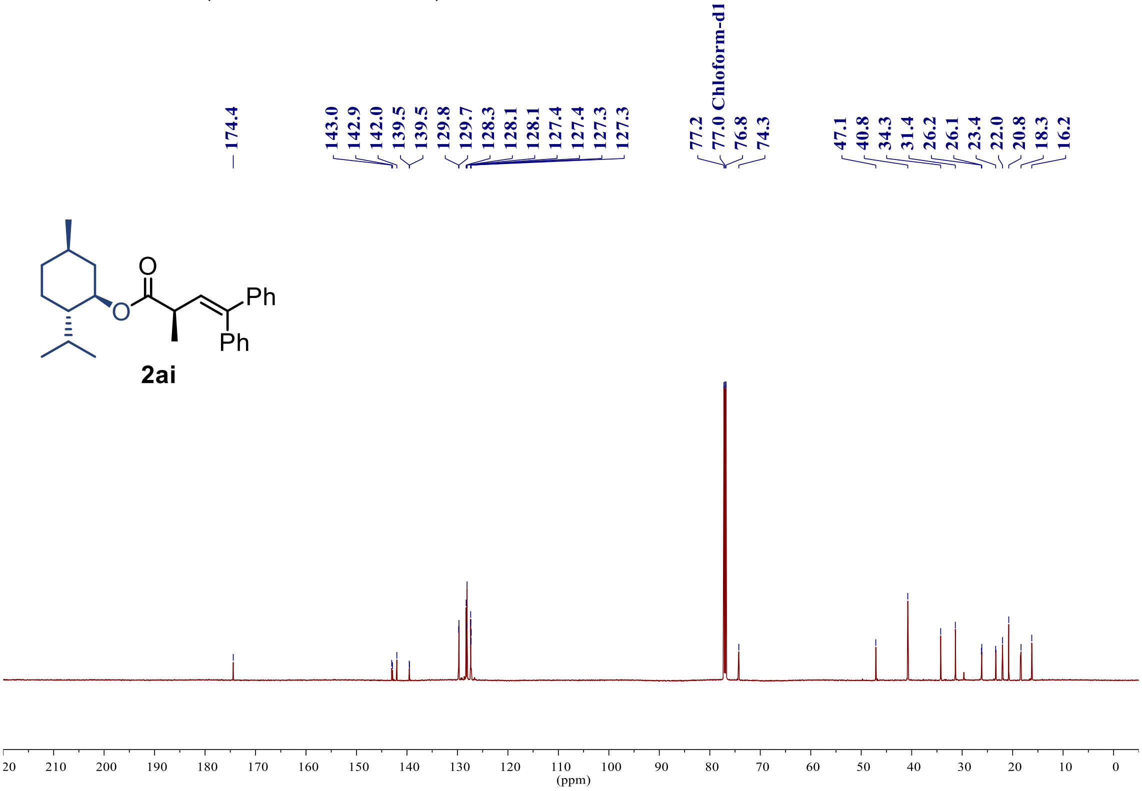

Back to contents

^{1}H NMR (600 MHz, $CDCl_3$) of **2aj**, See procedure

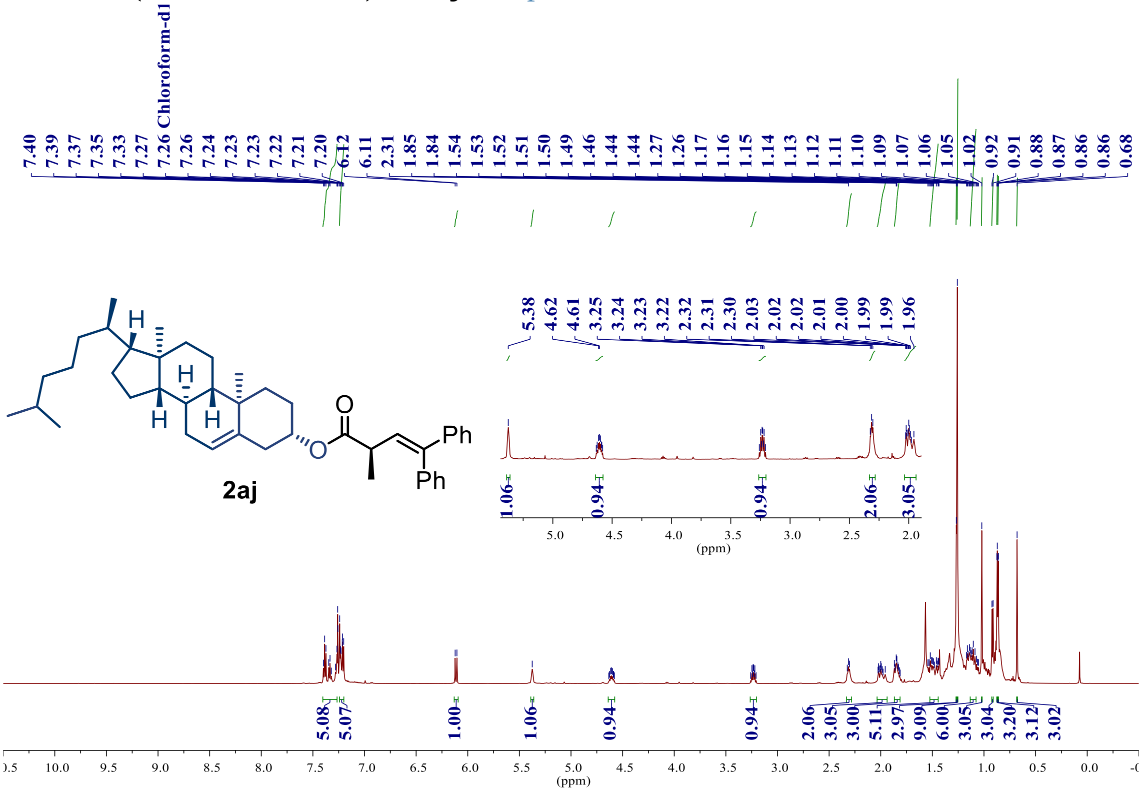

^{13}C{^{1}H} NMR (151 MHz, $CDCl_3$) of **2aj**

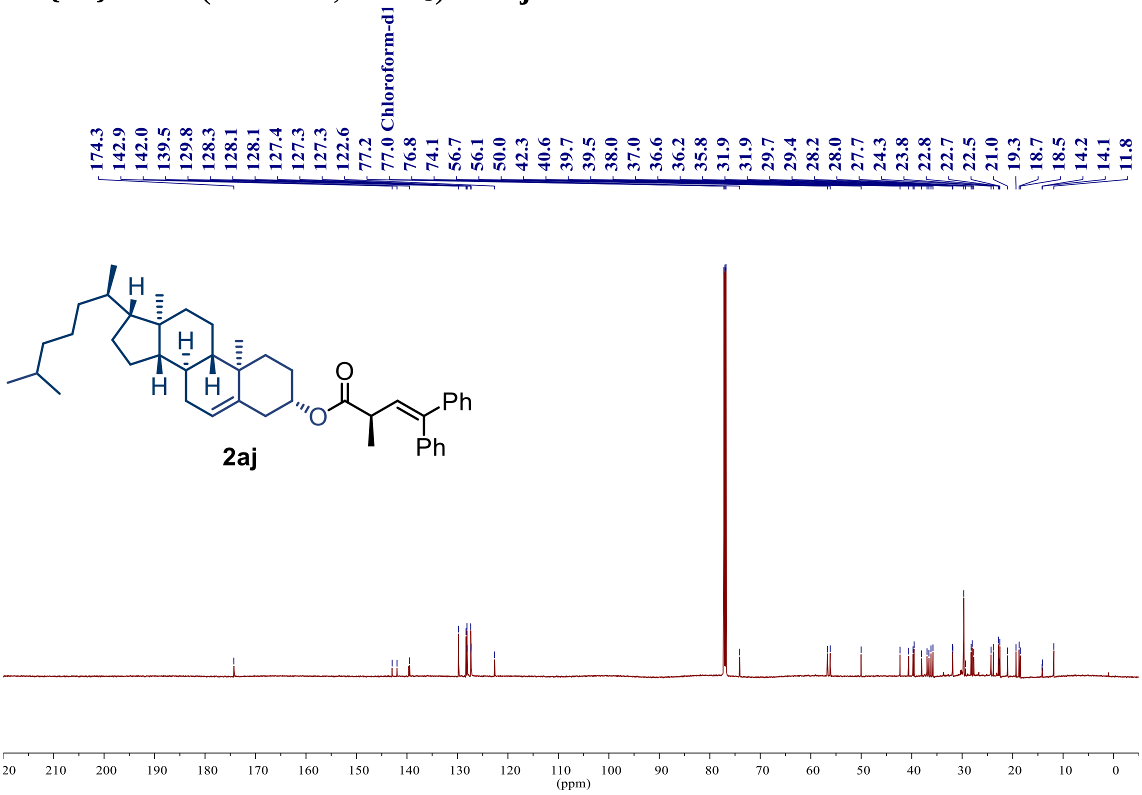

Back to contents

^{1}H NMR (600 MHz, $CDCl_3$) of **2ak**, See procedure

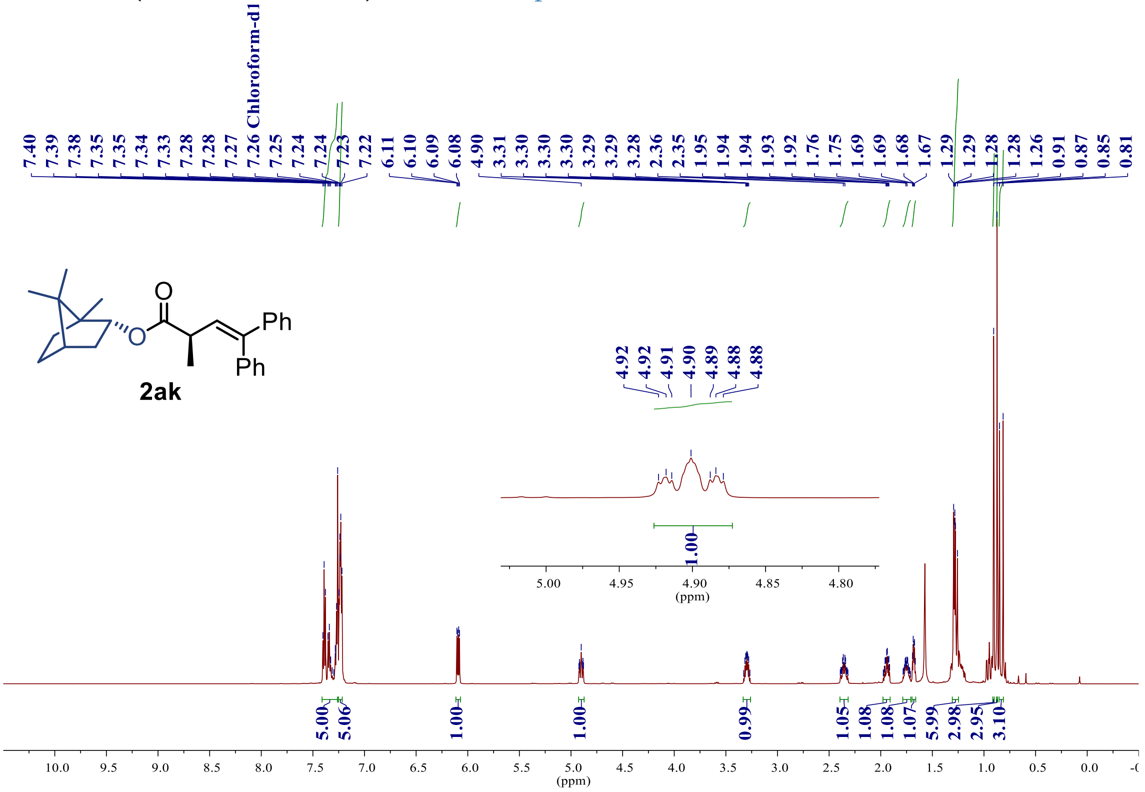

^{13}C{^{1}H} NMR (151 MHz, $CDCl_3$) of **2ak**

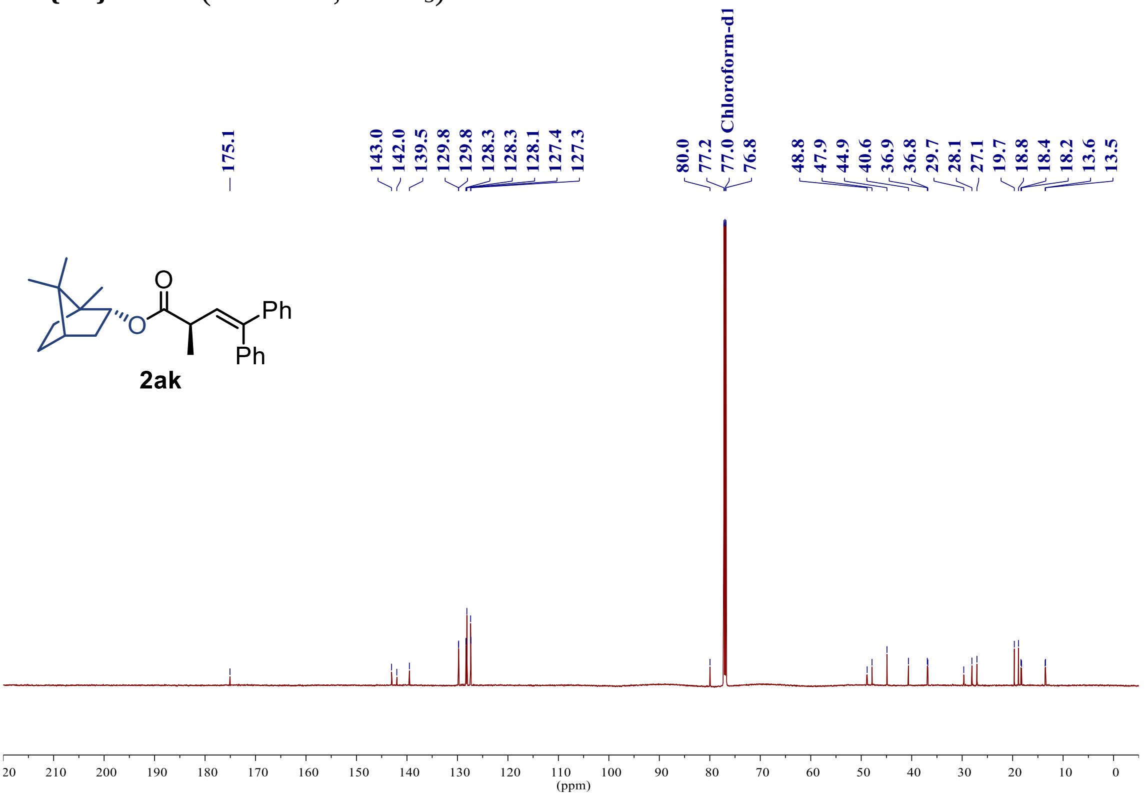

Back to contents

^{1}H NMR (400 MHz, $CDCl_3$) of **2al**, See procedure

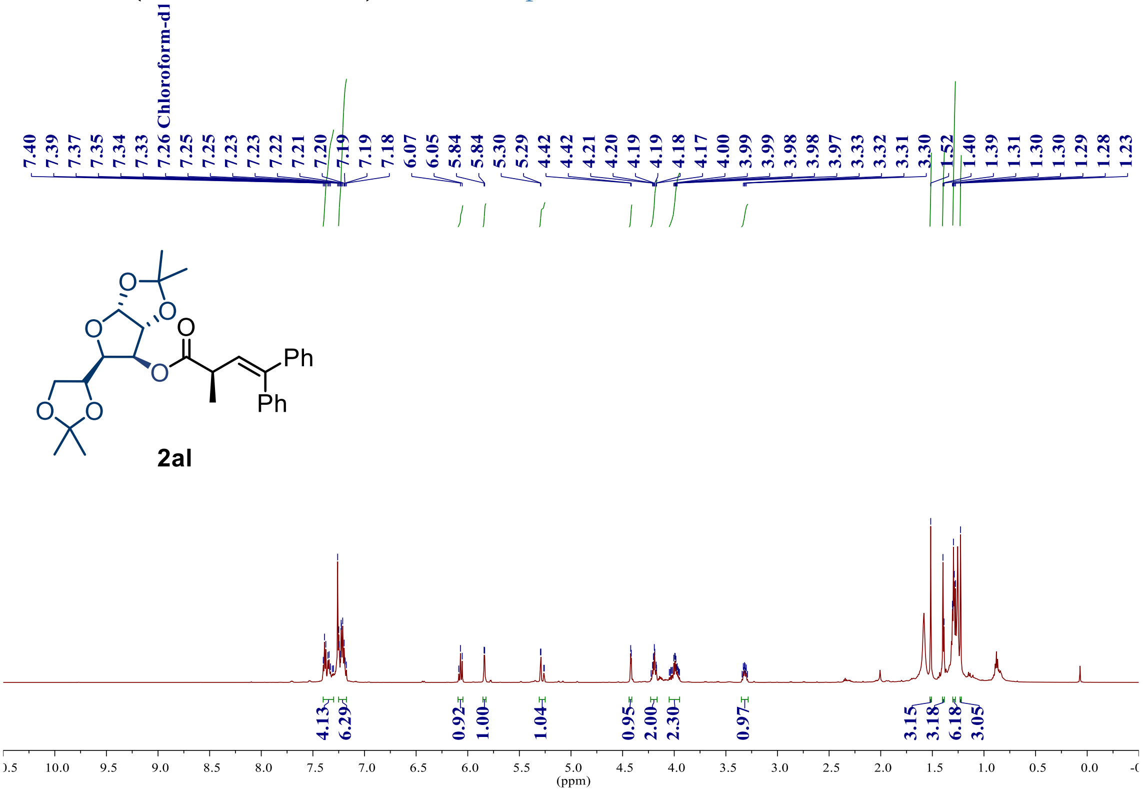

^{13}C{^{1}H} NMR (151 MHz, $CDCl_3$) of **2al**

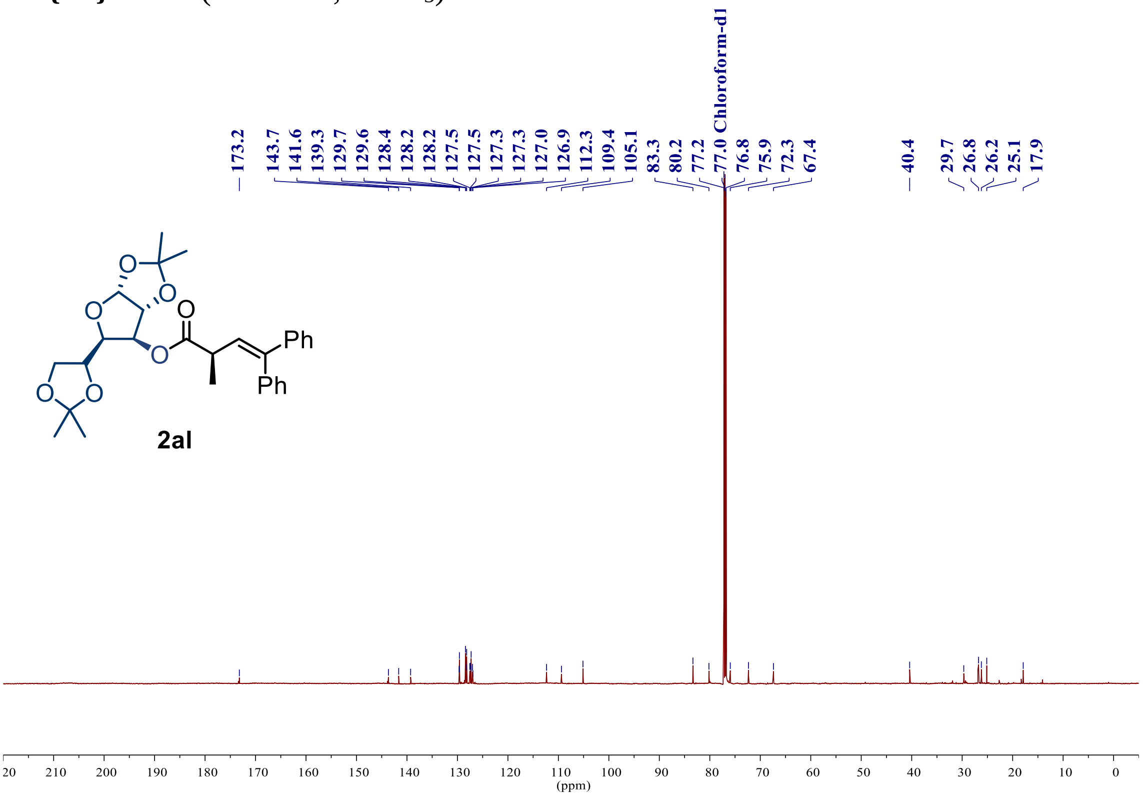

Back to contents

¹H NMR (400 MHz, $CDCl_3$) of (*S*)-**3a**, See procedure

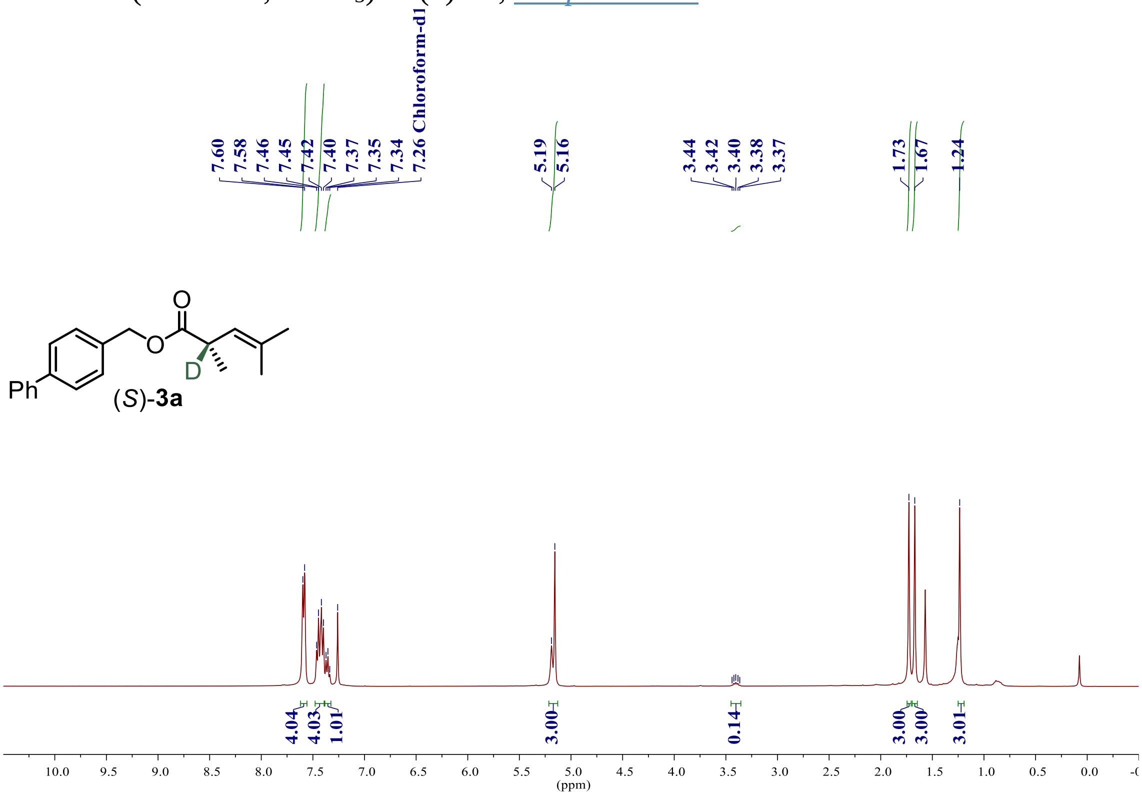

²H NMR (92 MHz, $CDCl_3$) of (*S*)-**3a**

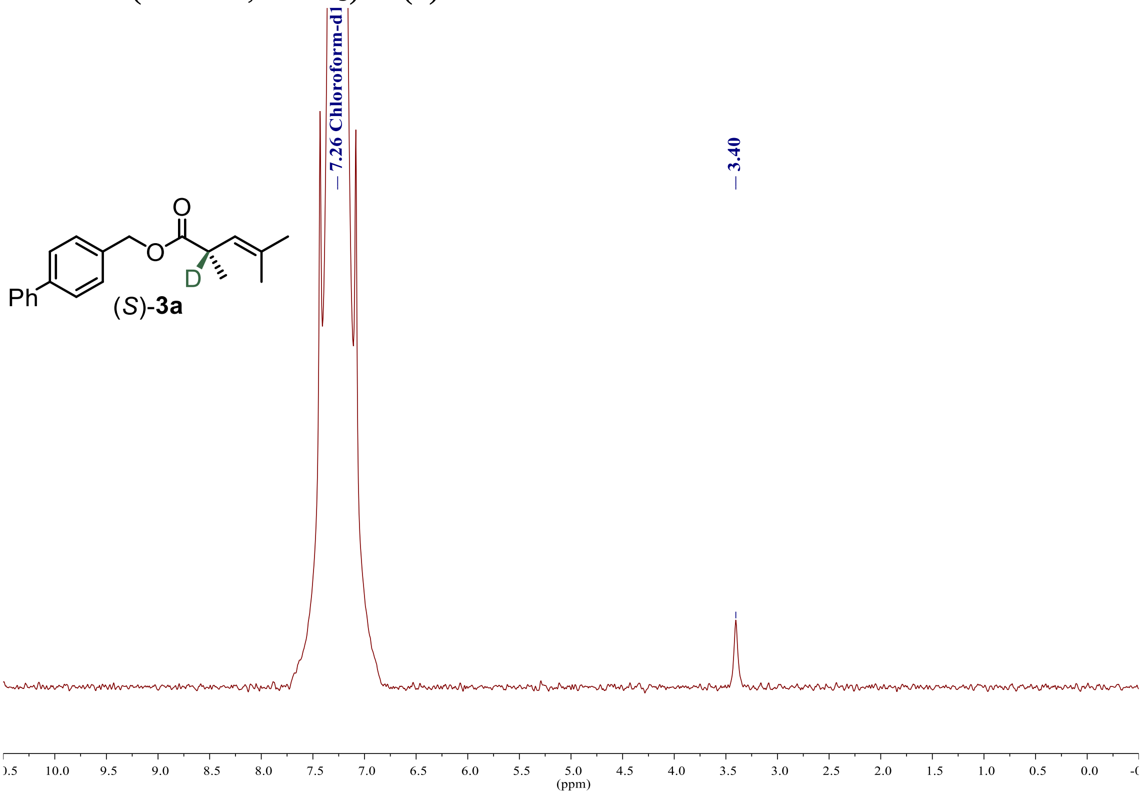

Back to contents

$^{13}C\{^{1}H\}$ NMR (151 MHz, $CDCl_3$) of (*S*)-**3a**

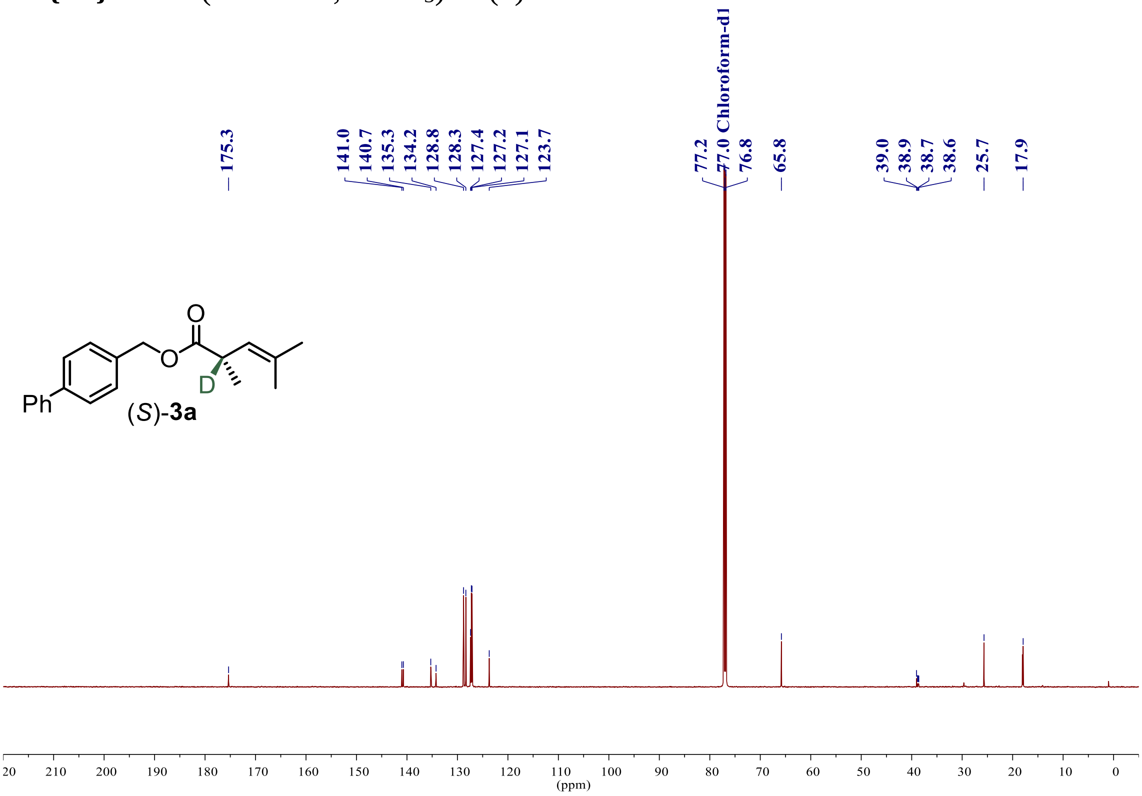

^{1}H NMR (300 MHz, $CDCl_3$) of (*R*)-**3a**, See procedure

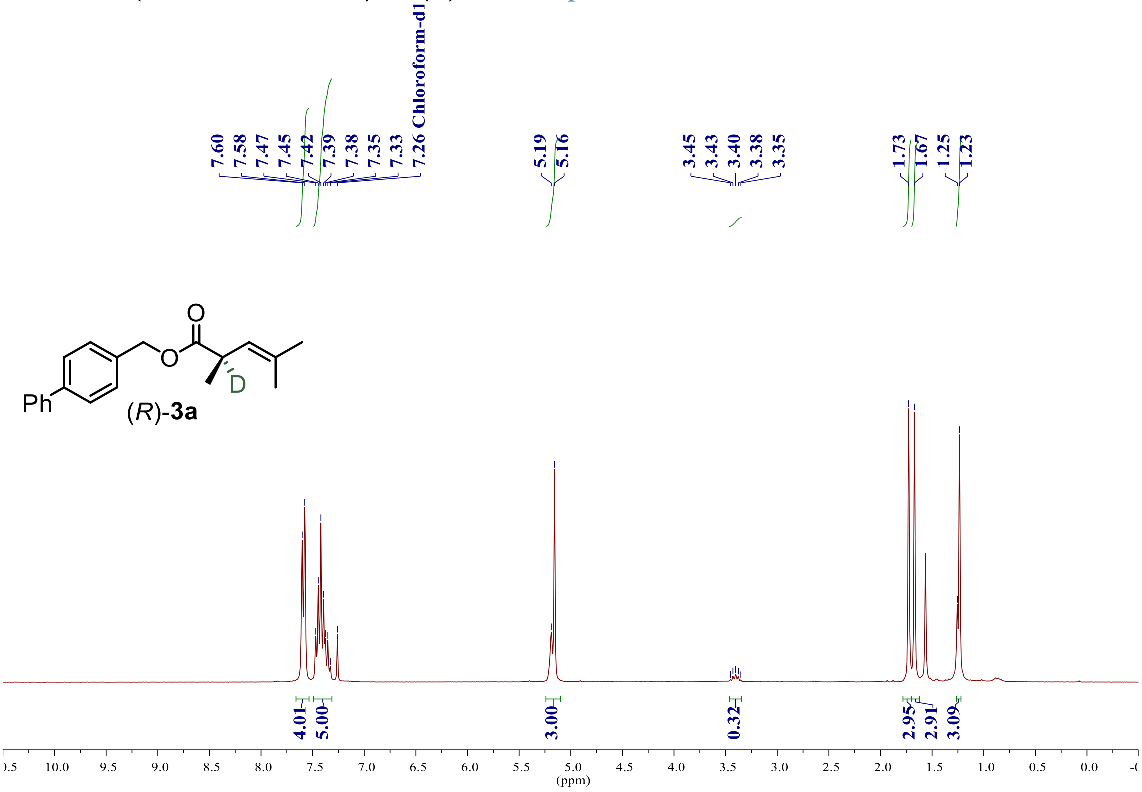

Back to contents

^{2}H NMR (92 MHz, $CDCl_3$) of (*R*)-**3a**

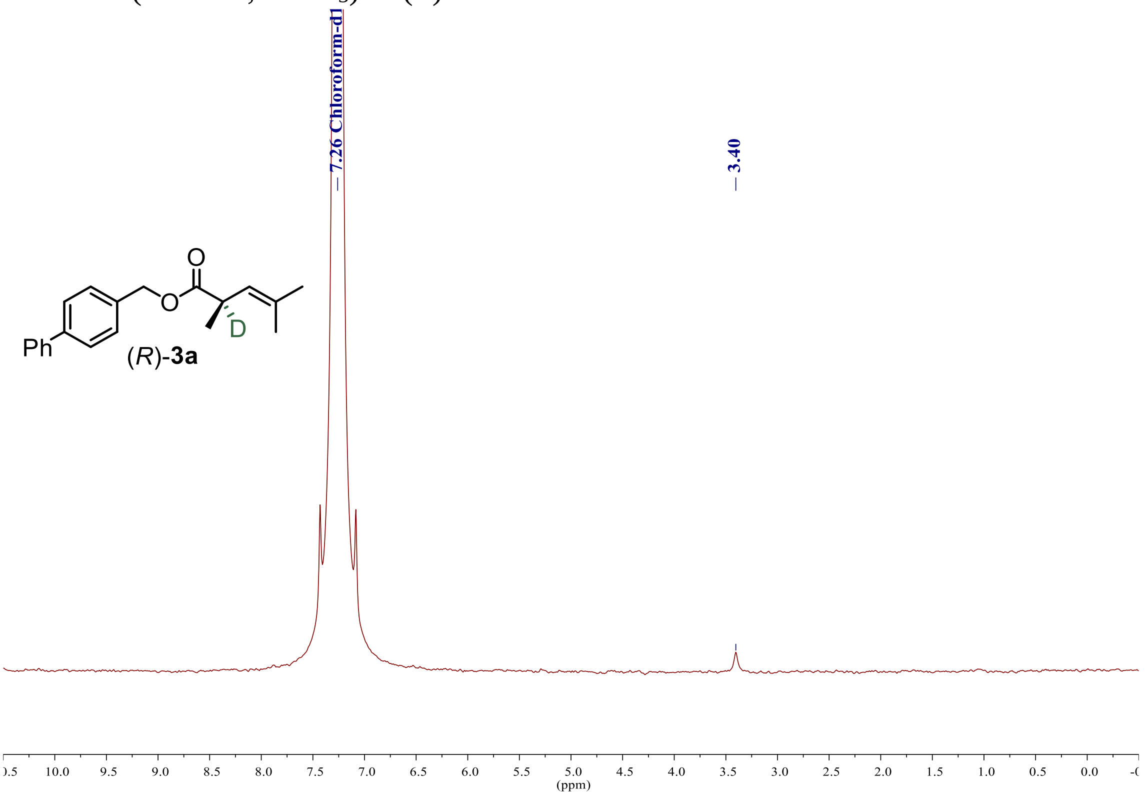

^{13}C{^{1}H} NMR (151 MHz, $CDCl_3$) of (*R*)-**3a**

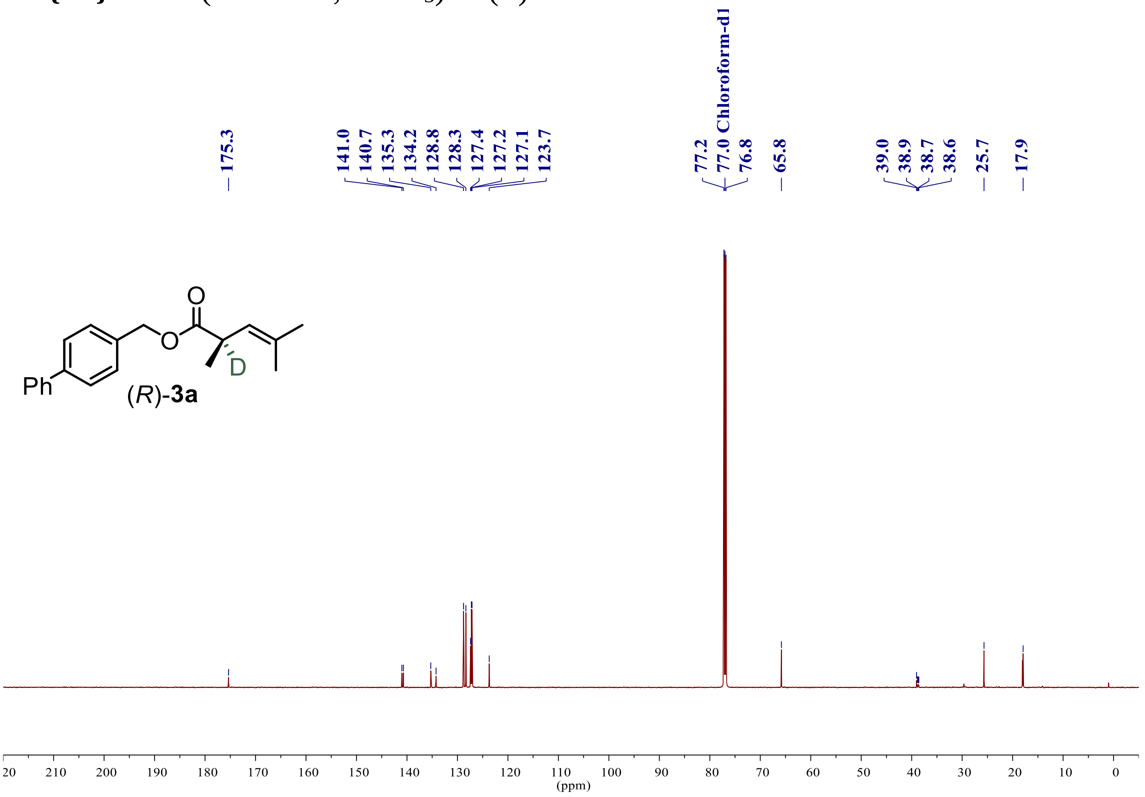

Back to contents

^{1}H NMR (400 MHz, $CDCl_3$) of **3b**, See procedure

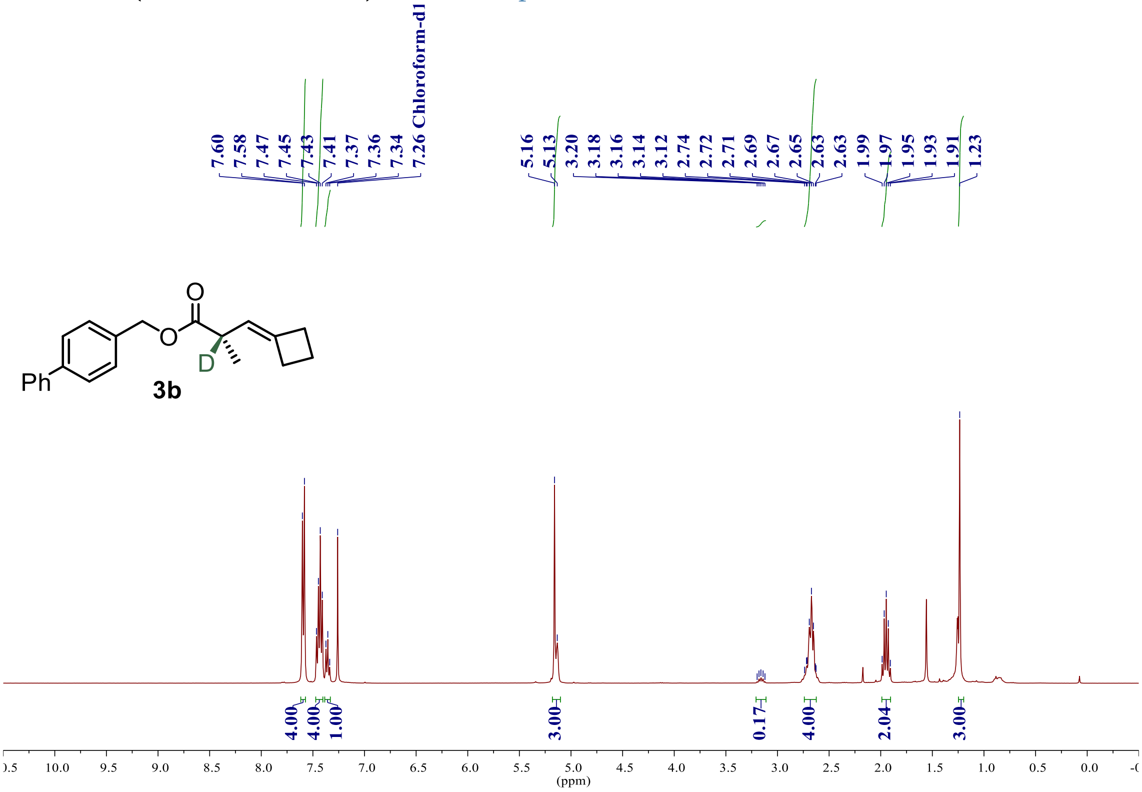

^{2}H NMR (92 MHz, $CDCl_3$) of **3b**

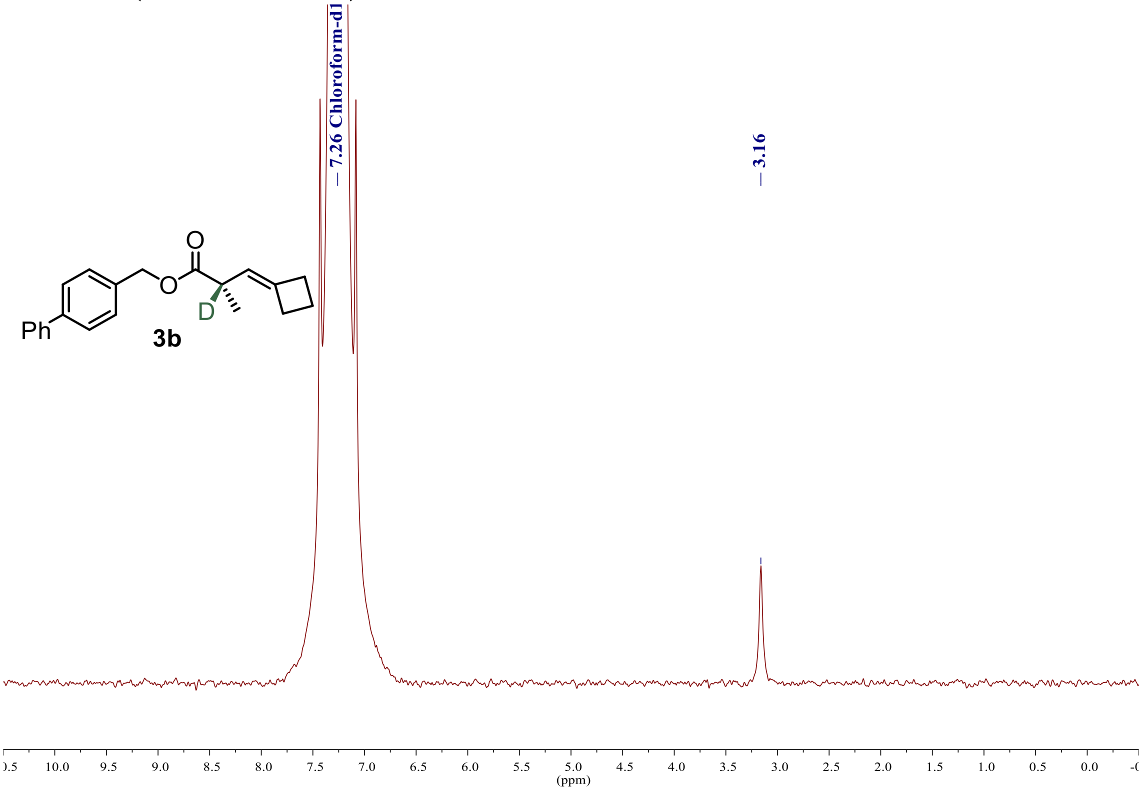

Back to contents

$^{13}C\{^1H\}$ NMR (151 MHz, $CDCl_3$) of **3b**

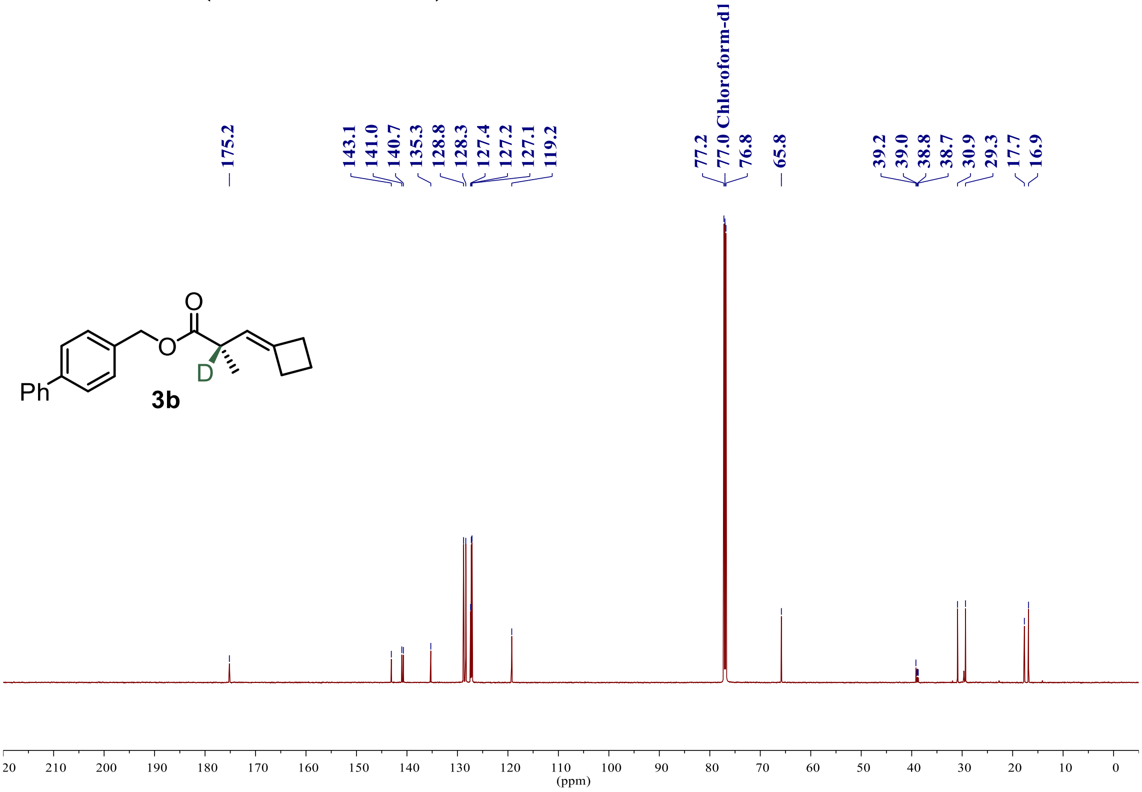

^1H NMR (400 MHz, $CDCl_3$) of **3c**, See procedure

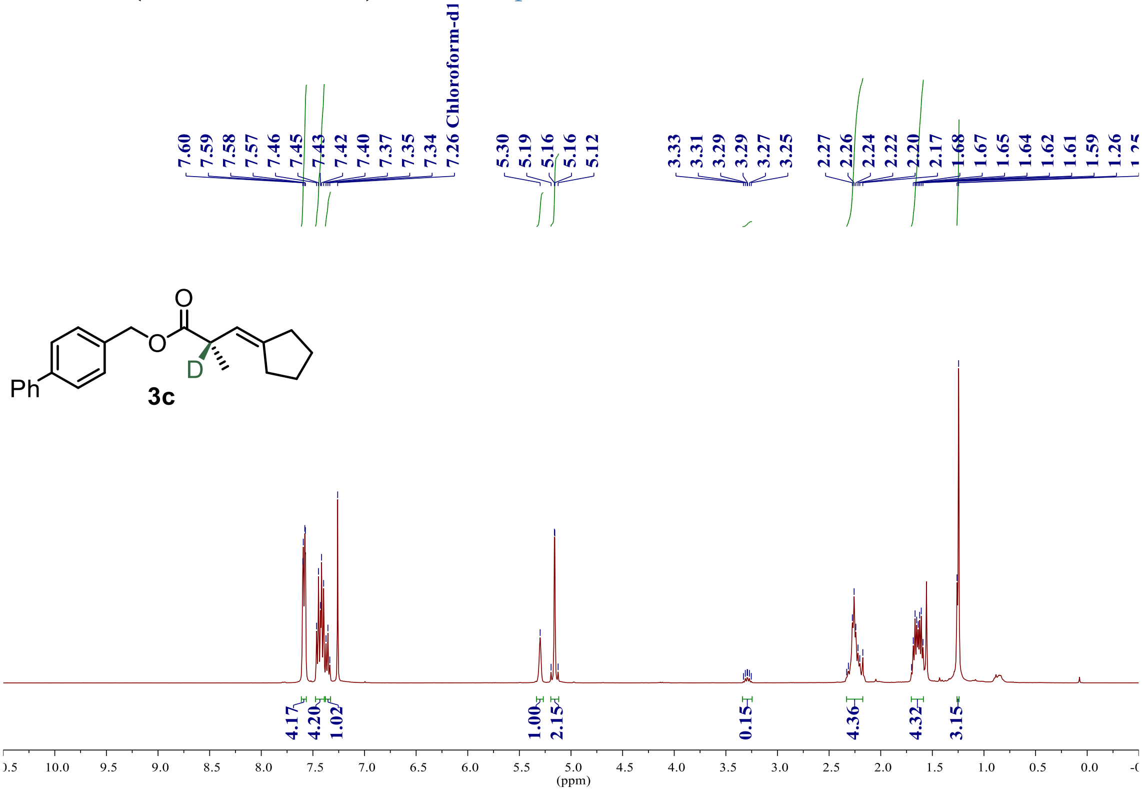

^{2}H NMR (92 MHz, $CDCl_3$) of **3c**

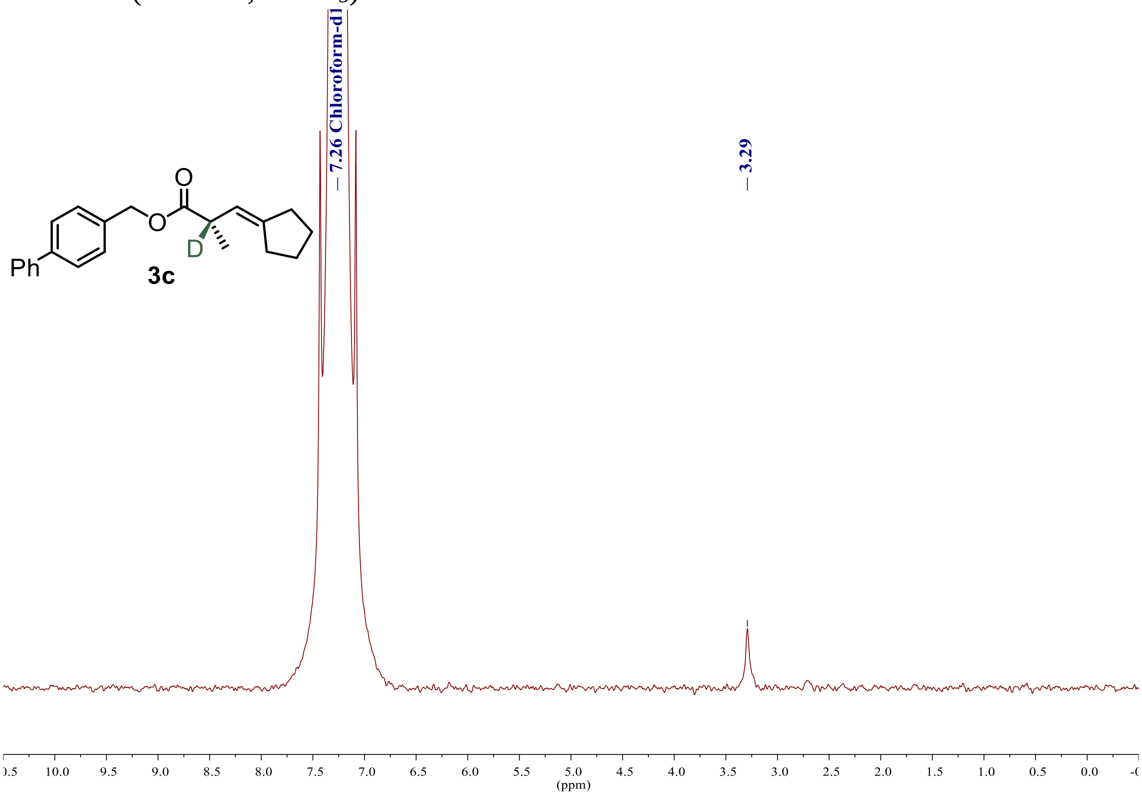

^{13}C{^{1}H} NMR (151 MHz, $CDCl_3$) of **3c**

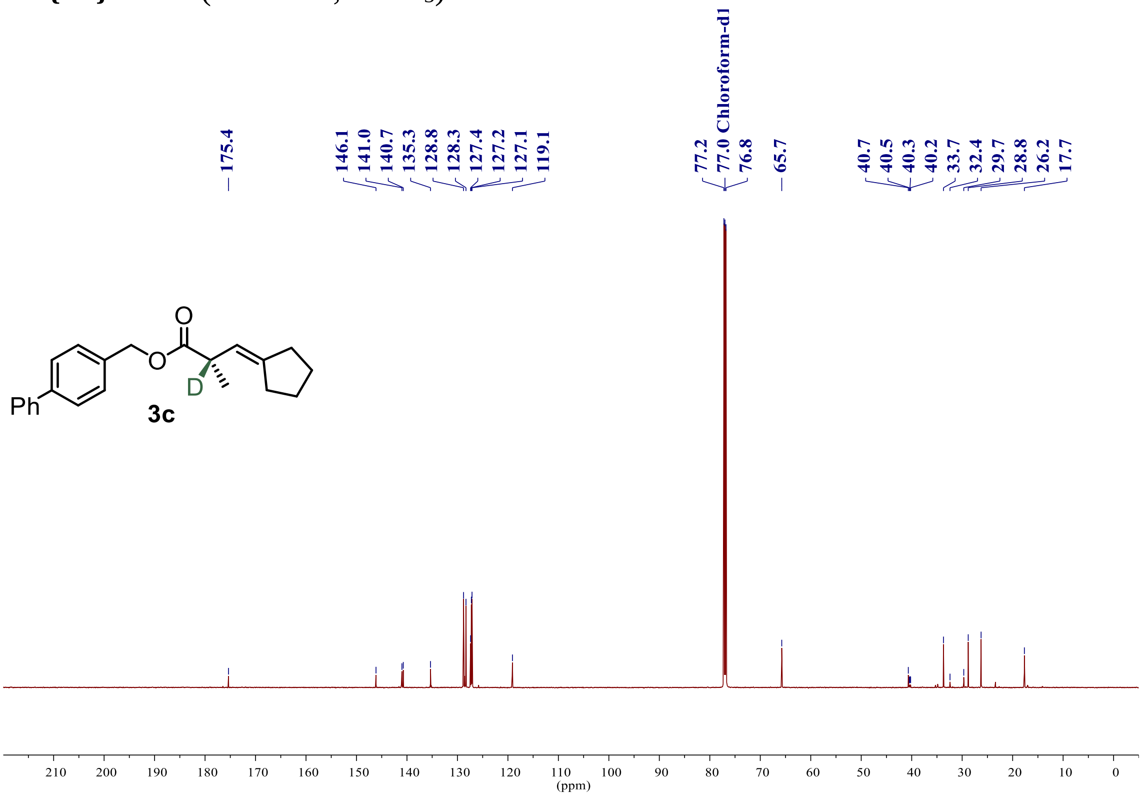

[Back to contents](#)

^{1}H NMR (300 MHz, $CDCl_3$) of **3d**, *See procedure*

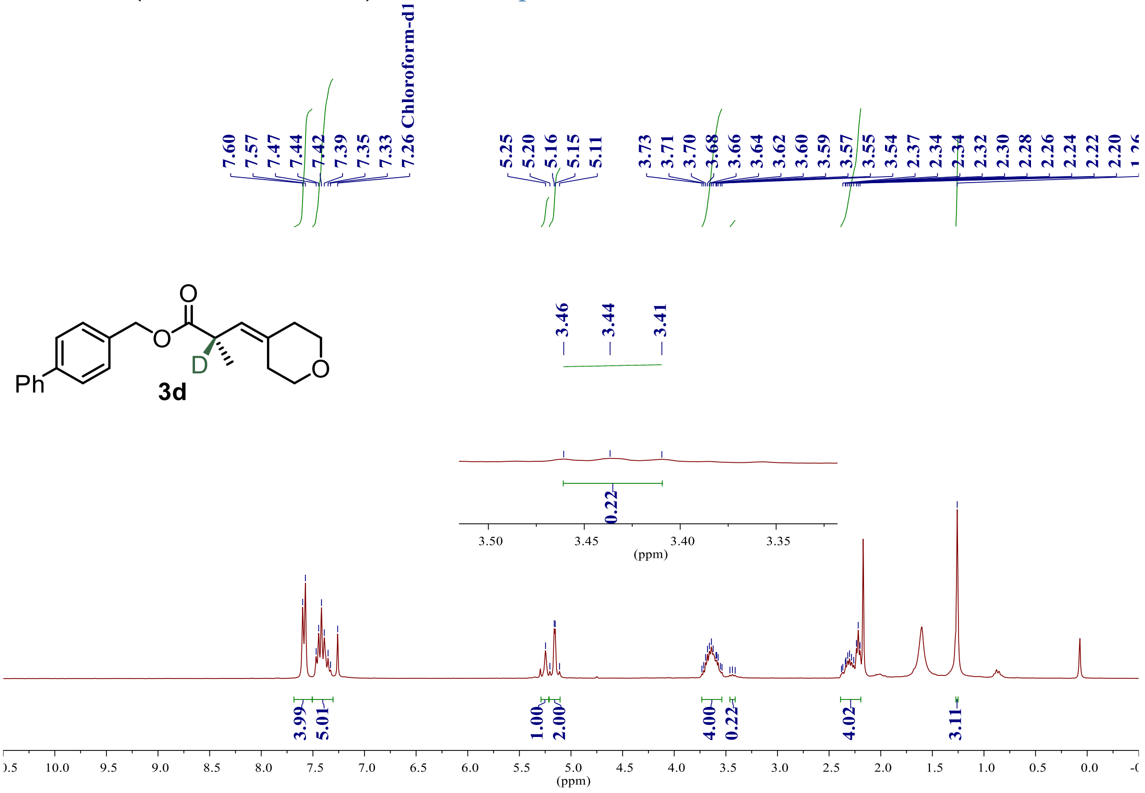

^{2}H NMR (92 MHz, $CDCl_3$) of **3d**

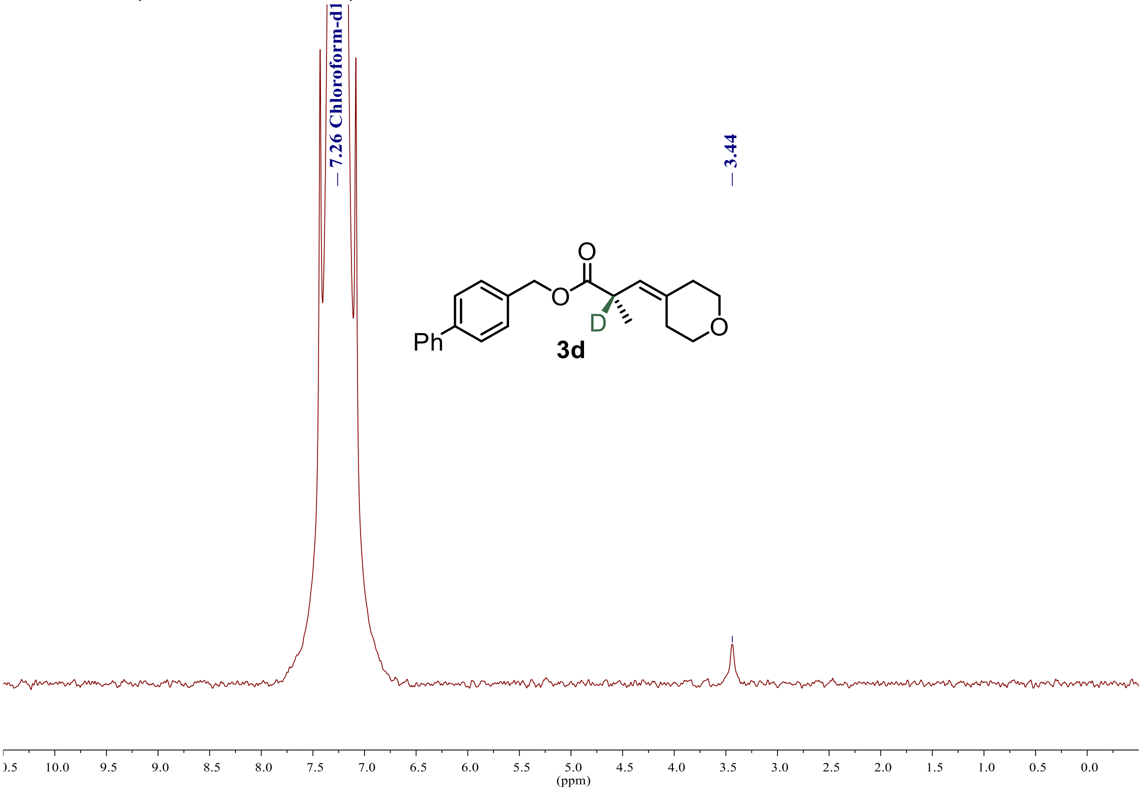

Back to contents

$^{13}C\{^{1}H\}$ NMR (151 MHz, $CDCl_3$) of **3d**

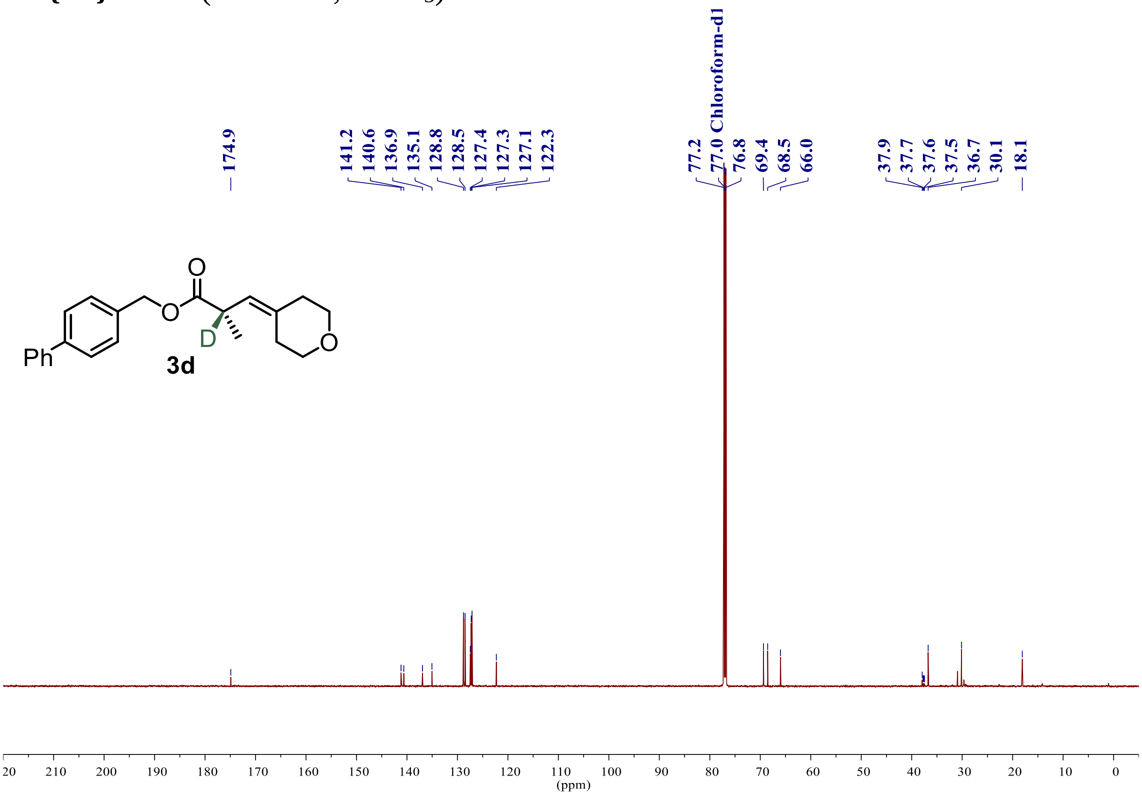

^{1}H NMR (400 MHz, $CDCl_3$) of **3e**, See procedure

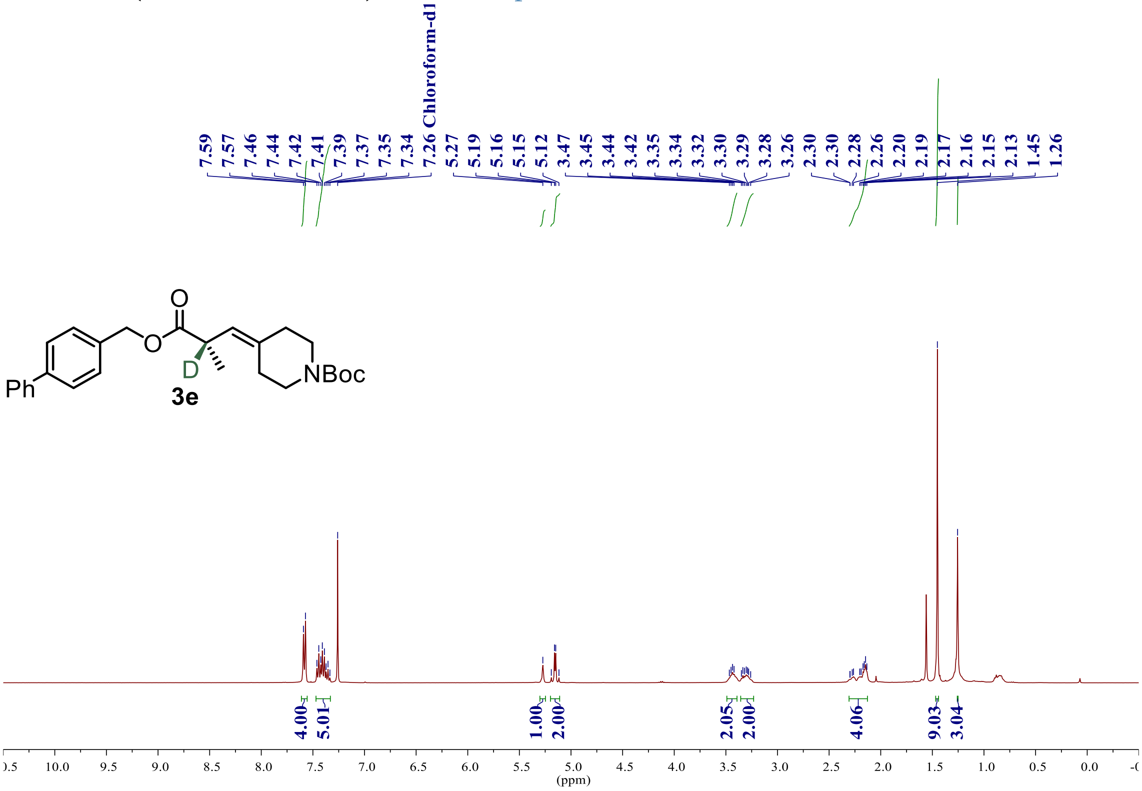

Back to contents

^{2}H NMR (92 MHz, $CDCl_3$) of **3e**

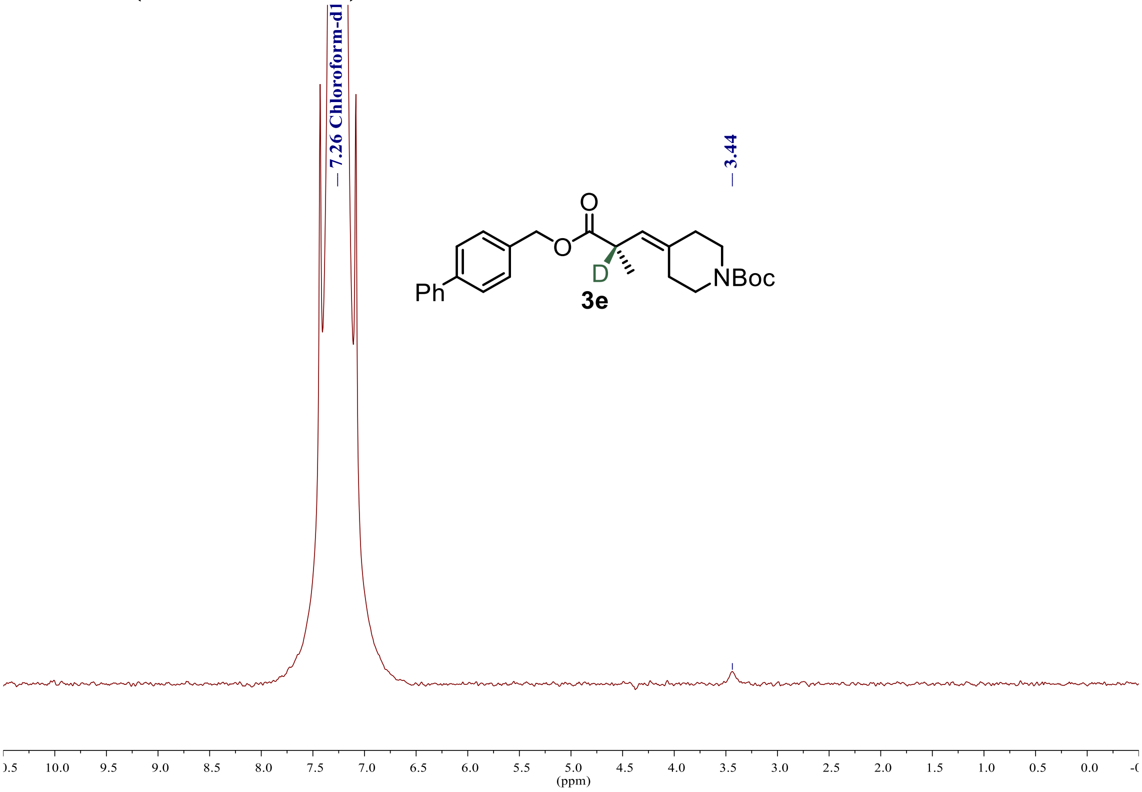

^{13}C{^{1}H} NMR (151 MHz, $CDCl_3$) of **3e**

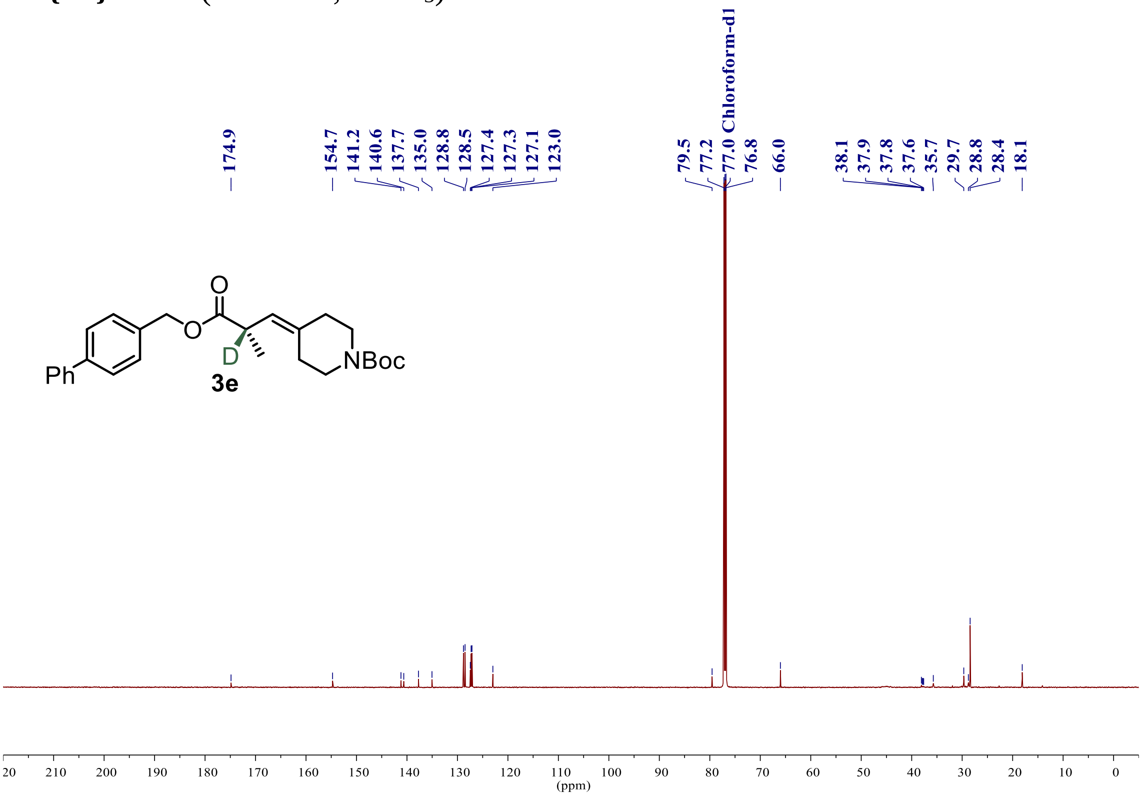

Back to contents

^{1}H NMR (400 MHz, $CDCl_3$) of **3f**, See procedure

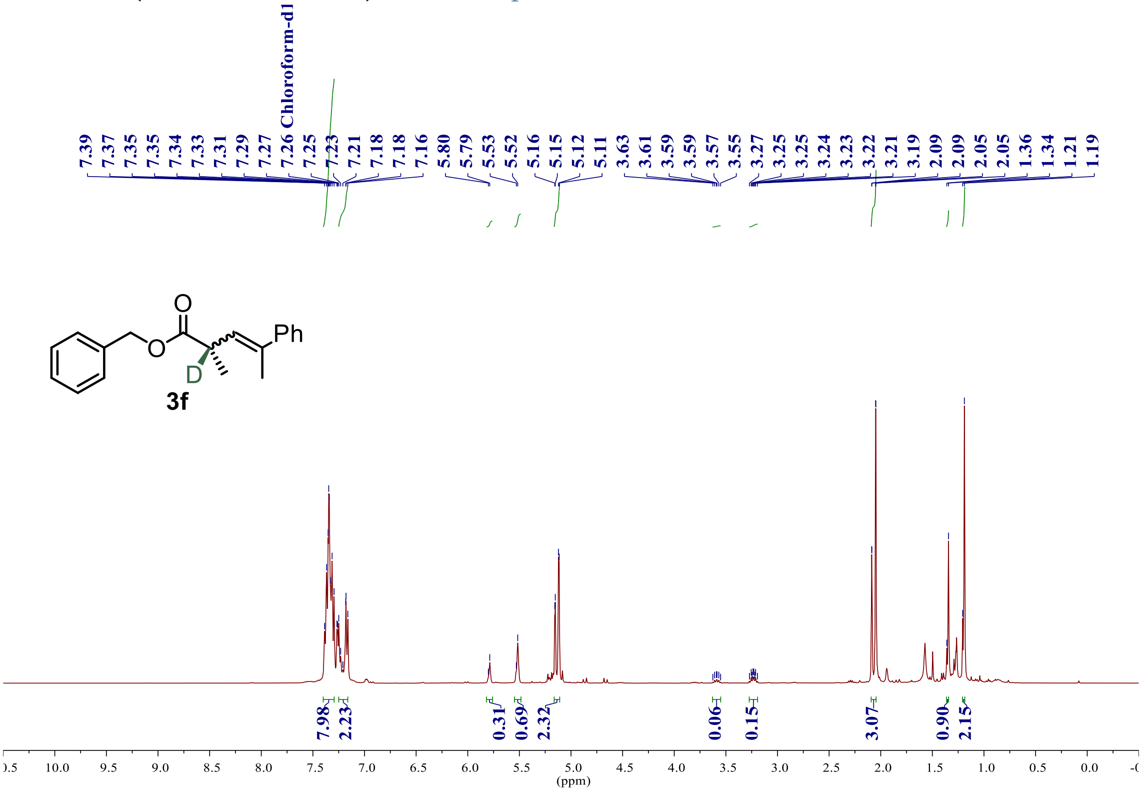

^{2}H NMR (92 MHz, $CDCl_3$) of **3f**

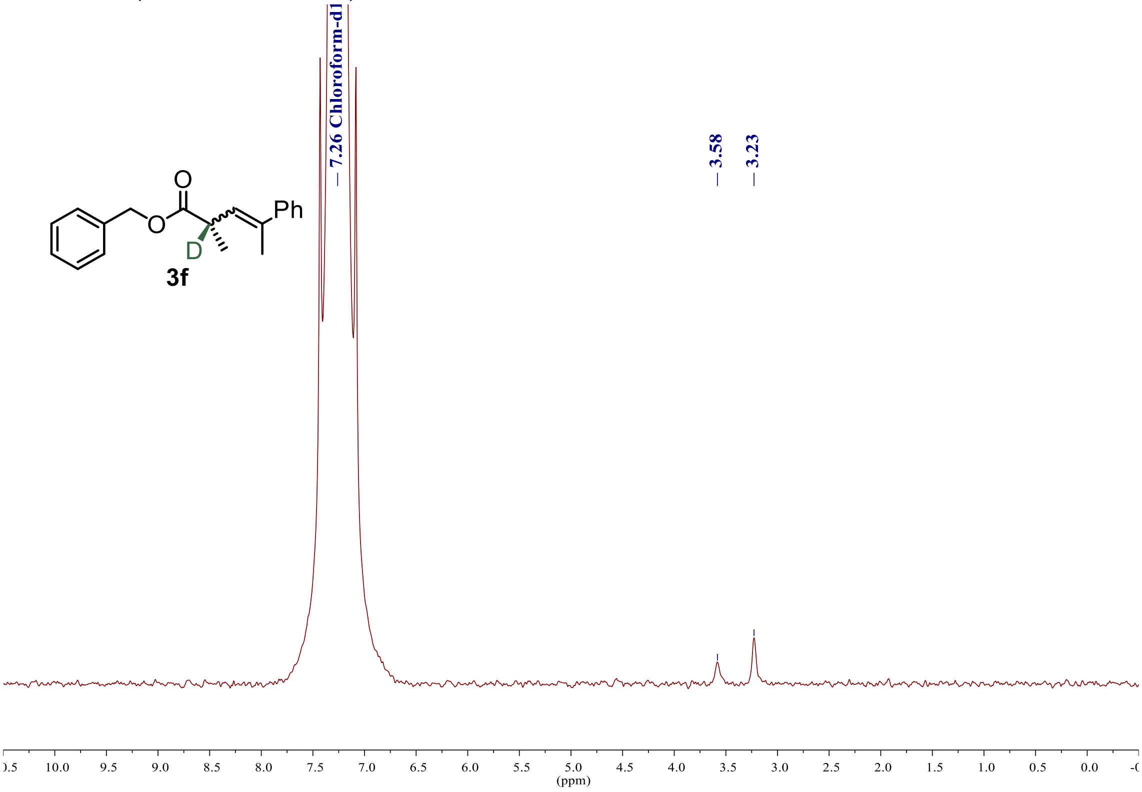

Back to contents

^{13}C{^{1}H} NMR (151 MHz, $CDCl_3$) of **3f**

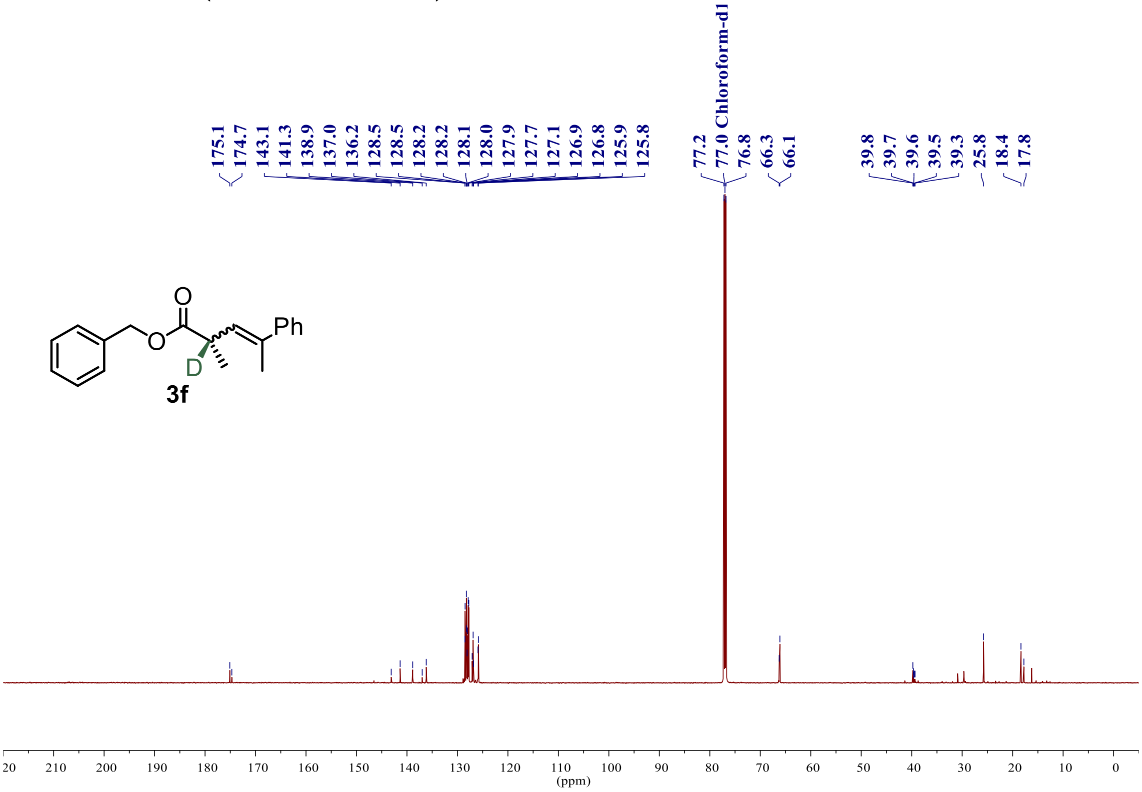

^{1}H NMR (400 MHz, $CDCl_3$) of **3g**, See procedure

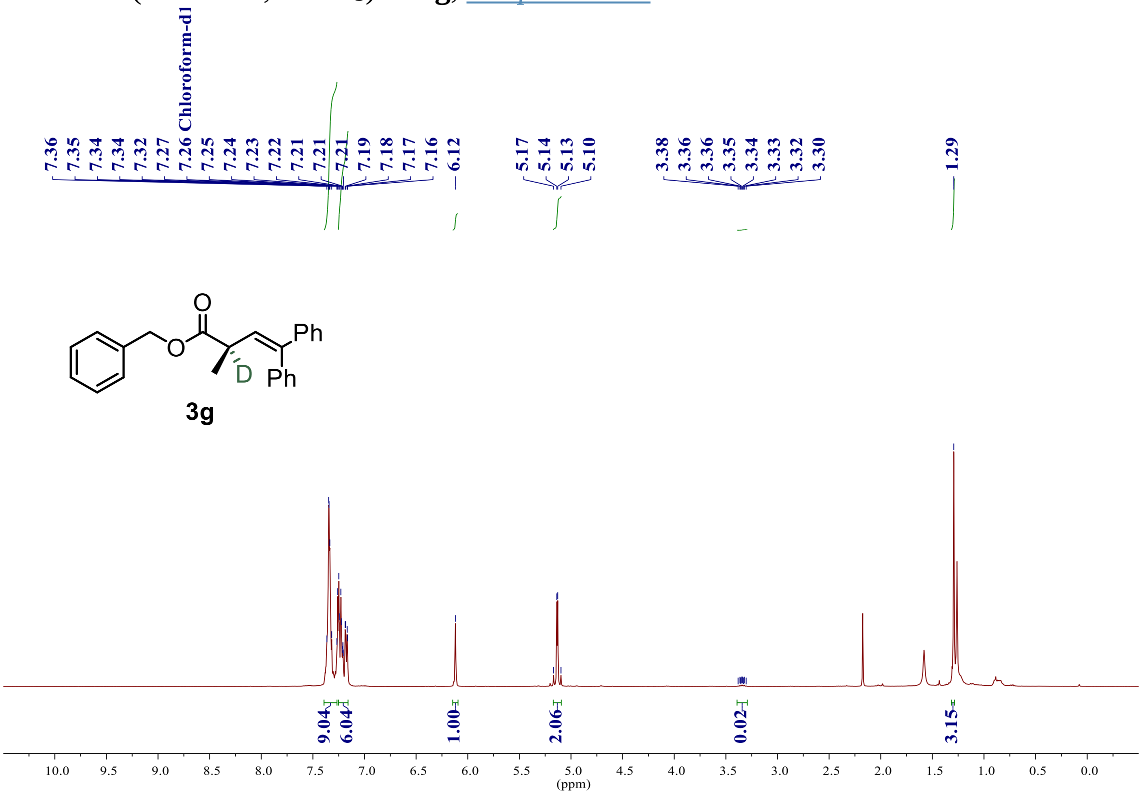

^{2}H NMR (92 MHz, $CDCl_3$) of **3g**

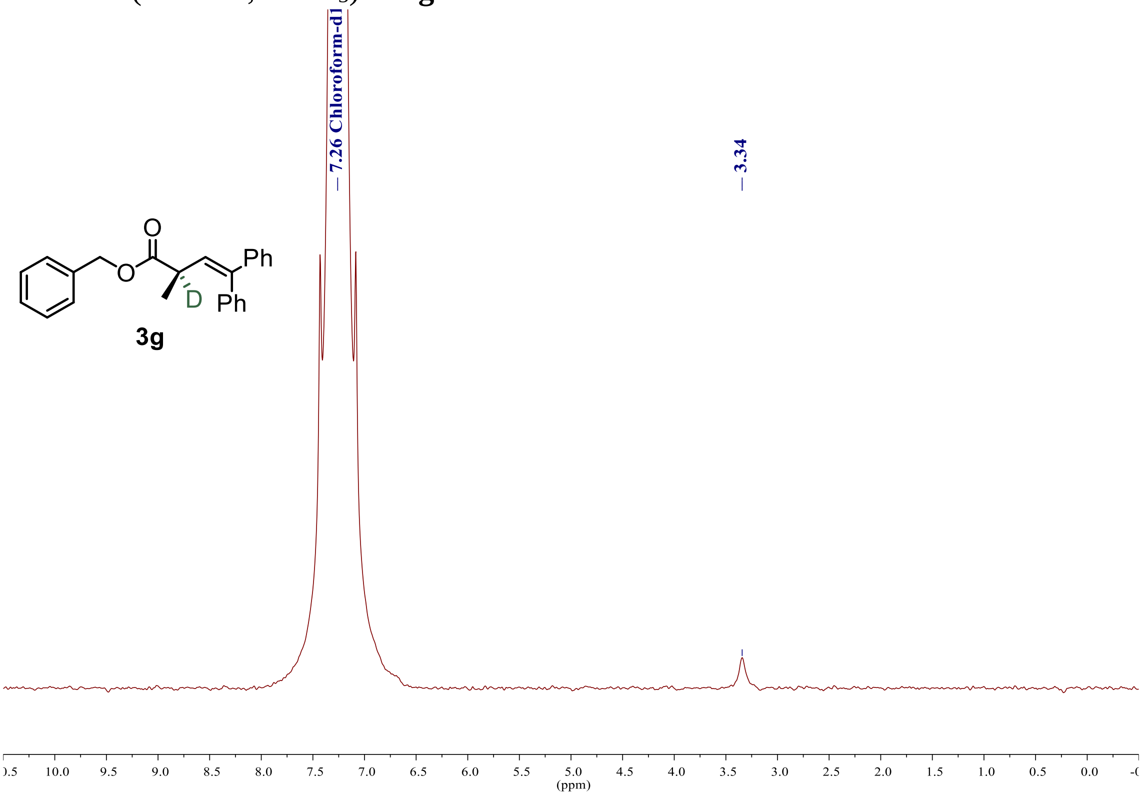

^{13}C{^{1}H} NMR (151 MHz, $CDCl_3$) of **3g**

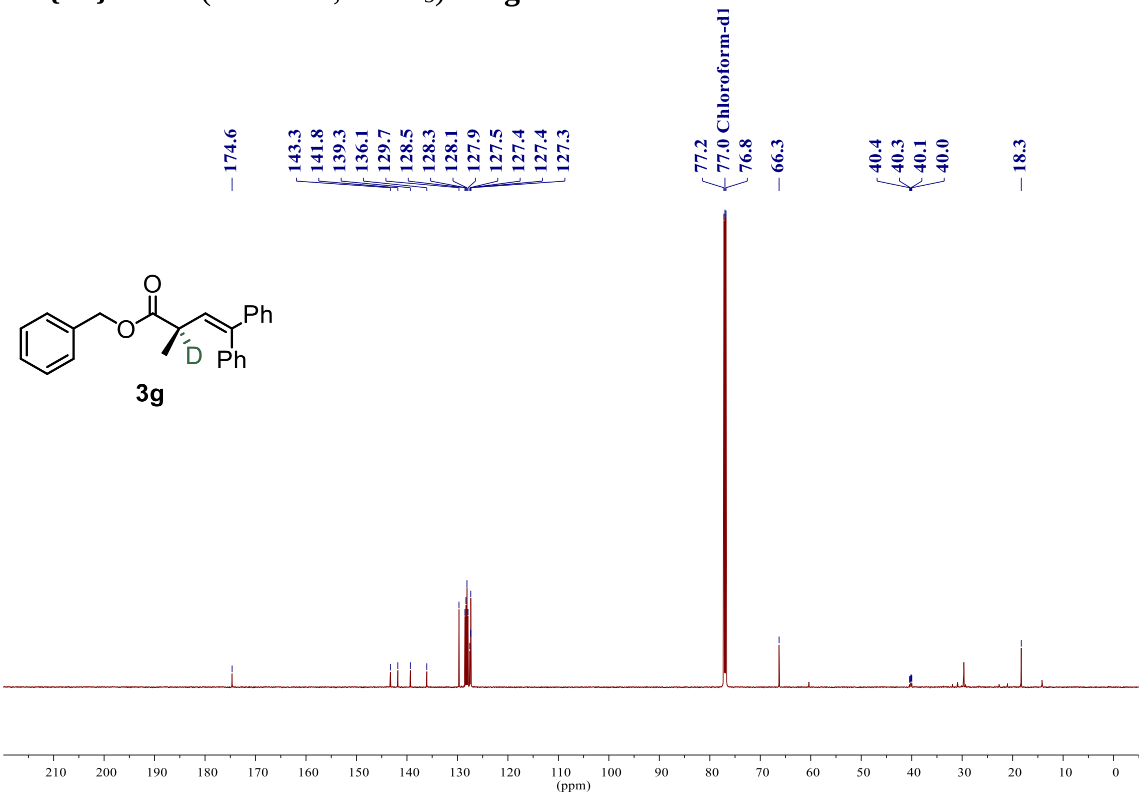

^{1}**H NMR** (400 MHz, $CDCl_3$) of **3h**, See procedure

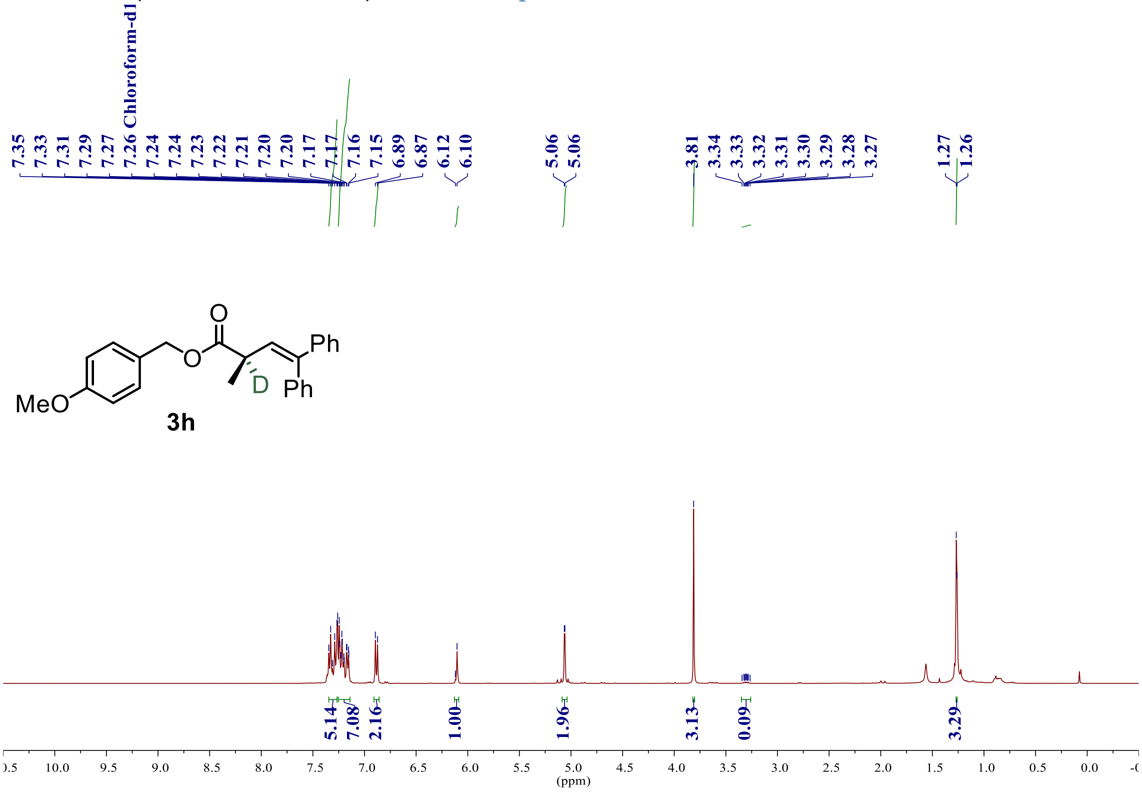

^{2}**H NMR** (92 MHz, $CDCl_3$) of **3h**

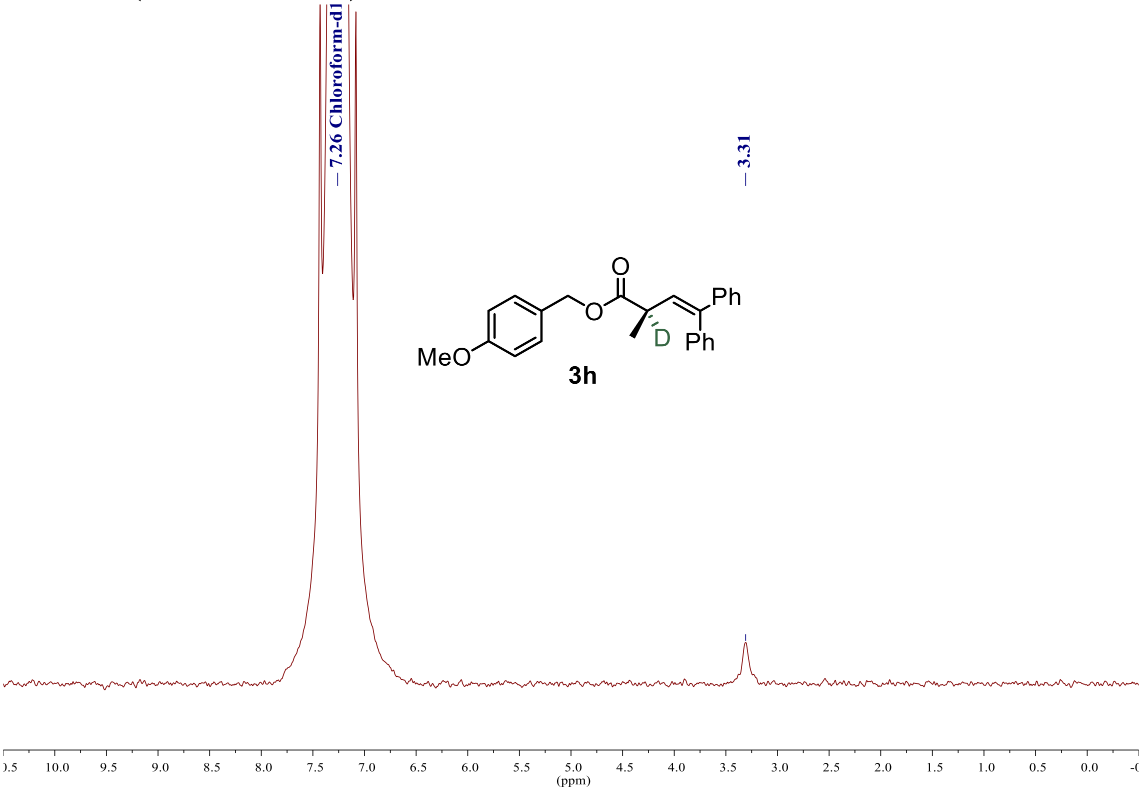

Back to contents

$^{13}C\{^{1}H\}$ NMR (151 MHz, $CDCl_3$) of **3h**

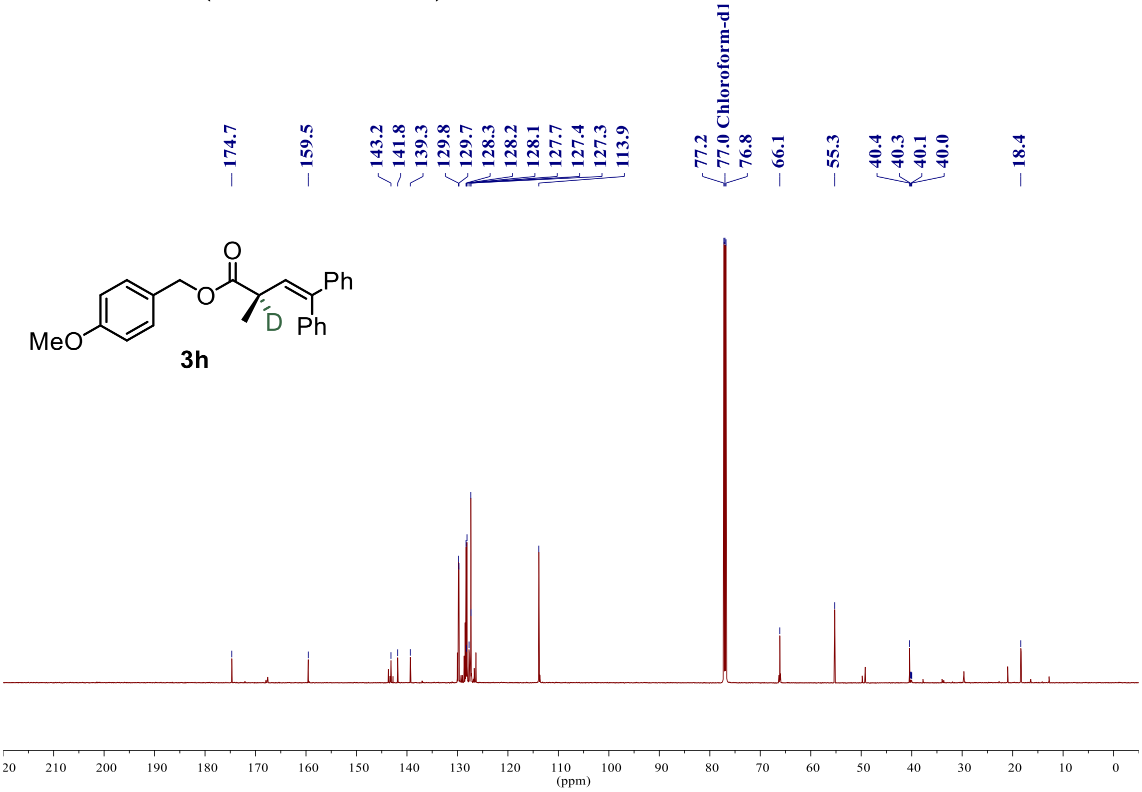

^{1}H NMR (400 MHz, $CDCl_3$) of **3i**, See procedure

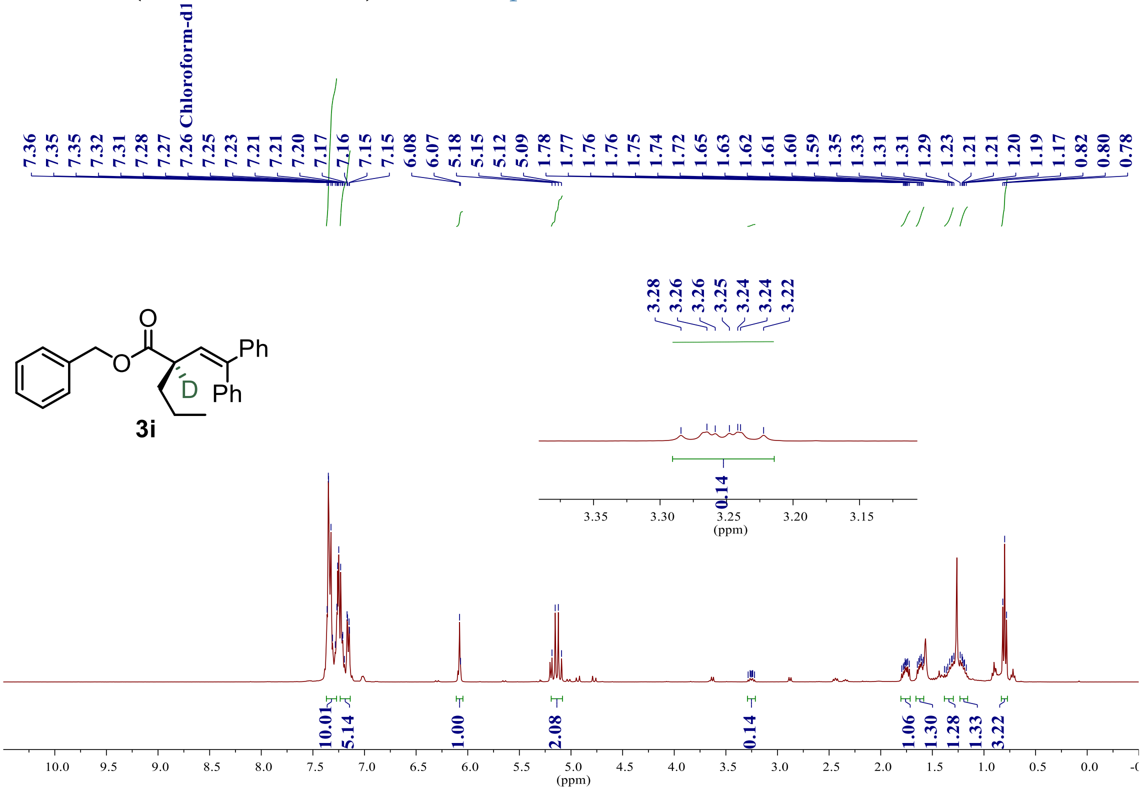

[Back to contents](#)

^{2}**H NMR** (92 MHz, $CDCl_3$) of **3i**

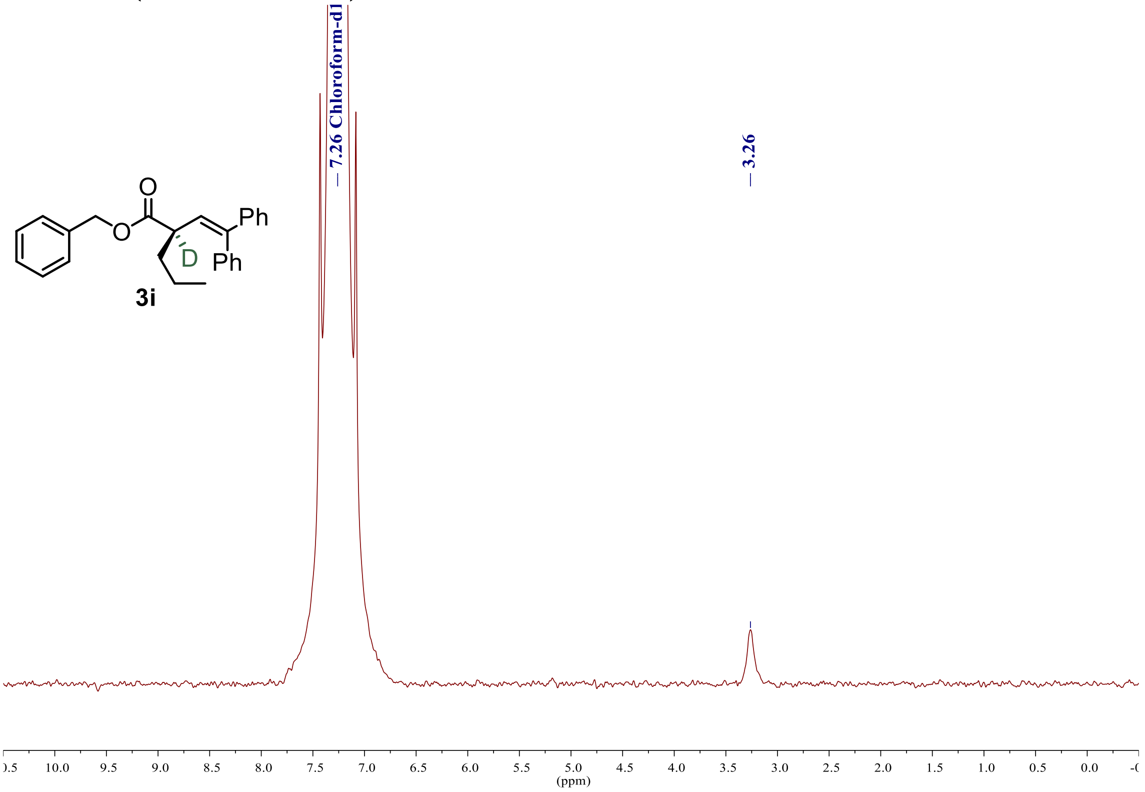

^{13}**C{^{1}H} NMR** (151 MHz, $CDCl_3$) of **3i**

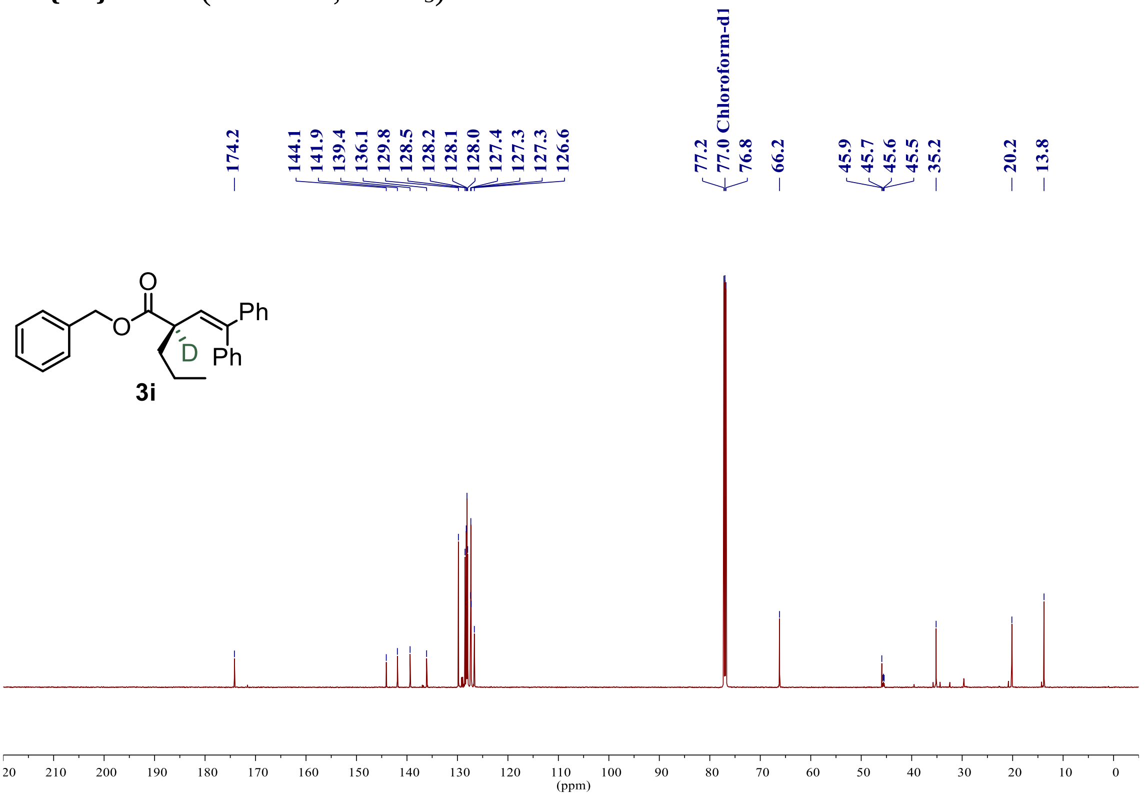

Back to contents

^{1}H NMR (600 MHz, $CDCl_3$) of **S5a**, See procedure

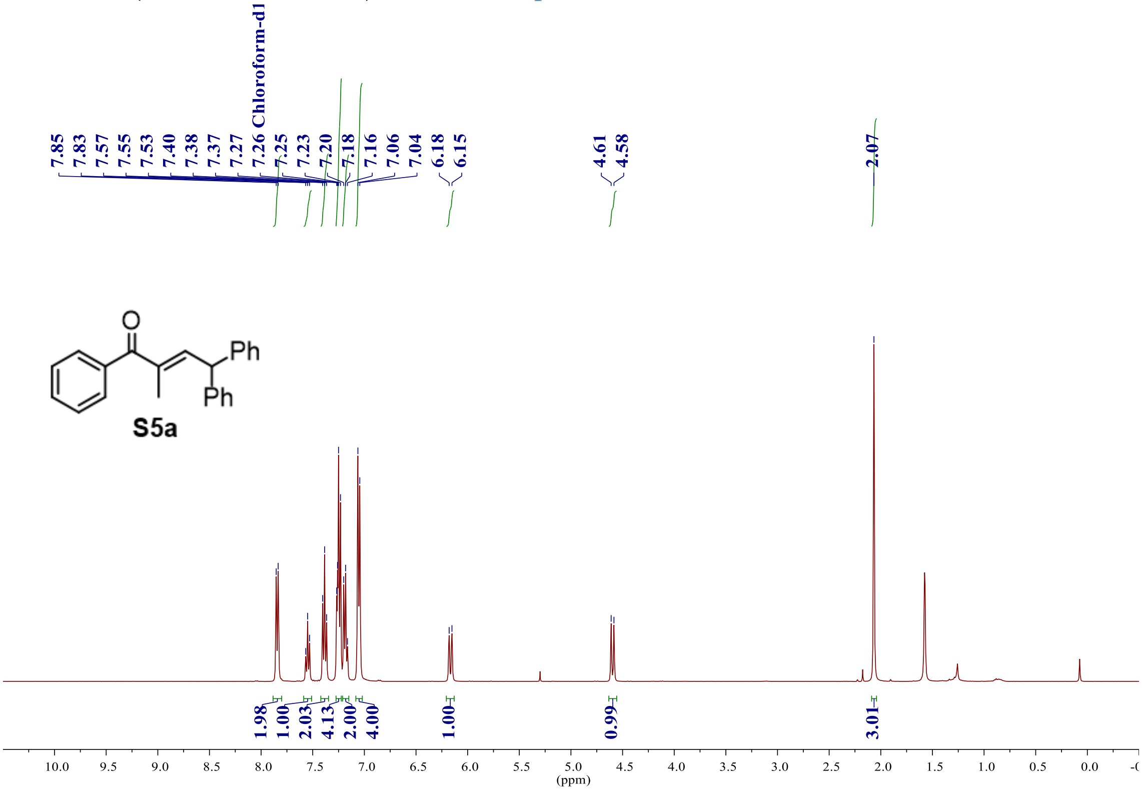

^{13}C{^{1}H} NMR (151 MHz, $CDCl_3$) of **S5a**

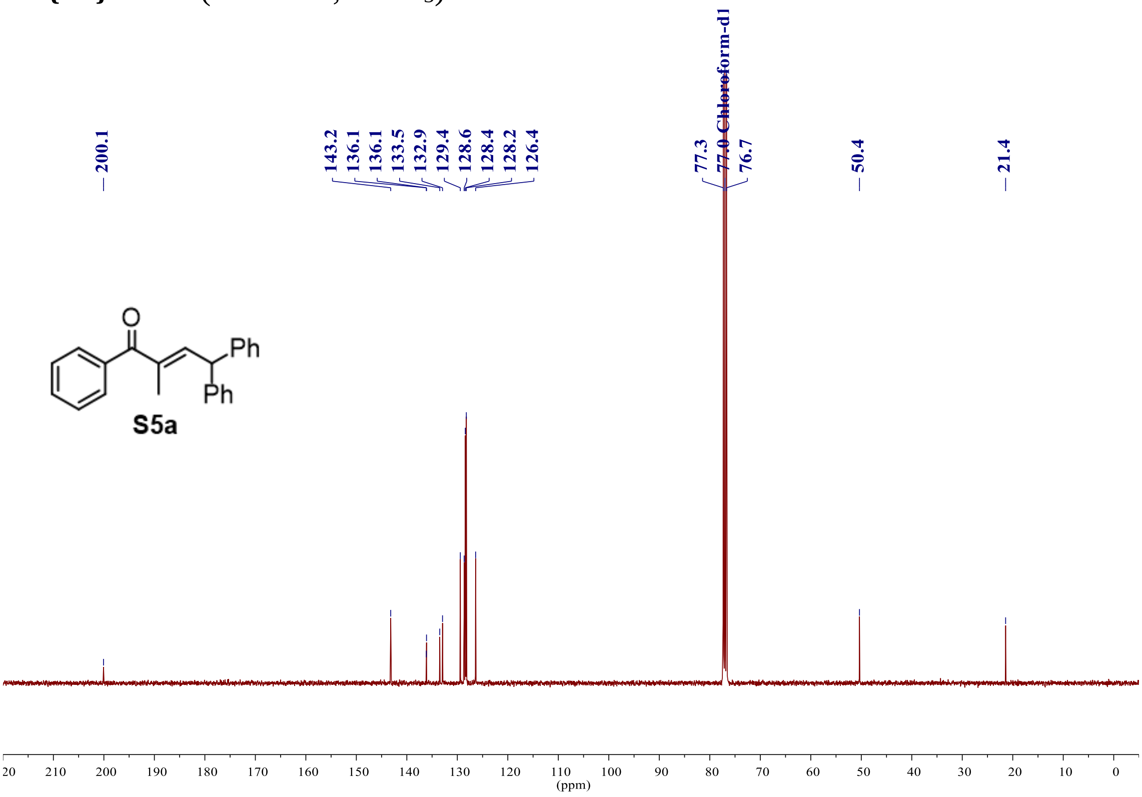

Back to contents

^{1}H NMR (600 MHz, $CDCl_3$) of **S5b**, See procedure

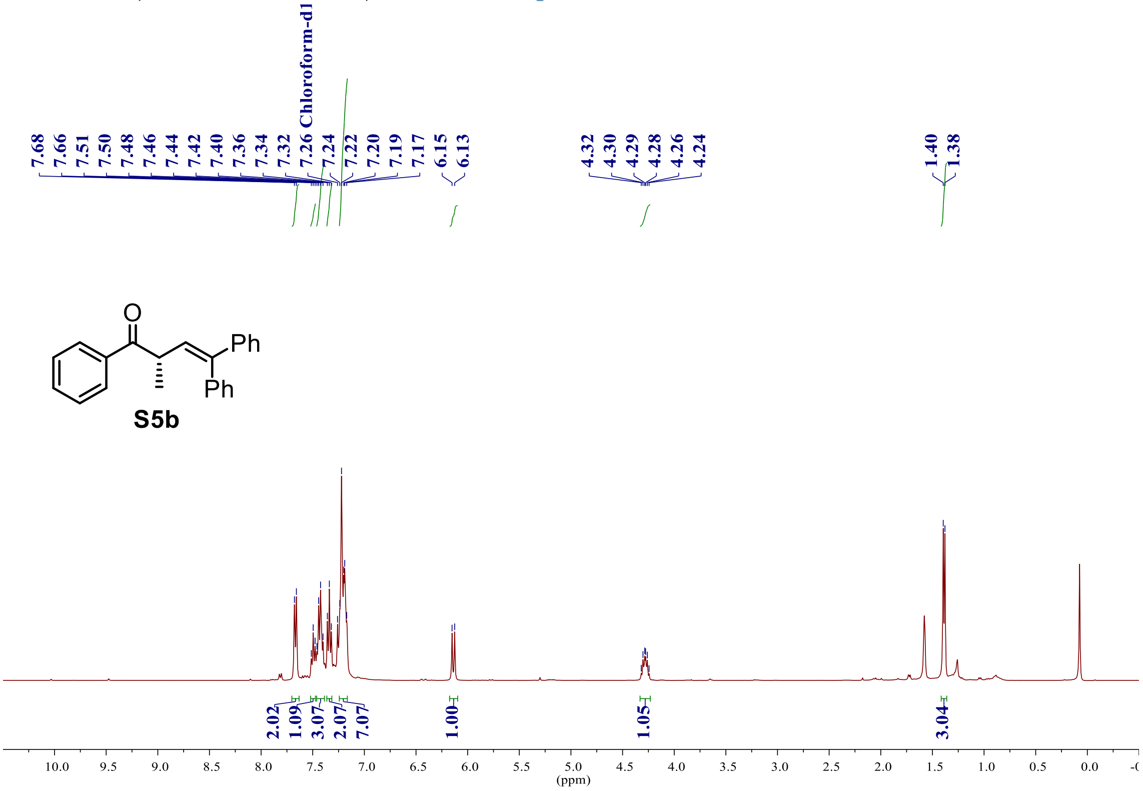

^{13}C{^{1}H} NMR (151 MHz, $CDCl_3$) of **S5b**

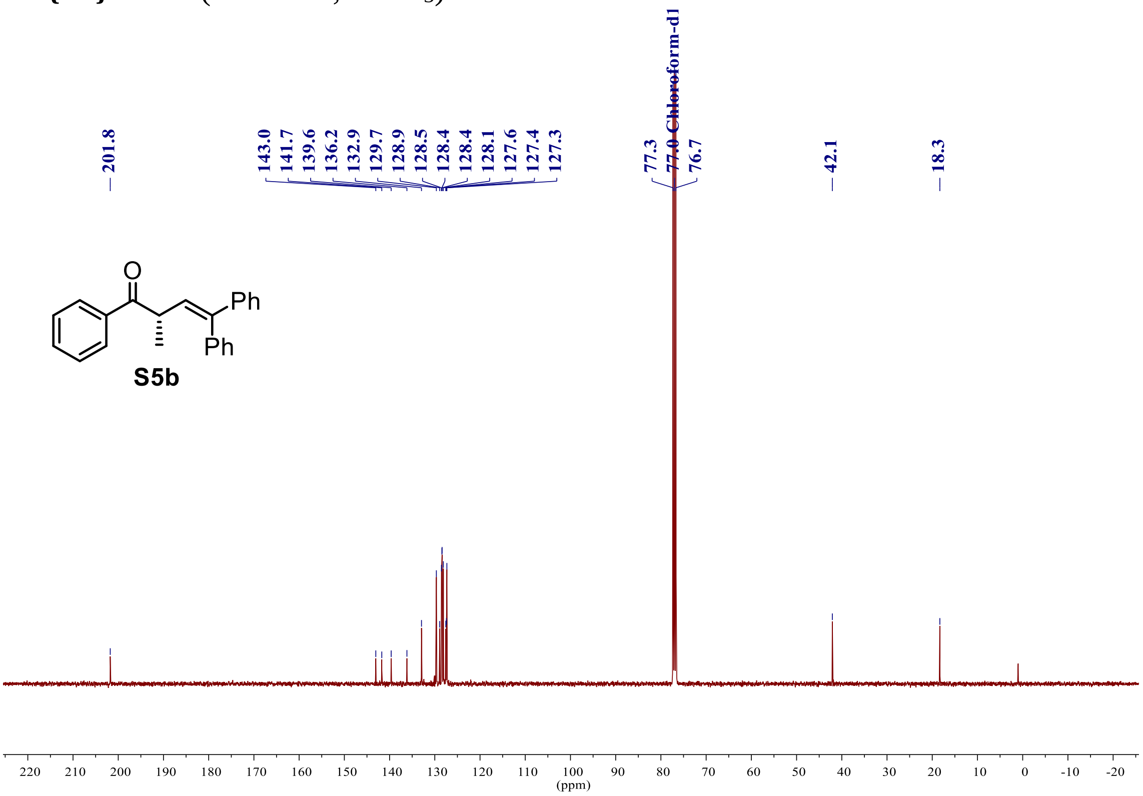

[Back to contents](#)

^{1}H NMR (300 MHz, $CDCl_3$) of **4**, See procedure

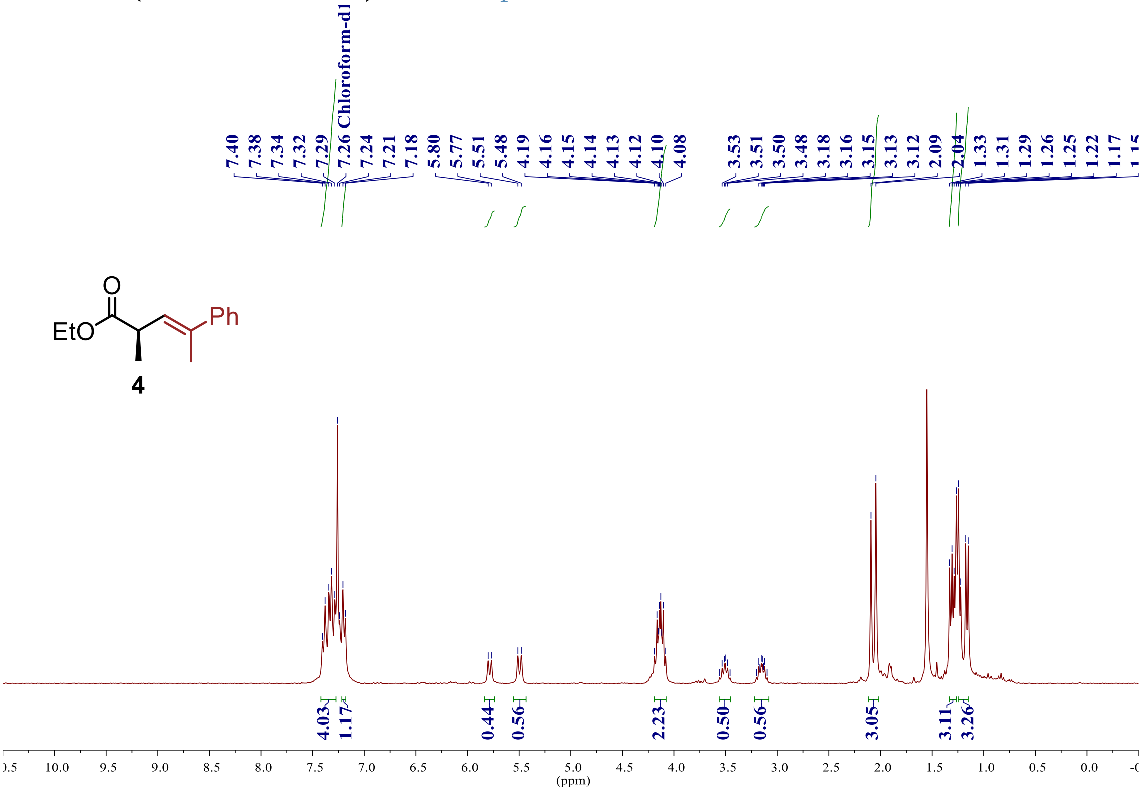

^{13}C{^{1}H} NMR (101 MHz, $CDCl_3$) of **4**

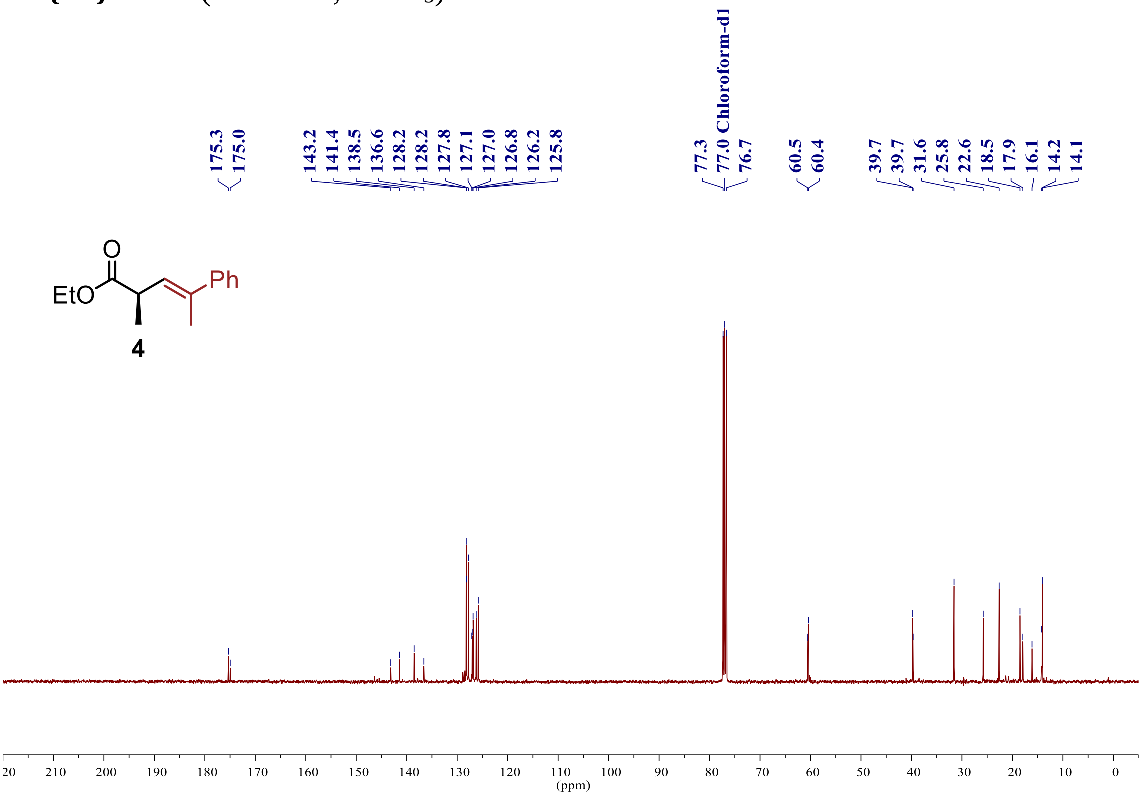

Back to contents

^{1}H NMR (400 MHz, $CDCl_3$) of **7**, See procedure

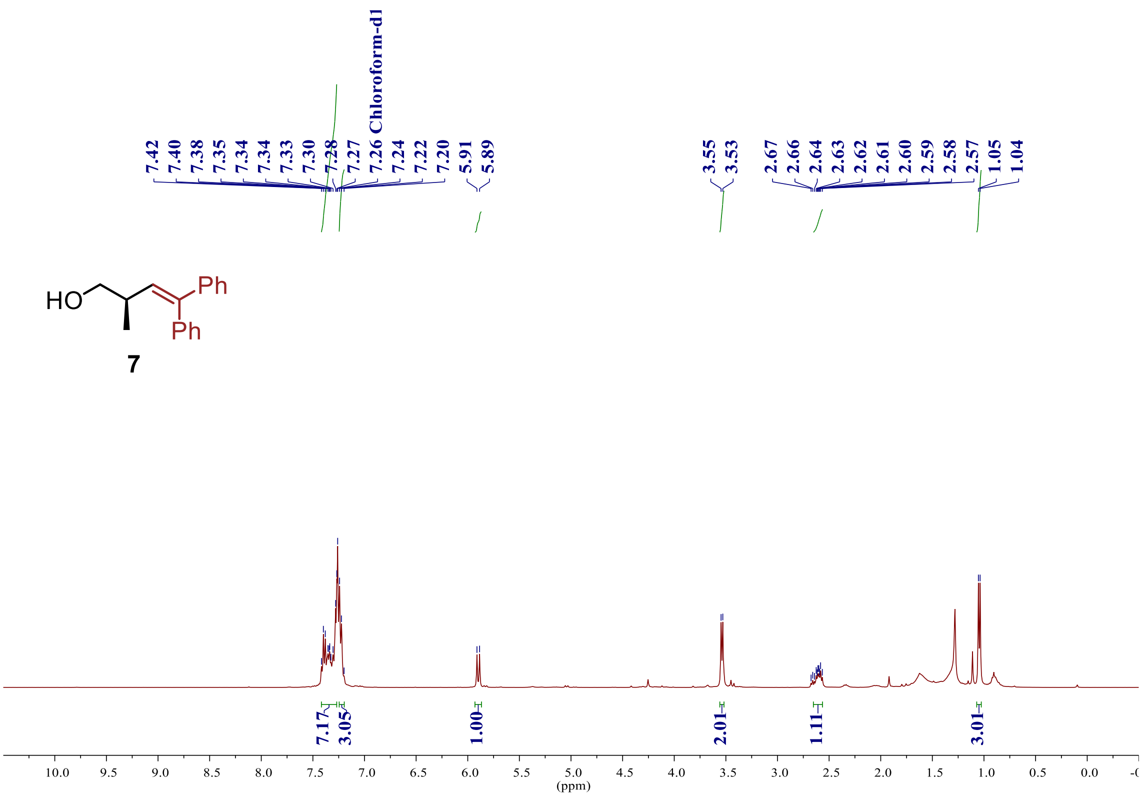

^{13}C{^{1}H} NMR (101 MHz, $CDCl_3$) of **7**

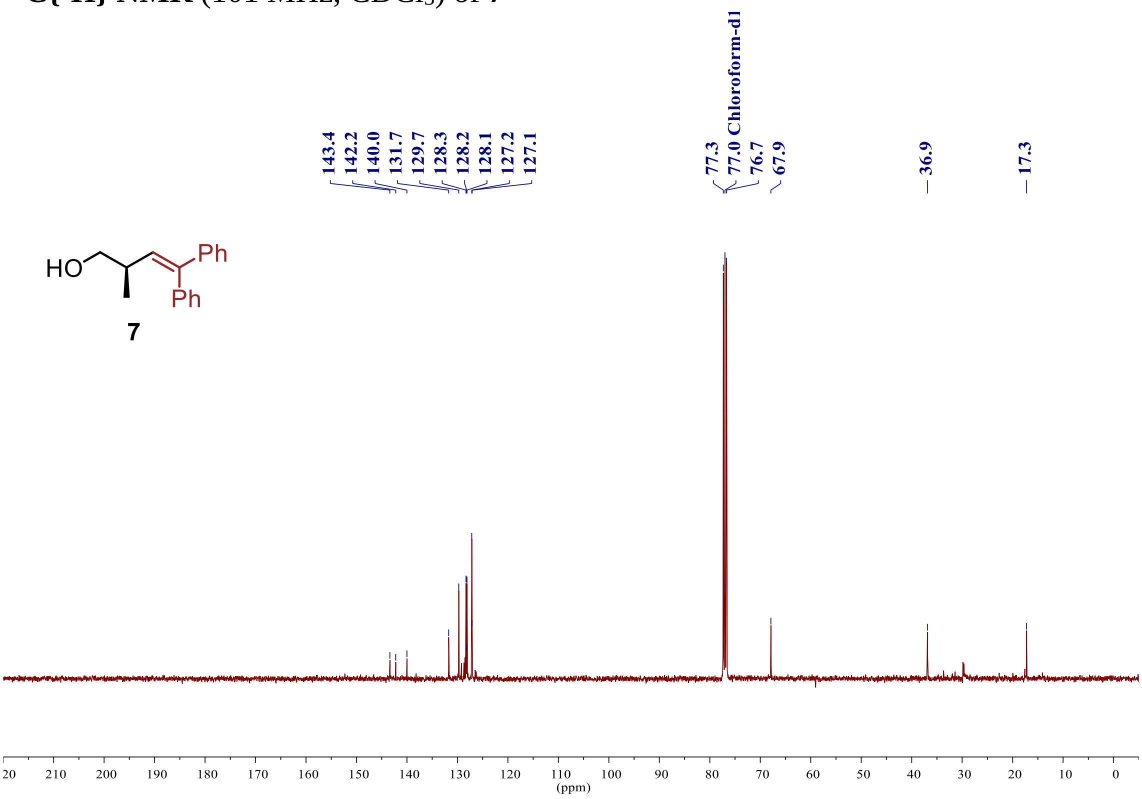

Back to contents

^{1}H NMR (400 MHz, $CDCl_3$) of **S4**, See procedure

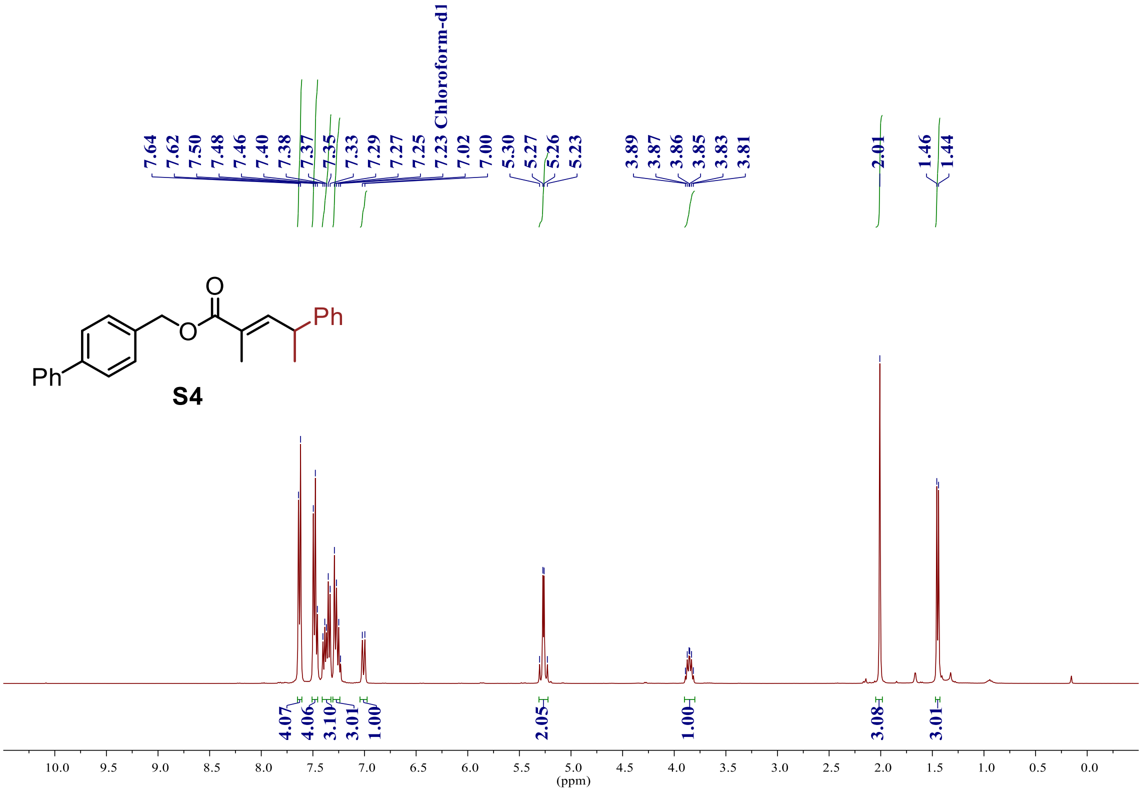

^{13}C{^{1}H} NMR (101 MHz, $CDCl_3$) of **S4**

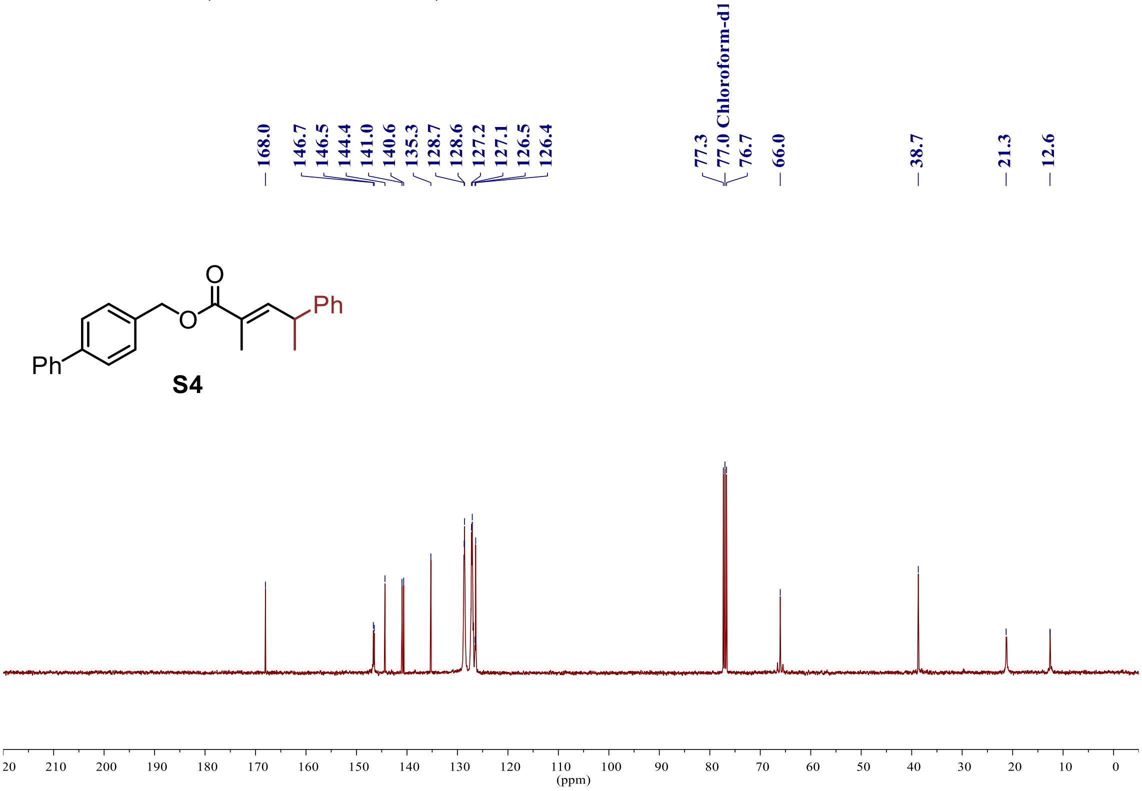

[Back to contents]

^{1}H NMR (300 MHz, $CDCl_3$) of **8**, See procedure

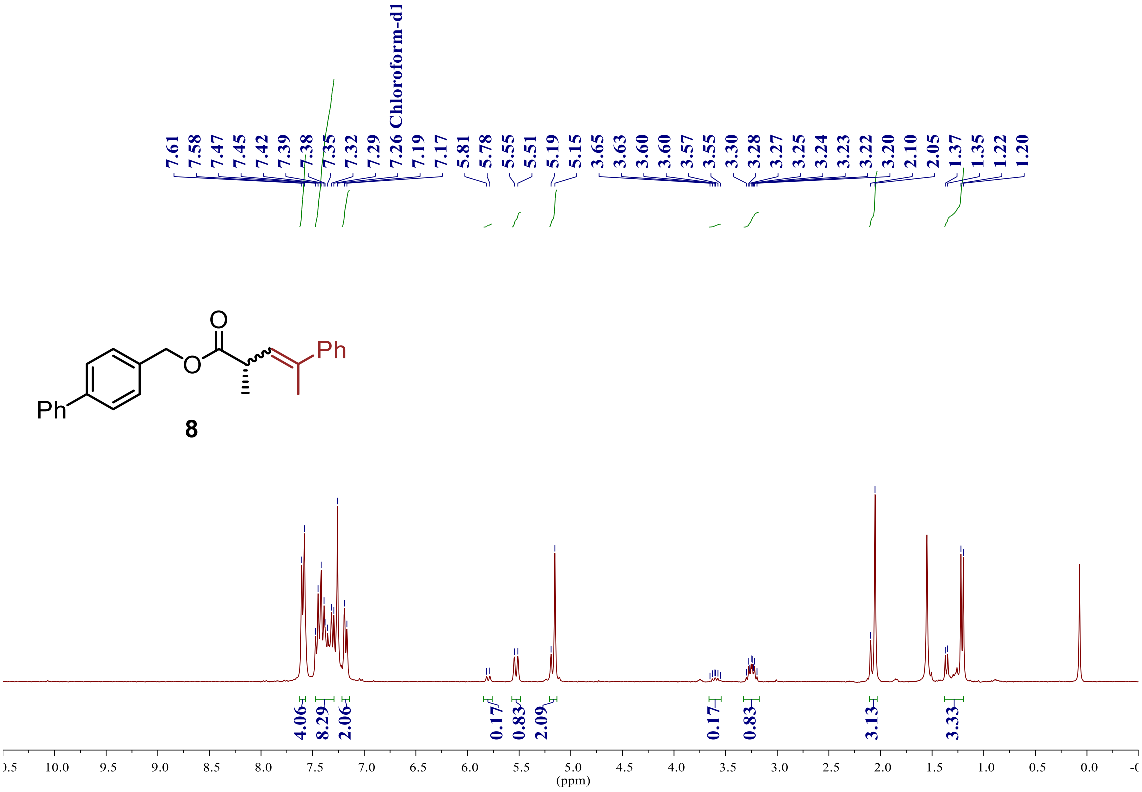

^{13}C{^{1}H} NMR (101 MHz, $CDCl_3$) of **8**

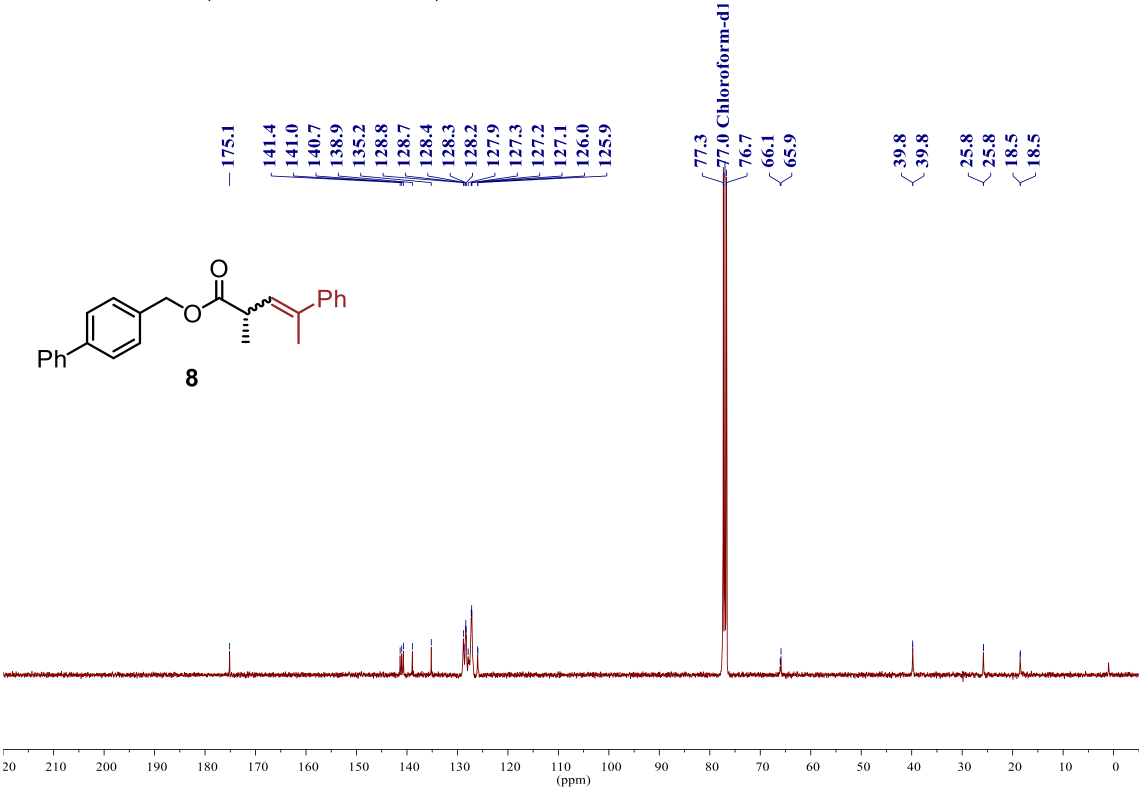

^{1}H NMR (300 MHz, $CDCl_3$) of **9**, See procedure

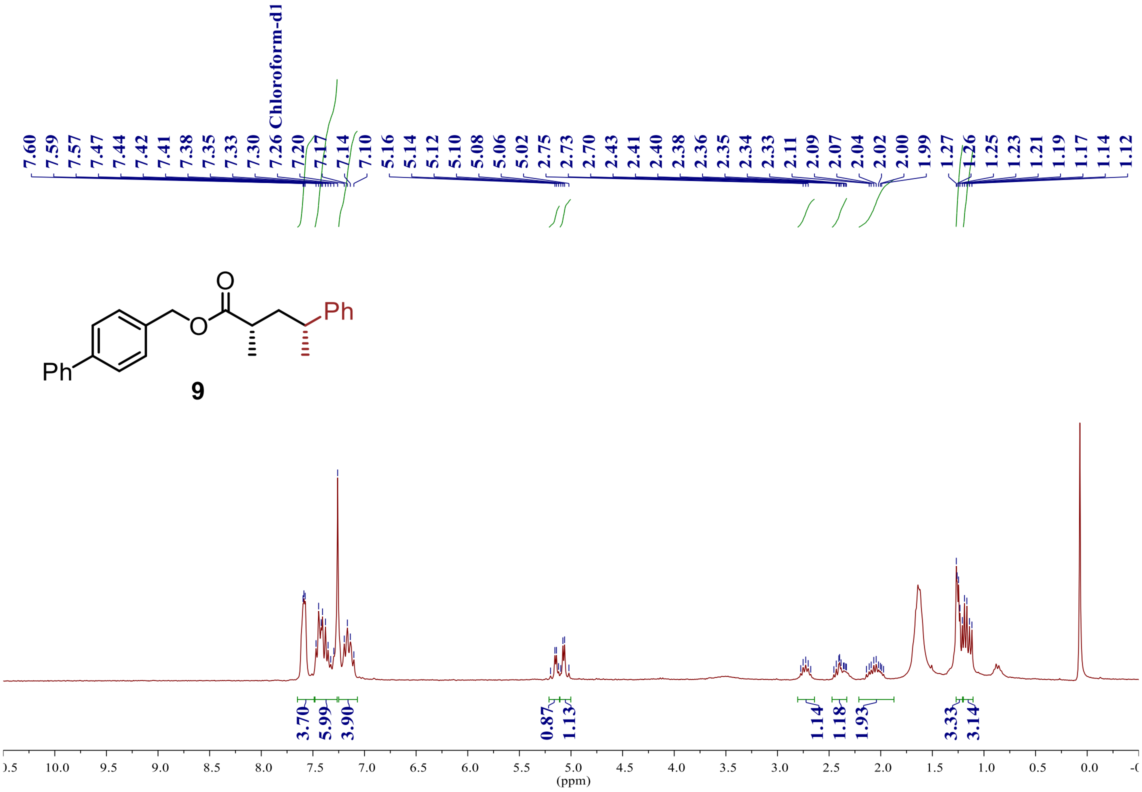

^{13}C{^{1}H} NMR (101 MHz, $CDCl_3$) of **9**

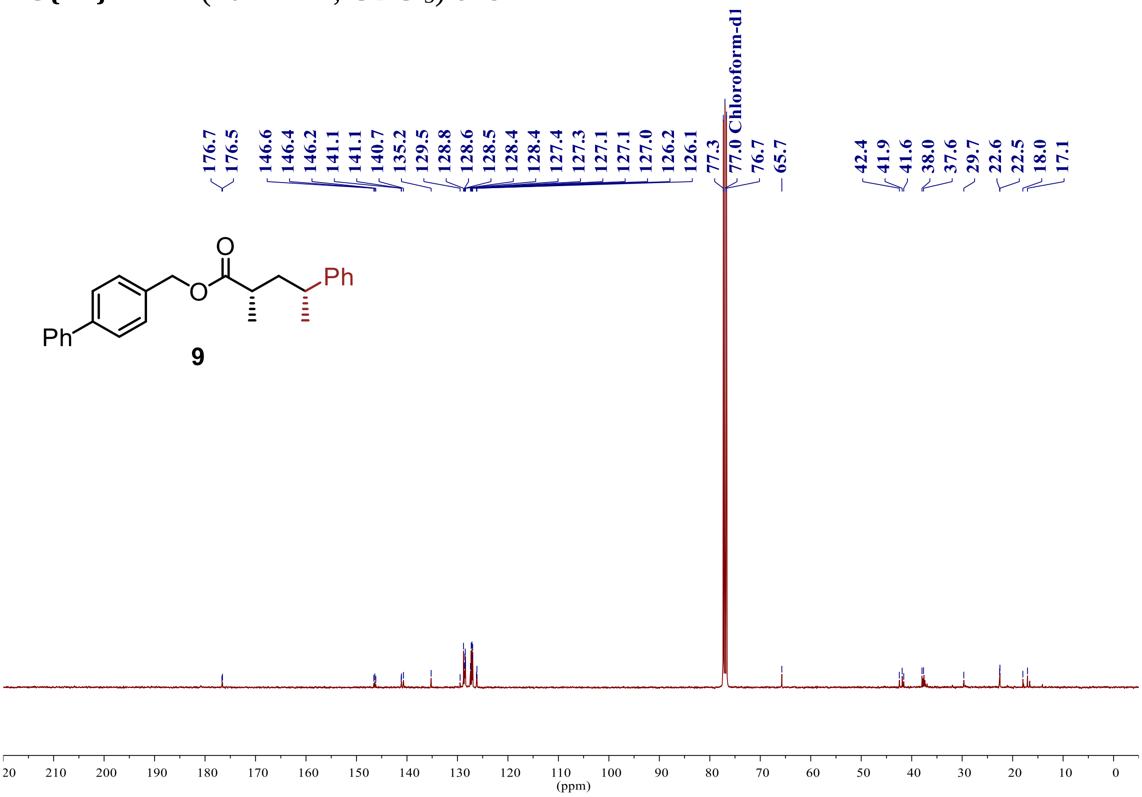

Back to contents

10. HPLC Spectra

Chiral HPLC (Chiralpak® OJ-3, Hexane/^{i}PrOH = 90:10, 0.9 mL/min) of **2a**

See procedure

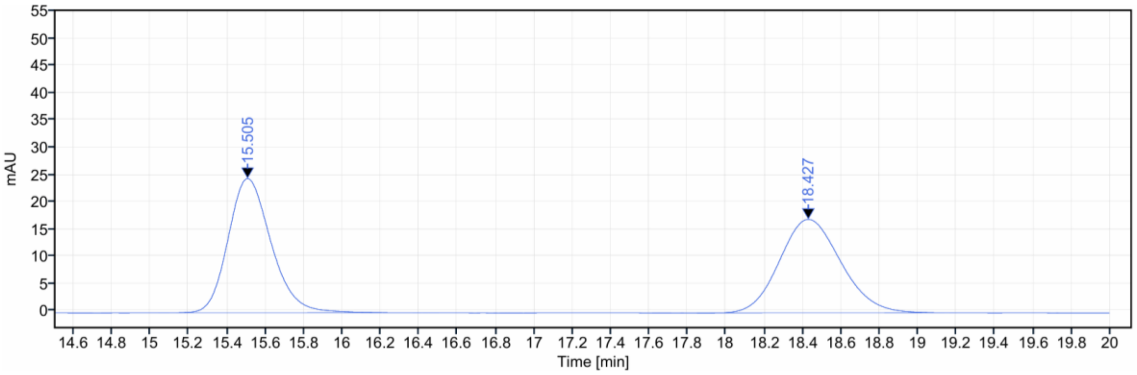

Signal: DAD1A,Sig=250,4 Ref=off

RT [min]	Type	Width [min]	Area	Height	Area%	Name
15.505	MM m	0.24	382.66	24.63	50.02	
18.427	MM m	0.35	382.32	17.17	49.98	

Chiral HPLC (Chiralpak® OJ-3, Hexane/^{i}PrOH = 90:10, 0.9 mL/min) of (*S*)-**2a**

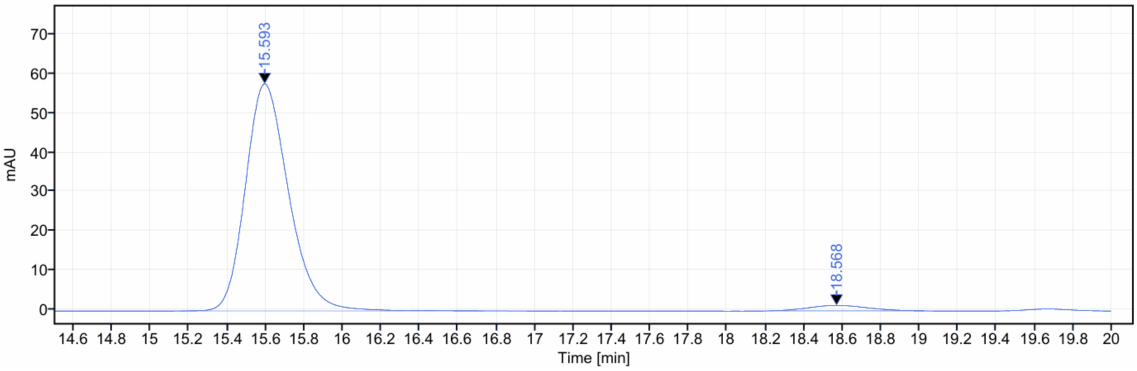

Signal: DAD1A,Sig=250,4 Ref=off

RT [min]	Type	Width [min]	Area	Height	Area%	Name
15.593	BM m	0.24	898.75	57.54	96.87	
18.568	MM m	0.32	29.08	1.37	3.13	

Chiral HPLC (Chiralpak® OJ-3, Hexane/^{i}PrOH = 90:10, 0.9 mL/min) of (*R*)-**2a**

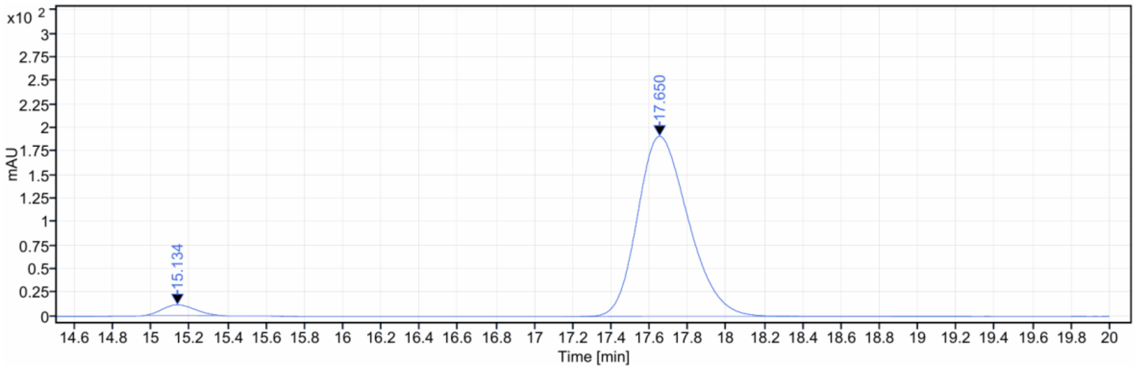

Signal: DAD1A,Sig=250,4 Ref=off

RT [min]	Type	Width [min]	Area	Height	Area%	Name
15.134	MM m	0.18	131.87	11.27	3.61	
17.650	MM m	0.28	3523.35	191.50	96.39	

Back to contents

Chiral HPLC (Chiralpak® OJ-3, Hexane/^{i}PrOH = 90:10, 0.8 mL/min) of **2b**

See procedure

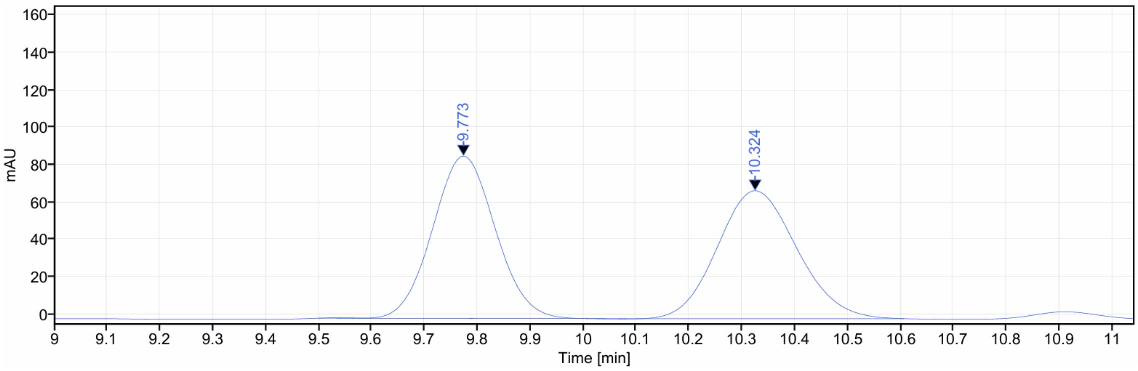

Signal: DAD1A,Sig=250,4 Ref=off

RT [min]	Type	Width [min]	Area	Height	Area%	Name
9.773	MM m	0.13	716.97	86.98	50.01	
10.324	MM m	0.16	716.71	68.51	49.99	

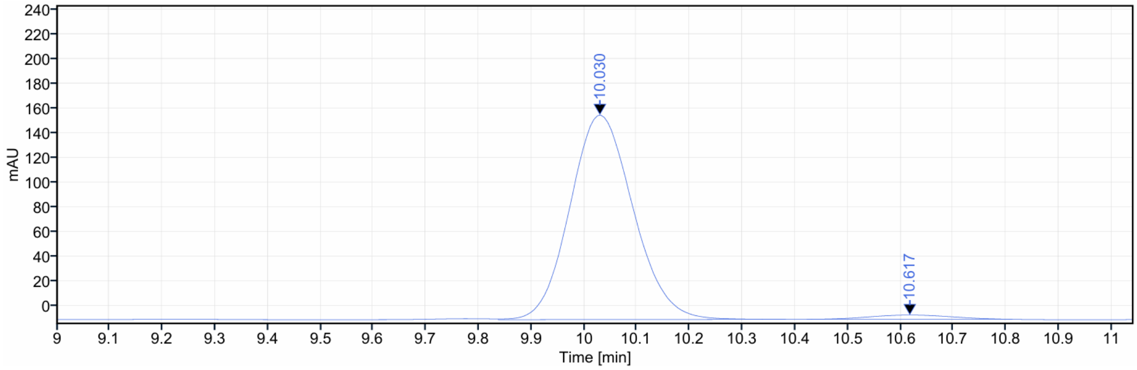

Signal: DAD1A,Sig=250,4 Ref=off

RT [min]	Type	Width [min]	Area	Height	Area%	Name
10.030	VM m	0.13	1382.89	165.95	97.32	
10.617	MM m	0.17	38.12	3.50	2.68	

Back to contents

Chiral HPLC (Chiralpak® OJ-3, Hexane/^{i}PrOH = 99:01, 0.9 mL/min) of **2c**

See procedure

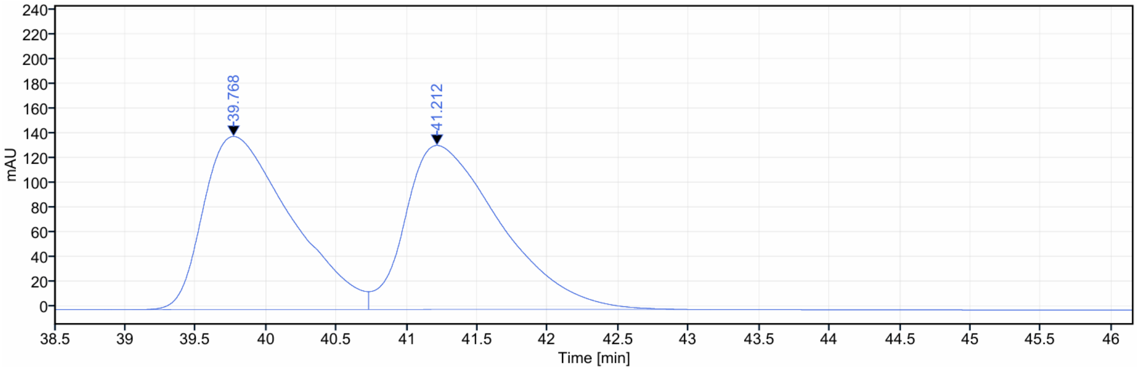

Signal: DAD1A,Sig=250,4 Ref=off

RT [min]	Type	Width [min]	Area	Height	Area%	Name
39.768	BM m	0.65	6064.19	140.17	49.35	
41.212	MM m	0.73	6223.34	132.79	50.65	

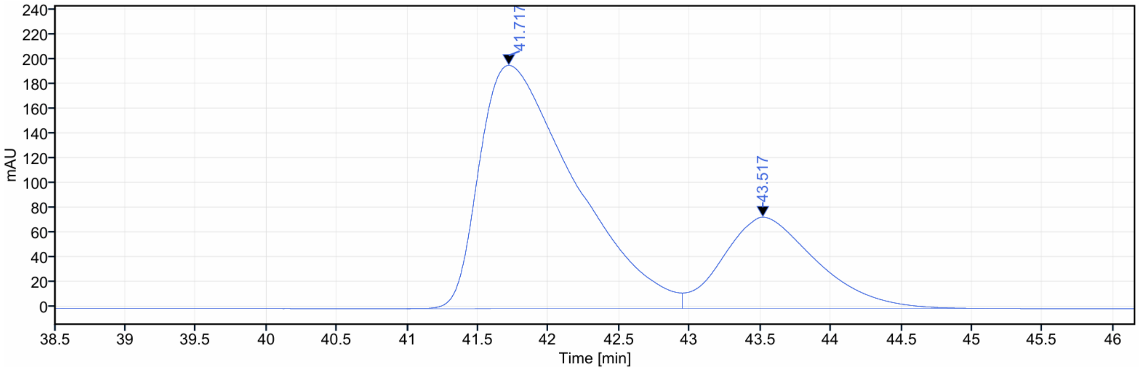

Signal: DAD1A,Sig=250,4 Ref=off

RT [min]	Type	Width [min]	Area	Height	Area%	Name
41.717	BM m	0.69	9243.25	196.80	73.10	
43.517	MM m	0.69	3401.16	73.93	26.90	

Back to contents

Chiral HPLC (Chiralpak® IA-3, Hexane/^{i}PrOH = 99:01, 0.5 mL/min) of **2d**
See procedure

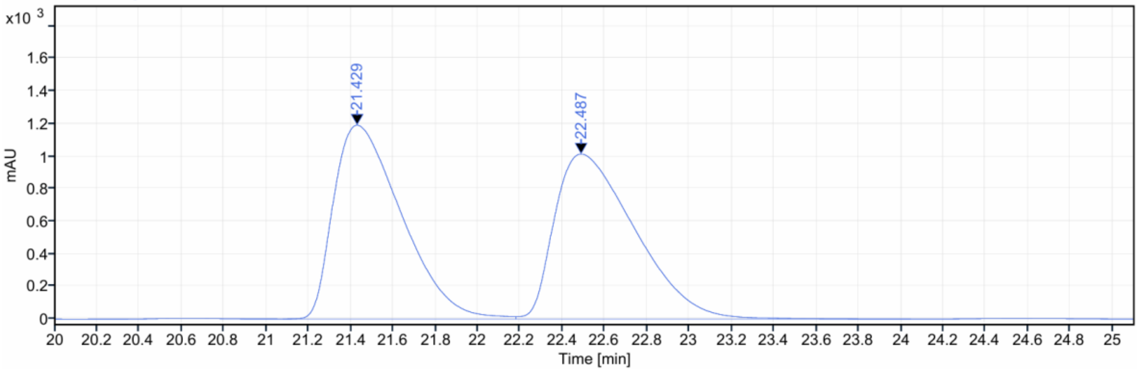

Signal:	DAD1A,Sig=250,4 Ref=off					
**RT [min]**	**Type**	**Width [min]**	**Area**	**Height**	**Area%**	**Name**
21.429	BV	1.10	26644.83	1193.60	49.87	
22.487	VB	1.87	26780.12	1015.71	50.13	

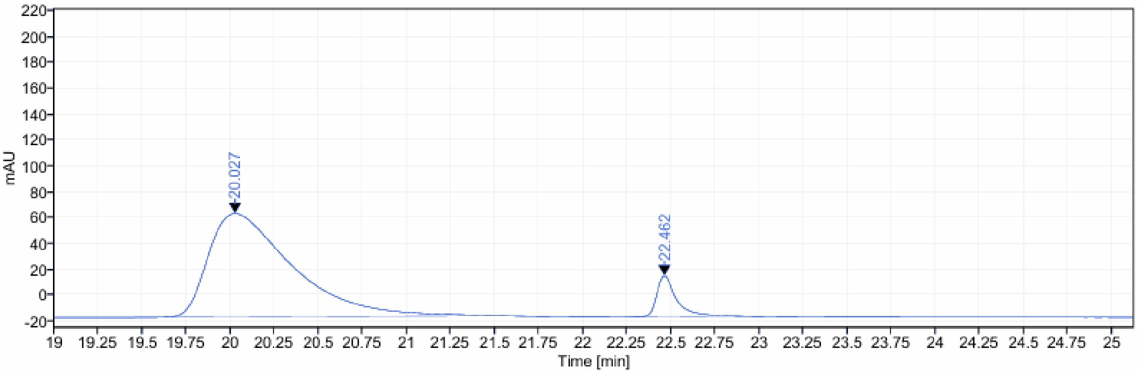

Signal:	DAD1A,Sig=250,4 Ref=off					
**RT [min]**	**Type**	**Width [min]**	**Area**	**Height**	**Area%**	**Name**
20.027	MM m	0.50	2638.73	79.97	91.56	
22.462	MM m	0.11	243.18	31.65	8.44	

Back to contents

Chiral HPLC (Chiralpak® IA-3, Hexane/^{i}PrOH = 99:01, 0.5 mL/min) of **2e**

See procedure

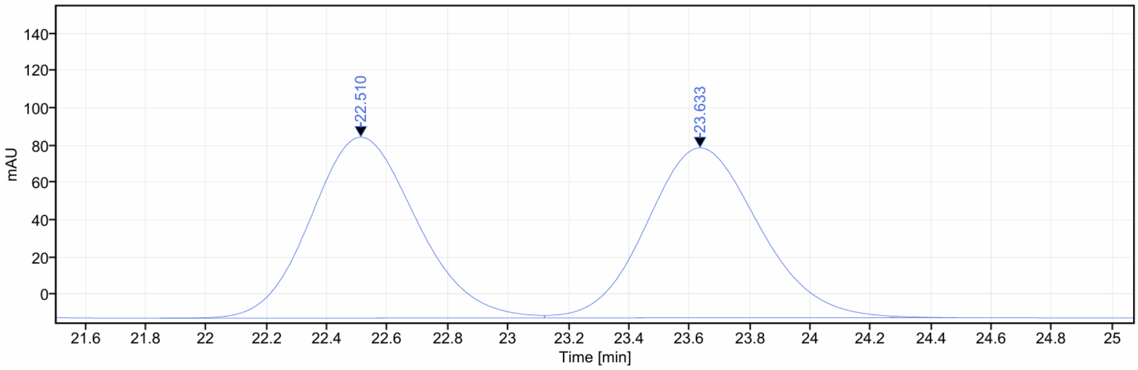

Signal: DAD1A,Sig=250,4 Ref=off

RT [min]	Type	Width [min]	Area	Height	Area%	Name
22.510	BM m	0.38	2363.96	96.94	50.00	
23.633	MM m	0.40	2363.85	91.06	50.00	

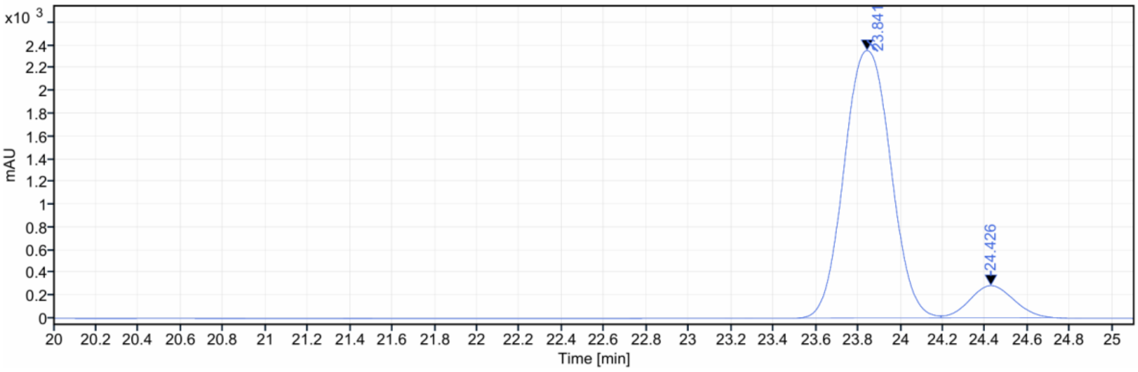

Signal: DAD1A,Sig=250,4 Ref=off

RT [min]	Type	Width [min]	Area	Height	Area%	Name
23.841	MM m	0.24	35805.39	2350.41	89.84	
24.426	MM m	0.23	4050.58	281.65	10.16	

Back to contents

Chiral HPLC (Chiralpak® IA-3, Hexane/^{i}PrOH = 99:01, 0.8 mL/min) of **2f**

See procedure

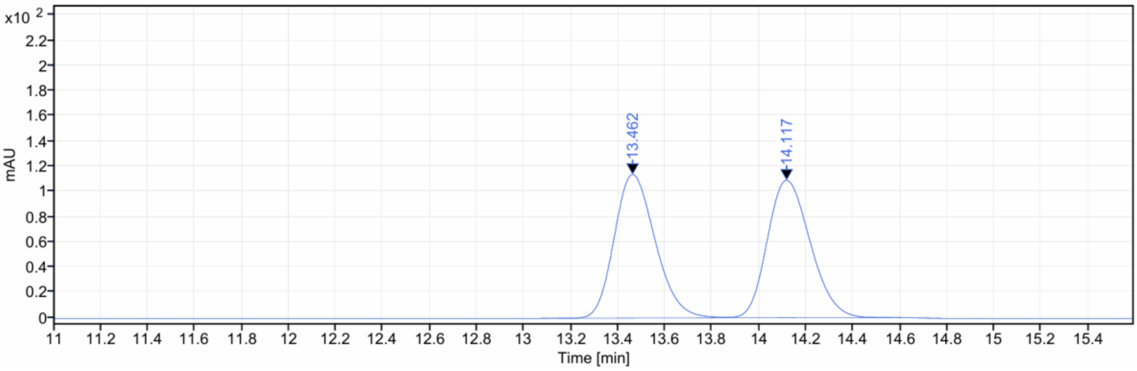

Signal: DAD1A,Sig=250,4 Ref=off

RT [min]	Type	Width [min]	Area	Height	Area%	Name
13.462	MM m	0.19	1384.22	114.11	50.00	
14.117	MM m	0.20	1384.22	109.27	50.00	

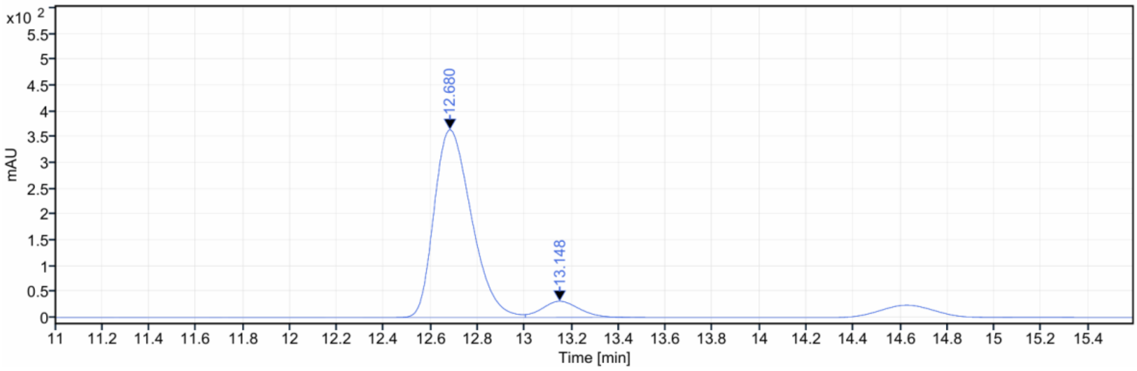

Signal: DAD1A,Sig=250,4 Ref=off

RT [min]	Type	Width [min]	Area	Height	Area%	Name
12.680	MM m	0.17	4027.15	364.41	92.15	
13.148	MM m	0.17	343.18	31.28	7.85	

Back to contents

Chiral HPLC (Chiralpak® IA-3, Hexane/^{i}PrOH = 99:01, 0.8 mL/min) of **2g**

See procedure

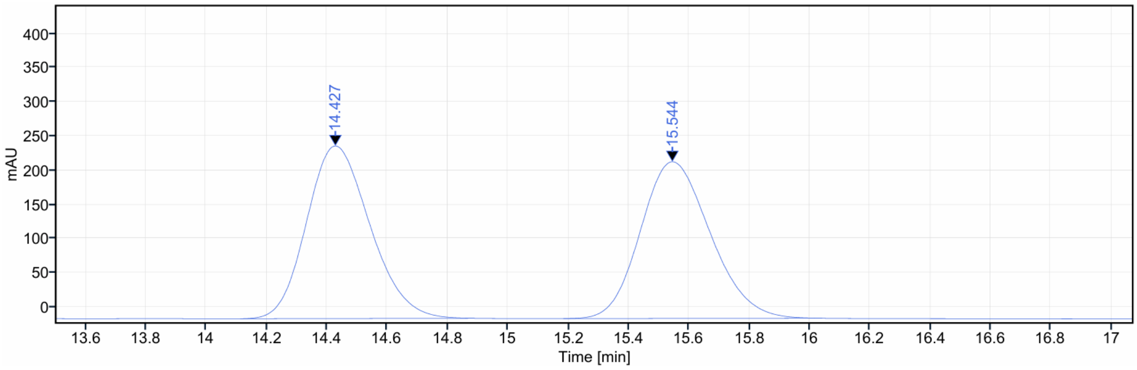

Signal: DAD1A,Sig=250,4 Ref=off

RT [min]	Type	Width [min]	Area	Height	Area%	Name
14.427	MM m	0.23	3691.00	252.83	50.00	
15.544	MM m	0.25	3690.94	229.55	50.00	

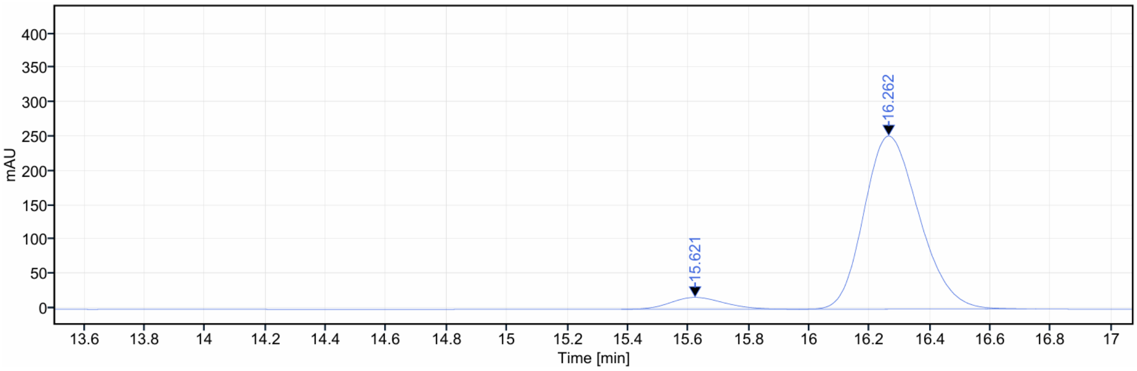

Signal: DAD1A,Sig=250,4 Ref=off

RT [min]	Type	Width [min]	Area	Height	Area%	Name
15.621	MM m	0.19	215.51	17.35	6.31	
16.262	VM m	0.20	3201.21	252.75	93.69	

[Back to contents](#)

Chiral HPLC (Chiralpak® IC-3, Hexane/^{i}PrOH = 93:07, 0.9 mL/min) of **2h**

[See procedure](#)

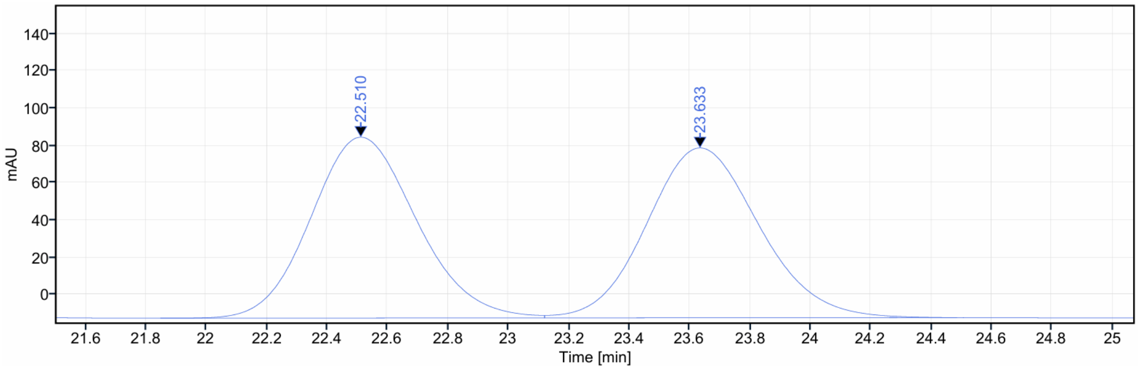

Signal: DAD1A,Sig=250,4 Ref=off

RT [min]	Type	Width [min]	Area	Height	Area%	Name
22.510	BM m	0.38	2363.96	96.94	50.00	
23.633	MM m	0.40	2363.85	91.06	50.00	

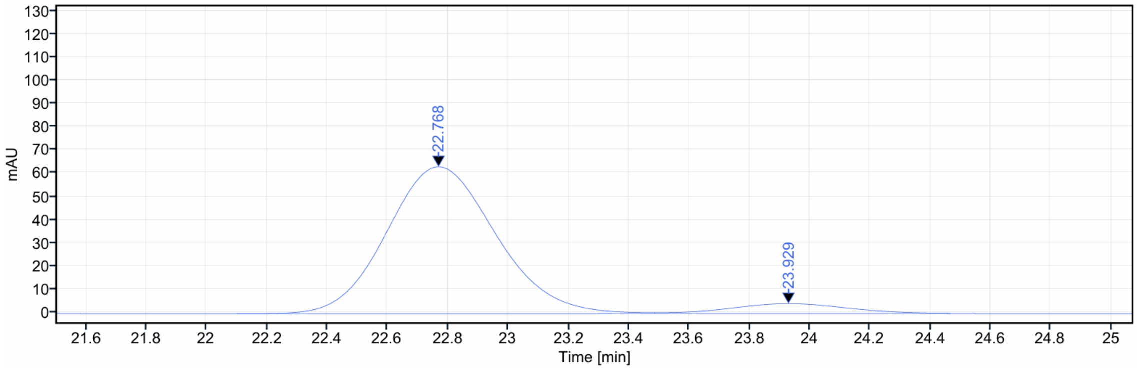

Signal: DAD1A,Sig=250,4 Ref=off

RT [min]	Type	Width [min]	Area	Height	Area%	Name
22.768	BM m	0.39	1589.77	63.18	93.46	
23.929	MM m	0.39	111.25	4.24	6.54	

Chiral HPLC (Chiralpak® IA-3, Hexane/^{i}PrOH = 90:10, 0.9 mL/min) of **2i**

See procedure

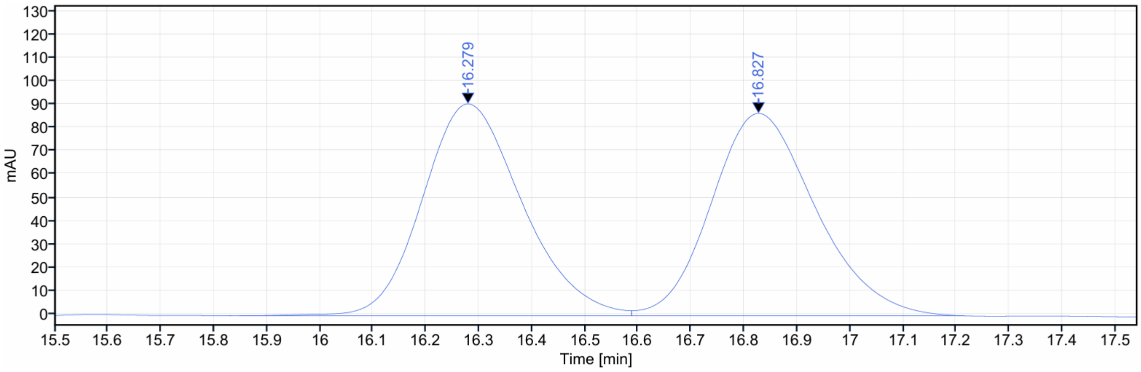

Signal: DAD1A,Sig=250,4 Ref=off

RT [min]	Type	Width [min]	Area	Height	Area%	Name
16.279	MM m	0.20	1187.55	90.78	50.00	
16.827	MM m	0.21	1187.54	86.72	50.00	

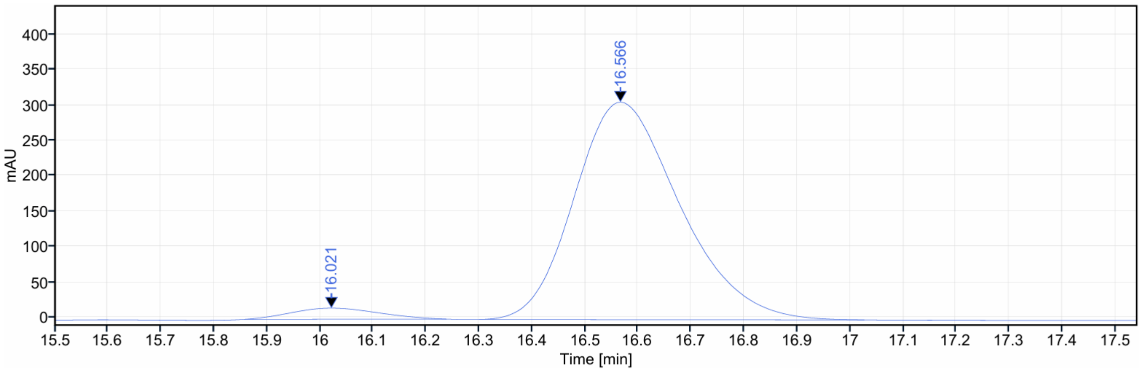

Signal: DAD1A,Sig=250,4 Ref=off

RT [min]	Type	Width [min]	Area	Height	Area%	Name
16.021	MM m	0.18	179.84	15.74	4.06	
16.566	MM m	0.21	4245.48	306.87	95.94	

Back to contents

Chiral HPLC (Chiralpak® IA-3, Hexane/^{i}PrOH = 99:01, 0.7 mL/min) of **2k**

See procedure

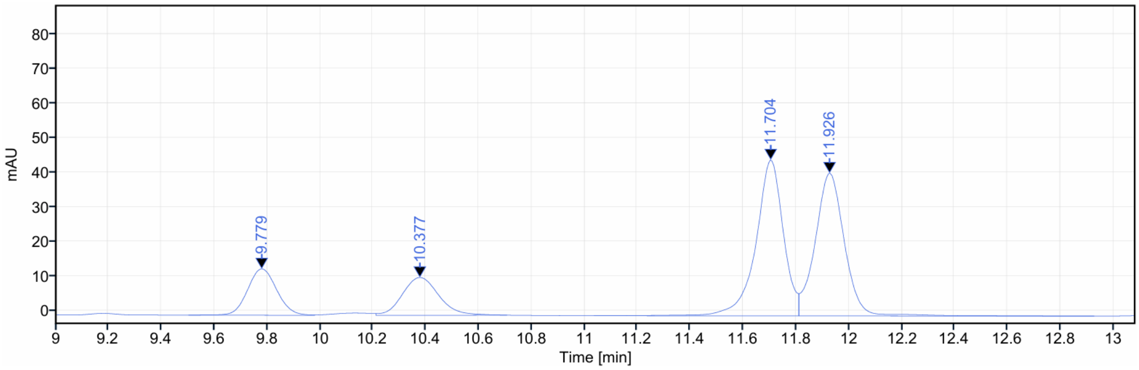

Signal: DAD1A,Sig=250,4 Ref=off

RT [min]	Type	Width [min]	Area	Height	Area%	Name
9.779	BV	0.48	102.62	13.41	12.06	
10.377	VM m	0.15	104.86	10.95	12.33	
11.704	BV	0.77	325.81	45.08	38.30	
11.926	VB	1.12	317.39	41.28	37.31	

Chiral HPLC (Chiralpak® IA-3, Hexane/^{i}PrOH = 99:01, 0.7 mL/min) of (*S*)-**2k**

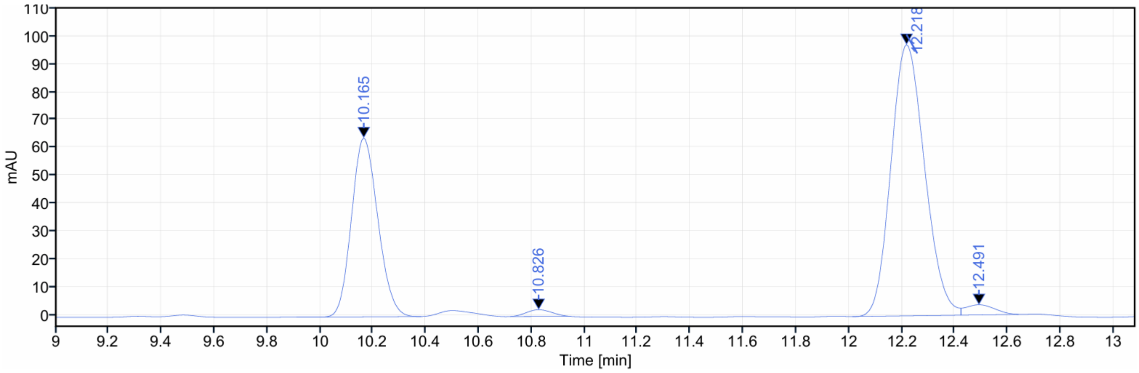

Signal: DAD1A,Sig=250,4 Ref=off

RT [min]	Type	Width [min]	Area	Height	Area%	Name
10.165	BM m	0.11	444.08	64.18	32.87	
10.826	MM m	0.11	16.34	2.44	1.21	
12.218	BM m	0.14	862.56	97.29	63.85	
12.491	MM m	0.12	27.86	3.64	2.06	

Back to contents

Chiral HPLC (Chiralpak® IA-3, Hexane/^{i}PrOH = 99:01, 0.7 mL/min) of (*R*)-**2k**

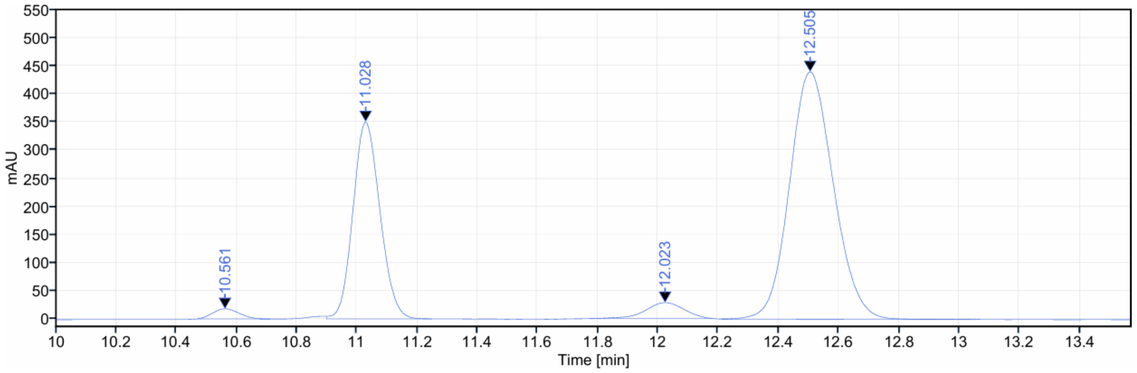

Signal: DAD1A,Sig=250,4 Ref=off

RT [min]	Type	Width [min]	Area	Height	Area%	Name
10.561	MM m	0.10	110.14	18.07	1.57	
11.028	MM m	0.10	2176.02	349.87	31.11	
12.023	MM m	0.14	256.96	28.51	3.67	
12.505	MM m	0.16	4450.81	439.09	63.64	

Back to contents

Chiral HPLC (Chiralpak® IA-3, Hexane/^{i}PrOH = 99:01, 0.7 mL/min) of **2l**

See procedure

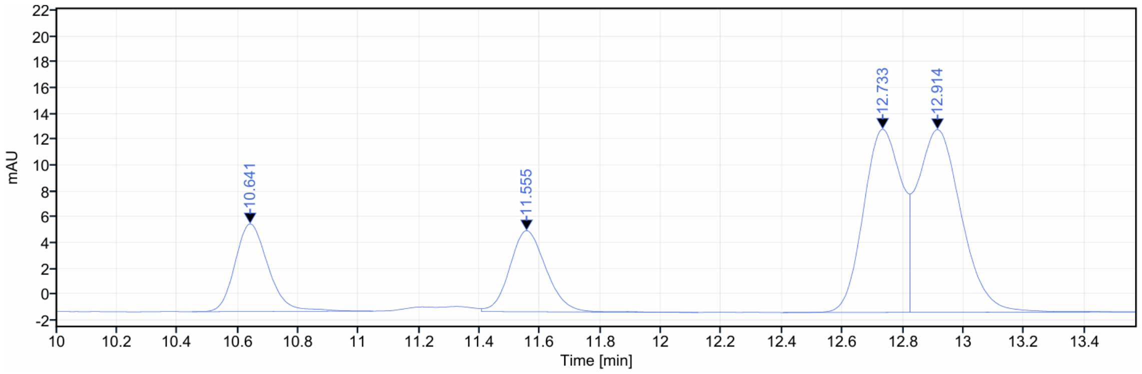

Signal: DAD1A,Sig=250,4 Ref=off

RT [min]	Type	Width [min]	Area	Height	Area%	Name
10.641	BB	0.60	51.66	6.80	13.88	
11.555	VB	0.72	52.56	6.28	14.13	
12.733	BV	0.42	124.52	14.19	33.46	
12.914	VB	0.76	143.36	14.16	38.53	

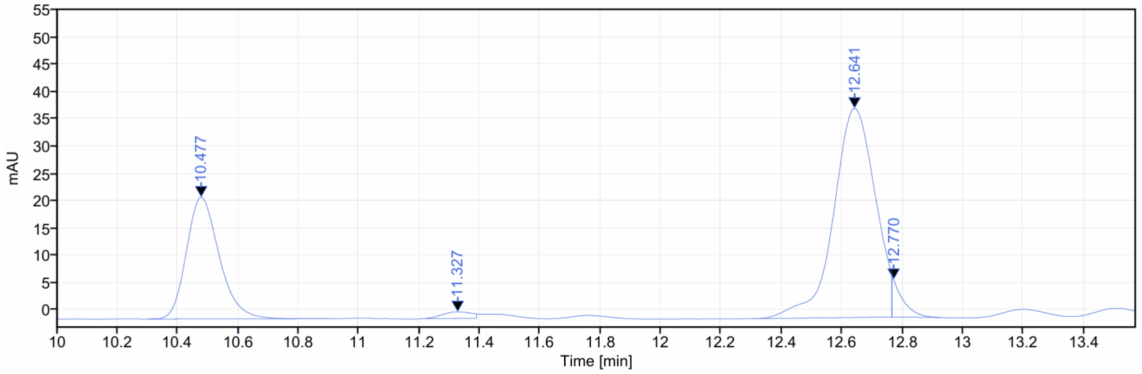

Signal: DAD1A,Sig=250,4 Ref=off

RT [min]	Type	Width [min]	Area	Height	Area%	Name
10.477	BM m	0.11	168.32	22.33	30.12	
11.327	MM m	0.10	8.01	1.22	1.43	
12.641	MM m	0.15	365.06	38.34	65.32	
12.770	MM m	0.04	17.51	6.89	3.13	

Back to contents

Chiral HPLC (Chiralpak® IA-3, Hexane/^{i}PrOH = 97:03, 0.5 mL/min) of **2m**

See procedure

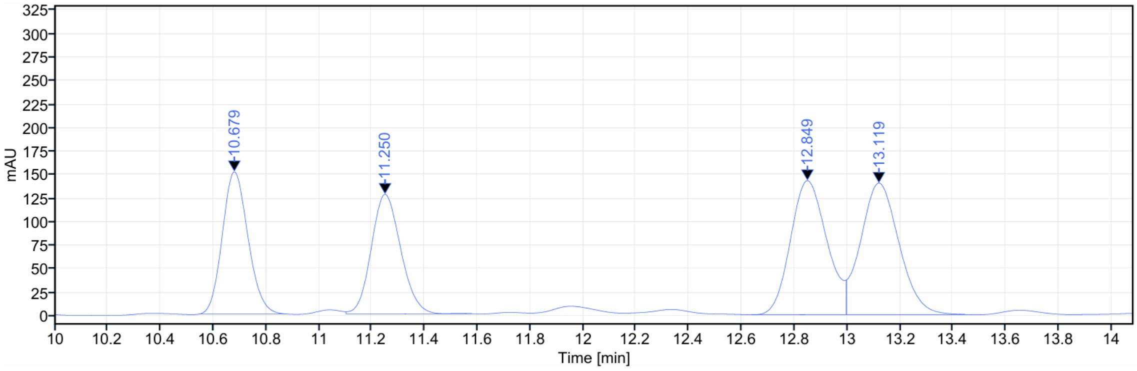

Signal: DAD1A,Sig=250,4 Ref=off

RT [min]	Type	Width [min]	Area	Height	Area%	Name
10.679	MM m	0.10	1022.05	151.50	21.35	
11.250	VM m	0.12	987.43	127.47	20.63	
12.849	BM m	0.15	1369.13	142.67	28.60	
13.119	MM m	0.16	1408.48	139.97	29.42	

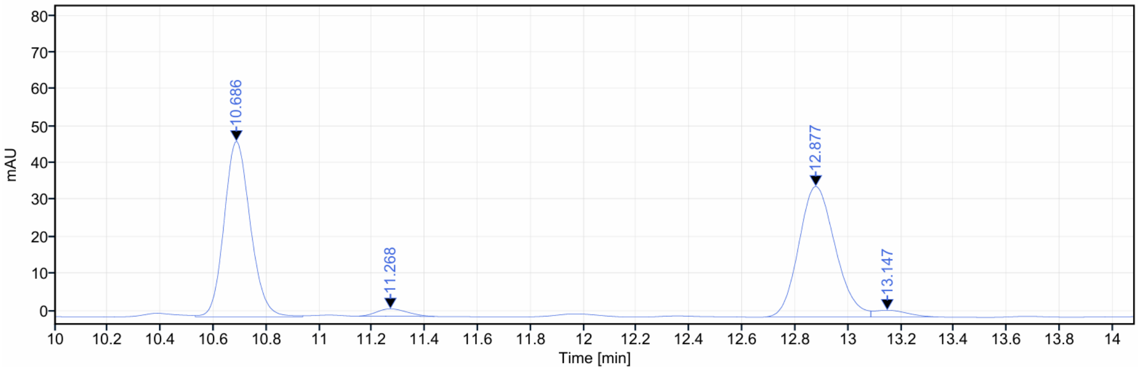

Signal: DAD1A,Sig=250,4 Ref=off

RT [min]	Type	Width [min]	Area	Height	Area%	Name
10.686	VV	0.41	327.24	47.55	46.95	
11.268	MM m	0.12	15.91	2.01	2.28	
12.877	MM m	0.15	337.87	35.38	48.47	
13.147	MM m	0.13	15.98	1.83	2.29	

Back to contents

Chiral HPLC (Chiralpak® IA-3, Hexane/^{i}PrOH = 97:03, 0.5 mL/min) of **2n**

See procedure

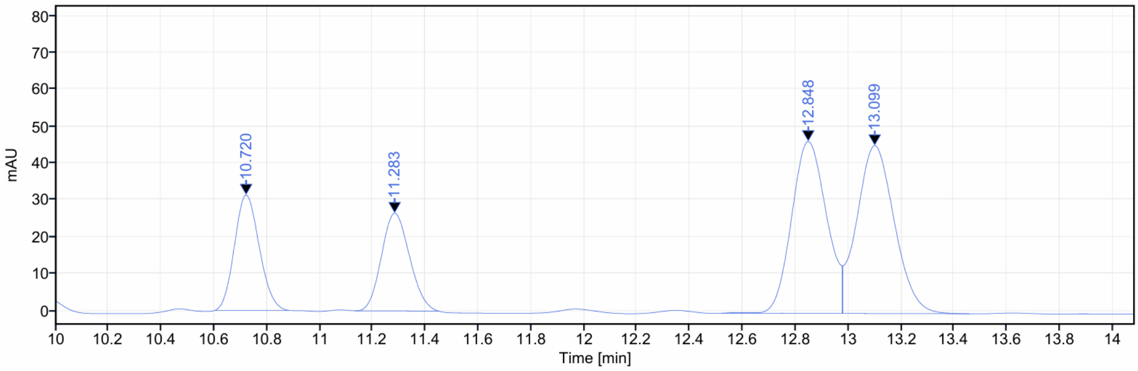

Signal: DAD1A,Sig=250,4 Ref=off

RT [min]	Type	Width [min]	Area	Height	Area%	Name
10.720	MM m	0.10	204.09	31.30	15.95	
11.283	MM m	0.11	195.61	26.52	15.29	
12.848	MM m	0.14	429.08	46.64	33.54	
13.099	MM m	0.15	450.67	45.55	35.22	

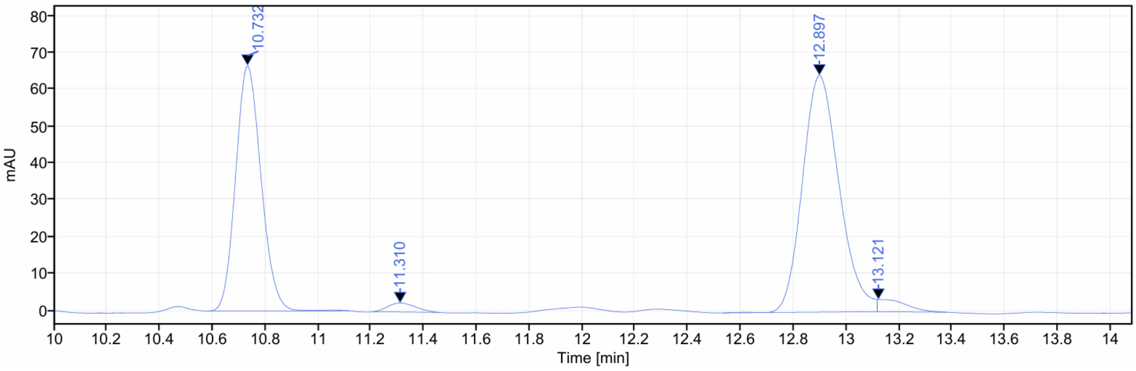

Signal: DAD1A,Sig=250,4 Ref=off

RT [min]	Type	Width [min]	Area	Height	Area%	Name
10.732	MM m	0.10	445.14	66.53	40.15	
11.310	MM m	0.12	17.88	2.42	1.61	
12.897	MM m	0.15	620.83	64.20	55.99	
13.121	MM m	0.13	24.94	3.29	2.25	

Back to contents

Chiral HPLC (Chiralpak® OJ-3, Hexane/^{i}PrOH = 95:05, 0.5 mL/min) of **2n’**

See procedure

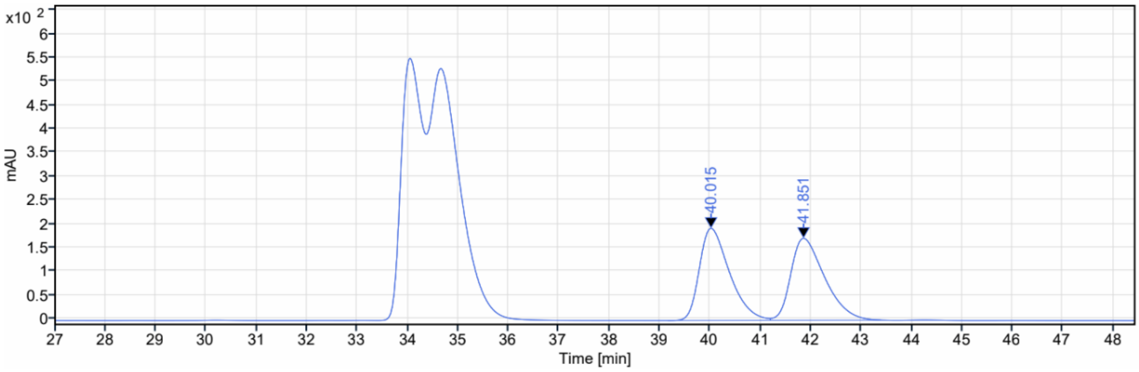

Signal: DAD1A,Sig=250,4 Ref=off

RT [min]	Type	Width [min]	Area	Height	Area%	Name
40.015	BM m	0.63	8055.72	194.30	50.00	
41.851	MM m	0.71	8055.48	172.89	50.00	

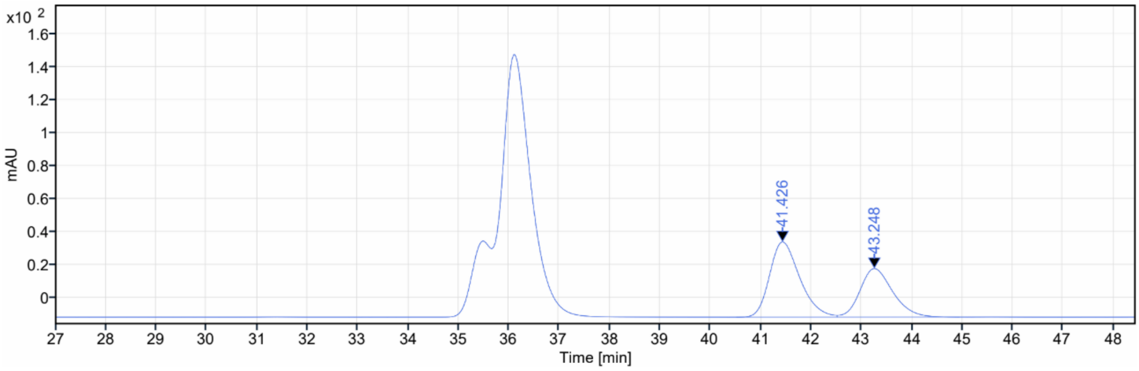

Signal: DAD1A,Sig=250,4 Ref=off

RT [min]	Type	Width [min]	Area	Height	Area%	Name
41.426	BM m	0.63	1902.84	45.66	58.84	
43.248	MM m	0.68	1331.06	29.48	41.16	

Back to contents

Chiral HPLC (Chiralpak® IC-3, Hexane/^{i}PrOH = 93:07, 0.7 mL/min) of **2o**

See procedure

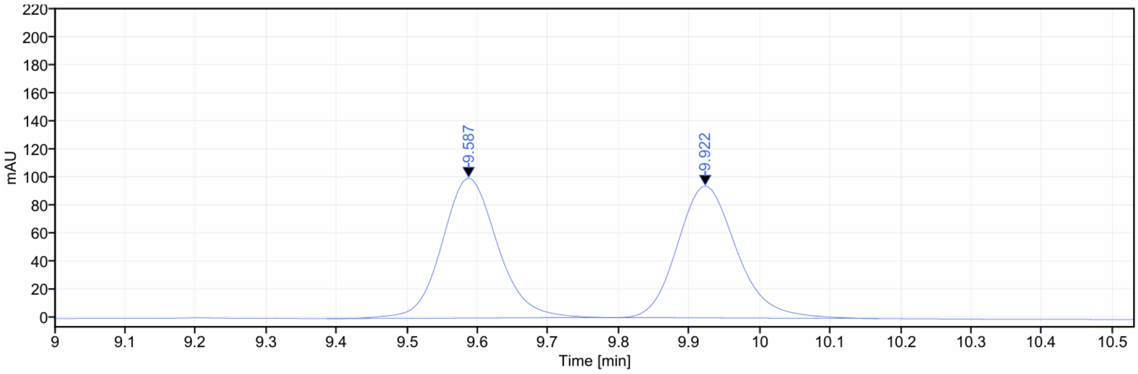

Signal: DAD1A,Sig=254,4 Ref=off

RT [min]	Type	Width [min]	Area	Height	Area%	Name
9.587	BM m	0.08	546.14	99.67	49.96	
9.922	MM m	0.09	547.11	93.87	50.04	

Chiral HPLC (Chiralpak® IC-3, Hexane/^{i}PrOH = 93:07, 0.7 mL/min) of (*R*)-**2o**

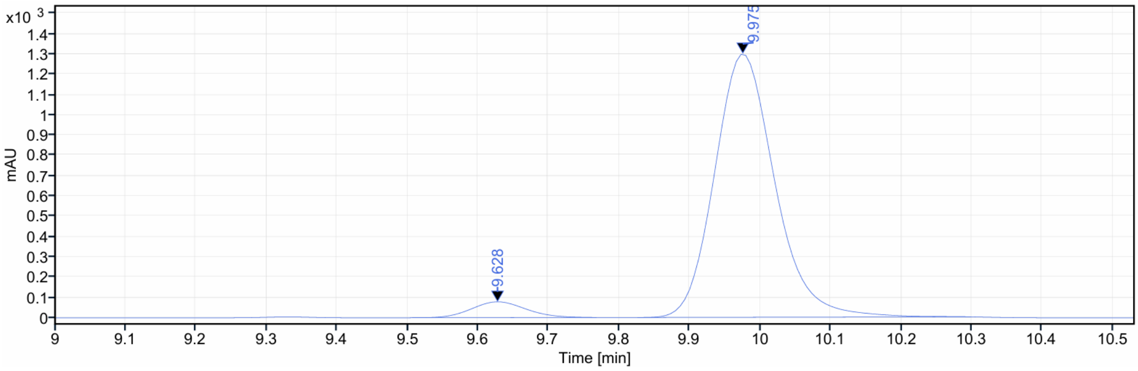

Signal: DAD1A,Sig=254,4 Ref=off

RT [min]	Type	Width [min]	Area	Height	Area%	Name
9.628	MM m	0.08	419.89	78.26	5.17	
9.975	MM m	0.09	7702.09	1300.97	94.83	

Chiral HPLC (Chiralpak® IC-3, Hexane/^{i}PrOH = 93:07, 0.7 mL/min) of (*S*)-**2o**

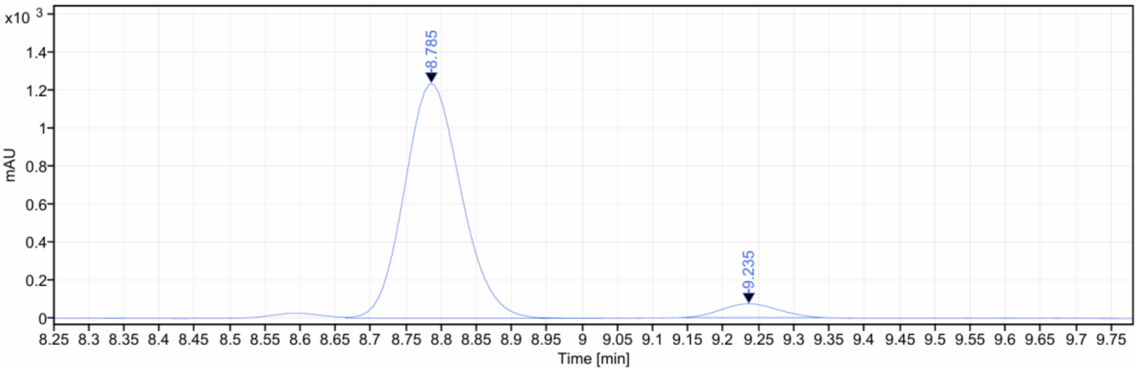

Signal: DAD1A,Sig=250,4 Ref=off

RT [min]	Type	Width [min]	Area	Height	Area%	Name
8.785	VM m	0.08	6912.04	1241.11	94.34	
9.235	MM m	0.09	414.32	73.47	5.66	

Back to contents

Chiral HPLC (Chiralpak® IC-3, Hexane/^{i}PrOH = 99:01, 0.7 mL/min) of **2p**

See procedure

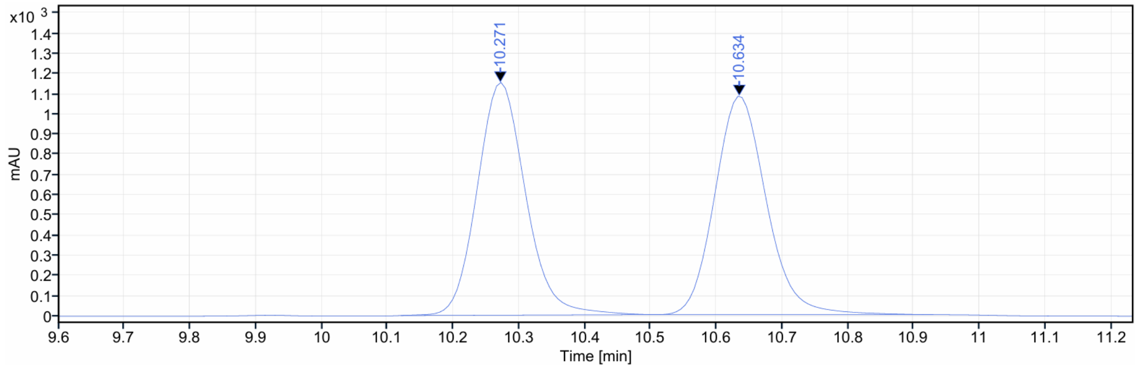

Signal: DAD1A,Sig=254,4 Ref=off

RT [min]	Type	Width [min]	Area	Height	Area%	Name
10.271	MM m	0.08	5895.57	1154.32	49.97	
10.634	MM m	0.08	5902.03	1085.12	50.03	

Chiral HPLC (Chiralpak® IC-3, Hexane/^{i}PrOH = 99:01, 0.7 mL/min) of (*R*)-**2p**

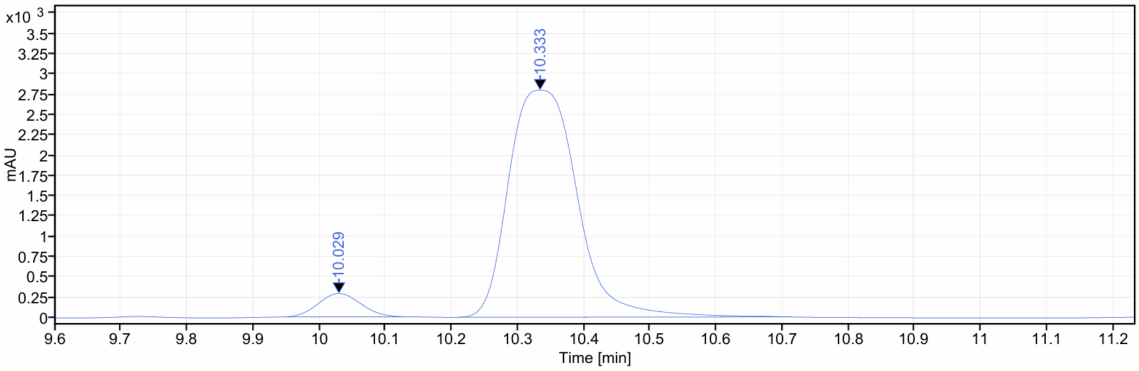

Signal: DAD1A,Sig=254,4 Ref=off

RT [min]	Type	Width [min]	Area	Height	Area%	Name
10.029	MM m	0.07	1269.02	287.06	5.93	
10.333	MM m	0.12	20145.19	2798.32	94.07	

Chiral HPLC (Chiralpak® IC-3, Hexane/^{i}PrOH = 99:01, 0.7 mL/min) of (*S*)-**2p**

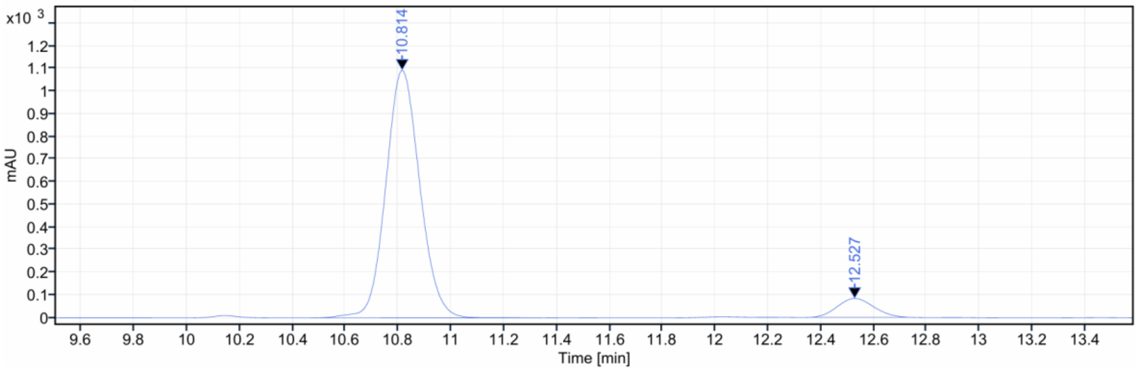

Signal: DAD1A,Sig=250,4 Ref=off

RT [min]	Type	Width [min]	Area	Height	Area%	Name
10.814	MM m	0.14	9777.48	1093.46	92.59	
12.527	MM m	0.15	782.90	82.73	7.41	

[Back to contents]

Chiral HPLC (Chiralpak® IC-3, Hexane/^{i}PrOH = 99:01, 0.7 mL/min) of **2q**

See procedure

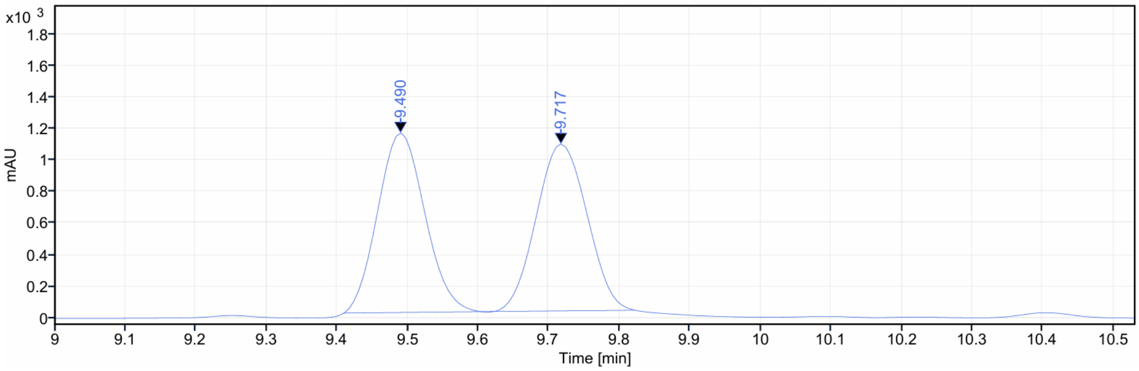

Signal: DAD1A,Sig=254,4 Ref=off

RT [min]	Type	Width [min]	Area	Height	Area%	Name
9.490	MM m	0.07	5265.94	1138.38	50.00	
9.717	MM m	0.08	5265.34	1058.38	50.00	

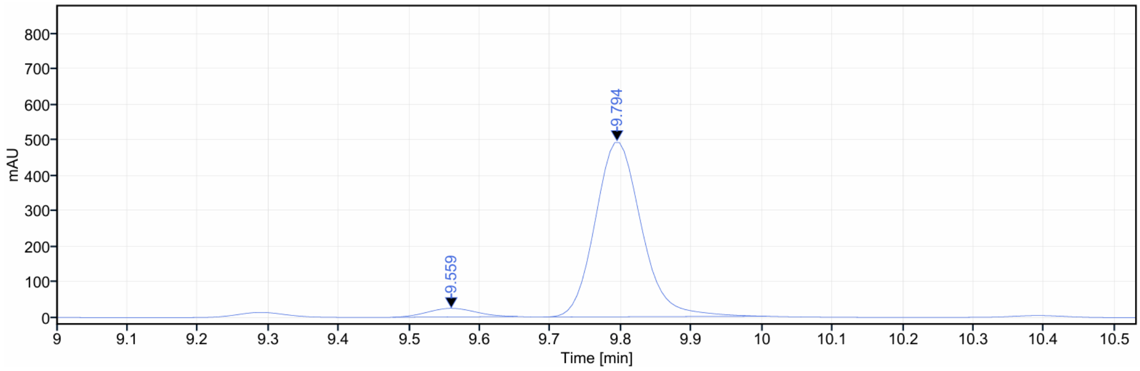

Signal: DAD1A,Sig=254,4 Ref=off

RT [min]	Type	Width [min]	Area	Height	Area%	Name
9.559	MM m	0.08	114.18	24.22	4.83	
9.794	MM m	0.07	2247.65	494.88	95.17	

Back to contents

Chiral HPLC (Chiralpak® IC-3, Hexane/^{i}PrOH = 93:07, 0.7 mL/min) of **2r**

See procedure

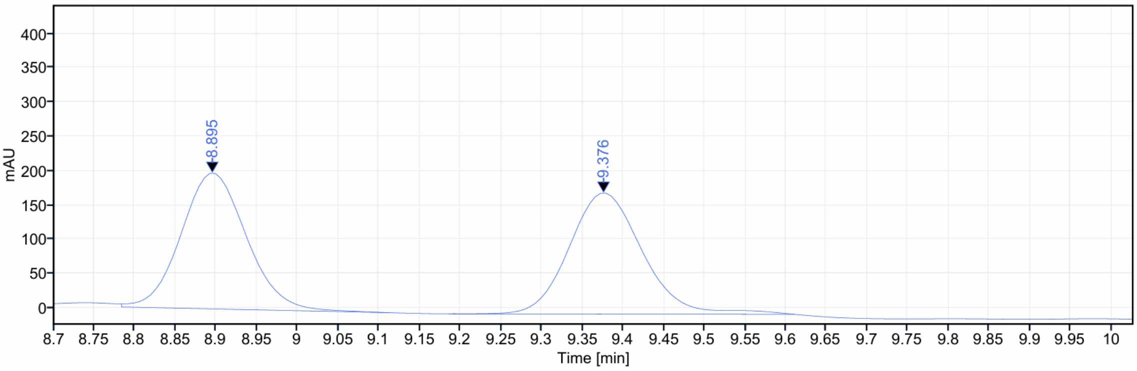

Signal: DAD1C,Sig=210,4 Ref=off

RT [min]	Type	Width [min]	Area	Height	Area%	Name
8.895	VM m	0.09	1109.08	198.66	50.00	
9.376	BM m	0.10	1109.20	177.10	50.00	

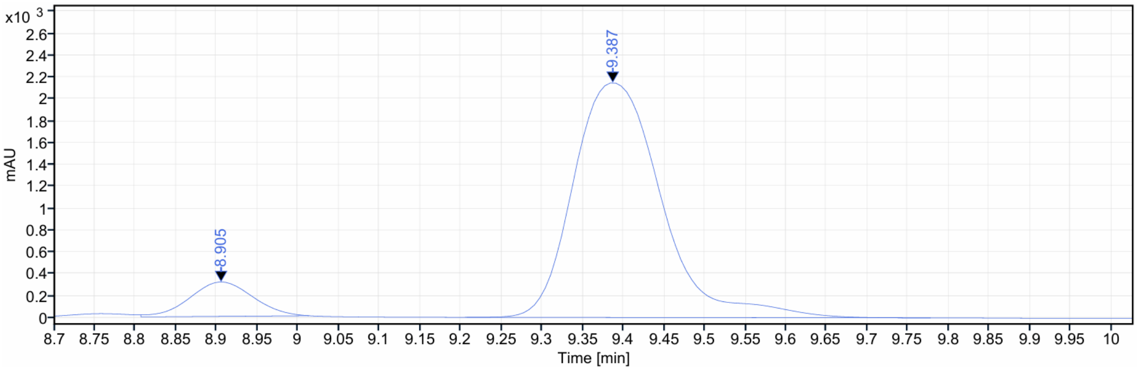

Signal: DAD1C,Sig=210,4 Ref=off

RT [min]	Type	Width [min]	Area	Height	Area%	Name
8.905	VM m	0.08	1696.28	315.66	9.51	
9.387	MM m	0.12	16140.34	2149.16	90.49	

Back to contents

Chiral HPLC (Chiralpak® IC-3, Hexane/^{i}PrOH = 93:07, 0.7 mL/min) of **2s**

See procedure

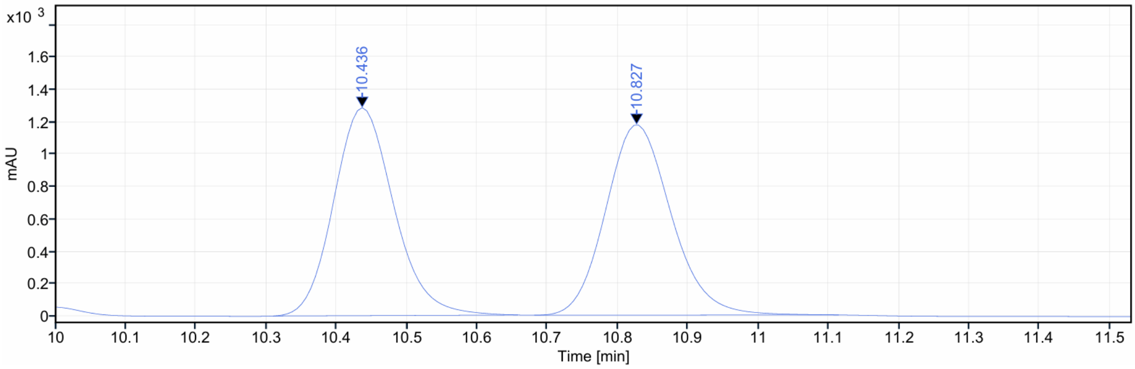

Signal: DAD1A,Sig=254,4 Ref=off

RT [min]	Type	Width [min]	Area	Height	Area%	Name
10.436	MM m	0.09	7657.08	1287.74	50.00	
10.827	MM m	0.10	7658.22	1179.58	50.00	

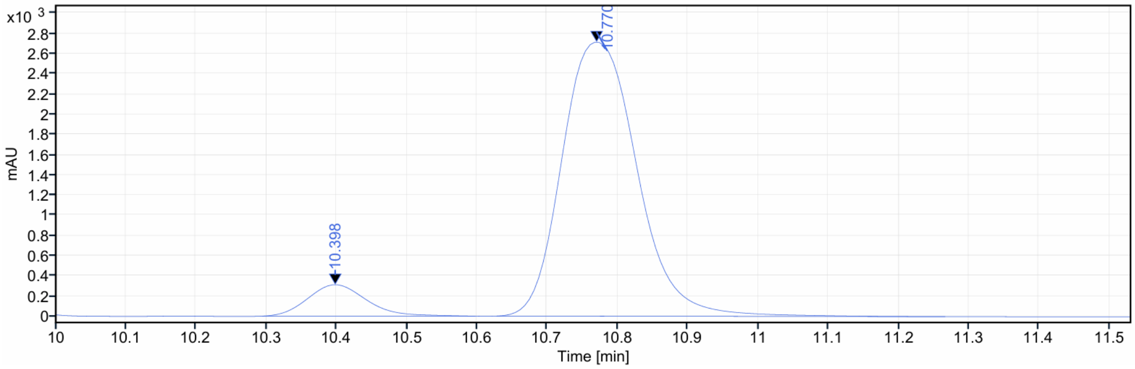

Signal: DAD1A,Sig=254,4 Ref=off

RT [min]	Type	Width [min]	Area	Height	Area%	Name
10.398	MM m	0.09	1805.16	312.01	8.22	
10.770	MM m	0.12	20167.55	2714.90	91.78	

Back to contents

Chiral HPLC (Chiralpak® IC-3, Hexane/^{i}PrOH = 93:07, 0.7 mL/min) of **2t**

See procedure

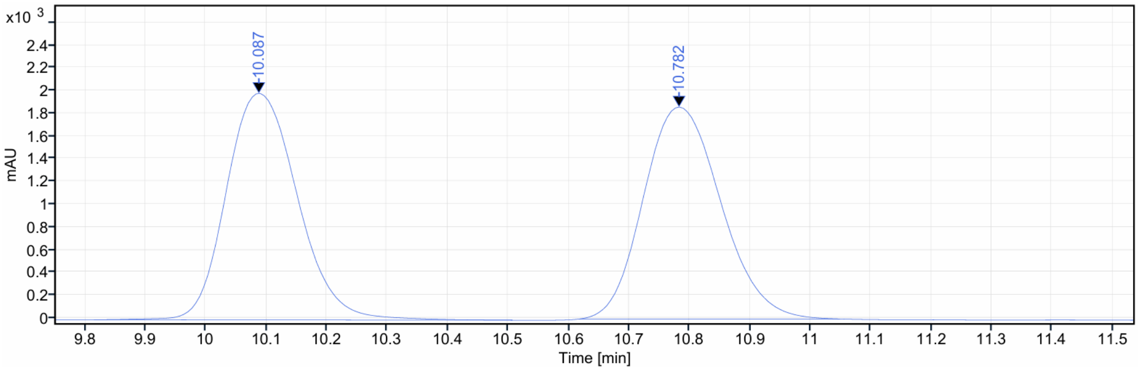

Signal: DAD1C,Sig=210,4 Ref=off

RT [min]	Type	Width [min]	Area	Height	Area%	Name
10.087	MM m	0.13	16332.55	1994.72	49.55	
10.782	MM m	0.14	16629.07	1865.97	50.45	

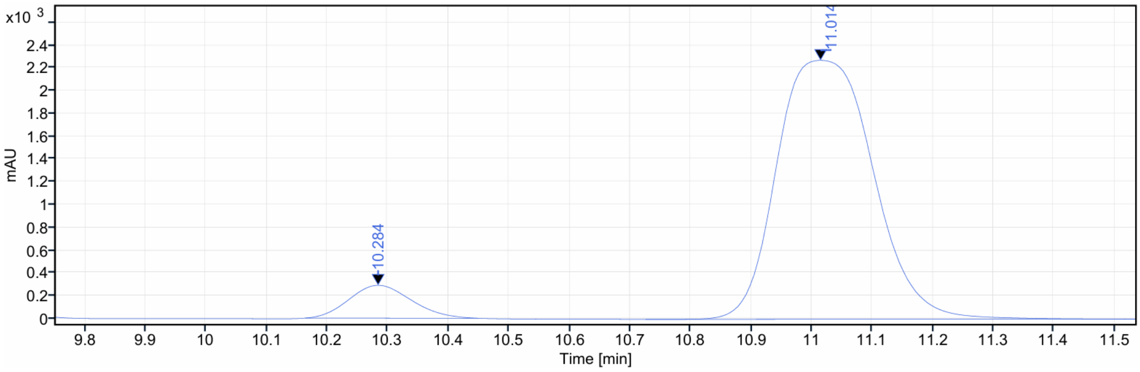

Signal: DAD1C,Sig=210,4 Ref=off

RT [min]	Type	Width [min]	Area	Height	Area%	Name
10.284	MM m	0.11	2063.88	289.54	7.60	
11.014	MB m	0.18	25085.27	2273.82	92.40	

Back to contents

Chiral HPLC (Chiralpak® IC-3, Hexane/^{i}PrOH = 93:07, 0.7 mL/min) of **2u**

See procedure

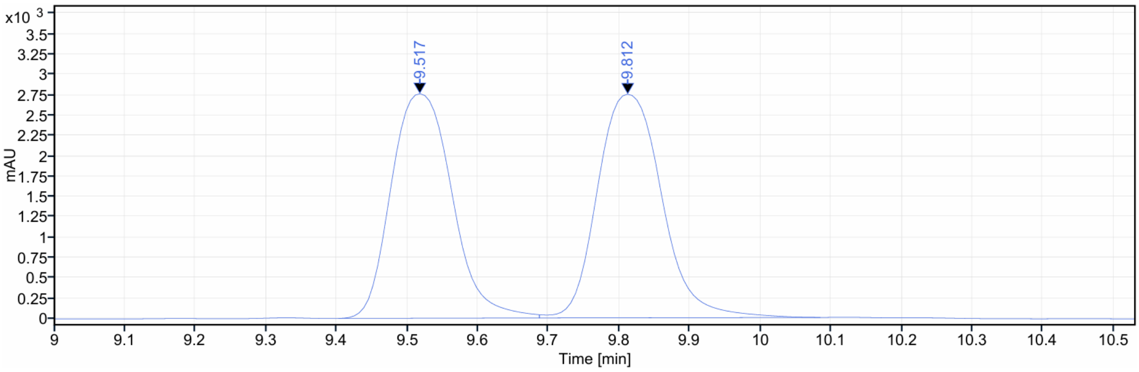

Signal: DAD1A,Sig=254,4 Ref=off

RT [min]	Type	Width [min]	Area	Height	Area%	Name
9.517	MM m	0.10	16530.79	2761.64	48.74	
9.812	MM m	0.10	17386.09	2749.83	51.26	

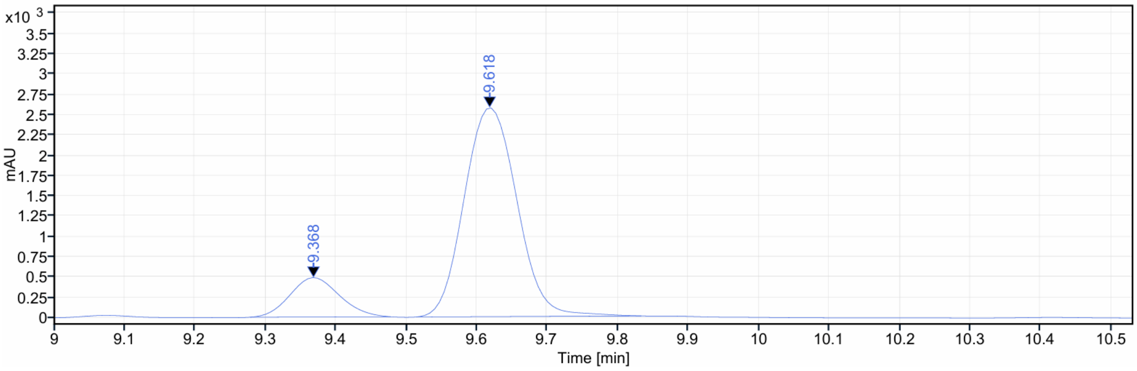

Signal: DAD1A,Sig=254,4 Ref=off

RT [min]	Type	Width [min]	Area	Height	Area%	Name
9.368	MM m	0.07	2355.30	485.64	14.99	
9.618	MM m	0.08	13352.34	2575.35	85.01	

Back to contents

Chiral HPLC (Chiralpak® IC-3, Hexane/^{i}PrOH = 93:07, 0.7 mL/min) of **2v**

See procedure

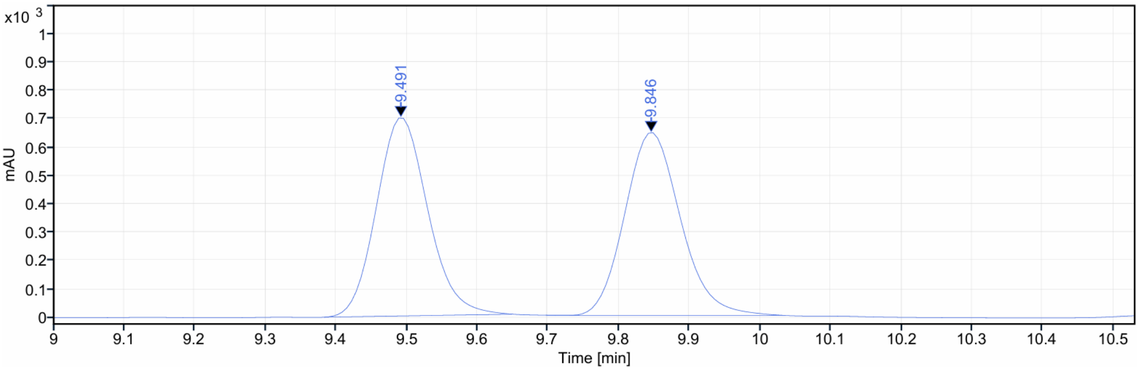

Signal: DAD1A,Sig=254,4 Ref=off

RT [min]	Type	Width [min]	Area	Height	Area%	Name
9.491	MM m	0.08	3529.92	700.95	50.00	
9.846	MM m	0.08	3530.43	645.75	50.00	

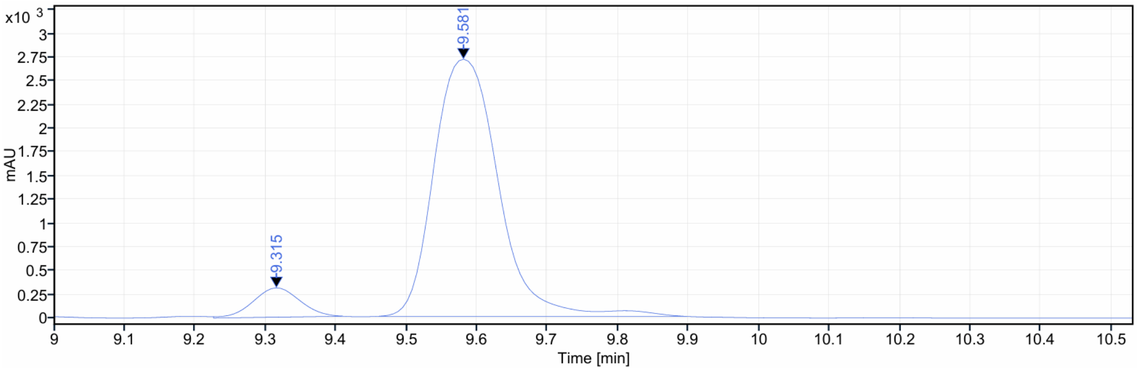

Signal: DAD1A,Sig=254,4 Ref=off

RT [min]	Type	Width [min]	Area	Height	Area%	Name
9.315	VM m	0.07	1425.82	309.89	7.62	
9.581	MM m	0.10	17280.74	2715.00	92.38	

Back to contents

Chiral HPLC (Chiralpak® IC-3, Hexane/^{i}PrOH = 97:03, 0.7 mL/min) of **2w**

See procedure

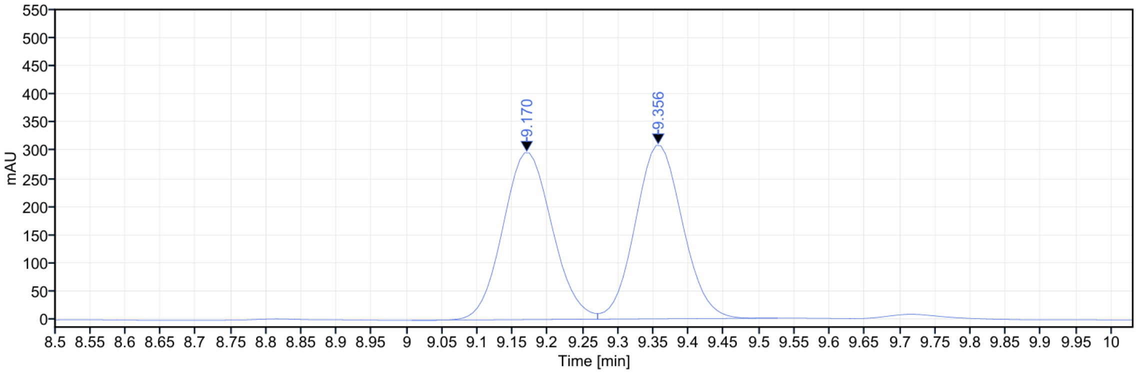

Signal: DAD1A,Sig=254,4 Ref=off

RT [min]	Type	Width [min]	Area	Height	Area%	Name
9.170	BM m	0.08	1440.78	297.01	50.09	
9.356	MM m	0.07	1435.79	308.68	49.91	

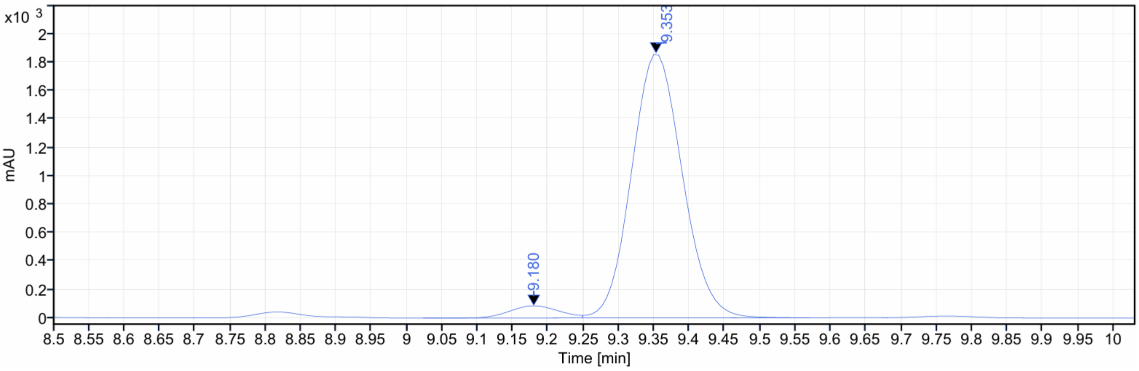

Signal: DAD1A,Sig=254,4 Ref=off

RT [min]	Type	Width [min]	Area	Height	Area%	Name
9.180	BV	0.23	415.02	86.13	4.28	
9.353	VB	0.32	9286.83	1862.26	95.72	

Back to contents

Chiral HPLC (Chiralpak® IC-3, Hexane/^{i}PrOH = 99:01, 0.8 mL/min) of **2x**

See procedure

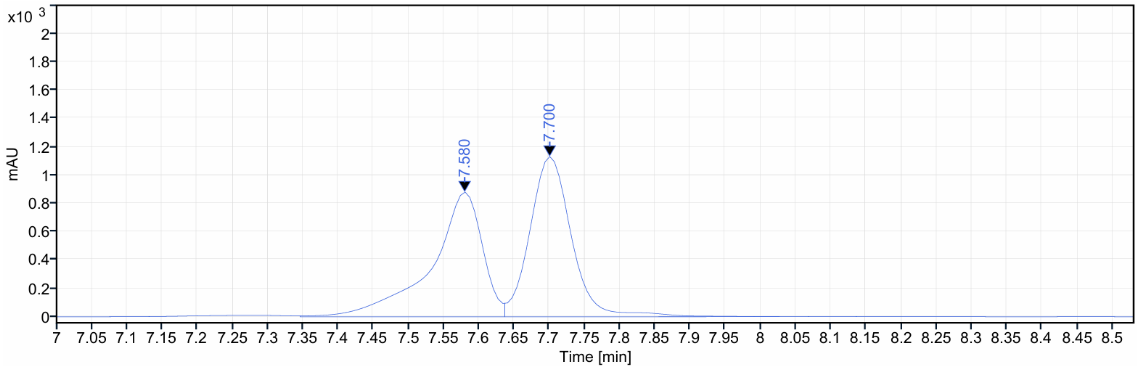

Signal: DAD1A,Sig=250,4 Ref=off

RT [min]	Type	Width [min]	Area	Height	Area%	Name
7.580	VV	0.29	4423.75	880.85	48.74	
7.700	VB	0.39	4651.93	1131.16	51.26	

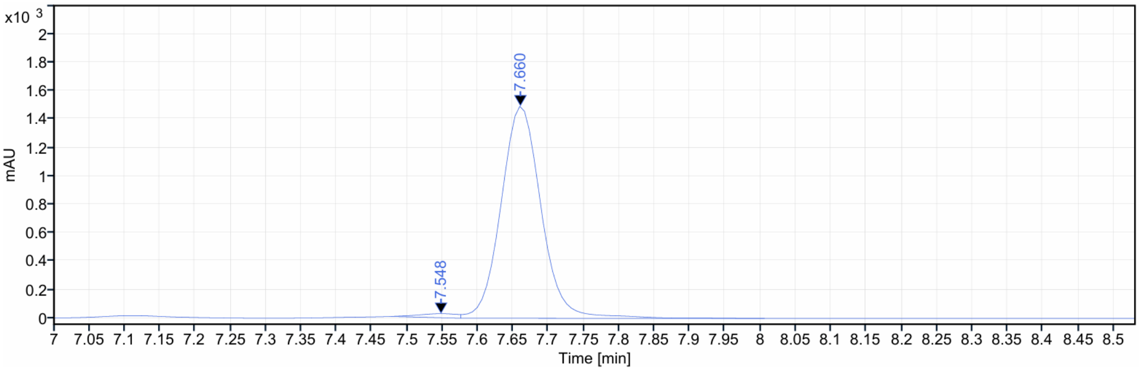

Signal: DAD1A,Sig=250,4 Ref=off

RT [min]	Type	Width [min]	Area	Height	Area%	Name
7.548	MM m	0.06	107.69	29.12	1.79	
7.660	VB	0.43	5901.50	1492.70	98.21	

Back to contents

Chiral HPLC (Chiralpak® IC-3, Hexane, 1.8 mL/min) of **2y**

See procedure

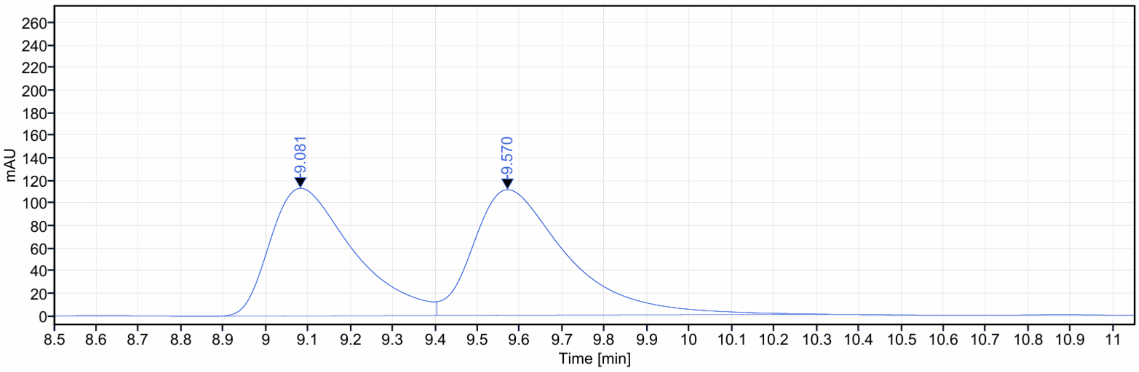

Signal: DAD1A,Sig=250,4 Ref=off

RT [min]	Type	Width [min]	Area	Height	Area%	Name
9.081	MM m	0.20	1545.76	113.09	47.47	
9.570	MM m	0.21	1710.21	111.25	52.53	

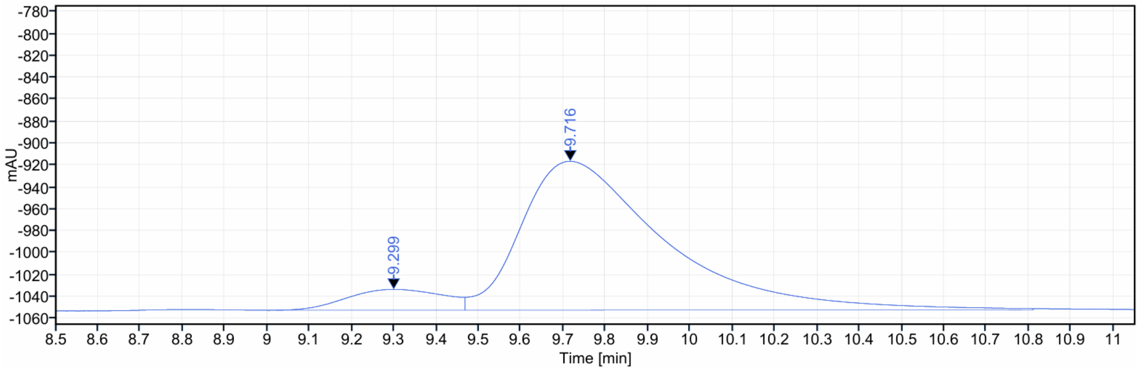

Signal: DAD1A,Sig=250,4 Ref=off

RT [min]	Type	Width [min]	Area	Height	Area%	Name
9.299	BV	0.45	301.73	18.97	8.61	
9.716	VV	1.34	3202.62	136.10	91.39	

Back to contents

Chiral HPLC (Chiralpak® IC-3, Hexane/^{i}PrOH = 93:07, 0.7 mL/min) of **2z**

See procedure

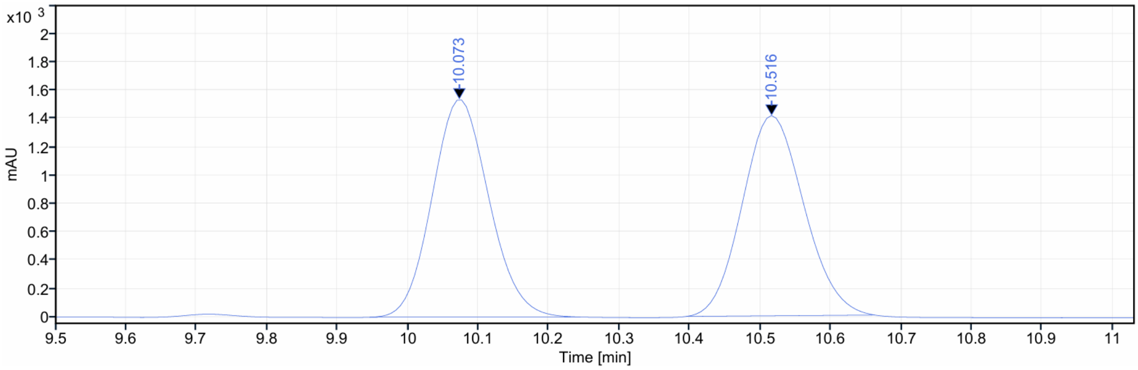

Signal:	DAD1A,Sig=250,4 Ref=off					
RT [min]	Type	Width [min]	Area	Height	Area%	Name
10.073	MM m	0.09	8630.19	1537.43	50.00	
10.516	MM m	0.10	8630.97	1413.12	50.00	

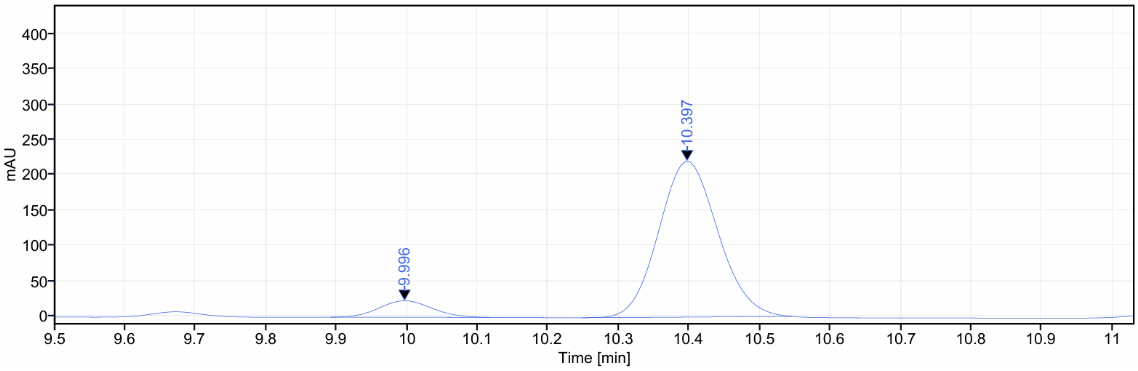

Signal:	DAD1A,Sig=250,4 Ref=off					
RT [min]	Type	Width [min]	Area	Height	Area%	Name
9.996	MM m	0.08	121.37	23.45	8.62	
10.397	MM m	0.09	1286.35	220.69	91.38	

Back to contents

Chiral HPLC (Chiralpak® IC-3, Hexane/^{i}PrOH = 90:10, 0.4 mL/min) of **2aa**

See procedure

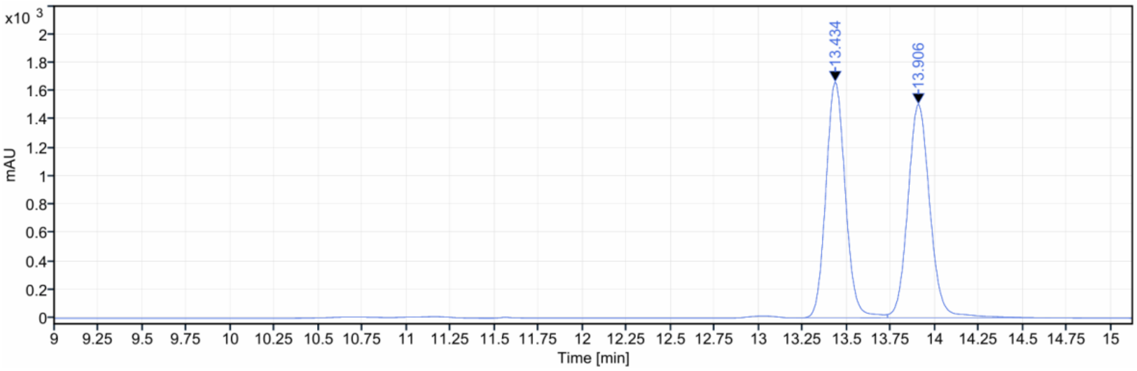

Signal: DAD1A,Sig=254,4 Ref=off

RT [min]	Type	Width [min]	Area	Height	Area%	Name
13.434	MM m	0.12	12762.66	1663.72	49.38	
13.906	MM m	0.14	13082.30	1505.79	50.62	

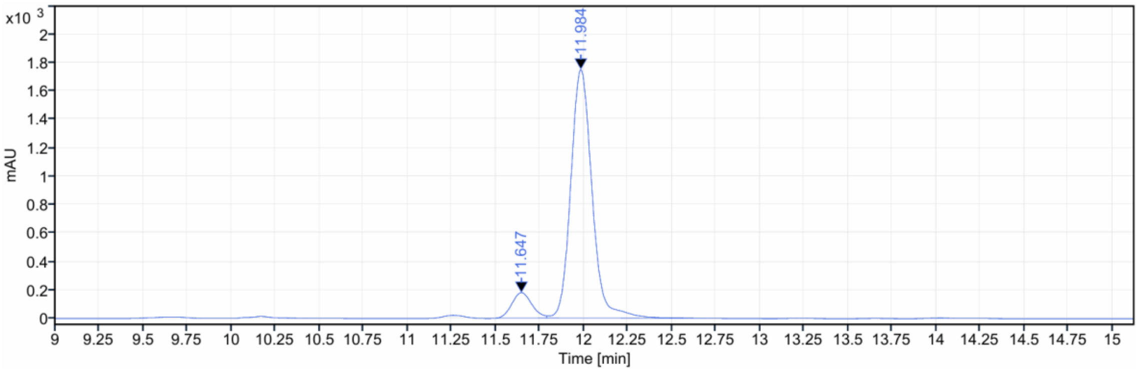

Signal: DAD1A,Sig=254,4 Ref=off

RT [min]	Type	Width [min]	Area	Height	Area%	Name
11.647	MM m	0.12	1402.95	179.20	8.71	
11.984	MM m	0.13	14697.50	1749.87	91.29	

Back to contents

Chiral HPLC (Chiralpak® IC-3, Hexane/^{i}PrOH = 93:07, 0.7 mL/min) of **2ab**

See procedure

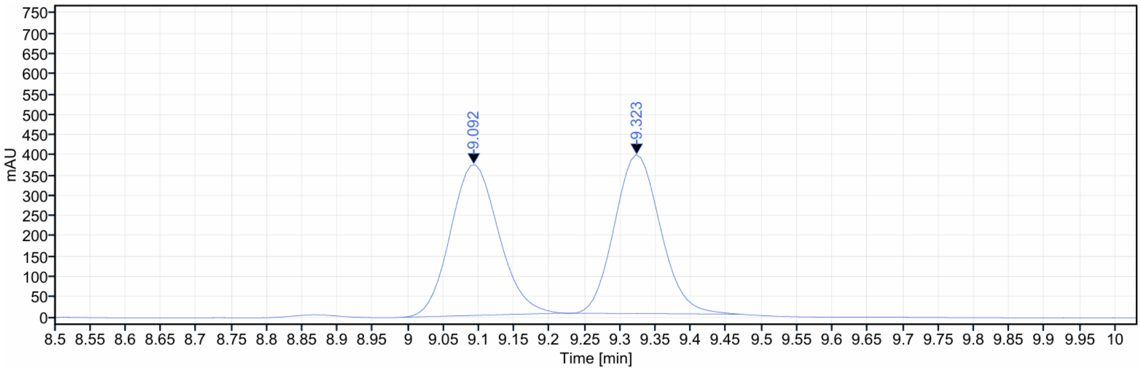

Signal: DAD1A,Sig=254,4 Ref=off

RT [min]	Type	Width [min]	Area	Height	Area%	Name
9.092	MM m	0.08	1767.86	372.95	49.99	
9.323	MM m	0.07	1768.71	392.25	50.01	

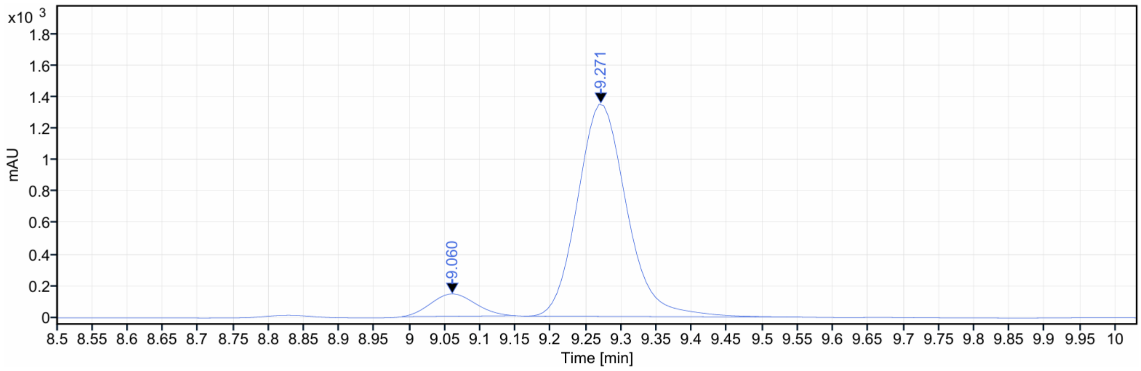

Signal: DAD1A,Sig=254,4 Ref=off

RT [min]	Type	Width [min]	Area	Height	Area%	Name
9.060	MM m	0.07	625.09	142.49	8.73	
9.271	MM m	0.07	6537.37	1350.19	91.27	

Back to contents

Chiral HPLC (Chiralpak® IC-3, Hexane/^{i}PrOH = 93:07, 0.7 mL/min) of **2ac**

See procedure

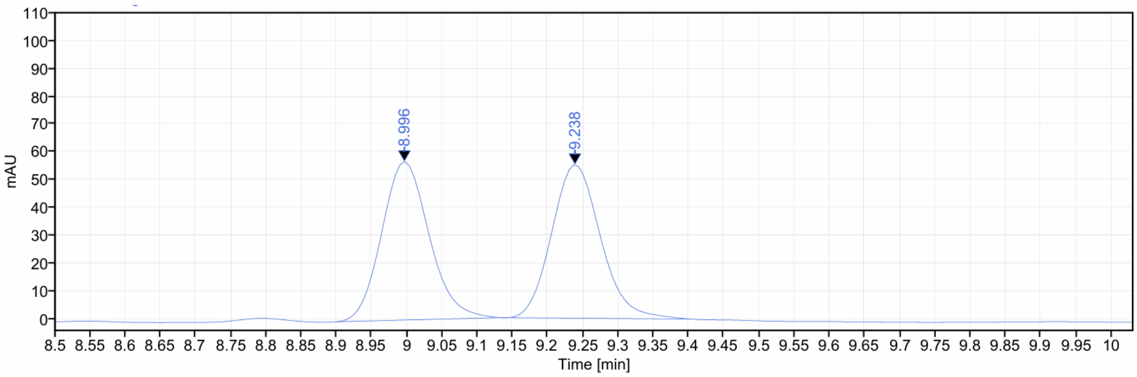

Signal: DAD1A,Sig=254,4 Ref=off

RT [min]	Type	Width [min]	Area	Height	Area%	Name
8.996	MM m	0.07	265.82	57.02	50.00	
9.238	MM m	0.07	265.82	55.28	50.00	

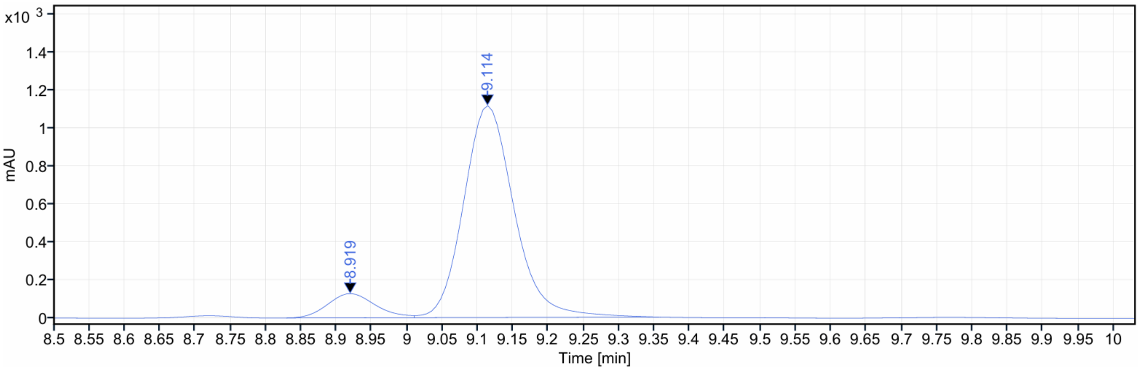

Signal: DAD1A,Sig=254,4 Ref=off

RT [min]	Type	Width [min]	Area	Height	Area%	Name
8.919	MM m	0.07	597.83	128.28	9.74	
9.114	MM m	0.08	5542.39	1119.24	90.26	

Back to contents

Chiral HPLC (Chiralpak® IC-3, Hexane/^{i}PrOH = 95:05, 0.5 mL/min) of **2ad**

See procedure

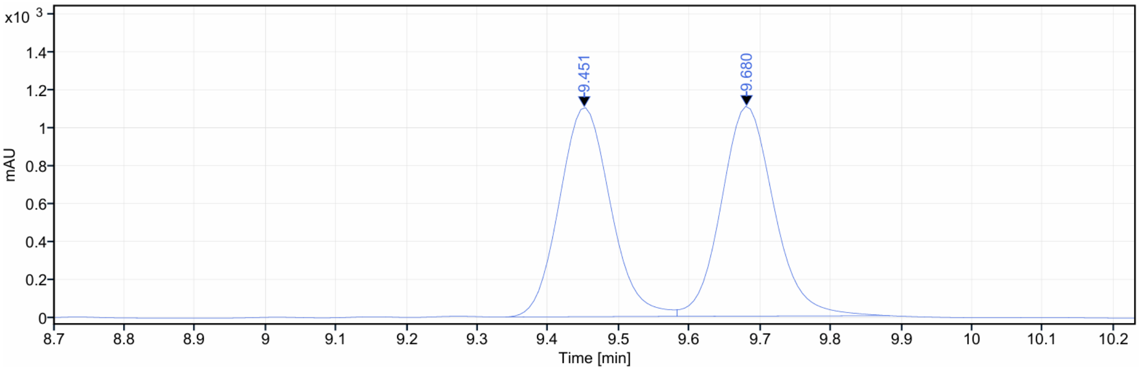

Signal: DAD1A,Sig=254,4 Ref=off

RT [min]	Type	Width [min]	Area	Height	Area%	Name
9.451	MM m	0.08	5567.97	1106.30	49.24	
9.680	MM m	0.08	5739.32	1109.15	50.76	

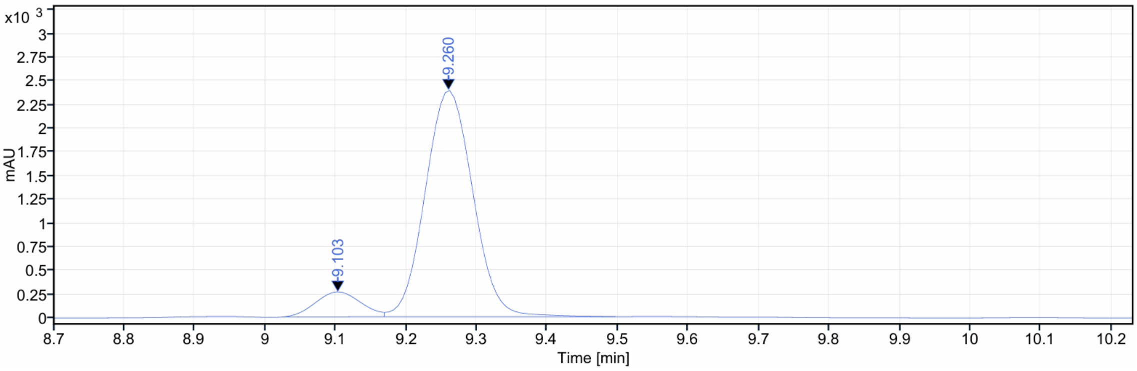

Signal: DAD1A,Sig=254,4 Ref=off

RT [min]	Type	Width [min]	Area	Height	Area%	Name
9.103	MM m	0.07	1167.46	263.55	9.43	
9.260	MM m	0.07	11214.73	2394.26	90.57	

[Back to contents](#)

Chiral HPLC (Chiralpak® IC-3, Hexane/^{i}PrOH = 99:01, 0.6 mL/min) of **2ae**

See procedure

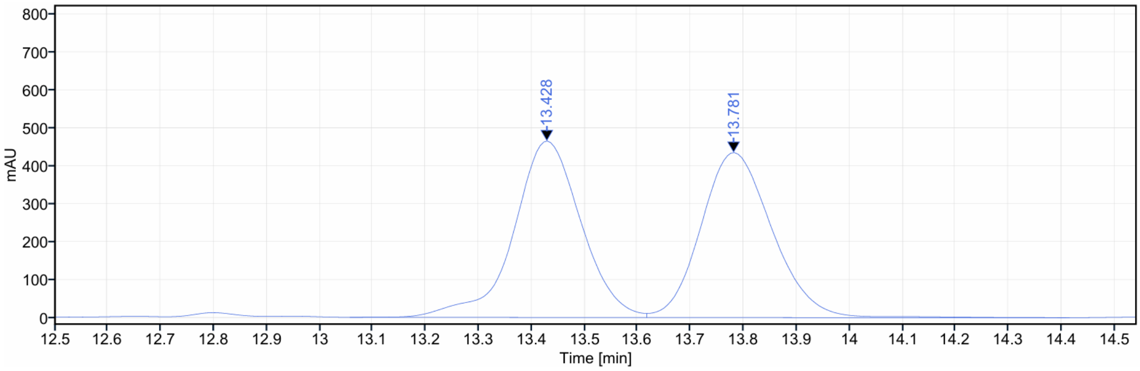

Signal: DAD1A,Sig=250,4 Ref=off

RT [min]	Type	Width [min]	Area	Height	Area%	Name
13.428	BV	0.56	4136.07	466.57	50.35	
13.781	VV	0.80	4078.12	436.22	49.65	

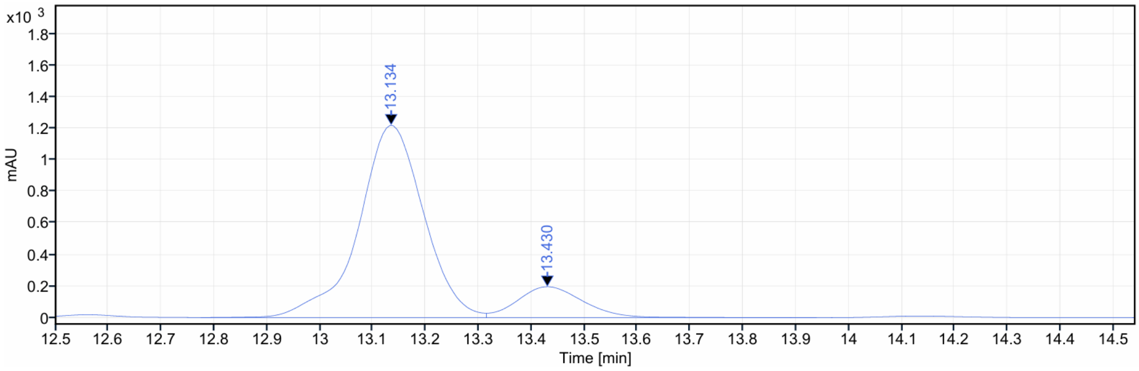

Signal: DAD1A,Sig=250,4 Ref=off

RT [min]	Type	Width [min]	Area	Height	Area%	Name
13.134	VV	0.54	10458.81	1220.95	85.97	
13.430	VB	0.62	1707.53	196.83	14.03	

Back to contents

Chiral HPLC (Chiralpak® IC-3, Hexane/^{i}PrOH = 99:01, 0.9 mL/min) of **2af**
See procedure

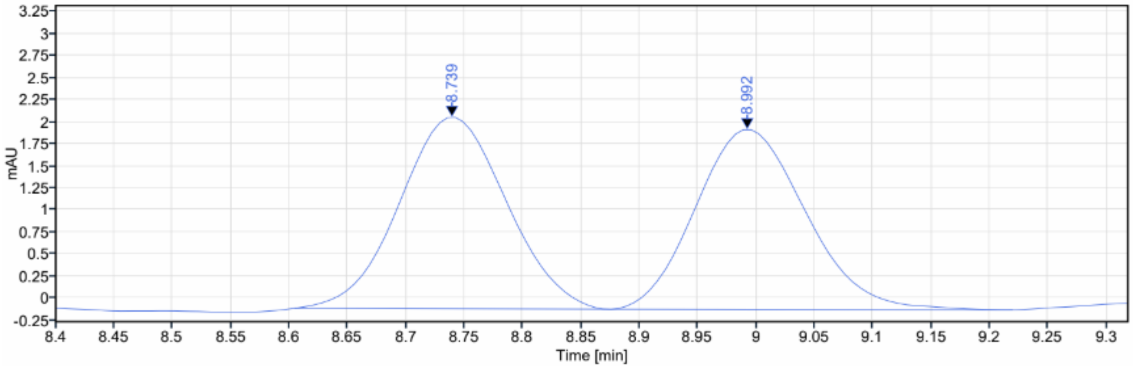

Signal: DAD1A,Sig=250,4 Ref=off

RT [min]	Type	Width [min]	Area	Height	Area%	Name
8.739	MM m	0.10	13.82	2.18	50.85	
8.992	MM m	0.10	13.36	2.04	49.15	

***1af** as starting material*

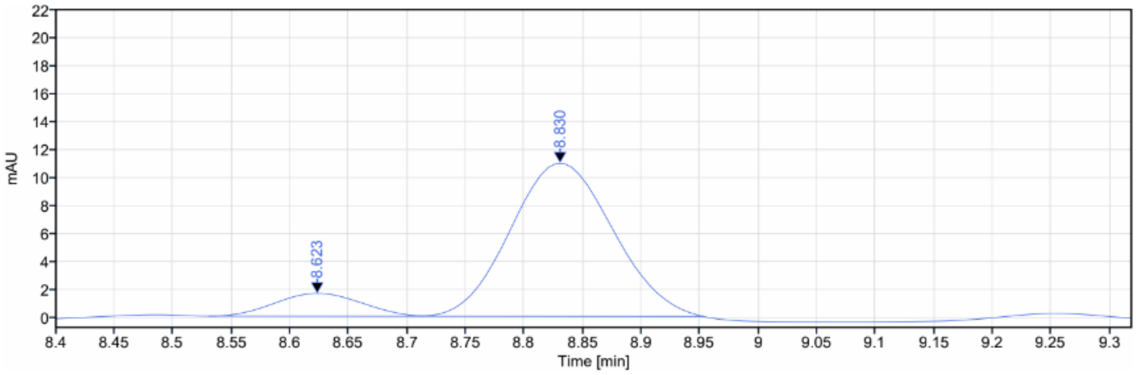

Signal: DAD1A,Sig=250,4 Ref=off

RT [min]	Type	Width [min]	Area	Height	Area%	Name
8.623	MM m	0.08	8.31	1.63	11.12	
8.830	MM m	0.09	66.40	10.93	88.88	

*(Z)-**1af** as starting material*

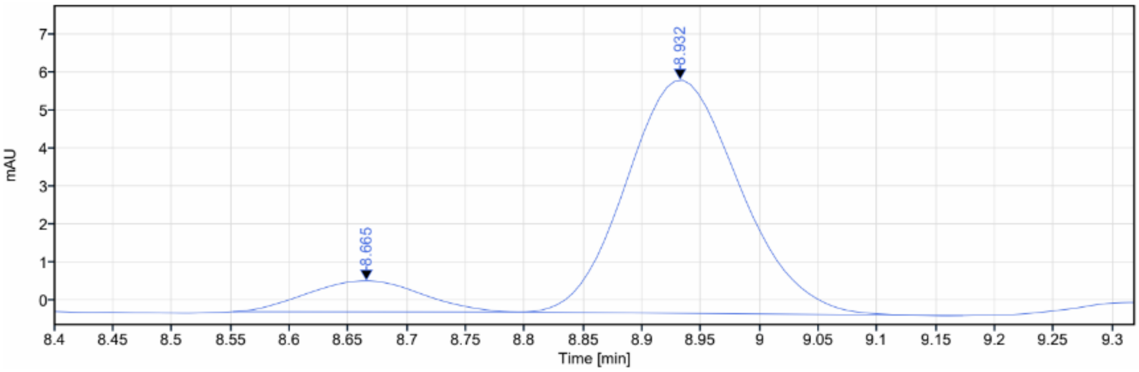

Signal: DAD1A,Sig=250,4 Ref=off

RT [min]	Type	Width [min]	Area	Height	Area%	Name
8.665	MM m	0.11	5.54	0.82	11.82	
8.932	MM m	0.10	41.33	6.16	88.18	

Back to contents

Chiral HPLC (Chiralpak® IC-3, Hexane/^{i}PrOH = 93:07, 0.7 mL/min) of **2ag**

See procedure

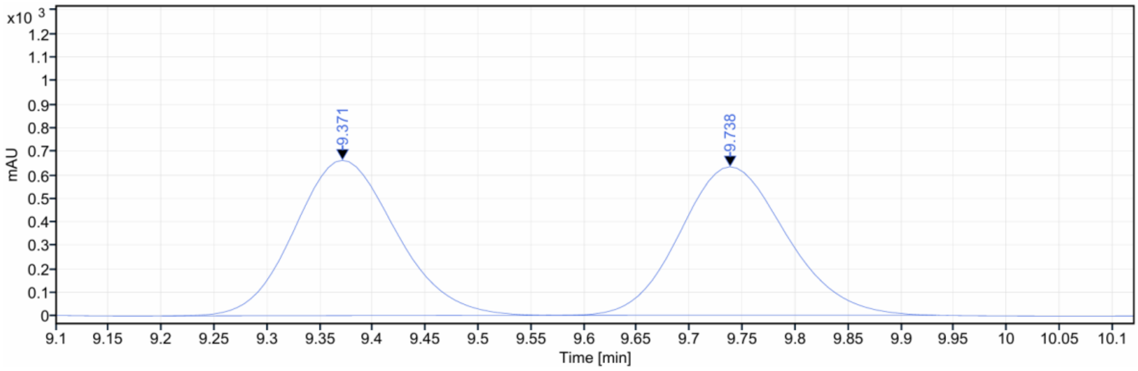

Signal:	DAD1A,Sig=250,4 Ref=off					
RT [min]	Type	Width [min]	Area	Height	Area%	Name
9.371	MM m	0.10	4429.21	660.53	49.98	
9.738	MM m	0.11	4433.63	631.21	50.02	

***1ag** as starting material*

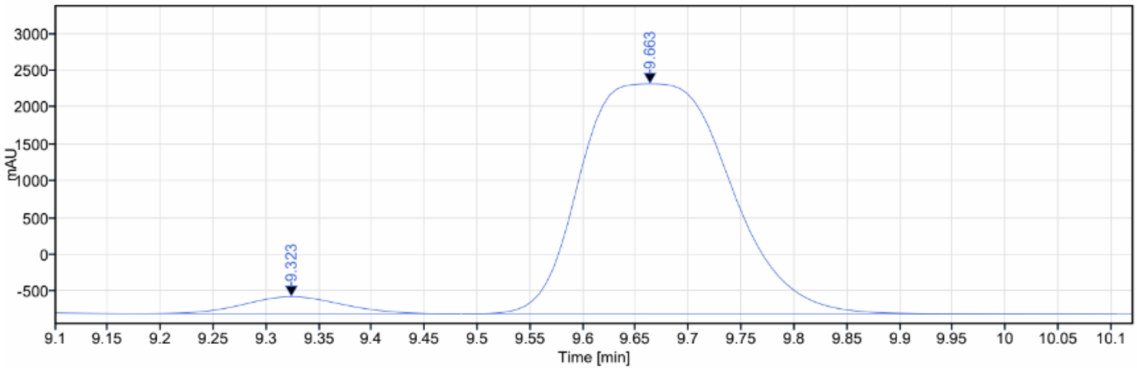

Signal:	DAD1A,Sig=250,4 Ref=off					
RT [min]	Type	Width [min]	Area	Height	Area%	Name
9.323	BB	0.31	1488.99	230.13	4.70	
9.663	BB	0.62	30166.10	3124.26	95.30	

*(Z)-**1ag** as starting material*

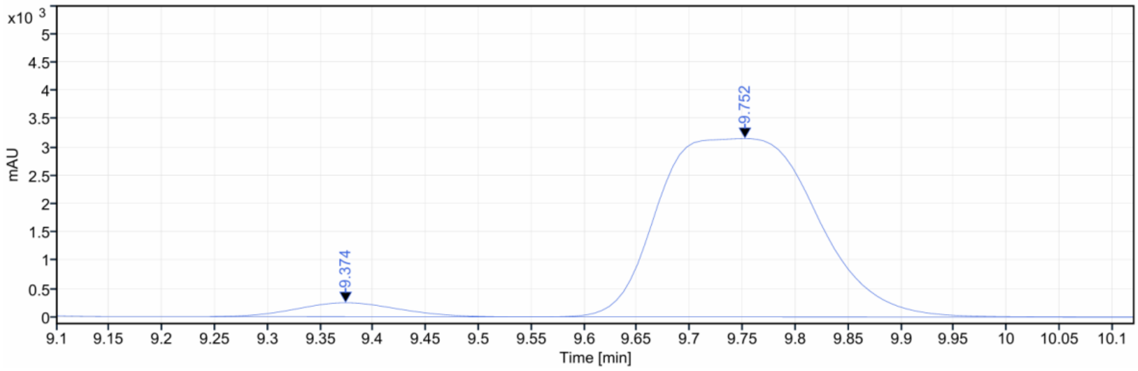

Signal: DAD1A,Sig=250,4 Ref=off

RT [min]	Type	Width [min]	Area	Height	Area%	Name
9.374	MM m	0.11	1663.68	246.60	4.90	
9.752	MB m	0.16	32280.99	3149.15	95.10	

Back to contents

Chiral HPLC (Chiralpak® OJ-3, Hexane/^{i}PrOH = 93:07, 0.9 mL/min) of **2ah**
See procedure

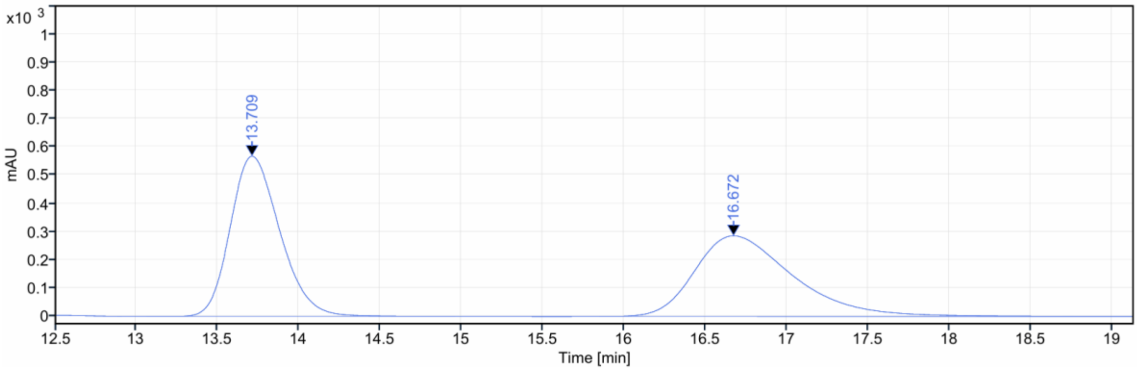

Signal: DAD1A,Sig=250,4 Ref=off

RT [min]	Type	Width [min]	Area	Height	Area%	Name
13.709	BB	1.90	12024.39	567.73	50.00	
16.672	BM m	0.64	12023.68	285.68	50.00	

***1ah** as starting material*

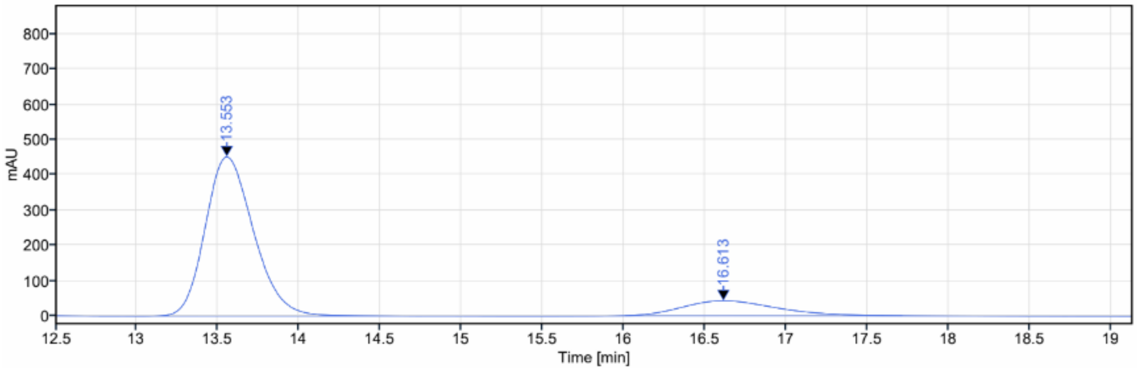

Signal: DAD1A,Sig=250,4 Ref=off

RT [min]	Type	Width [min]	Area	Height	Area%	Name
13.553	MM m	0.32	9375.95	452.04	84.59	
16.613	MM m	0.62	1708.16	42.55	15.41	

*(Z)-**1ah** as starting material*

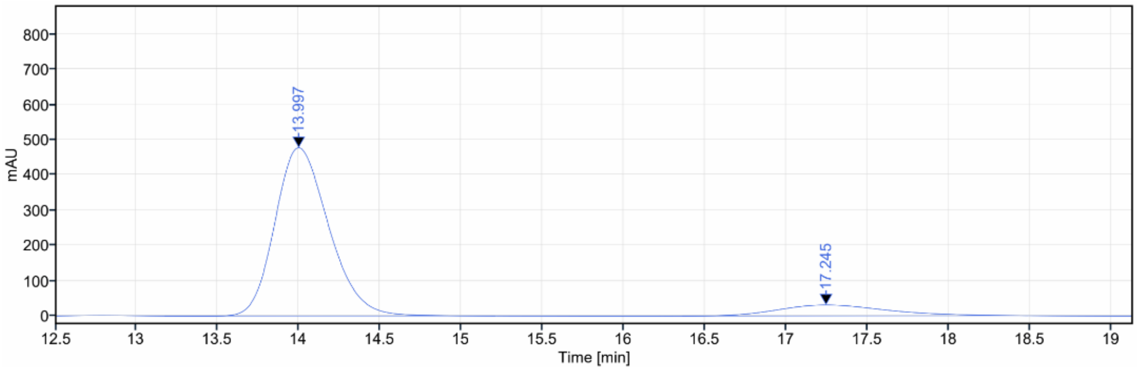

Signal: DAD1A,Sig=250,4 Ref=off

RT [min]	Type	Width [min]	Area	Height	Area%	Name
13.997	MM m	0.35	11000.91	477.98	88.38	
17.245	MM m	0.70	1445.90	30.98	11.62	

Back to contents

Chiral HPLC (Chiralpak® IC-3, Hexane/^{i}PrOH = 99:01, 0.2 mL/min) of **2ai**

See procedure

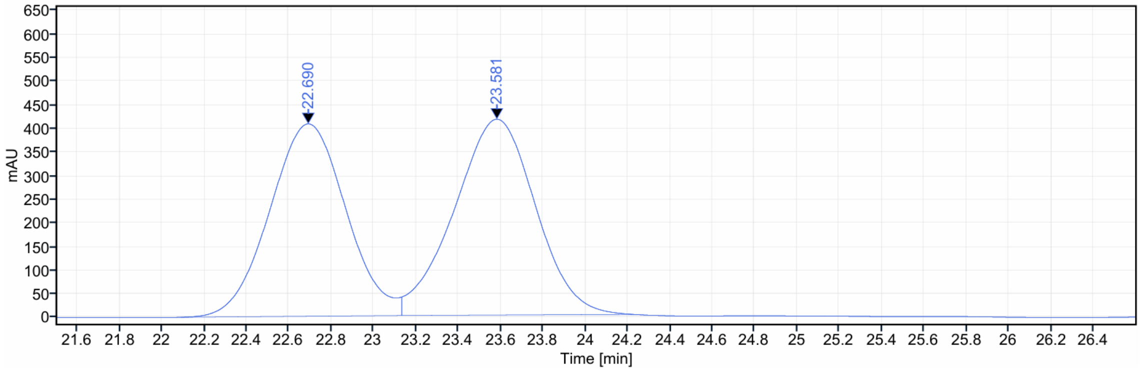

Signal: DAD1A,Sig=250,4 Ref=off

RT [min]	Type	Width [min]	Area	Height	Area%	Name
22.690	MM m	0.39	10187.00	407.15	47.96	
23.581	MM m	0.41	11055.25	414.47	52.04	

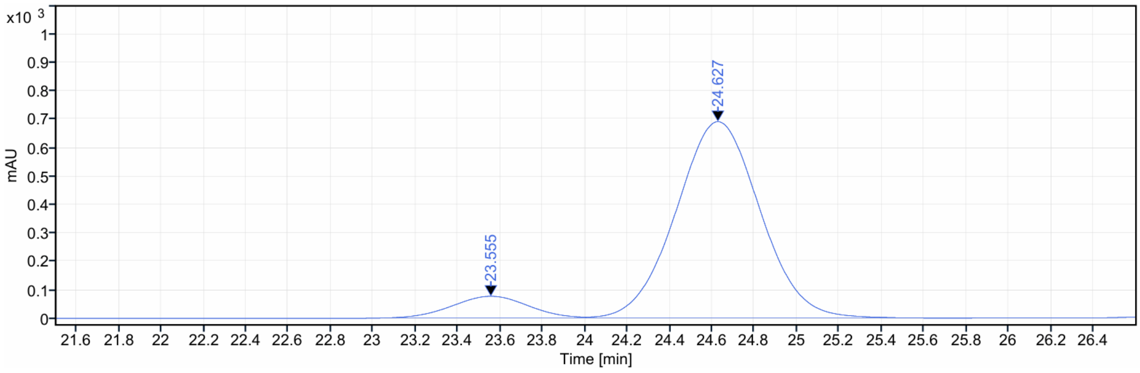

Signal: DAD1A,Sig=250,4 Ref=off

RT [min]	Type	Width [min]	Area	Height	Area%	Name
23.555	MM m	0.39	1894.18	76.19	9.04	
24.627	MM m	0.43	19048.27	690.58	90.96	

Back to contents

Chiral HPLC (Chiralpak® IC-3, Hexane/^{i}PrOH = 99:01, 0.5 mL/min) of **2aj**

See procedure

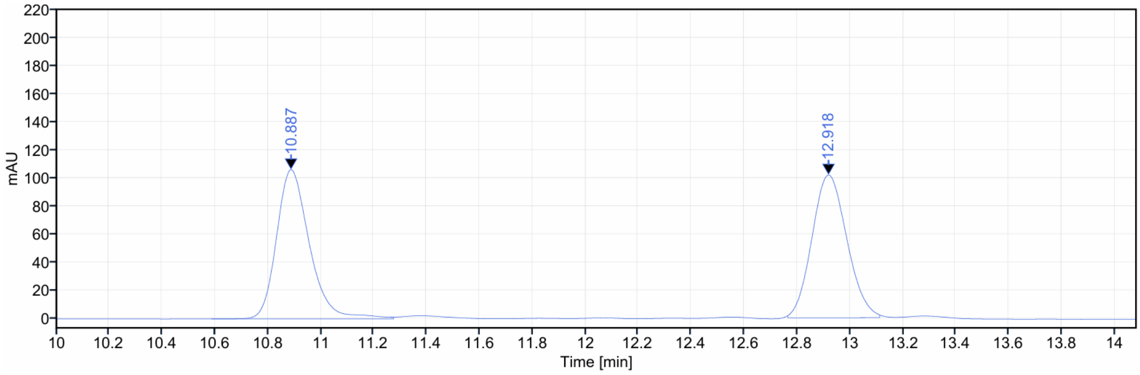

Signal: DAD1A,Sig=250,4 Ref=off

RT [min]	Type	Width [min]	Area	Height	Area%	Name
10.887	MM m	0.13	917.92	106.13	49.71	
12.918	MM m	0.14	928.60	101.55	50.29	

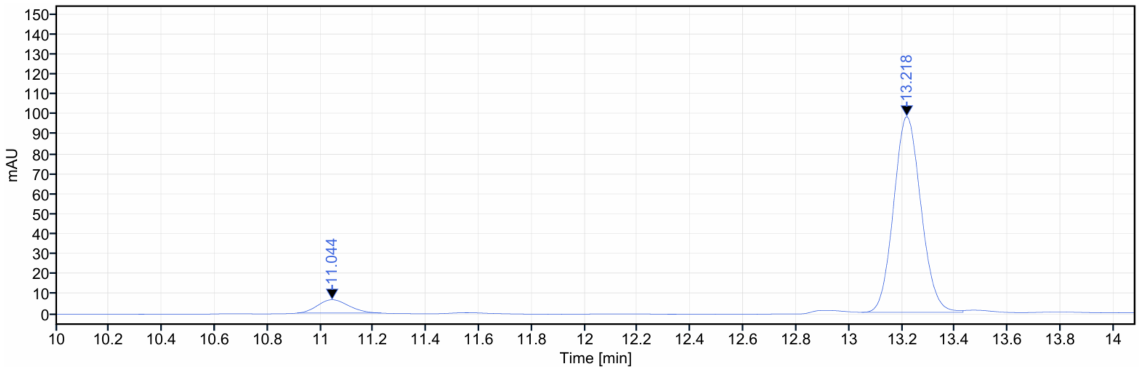

Signal: DAD1A,Sig=250,4 Ref=off

RT [min]	Type	Width [min]	Area	Height	Area%	Name
11.044	MM m	0.13	54.73	6.69	7.23	
13.218	MM m	0.11	701.95	97.93	92.77	

Back to contents

Chiral HPLC (Chiralpak® IA-3, Hexane/^{i}PrOH = 95:05, 0.5 mL/min) of **2ak**

See procedure

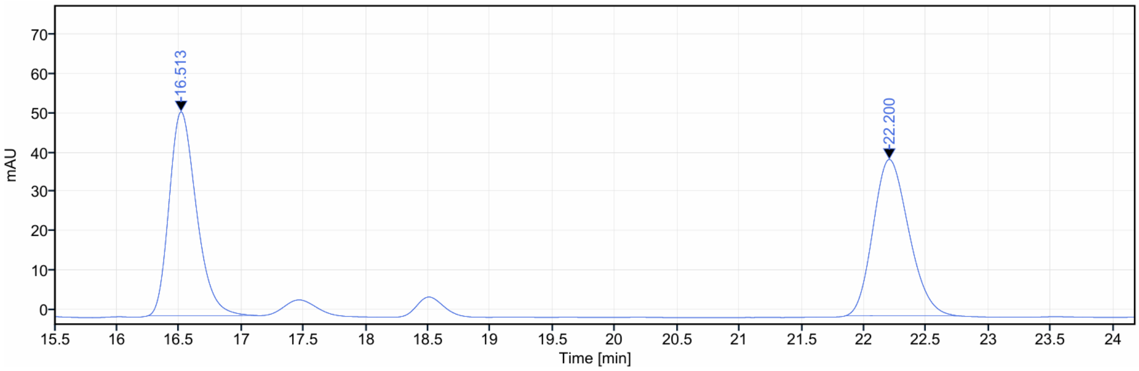

Signal: DAD1A,Sig=250,4 Ref=off

RT [min]	Type	Width [min]	Area	Height	Area%	Name
16.513	MM m	0.23	783.92	51.63	49.87	
22.200	MM m	0.31	788.04	39.59	50.13	

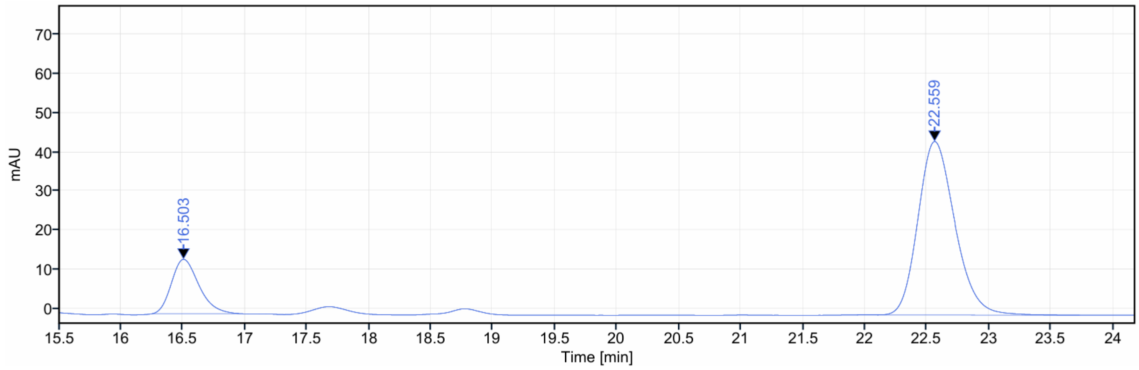

Signal: DAD1A,Sig=250,4 Ref=off

RT [min]	Type	Width [min]	Area	Height	Area%	Name
16.503	MM m	0.23	212.07	13.82	18.79	
22.559	MB m	0.32	916.44	44.09	81.21	

Back to contents

Chiral HPLC (Chiralpak® IC-3, Hexane/^{i}PrOH = 99:01, 0.3 mL/min) of **2al**

See procedure

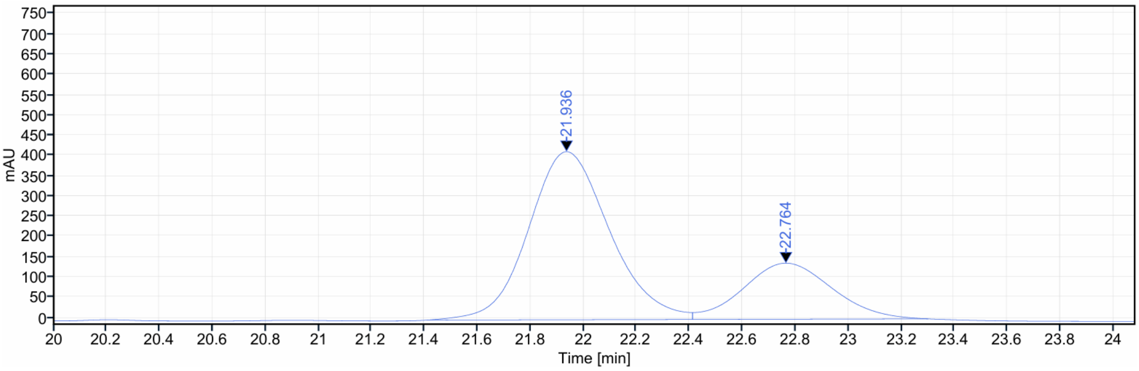

Signal: DAD1A,Sig=250,4 Ref=off

RT [min]	Type	Width [min]	Area	Height	Area%	Name
21.936	MM m	0.33	8837.12	415.20	73.20	
22.764	MM m	0.36	3235.65	138.43	26.80	

Chiral HPLC (Chiralpak® IC-3, Hexane/^{i}PrOH = 99:01, 0.3 mL/min) of (*R*)-**2al**

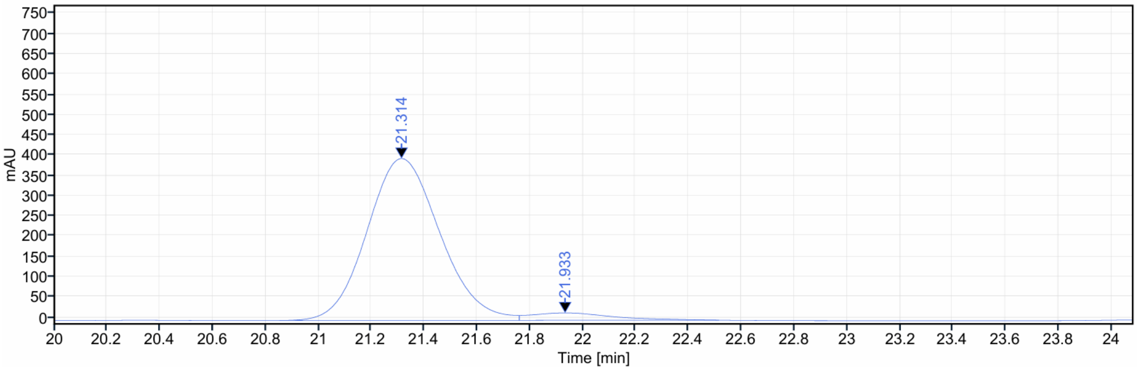

Signal: DAD1A,Sig=250,4 Ref=off

RT [min]	Type	Width [min]	Area	Height	Area%	Name
21.314	MM m	0.30	7666.08	399.87	95.13	
21.933	MM m	0.32	392.09	18.19	4.87	

Chiral HPLC (Chiralpak® IC-3, Hexane/^{i}PrOH = 99:01, 0.3 mL/min) of (*S*)-**2al**

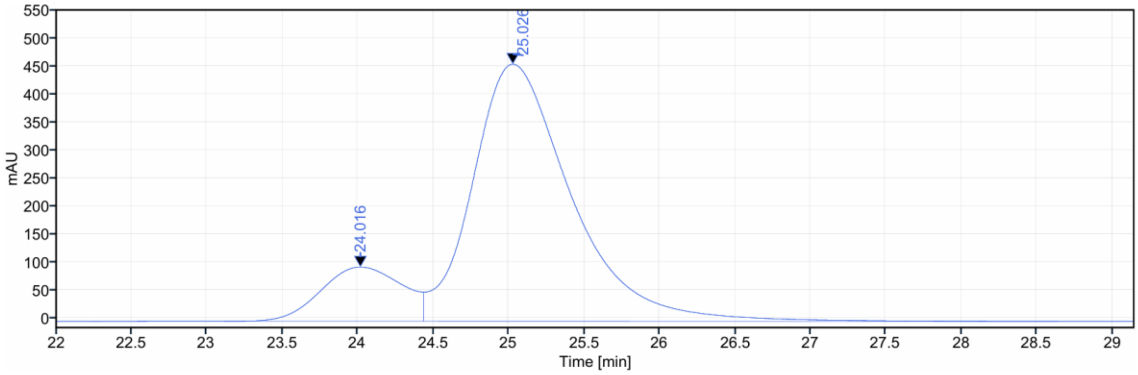

Signal: DAD1A,Sig=250,4 Ref=off

RT [min]	Type	Width [min]	Area	Height	Area%	Name
24.016	BV	1.32	3618.91	96.50	14.83	
25.026	VB	4.21	20784.43	457.82	85.17	

Back to contents

Chiral HPLC (Chiralpak® OJ-3, Hexane/^{i}PrOH = 90:10, 0.9 mL/min) of (*S*)-**3a**

See procedure

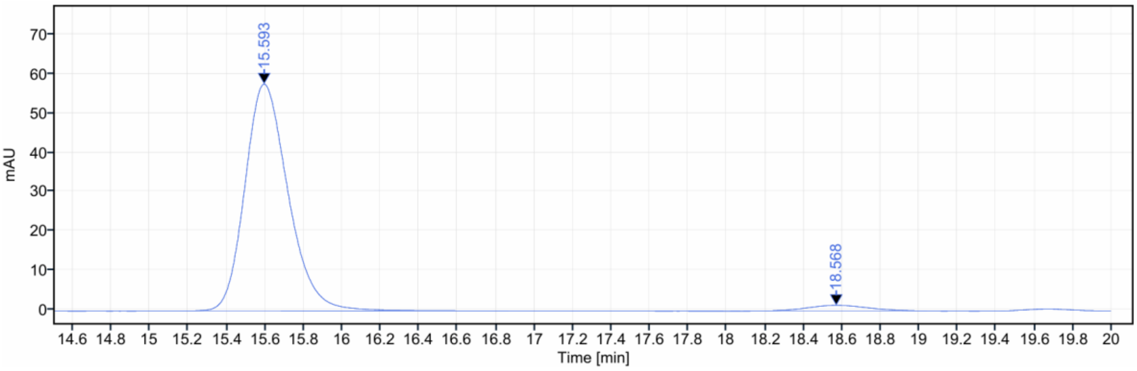

Signal: DAD1A,Sig=250,4 Ref=off

RT [min]	Type	Width [min]	Area	Height	Area%	Name
15.593	BM m	0.24	898.75	57.54	96.87	
18.568	MM m	0.32	29.08	1.37	3.13	

Chiral HPLC (Chiralpak® OJ-3, Hexane/^{i}PrOH = 90:10, 0.9 mL/min) of (*R*)-**3a**

See procedure

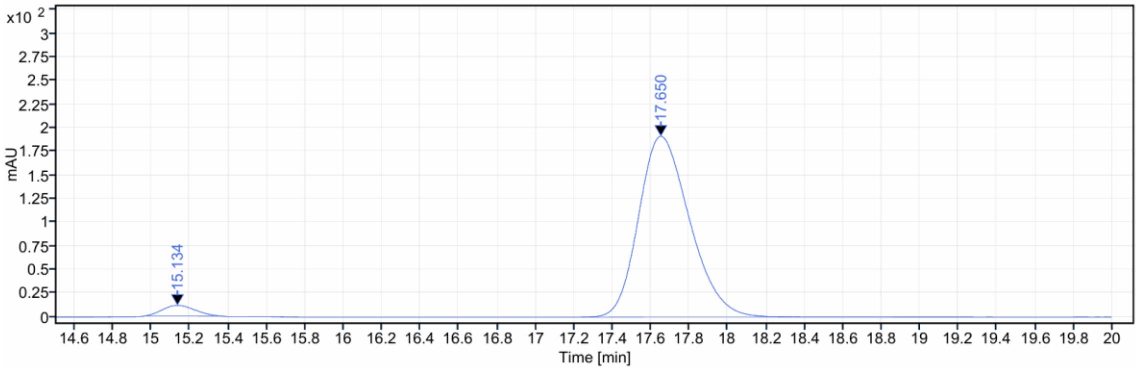

Signal: DAD1A,Sig=250,4 Ref=off

RT [min]	Type	Width [min]	Area	Height	Area%	Name
15.134	MM m	0.18	131.87	11.27	3.61	
17.650	MM m	0.28	3523.35	191.50	96.39	

Back to contents

Chiral HPLC (Chiralpak® IA-3, Hexane/^{i}PrOH = 99:01, 0.3 mL/min) of **3b**

See procedure

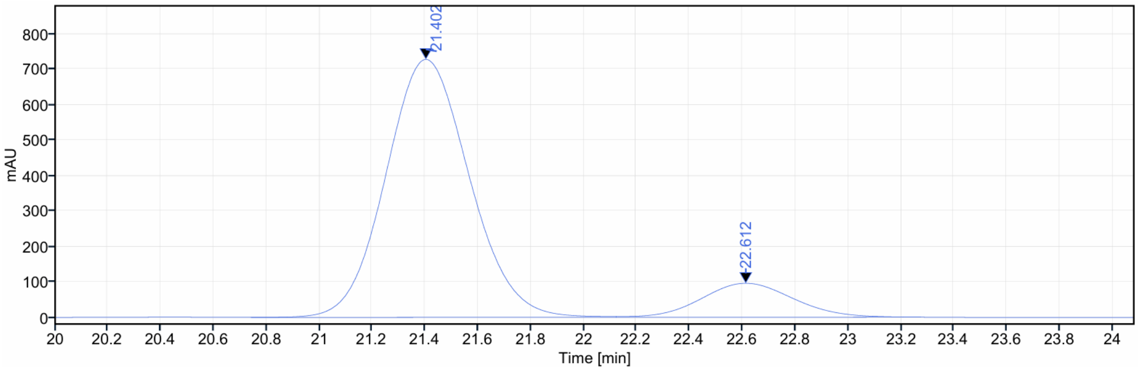

Signal: DAD1A,Sig=250,4 Ref=off

RT [min]	Type	Width [min]	Area	Height	Area%	Name
21.402	VM m	0.34	16002.67	728.23	87.35	
22.612	MM m	0.38	2317.09	95.89	12.65	

Chiral HPLC (Chiralpak® IA-3, Hexane/^{i}PrOH = 99:01, 0.3 mL/min) of **3c**

See procedure

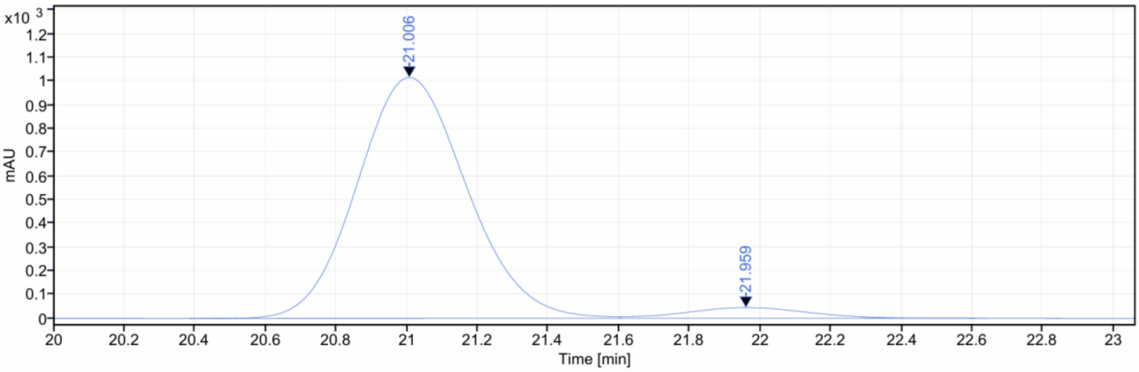

Signal: DAD1A,Sig=250,4 Ref=off

RT [min]	Type	Width [min]	Area	Height	Area%	Name
21.006	BM m	0.34	21969.16	1017.13	95.27	
21.959	MM m	0.37	1089.57	45.68	4.73	

Back to contents

Chiral HPLC (Chiralpak® IA-3, Hexane/^{i}PrOH = 99:01, 0.8 mL/min) of **3d**
See procedure

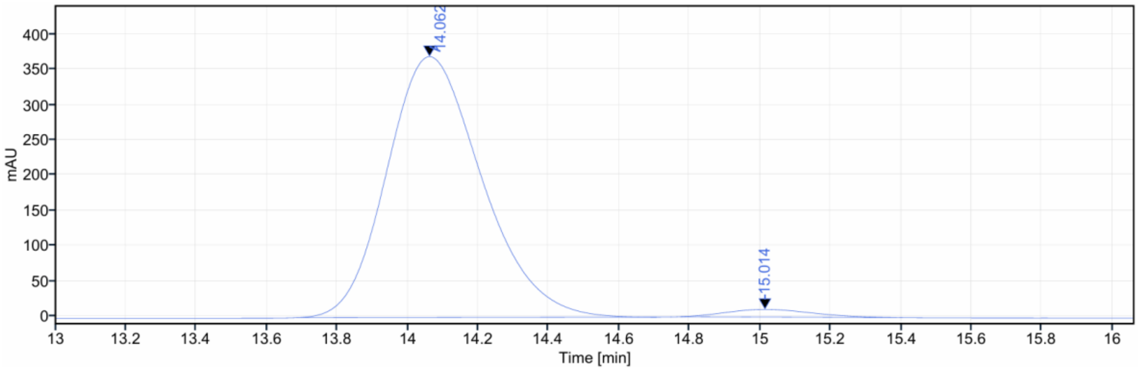

Signal: DAD1A,Sig=250,4 Ref=off

RT [min]	Type	Width [min]	Area	Height	Area%	Name
14.062	MM m	0.29	6932.41	369.70	97.41	
15.014	MM m	0.27	184.09	10.86	2.59	

Chiral HPLC (Chiralpak® IC-3, Hexane/^{i}PrOH = 93:07, 0.9 mL/min) of **3e**
See procedure

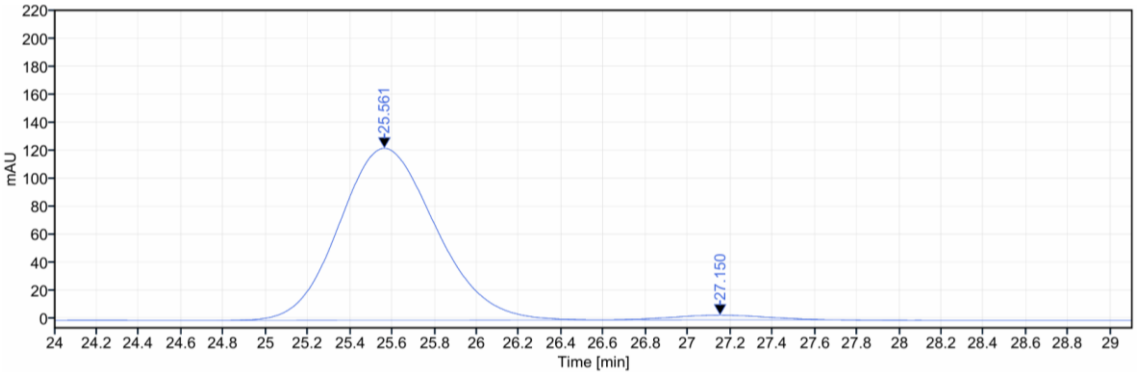

Signal: DAD1A,Sig=250,4 Ref=off

RT [min]	Type	Width [min]	Area	Height	Area%	Name
25.561	BM m	0.49	3898.38	122.47	97.33	
27.150	MM m	0.42	106.94	3.37	2.67	

Back to contents

Chiral HPLC (Chiralpak® IA-3, Hexane/^{i}PrOH = 99:01, 0.7 mL/min) of **3f**

See procedure

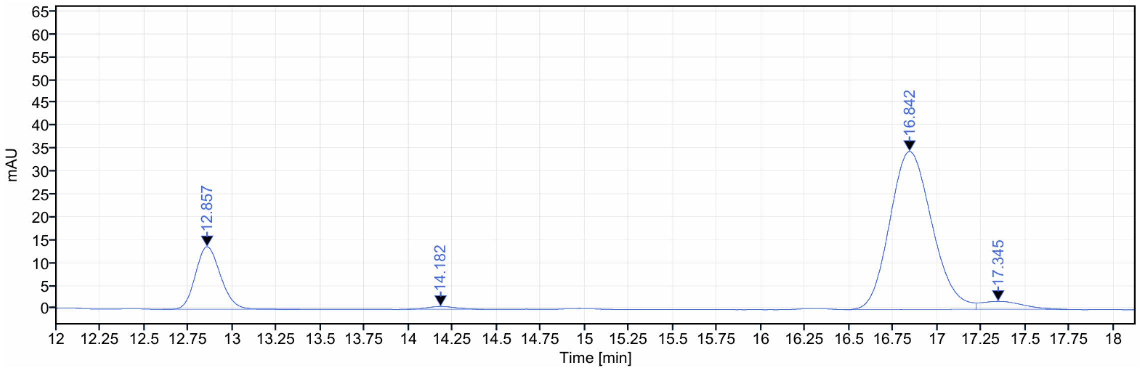

Signal: DAD1A,Sig=250,4 Ref=off

RT [min]	Type	Width [min]	Area	Height	Area%	Name
12.857	BB	0.78	134.78	13.68	18.08	
14.182	MM m	0.17	6.83	0.62	0.92	
16.842	VM m	0.26	574.95	34.55	77.13	
17.345	MM m	0.24	28.86	1.74	3.87	

Chiral HPLC (Chiralpak® IC-3, Hexane/^{i}PrOH = 93:07, 0.7 mL/min) of **3g**

See procedure

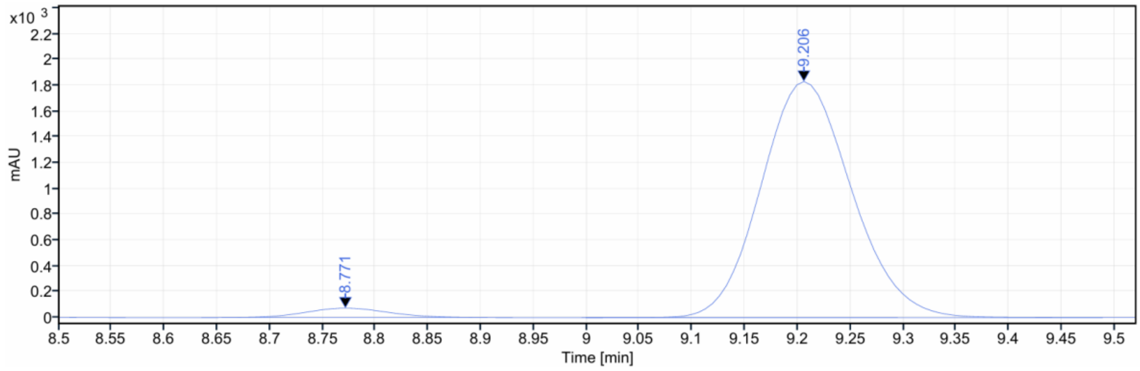

Signal: DAD1A,Sig=250,4 Ref=off

RT [min]	Type	Width [min]	Area	Height	Area%	Name
8.771	MM m	0.09	404.29	73.44	3.55	
9.206	MV m	0.09	10971.12	1833.55	96.45	

Back to contents

Chiral HPLC (Chiralpak® IC-3, Hexane/^{i}PrOH = 93:07, 0.7 mL/min) of **3h**

See procedure

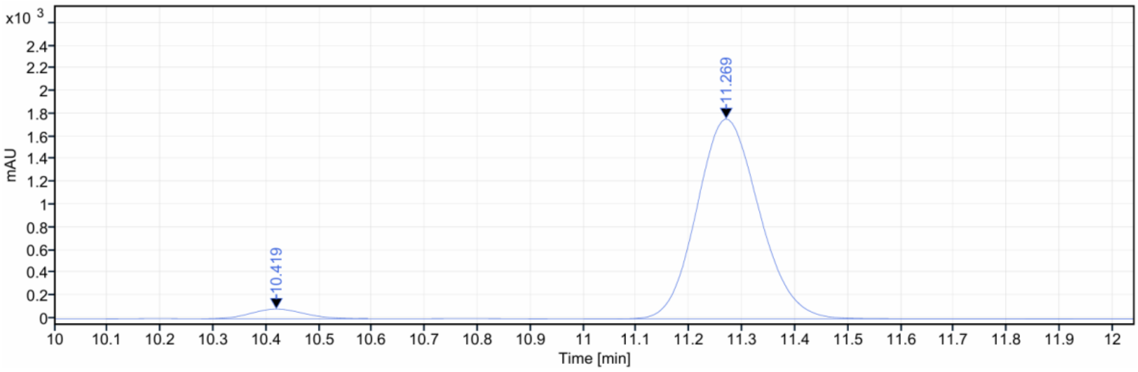

Signal: DAD1A,Sig=250,4 Ref=off

RT [min]	Type	Width [min]	Area	Height	Area%	Name
10.419	MM m	0.11	595.68	85.45	3.96	
11.269	MB m	0.13	14434.21	1761.56	96.04	

Chiral HPLC (Chiralpak® IC-3, Hexane/^{i}PrOH = 93:07, 0.7 mL/min) of **3i**

See procedure

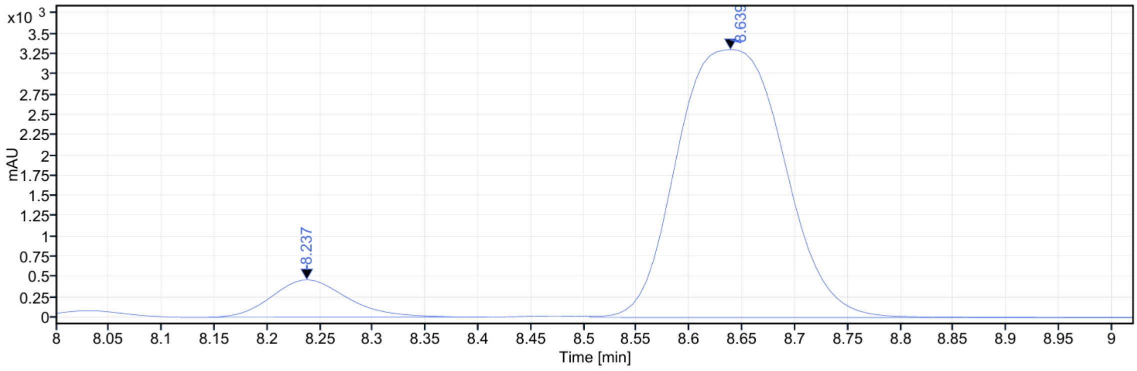

Signal: DAD1A,Sig=250,4 Ref=off

RT [min]	Type	Width [min]	Area	Height	Area%	Name
8.237	MM m	0.08	2285.20	461.24	8.98	
8.639	VB	0.53	23169.31	3303.75	91.02	

Back to contents

Chiral HPLC (Chiralpak® OD-3, Hexane/^{i}PrOH = 97:03, 0.9 mL/min) of **S5b**

See procedure

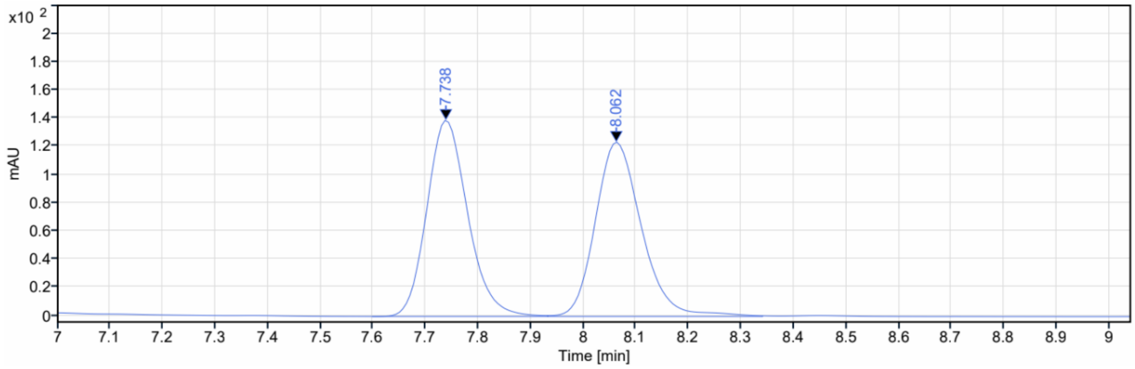

Signal: DAD1A,Sig=250,4 Ref=off

RT [min]	Type	Width [min]	Area	Height	Area%	Name
7.738	BM m	0.08	752.29	139.29	49.95	
8.062	MM m	0.09	753.65	123.73	50.05	

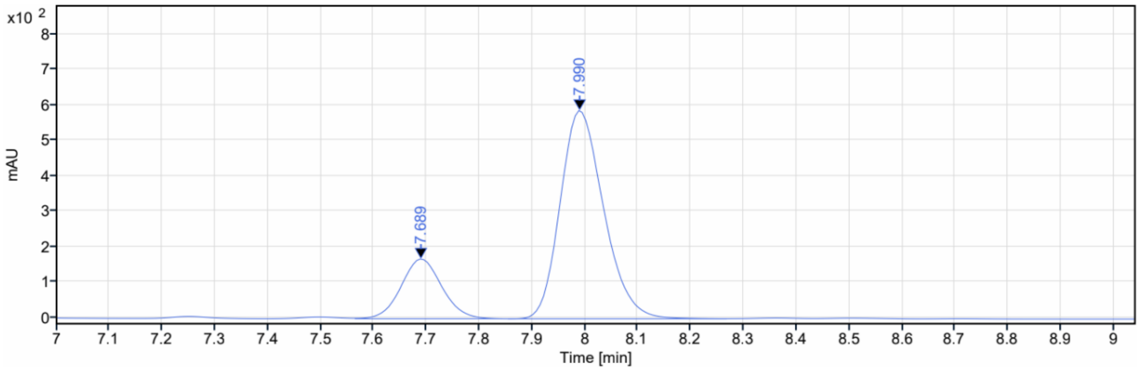

Signal: DAD1A,Sig=250,4 Ref=off

RT [min]	Type	Width [min]	Area	Height	Area%	Name
7.689	VM m	0.08	904.19	168.87	21.22	
7.990	MM m	0.09	3356.75	587.88	78.78	

Back to contents

Chiral HPLC (Chiralpak® IC-3, Hexane/^{i}PrOH = 97:03, 0.5 mL/min) of **4**
See procedure

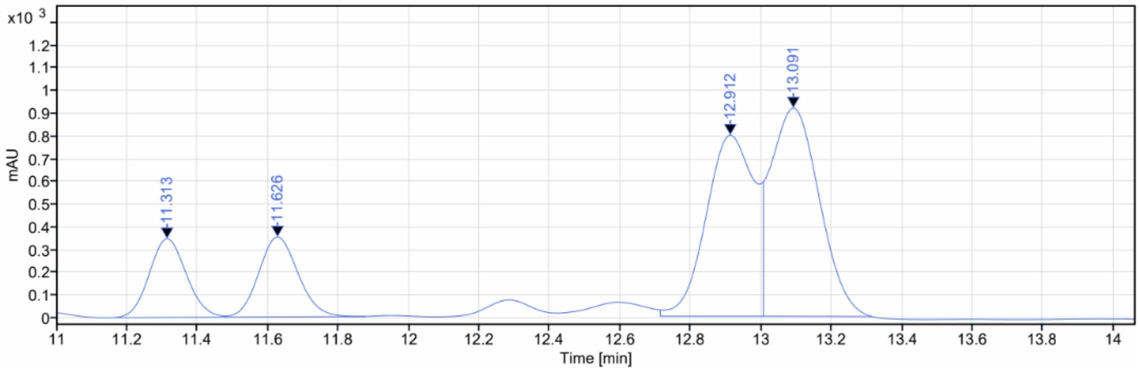

Signal: DAD1A,Sig=250,4 Ref=off

RT [min]	Type	Width [min]	Area	Height	Area%	Name
11.313	MM m	0.11	2523.51	346.44	11.70	
11.626	MM m	0.12	2720.58	351.87	12.61	
12.912	MM m	0.14	7607.52	797.44	35.26	
13.091	MM m	0.15	8724.54	916.95	40.44	

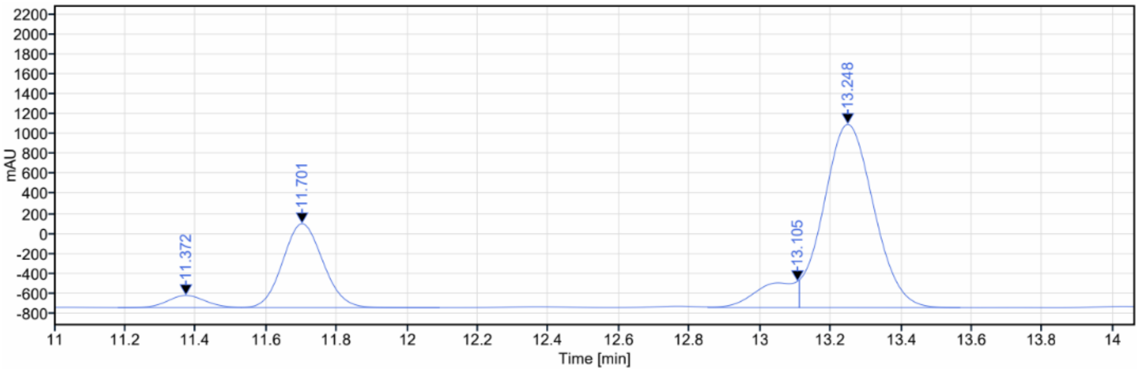

Signal: DAD1A,Sig=250,4 Ref=off

RT [min]	Type	Width [min]	Area	Height	Area%	Name
11.372	BV	0.35	914.75	122.61	3.29	
11.701	VB	0.56	6658.88	841.98	23.95	
13.105	MM m	0.13	2106.50	262.60	7.58	
13.248	MM m	0.15	18128.37	1837.95	65.19	

Back to contents

Chiral HPLC (Luna® CN, Hexane/^{i}PrOH = 99:01, 0.9 mL/min) of **5** and *iso*-**5**

See procedure

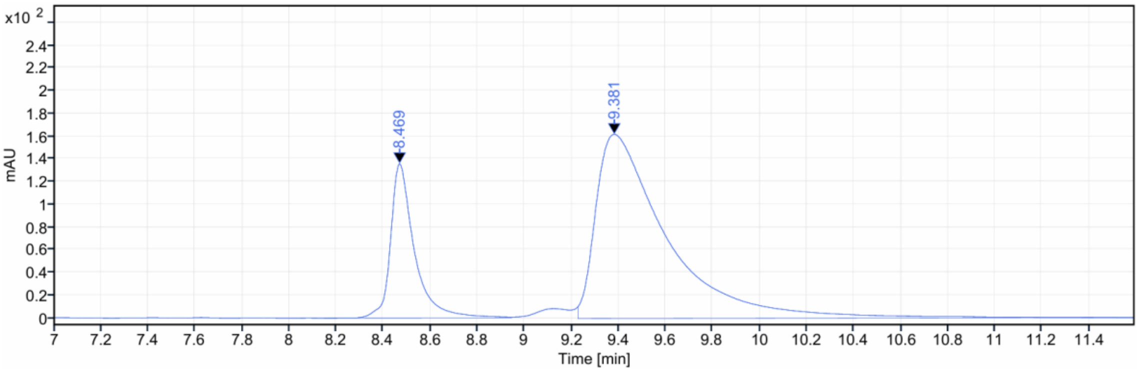

Signal: DAD1A,Sig=250,4 Ref=off

RT [min]	Type	Width [min]	Area	Height	Area%	Name
8.469	BV	0.80	956.87	136.03	21.93	
9.381	MB m	0.31	3405.94	162.18	78.07	

Chiral HPLC (Chiralpak® OJ-3, Hexane/^{i}PrOH = 90:10, 0.5 mL/min) of **5** and *iso*-**5**

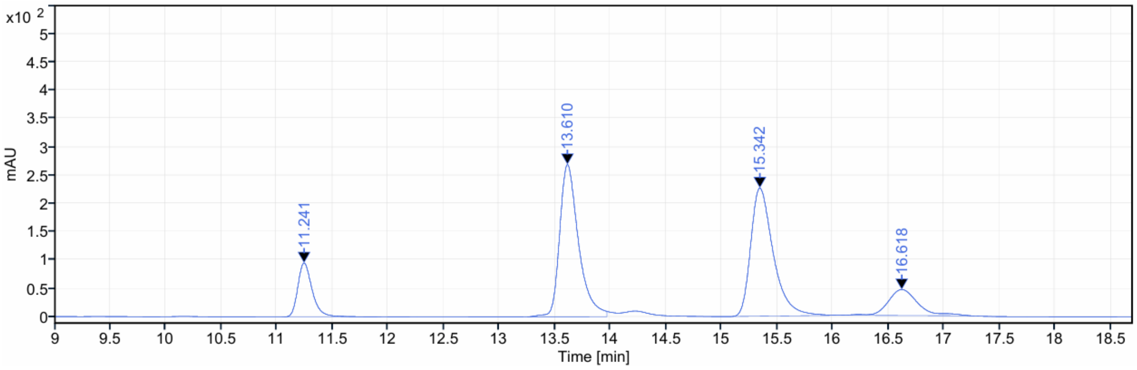

Signal: DAD1A,Sig=250,4 Ref=off

RT [min]	Type	Width [min]	Area	Height	Area%	Name
11.241	BB	0.89	859.54	95.35	10.64	
13.610	MM m	0.18	3178.74	268.94	39.36	
15.342	MM m	0.21	3183.59	226.42	39.42	
16.618	MM m	0.29	854.37	45.87	10.58	

Back to contents

Chiral HPLC (Chiralpak® OJ-3, Hexane/^{i}PrOH = 90:10, 0.5 mL/min) of **5**

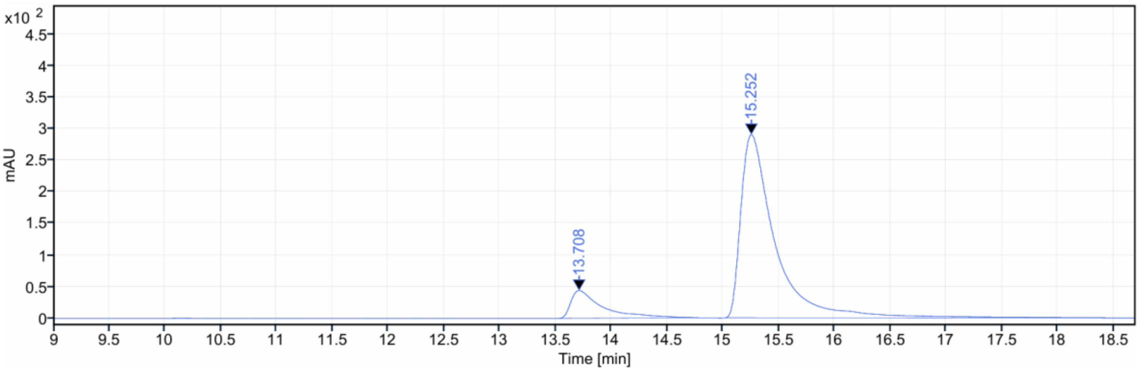

Signal:	DAD1A,Sig=250,4 Ref=off					
RT [min]	Type	Width [min]	Area	Height	Area%	Name
13.708	MM m	0.27	862.08	43.93	12.16	
15.252	MB m	0.31	6226.55	289.92	87.84	

Chiral HPLC (Chiralpak® OJ-3, Hexane/^{i}PrOH = 90:10, 0.5 mL/min) of *iso*-**5**

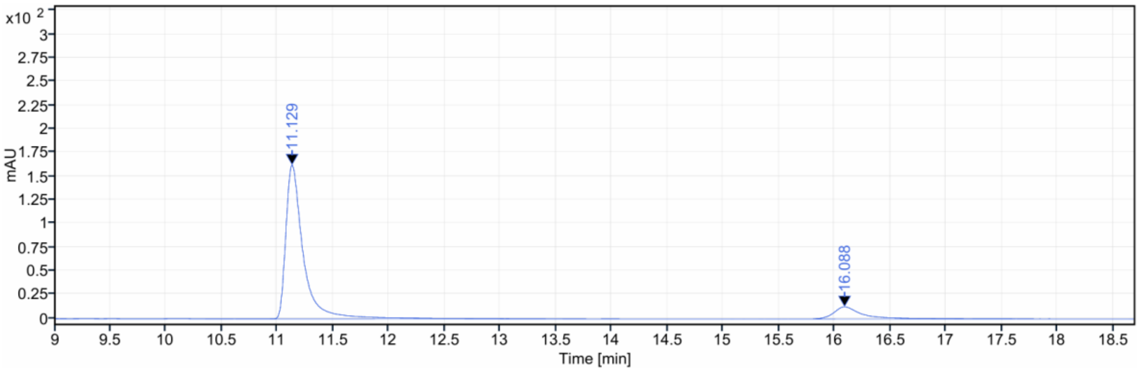

Signal:	DAD1A,Sig=250,4 Ref=off					
RT [min]	Type	Width [min]	Area	Height	Area%	Name
11.129	BB	2.47	1790.91	162.70	88.07	
16.088	BB	1.86	242.57	12.96	11.93	

Back to contents

Chiral HPLC (Chiralpak® IC-3, Hexane/^{i}PrOH = 90:10, 0.9 mL/min) of **7**
See procedure

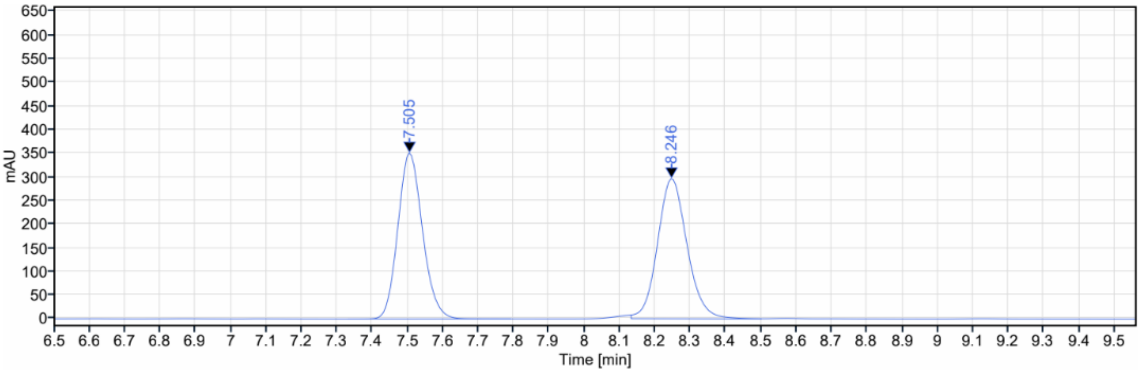

Signal: DAD1A,Sig=250,4 Ref=off

RT [min]	Type	Width [min]	Area	Height	Area%	Name
7.505	BM m	0.07	1697.07	351.47	49.60	
8.246	MV m	0.09	1724.36	296.92	50.40	

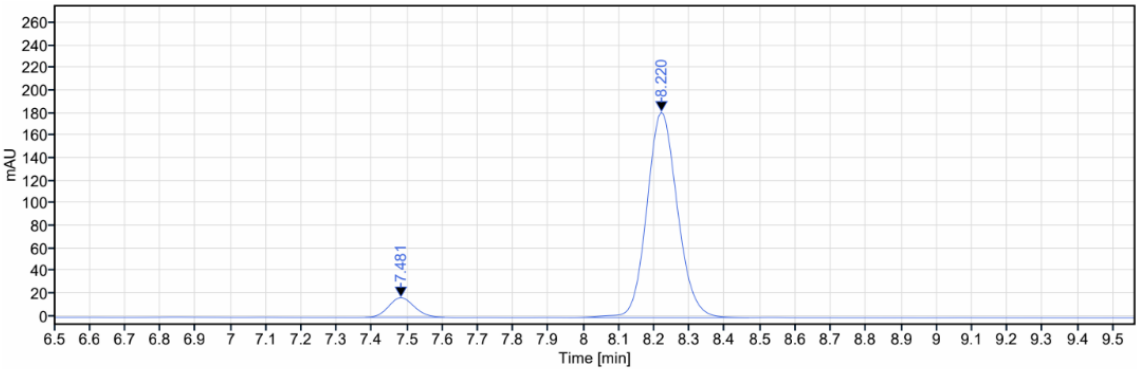

Signal: DAD1A,Sig=250,4 Ref=off

RT [min]	Type	Width [min]	Area	Height	Area%	Name
7.481	MM m	0.08	88.27	17.47	7.47	
8.220	BV	0.51	1093.82	182.15	92.53	

Back to contents

Chiral HPLC (Chiralpak® IC-3, Hexane/^{i}PrOH = 97:03, 0.5 mL/min) of **8**

See procedure

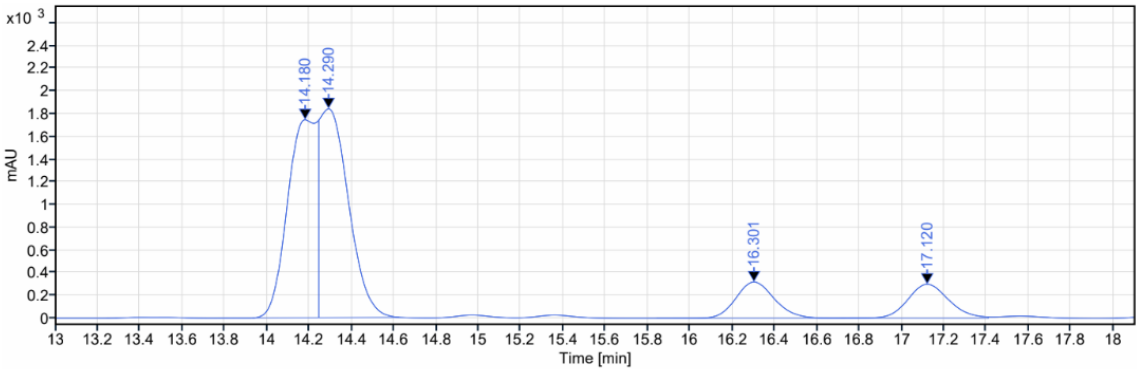

Signal: DAD1A,Sig=250,4 Ref=off

RT [min]	Type	Width [min]	Area	Height	Area%	Name
14.180	MM m	0.14	16716.66	1743.57	39.86	
14.290	MM m	0.15	17184.61	1839.27	40.97	
16.301	BM m	0.20	4022.00	319.10	9.59	
17.120	MM m	0.21	4018.75	298.96	9.58	

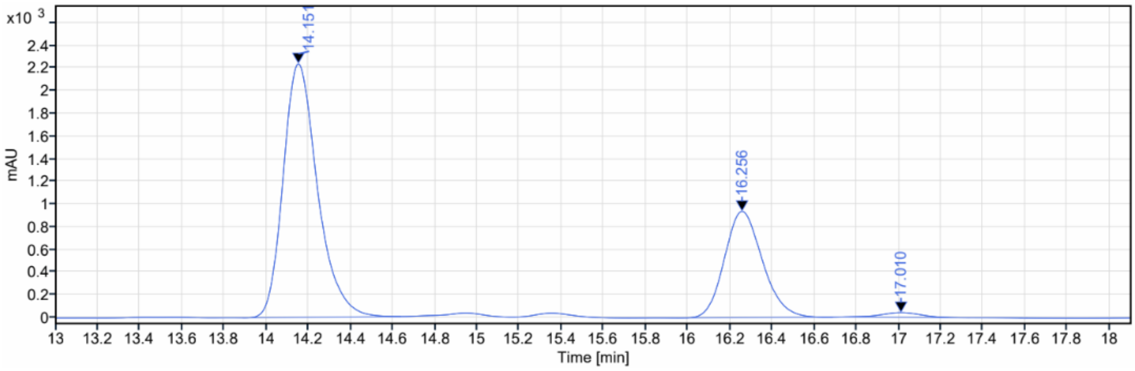

Signal: DAD1A,Sig=250,4 Ref=off

RT [min]	Type	Width [min]	Area	Height	Area%	Name
14.151	MM m	0.17	25344.78	2237.38	67.36	
16.256	MM m	0.19	11740.58	936.77	31.20	
17.010	MM m	0.20	540.38	41.80	1.44	

Back to contents

Chiral HPLC (Chiralpak® IC-3, Hexane/^{i}PrOH = 97:03, 0.9 mL/min) of **9**

See procedure

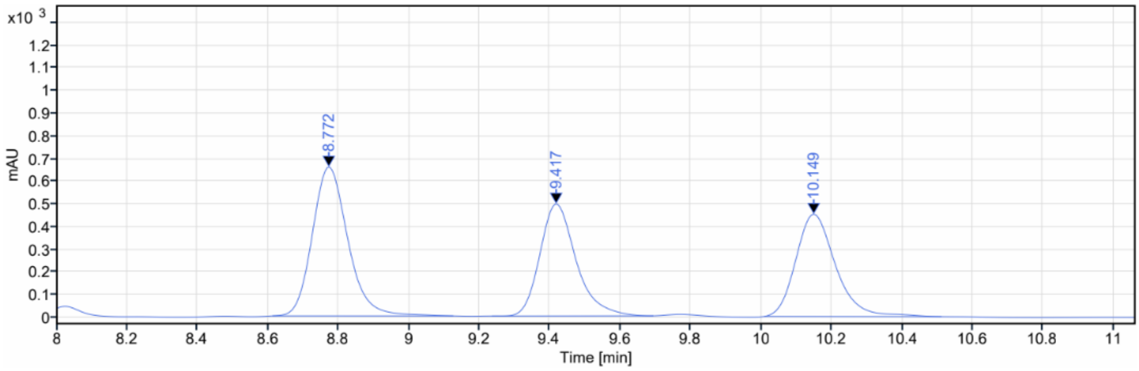

Signal: DAD1A,Sig=250,4 Ref=off

RT [min]	Type	Width [min]	Area	Height	Area%	Name
8.772	MM m	0.11	4588.94	657.22	39.44	
9.417	MM m	0.11	3520.60	494.38	30.26	
10.149	MM m	0.12	3524.30	451.56	30.29	

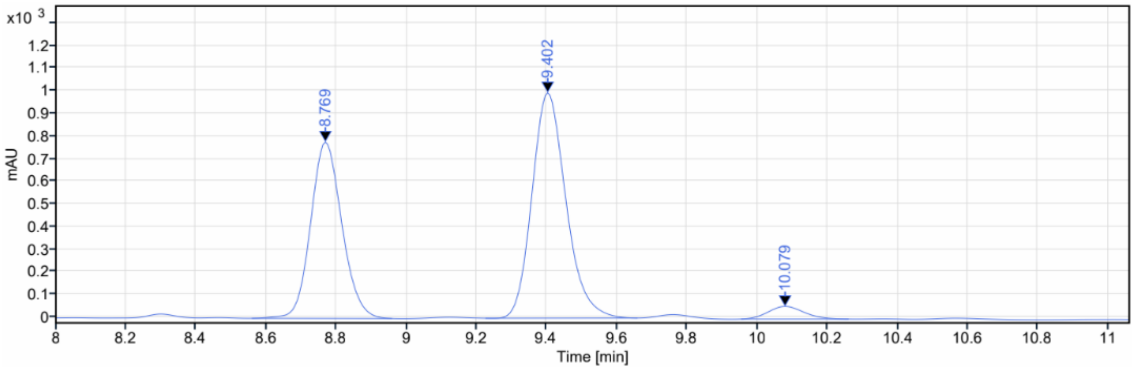

Signal: DAD1A,Sig=250,4 Ref=off

RT [min]	Type	Width [min]	Area	Height	Area%	Name
8.769	MM m	0.09	4813.10	780.02	40.43	
9.402	MM m	0.10	6693.87	996.79	56.23	
10.079	MM m	0.11	397.71	56.16	3.34	
