# Supplementary figures and images for: Photoenolization of α,β-Unsaturated Esters Enables Enantioselective Contra-Thermodynamic Positional Isomerization to α-Tertiary β,γ-Alkenyl Esters
Source: J Am Chem Soc. 2025 Feb 24;147(9):7452–60. doi: 10.1021/jacs.4c15732 (PMC11887454; doi:10.1021/jacs.4c15732)

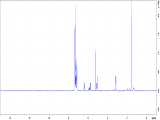

Supplement: Supplementary file 3 — ja4c15732_si_003.zip [file ja4c15732_si_003.zip › NMR raw data/1/1ad/1ad 1H 300 MHz/pdata/1/thumb.png]

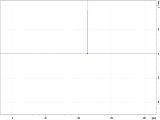

Supplement: Supplementary file 3 — ja4c15732_si_003.zip [file ja4c15732_si_003.zip › NMR raw data/1/1p/1p 19F 282 MHz/pdata/1/thumb.png]

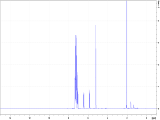

Supplement: Supplementary file 3 — ja4c15732_si_003.zip [file ja4c15732_si_003.zip › NMR raw data/1/1p/1p 1H 300 MHz/pdata/1/thumb.png]

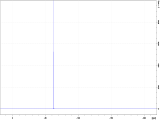

Supplement: Supplementary file 3 — ja4c15732_si_003.zip [file ja4c15732_si_003.zip › NMR raw data/1/1q/1q 19F 282 MHz/pdata/1/thumb.png]

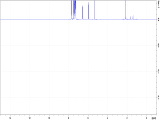

Supplement: Supplementary file 3 — ja4c15732_si_003.zip [file ja4c15732_si_003.zip › NMR raw data/1/1q/1q 1H 300 MHz/pdata/1/thumb.png]

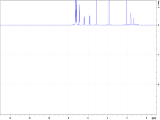

Supplement: Supplementary file 3 — ja4c15732_si_003.zip [file ja4c15732_si_003.zip › NMR raw data/1/1s/1s 1H 300 MHz/pdata/1/thumb.png]
