# Supplementary material for: Photoenolization of α,β-Unsaturated Esters Enables Enantioselective Contra-Thermodynamic Positional Isomerization to α-Tertiary β,γ-Alkenyl Esters
Source: J Am Chem Soc. 2025 Feb 24;147(9):7452–60. doi: 10.1021/jacs.4c15732 (PMC11887454; doi:10.1021/jacs.4c15732)

checkCIF/PLATON report

Structure factors have been supplied for datablock(s) 240203lt_auto

THIS REPORT IS FOR GUIDANCE ONLY. IF USED AS PART OF A REVIEW PROCEDURE FOR PUBLICATION, IT SHOULD NOT REPLACE THE EXPERTISE OF AN EXPERIENCED CRYSTALLOGRAPHIC REFEREE.

No syntax errors found. CIF dictionary Interpreting this report

Datablock: 240203lt_auto

Bond precision: C-C = 0.0041 A Wavelength=1.54184

Cell:	a=8.00935(6)	b=21.71113(19)	c=15.03384(11)
	alpha=90	beta=103.2966(7)	gamma=90
Temperature:	100 K		

	Calculated	Reported
Volume	2544.18(4)	2544.18(4)
Space group	P 21	P 1 21 1
Hall group	P 2yb	P 2yb
Moiety formula	C28 H28 N2 O6 S	C28 H28 N2 O6 S
Sum formula	C28 H28 N2 O6 S	C28 H28 N2 O6 S
Mr	520.58	520.58
Dx,g cm-3	1.359	1.359
Z	4	4
Mu (mm-1)	1.521	1.521
F000	1096.0	1096.0
F000’	1100.64	
h,k,lmax	9,25,17	9,25,17
Nref	9091[4677]	8703
Tmin,Tmax	0.849,0.913	0.925,1.000
Tmin’	0.846	

Correction method= # Reported T Limits: Tmin=0.925 Tmax=1.000
AbsCorr = MULTI-SCAN

Data completeness= 1.86/0.96 Theta(max)= 67.070

R(reflections)= 0.0309(8233) wR2(reflections)= 0.0789(8703)

S = 1.053 Npar= 670

The following ALERTS were generated. Each ALERT has the format
test-name_ALERT_alert-type_alert-level.
Click on the hyperlinks for more details of the test.

Alert level C

```
PLAT089_ALERT_3_C Poor Data / Parameter Ratio (Zmax < 18) ........       6.98 Note
PLAT340_ALERT_3_C Low Bond Precision on  C-C Bonds ...............    0.00409 Ang.
```

Alert level G

```
PLAT111_ALERT_2_G ADDSYM Detects New (Pseudo) Centre of Symmetry .         90 %Fit
PLAT113_ALERT_2_G ADDSYM Suggests Possible Pseudo/New  Space Group      P21/c Check
              Check Model Parameter Symmetry for Reflection Data Support
PLAT142_ALERT_4_G s.u. on b - Axis Small or Missing .............    0.00019 Ang.
PLAT143_ALERT_4_G s.u. on c - Axis Small or Missing .............    0.00011 Ang.
PLAT791_ALERT_4_G Model has Chirality at C16        (Sohncke SpGr)          S Verify
PLAT791_ALERT_4_G Model has Chirality at C53        (Sohncke SpGr)          S Verify
PLAT909_ALERT_3_G Percentage of I>2sig(I) Data at Theta(Max) Still        88% Note
PLAT969_ALERT_5_G The 'Henn et al.' R-Factor-gap value ..........      4.054 Note
              Predicted wR2: Based on SigI**2  1.95 or SHELX Weight  7.49
PLAT978_ALERT_2_G Number C-C Bonds with Positive Residual Density.         12 Info
```

0 **ALERT level A** = Most likely a serious problem - resolve or explain
0 **ALERT level B** = A potentially serious problem, consider carefully
2 **ALERT level C** = Check. Ensure it is not caused by an omission or oversight
9 **ALERT level G** = General information/check it is not something unexpected

0 ALERT type 1 CIF construction/syntax error, inconsistent or missing data
3 ALERT type 2 Indicator that the structure model may be wrong or deficient
3 ALERT type 3 Indicator that the structure quality may be low
4 ALERT type 4 Improvement, methodology, query or suggestion
1 ALERT type 5 Informative message, check

It is advisable to attempt to resolve as many as possible of the alerts in all categories. Often the minor alerts point to easily fixed oversights, errors and omissions in your CIF or refinement strategy, so attention to these fine details can be worthwhile. In order to resolve some of the more serious problems it may be necessary to carry out additional measurements or structure refinements. However, the purpose of your study may justify the reported deviations and the more serious of these should normally be commented upon in the discussion or experimental section of a paper or in the "special_details" fields of the CIF. checkCIF was carefully designed to identify outliers and unusual parameters, but every test has its limitations and alerts that are not important in a particular case may appear. Conversely, the absence of alerts does not guarantee there are no aspects of the results needing attention. It is up to the individual to critically assess their own results and, if necessary, seek expert advice.

Publication of your CIF in IUCr journals

A basic structural check has been run on your CIF. These basic checks will be run on all CIFs submitted for publication in IUCr journals (*Acta Crystallographica*, *Journal of Applied Crystallography*, *Journal of Synchrotron Radiation*); however, if you intend to submit to *Acta Crystallographica Section C* or *E* or *IUCrData*, you should make sure that full publication checks are run on the final version of your CIF prior to submission.

Publication of your CIF in other journals

Please refer to the *Notes for Authors* of the relevant journal for any special instructions relating to CIF submission.

PLATON version of 22/08/2024; check.def file version of 21/08/2024

Datablock 240203lt_auto - ellipsoid plot

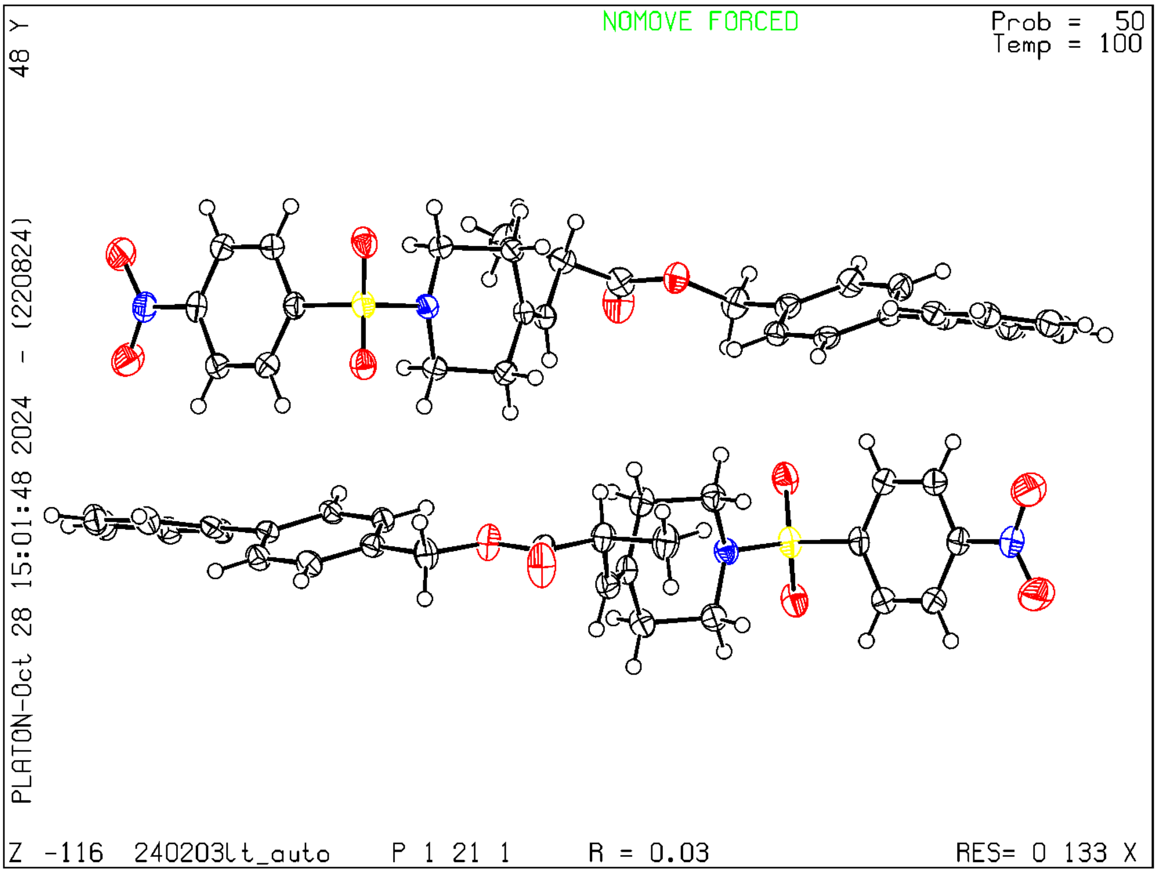

Supplement: Supplementary file 4 — ja4c15732_si_004.zip [file ja4c15732_si_004.zip › Crystallographic data/2j/checkcif/checkcif.pdf]
